# Convenient access to pyrrolidin-3-ylphosphonic acids and tetrahydro-2*H*-pyran-3-ylphosphonates with multiple contiguous stereocenters from nonracemic adducts of a Ni(II)-catalyzed Michael reaction

**DOI:** 10.3762/bjoc.16.174

**Published:** 2020-08-25

**Authors:** Alexander N Reznikov, Dmitry S Nikerov, Anastasiya E Sibiryakova, Victor B Rybakov, Evgeniy V Golovin, Yuri N Klimochkin

**Affiliations:** 1 Department of Organic Chemistry, Samara State Technical University, Molodogvardeyskaya str., 244, 443100 Samara, Russian Federation; 2 Department of Chemistry, Moscow State University, Leninskie Gory, 1, 119991, Mosсow, Russian Federation

**Keywords:** asymmetric catalysis, Michael addition, phosphonates, pyrrolidines, tetrahydropyranes

## Abstract

A new synthetic strategy toward nonracemic phosphoryl-substituted pyrrolidines and tetrahydropyranes with three and five contiguous stereocenters is presented. Readily available β-keto phosphonates react with conjugated nitroolefins in the presence of a chiral Ni(II) complex to give nitro keto phosphonates with two stereocenters with excellent enantioselectivity and moderate to high diastereoselectivity. These products were used for a reductive cyclization leading to pyrrolidin-3-ylphosphonic acid and for reactions with aldehydes yielding tetrahydropyranylphosphonates as individual stereoisomers. These nonracemic heterocycles containing phosphoryl moieties are useful for designing new pharmacologically active compounds.

## Introduction

**Figure 1:**
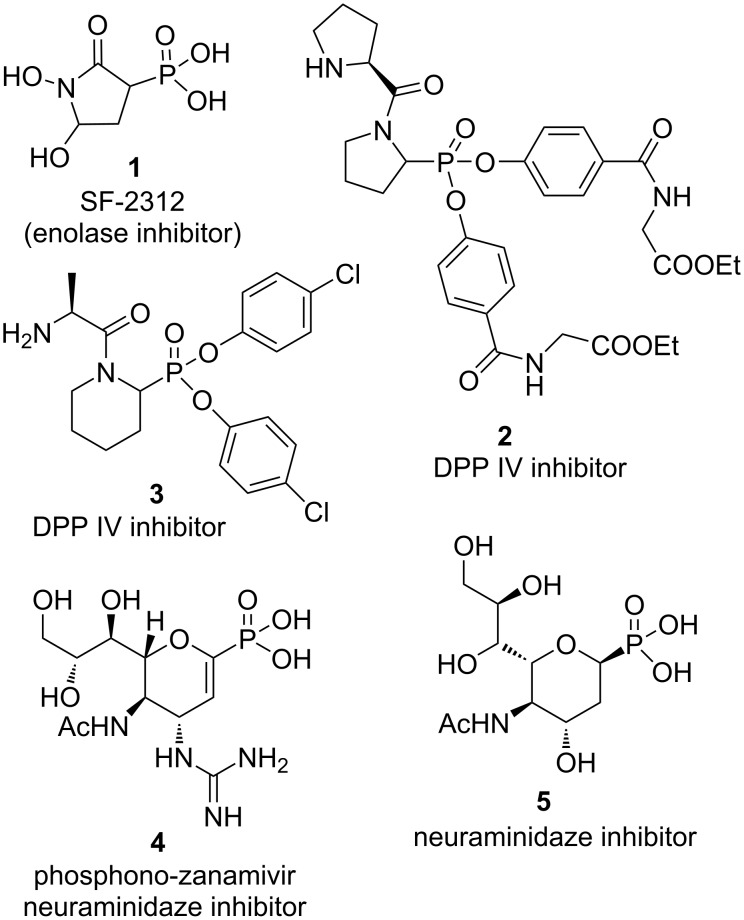
Pharmacologically active nonracemic phosphonates with heterocyclic moieties.

Сhiral phosphonates [1–2] and phosphoryl-substituted heterocycles [2–4] have received significant attention in recent years due to their wide range of biological activity. For example, SF-2312 (**1**) is a natural antibiotic – an enolase inhibitor produced by the actinomycete *Micromonospora* [5]. Dipeptide analogs with phosphonoproline **2** and piperidine-2-phosphonic acid **3** are potent inhibitors of dipeptidyl peptidase IV [6–7]. Oxygen-containing heterocycles containing a phosphoryl group are also of interest in the development of new drugs. It is known that phosphorylated carbohydrate analogues **4** and **5** are neuraminidase inhibitors (Figure 1) [8–9].

These circumstances create an interest in discovering synthetic routes for obtaining nonracemic phosphoryl-substituted heterocycles. Thus, in recent years, effective methods for the asymmetric synthesis of chiral phosphonates containing octahydroindole [10], tetrahydroquinoline [11], tetrahydroisoquinoline [11–12], β-carboline [13], morpholine [14], and isoindoline [15] moieties have been developed. Obtained in an enantiomerically pure form, phosphonoproline and its analogues [16–19] were among the first compounds in this series. Surprisingly, the methods for obtaining chiral pyrrolidin-3-ylphosphonic acids, tetrahydropyranylphosphonic acids and their esters have experienced minimal development [20–22]. The use of atom-efficient methods of asymmetric catalysis for the synthesis of such compounds is extremely relevant. The chiral metal-complexes-catalyzed [23–26] or organocatalyzed [27–32] Michael reaction is one of the most important synthetic tools for the asymmetric formation of a C–C bond. Moreover, the generation of the first stereocenter of a given configuration during the catalytic process leads to the possibility of creating enantiomerically pure compounds with several stereocenters through the directed regulation of diastereoselectivity in subsequent transformations, which can be realized as stepwise or cascade processes. Due to this reason, the Michael reaction was successfully used over the last decade to synthesize a wide range of heterocycles [33–44]. 2-Oxo-4-nitrophosphonates [45–46], the Michael adducts of β-keto phosphonates and nitroolefins, can be considered as versatile reagents for the synthesis of various phosphoryl-substituted heterocycles. The present work reports on the synthesis of nonracemic phosphoryl-substituted pyrrolidines and tetrahydropyranes via Ni(II)-catalyzed asymmetric Michael addition of β-keto phosphonates to conjugated nitroolefins.

## Results and Discussion

**Figure 2:**
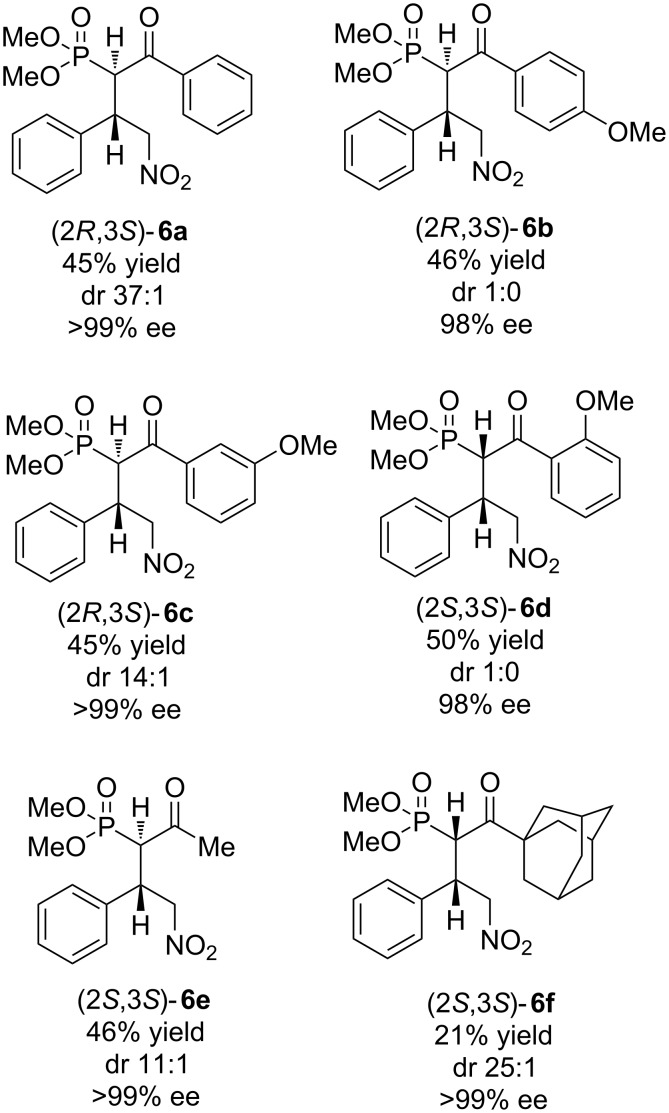
Starting nonracemic 4-nitro-2-oxophosphonates.

For the synthesis of nonracemic polysubstituted pyrrolidin-3-ylphosphonic acids and tetrahydropyranylphosphonates, we assumed that the 4-nitro-2-oxophosphonates – the Michael adducts of β-keto phosphonates and nitroolefins – are suitable precursors, considering that the Ni(II)-catalyzed Michael addition was carried out not only enantioselectively, but also diastereoselectively, such has been previously described [45]. These considerations prompted us to explore further applications of 4-nitro-2-oxophosphonates containing two contiguous stereocenters for the synthesis of the target compounds. Initially, 4-nitro-2-oxophosphonates **6a**–**f** (Figure 2) were synthesized similar to the reported procedure [45–46] from corresponding β-keto phosphonates and nitroolefins in the presence of 2 mol % of the Ni(II) complex with dr 11:1 to 1:0 and 98 –> 99% ee (see Supporting Information File 1).

**Scheme 1:**
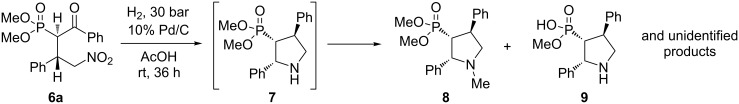
Intermolecular N-methylation of reduction product **7**.

A search for acceptable hydrogenation conditions was carried out using compound **6a**. Hydrogenation in the presence of Raney nickel or Pd/C in various solvents leads to a mixture of products, among which **8** and **9** are identified (Scheme 1).

A satisfactory yield of the desired product **7** (78% by ^31^P NMR of reaction mixture) was achieved when the hydrogenation was carried out in acetic acid in the presence of Pd/C. However, phosphonate **7** was unstable: already during its isolation, it was partially transformed into compounds **8** and **9**. It can be assumed that N-alkylation by the phosphoryl group occurs [47–49].

**Scheme 2:**
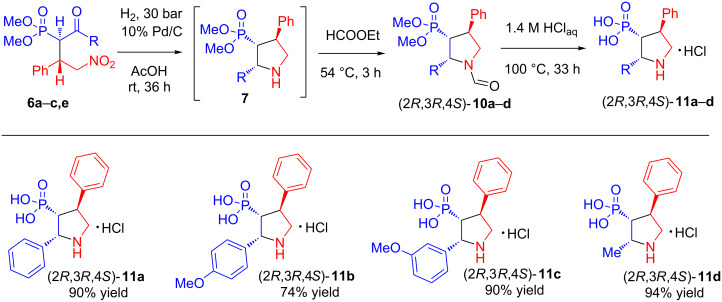
Synthesis of pyrrolidinyl phosphonic acids **11a**–**d**.

To avoid this undesirable process, the product **7** was formylated before its isolation. The formylated product **10a** may be purified by chromatography without decomposition. Subsequent acid hydrolysis of compound **10a** leads to pyrrolidinylphosphonic acid hydrochloride **11a** (Scheme 2).

Similarly, pyrrolidinylphosphonic acids **11b**–**d** were obtained from the corresponding phosphonates **6b,c,e** (Scheme 2).

**Figure 3:**
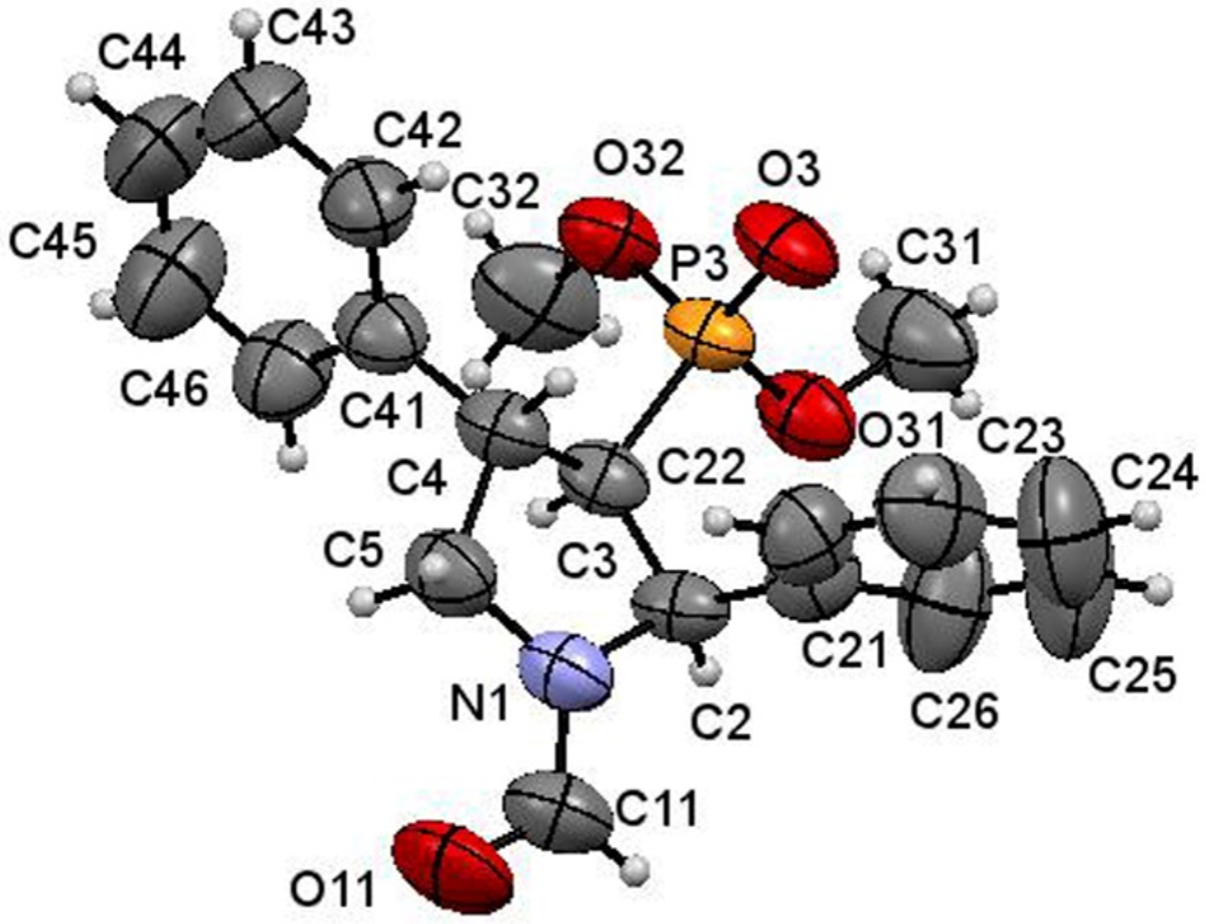
ORTEP diagram of (2*R*,3*R*,4*S*)-**10a**.

An X-ray diffraction study [50] of the formyl derivative **10a** showed its (2*R*,3*R*,4*S*)-configuration (Figure 3). The absolute configuration of the other phosphonates **10b**–**d** and **11b**–**d** is assumed by analogy.

Hydrogenation of phosphonates **6d,f** with *o*-anisyl and bulky adamantan-1-yl substituents at the carbonyl leads to a mixture of unidentified products.

At the next stage of this work, the Henry/acetalization reaction with phosphonate **6e** was studied. To optimize the reaction conditions various bases and solvents were used. The reaction of phosphonate **6e** with propionic aldehyde **12a** was used as a model reaction (see Table 1).

**Table 1:** Optimization of the conditions of Henry/acetalyzation reaction with phosphonate **6e**^a^.

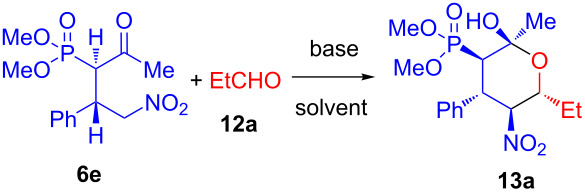					

Entry	Base	Phase transfer catalyst	Solvent	Yield of **13a** (%)	dr^b^

1	PhNMe_2_	–	THF	0	–
2	Et_3_N	–	THF	0^c^	–
3	DBU	–	THF	0^c^	–
4	K_3_PO_4_	TEBAC	THF	0	–
5	K_2_CO_3_	TEBAC	THF	0	–
6	KF	TEBAC	THF	0^c^	–
7	Cs_2_CO_3_	–	THF	0^c^	–
8	K_2_CO_3_	TEBAC	THF/H_2_O 1:1	0^c^	–
9	K_2_CO_3_	TEBAC	THF/H_2_O 7.5:1	34	1:0
10	K_2_CO_3_	18-crown-6	CH_3_CN	30	11:1
11	K_2_CO_3_	18-crown-6	THF	17	16:1
12	K_2_CO_3_	18-crown-6	CH_2_Cl_2_	12	10:1

^a^Reaction conditions: phosphonate **6e** (1.58 mmol), aldehyde **12a** (15.8 mmol), solvent (17 mL), base (1.58 mmol), phase transfer catalyst (0.158 mmol), rt, 72 h; ^b^determined by ^31^P NMR; ^c^full phosphonate **6e** conversion was observed and unidentified products were formed.

Initially, the catalytic ability of organic bases was studied for the Henry/acetalyzation reaction by using 0.1–1.0 equiv of catalyst in THF as a solvent. Unfortunately, no product formation was observed in the presence of *N*,*N*-dimethylaniline (Table 1, entry 1) and unidentified products were formed when stronger bases such as triethylamine or DBU were used (Table 1, entries 2 and 3). Later on, inorganic bases in combination with a phase transfer agent such as benzyltriethylammonium chloride (TEBAC) were tried as catalysts for the same reaction. Similarly, no products were detected in the reaction even after 72 h in the presence of 1 equiv of potassium phosphate or carbonate with TEBAC (0.1 equiv, Table 1, entries 4 and 5). If potassium fluoride or cesium carbonate were used as a base, the initial phosphonate **6e** was consumed in a few hours, but unidentified products were formed (Table 1, entries 6 and 7). After this, the reaction was carried out in an aqueous–organic medium. Fortunately, an individual diastereomer of Henry/acetalyzation product **13a** was obtained when K_2_CO_3_ in combination with TEBAC was used as a catalyst. A THF/water ratio of 7.5:1 is important in order to get the best yield (Table 1, entry 9). Lower yields and dr values for compound **13a** were obtained when 18-crown-6 was used as the phase-transfer catalyst (Table 1, entries 10–12).

**Scheme 3:**
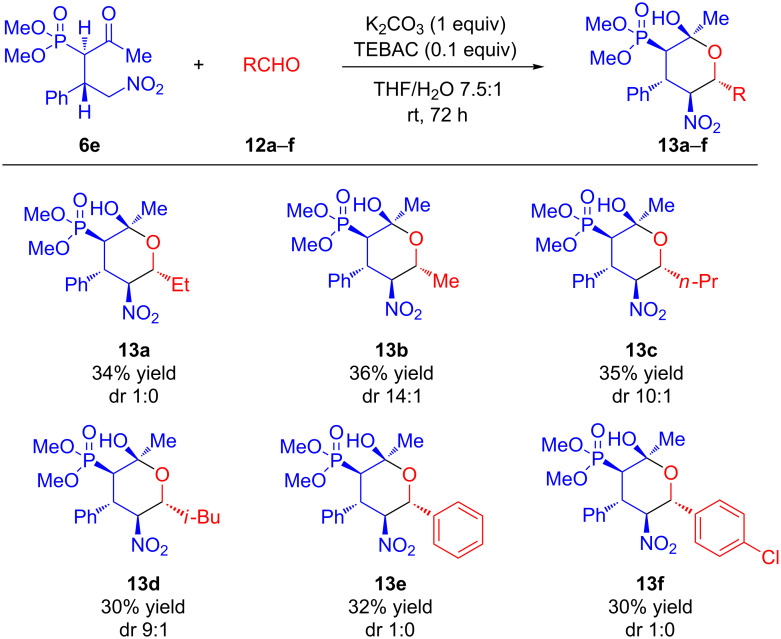
Synthesis of tetrahydropyranylphosphonates **13a**–**f** via diastereoselective Henry/acetalyzation reaction.

With the optimized reaction conditions in hand, the transformation was studied with a series of phosphonates and aldehydes. As shown in Scheme 3, the reaction of phosphonate **6e** gave moderate yields with various aliphatic and aromatic aldehydes **12a**–**f**. The presence of a bulkier (compared to methyl) group at the carbonyl in the phosphonates **6a**–**d,f** leads to no reaction.

Tetrahydropyranols **13a**–**f** were obtained with good diastereomeric purity. In the ^1^H NMR spectra of **13a**–**f** signals of methine groups with characteristic spin–spin coupling constants for axially located protons (12.1, 12.3 and 12.5 Hz) were observed.

**Figure 4:**
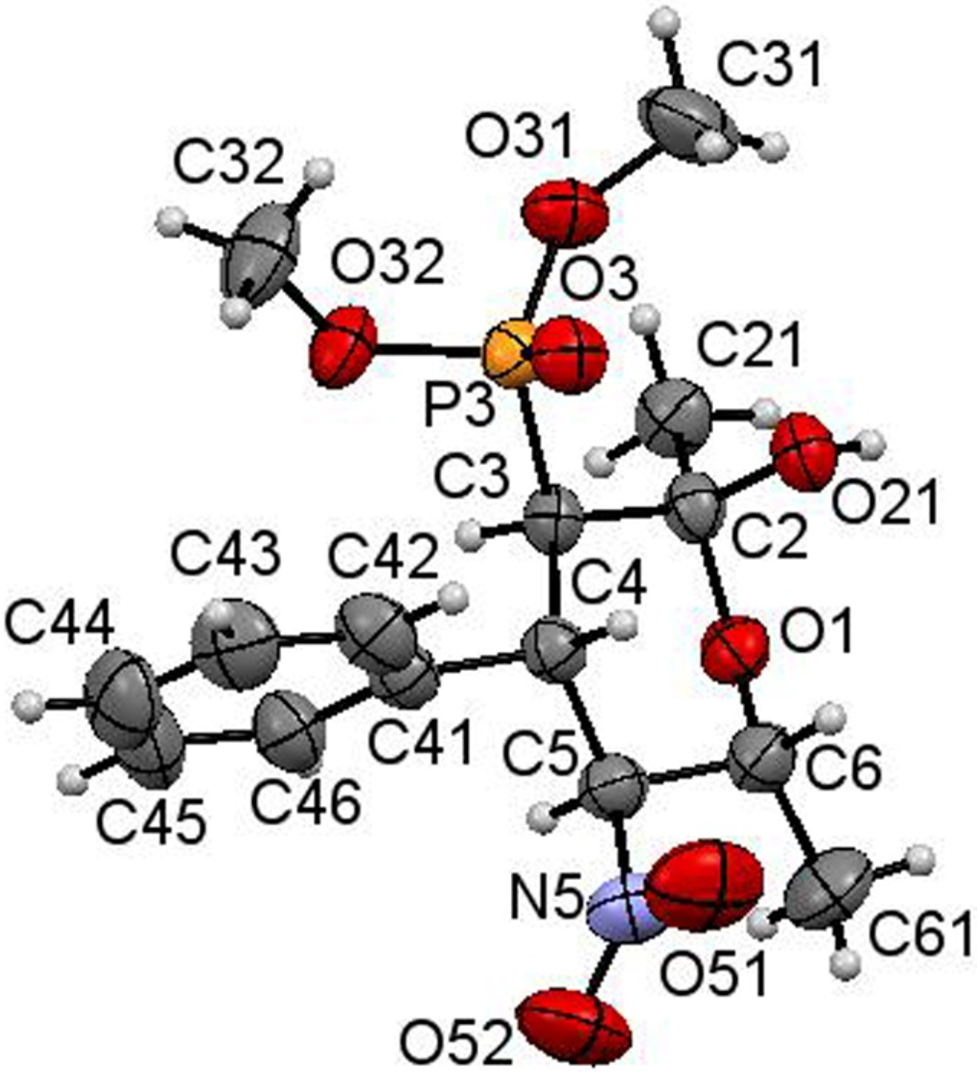
ORTEP diagram of (2*S*,3*R*,4*S*,5*S*,6*R*)-**13b**.

The structure and absolute (2*S*,3*R*,4*S*,5*S*,6*R*)-configuration of the tetrahydropyranol **13b** have been confirmed by single-crystal X-ray crystallographic analysis (Figure 4) [51].

All hydrogen atoms and the hydroxy group occupy an axial position, while the remaining substituents are in equatorial positions. This is consistent with the ^1^H NMR data for other similar tetrahydropyranols **13b**–**f**, therefore, their configuration was assumed by analogy.

**Scheme 4:**
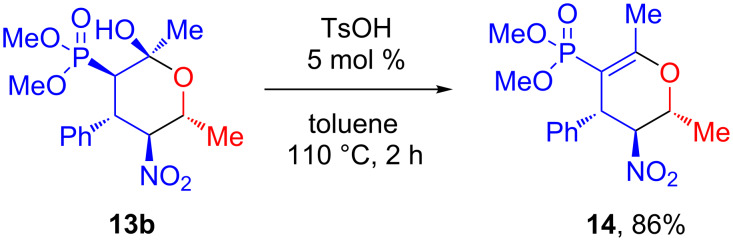
Synthesis of (3,4-dihydro-2*H*-pyran-5-yl)phosphonate **14**.

Dehydration of compound **13b** in the presence of *p*-toluenesulfonic acid gives dihydropyran **14** as a single stereoisomer (Scheme 4).

## Conclusion

In summary, a convenient protocol for the synthesis of phosphoryl-substituted pyrrolidines with three contiguous stereocenters via asymmetric addition of β-keto phosphonates to nitroolefins and subsequent reductive cyclization of enantioenriched, diastereomerically pure Michael adducts was developed. The present study also demonstrates that the diastereoselective cascade Henry/acetalyzation reaction with keto nitro phosphonates and aldehydes leading to tetrahydropyranylphosphonates can be efficiently performed within an aqueous-organic medium with K_2_CO_3_/TEBAC as the catalyst, and the corresponding products can be obtained with high diastereomeric purity. These methods enable the formation of highly enantioenriched phosphoryl-substituted heterocycles from readily available β-keto phosphonates using an inexpensive Ni(II) complex as the catalyst in the key step.

## Supporting Information

File 1 Experimental procedures, copies of NMR, FTIR, and mass spectra, HPLC and X-ray diffraction data.

File 2 Crystallographic information files (CIF).
