# Supplementary material for: Convenient access to pyrrolidin-3-ylphosphonic acids and tetrahydro-2H-pyran-3-ylphosphonates with multiple contiguous stereocenters from nonracemic adducts of a Ni(II)-catalyzed Michael reaction
Source: Beilstein J Org Chem. 2020 Aug 25;16:2073–9. doi: 10.3762/bjoc.16.174 (PMC7476589; doi:10.3762/bjoc.16.174)
Supplement: File 1 — Experimental procedures, copies of NMR, FTIR, and mass spectra, HPLC and X-ray diffraction data. [file Beilstein_J_Org_Chem-16-2073-s001.pdf]

BEILSTEIN JOURNAL OF ORGANIC CHEMISTRY

Supporting Information

for

Convenient access to pyrrolidin-3-ylphosphonic acids and tetrahydro-2*H*-pyran-3-ylphosphonates with multiple contiguous stereocenters from nonracemic adducts of a Ni(II)-catalyzed Michael reaction

Alexander N. Reznikov, Dmitry S. Nikerov, Anastasiya E. Sibiryakova, Victor B. Rybakov, Evgeniy V. Golovin and Yuri N. Klimochkin

Beilstein J. Org. Chem. **2020,** *16,* 2073–2079. doi:10.3762/bjoc.16.174

Experimental procedures, copies of NMR, FTIR, and mass spectra, HPLC and X-ray diffraction data

License and Terms: This is a supporting information file under the terms of the Creative Commons Attribution License (https://creativecommons.org/licenses/by/4.0). Please note that the reuse, redistribution and reproduction in particular requires that the authors and source are credited.
The license is subject to the *Beilstein Journal of Organic Chemistry* terms and conditions: (https://www.beilstein-journals.org/bjoc)

Table of contents

1. General information... S3

2. Synthesis of initial compounds.. S4

3. Catalysis.. S15

4. Synthesis of pyrrolidin-3-ylphosphonic acids.. S22

5. Synthesis of tetrahydro-2*H*-pyran-3-ylphosphonates............................ S33

6. Copies of NMR and FTIR spectra for initial compounds........................ S40

7. Copies of NMR, FTIR and mass spectra for phosphonates 6a–f........... S72

8. Copies of NMR, FTIR and mass spectra for compounds 7–14............. S92

9. Copies of HPLC chromatograms for compounds 6a–d,f and 13b......... S147

10. X-ray diffraction data of compounds 10a and 13b.............................. S155

References.. S161

1. General information

All commercially obtained reagents were used without further purification. All solvents were distilled prior to use. Dimethyl 2-oxopropylphosphonate **VIe** [1] and dimethyl 2-(adamantan-1-yl)-2-oxoethyl phosphonate **VIf** [2] were obtained according to reported procedures. Other β-keto phosphonates **Via–d** were synthesized following the modified Corbel's procedures [3] (see below). Bis[(1*R*,2*R*)-*N*,*N*'-dibenzylcyclohexane-1,2-diamine-κ^2*N*,*N*'](dibromo)nickel **VIII** was obtained according to a reported procedure [4]. Melting points were measured with an OptiMelt automated melting point system. All the reactions were monitored by TLC, performed on precoated silica gel plates (Sorbfil); compounds were made visible with I_2. ^{1}H, ^{13}C and ^{31}P NMR spectra were recorded with a JEOL JNM-ECX400 spectrometer at 399.78, 100.53 and 161.83 MHz, respectively, in $CDCl_3$, DMSO-d_6 and D_2O solution. FTIR spectra were recorded with a Shimadzu IR Affinity-1 spectrophotometer with Specac® Quest ATR. Elemental analysis was performed with an EuroVector EA-3000 analyzer. High-resolution mass spectrometry was recorded with an Agilent AccuTOF 6230 mass-spectrometer. Optical rotations were measured with Rudolph Research Analytical (Autopol V Plus Automatic Polarimeter). The enantiomeric purity of the products was determined by HPLC analysis on Shimadzu Prominence LC-20AD (Spd-20auv vis detector, Cto-20a column over, Dgu-20a degasing unit) equipped with a chiral stationary phase column (ChiralPAK AD-3) with hexane/2-propanol as eluent and with a chiral stationary phase column ChiralPAK AD-3R with water/acetonitrile as eluent. Column chromatography was performed on Silica gel 60, Merck (230–400 mesh). X-ray crystallographic data were collected using a Enraf-Nonius CAD-4 diffractometer.

2. Synthesis of initial compounds

The initial 4-nitro-2-oxophosphonates were synthesized according to scheme S1:

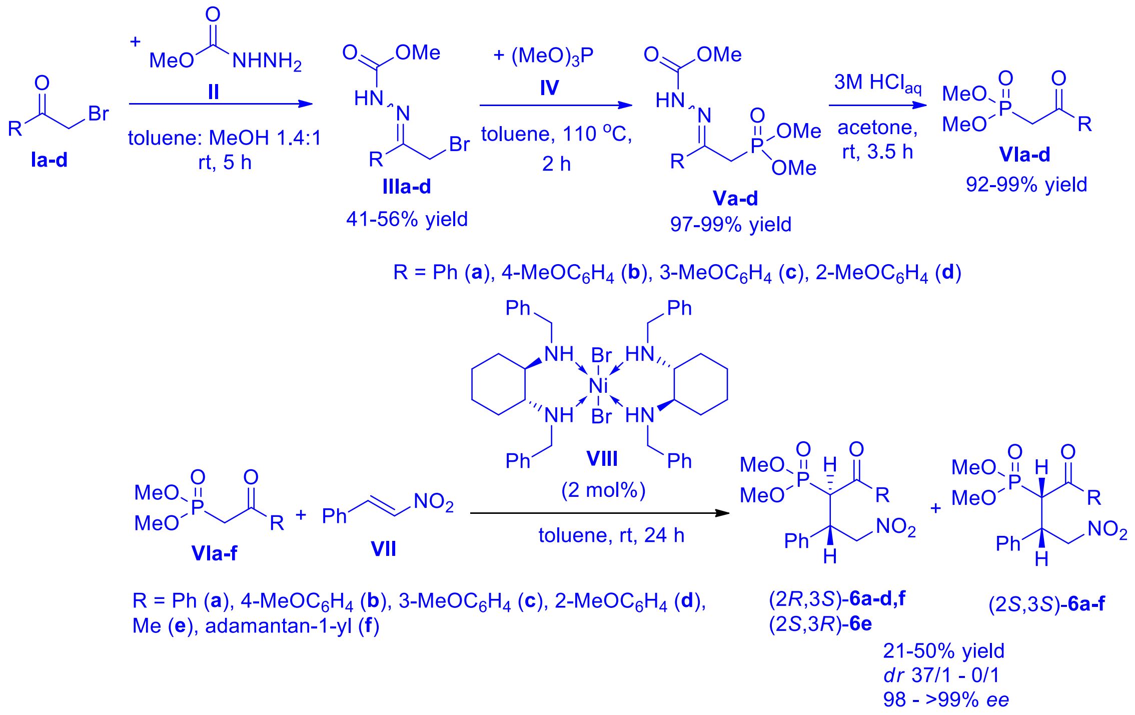

Scheme S1: General scheme for the synthesis of initial compounds.

General procedure for the synthesis of methyl 2-(2-bromo-1-arylethylidene)-hydrazine-1-carboxylates IIIa–d.

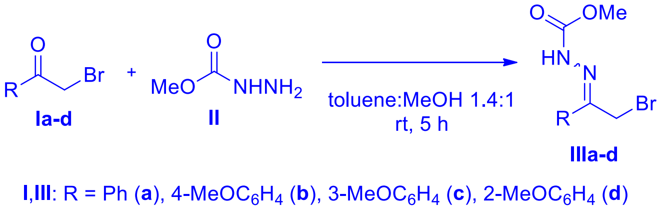

Scheme S2: Synthesis of methyl 2-(2-bromo-1-arylethylidene)hydrazine-1-carboxylates **IIIa–d**.

A solution of α-bromoketone **Ia-d** (93.8 mmol) and methylhydrazine carboxylate **II** (85.3 mmol) in toluene (84 mL) and methanol (59 mL) was stirred for 10 h at 25 °C. Then it was kept at −10 °C for 2 h. The precipitate was filtered off and recrystallized from acetone.

Methyl 2-(2-bromo-1-phenylethylidene)hydrazine-1-carboxylate (IIIa).

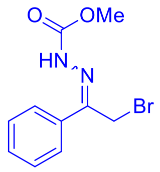

Mixture of isomers 2.2:1. Yield: 10.4 g (45%), white crystals, m.p. 130-131 °C (acetone).

^{1}H NMR (400 MHz, $CDCl_3$, 298 K) δ [ppm] 8.63 (br s, 1H, NH, minor isomer), 7.87 (br s, 1H, NH, major isomer), 7.72-7.71 (m, 1H, aromatic, major isomer), 7.50-7.42 (m, 2H, aromatic, major isomer, 2H, aromatic, minor isomer), 7.35-7.34 (m, 1H, aromatic, major isomer, 1H, aromatic, minor isomer), 7.27-7.25 (m, 1H, aromatic, major isomer, 2H, aromatic, minor isomer), 4.29 (s, 2H, CH_2, major isomer), 4.25 (s, 2H, CH_2, minor isomer), 3.83 (s, 3H, Me, minor isomer), 3.74 (s, 3H, Me, major isomer).

^{13}C NMR (101 MHz, $CDCl_3$, 298 K) δ [ppm] 154.5 (s, C=O, minor isomer), 153.8 (s, C=O, major isomer), 149.1 (s, C=N, major isomer), 145.7 (s, C=N, minor isomer), 135.4 (s, aromatic, C, major isomer), 134.0 (s, aromatic, C, minor isomer), 130.7 (s, aromatic, CH, minor isomer), 130.4 (s, aromatic, CH, major isomer), 129.8 (s, aromatic, 2CH, major isomer), 128.8 (s, aromatic, 2CH, minor isomer), 127.7 (s, aromatic, 2CH, major isomer), 126.3 (s, aromatic, 2CH, minor isomer), 53.4 (s, Me, major isomer), 53.2 (s, Me, minor isomer), 34.9 (s, CH_2, major isomer), 19.1 (s, CH_2, minor isomer).

Anal. Calcd. for $C_{10}H_{11}BrN_2O_2$: C, 44.30; H, 4.09; N 10.33. Found: C, 44.25; H, 4.14; N 10.37.

Methyl 2-[2-bromo-1-(4-methoxyphenyl)ethylidene]hydrazine-1-carboxylate (IIIb).

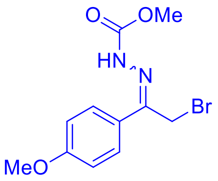

Mixture of isomers 2.0:1. Yield: 14.4 g (56%), white crystals, m.p. 128-129 °C (acetone).

^{1}H NMR (400 MHz, $CDCl_3$, 298 K) δ [ppm] 8.25 (br.s, 1H, NH, minor

isomer), 7.91 (br.s, 1H, NH, major isomer), 7.70-7.68 (m, 2H, aromatic, minor isomer), 7.25-7.22 (m, 2H, aromatic, major isomer), 7.01-6.99 (m, 2H, aromatic, major isomer), 6.90-6.87 (m, 2H, aromatic, minor isomer), 4.30 (s, 2H, CH_2, major isomer), 4.20 (s, 2H, CH_2, minor isomer), 3.86-3.78 (m, 6H, 2Me, minor isomer, 6H, 2Me, major isomer).

^{13}C NMR (101 MHz, $CDCl_3$, 298 K) δ [ppm] 161.1 (s, aromatic, C(OMe), minor isomer), 160.9 (s, aromatic, C(OMe), major isomer), 154.4 (s, C=O, minor isomer), 153.8 (s, C=O, major isomer), 149.1 (s, C=N, major isomer), 145.9 (s, C=N, minor isomer), 129.2 (s, aromatic, 2CH, major isomer), 127.8 (s, aromatic, 2CH, minor isomer), 122.5 (s, aromatic, C, minor isomer, C, major isomer), 115.3 (s, aromatic, 2CH, major isomer), 114.2 (s, aromatic, 2CH, minor isomer), 55.5 (s, 2Me, major isomer), 53.4 (s, 2Me, minor isomer), 35.2 (s, CH_2, major isomer), 18.9 (s, CH_2, minor isomer).

IR (ATR) ν [cm^{-1}] 3226 (w), 3170 (w), 3128 (w), 2956 (w), 2831 (w), 1707 (vs), 1604 (s), 1591 (s), 1514 (s), 1494 (s), 1469 (vs), 1413 (s), 1363 (vs), 1303 (vs), 1251 (vs), 1201 (s), 1178 (s), 1149 (s), 1066 (vs), 833 (vs), 812 (s), 796 (s), 686 (s), 607 (s), 555 (s), 534 (vs).

Anal. Calcd for $C_{11}H_{13}BrN_2O_3$: C, 43.87; H, 4.35; N 9.30. Found: C, 43.81; H, 4.39; N 9.39.

Methyl 2-[2-bromo-1-(3-methoxyphenyl)ethylidene]hydrazine-1-carboxylate (IIIc).

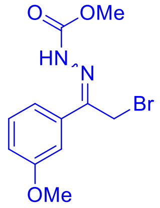

Mixture of isomers 1.9:1. Yield: 10.5 g (41%); white crystals; m.p. 129-130 °C (acetone).

^{1}H NMR (400 MHz, $CDCl_3$, 298 K) δ [ppm] 8.63 (br.s, 1H, NH, minor isomer), 7.93 (br.s, 1H, NH, major isomer), 7.41-7.25 (m, 2H, aromatic, minor isomer, 2H, major isomer), 6.97-6.77 (m, 2H, aromatic, minor isomer, 2H, major isomer), 4.28 (s, 2H, CH_2, major isomer), 4.23 (s, 2H, CH_2, minor isomer), 3.83 (s, 6H, 2Me, minor isomer), 3.79 (s, 6H, 2Me, major isomer).

^{13}C NMR (101 MHz, $CDCl_3$, 298 K) δ [ppm] 160.5 (s, aromatic, C(OMe), major isomer), 159.9 (s, aromatic, C(OMe), minor isomer), 154.5 (s, C=O, minor isomer), 153.8 (s, C=O, major isomer), 148.9 (s, C=N, major isomer), 145.6 (s, C=N, minor isomer), 136.9 (s, aromatic, C, minor isomer), 131.9 (s, aromatic, C, major isomer), 131.1 (s, aromatic, CH, major isomer), 129.7 (s, aromatic, CH, minor isomer), 119.6 (s, aromatic, CH, major isomer), 118.7 (s, aromatic, CH, minor isomer), 115.8 (s, aromatic, CH, major isomer), 115.6 (s, aromatic, CH, minor isomer), 113.2 (s, aromatic, CH, major isomer), 111.7 (s, aromatic, CH, minor isomer), 55.5 (s, 2Me, major isomer), 53.4 (s, 2Me, minor isomer), 34.8 (s, CH_2, major isomer), 19.2 (s, CH_2, minor isomer).

IR (ATR) ν [cm^{-1}] 3226 (w), 3124 (w), 2956 (w), 2831 (w), 1714 (vs), 1608 (s), 1591 (s), 1570 (s), 1469 (vs), 1429 (s), 1367 (vs), 1321 (s), 1292 (vs), 1222 (vs), 1155 (s), 1068 (vs), 1053 (vs), 1020 (vs), 856 (vs), 781 (vs), 682 (vs), 623 (s), 507 (s), 478 (s).

Anal. Calcd for $C_{11}H_{13}BrN_2O_3$: C, 43.87; H, 4.35; N 9.30. Found: C, 43.80; H, 4.43; N 9.36.

Methyl 2-[2-bromo-1-(2-methoxyphenyl)ethylidene]hydrazine-1-carboxylate (IIId).

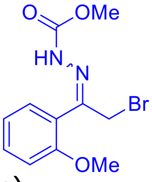

Yield: 11.8 g (46%), white crystals, m.p. 135-137 °C (acetone).

^{1}H NMR (400 MHz, $CDCl_3$, 298 K) δ [ppm] 7.74 (br.s, 1H, NH), 7.44-7.40 (m, 1H, aromatic), 7.16-7.14 (m, 1H, aromatic), 7.05-6.97 (m, 2H, aromatic), 4.33 (s, 2H, CH_2), 3.78 (s, 6H, 2Me).

^{13}C NMR (101 MHz, $CDCl_3$, 298 K) δ [ppm] 156.2 (s, aromatic, C(OMe)), 153.9 (s, C=O), 147.5 (s, C=N), 132.1 (s, aromatic, CH), 129.9 (s, aromatic, CH), 121.4 (s, aromatic, CH), 118.8 (s, aromatic, C), 111.9 (s, aromatic, CH), 55.8 (s, 2Me), 34.9 (s, CH_2).

IR (ATR) ν [cm^{-1}] 3226 (w), 3124 (w), 3026 (w), 2958 (w), 2837 (w), 1693 (vs), 1616 (m), 1597 (m), 1579 (m), 1471 (s), 1452 (s), 1367 (s), 1290 (m), 1273 (m), 1246 (vs), 1220 (s), 1132 (m), 1076 (vs), 1056 (s), 1024 (vs), 750 (vs), 690 (s), 559 (s), 518 (s).

Anal. Calcd for $C_{11}H_{13}BrN_2O_3$: C, 43.87; H, 4.35; N 9.30. Found: C, 43.81; H, 4.41; N 9.38.

General procedure for the synthesis of methyl 2-[2-(dimethoxyphosphoryl)-1-arylethylidene]hydrazinecarboxylates Va–d.

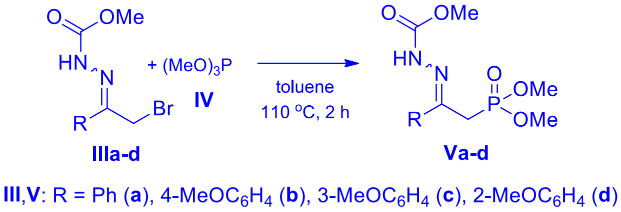

Scheme S3: Synthesis of methyl 2-[2-(dimethoxyphosphoryl)-1-arylethylidene]hydrazinecarboxylates **Va–d**

The resulting hydrazone **IIIa–d** (25.0 mmol) was added with stirring in an argon atmosphere for 1 h to a boiling solution of trimethyl phosphite **IV** (26.1 mmol) in toluene (25 mL). The mixture was stirred for 3 h, cooled and washed with water. The mixture was extracted with $CHCl_3$ (3 × 50 mL). The combined organic extracts were dried over Na_2SO_4, filtered, and evaporated in vacuo.

Methyl 2-[2-(dimethoxyphosphoryl)-1-phenylethylidene]hydrazine-1-carboxylate (Va).

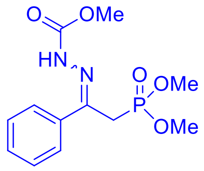

Yield: 7.28 g (97%), yellow solid, m.p. 107-108 °C (toluene).

^{1}H NMR (400 MHz, $CDCl_3$, 298 K) δ [ppm] 9.85 (br s, 1H, NH), 7.70-7.68 (m, 2H, aromatic), 7.32-7.20 (m, 3H, aromatic), 3.73 (s, 3H, Me), 3.62 (d, $^3J_{HP}$ = 11.2 Hz, 6H, 2MeO), 3.26 (d, $^2J_{HP}$ = 23.2 Hz, 2H, CH_2).

^{13}C NMR (101 MHz, $CDCl_3$, 298 K) δ [ppm] 155.5 (s, C=O), 143.8 (d, $^2J_{CP}$ = 10.5 Hz, C=N), 137.0 (d, $^3J_{CP}$ = 3.8 Hz, C), 129.6 (s, aromatic, CH), 128.5 (s, aromatic, 2CH),

126.6 (s, aromatic, 2CH), 53.6 (d, $^2J_{CP}$ = 6.7 Hz, MeO), 52.8 (s, Me), 27.2 (d, $^1J_{CP}$ = 137.1 Hz, CH_2).

^{31}P NMR (162 MHz, $CDCl_3$, 298 K) δ [ppm] 26.64.

Anal. Calcd for $C_{12}H_{17}N_2O_5P$: C, 48.00; H, 5.71; N 9.33. Found: C, 47.91; H, 5.79; N 9.36.

Methyl 2-(2-(dimethoxyphosphoryl)-1-(4-methoxyphenyl)ethylidene)hydrazine-1-carboxylate (Vb).

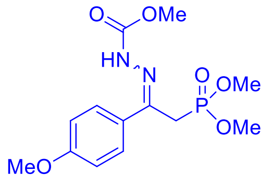

Mixture of isomers 8.0:1. Yield: 8.17 g (99%), yellow solid, m.p. 92-94 °C (toluene).

^1H NMR (400 MHz, $CDCl_3$, 298 K) δ [ppm] 9.75 (br.s, 1H, NH, major isomer, 1H, NH, minor isomer), 7.68-7.66 (m, 2H, aromatic, minor isomer, 2H, aromatic, major isomer), 6.82-6.80 (m, 2H, aromatic, minor isomer, 2H, aromatic, major isomer), 3.75 (s, 3H, Me, minor isomer, 3H, Me, major isomer), 3.74 (s, 3H, Me, minor isomer, 3H, Me, major isomer), 3.64 (d, $^3J_{HP}$ = 9.2 Hz, 6H, 2MeO, minor isomer, $^3J_{HP}$ = 9.2 Hz, 6H, 2MeO, major isomer), 3.24 (d, $^2J_{HP}$ = 22.7 Hz, 2H, CH_2, major isomer, $^2J_{HP}$ = 22.7 Hz, 2H, CH_2, minor isomer).

^{13}C NMR (101 MHz, $CDCl_3$, 298 K) δ [ppm] 160.9 (s, aromatic, C(OMe), major isomer), 160.6 (s, aromatic, C(OMe), minor isomer), 155.6 (s, C=O, minor isomer, C=O, major isomer), 144.0 (d, $^2J_{CP}$ = 10.5 Hz, C=N), 129.5 (d, $^3J_{CP}$ = 3.8 Hz, C, aromatic), 128.1 (s, aromatic, 2CH, minor isomer), 127.7 (s, aromatic, 2CH, major isomer), 115.0 (s, aromatic, 2CH, minor isomer), 113.9 (s, aromatic, 2CH, major isomer), 55.4 (s, Me, minor isomer, Me, major isomer), 53.6 (d, $^2J_{CP}$ = 4.7 Hz, MeO, major isomer, $^2J_{CP}$ = 4.7 Hz, MeO, minor isomer), 52.8 (s, Me, minor isomer, Me, major isomer), 35.8 (d, $^1J_{CP}$ = 138.0 Hz, CH_2, minor isomer), 27.1 (d, $^1J_{CP}$ = 136.0 Hz, CH_2, major isomer).

^{31}P NMR (162 MHz, $CDCl_3$, 298 K) δ [ppm] 26.80 (major isomer), 26.63 (minor isomer).

IR (ATR) ν [cm^{-1}] 3174 (w), 2958 (w), 2908 (w), 2852 (w), 2837 (w), 1737 (vs), 1610 (s), 1544 (s), 1510 (s), 1442 (s), 1398 (s), 1325 (s), 1240 (vs), 1174 (vs), 1018 (vs), 966 (s), 879 (s), 831 (s), 802 (s), 775 (s), 756 (s), 738 (s), 630 (s), 582 (s), 532 (s).

Anal. Calcd for $C_{13}H_{19}N_2O_6P$: C, 47.28; H, 5.80; N 8.48. Found: C, 47.21; H, 5.89; N 8.48.

Methyl 2-(2-(dimethoxyphosphoryl)-1-(3-methoxyphenyl)ethylidene)hydrazine-1-carboxylate (Vc).

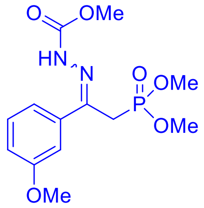

Mixture of isomers 7.1:1. Yield: 8.18 g (99%), yellow solid, m.p. 96-98 °C (toluene).

^{1}H NMR (400 MHz, $CDCl_3$, 298 K) δ [ppm] 9.89 (br.s, 1H, NH, major isomer), 7.88 (br.s, 1H, NH, minor isomer), 7.35-7.11 (m, 3H, aromatic, minor isomer, 3H, aromatic, major isomer), 6.96-6.76 (m, 1H, aromatic, minor isomer, 1H, aromatic, major isomer), 3.77 (s, 6H, 2Me, minor isomer, 6H, 2Me, major isomer), 3.68 (d, $^3J_{HP}$ = 11.2 Hz, 6H, 2MeO, minor isomer, $^3J_{HP}$ = 11.2 Hz, 6H, 2MeO, major isomer), 3.27 (d, $^2J_{HP}$ = 22.0 Hz, 2H, CH_2, major isomer, $^2J_{HP}$ = 22.0 Hz, 2H, CH_2, minor isomer).

^{13}C NMR (101 MHz, $CDCl_3$, 298 K) δ [ppm] 160.5 (s, aromatic, C(OMe), minor isomer), 159.8 (s, aromatic, C(OMe), major isomer), 155.5 (s, C=O, minor isomer, C=O, major isomer), 143.7 (d, $^2J_{CP}$ = 10.5 Hz, C=N, minor isomer, $^2J_{CP}$ = 10.5 Hz, C=N, major isomer), 138.5 (d, $^3J_{CP}$ = 3.8 Hz, C, minor isomer, $^3J_{CP}$ = 3.8 Hz, C, major isomer), 129.5 (s, aromatic, CH, major isomer), 129.1 (s, aromatic, CH, minor isomer), 119.1 (s, aromatic, CH, major isomer), 119.0 (s, aromatic, CH, minor isomer), 115.6 (s, aromatic, CH, major isomer, aromatic, CH, minor isomer), 111.9 (s, aromatic, CH, major isomer, aromatic, CH, minor isomer), 55.4 (s, Me, minor isomer, Me, major isomer), 53.7 (d, $^2J_{CP}$ = 5.7 Hz, MeO), 52.9 (s, Me), 35.6 (d, $^1J_{CP}$ = 138.0 Hz, CH_2, minor isomer), 27.5 (d, $^1J_{CP}$ = 137.1 Hz, CH_2, major isomer).

^{31}P NMR (162 MHz, $CDCl_3$, 298 K) δ [ppm] 26.65 (major isomer), 26.45 (minor isomer).

IR (ATR) ν [cm^{-1}] 3217 (w), 3003 (w), 2954 (w), 2852 (w), 1743 (s), 1720 (s), 1598 (m), 1575 (m), 1531 (s), 1487 (s), 1448 (s), 1431 (s), 1359 (m), 1220 (vs), 1182 (s), 1024 (vs), 974 (s), 910 (s), 860 (s), 806 (s), 786 (s), 727 (vs), 688 (s).

Anal. Calcd for $C_{13}H_{19}N_2O_6P$: C, 47.28; H, 5.80; N 8.48. Found: C, 47.28; H, 5.80; N 8.48.

Methyl 2-(2-(dimethoxyphosphoryl)-1-(2-methoxyphenyl)ethylidene)hydrazine-1-carboxylate (Vd).

Mixture of isomers 2.7:1. Yield: 8.17 g (99%), yellow solid, m.p. 89-91 °C (toluene).

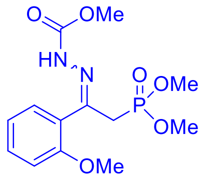

^{1}H NMR (400 MHz, $CDCl_3$, 298 K) δ [ppm] 9.92 (br.s, 1H, NH, major isomer), 7.73 (br.s, 1H, NH, minor isomer), 7.38-6.81 (m, 4H, aromatic, minor isomer, 4H, aromatic, major isomer), 3.76 (s, 6H, 2Me, minor isomer, 6H, 2Me, major isomer), 3.60 (d, $^3J_{HP}$ = 11.1 Hz, 6H, 2MeO, minor isomer, $^3J_{HP}$ = 11.1 Hz, 6H, 2MeO, major isomer), 3.39 (d, $^2J_{HP}$ = 22.4 Hz, 2H, CH_2, major isomer, $^2J_{HP}$ = 22.4 Hz, 1H, CH_2, minor isomer), 3.22 (d, $^2J_{HP}$ = 22.4 Hz, 1H, CH_2, minor isomer).

^{13}C NMR (101 MHz, $CDCl_3$, 298 K) δ [ppm] 157.1 (s, aromatic, C(OMe), major isomer), 156.2 (s, C=O, minor isomer), 155.9 (s, aromatic, C(OMe), minor isomer), 155.5 (s, C=O, major isomer), 145.7 (d, $^2J_{CP}$ = 10.5 Hz, C=N), 131.7 (s, aromatic, CH, minor isomer), 131.1 (s, aromatic, CH, major isomer), 130.9 (s, aromatic, CH, major isomer), 129.0 (s, aromatic, CH, minor isomer), 127.6 (s, aromatic, C, major isomer), 121.3 (s, aromatic, CH, minor isomer), 120.9 (s, aromatic, CH, major isomer), 120.7 (s, aromatic, C, minor isomer), 111.9 (s, aromatic, CH, minor isomer), 110.9 (s, aromatic, CH, major isomer), 55.7 (s, Me, minor isomer), 55.5 (s, Me, major isomer), 53.2 (d, $^2J_{CP}$ = 5.7 Hz, MeO, major isomer), 52.8 (s, Me, minor isomer, major isomer), 52.7 (d, $^2J_{CP}$ = 5.7 Hz, MeO, minor isomer), 34.9 (d, $^1J_{CP}$ = 138.0 Hz, CH_2, minor isomer), 29.5 (d, $^1J_{CP}$ = 134.2 Hz, CH_2, major isomer).

^{31}P NMR (162 MHz, $CDCl_3$, 298 K) δ [ppm] 27.55 (major isomer), 27.00 (minor isomer).

IR (ATR) ν [cm^{-1}] 3211 (w), 2999 (w), 2953 (w), 1747 (s), 1720 (s), 1598 (m), 1579 (w), 1490 (s), 1458 (s), 1436 (s), 1359 (m), 1303 (m), 1236 (vs), 1220 (vs), 1020 (vs), 968 (s), 879 (s), 842 (s), 802 (s), 752 (vs).

Anal. Calcd for $C_{13}H_{19}N_2O_6P$: C, 47.28; H, 5.80; N 8.48. Found: C, 47.28; H, 5.80; N 8.48.

General procedure for the synthesis of β-keto phosphonates Via–d.

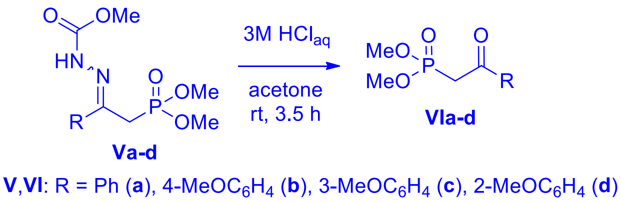

Scheme S4: Synthesis of β-keto phosphonates **VIa–d**

Hydrochloric acid (3M, 12 mL) was added to a solution of methyl 2-[2-(dimethoxyphosphoryl)-1-arylethylidene]hydrazinecarboxylate **Va–d** (20 mmol) in acetone (12 mL). The resulting mixture was stirred for 3.5 h at rt, then the acetone was removed in vacuo and the residue was extracted with $CHCl_3$ (3 × 50 mL). The combined organic layers were dried (Na_2SO_4) and evaporated to give the β-keto phosphonates.

Dimethyl (2-oxo-2-phenylethyl)phosphonate (VIa).

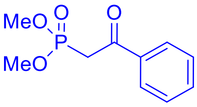

Yield: 2.26 g (99%); pale yellow oil.

^{1}H NMR (400 MHz, $CDCl_3$, 298 K) δ [ppm] 7.93-7.90 (m, 2H, Ph), 7.53-7.48 (m, 1H, Ph), 7.41-7.36 (m, 2H, Ph), 3.68 (d, $^{3}J_{HP}$ = 11.2 Hz, 6H, CH_3O), 3.57 (d, $^{2}J_{HP}$ = 22.7 Hz, 2H, CH_2).

^{13}C NMR (101 MHz, $CDCl_3$, 298 K) δ [ppm] 191.8 (s, C=O), 136.4 (s, C, Ph), 133.8 (s, CH, Ph), 129.0 (s, CH, Ph), 128.7 (s, CH, Ph), 53.2 (d, CH_3O, $^2J_{CP}$ = 1.9 Hz), 37.5 (d, CH_2, $^1J_{CP}$ = 130.6 Hz).

^{31}P NMR (162 MHz, $CDCl_3$, 298 K) δ [ppm] 23.44.

Anal. Calcd for $C_{10}H_{13}O_4P$: C, 52.64; H, 5.74. Found: C, 52.58; H, 5.81.

Dimethyl [2-(4-methoxyphenyl)-2-oxoethyl]phosphonate (VIb).

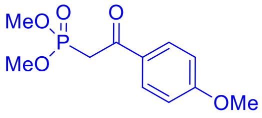

Yield: 2.45 g (95%); pale yellow oil.

^1H NMR (400 MHz, $CDCl_3$, 298 K) δ [ppm] 7.75 (d, $^3J_{HH}$ = 9.0 Hz, 2H, aromatic), 6.71 (d, $^3J_{HH}$ = 9.0 Hz, 2H, aromatic), 3.63 (s, 3H, CH_3O), 3.54 (d, $^3J_{HP}$ = 11.2 Hz, 6H, CH_3O), 3.39 (d, $^2J_{HP}$ = 22.7 Hz, 2H, CH_2).

^{13}C NMR (101 MHz, $CDCl_3$, 298 K) δ [ppm] 190.0 (d, $^2J_{CP}$ = 5.7 Hz, C=O), 164.0 (s, C, aromatic), 131.3 (s, CH, aromatic), 129.3 (d, $^3J_{CP}$ = 2.9 Hz , C, aromatic), 113.8 (s, CH, aromatic), 54.4 (s, CH_3O), 53.5 (d, $^2J_{CP}$ = 5.7 Hz, CH_3O), 36.5 (d, $^1J_{CP}$ = 129.6 Гц, CH_2).

^{31}P NMR (162 MHz, $CDCl_3$, 298 K) δ [ppm] 23.87.

IR (ATR) ν [cm^{-1}] 3007 (w), 2955 (w), 2845 (w), 1667 (s), 1597 (s), 1574 (m), 1512 (m), 1460 (m), 1421 (m), 1315 (m), 1283 (m), 1250 (vs), 1173 (s), 1134 (m), 1113 (m), 1053 (s), 1020 (vs), 989 (vs), 878 (m), 829 (s), 799 (vs), 721 (m), 644 (w), 631 (w), 567 (s), 517 (s).

Anal. Calcd for $C_{11}H_{15}O_5P$: C, 51.17; H, 5.86. Found: C, 51.07; H, 5.89.

Dimethyl [2-(3-methoxyphenyl)-2-oxoethyl]phosphonate (VIc).

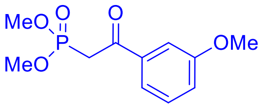

Yield: 2.45 g (95%); pale yellow oil.

^1H NMR (400 MHz, $CDCl_3$, 298 K) δ [ppm] 7.55-7.44 (m, 1H, CH), 7.38-7.31 (m, 2H, CH), 7.02-6.95 (m, 1H, CH), 3.67 (s, 3H, CH_3O), 3.52 (d, $^3J_{HP}$ = 11.2 Hz, 6H, CH_3O), 3.36 (d, $^2J_{HP}$ = 22.7 Hz, 2H, CH_2).

^{13}C NMR (101 MHz, $CDCl_3$, 298 K) δ [ppm] 192.5 (d, $^2J_{CP}$ = 5.8 Hz, C=O), 161.2 (s, C, aromatic), 137.1 (s, C, aromatic), 133.1 (s, CH, aromatic), 123.6 (s, CH, aromatic),

121.3 (s, CH, aromatic), 113.0 (s, CH, aromatic), 54.3 (s, CH_3O), 53.3 (d, $^2J_{CP}$ = 5.7 Hz, CH_3O), 36.2 (d, $^1J_{CP}$ = 129.6 Hz, CH_2).

^{31}P NMR (162 MHz, $CDCl_3$, 298 K) δ [ppm] 23.87.

IR (ATR) ν [cm^{-1}] 2999 (w), 2954 (w), 2853 (w), 1678 (s), 1597 (m), 1584 (m), 1487 (m), 1450 (m), 1429 (m), 1252 (vs), 1182 (s), 1128 (w), 1022 (vs), 864 (s), 841 (s), 791 (vs), 746 (vs), 685 (m), 665 (m), 596 (w), 542 (m), 517 (s).

Anal. Calcd for $C_{11}H_{15}O_5P$: C, 51.17; H, 5.86. Found: C, 51.11; H, 5.93.

Dimethyl [2-(2-methoxyphenyl)-2-oxoethyl]phosphonate (VId).

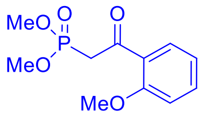

Yield: 2.38 g (92%), pale yellow oil.

^{1}H NMR (400 MHz, $CDCl_3$, 298 K) δ [ppm] 7.69 (dd, $^3J_{HH}$ = 7.3 Hz, $^4J_{HH}$ = 1.6 Hz, 1H, aromatic), 7.45 (dt, $^3J_{HH}$ = 7.9 Hz, $^4J_{HH}$ = 1.8 Hz, 1H, aromatic), 6.98-6.92 (m, 2H, aromatic), 3.89 (s, 3H, CH_3O), 3.78 (d, $^2J_{HP}$ = 21.5 Hz, 2H, CH_2), 3.70 (d, $^3J_{HP}$ = 11.2 Hz, 6H, CH_3O).

^{13}C NMR (101 MHz, $CDCl_3$, 298 K) δ [ppm] 193.4 (d, $^2J_{CP}$ = 6.7 Hz, C=O), 158.8 (s, C, aromatic), 134.6 (s, CH, aromatic), 131.0 (s, CH, aromatic), 127.4 (d, $^3J_{CP}$ = 3.4 Hz, C, aromatic), 120.9 (s, CH, aromatic), 111.7 (s, CH, aromatic), 55.7 (s, CH_3O), 52.9 (d, $^2J_{CP}$ = 1.9 Hz, CH_3O), 41.2 (d, $^1J_{CP}$ = 131.6 Hz, CH_2).

^{31}P NMR (162 MHz, $CDCl_3$, 298 K) δ [ppm] 24.79.

IR (ATR) ν [cm^{-1}] 3003 (w), 2953 (w), 2851 (w), 1667 (m), 1597 (m), 1578 (w), 1485 (m), 1464 (m), 1437 (m), 1395 (w), 1296 (s), 1242 (vs), 1182 (m), 1163 (m), 1144 (w), 1101 (w), 1018 (vs), 1001 (vs), 876 (m), 843 (m), 800 (s), 756 (vs), 731 (s), 700 (m), 662 (w), 586 (m), 513 (m).

Anal. Calcd for $C_{11}H_{15}O_5P$: C, 51.17; H, 5.86. Found: C, 51.09; H, 5.92.

3. Catalysis

General procedure for catalytic asymmetric Michael addition of β-keto phosphonates to ω-nitrostyrene in the presence of a Ni(II) complex.

A mixture of β-keto phosphonate **VIa–f** (8.10 mmol), ω-nitrostyrene **VII** (8.90 mmol) and bis[(1*R*,2*R*)-*N*,*N*'-dibenzylcyclohexane-1,2-diamine-κ^2*N*,*N*'](dibromo)nickel **VIII** (0.16 mmol) in 10 mL of toluene was stirred at rt for 24 h. The formed crystalline product was filtered and recrystallized from appropriate solvent (MeOH for **6a**,**d**,**e**,**f**; EtOH for **6c**; toluene for **6b**) to give an individual diastereomer.

Table S1: Asymmetric Michael addition of β-keto phosphonates to ω-nitrostyrene in the presence of bis[(1*R*,2*R*)-*N*,*N*'-dibenzylcyclohexane-1,2-diamine-κ^2*N*,*N*'](dibromo)nickel

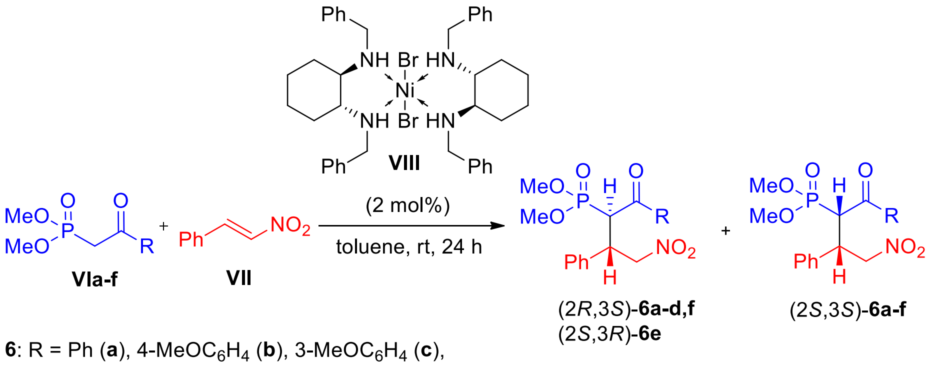

Entry	R	Compd.	Conv., %	dr[a]	ee[a], %	Yield[b], %	dr[c]	ee[c], %
1[d]	Ph	**6a**	90	37/1	>99	45	37/1	>99
2	4-MeOC$_6$H$_4$	**6b**	92	13/1	97	46	1/–	98
3	3-MeOC$_6$H$_4$	**6c**	87	12/1	86	45	14/1	>99
4	2-MeOC$_6$H$_4$	**6d**	82	1/2	83	50	–/1	98
5	Me	**6e**	86	1.2/1	ND[e]	46	11/1	>99[f]
6[d]	1-Ad	**6f**	81	1/1.8	85	21	1/25	>99

[a]dr (by ^{31}P NMR) and ee (by chiral HPLC) in reaction mixture; [b]isolated yield; [c]dr (by ^{31}P NMR) and *ee* (by chiral HPLC) after crystallization; [d]previously reported data [2,5]; [e]not determined; [f]The enantiomeric excess for compound **6e** could not be determined by HPLC due to overlapping peaks of the corresponding enantiomers in the chromatograms. For this reason, the value of the *ee* was determined on the basis of HPLC analysis of its derivative - compound **13b** (see below).

The absolute (2*R*,3*S*)-configuration of compound **6a** was determined earlier by X-ray diffraction analysis [5]. The absolute configurations of other Michael adducts **6b–f** were assumed by analogy and is additionally confirmed by X-ray diffraction data for **13b** (see below), since the absolute configuration of the stereo center at C-2 in **6e** does not change during the transformation of **6e** to **13b**. The relative configurations of **6b–f** were also confirmed by comparing the NMR spectra of these compounds and **6a**. For (2*R*,3*S*)-isomers the values of $^3J_{HH}$ for proton at 2-C were 6.6–6.9 Hz, while for (2*S*,3*S*)-isomers these values were 11 Hz. A characteristic difference in the values of the spin – spin coupling constants was previously observed for structurally similar phosphonates [5] and sulfones [6].

Dimethyl [(2*R*,3*S*)-4-nitro-1-oxo-1,3-diphenylbutan-2-yl]phosphonate (6a) [5]

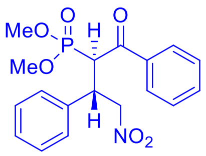

Yield: 1.13 g (45%), white crystals, m.p. 160–162 °C (methanol).

$[\alpha]_D^{20}$ -40.0 (*c* 2.5, $CHCl_3$), dr 1:-, > 99% ee.

^{1}H NMR (400 MHz, $CDCl_3$, 298 K) δ [ppm] 7.79-7.77 (m, 2H, aromatic), 7.56-7.52 (m, 1H, aromatic), 7.42-7.38 (m, 2H, aromatic), 7.27-7.18 (m, 5H, aromatic), 5.14-5.01 (m, 2H, H-4), 4.63 (dd, $^2J_{HP}$ = 23.6 Hz, $^3J_{HH}$ = 7.2 Hz, 1H, H-2), 4.42-4.30 (m, 1H, H-3), 3.55 (d, $^3J_{HP}$ = 10.4 Hz, 3H, MeO), 3.52 (d, $^3J_{HP}$ = 10.0 Hz, 3H, MeO).

^{13}C NMR (101 MHz, $CDCl_3$, 298 K) δ [ppm] 195.5 (d, $^2J_{CP}$ = 4.8 Hz, C-1), 137.43 (d, $^3J_{CP}$ = 9.5 Hz, aromatic, C), 137.27 (s, aromatic, C), 134.1 (s, aromatic, CH), 129.1 (s,

aromatic, 2CH), 128.9 (s, aromatic, 2CH), 128.7 (s, aromatic, 2CH), 128.4 (s, aromatic, CH), 128.0 (s, aromatic, 2CH), 77.4 (s, C-4), 53.7 (d, $^{2}J_{CP}$ = 6.6 Hz, MeO), 53.5 (d, $^{2}J_{CP}$ = 7.6 Hz, MeO), 50.3 (d, $^{1}J_{CP}$ = 128.5 Hz, C-2), 43.0 (d, $^{2}J_{CP}$ = 2.9 Hz, C-3).

^{31}P NMR (162 MHz, $CDCl_3$, 298 K) δ [ppm] 22.48.

IR (ATR) ν [cm^{-1}] 3063 (w), 2949 (w), 2853 (w), 1674 (s), 1597 (w), 1545 (s), 1449 (w), 1383 (w), 1250 (s), 1045 (s), 968 (m), 858 (w), 849 (w), 783 (m), 706 (m), 519 (m).

HRMS (APPI): *m/z* [M+H]$^{+}$ calcd for $C_{18}H_{20}NO_6P{\cdot}H^{+}$: 378.1101; found: 378.1101.

HPLC analysis (CHIRALPAK AD-3 column; hexane/2-propanol, 80:20; flow rate 1.0 mL/min; wavelength 230 nm): t_R = 9.7 (2*R*,3*S*), 22.3 (2*S*,3*R*) min.

Dimethyl [(2*R*,3*S*)-1-(4-methoxyphenyl)-4-nitro-1-oxo-3-phenylbutan-2-yl]-phosphonate (6b).

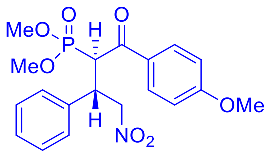

Yield: 1.51 g (46%), white crystals, m.p. 136–138 °C (toluene).

$[\alpha]_D^{20}$ -75.8 (*c* 1.0, $CHCl_3$), 98% ee.

^{1}H NMR (400 MHz, $CDCl_3$, 298 K) δ [ppm] 7.79 (d, $^{3}J_{HH}$ = 9.0 Hz, 2H, aromatic), 7.29-7.19 (m, 5H, aromatic), 6.87 (d, $^{3}J_{HH}$ = 9.0 Hz, 2H, aromatic), 5.16-5.01 (m, 2H, H-4), 4.56 (dd, $^{2}J_{HP}$ = 23.2 Hz, $^{3}J_{HH}$ = 6.4 Hz, 1H, H-2), 4.39-4.31 (m, 1H, H-3), 3.84 (s, 3H, MeO), 3.58 (d, $^{3}J_{HP}$ = 11.2 Hz, 3H, MeO), 3.55 (d, $^{3}J_{HP}$ = 11.2 Hz, 3H, MeO).

^{13}C NMR (101 MHz, $CDCl_3$, 298 K) δ [ppm] 193.5 (d, $^{2}J_{CP}$ = 4.8 Hz, C-1), 164.4 (s, aromatic, C), 137.6 (d, $^{3}J_{CP}$ = 10.5 Hz, aromatic, C), 131.3 (s, aromatic, 2CH), 130.3 (d, $^{3}J_{CP}$ = 1.9 Hz, aromatic, C), 129.1 (s, aromatic, 2CH), 128.3 (s, aromatic, CH), 128.0 (s, aromatic, 2CH), 114.1 (s, aromatic, 2CH), 77.3 (s, C-4), 55.7 (s, MeO), 53.7 (d, $^{2}J_{CP}$ = 6.7 Hz, MeO), 53.5 (d, $^{2}J_{CP}$ = 5.7 Hz, MeO), 49.9 (d, $^{1}J_{CP}$ = 129.4 Hz, C-2); 43.0 (d, $^{2}J_{CP}$ = 3.8 Hz, C-3).

^{31}P NMR (162 MHz, $CDCl_3$, 298 K) δ [ppm] 23.87.

IR (ATR) ν [cm^{-1}] 3063 (w), 3008 (w), 2955 (w), 2843 (w), 1667 (vs), 1597 (vs), 1551 (vs), 1516 (m), 1458 (m), 1435 (m), 1381 (m), 1334 (m), 1311 (m), 1269 (vs), 1242 (vs), 1172 (vs), 1061 (vs), 1018 (vs), 972 (m), 837 (m), 818 (m), 702 (m).

HRMS (APPI): *m*/*z* [M+H]$^+$ calcd for $C_{19}H_{22}NO_7P\cdot H^+$: 408.1207; found: 408.1212.

HPLC analysis (CHIRALPAK AD-3R column; water/acetonitrile, gradient: 0.1 min 30% acetonitrile to 50 min 100% acetonitrile; flow rate 0.2 mL/min; wavelength 230 nm): t_R = 13.3 (2*R*,3*S*), 16.3 (2*S*,3*R*) min.

Dimethyl [(2*R*,3*S*)-1-(3-methoxyphenyl)-4-nitro-1-oxo-3-phenylbutan-2-yl]phosphonate (6c).

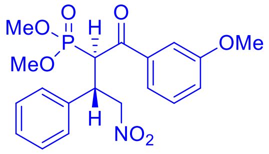

Yield: 1.49 g (45%), white crystals, m.p. 120–121 °C (EtOH).

$[\alpha]_D^{20}$ -54.6 (*c* 1.0, $CHCl_3$), dr 14:1, >99% ee.

^{1}H NMR (400 MHz, $CDCl_3$, 298 K) δ [ppm] 7.32-7.20 (m, 8H, aromatic), 7.09-7.05 (m, 1H, aromatic), 5.14-5.00 (m, 2H, H-4), 4.58 (dd, $^2J_{HP}$ = 23.2 Hz, $^3J_{HH}$ = 6.8 Hz, 1H, H-2), 4.44-4.32 (m, 1H, H-3), 3.79 (s, 3H, MeO), 3.57 (d, $^3J_{HP}$ = 9.0 Hz, 3H, MeO), 3.54 (d, $^3J_{HP}$ = 9.0 Hz, 3H, MeO).

^{13}C NMR (101 MHz, $CDCl_3$, 298 K) δ [ppm] 195.3 (d, $^2J_{CP}$ = 4.7 Hz, C-1), 159.9 (s, aromatic), 138.7 (s, aromatic), 137.5 (d, $^3J_{CP}$ = 10.6 Hz, aromatic), 129.8 (s, aromatic), 129.1 (s, aromatic), 128.4 (s, aromatic), 128.0 (s, aromatic), 121.4 (s, aromatic), 120.6 (s, aromatic), 112.8 (s, aromatic), 77.3 (s, C-4), 55.5 (s, MeO), 53.7 (d, $^2J_{CP}$ = 7.2 Hz, MeO), 53.5 (d, $^2J_{CP}$ = 7.2 Hz, MeO), 50.6 (d, $^1J_{CP}$ = 128.5 Hz, C-2), 43.0 (d, $^2J_{CP}$ = 2.8 Hz, C-3).

^{31}P NMR (162 MHz, $CDCl_3$, 298 K) δ [ppm] 22.47.

IR (ATR) ν [cm^{-1}] 3049 (w), 3022 (w), 2961 (w), 2839 (w), 1710 (m), 1670 (s), 1597 (m), 1549 (vs), 1454 (m), 1429 (m), 1381 (m), 1269 (s), 1250 (s), 1234 (s), 1217 (s), 1167 (s), 1057 (s), 1043 (vs), 1030 (vs), 1009 (vs), 878 (m), 851 (m), 826 (m), 795 (vs), 758 (s), 698 (vs), 679 (s), 638 (m), 553 (m), 513 (vs).

HRMS (APPI): *m/z* $[M+H]^+$ calcd for $C_{19}H_{22}NO_7P{\cdot}H^+$: 408.1207; found: 408.1213.

HPLC analysis (CHIRALPAK AD-3 column; hexane/2-propanol, 80:20; flow rate 1.2 mL/min; wavelength 210 nm): t_R = 11.7 (2*R*,3*S*), 22.3 (2*S*,3*R*) min.

For (2*R*,3*R*)-isomer:

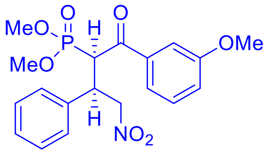

Yield: 1.19 g (36%), white crystals, m.p. 124–125 °C (MeOH).

$[\alpha]_D^{20}$ +51.3 (*c* 1.0, $CHCl_3$), dr 8:1, >99% ee.

^{1}H NMR (400 MHz, $CDCl_3$, 298 K) δ [ppm] 7.31-6.99 (m, 9H, aromatic), 5.24 (dd, $^2J_{HH}$ = 13.2 Hz, $^3J_{HH}$ = 4.0 Hz, 1H, H-4), 4.92 (dd, $^2J_{HH}$ = 12.8 Hz, $^3J_{HH}$ = 10.4 Hz, 1H, H-4), 4.64 (dd, $^2J_{HP}$ = 20.4 Hz, $^3J_{HH}$ = 11.6 Hz, 1H, H-2), 4.44-4.36 (m, 1H, H-3), 3.82 (d, $^3J_{HP}$ = 10.8 Hz, 3H, MeO), 3.76 (s, 3H, MeO), 3.72 (d, $^3J_{HP}$ = 11.2 Hz, 3H, MeO).

^{13}C NMR (101 MHz, $CDCl_3$, 298 K) δ [ppm] 194.0 (d, $^2J_{CP}$ = 5.8 Hz, C-1), 159.8 (s, aromatic, C(OMe)), 138.6 (s, aromatic, C), 136.9 (d, $^3J_{CP}$ = 15.3 Hz, aromatic, C), 129.7 (s, aromatic, CH), 129.0 (s, aromatic, 2CH), 128.2 (s, aromatic, CH), 128.0 (s, aromatic, 2CH), 120.9 (s, aromatic, CH), 120.2 (s, aromatic, CH), 112.6 (s, aromatic, CH), 78.8 (s, C-4), 55.5 (s, MeO), 54.0 (d, $^2J_{CP}$ = 6.7 Hz, MeO), 53.8 (d, $^2J_{CP}$ = 7.6 Hz, MeO), 49.6 (d, $^1J_{CP}$ = 125.5 Hz, C-2), 43.3 (d, $^2J_{CP}$ = 3.9 Hz, C-3).

^{31}P NMR (162 MHz, $CDCl_3$, 298 K) δ [ppm] 22.62.

IR (ATR) ν [cm^{-1}] 3047 (w), 2960 (w), 1670 (vs), 1597 (vs), 1548 (vs), 1489 (s), 1454 (s), 1429 (s), 1381 (s), 1269 (s), 1234 (vs), 1217 (vs), 1029 (vs), 1008 (vs), 875 (s), 850 (s), 825 (s), 794 (vs), 758 (vs), 698 (vs), 677 (s).

HPLC analysis (CHIRALPAK AD-3 column; hexane/2-propanol, 80:20; flow rate 1.2 mL/min; wavelength 210 nm): t_R = 9.3 (2*S*,3*S*), 12.8 (2*R*,3*R*) min.

Dimethyl [(2*S*,3*S*)-1-(2-methoxyphenyl)-4-nitro-1-oxo-3-phenylbutan-2-yl]phosphonate (6d).

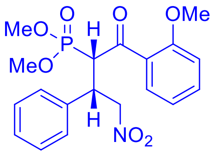

Yield: 1.65 g (50%), white crystals, m.p. 138–139 °C (MeOH).

$[\alpha]_D^{20}$ -88.7 (*c* 1.0, $CHCl_3$), dr 1:-, 98% ee.

^{1}H NMR (400 MHz, $CDCl_3$, 298 K) δ [ppm] 7.40-7.33 (m, 2H, aromatic), 7.20-7.05 (m, 5H, aromatic), 6.92-6.80 (m, 2H, aromatic), 5.22 (dd, $^2J_{HH}$ = 13.6 Hz, $^3J_{HH}$ = 4.4 Hz, 1H, H-4), 5.18 (dd, $^2J_{HH}$ = 21.6 Hz, $^3J_{HH}$ = 10.8 Hz, 1H, H-4), 4.93 (dd, $^2J_{HP}$ = 13.6 Hz, $^3J_{HH}$ = 10.8 Hz, 1H, H-2), 4.41-4.32 (m, 1H, H-3), 3.92 (s, 3H, MeO), 3.79 (d, $^3J_{HP}$ = 11.0 Hz, 3H, MeO), 3.65 (d, $^3J_{HP}$ = 11.0 Hz, 3H, MeO).

^{13}C NMR (101 MHz, $CDCl_3$, 298 K) δ [ppm] 195.0 (d, $^2J_{CP}$ = 5.7 Hz, C-1), 158.1 (s, aromatic), 137.4 (d, $^3J_{CP}$ = 14.3 Hz, aromatic), 134.5 (s, aromatic), 131.3 (s, aromatic), 128.6 (s, aromatic), 128.3 (s, aromatic), 127.9 (s, aromatic), 127.6 (s, aromatic), 121.0 (s, aromatic), 111.7 (s, aromatic), 78.9 (s, C-4), 55.8 (d, $^2J_{CP}$ = 2.9 Hz, MeO), 53.6 (s), 53.4 (s) 53.3 (d, $^1J_{CP}$ = 125.6 Hz, C-2), 43.4 (d, C-3, $^2J_{CP}$ = 3.8 Hz, C-3).

^{31}P NMR (162 MHz, $CDCl_3$, 298 K) δ [ppm] 23.82.

IR (ATR) ν [cm^{-1}] 3050 (w), 3021 (w), 2959 (w), 2851 (w), 1674 (vs), 1597 (m), 1543 (vs), 1485 (m), 1443 (m), 1385 (m), 1323 (m), 1300 (m), 1242 (vs), 1196 (m), 1061 (s), 1026 (vs), 841 (w), 814 (m), 764 (s), 702 (m).

HRMS (APPI): *m*/*z* [M+H]$^+$ calcd for $C_{19}H_{22}NO_7P{\cdot}H^+$: 408.1207; found: 408.1214.

HPLC analysis (CHIRALPAK AD-3 column; hexane/2-propanol, 85:15; flow rate 1.2 mL/min; wavelength 210 nm): t_R = 15.1 (2*S*,3*S*), 17.5 (2*R*,3*R*) min.

Dimethyl [(2*S*,3*R*)-1-nitro-4-oxo-2-phenylpentan-3-yl]phosphonate (6e).

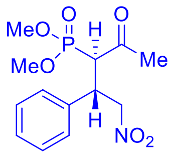

Yield: 1.17 g (46%), white crystals, m.p. 130–132 °C (MeOH).

$[\alpha]_D^{20}$ +70.7 (*c* 1.0, $CHCl_3$), dr 11:1.

^{1}H NMR (400 MHz, $CDCl_3$, 298 K) δ [ppm] 7.32-7.19 (m, 5H, Ph) 5.02 (dd, $^2J_{HH}$ = 13.4 Hz, $^3J_{HH}$ = 9.5 Hz, 1H, H-1), 4.88 (dd, $^2J_{HH}$ = 13.4 Hz, $^3J_{HH}$ = 3.4 Hz, 1H, H-1), 4.12-4.05 (m, 1H, H-2), 3.85-3.75 (m, 1H, H-3), 3.59 (d, $^3J_{HP}$ = 4.0 Hz, 3H, MeO), 3.56 (d, $^3J_{HP}$ = 4.0 Hz, 3H, MeO), 2.23 (s, 3H, H-5).

^{13}C NMR (101 MHz, $CDCl_3$, 298 K) δ [ppm] 203.4 (d, $^2J_{CP}$ = 3.8 Hz, C-4), 137.4 (d, $^3J_{CP}$ = 9.5 Hz, Ph), 129.2 (s, Ph), 128.4 (s, Ph), 127.9 (s, Ph), 127.8 (s, Ph), 77.1 (d, $^3J_{CP}$ =

7.6 Hz, C-1), 55.7 (d, $^{1}J_{CP}$ = 125.6 Hz, C-3), 53.9 (d, $^{2}J_{CP}$ = 6.6 Hz, MeO), 53.1 (d, $^{2}J_{CP}$ = 6.6 Hz, MeO), 42.2 (d, $^{2}J_{CP}$ = 2.8 Hz, C-2), 33.1 (s, C-5).

^{31}P NMR (162 MHz, $CDCl_3$, 298 K) δ [ppm] 21.95.

IR (ATR) ν [cm^{-1}] 3050 (w), 3025 (w), 2959 (m), 2840 (w), 1713 (vs), 1547 (vs), 1458 (m), 1439 (m), 1381 (m), 1362 (m), 1250 (vs), 1181 (m), 1157 (m), 1038 (vs), 880 (m), 826 (s), 764 (m), 706 (s), 606 (s).

HRMS (APPI): *m/z* $[M+H]^+$ calcd for $C_{13}H_{19}NO_6P{\cdot}H^+$ 316.0945; found 316.0944.

Dimethyl [(2*S*,3*S*)-1-(adamantan-1-yl)-4-nitro-1-oxo-3-phenylbutan-2-yl]phosphonate (6f) [2]

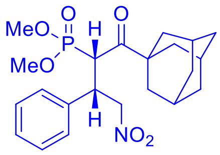

Yield: 0.74 g (21%), white crystals, m.p. 179–180 °C (MeOH).

$[\alpha]_D^{20}$ -18.6 (*c* 1.0, $CHCl_3$), dr 25:1, >99% ee.

^{1}H NMR (400 MHz, $CDCl_3$, 298 K) δ [ppm] 7.32-7.16 (m, 5H, aromatic), 5.16 (dd, $^{2}J_{HH}$ = 13.2 Hz, $^{3}J_{HH}$ = 4.4 Hz, 1H, H-4), 4.85 (dd, $^{2}J_{HH}$ = 13.2 Hz, $^{3}J_{HH}$ = 11.6 Hz, 1H, H-4), 4.19-4.13 (m, 1H, H-3), 4.02 (dd, $^{2}J_{HP}$ = 18.4 Hz, $^{3}J_{HH}$ = 10.8 Hz, 1H, H-2), 3.82 (d, $^{3}J_{HP}$ = 10.8 Hz, 3H, MeO), 3.79 (d, $^{3}J_{HP}$ = 11.9 Hz, 3H, MeO), 1.84 (br s, 3H, Ad), 1.66-1.37 (m, 12H, Ad).

^{13}C NMR (101 MHz, $CDCl_3$, 298 K) δ [ppm] 209.7 (d, $^{2}J_{CP}$ = 5.7 Hz, C-1), 137.3 (d, $^{3}J_{CP}$ = 15.3 Hz, aromatic, C), 129.0 (s, aromatic, 2CH), 128.7 (s, aromatic, 2CH), 128.4 (s, aromatic, CH), 77.0 (s, C-4), 54.0 (d, $^{2}J_{CP}$ = 6.7 Hz, MeO), 53.4 (d, $^{2}J_{CP}$ = 6.7 Hz, MeO), 49.5 (d, $^{1}J_{CP}$ = 124.5 Hz, C-2), 47.4 (s, C-3), 43.7 (d, $^{3}J_{CP}$ = 4.7 Hz, C, Ad), 37.8 (s, CH_2, Ad), 36.2 (s, CH_2, Ad), 27.8 (s, CH, Ad).

^{31}P NMR (162 MHz, $CDCl_3$, 298 K) δ [ppm] 23.39.

IR (ATR) ν [cm^{-1}] 3032 (w), 2916 (vs), 2854 (s), 1689 (vs), 1546 (vs), 1496 (m), 1452 (s), 1381 (s), 1344 (m), 1307 (m), 1232 (vs), 1176 (s), 1058 (vs), 1016 (vs), 999 (vs), 867 (s), 821 (s), 812 (s), 775 (s), 759 (s), 744 (s), 702 (s), 555 (s), 528 (s).

HRMS (APPI): *m/z* $[M+H]^+$ calcd for $C_{22}H_{30}NO_6P{\cdot}H^+$: 436.1883; found: 436.1892.

HPLC analysis (CHIRALPAK AD-3 column; hexane/2-propanol, 90:10; flow rate 1.2 mL/min; wavelength 210 nm): t_R = 7.8 (2*S*,3*S*), 8.7 (2*R*,3*R*) min.

For (2*R*,3*S*)-isomer:

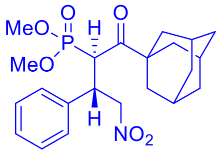

Yield: 176 mg (5%), white crystals, m.p. 91–93 °C (benzene).

$[\alpha]_D^{20}$ +12.2 (*c* 1.0, $CHCl_3$), dr 30:1.

^{1}H NMR (400 MHz, $CDCl_3$, 298 K) δ [ppm] 7.31-7.18 (m, 5H, aromatic), 5.05 (d, $^2J_{HH}$ = 7.2 Hz, 2H, H-4), 4.23-4.17 (m, 1H, H-3), 4.15 (dd, $^2J_{HP}$ = 28.4 Hz, $^3J_{HH}$ = 6.4 Hz, 1H, H-2), 3.66 (d, $^3J_{HP}$ = 11.2 Hz, 3H, MeO), 3.62 (d, $^3J_{HP}$ = 11.2 Hz, 3H, MeO), 1.92 (br s, 3H, Ad), 1.65-1.52 (m, 12H, Ad).

^{13}C NMR (101 MHz, $CDCl_3$, 298 K) δ [ppm] 211.2 (d, $^2J_{CP}$ = 4.8 Hz, C-1), 137.5 (d, $^3J_{CP}$ = 10.5 Hz, aromatic, C), 129.0 (s, aromatic, 2CH), 128.4 (s, aromatic, CH), 128.0 (s, aromatic, 2CH), 77.0 (s, C-4), 54.0 (d, $^2J_{CP}$ = 6.7 Hz, MeO), 53.1 (d, $^2J_{CP}$ = 7.6 Hz, MeO), 49.6 (d, $^1J_{CP}$ = 126.5 Hz, C-2), 47.9 (s, C-3), 43.0 (d, $^3J_{CP}$ = 3.8 Hz, C, Ad), 37.9 (s, CH_2, Ad), 36.2 (s, CH_2, Ad), 27.8 (s, CH, Ad).

^{31}P NMR (162 MHz, $CDCl_3$, 298 K) δ [ppm] 22.78.

IR (ATR) ν [cm^{-1}] 3032 (w), 2904 (vs), 2852 (s), 1687 (vs), 1544 (vs), 1496 (m), 1452 (s), 1381 (s), 1342 (m), 1325 (m), 1249 (vs), 1211 (s), 1145 (m), 1056 (vs), 1016 (vs), 935 (m), 916 (m), 871 (s), 844 (s), 829 (s), 812 (s), 775 (s), 698 (s), 559 (s), 532 (s).

4. Synthesis of pyrrolidin-3-ylphosphonic acids

The study of the hydrogenation of phosphonate 6a under various conditions

A solution of phosphonate **6a** (2.65 mmol) in various solvents (MeOH, EtOH, iPrOH, THF, EtOAc, 9 mL) was transferred in a 100 mL steel autoclave under argon and 10% Pd/C (0.3 g) was added. Argon was replaced with hydrogen with 3 cycles of pressurization (10 bar)/depressurization. The reactor was finally charged with a

pressure of H_2 (30 bar), then left stirring with magnetic stirrer for 36 h at 25 °C. Then the reactor was depressurized. The reaction mixture was diluted by chloroform (30 mL), filtered and evaporated in vacuo. The components of the reaction mixture were separated by column liquid chromatography (eluent – chloroform/ethanol 0–5%) and analyzed by NMR ^1H, ^{13}C and ^{31}P spectroscopy.

Dimethyl [(2*R*,3*R*,4*S*)-2,4-diphenylpyrrolidin-3-yl]phosphonate (7).

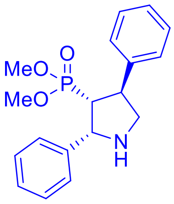

^1H NMR (400 MHz, $CDCl_3$, 298 K) δ [ppm] 7.52-7.22 (m, 10H, Ph), 4.70 (dd, $^3J_{HH}$ = 8.0 Hz, $^3J_{HP}$ = 25.2 Hz, 1H, H-2), 3.85-3.72 (m, 1H, H-4, 1H, H-5), 3.27 (d, $^3J_{HP}$ = 10.8 Hz, 3H, CH_3O), 3.11-3.03 (m, 1H, H-5), 2.96 (d, $^3J_{HP}$ =10.4 Hz, 3H, CH_3O), 2.88-2.80 (m, 1H, H-3).

^{13}C NMR (101 MHz, $CDCl_3$, 298 K) δ [ppm] 142.6 (d, $^3J_{CP}$ = 5.8 Hz, C, aromatic), 139.4 (d, $^3J_{CP}$ = 5.7 Hz, C, aromatic), 128.8 (s, 2CH, aromatic), 128.1 (s, 2CH, aromatic), 128.0 (s, 2CH, aromatic), 127.6 (s, 2CH, aromatic), 127.5 (s, CH, aromatic), 127.0 (s, CH, aromatic), 65.4 (s, C-2), 56.2 (d, $^3J_{CP}$ = 8.6 Hz, C-5), 51.88 (d, $^2J_{CP}$ = 6.7 Hz, CH_3O), 51.86 (d, $^2J_{CP}$ = 7.6 Hz, CH_3O), 50.1 (d, $^1J_{CP}$ = 144.7 Hz, C-3), 47.8 (s, C-4).

^{31}P NMR (162 MHz, $CDCl_3$, 298 K) δ [ppm] 31.49.

Dimethyl [(2*R*,3*R*,4*S*)-1-methyl-2,4-diphenylpyrrolidin-3-yl]phosphonate (8).

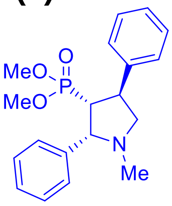

^1H NMR (400 MHz, DMSO-d^6, 298 K) δ [ppm] 7.44-7.38 (m, 4H, aromatic), 7.31-7.27 (m, 4H, aromatic), 7.22-7.20 (m, 2H, aromatic), 3.77 (dd, $^3J_{HH}$ = 10.0 Hz, $^3J_{HP}$ = 24.0 Hz, 1H, H-2), 3.65-3.53 (m, 1H, H-4), 3.43-3.38 (m, 1H, H-5), 3.03 (d, $^3J_{HP}$ = 10.8 Hz, 3H, MeO), 2.76 (d, $^3J_{HP}$ = 10.8 Hz, 3H, MeO), 2.47-2.46 (m, 1H, H-5, 1H, H-3), 2.04 (s, 3H, Me).

^{13}C NMR (101 MHz, DMSO-d^6, 298 K) δ [ppm] 142.6 (d, $^3J_{HP}$ = 2.8 Hz, aromatic, C), 139.5 (d, $^3J_{HP}$ = 6.6 Hz, aromatic, C), 130.0 (s, aromatic, 2CH), 128.8 (s, aromatic, 2CH), 128.5 (s, aromatic, 2CH), 127.9 (s, aromatic, 2CH), 127.7 (s, aromatic, CH), 127.2 (s, aromatic, CH), 72.2 (s, C-2), 65.7 (d, $^3J_{CP}$ = 9.6 Hz, C-5), 51.8 (d, $^2J_{CP}$ = 7.6

Hz, MeO), 51.7 (d, $^{2}J_{CP}$ = 6.7 Hz, MeO), 48.4 (d, $^{1}J_{CP}$ = 146.6 Hz, C-3), 45.0 (d, $^{2}J_{CP}$ = 1.9 Hz, C-4), 40.3 (s, Me-N).

^{31}P NMR (162 MHz, DMSO-d^{6}, 298 K) δ [ppm] 31.52.

IR (ATR) ν [cm^{-1}] 3062 (w), 3028 (w), 2947 (w), 2914 (w), 2850 (w), 2789 (w), 1494 (m), 1452 (m), 1253 (m), 1234 (s), 1215 (s), 1182 (m), 1147 (m), 1066 (vs), 1051 (vs), 1020 (vs), 997 (s), 914 (m), 842 (s), 817 (s), 777 (vs), 761 (s), 754 (s), 698 (vs), 690 (vs).

Methyl hydrogen [(2*R*,3*R*,4*S*)-2,4-diphenylpyrrolidin-3-yl]phosphonate (9).

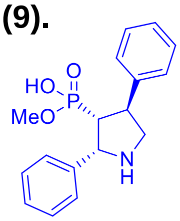

^{1}H NMR (400 MHz, D_2O, 298 K) δ [ppm] 7.16-6.96 (m, 10H, aromatic), 4.98 (t, $^{3}J_{HH}$ = 8.4 Hz, $^{3}J_{HP}$ = 8.4 Hz, 1H, H-2), 3.80-3.71 (m, 2H, H-5), 3.23-3.16 (m, 1H, H-4), 3.03-2.93 (m, 1H, H-3), 2.74 (d, $^{3}J_{HP}$ = 11.2 Hz, 3H, MeO).

^{13}C NMR (101 MHz, D_2O, 298 K) δ [ppm] 136.8 (s, aromatic, C), 132.5 (d, $^{3}J_{CP}$ = 3.8 Hz, aromatic, C), 129.6 (s, aromatic, CH), 129.0 (s, aromatic, CH), 129.0 (s, aromatic, 2CH), 128.7 (s, aromatic, 2CH), 128.1 (s, aromatic, 2CH), 127.7 (s, aromatic, 2CH), 64.2 (d, $^{2}J_{CP}$ = 13.5 Hz, C-2), 51.8 (s, C-5), 51.8 (d, $^{2}J_{CP}$ = 14.4 Hz, MeO), 47.0 (d, $^{1}J_{CP}$ = 150.5 Hz, C-3), 44.4 (s, C-4).

^{31}P NMR (162 MHz, D_2O, 298 K) δ [ppm] 22.67.

IR (ATR) ν [cm^{-1}] 3412 (w), 3062 (w), 2968 (w), 1602 (m), 1587 (m), 1496 (m), 1456 (s), 1203 (vs), 1172 (vs), 1051 (vs), 756 (vs), 696 (vs).

General procedure for the synthesis of 1-formylpyrrolidin-3-ylphosphonates (10a–d).

A solution of phosphonate **6a–c, e** (2.65 mmol) in AcOH (9 mL) was transferred in a 100 mL steel autoclave under argon and 10% Pd/C (0.3 g) was added. Argon was replaced with hydrogen with 3 cycles of pressurization (10 bar)/depressurization. The reactor was finally charged with a pressure of H_2 (30 bar), then left stirring with magnetic stirrer for 36 h at 25 °C. Then the reactor was depressurized. The reaction mixture was diluted by chloroform (30 mL), filtered and evaporated in vacuo. The residue was treated by

$NaHCO_3$ and water. The mixture was extracted with CH_2Cl_2 (3 × 10 mL). The combined organic extracts were dried over Na_2SO_4, filtered, and evaporated in vacuo. Ethyl formate (16 mL) was added to a residue and the reaction mixture was refluxed for 3 h. The excess of ethyl formate was evaporated in vacuo. The crude product was purified by flash chromatography on silica gel and eluted with a mixture of chloroform/ethanol (0–5%) to give a mixture – a yellow oil. From this mixture, a product is precipitated in cyclohexane and recrystallized from CCl_4 to give a white solid.

Dimethyl [(2*R*,3*R*,4*S*)-1-formyl-2,4-diphenylpyrrolidin-3-yl]phosphonate (10a).

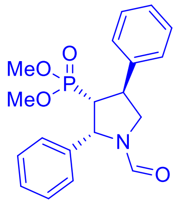

Mixture of rotamers 4.8:1. Yield: 0.50 g (52%), white crystals, m.p. 126–129 °C (cyclohexane); $[\alpha]_D^{20}$ +45.4 (*c* 1.0, $CHCl_3$).

^1H NMR (400 MHz, $CDCl_3$, 298 K) δ [ppm] 8.30 (s, 1H, CHO, minor rotamer), 8.15 (s, 1H, CHO, major rotamer), 7.40-7.24 (m, 10H, aromatic, major rotamer, 10H, aromatic, minor rotamer), 5.56 (dd, $^3J_{HH}$ = 2.8 Hz, $^3J_{PH}$ = 8.0 Hz, 1H, H-2, minor rotamer), 5.40 (dd, $^3J_{HH}$ = 2.0 Hz, $^3J_{PH}$ = 7.6 Hz, 1H, H-2, major rotamer), 4.37-4.32 (m, 1H, H-4, minor rotamer), 4.29-4.24 (m, 1H, H-4, major rotamer), 4.03-3.95 (m, 1H, H-5, minor rotamer), 3.90-3.80 (m, 1H, H-5, major rotamer), 3.79-3.67 (m, 1H, H-5, minor rotamer, 1H, H-5, major rotamer), 3.14 (d, $^3J_{HP}$ = 11.2 Hz, 3H, MeO, minor rotamer), 3.13 (d, $^3J_{HP}$ = 11.2 Hz, 3H, MeO, major rotamer), 3.10-3.02 (m, 1H, H-3, minor rotamer, 1H, H-3, major rotamer), 2.91 (d, $^3J_{HP}$ = 10.8 Hz, 3H, MeO, minor rotamer), 2.90 (d, $^3J_{HP}$ = 10.8 Hz, 3H, MeO, major rotamer).

^{13}C NMR (101 MHz, $CDCl_3$, 298 K) δ [ppm] 161.6 (s, CHO, major rotamer), 160.3 (s, CHO, minor rotamer), 139.7 (d, $^3J_{CP}$ = 2.8 Hz, aromatic, minor rotamer), 138.3 (s, aromatic, major rotamer), 128.9 (s, aromatic, minor rotamer), 128.8 (s, aromatic, major rotamer), 128.7 (s, aromatic, minor rotamer), 128.5 (s, aromatic, major rotamer), 128.4 (s, aromatic, major rotamer), 128.2 (s, aromatic, major rotamer), 128.1 (s, aromatic, minor rotamer), 128.0 (s, aromatic, minor rotamer), 127.9 (s, aromatic, minor rotamer),

127.9 (s, aromatic, major rotamer), 127.8 (s, aromatic, minor rotamer), 127.7 (s, aromatic, major rotamer), 62.8 (d, $^{2}J_{CP}$ = 5.7 Hz, C-2, major rotamer), 60.8 (d, $^{2}J_{CP}$ = 4.7 Hz, C-2, minor rotamer), 54.3 (d, $^{3}J_{CP}$ = 14.4 Hz, C-5, minor rotamer), 52.4 (d, $^{3}J_{CP}$ = 14.4 Hz, C-5, major rotamer), 52.2 (d, $^{2}J_{CP}$ = 6.7 Hz, MeO, minor rotamer, MeO, major rotamer), 51.6 (d, $^{2}J_{CP}$ = 6.7 Hz, MeO, minor rotamer, MeO, major rotamer), 49.4 (d, $^{1}J_{CP}$ = 154.3 Hz, C-3, major rotamer), 48.6 (d, $^{1}J_{CP}$ = 155.3 Hz, C-3, minor rotamer), 43.5 (s, C-4, minor rotamer), 42.8 (d, $^{2}J_{CP}$ = 1.9 Hz, C-4, major rotamer).

^{31}P NMR (162 MHz, $CDCl_3$, 298 K) δ [ppm] 26.02 (minor rotamer), 25.44 (major rotamer).

IR (ATR) ν [cm^{-1}] 3061 (w), 3032 (w), 2951 (w), 2920 (w), 2891 (w), 2852 (w), 1658 (vs), 1604 (m), 1494 (m), 1469 (m), 1454 (m), 1413 (m), 1388 (s), 1247 (vs), 1184 (s), 1041 (vs), 1020 (vs), 1006 (vs), 823 (vs), 781 (s), 734 (vs), 696 (vs), 671 (s), 569 (s), 524 (s), 426 (s).

HRMS (APPI): *m*/*z* $[M+H]^+$ calcd for $C_{19}H_{22}NO_4P{\cdot}H^+$, 360.1359, found 360.1361.

Dimethyl [(2*R*,3*R*,4*S*)-1-formyl-2-(4-methoxyphenyl)-4-phenylpyrrolidin-3-yl]-phosphonate (10b).

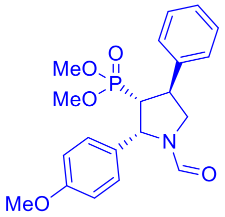

Mixture of rotamers 4.8:1. Yield: 0.52 g (50%), white crystals, m.p. 120–122 °C (CCl_4). $[\alpha]_D^{20}$ +47.5 (*c* 1.0, $CHCl_3$).

^{1}H NMR (400 MHz, $CDCl_3$, 298 K) δ [ppm] 8.28 (s, 1H, CHO, minor rotamer), 8.14 (s, 1H, CHO, major rotamer), 7.35-7.23 (m, 7H, aromatic, minor rotamer, 7H, aromatic, major rotamer), 6.91 (s, 1H, aromatic, major rotamer), 6.88 (s, 1H, aromatic, major rotamer, 1 H, aromatic, minor rotamer), 6.86 (s, 1H, aromatic, minor rotamer), 5.52 (dd, $^{3}J_{HP}$ = 8.0 Hz, $^{3}J_{HH}$ = 2.5 Hz, 1H, H-2, minor rotamer), 5.34 (dd, $^{3}J_{HP}$ = 7.8 Hz, $^{3}J_{HH}$ = 1.8 Hz, 1H, H-2 major rotamer), 4.34-4.29 (m, 1H, H-4, minor rotamer), 4.27-4.21 (m, 1H, H-4, major rotamer), 4.01-3.95 (m, 1H, H-5, minor rotamer), 3.86-3.81 (m, 1H, H-5, major rotamer, 1H, H-5, minor rotamer), 3.79 (s, 3H, MeO, major rotamer), 3.76 (s, 3H,

MeO, minor rotamer), 3.69-3.63 (m, 1H, H-5, major rotamer), 3.17 (d, $^3J_{HP}$ = 10.9 Hz, 3H, MeO, minor rotamer), 3.15 (d, $^3J_{HP}$ = 10.9 Hz, 3H, MeO, major rotamer), 3.08-2.98 (m, 1H, H-3, minor rotamer, 1H, H-3, major rotamer), 2.95 (d, $^3J_{HP}$ = 10.7 Hz, 3H, MeO, minor rotamer), 2.94 (d, $^3J_{HP}$ = 10.7 Hz, 3H, MeO, major rotamer).

^{13}C NMR (101 MHz, $CDCl_3$, 298 K) δ [ppm] 161.6 (s, CHO, major rotamer), 160.2 (s, CHO, minor rotamer), 159.7 (s, aromatic, major rotamer), 159.3 (s, aromatic, minor rotamer), 138.5 (s, aromatic, minor rotamer), 138.4 (s, aromatic, major rotamer), 131.8 (d, $^3J_{CP}$ = 2.8 Hz, aromatic, major rotamer), 130.7 (d, $^3J_{CP}$ = 2.8 Hz, aromatic, minor rotamer), 129.6 (s, aromatic, major rotamer), 129.4 (s, aromatic, minor rotamer), 128.9 (s, aromatic, minor rotamer), 128.8 (s, aromatic, major rotamer), 127.9 (s, aromatic, major rotamer), 127.78 (s, aromatic, minor rotamer), 127.75 (s, aromatic, minor rotamer, aromatic, major rotamer), 113.8 (s, aromatic, major rotamer), 113.6 (s, aromatic, minor rotamer), 62.3 (d, $^2J_{CP}$ = 4.8 Hz, C-2, major rotamer), 60.3 (d, $^2J_{CP}$ = 4.8 Hz, C-2, minor rotamer), 55.4 (s, MeO, major rotamer), 55.3 (s, MeO, minor rotamer), 54.2 (d, $^3J_{CP}$ = 13.4 Hz, C-5, minor rotamer), 52.3 (d, $^2J_{CP}$ = 6.7 Hz, MeO, minor rotamer, MeO, major rotamer), 52.2 (d, $^3J_{CP}$ = 14.3 Hz, C-5, major rotamer), 51.5 (d, $^2J_{CP}$ = 6.7 Hz, MeO, minor rotamer, MeO, major rotamer), 49.4 (d, $^1J_{CP}$ = 155.3 Hz, C-3, major rotamer), 48.7 (d, $^1J_{CP}$ = 155.3 Hz, C-3, minor rotamer), 43.6 (s, C-4, minor rotamer), 42.8 (s, C-4, major rotamer).

^{31}P NMR (162 MHz, $CDCl_3$, 298 K) δ [ppm] 25.76 (major rotamer), 26.30 (minor rotamer).

IR (ATR) ν [cm^{-1}] 3066 (w), 3001 (w), 2949 (w), 2843 (w), 1654 (vs), 1612 (m), 1512 (s), 1382 (s), 1244 (vs), 1176 (s), 1043 (vs), 1022 (vs), 821 (s), 781 (s), 756 (vs), 702 (s), 661 (m), 569 (s), 532 (s).

HRMS (APPI): *m/z* $[M+H]^+$ calcd for $C_{20}H_{24}NO_5P\cdot H^+$, 390.1464, found 390.1463.

Dimethyl [(2*R*,3*R*,4*S*)-1-formyl-2-(3-methoxyphenyl)-4-phenylpyrrolidin-3-yl]-phosphonate (10c).

Mixture of rotamers 4.1:1. Yield: 0.49 g (48%), white crystals, m.p. 158–159 °C (CCl_4). $[\alpha]_D^{20}$ +41.9 (*c* 1.0, $CHCl_3$).

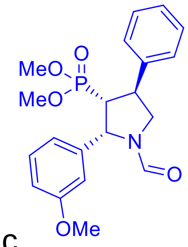

^{1}H NMR (400 MHz, $CDCl_3$, 298 K) δ [ppm] 8.29 (s, 1H, CHO, minor rotamer), 8.15 (s, 1H, CHO, major rotamer), 7.29-7.26 (m, 6H, aromatic, minor rotamer, 6H, aromatic, major rotamer), 6.96-6.92 (m, 2H, aromatic, minor rotamer, 2H, aromatic, major rotamer), 6.85-6.78 (m, 1H, aromatic, minor rotamer, 1H, aromatic, major rotamer), 5.54 (d, $^3J_{HP}$ = 8.0 Hz, 1H, H-2, minor rotamer), 5.36 (d, $^3J_{HP}$ = 7.5 Hz, 1H, H-2, major rotamer), 4.31 (t, $^3J_{HH}$ = 10.0 Hz, 1H, H-4, minor rotamer), 4.23 (t, $^3J_{HH}$ = 11.1 Hz, 1H, H-4, major rotamer), 3.98-3.91 (m, 1H, H-5, minor rotamer), 3.87-3.75 (m, 1H, H-5, minor rotamer, 1H, H-5, major rotamer), 3.71-3.65 (m, 1H, H-5, major rotamer), 3.81 (s, 3H, MeO, major rotamer), 3.79 (s, 3H, MeO, minor rotamer), 3.14 (d, $^3J_{HP}$ = 11.2 Hz, 3H, MeO, minor rotamer), 3.13 (d, $^3J_{HP}$ = 10.9 Hz, 3H, MeO, major rotamer), 3.09-2.98 (m, 1H, H-3, minor rotamer, 1H, H-3, major rotamer), 2.95 (d, $^3J_{HP}$ = 10.7 Hz, 3H, MeO, minor rotamer), 2.94 (d, $^3J_{HP}$ = 10.9 Hz, 3H, MeO, major rotamer).

^{13}C NMR (101 MHz, $CDCl_3$, 298 K) δ [ppm] 161.6 (s, CHO, major rotamer), 160.3 (s, CHO, minor rotamer), 159.6 (s, aromatic, major rotamer), 159.4 (s, aromatic, minor rotamer), 141.2 (d, $^3J_{CP}$ = 3.8 Hz, aromatic, major rotamer), 140.0 (d, $^3J_{CP}$ = 3.8 Hz, aromatic, minor rotamer), 138.4 (s, aromatic), 129.5 (s, aromatic, major rotamer), 129.2 (s, aromatic, minor rotamer), 128.9 (s, aromatic, minor rotamer), 128.8 (s, aromatic, major rotamer), 127.9 (s, aromatic, major rotamer), 127.79 (s, aromatic, minor rotamer), 127.76 (s, aromatic), 120.5 (s, aromatic, major rotamer), 120.3 (s, aromatic, minor rotamer), 114.5 (s, aromatic, major rotamer), 114.3 (s, aromatic, minor rotamer), 113.7 (s, aromatic, major rotamer), 113.3 (s, aromatic, minor rotamer), 62.7 (d, $^2J_{CP}$ = 5.7 Hz,

C-2, major rotamer), 60.6 (d, $^{2}J_{CP}$ = 4.8 Hz, C-2, minor rotamer), 55.4 (s, MeO, major rotamer), 55.3 (s, MeO, minor rotamer), 54.2 (d, $^{3}J_{CP}$ = 14.3 Hz, C-5, minor rotamer), 52.3 (d, $^{3}J_{CP}$ = 14.3 Hz, C-5, major rotamer), 52.2 (d, $^{2}J_{CP}$ = 6.7 Hz, MeO, minor rotamer, MeO, major rotamer), 51.6 (d, $^{2}J_{CP}$ = 6.7 Hz, MeO, minor and major rotamer), 49.4 (d, $^{1}J_{CP}$ = 154.3 Hz, C-3, major rotamer), 48.6 (d, $^{1}J_{CP}$ = 155.2 Hz, C-3, minor rotamer), 43.5 (s, C-4, minor rotamer), 42.9 (s, C-4, major rotamer).

^{31}P NMR (162 MHz, $CDCl_3$, 298 K) δ [ppm] 25.50 (major rotamer), 25.97 (minor rotamer).

IR (ATR) ν [cm^{-1}] 3055 (w), 3012 (w), 2953 (w), 2908 (w), 1654 (vs), 1600 (m), 1463 (m), 1386 (s), 1246 (vs), 1043 (vs), 1024 (vs), 817 (vs), 792 (vs), 754 (vs), 721 (vs), 700 (vs), 570 (s), 524 (vs).

HRMS (APPI): *m/z* [M+H]$^+$ calcd for $C_{20}H_{24}NO_5P{\cdot}H^+$, 390.1464, found 390.1464.

Dimethyl [(2*R*,3*R*,4*S*)-1-formyl-2-methyl-4-phenylpyrrolidin-3-yl]phosphonate (10d).

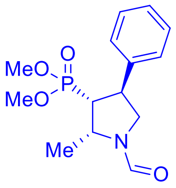

Mixture of rotamers 2.1:1. Yield: 0.39 g (49%), white crystals, m.p. 79–80 °C. $[\alpha]_D^{20}$ +5.8 (*c* 1.0, $CHCl_3$).

^{1}H NMR (400 MHz, $CDCl_3$, 298 K) δ [ppm] 8.26 (s, 1H, CHO, major rotamer), 8.16 (s, 1H, CHO, minor rotamer), 7.34-7.21 (m, 5H, aromatic, minor rotamer, 5H, aromatic, major rotamer), 4.67-4.60 (m, 1H, H-2, minor rotamer), 4.40-4.33 (m, 1H, H-2, major rotamer), 4.03-3.98 (m, 1H, H-4, minor rotamer), 3.92-3.86 (m, 1H, H-4, major rotamer), 3.85-3.69 (m, 1H, H-5, minor rotamer, 1H, H-5, major rotamer), 3.55-3.48 (m, 1H, H-5, minor rotamer, 1H, H-5, major rotamer), 3.54 (d, $^{3}J_{HP}$ = 10.9 Hz, 3H, MeO, minor rotamer), 3.49 (d, $^{3}J_{HP}$ = 10.9 Hz, 3H, MeO, major rotamer), 3.20 (d, $^{3}J_{HP}$ = 10.7 Hz, 3H, MeO, major rotamer), 3.15 (d, $^{3}J_{HP}$ = 10.9 Hz, 3H, MeO, minor rotamer), 2.76-2.65 (m, 1H, H-3, minor rotamer, 1H, H-3, major rotamer), 1.45 (d, $^{3}J_{HH}$ = 6.6 Hz, 3H, Me, major rotamer), 1.42 (d, $^{3}J_{HH}$ = 6.6 Hz, 3H, Me, minor rotamer).

^{13}C NMR (101 MHz, $CDCl_3$, 298 K) δ [ppm] 160.2 (s, CHO, minor rotamer), 160.1 (s, CHO, major rotamer), 138.8 (s, aromatic, major rotamer), 138.6 (s, aromatic, minor rotamer), 129.0 (s, aromatic, minor rotamer), 128.9 (s, aromatic, major rotamer), 127.9 (s, aromatic, minor rotamer, aromatic, major rotamer), 127.8 (s, aromatic, minor rotamer, aromatic, major rotamer), 55.3 (d, $^2J_{CP}$ = 4.7 Hz, C-2, major rotamer), 53.1 (d, $^2J_{CP}$ = 2.8 Hz, C-2, minor rotamer), 52.5 (d, $^2J_{CP}$ = 6.7 Hz, MeO, major rotamer), 52.4 (d, $^2J_{CP}$ = 6.7 Hz, MeO, minor rotamer), 51.79 (d, $^2J_{CP}$ = 6.7 Hz, MeO, major rotamer), 51.77 (d, $^2J_{CP}$ = 6.7 Hz, MeO, minor rotamer), 50.9 (d, $^3J_{CP}$ = 14.3 Hz, C-5, major rotamer, C-5, minor rotamer), 47.3 (d, $^1J_{CP}$ = 151.4 Hz, C-3, major rotamer), 46.6 (d, $^1J_{CP}$ = 151.4 Hz, C-3, minor rotamer), 43.1 (s, C-4, minor rotamer), 42.6 (s, C-4, major rotamer), 20.4 (d, $^3J_{CP}$ = 1.9 Hz, Me, major rotamer), 16.5 (d, $^3J_{CP}$ = 2.8 Hz, Me, minor rotamer).

^{31}P NMR (162 MHz, $CDCl_3$, 298 K) δ [ppm] 28.03 (major rotamer), 28.44 (minor rotamer).

IR (ATR) ν [cm^{-1}] 3061 (w), 2989 (w), 2953 (w), 2881 (w), 2852 (w), 1662 (vs), 1606 (m), 1498 (m), 1467 (m), 1456 (m), 1413 (m), 1381 (s), 1232 (vs), 1182 (s), 1022 (vs), 1002 (vs), 821 (vs), 781 (s), 748 (vs), 702 (vs), 650 (m), 596 (s), 565 (s), 526 (vs).

HRMS (APPI): *m*/*z* $[M+H]^+$ calcd for $C_{14}H_{20}NO_4P{\cdot}H^+$, 298.1202, found 298.1201.

General procedure for the synthesis of pyrrolidin-3-ylphosphonic acids (11a–d)

The mixture of 1-formylpyrrolidin-3-ylphosphonate **10a–d** (1.30 mmol) and 5% hydrochloric acid (5 mL) was refluxed for 33 h and resulting colorless solution was evaporated at reduced pressure. The residue was dried in vacuo to a constant weight.

(2*R*,3*R*,4*S*)-2,4-Diphenylpyrrolidin-3-ylphosphonic acid hydrochloride (11a).

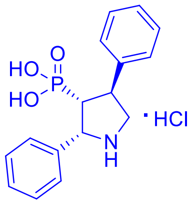

Yield: 398 mg (90%), white crystals, m.p. 179–180 °C (decomp.).

$[\alpha]_D^{20}$ +81.0 (*c* 1.0, H_2O).

^1H NMR (400 MHz, D_2O, 298 K) δ [ppm] 7.54-7.27 (m, 10H, aromatic),

5.22 (dd, $^3J_{HH}$ 8.4 Hz, $^3J_{HP}$ 16.8 Hz, 1H, H-2), 4.21-4.04 (m, 1H, H-5, 1H, H-4), 3.53-3.48 (m, 1H, H-5), 3.12-3.04 (m, 1H, H-3).

^{13}C NMR (101 MHz, D_2O, 298 K) δ [ppm] 138.6 (d, $^3J_{CP}$ 1.9 Hz, aromatic, C), 133.2 (d, $^3J_{CP}$ 3.8 Hz, aromatic, C), 129.4 (s, aromatic, CH), 129.1 (s, aromatic, 2CH), 128.8 (s, aromatic, 2CH), 128.2 (s, aromatic, 2CH), 128.0 (s, aromatic, CH), 127.8 (s, aromatic, 2CH), 64.9 (d, $^2J_{CP}$ 1.9 Hz, C-2), 51.8 (d, $^3J_{CP}$ 12.4 Hz, C-5), 48.9 (d, $^1J_{CP}$ 143.8 Hz, C-3), 44.7 (s, C-4).

^{31}P NMR (162 MHz, D_2O, 298 K) δ [ppm] 17.01.

IR (ATR) ν [cm^{-1}] 2891 (s), 2727 (s), 1602 (m), 1496 (m), 1456 (m), 1159 (s), 983 (s), 916 (s), 756 (s), 694 (s).

HRMS (APPI): *m*/*z* [M-Cl]$^+$ calcd for $C_{16}H_{19}ClNO_3P$-HCl·H$^+$, 304.1097, found 304.1099.

(2*R*,3*R*,4*S*)-2-(4-methoxyphenyl)-4-phenylpyrrolidin-3-ylphosphonic acid hydrochloride (11b).

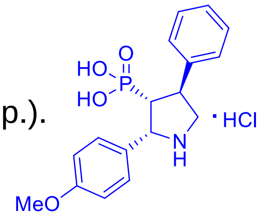

Yield: 356 mg (74%), white crystals, m.p. 205–208 °C (decomp.).

$[\alpha]_D^{20}$ +75.1 (*c* 1.0, H_2O).

^{1}H NMR (400 MHz, DMSO-d^6, 298 K) δ [ppm] 11.00 (br s, 1H, NH), 9.02 (br s., 1H, OH), 7.48-7.46 (m, 2H, aromatic), 7.43-7.41 (m, 2H, aromatic), 7.32-7.29 (m, 2H, aromatic), 7.24-7.20 (m, 1H, aromatic), 6.89-6.87 (m, 2H, aromatic), 5.07-4.97 (m, 1H, H-2), 4.03-3.90 (m, 1H, H-5, 1H, H-4), 3.73 (s, 3H, MeO), 3.36-3.21 (br s, 1H, H-5), 2.95-2.86 (m, 1H, H-3).

^{13}C NMR (101 MHz, DMSO-d^6, 298 K) δ [ppm] 159.7 (s, aromatic, **C**(OMe)), 141.0 (d, $^3J_{CP}$ = 2.8 Hz, aromatic, C), 130.8 (s, aromatic, 2CH), 129.0 (s, aromatic, 2CH), 128.6 (s, aromatic, 2CH), 127.6 (s, aromatic, CH), 126.4 (d, $^3J_{CP}$ = 2.8 Hz, aromatic, C), 113.8 (s, aromatic, 2CH), 64.0 (s, C-2), 55.7 (s, MeO), 51.4 (d, $^3J_{CP}$ = 10.5 Hz, C-5), 49.2 (d, $^1J_{CP}$ = 144.7 Hz, C-3), 44.5 (s, C-4).

^{31}P NMR (162 MHz, DMSO-d^6, 298 K) δ [ppm] 18.81.

IR (ATR) ν [cm^{-1}] 2899 (s), 2723 (s), 1612 (m), 1585 (m), 1517 (s), 1496 (m), 1456 (m), 1253 (s), 1182 (s), 989 (s), 923 (s), 827 (s), 700 (s), 520 (s).

HRMS (APPI): *m/z* [M-Cl]$^+$ calcd for $C_{17}H_{21}ClNO_4P$-HCl·H$^+$, 334.1202, found 334.1204.

(2*R*,3*R*,4*S*)-2-(3-methoxyphenyl)-4-phenylpyrrolidin-3-ylphosphonic acid hydrochloride (11c).

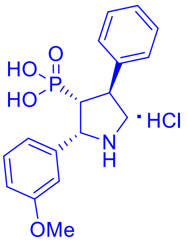

Yield: 481 mg (90%), white crystals, m.p. 172–173 °C (decomp.).

$[\alpha]_D^{20}$ +70.1 (*c* 1.0, H_2O).

^{1}H NMR (400 MHz, DMSO-*d*^{6}, 298 K) δ [ppm] 11.09 (br s, 1H, NH), 9.08 (br s., 1H, OH), 7.49-7.47 (m, 2H, aromatic), 7.33-7.21 (m, 4H, aromatic), 7.09-7.04 (m, 2H, aromatic), 6.89-6.86 (m, 1H, aromatic), 5.06-5.01 (m, 1H, H-2), 4.00-3.95 (m, 1H, H-5, 1H, H-4), 3.74 (s, 3H, MeO), 3.30 (br s, 1H, H-5), 2.98-2.90 (m, 1H, H-3).

^{13}C NMR (101 MHz, DMSO-*d*^{6}, 298 K) δ [ppm] 159.2 (s, aromatic, **C**(OMe)), 141.1 (d, $^3J_{CP}$ = 3.8 Hz, aromatic, C), 135.6 (d, $^3J_{CP}$ = 3.8 Hz, aromatic, C), 129.6 (s, aromatic, CH), 129.0 (s, aromatic, 2CH), 128.5 (s, aromatic, 2CH), 127.6 (s, aromatic, CH), 121.3 (s, aromatic, CH), 115.3 (s, aromatic, CH), 114.3 (s, aromatic, CH), 64.1 (d, $^2J_{CP}$ = 6.7 Hz, C-2), 55.6 (s, MeO), 51.4 (d, $^3J_{CP}$ = 9.6 Hz, C-5), 49.1 (d, $^1J_{CP}$ = 144.7 Hz, C-3), 44.5 (s, C-4).

^{31}P NMR (162 MHz, DMSO-*d*^{6}, 298 K) δ [ppm] 18.89.

IR (ATR) ν [cm^{-1}] 3045 (s), 2139 (s), 1602 (m), 1587 (m), 1496 (m), 1456 (m), 1261 (m), 1201 (m), 1159 (s), 987 (vs), 925 (s), 779 (m), 758 (m), 696 (vs), 530 (m).

HRMS (APPI): *m/z* [M-Cl]$^+$ calcd for $C_{17}H_{21}ClNO_4P$-HCl·H$^+$, 334.1202, found 334.1204.

(2*R*,3*R*,4*S*)-2-methyl-4-phenylpyrrolidin-3-ylphosphonic acid hydrochloride (11d).

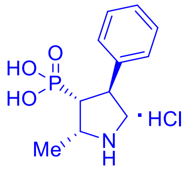

Yield: 339 mg (94%), white crystals, m.p. 214–215 °C (decomp.).

$[\alpha]_D^{20}$ +64.3 (*c* 1.0, H_2O).

^1H NMR (400 MHz, D_2O, 298 K) δ [ppm] 7.27-7.21 (m, 5H, aromatic), 4.17-4.12 (m, 1H, H-2), 3.81-3.64 (m, 1H, H-5, 1H, H-4), 3.19-3.13 (m, 1H, H-5), 2.62-2.54 (m, 1H, H-3), 1.43 (d, $^3J_{HH}$ = 6.4 Hz, 3H, Me).

^{13}C NMR (101 MHz, D_2O, 298 K) δ [ppm] 139.4 (s, aromatic, C), 129.0 (s, aromatic, 2CH), 127.8 (s, aromatic, 2CH), 127.6 (s, aromatic, CH), 57.9 (s, $^2J_{CP}$ 3.8 Hz, C-2), 51.0 (d, $^3J_{CP}$ = 12.4 Hz, C-5), 48.0 (d, $^1J_{CP}$ = 139.9 Hz, C-3), 44.1 (s, C-4), 14.8 (d, $^3J_{CP}$ = 2.8 Hz, Me).

^{31}P NMR (162 MHz, D_2O, 298 K) δ [ppm] 16.35.

IR (ATR) ν [cm^{-1}] 2964 (s), 2750 (s), 1544 (m), 1494 (m), 1460 (m), 1361 (m), 1274 (m), 1190 (vs), 1130 (s), 1062 (vs), 1026 (vs), 1012 (vs), 950 (vs), 771 (s), 756 (s), 702 (vs), 673 (s), 557 (vs), 495 (vs).

HRMS (APPI): *m*/*z* $[M-Cl]^+$ calcd for $C_{11}H_{17}ClNO_3P$-HCl·H^+, 242.0940, found 242.0934.

5. Synthesis of tetrahydro-2*H*-pyran-3-ylphosphonates

General procedure for the synthesis of tetrahydro-2*H*-pyran-3-ylphosphonates 13a–f

The aldehyde **12a–f** (15.9 mmol), K_2CO_3 (219 mg, 1.59 mmol), benzyltriethylammonium chloride (36 mg, 0.16 mmol) and water (2 mL) were added successively to a solution of phosphonate **6e** (0.50 g, 1.59 mmol) in THF (15 mL). The resulting mixture was stirred for 72 h at rt. Then, the mixture was extracted with CH_2Cl_2 (3 × 10 mL). The combined extracts were dried (Na_2SO_4) and evaporated at reduced pressure. Purification by column chromatography on silica gel, eluting with $CHCl_3$/MeOH (98:2), followed by recrystallization from methanol gave the desired compounds **13a–f**.

Dimethyl [(2*S*,3*R*,4*S*,5*S*,6*R*)-6-ethyl-2-hydroxy-2-methyl-5-nitro-4-phenyltetrahydro-2*H*-pyran-3-yl)phosphonate (13a).

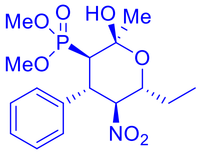

Yield: 206 mg (34%), white crystals, m.p. 173–174 °C (methanol).

$[\alpha]_D^{20}$ +42.4 (*c* 1.0, $CHCl_3$). *dr* 1:-.

^{1}H NMR (400 MHz, $CDCl_3$, 298 K) δ [ppm] 7.29-7.16 (m, 5H, aromatic), 4.47 (td, $^{3}J_{HH}$ = 10.8 Hz, $^{4}J_{HP}$ = 0.8 Hz, 1H, H-5), 4.38-4.33 (m, 1H, H-6), 3.91 (s, 1H, OH), 3.93 (td, $^{3}J_{HH}$ = 12.0 Hz, $^{3}J_{HP}$ = 8.9 Hz, 1H, H-4), 3.56 (d, $^{3}J_{HP}$ = 10.8 Hz, 3H, MeO), 3.19 (d, $^{3}J_{HP}$ = 11.1 Hz, 3H, MeO), 2.50 (ddd, $^{2}J_{HP}$ = 18.4, $^{3}J_{HH}$ = 12.4 Hz, $^{4}J_{HH}$ = 1.2 Hz, 1H, H-3), 1.82 (s, 3H, Me), 1.59-1.41 (m, 2H, CH_2), 0.95 (t, $^{3}J_{HH}$ = 7.6 Hz, 3H, **Me**CH_2).

^{13}C NMR (101 MHz, $CDCl_3$, 298 K) δ [ppm] 137.2 (s, aromatic), 128.6 (s, aromatic), 128.4 (s, aromatic), 128.0 (s, aromatic), 95.9 (s, C-2), 92.8 (d, $^{3}J_{CP}$ = 14.3 Hz, C-5), 70.5 (s, C-6), 53.5 (d, $^{2}J_{CP}$ = 6.7 Hz, MeO), 51.6 (d, $^{2}J_{CP}$ = 6.7 Hz, MeO), 48.4 (d, $^{1}J_{CP}$ = 138.9 Hz, C-3), 44.3 (s, C-4), 29.4 (s, Me), 24.8 (s, CH_2), 8.9 (s, Me).

^{31}P NMR (162 MHz, $CDCl_3$, 298 K) δ [ppm] 27.29.

IR (ATR) ν [cm^{-1}] 3267 (m), 2962 (w), 2926 (w), 2856 (w), 1550 (vs), 1377 (m), 1240 (vs), 1028 (vs), 813 (s), 702 (s), 520 (s).

HRMS (APPI): *m*/*z* $[M-H_2O+H]^+$ calcd for $C_{16}H_{24}NO_7P-H_2O\cdot H^+$ 356.1257; found 356.1258.

Dimethyl [(2*S*,3*R*,4*S*,5*S*,6*R*)-2-hydroxy-2,6-dimethyl-5-nitro-4-phenyltetrahydro-2*H*-pyran-3-yl]phosphonate (13b).

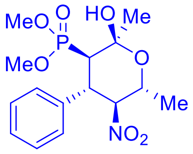

Yield: 202 mg (36%), white crystals, m.p. 193–194 °C (methanol).

$[\alpha]_D^{20}$ +34.1 (*c* 1.0, $CHCl_3$). *dr* 14:1, > 99% *ee*.

^{1}H NMR (400 MHz, $CDCl_3$, 298 K) δ [ppm] 7.31-7.16 (m, 5H, aromatic), 4.59-4.52 (m, 1H, H-6), 4.39 (td, $^{3}J_{HH}$ = 11.2 Hz, $^{4}J_{HP}$ = 1.2 Hz, 1H, H-5), 4.00 (s, 1H, OH), 3.93 (td, $^{3}J_{HH}$ = 12.0 Hz, $^{3}J_{HP}$ = 8.8 Hz, 1H, H-4), 3.56 (d, $^{3}J_{HP}$ = 10.7 Hz, 3H, MeO), 3.20 (d, $^{3}J_{HP}$

= 11.1 Hz, 3H, MeO), 2.50 (ddd, $^2J_{HP}$ = 18.7, $^3J_{HH}$ = 12.5 Hz, $^4J_{HH}$ = 1.3 Hz, 1H, H-3), 1.80 (s, 3H, Me), 1.20 (d, $^3J_{HH}$ = 6.4 Hz, 3H, Me).

^{13}C NMR (101 MHz, $CDCl_3$, 298 K) δ [ppm] 137.1 (s, aromatic), 128.6 (s, aromatic), 128.3 (s, aromatic), 128.0 (s, aromatic), 96.0 (s, C-2), 94.6 (d, $^3J_{CP}$ = 14.3 Hz, C-5), 66.2 (s, C-6), 53.5 (d, $^2J_{CP}$ = 6.7 Hz, MeO), 51.7 (d, $^2J_{CP}$ = 6.7 Hz, MeO), 48.3 (d, $^1J_{CP}$ = 138.0 Hz, C-3), 44.0 (s, C-4), 29.5 (s, Me), 18.1 (s, Me).

^{31}P NMR (162 MHz, $CDCl_3$, 298 K) δ [ppm] 27.32.

IR (ATR) ν [cm^{-1}] 3250 (m), 2958 (w), 2854 (w), 1544 (vs), 1379 (m), 1238 (vs), 1014 (vs), 740 (s), 700 (s), 545 (s).

HRMS (APPI): *m/z* $[M\text{-}H_2O\text{+}H]^+$ calcd for $C_{15}H_{22}NO_7P\text{-}H_2O\cdot H^+$ 342.1101; found 342.1096.

HPLC analysis (Chiralpak AD-3 column; hexane/*i*-PrOH, 92:8; flow rate 1.2 mL/min; wavelength 230 nm): t_R = 7.0 (2*R*,3*S*,4*R*,5*R*,6*S*), 7.7 (2*S*,3*R*,4*S*,5*S*,6*R*) min.

Dimethyl [(2*S*,3*R*,4*S*,5*S*,6*R*)-2-hydroxy-2-methyl-5-nitro-4-phenyl-6-propyl-tetrahydro-2*H*-pyran-3-yl)phosphonate (13c).

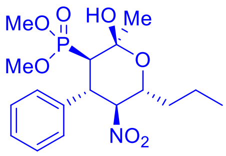

Yield: 216 mg (35%), white crystals, m.p. 180–181 °C (methanol).

$[\alpha]_D^{20}$ +55.6 (*c* 1.0, $CHCl_3$). *dr* 10:1.

^{1}H NMR (300 MHz, $CDCl_3$, 298 K) δ [ppm] 7.34-7.19 (m, 5H, aromatic), 4.48 (s, 1H, OH), 4.52-4.45 (m, 1H, H-5), 4.05-3.89 (m, 1H, H-4), 3.80-3.63 (m, 1H, H-6), 3.57 (d, $^3J_{HP}$ = 11.1 Hz, 3H, MeO), 3.24 (d, $^3J_{HP}$ = 11.1 Hz, 3H, MeO), 2.53 (dd, $^2J_{HP}$ = 18.3, $^3J_{HH}$ = 12.6 Hz, 1H, H-3), 1.84 (s, 3H, Me), 1.62-1.28 (m, 4H, $2CH_2$), 0.91 (t, $^3J_{HH}$ = 6.6 Hz, 3H, **Me**CH_2).

^{13}C NMR (75 MHz, $CDCl_3$, 298 K) δ [ppm] 137.2 (s, aromatic), 128.5 (s, aromatic), 128.2 (s, aromatic), 127.9 (s, aromatic), 95.8 (s, C-2), 93.1 (d, $^3J_{CP}$ = 14.3 Hz, C-5), 69.3 (s, C-6), 53.3 (d, $^2J_{CP}$ = 6.6 Hz, MeO), 51.5 (d, $^2J_{CP}$ = 6.7 Hz, MeO), 48.2 (d, $^1J_{CP}$ = 138.3 Hz, C-3), 44.2 (s, C-4), 33.6 (s, CH_2), 29.5 (s, Me), 17.8 (s, CH_2), 13.7 (s, Me).

^{31}P NMR (121 MHz, $CDCl_3$, 298 K) δ [ppm] 26.67.

IR (ATR) ν [cm^{-1}] 3275 (m), 2954 (w), 2929 (w), 2872 (w), 1546 (vs), 1379 (m), 1238 (vs), 1024 (vs), 810 (s), 779 (s), 702 (s), 520 (vs).

HRMS (APPI): *m/z* $[M-H_2O+H]^+$ calcd for $C_{17}H_{26}NO_7P-H_2O·H^+$ 370.1414; found 370.1418.

Dimethyl [(2*S*,3*R*,4*S*,5*S*,6*R*)-2-hydroxy-6-isobutyl-2-methyl-5-nitro-4-phenyl-tetrahydro 2*H*-pyran-3-yl)phosphonate (13d).

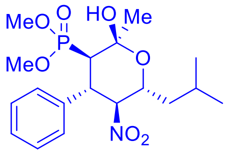

Yield: 191 mg (30%), white crystals, m.p. 153–154 °C (methanol).

$[\alpha]_D^{20}$ +51.5 (*c* 1.0, $CHCl_3$). *dr* 9:1.

^1H NMR (400 MHz, $CDCl_3$, 298 K) δ [ppm] 7.29-7.15 (m, 5H, aromatic), 4.48 (td, $^3J_{HH}$ = 9.9 Hz, $^4J_{HP}$ = 1.6 Hz, 1H, H-5), 4.41 (t, $^3J_{HH}$ = 9.9 Hz, 1H, H-6), 4.13 (s, 1H, OH), 3.97-3.89 (m, 1H, H-4), 3.56 (d, $^3J_{HP}$ = 10.7 Hz, 3H, MeO), 3.18 (d, $^3J_{HP}$ = 11.2 Hz, 3H, MeO), 2.49 (dd, $^2J_{HP}$ = 18.5, $^3J_{HH}$ = 12.3 Hz, 1H, H-3), 1.81 (s, 3H, Me), 1.53-1.46 (m, 1H, CH_2), 1.13-1.06 (m, 1H, CH_2), 0.89-0.84 (m, 1H, CH, *i*-Bu), 0.88 (d, $^3J_{HH}$ = 6.8 Hz, 3H, **Me**CH), 0.84 (d, $^3J_{HH}$ = 6.4 Hz, 3H, **Me**CH).

^{13}C NMR (101 MHz, $CDCl_3$, 298 K) δ [ppm] 137.2 (s, aromatic), 128.6 (s, aromatic), 128.3 (s, aromatic), 128.0 (s, aromatic), 95.8 (s, C-2), 93.7 (d, $^3J_{CP}$ 14.3 Hz, C-5), 67.9 (s, C-6), 53.5 (d, $^2J_{CP}$ 6.7 Hz, MeO), 51.6 (d, $^2J_{CP}$ 6.7 Hz, MeO), 48.5 (d, $^1J_{CP}$ 138.0 Hz, C-3), 44.4 (s, C-4), 40.4 (s, CH_2, *i*-Bu), 29.4 (s, Me), 23.9 (s, CH, *i*-Bu), 23.6 (s, CH_3, *i*-Bu), 21.2 (s, CH_3, *i*-Bu).

^{31}P NMR (162 MHz, $CDCl_3$, 298 K) δ [ppm] 27.37.

IR (ATR) ν [cm^{-1}] 3280 (m), 2954 (w), 2870 (w), 1548 (vs), 1369 (m), 1232 (vs), 1033 (vs), 761 (s), 698 (s).

HRMS (APPI): *m/z* $[M-H_2O+H]^+$ calcd for $C_{18}H_{28}NO_7P-H_2O·H^+$ 384.1570; found 384.1575.

Dimethyl [(2*S*,3*R*,4*S*,5*S*,6*R*)-2-hydroxy-2-methyl-5-nitro-4,6-diphenyltetrahydro-2*H*-pyran-3-yl]phosphonate (13e).

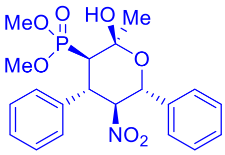

Yield: 214 mg (32%), white crystals, m.p. 197–198 °C (methanol).

$[\alpha]_D^{20}$ +66.0 (*c* 0.5, $CHCl_3$). *dr* 1:-.

^{1}H NMR (400 MHz, $CDCl_3$, 298 K) δ [ppm] 7.33-7.21 (m, 10H, aromatic), 5.46 (d, $^3J_{HH}$ = 9.8 Hz, 1H, H-6), 4.67 (td, $^3J_{HH}$ = 9.8 Hz, $^4J_{HP}$ = 1.1 Hz, 1H, H-5), 4.12 (td, $^3J_{HH}$ = 11.6 Hz, $^3J_{HP}$ = 8.7 Hz, 1H, H-4), 3.61 (d, $^3J_{HP}$ = 10.9 Hz, 3H, MeO), 3.23 (d, $^3J_{HP}$ = 10.9 Hz, 3H, MeO), 2.67 (dd, $^2J_{HP}$ = 18.7, $^3J_{HH}$ = 12.4 Hz, 1H, H-3), 1.92 (s, 3H, Me).

^{13}C NMR (101 MHz, $CDCl_3$, 298 K) δ [ppm] 136.8 (s, aromatic), 135.9 (s, aromatic), 129.4 (s, aromatic), 128.9 (s, aromatic), 128.6 (s, aromatic), 128.1 (s, aromatic), 127.1 (s, aromatic), 97.1 (s, C-2), 94.6 (d, $^3J_{CP}$ = 14.3 Hz, C-5), 72.8 (s, C-6), 53.6 (d, $^2J_{CP}$ = 6.7 Hz, MeO), 51.7 (d, $^2J_{CP}$ = 6.7 Hz, MeO), 48.2 (d, $^1J_{CP}$ = 138.0 Hz, C-3), 44.7 (s, C-4), 29.7 (s, Me).

^{31}P NMR (162 MHz, $CDCl_3$, 298 K) δ [ppm] 26.85.

IR (ATR) ν [cm^{-1}] 3300 (m), 3188 (m), 2954 (w), 2856 (w), 1550 (vs), 1375 (m), 1238 (vs), 1041 (vs), 761 (s), 750 (s), 696 (vs), 528 (s), 520 (s).

HRMS (APPI): *m*/*z* $[M-H_2O+H]^+$ calcd for $C_{20}H_{24}NO_7P-H_2O\cdot H^+$ 404.1257; found 404.1257.

Dimethyl [(2*S*,3*R*,4*S*,5*S*,6*R*)-6-(4-chlorophenyl)-2-hydroxy-2-methyl-5-nitro-4-phenyltetrahydro-2*H*-pyran-3-yl]phosphonate (13f).

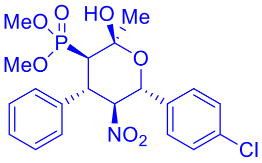

Yield: 217 mg (30%), white crystals, m.p. 183–184 °C (methanol).

$[\alpha]_D^{20}$ +66.4 (*c* 1.0, $CHCl_3$). *dr* 1:-.

^{1}H NMR (400 MHz, $CDCl_3$, 298 K) δ [ppm] 7.31-7.20 (m, 9H, aromatic), 5.44 (d, $^3J_{HH}$ = 9.8 Hz, 1H, H-6), 4.60 (t, $^3J_{HH}$ = 11.2 Hz, 1H, H-5), 4.10 (td, $^3J_{HH}$ = 11.9 Hz, $^3J_{HP}$ = 8.6 Hz, 1H, H-4), 3.59 (d, $^3J_{HP}$ = 10.9 Hz, 3H, MeO), 3.47 (s, 1H, OH), 3.24 (d, $^3J_{HP}$ = 11.2 Hz, 3H, MeO), 2.66 (dd, $^2J_{HP}$ = 18.7, $^3J_{HH}$ = 12.4 Hz, 1H, H-3), 1.91 (s, 3H, Me).

^{13}C NMR (101 MHz, $CDCl_3$, 298 K) δ [ppm] 136.6 (s, aromatic), 135.3 (s, aromatic), 134.5 (s, aromatic), 129.1 (s, aromatic), 128.7 (s, aromatic), 128.4 (s, aromatic), 128.2 (s, aromatic), 97.1 (s, C-2), 94.5 (d, $^3J_{CP}$ = 15.3 Hz, C-5), 72.2 (s, C-6), 53.6 (d, $^2J_{CP}$ = 6.7 Hz, MeO), 51.7 (d, $^2J_{CP}$ = 6.7 Hz, MeO), 48.2 (d, $^1J_{CP}$ = 138.0 Hz, C-3), 44.6 (s, C-4), 29.6 (s, Me).

^{31}P NMR (162 MHz, $CDCl_3$, 298 K) δ [ppm] 26.72.

IR (ATR) ν [cm^{-1}] 3321 (m), 2951 (w), 2916 (w), 2850 (w), 1548 (vs), 1373 (m), 1228 (vs), 1024 (vs), 900 (s), 752 (vs), 698 (s), 594 (s), 536 (s).

HRMS (APPI): *m*/*z* $[M\text{-}H_2O+H]^+$ calcd for $C_{20}H_{23}ClNO_7P\text{-}H_2O\cdot H^+$ 438.0867; found 438.0873.

Dimethyl [(2*R*,3*S*,4*S*)-2,6-dimethyl-3-nitro-4-phenyl-3,4-dihydro-2*H*-pyran-5-yl]phosphonate (14).

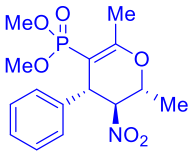

TsOH (5 mg, 0.029 mmol) was added to a solution of **13b** (206 mg, 0.573 mmol) in 8 mL of toluene and the resulting mixture was refluxed for 2 h. Then the reaction mixture was evaporated at reduced pressure. The residue was dissolved in $CHCl_3$ and filtered through silica gel (1.0 g). The filtrate was evaporated in vacuo.

Yield: 168 mg (86%); yellow oil; $[\alpha]_D^{20}$ -24.0 (*c* 1.0, $CHCl_3$).

^1H NMR (300 MHz, $CDCl_3$, 298 K) δ [ppm] 7.33-7.15 (m, 5H, aromatic), 4.48 (t, $^3J_{HH}$ = 9.3 Hz, 1H, H-5), 4.31-4.26 (m, 2H, H-4, H-6), 3.51 (d, $^3J_{HP}$ = 11.2 Hz, 3H, MeO), 3.25 (d, $^3J_{HP}$ = 11.1 Hz, 3H, MeO), 2.25 (s, 3H, Me), 1.35 (d, $^3J_{HH}$ = 6.2 Hz, 3H, Me).

^{13}C NMR (75 MHz, $CDCl_3$, 298 K) δ [ppm] 165.3 (d, $^2J_{CP}$ = 24.1 Hz, C-2), 139.1 (s, aromatic), 128.8 (s, aromatic), 127.9 (s, aromatic), 99.1 (d, $^1J_{CP}$ = 203.3 Hz, C-3), 93.4 (d, $^3J_{CP}$ = 11.8 Hz, C-5), 72.7 (s, C-6), 52.02 (d, $^2J_{CP}$ = 6.0 Hz, MeO), 51.95 (d, $^2J_{CP}$ = 6.0 Hz, MeO), 46.4 (d, $^2J_{CP}$ = 9.1 Hz, C-4), 20.1 (d, $^3J_{CP}$ = 2.2 Hz, Me), 17.2 (s, Me).

^{31}P NMR (121 MHz, $CDCl_3$, 298 K) δ [ppm] 21.79.

IR (ATR) ν [cm^{-1}] 2987 (w), 2953 (w), 2848 (w), 1620 (s), 1550 (vs) 1456 (m), 1249 (s), 1020 (vs), 754 (s), 700 (s), 596 (s).

HRMS (APPI): *m/z* $[M+H]^+$ calcd for $C_{15}H_{20}NO_6P\cdot H^+$ 342.1101; found 342.1106.

6. Copies of NMR, FTIR and mass spectra for initial compounds

^{1}H NMR spectra of methyl 2-(2-bromo-1-phenylethylidene)hydrazine-1-carboxylate **IIIa** in $CDCl_3$

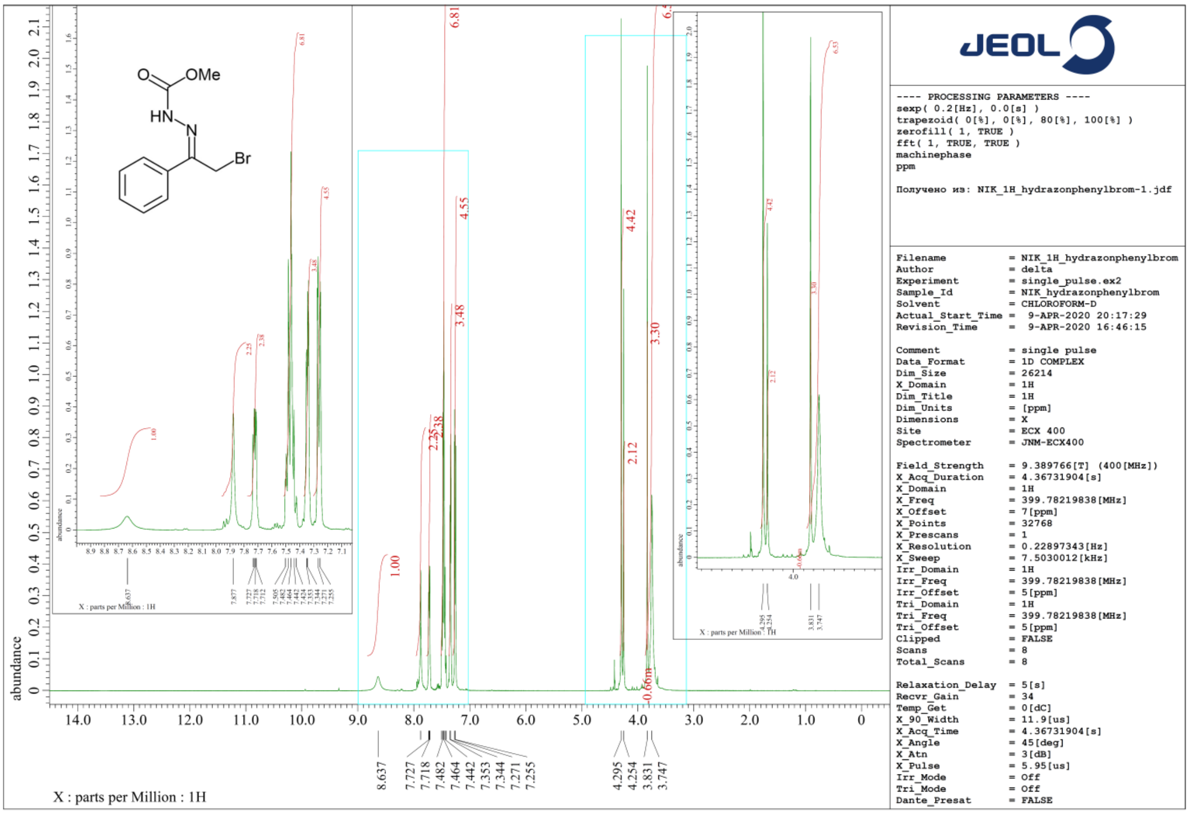

^{13}C NMR spectra of methyl 2-(2-bromo-1-phenylethylidene)hydrazine-1-carboxylate **IIIa** in $CDCl_3$

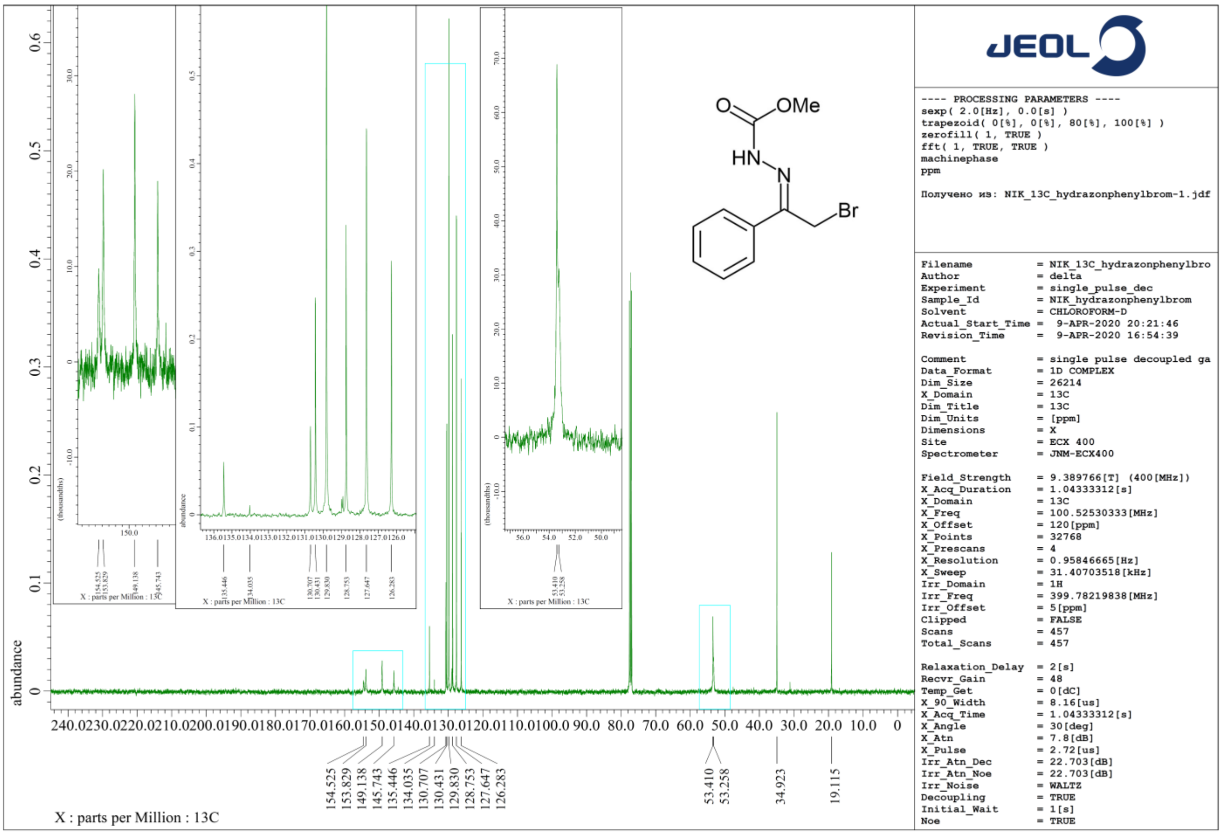

DEPT NMR spectra of methyl 2-(2-bromo-1-phenylethylidene)hydrazine-1-carboxylate **IIIa** in $CDCl_3$

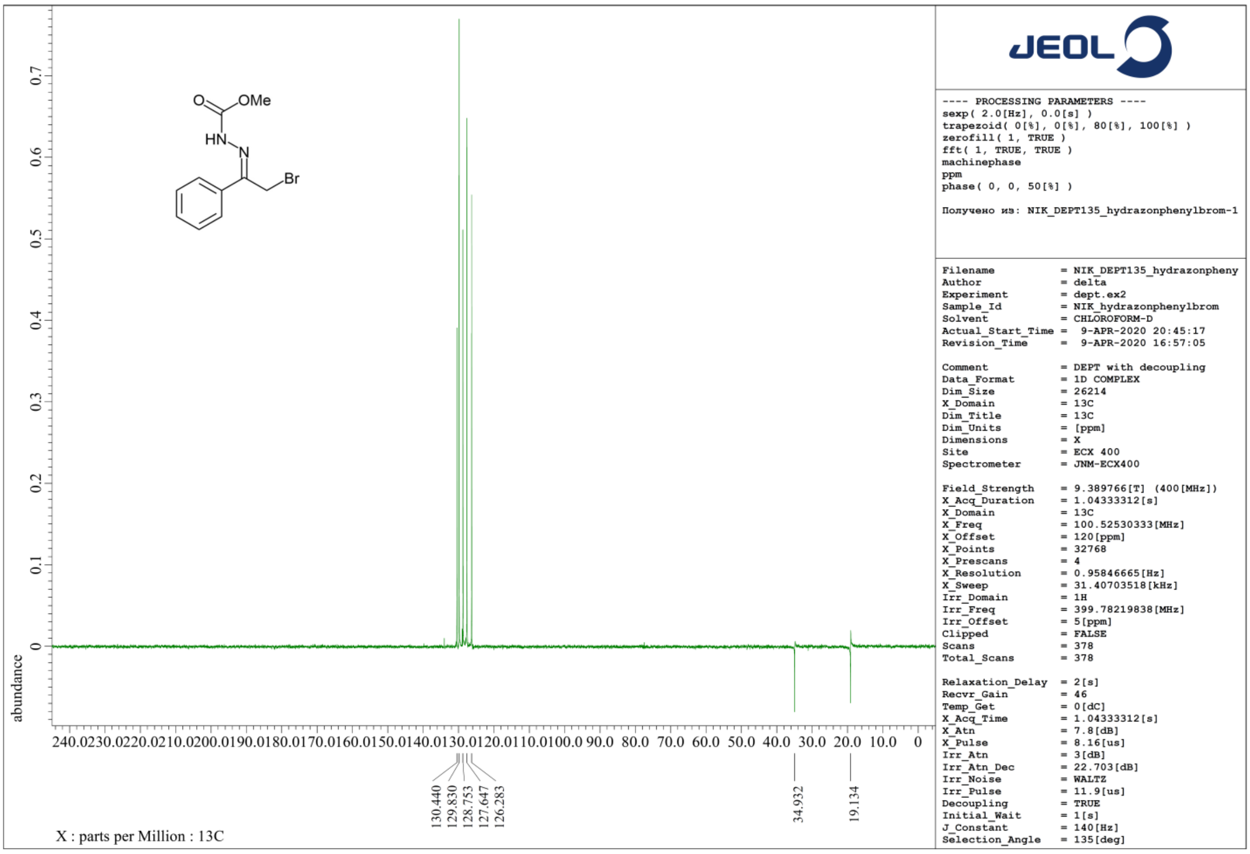

FTIR spectra of methyl 2-[2-bromo-1-(4-methoxyphenyl)ethylidene]hydrazine-1-carboxylate **IIIb**

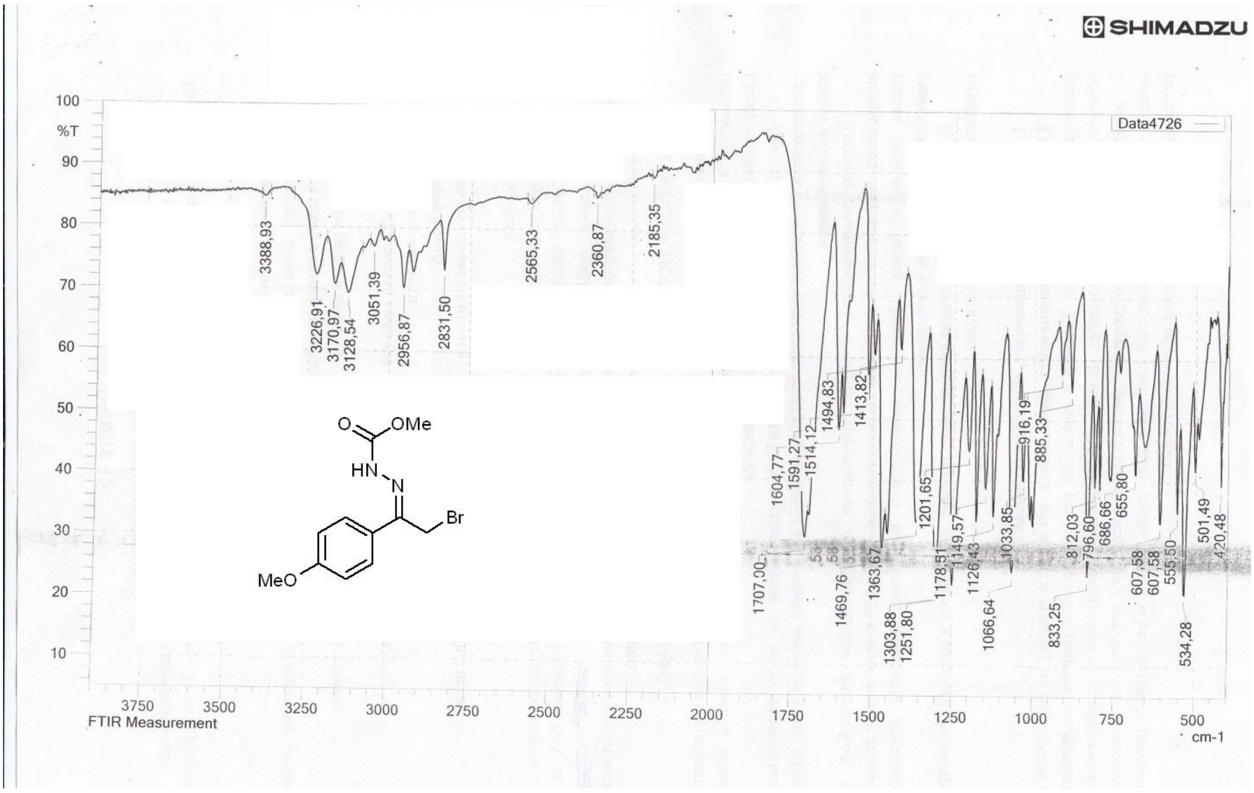

^{1}H NMR spectra of methyl 2-[2-bromo-1-(4-methoxyphenyl)ethylidene]hydrazine-1-carboxylate **IIIb** in $CDCl_3$

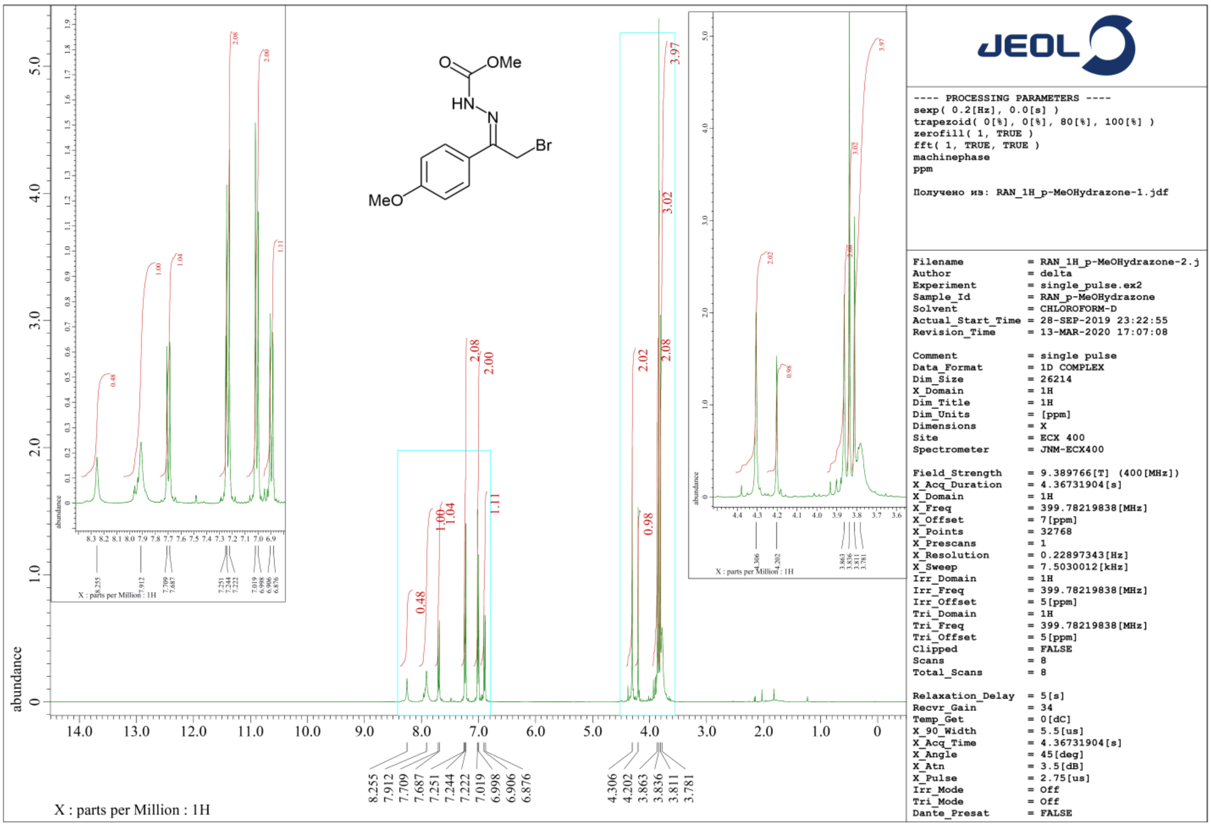

^{13}C NMR spectra of methyl 2-[2-bromo-1-(4-methoxyphenyl)ethylidene]hydrazine-1-carboxylate **IIIb** in $CDCl_3$

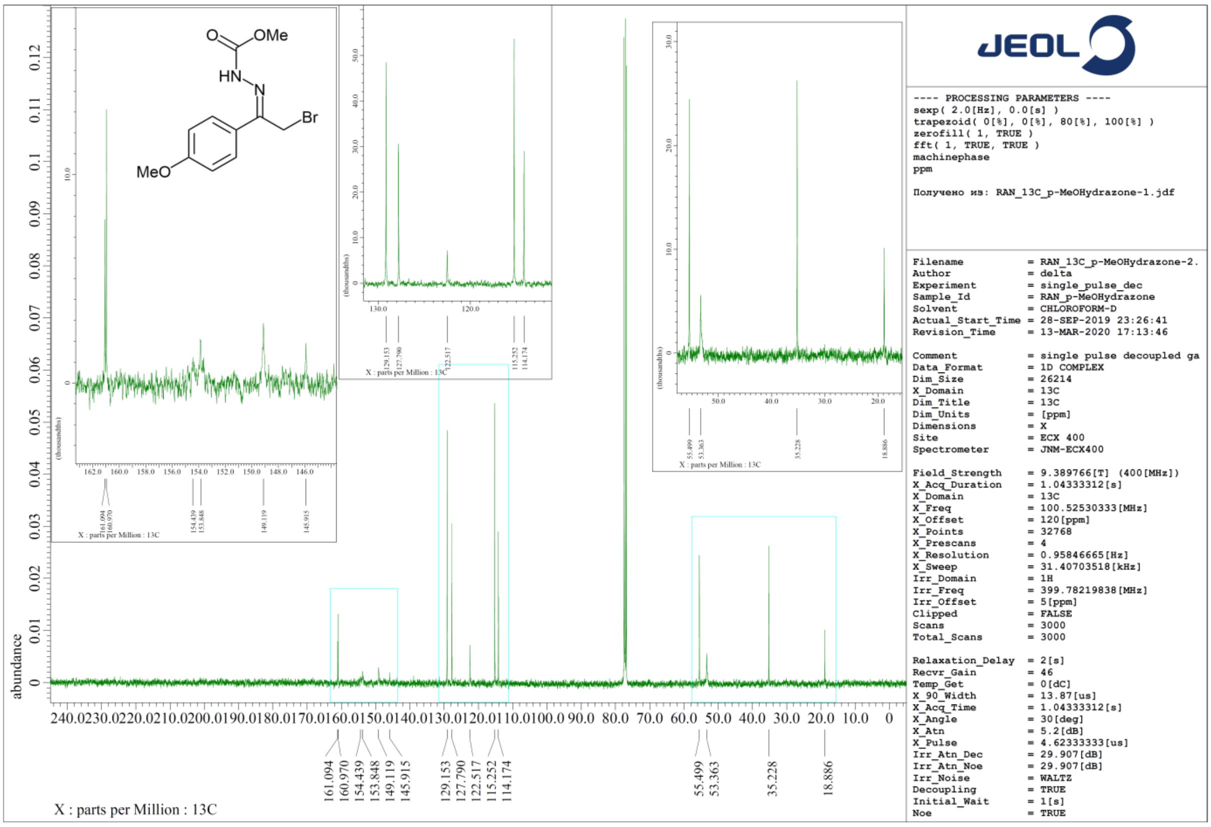

DEPT NMR spectra of methyl 2-[2-bromo-1-(4-methoxyphenyl)ethylidene]hydrazine-1-carboxylate **IIIb** in $CDCl_3$

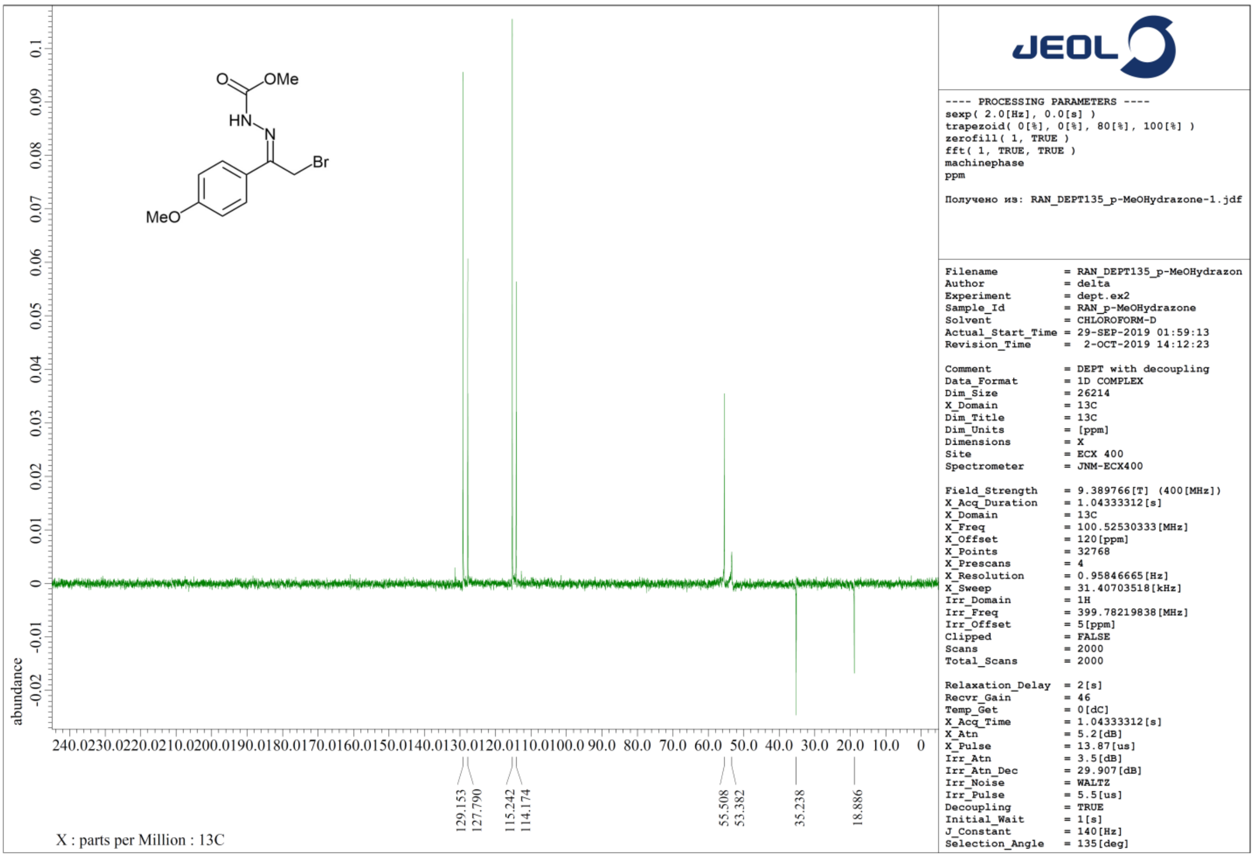

FTIR spectra of methyl 2-[2-bromo-1-(3-methoxyphenyl)ethylidene]hydrazine-1-carboxylate **IIIc**

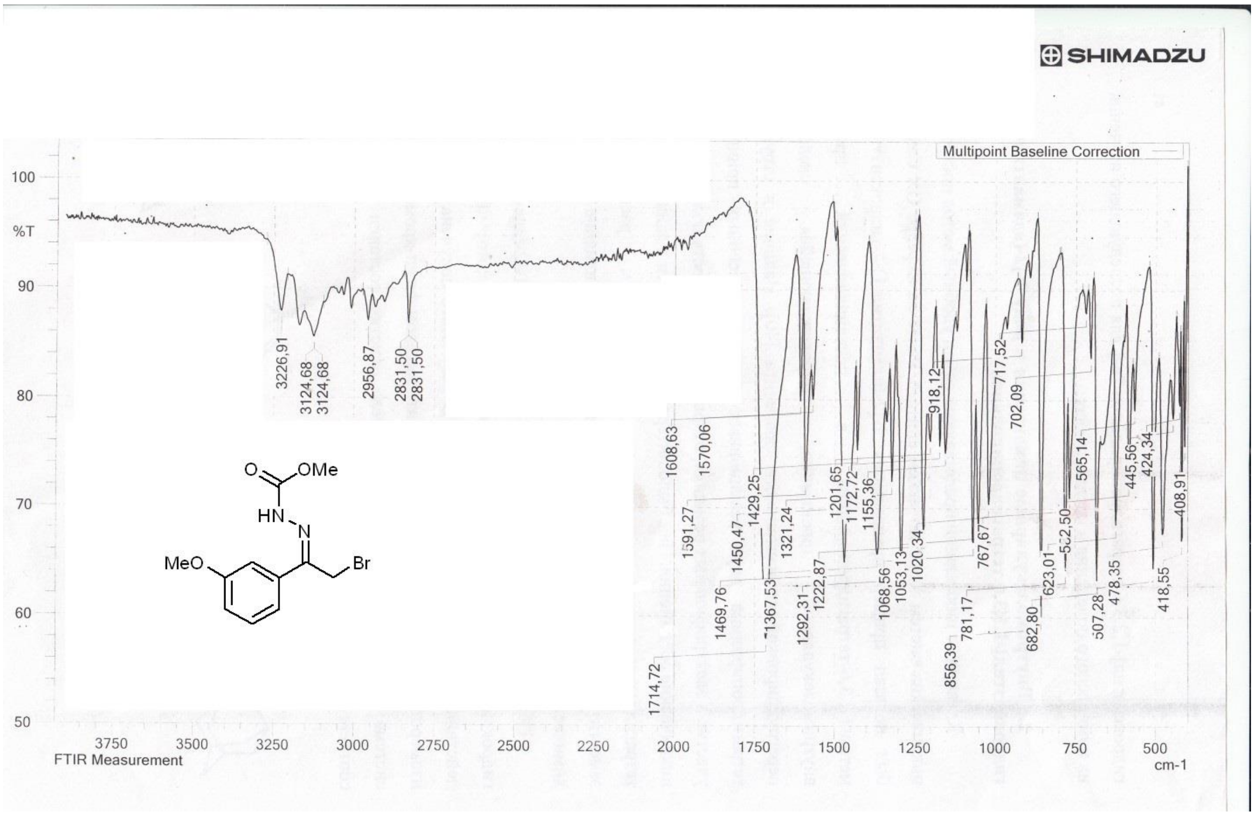

^{1}H NMR spectra of methyl 2-[2-bromo-1-(3-methoxyphenyl)ethylidene]hydrazine-1-carboxylate **IIIc** in $CDCl_3$

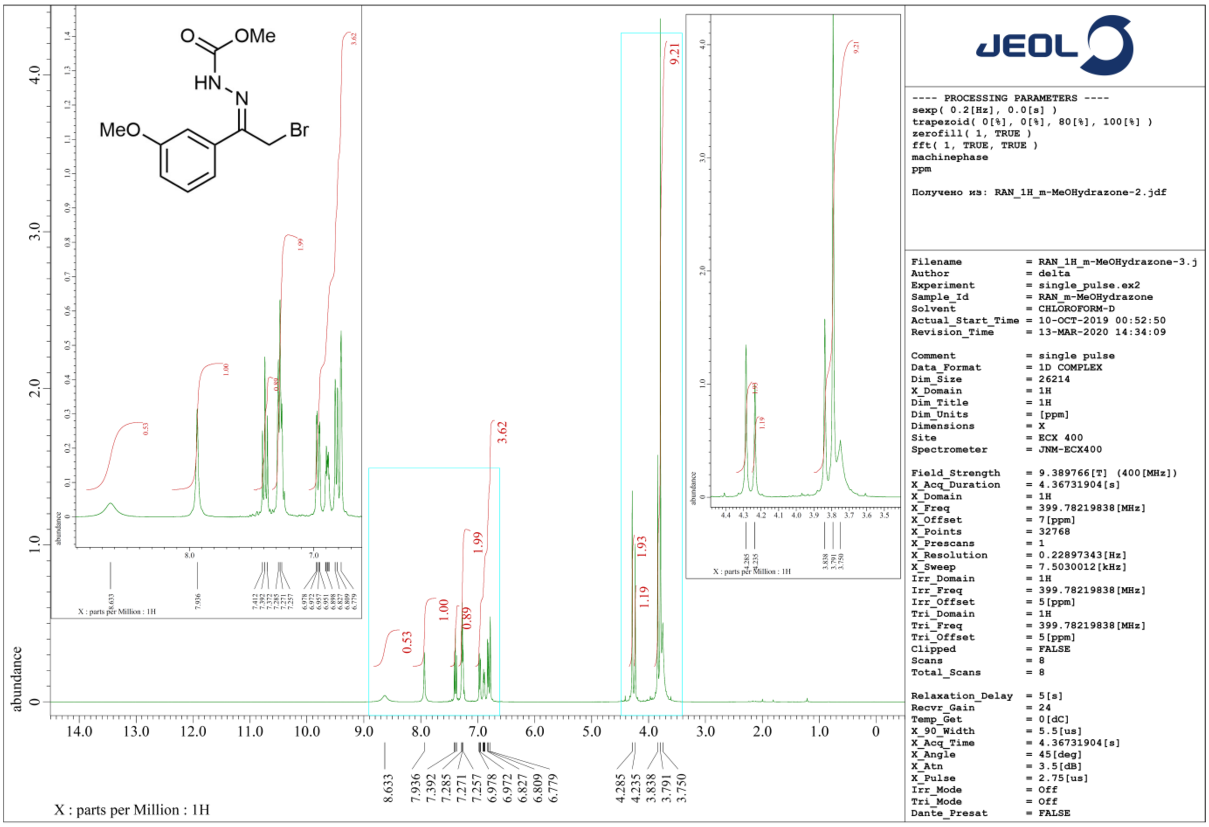

^{13}C NMR spectra of methyl 2-[2-bromo-1-(3-methoxyphenyl)ethylidene]hydrazine-1-carboxylate **IIIc** in $CDCl_3$

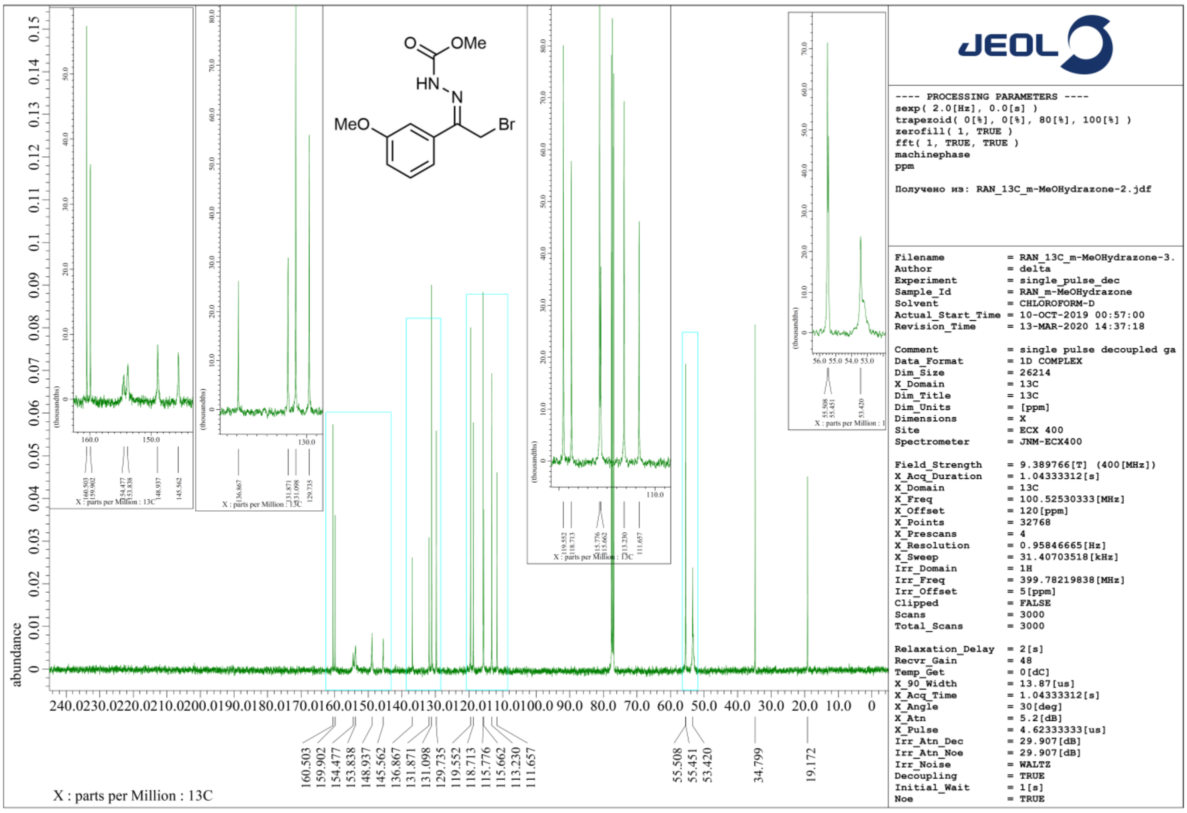

DEPT NMR spectra of methyl 2-[2-bromo-1-(3-methoxyphenyl)ethylidene]hydrazine-1-carboxylate **IIIc** in $CDCl_3$

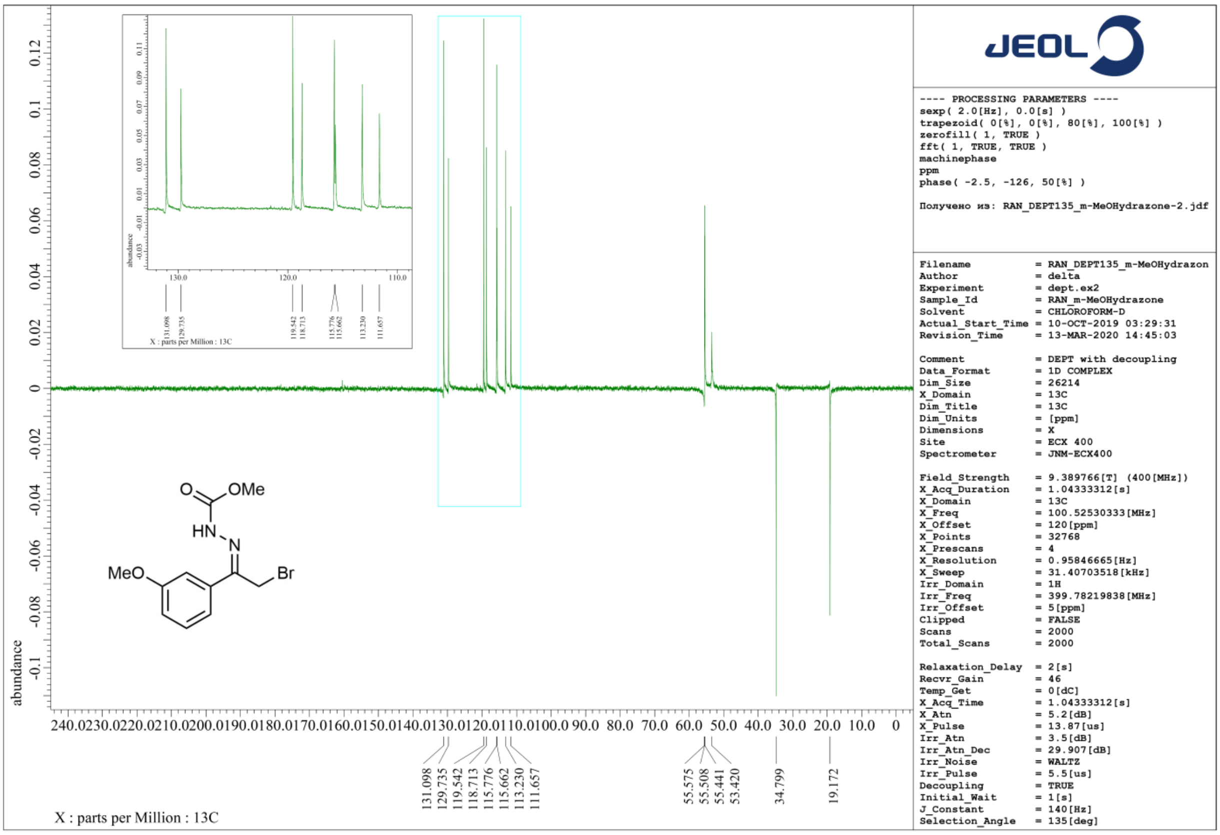

FTIR spectra of methyl 2-[2-bromo-1-(2-methoxyphenyl)ethylidene]hydrazine-1-carboxylate **IIId**

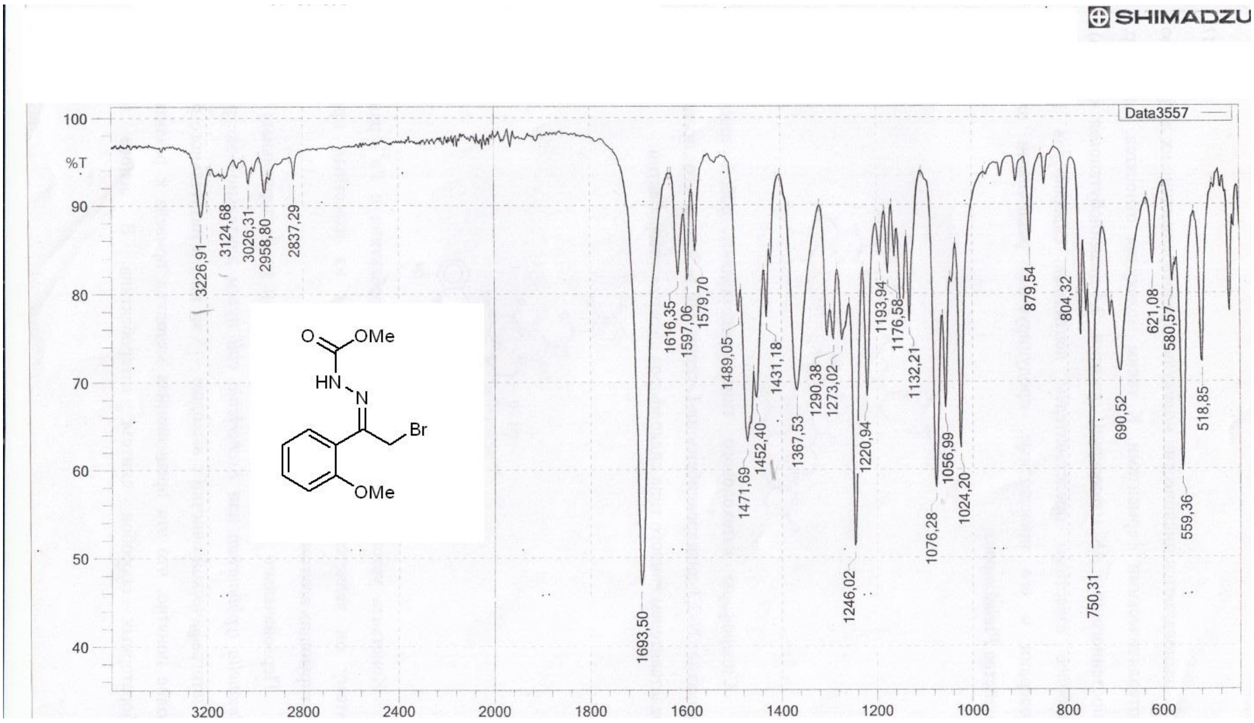

^{1}H NMR spectra of methyl 2-[2-bromo-1-(2-methoxyphenyl)ethylidene]hydrazine-1-carboxylate **IIId** in $CDCl_3$

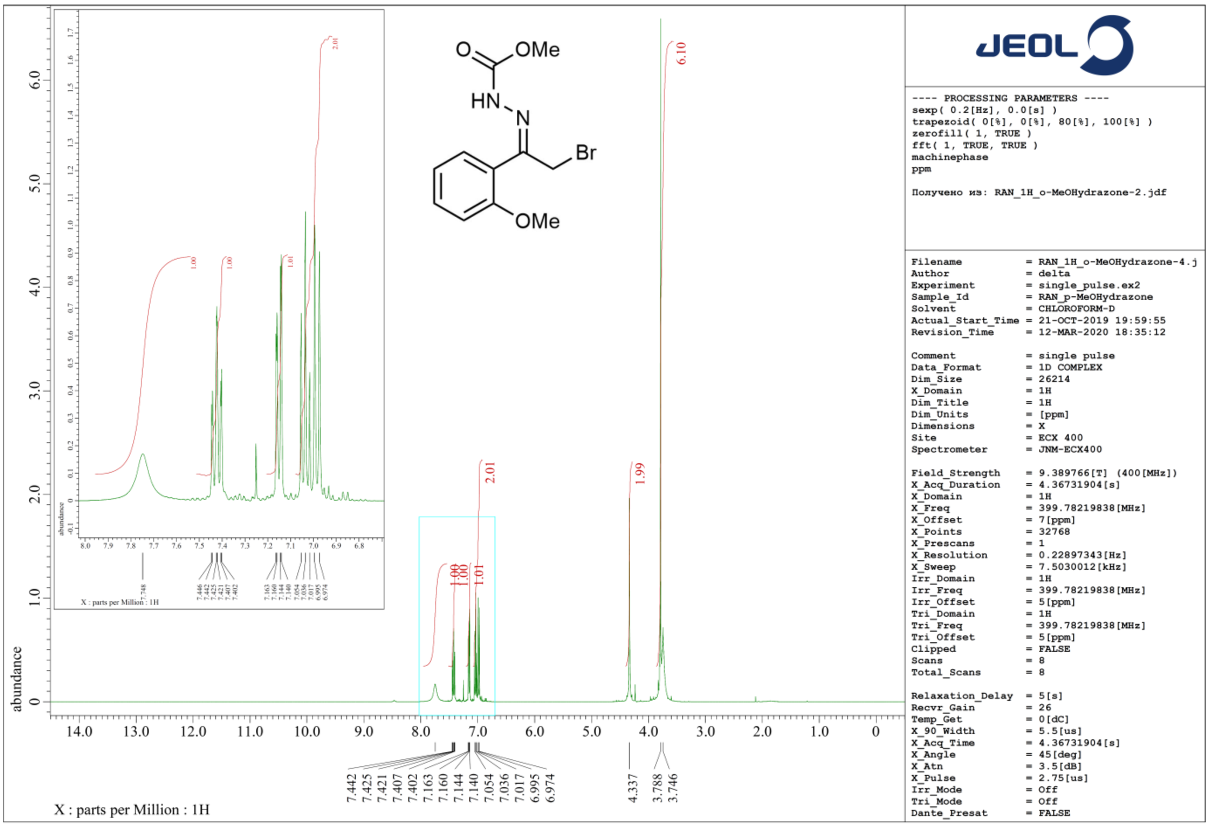

^{13}C NMR spectra of methyl 2-[2-bromo-1-(2-methoxyphenyl)ethylidene]hydrazine-1-carboxylate **IIId** in $CDCl_3$

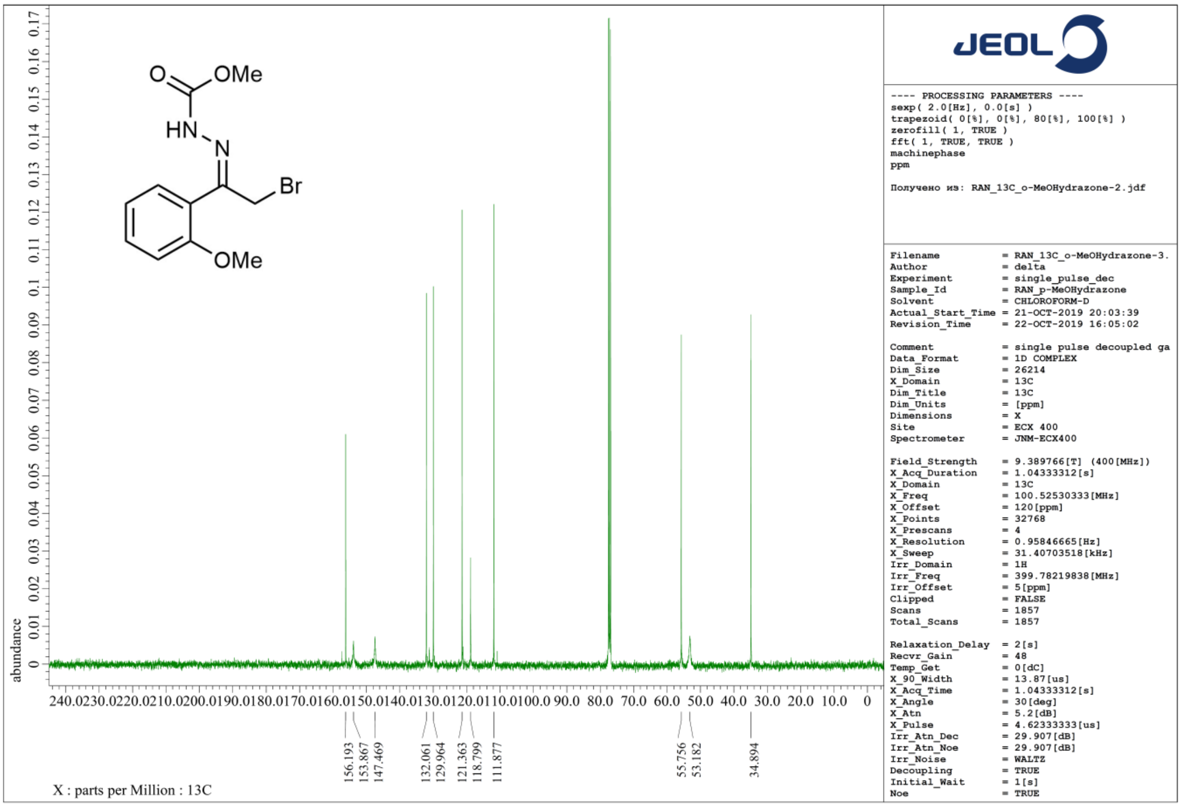

DEPT NMR spectra of methyl 2-[2-bromo-1-(2-methoxyphenyl)ethylidene]hydrazine-1-carboxylate **IIId** in $CDCl_3$

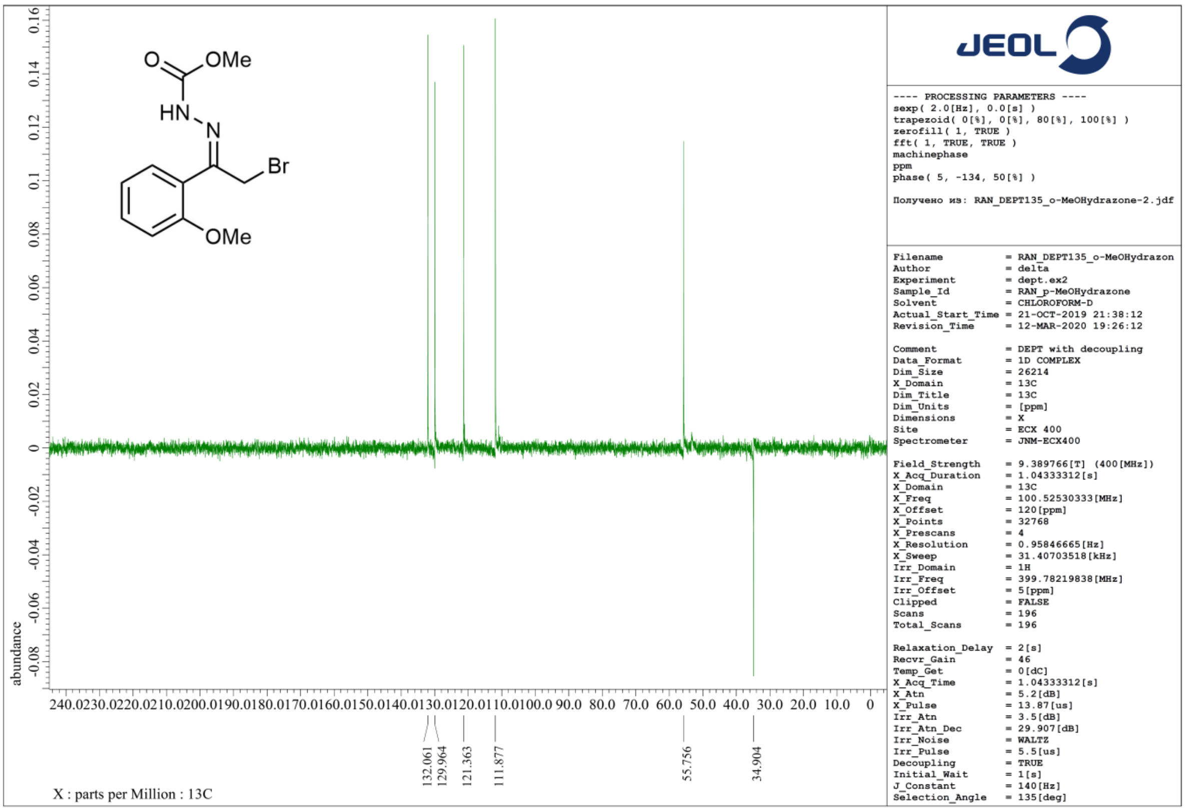

FTIR spectra of methyl 2-(1-(adamantan-1-yl)-2-bromoethylidene)hydrazine-1-carboxylate

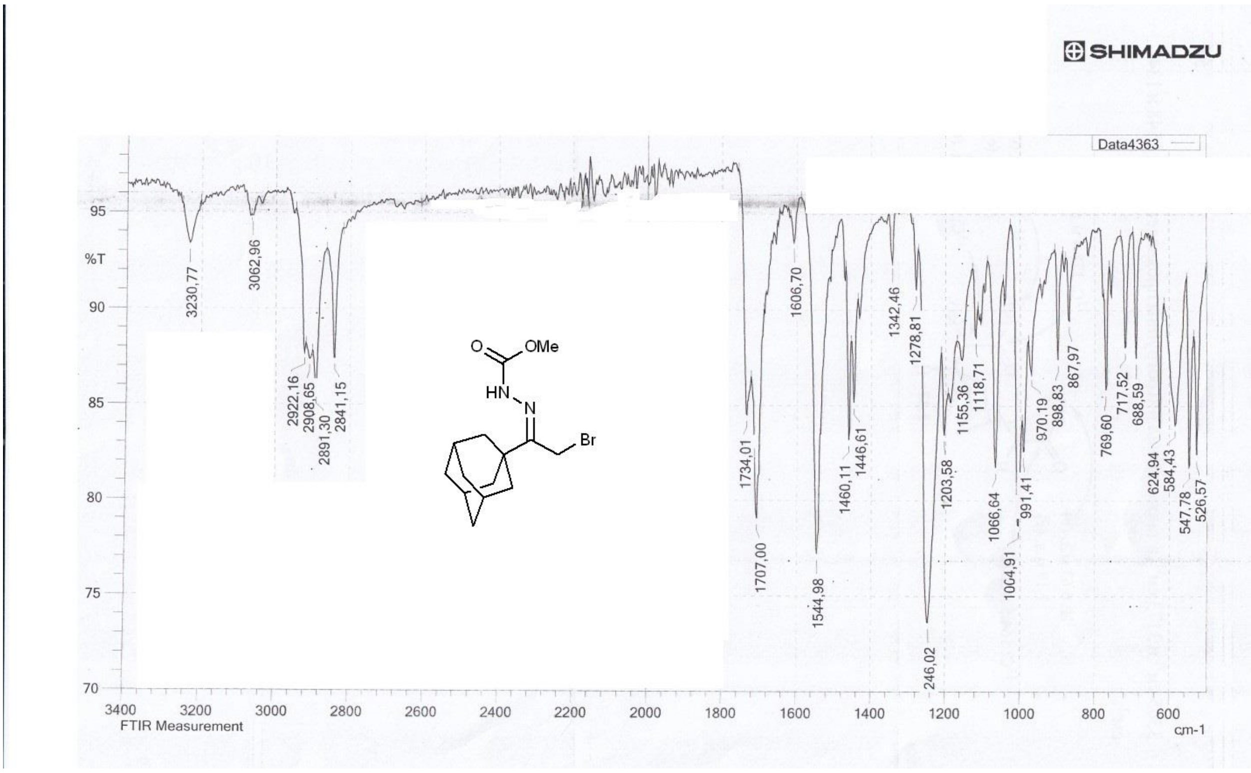

^{1}H NMR spectra of methyl 2-(1-(adamantan-1-yl)-2-bromoethylidene)hydrazine-1-carboxylate in $CDCl_3$

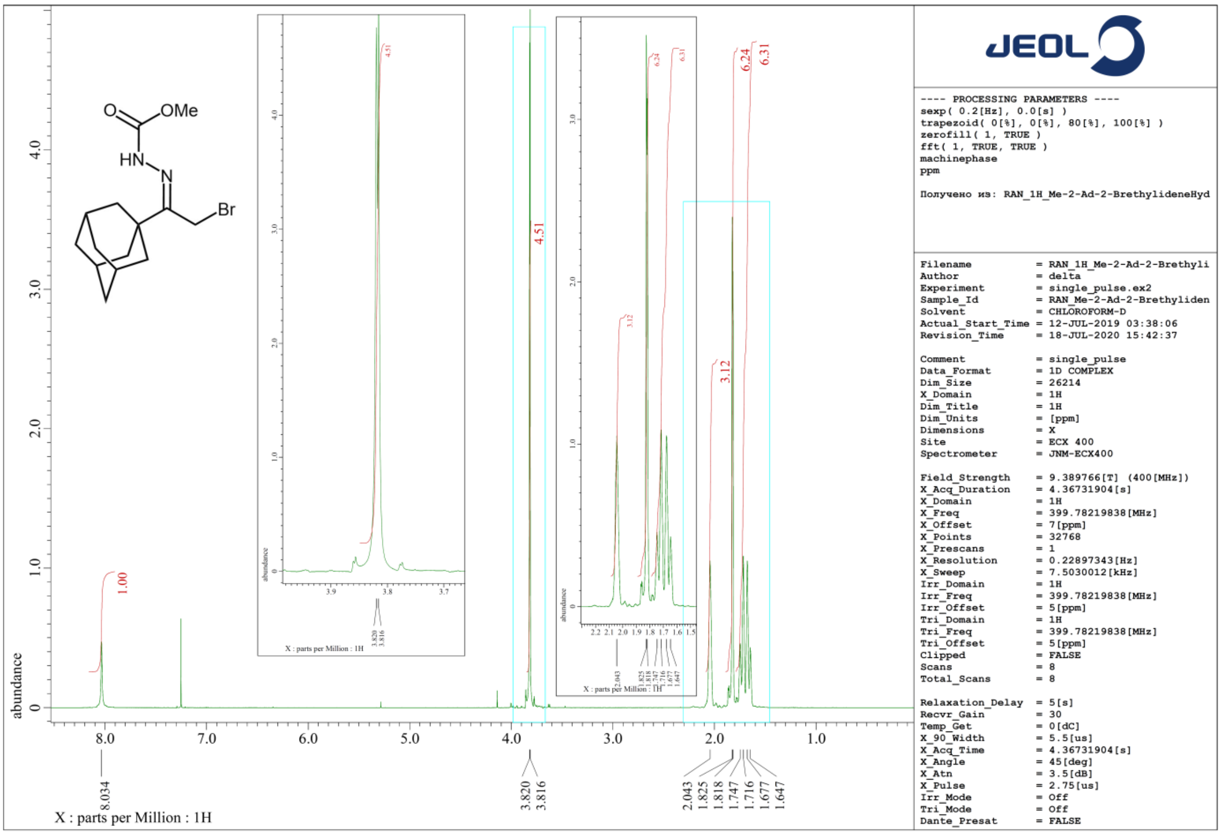

^{13}C NMR spectra of methyl 2-(1-(adamantan-1-yl)-2-bromoethylidene)hydrazine-1-carboxylate in $CDCl_3$

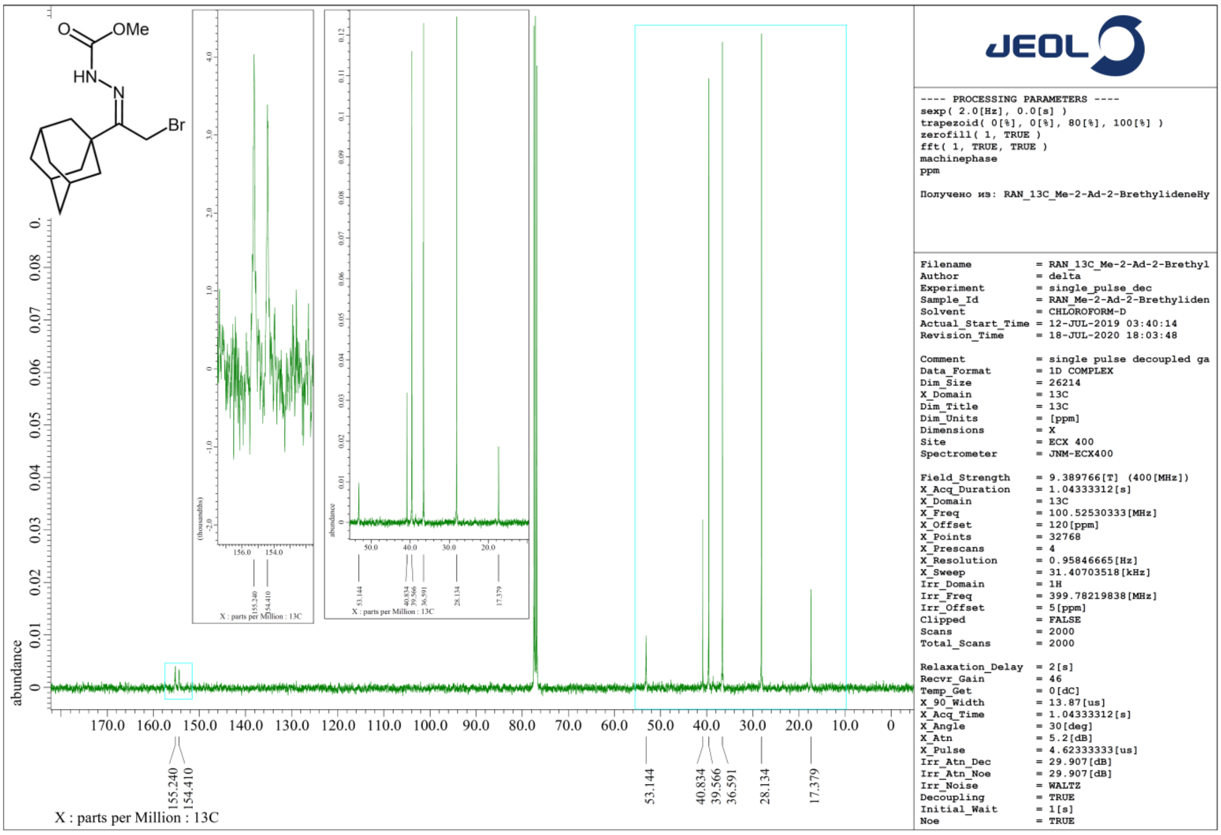

DEPT NMR spectra of methyl 2-(1-(adamantan-1-yl)-2-bromoethylidene)hydrazine-1-carboxylate in CDCl3

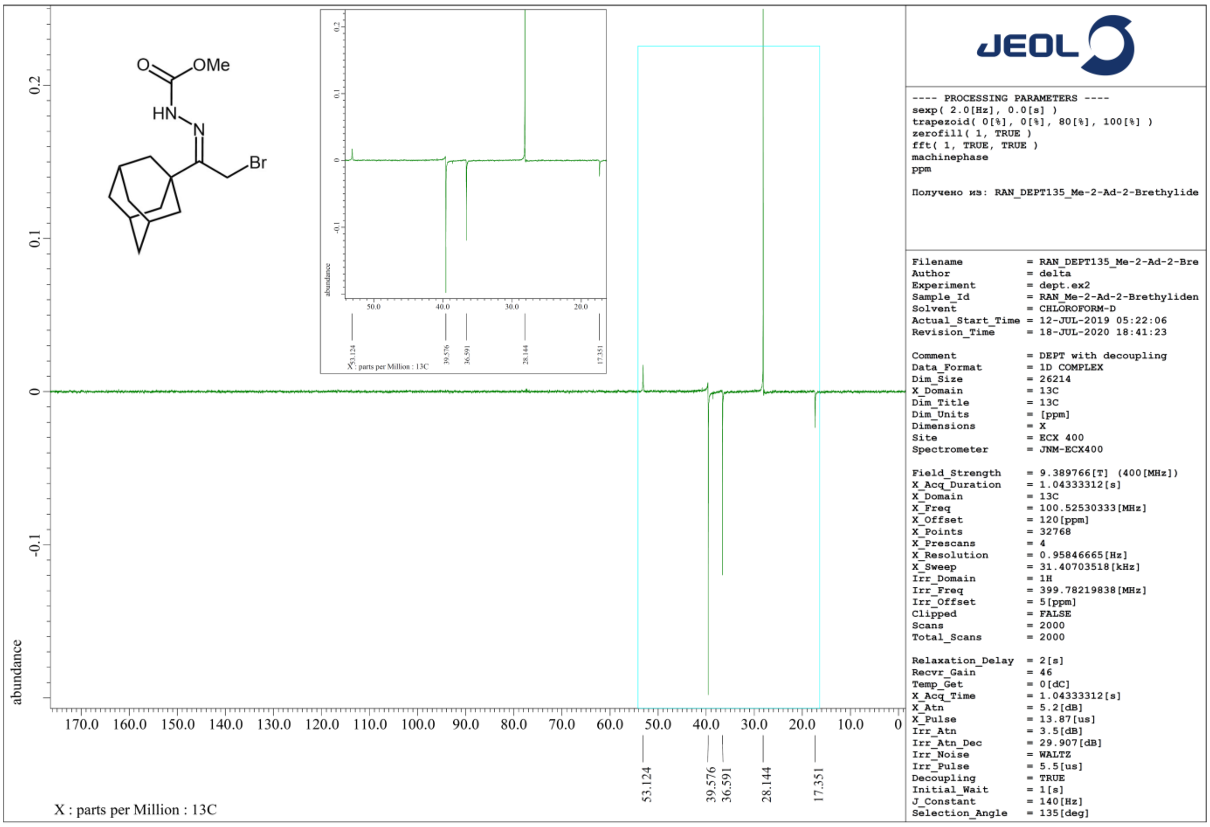

^{1}H NMR spectra of methyl 2-[2-(dimethoxyphosphoryl)-1-phenylethylidene]hydrazine-1-carboxylate **Va** in CDCl3

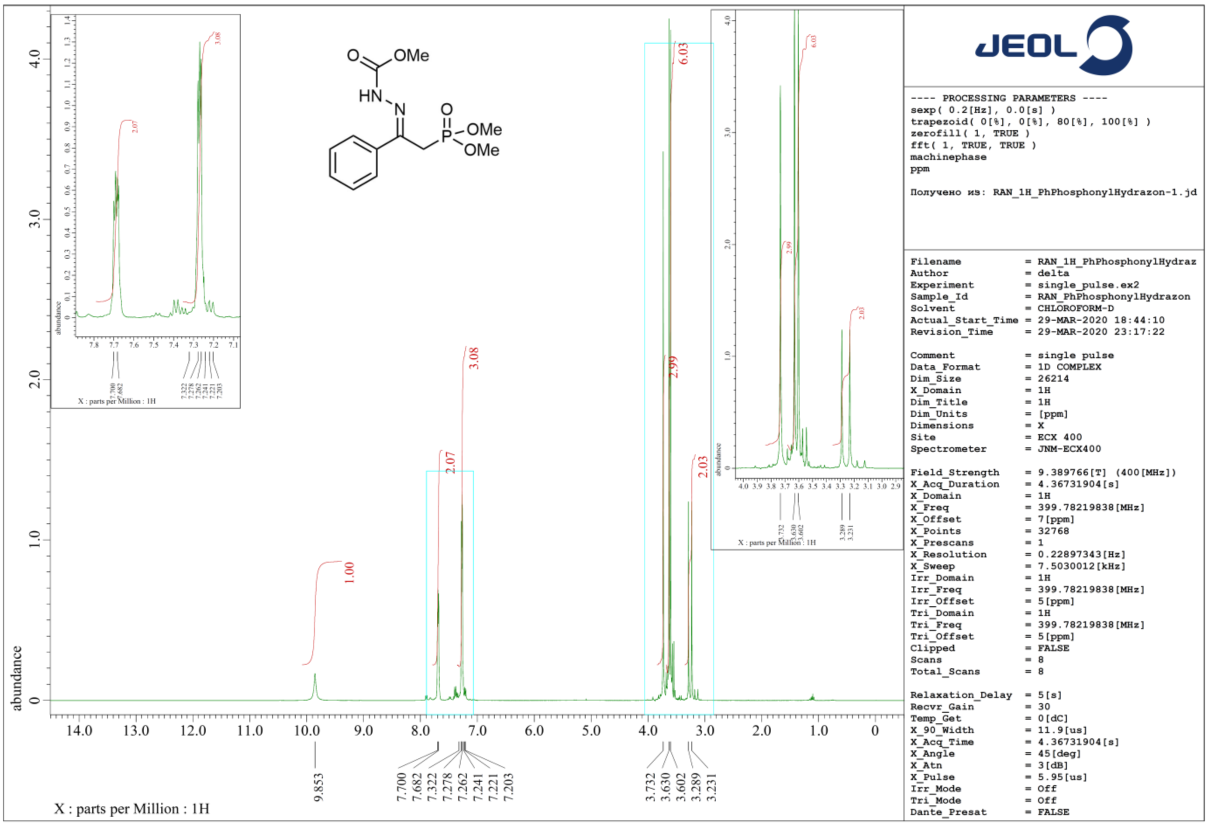

^{13}C NMR spectra of methyl 2-[2-(dimethoxyphosphoryl)-1-phenylethylidene]hydrazine-1-carboxylate **Va** in $CDCl_3$

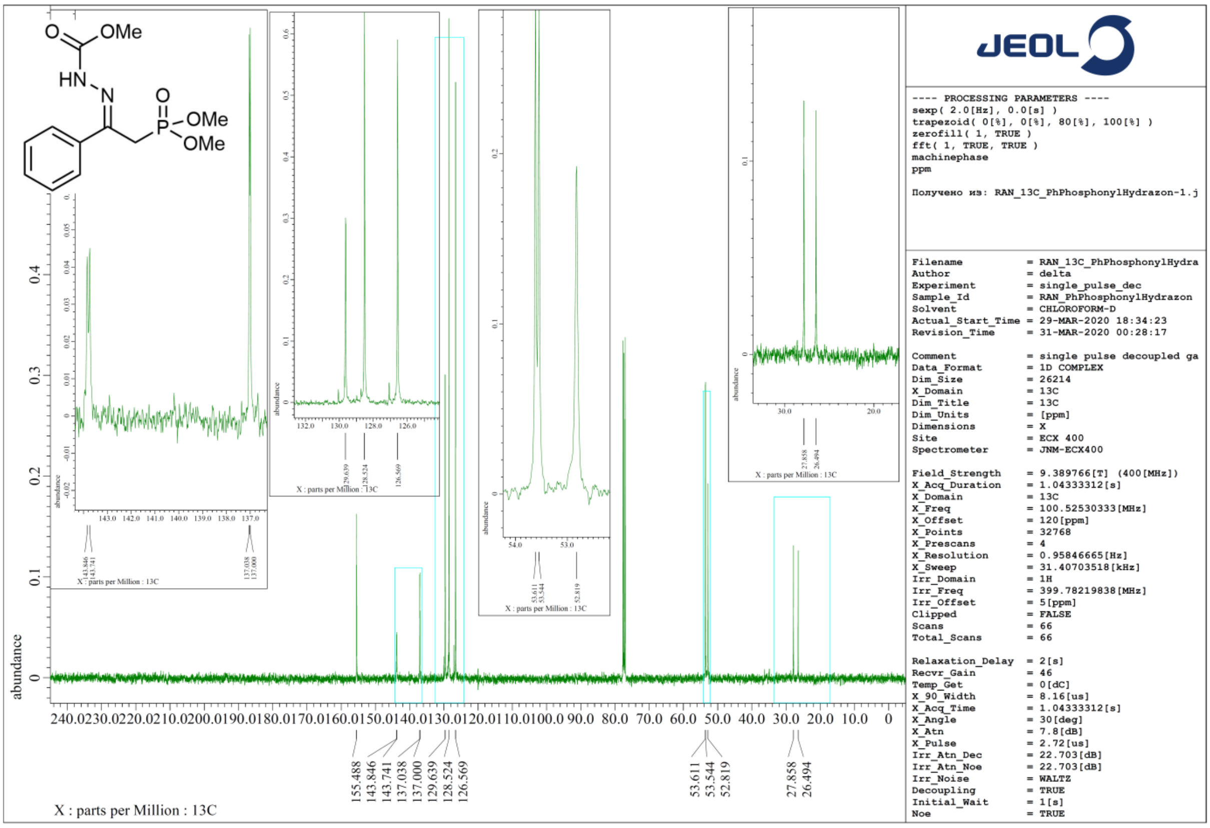

^{31}P NMR spectra of methyl 2-[2-(dimethoxyphosphoryl)-1-phenylethylidene]hydrazine-1-carboxylate **Va** in $CDCl_3$

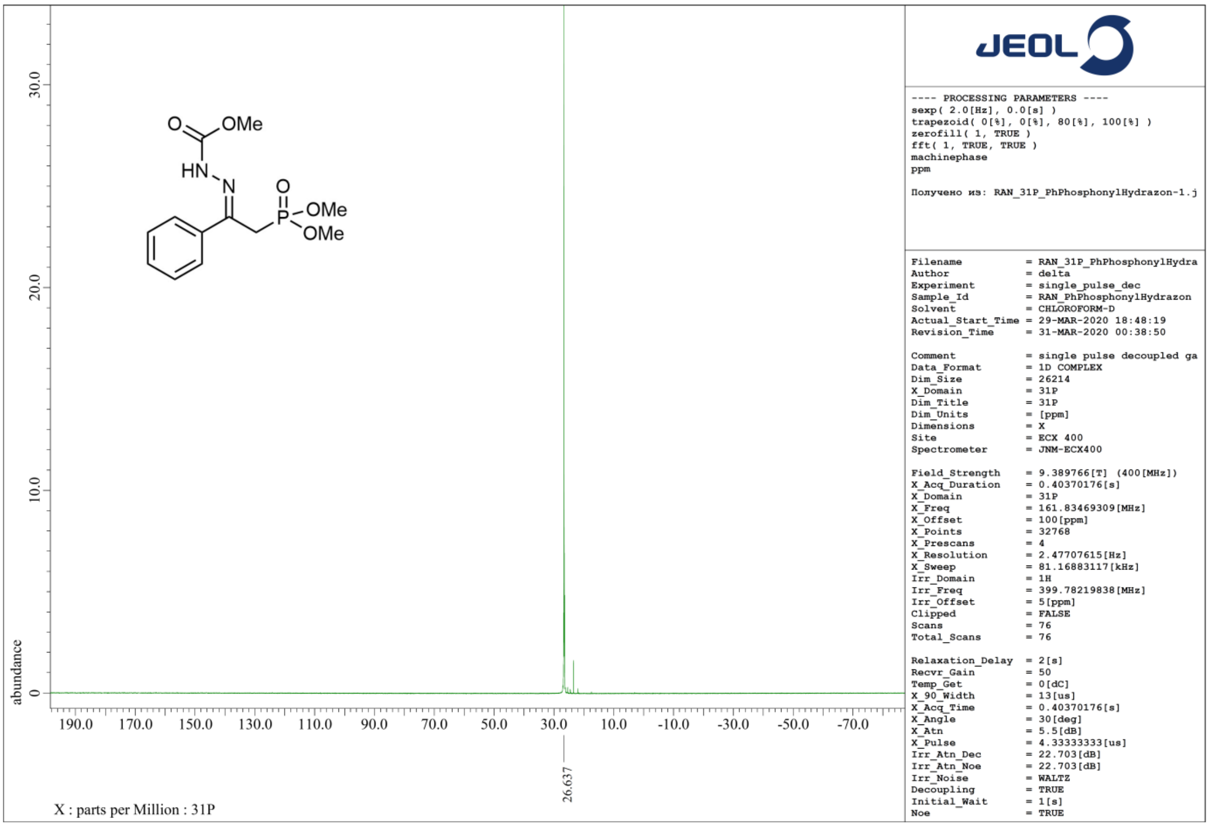

FTIR spectra of methyl 2-(2-(dimethoxyphosphoryl)-1-(4-methoxyphenyl)ethylidene)hydrazine-1-carboxylate **Vb**

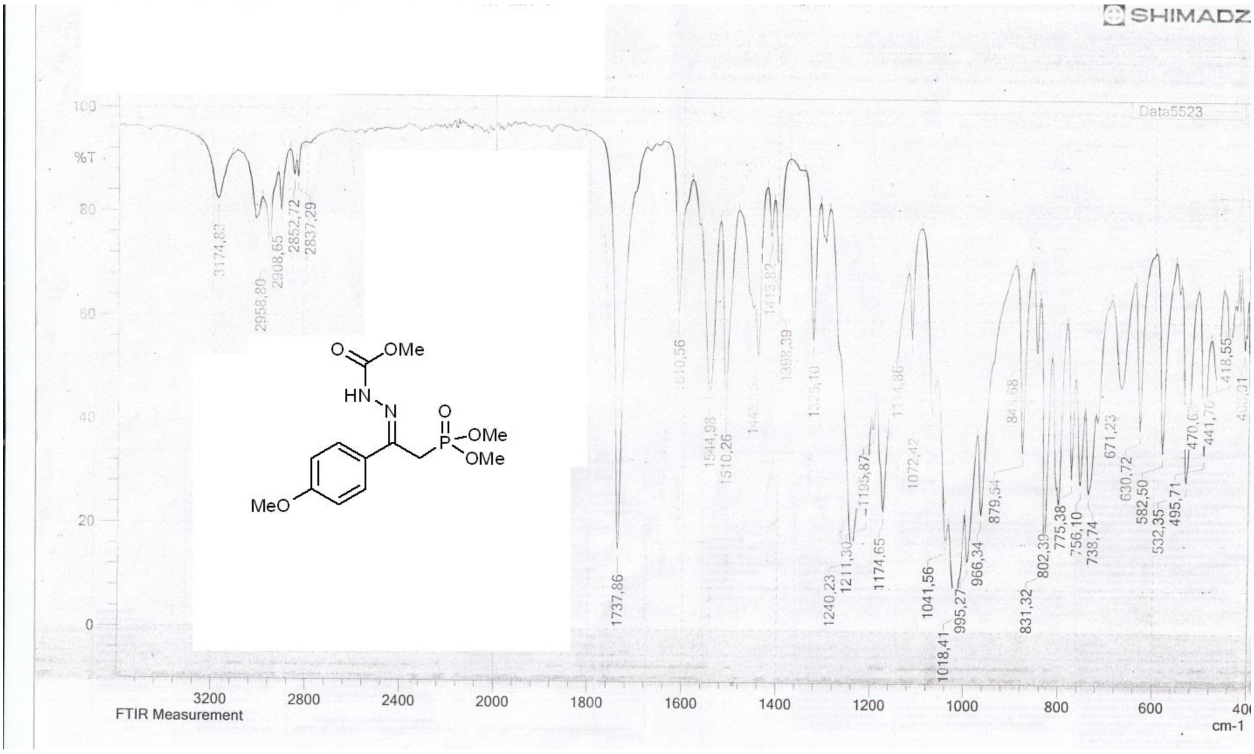

^{1}H NMR spectra of methyl 2-(2-(dimethoxyphosphoryl)-1-(4-methoxyphenyl)ethylidene)hydrazine-1-carboxylate **Vb** in $CDCl_3$

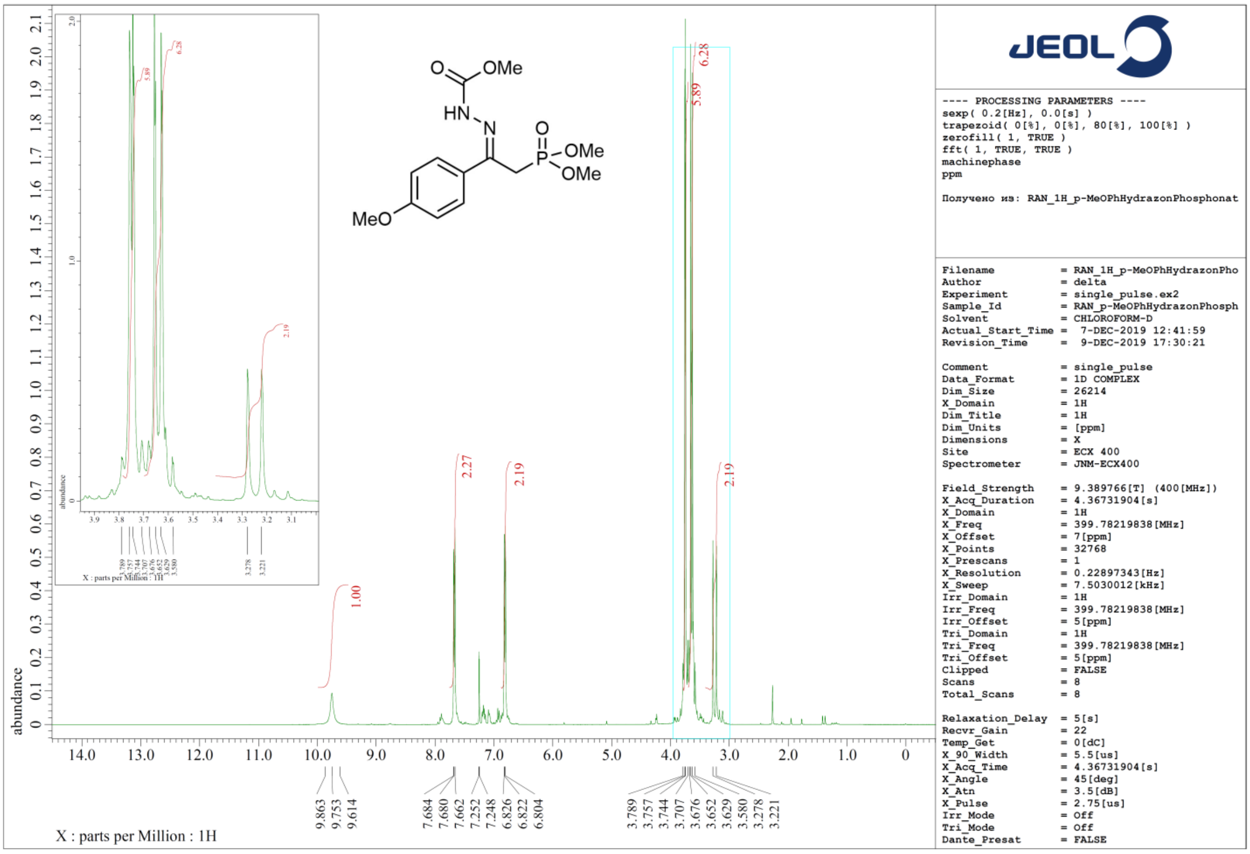

^{13}C NMR spectra of methyl 2-(2-(dimethoxyphosphoryl)-1-(4-methoxyphenyl)ethylidene)hydrazine-1-carboxylate **Vb** in $CDCl_3$

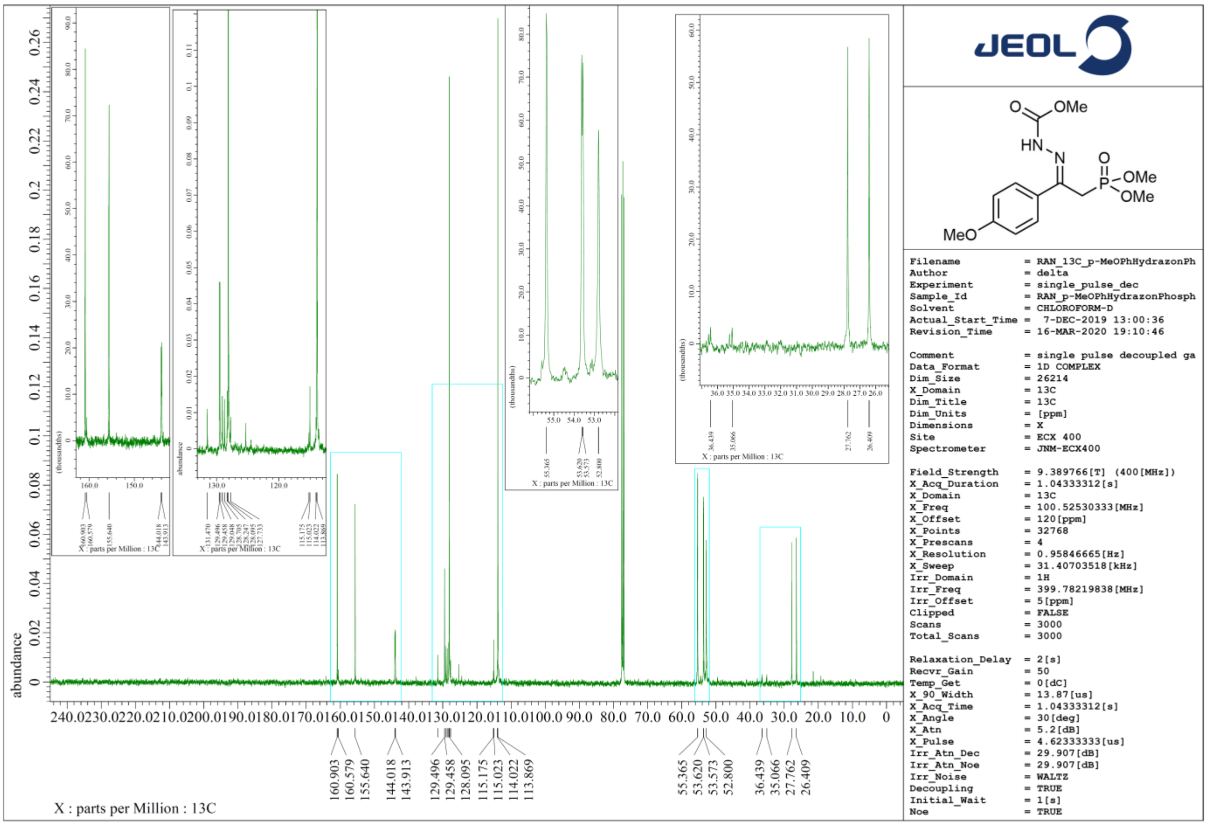

DEPT NMR spectra of methyl 2-(2-(dimethoxyphosphoryl)-1-(4-methoxyphenyl)ethylidene)hydrazine-1-carboxylate **Vb** in $CDCl_3$

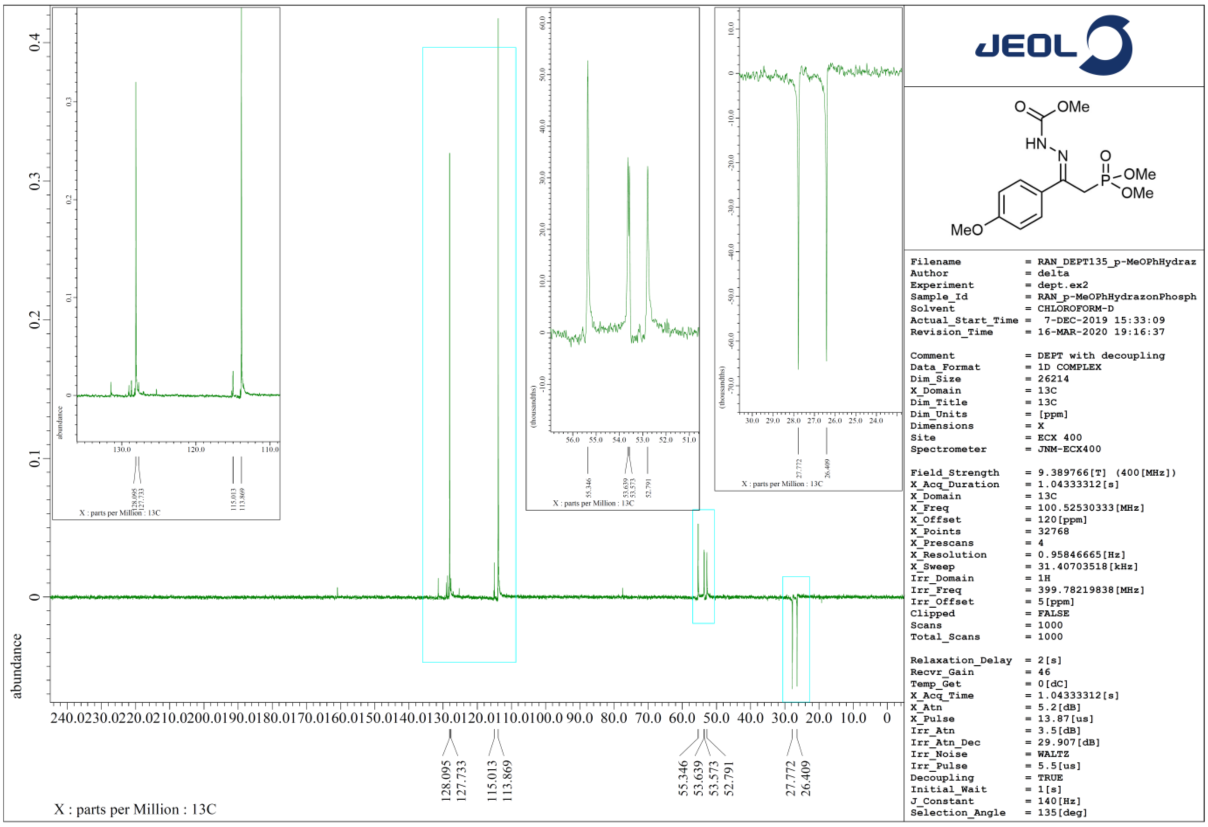

^{31}P NMR spectra of methyl 2-(2-(dimethoxyphosphoryl)-1-(4-methoxyphenyl)ethylidene)hydrazine-1-carboxylate **Vb** in $CDCl_3$

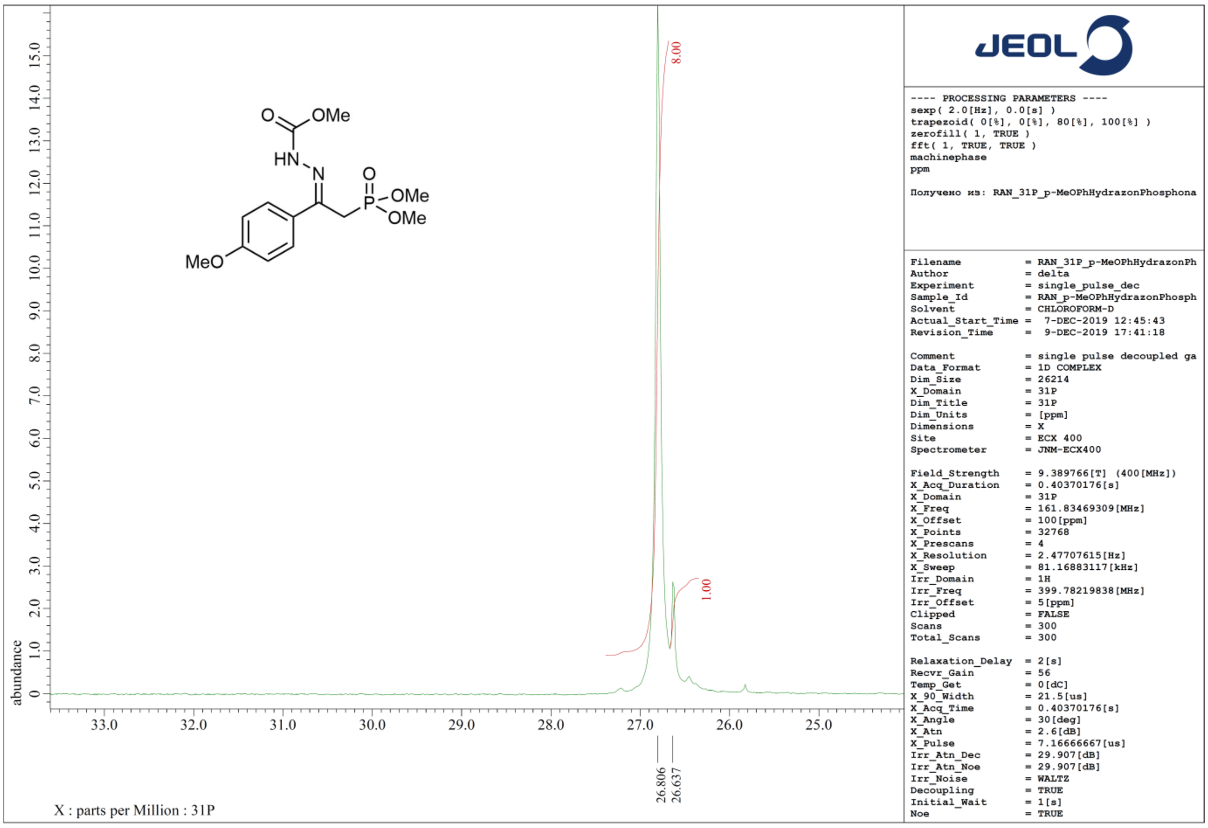

FTIR spectra of methyl 2-(2-(dimethoxyphosphoryl)-1-(3-methoxyphenyl)ethylidene)hydrazine-1-carboxylate **Vc**

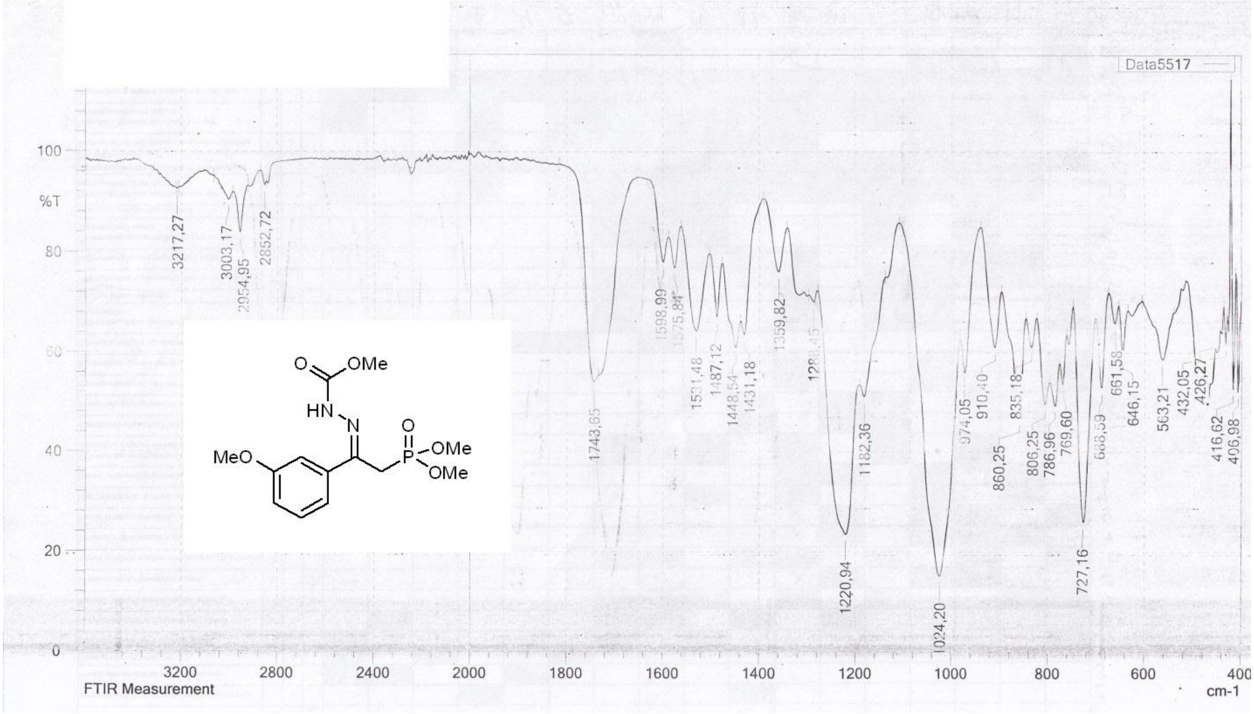

^{1}H NMR spectra of methyl 2-(2-(dimethoxyphosphoryl)-1-(3-methoxyphenyl)ethylidene)hydrazine-1-carboxylate **Vc** in $CDCl_3$

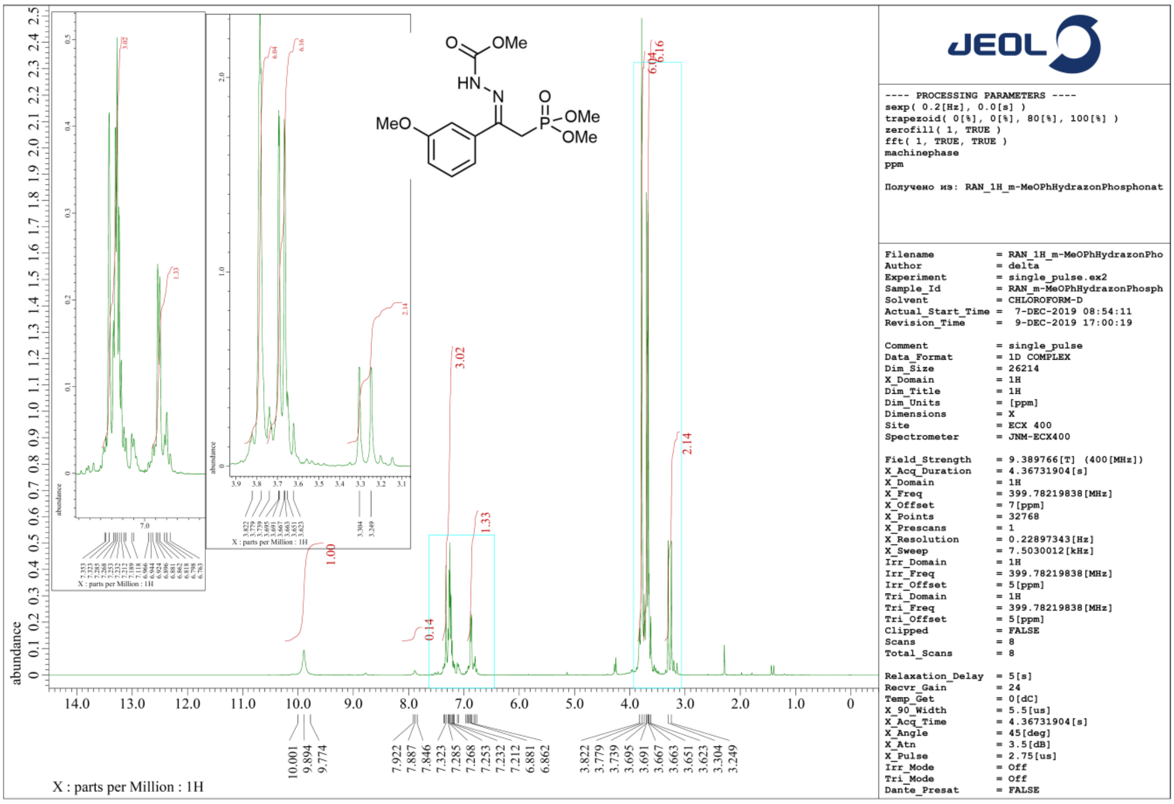

^{13}C NMR spectra of methyl 2-(2-(dimethoxyphosphoryl)-1-(3-methoxyphenyl)ethylidene)hydrazine-1-carboxylate **Vc** in $CDCl_3$

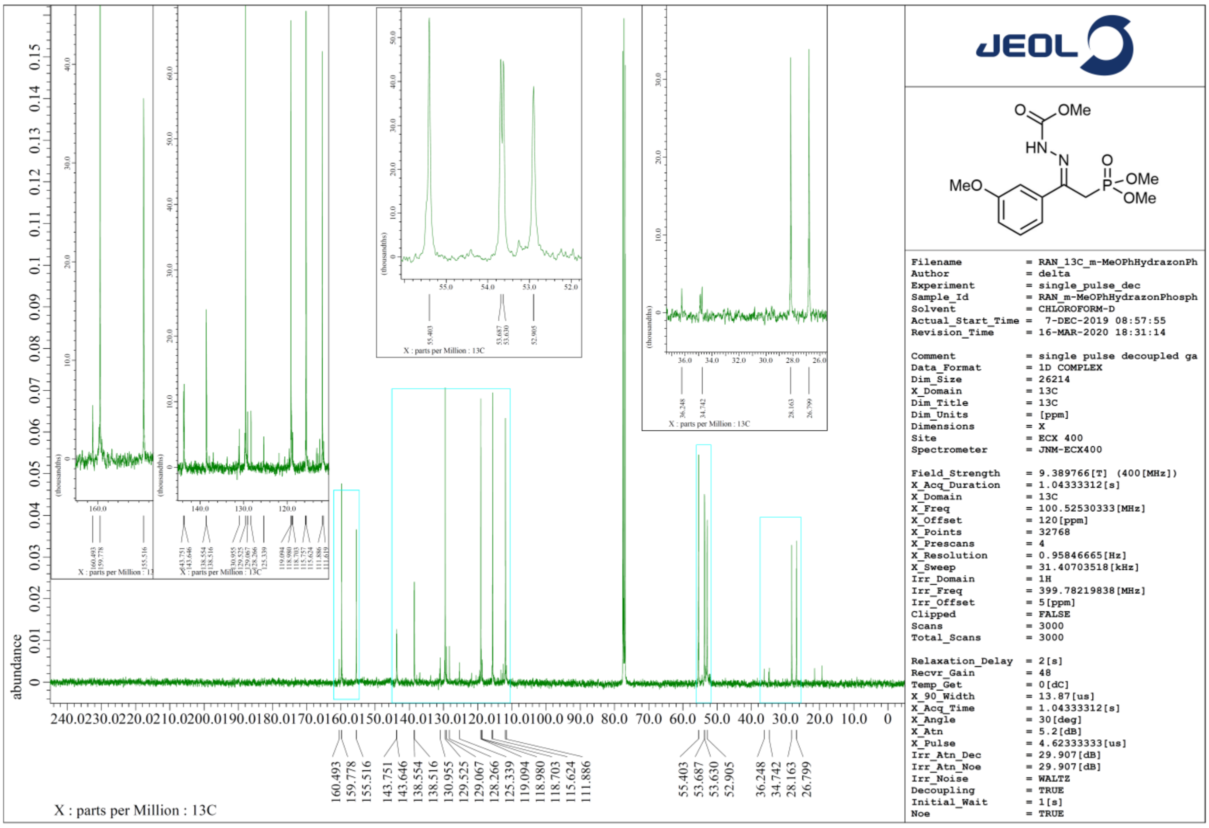

DEPT NMR spectra of methyl 2-(2-(dimethoxyphosphoryl)-1-(3-methoxyphenyl)ethylidene)hydrazine-1-carboxylate **Vc** in $CDCl_3$

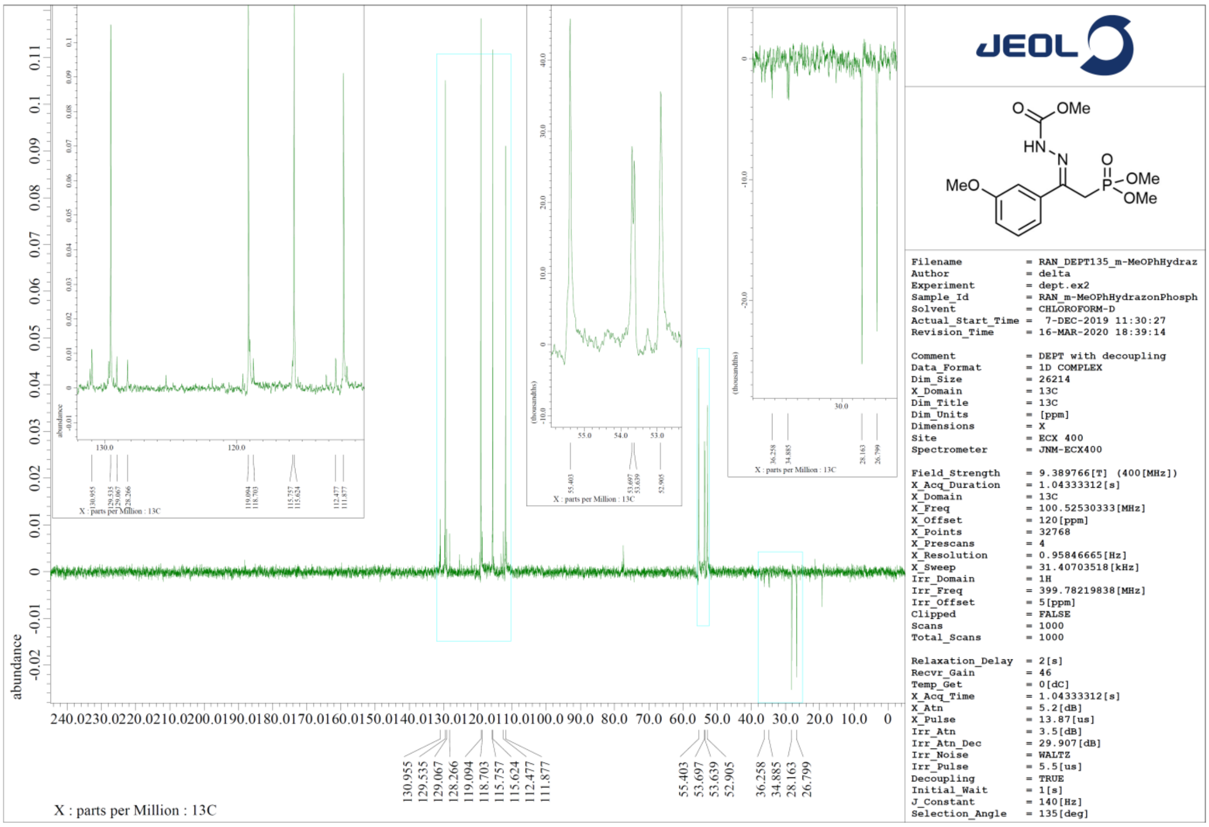

^{31}P NMR spectra of methyl 2-(2-(dimethoxyphosphoryl)-1-(3-methoxyphenyl)ethylidene)hydrazine-1-carboxylate **Vc** in $CDCl_3$

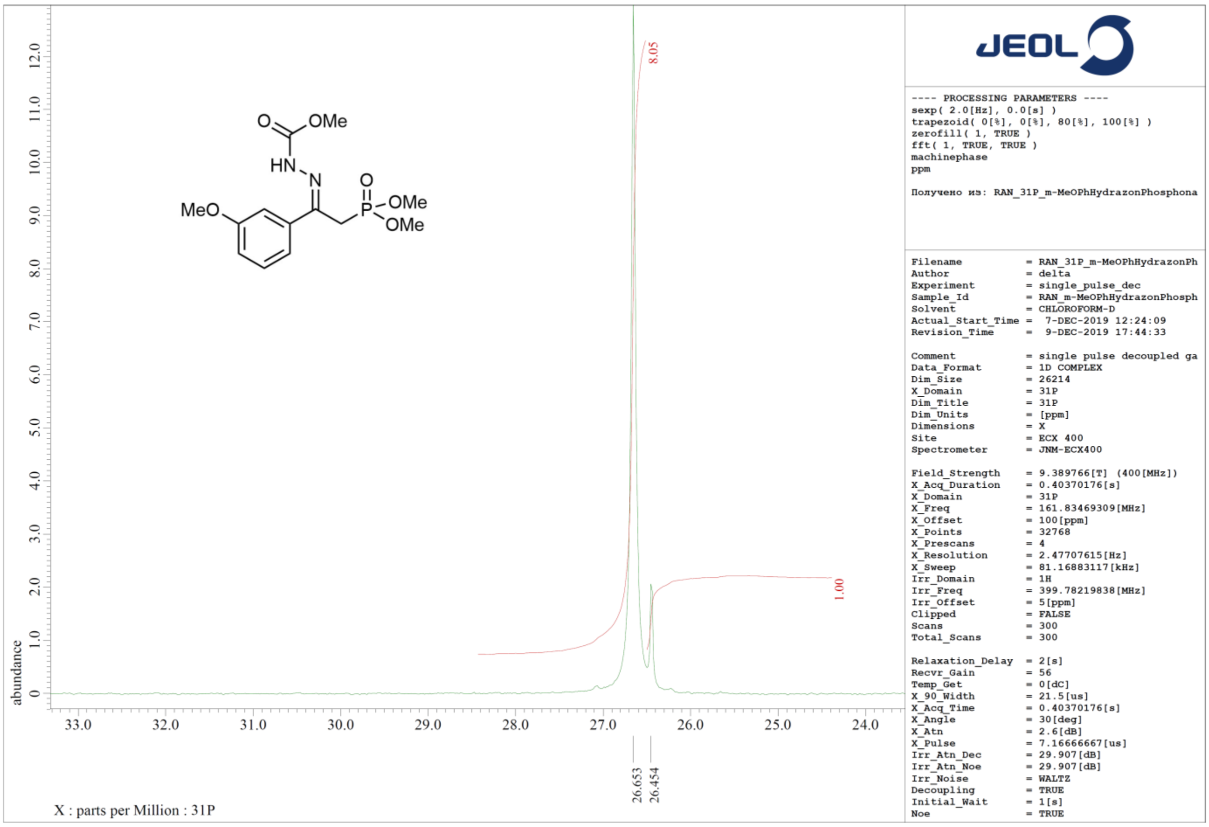

FTIR spectra of methyl 2-(2-(dimethoxyphosphoryl)-1-(2-methoxyphenyl)ethylidene)hydrazine-1-carboxylate **Vd**

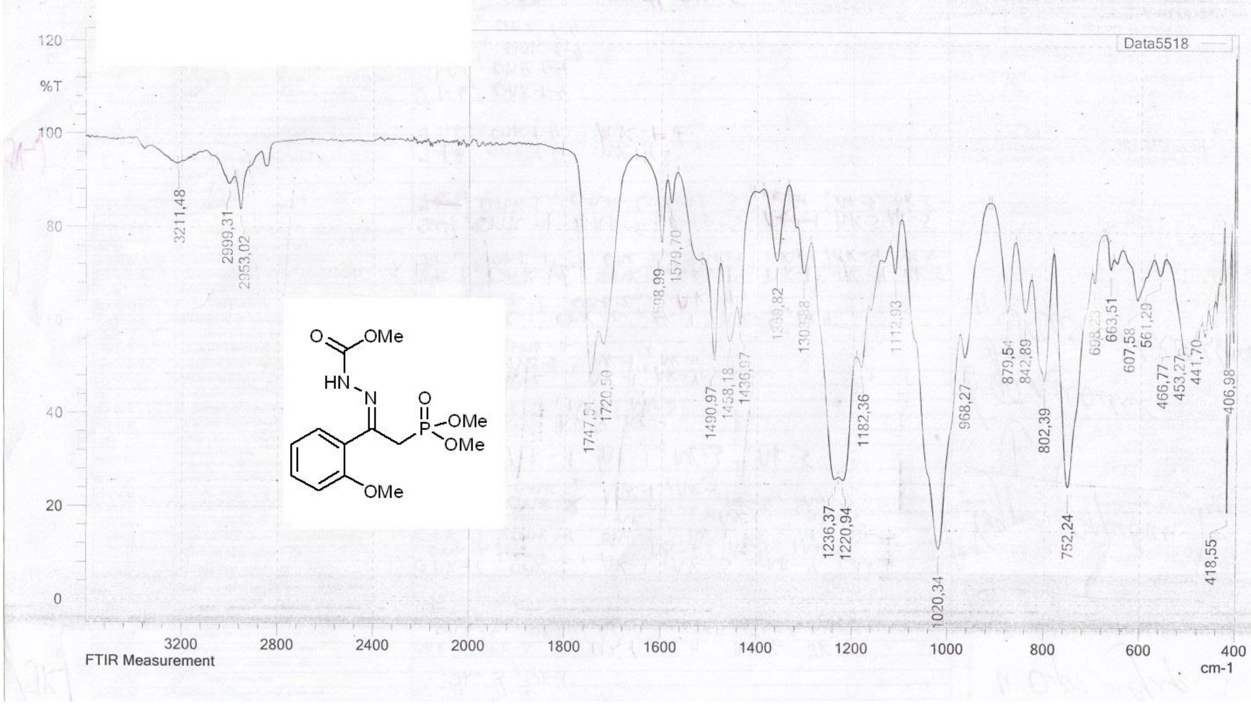

^{1}H NMR spectra of methyl 2-(2-(dimethoxyphosphoryl)-1-(2-methoxyphenyl)ethylidene)hydrazine-1-carboxylate **Vd** in $CDCl_3$

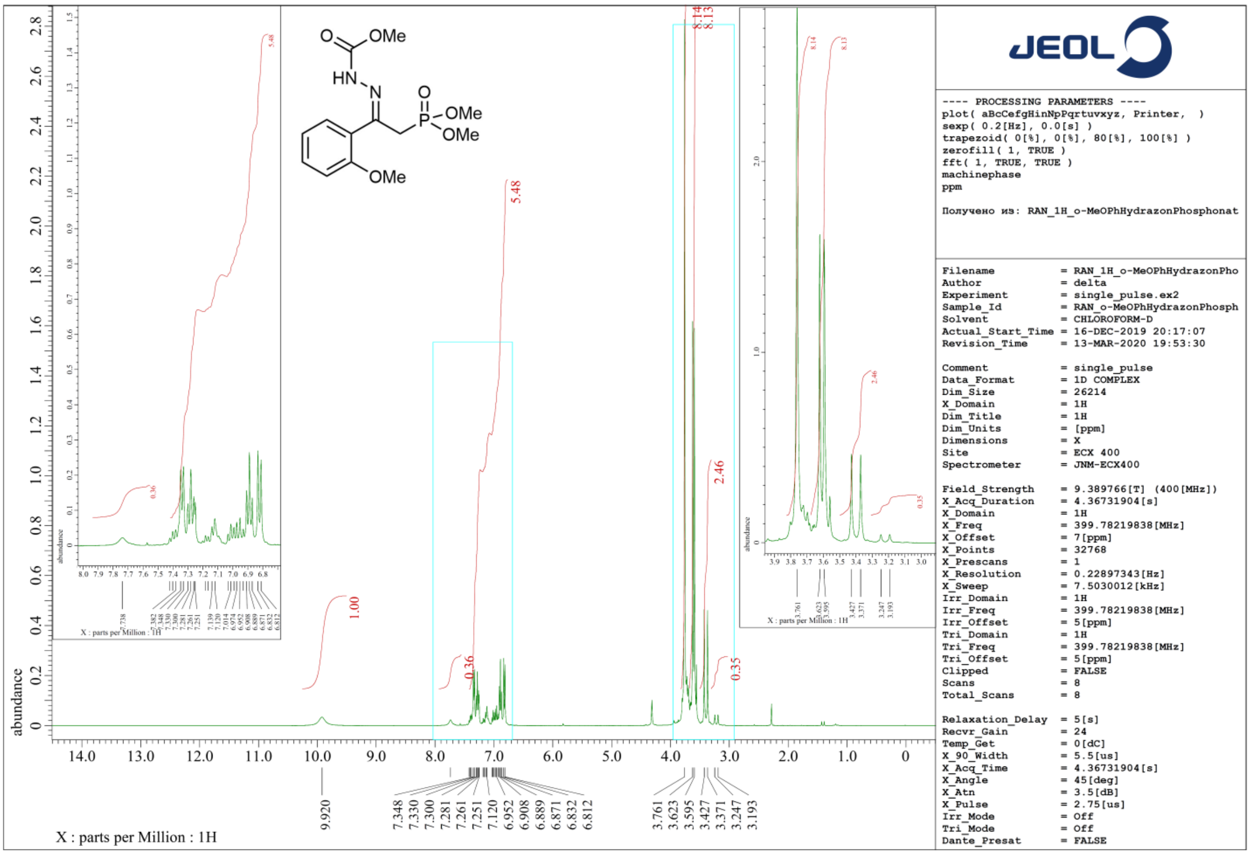

^{13}C NMR spectra of methyl 2-(2-(dimethoxyphosphoryl)-1-(2-methoxyphenyl)ethylidene)hydrazine-1-carboxylate **Vd** in $CDCl_3$

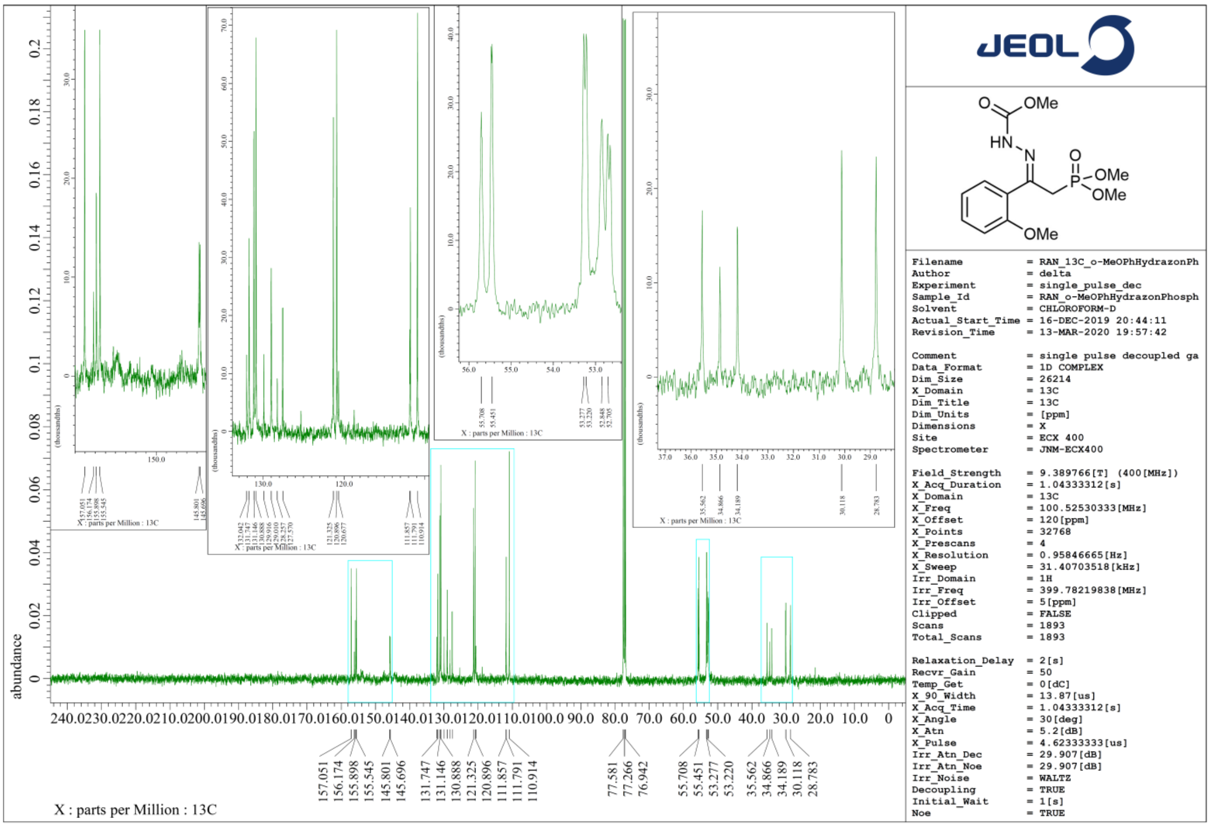

DEPT NMR spectra of methyl 2-(2-(dimethoxyphosphoryl)-1-(2-methoxyphenyl)ethylidene)hydrazine-1-carboxylate **Vd** in $CDCl_3$

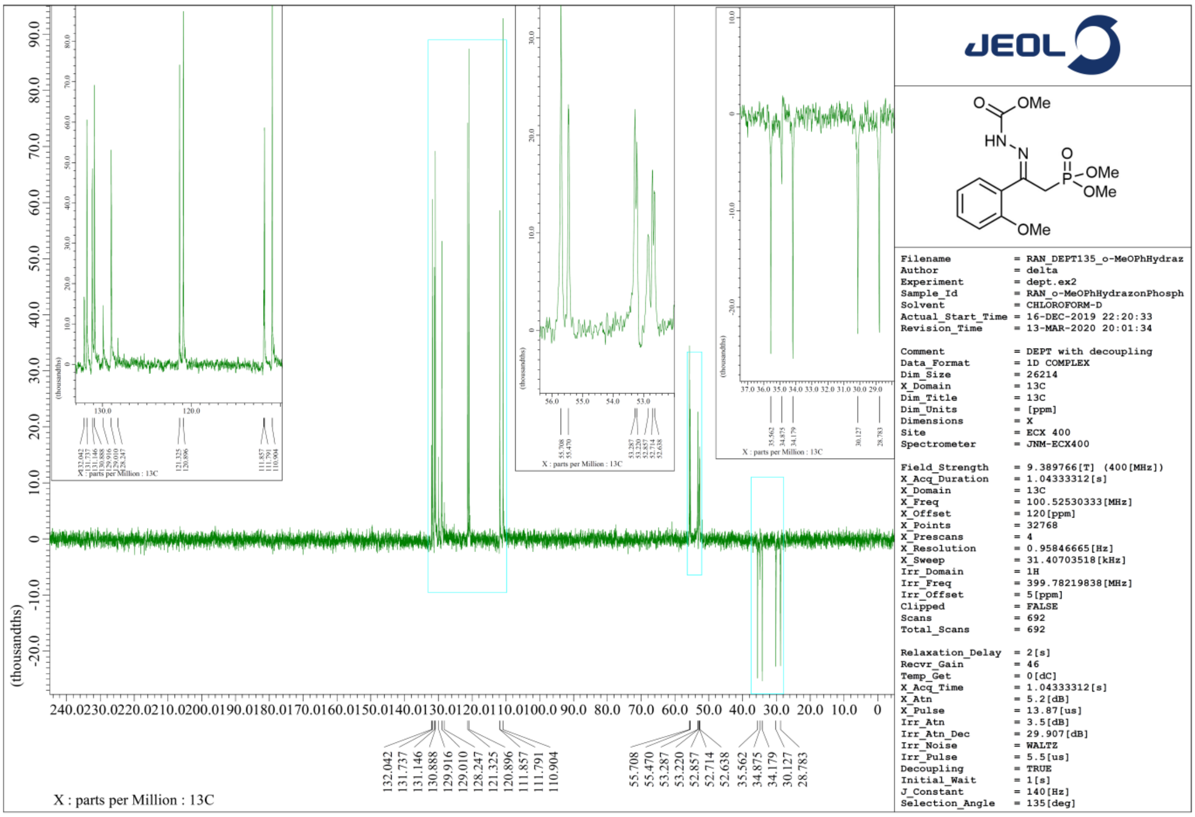

^{31}P NMR spectra of methyl 2-(2-(dimethoxyphosphoryl)-1-(2-methoxyphenyl)ethylidene)hydrazine-1-carboxylate **Vd** in $CDCl_3$

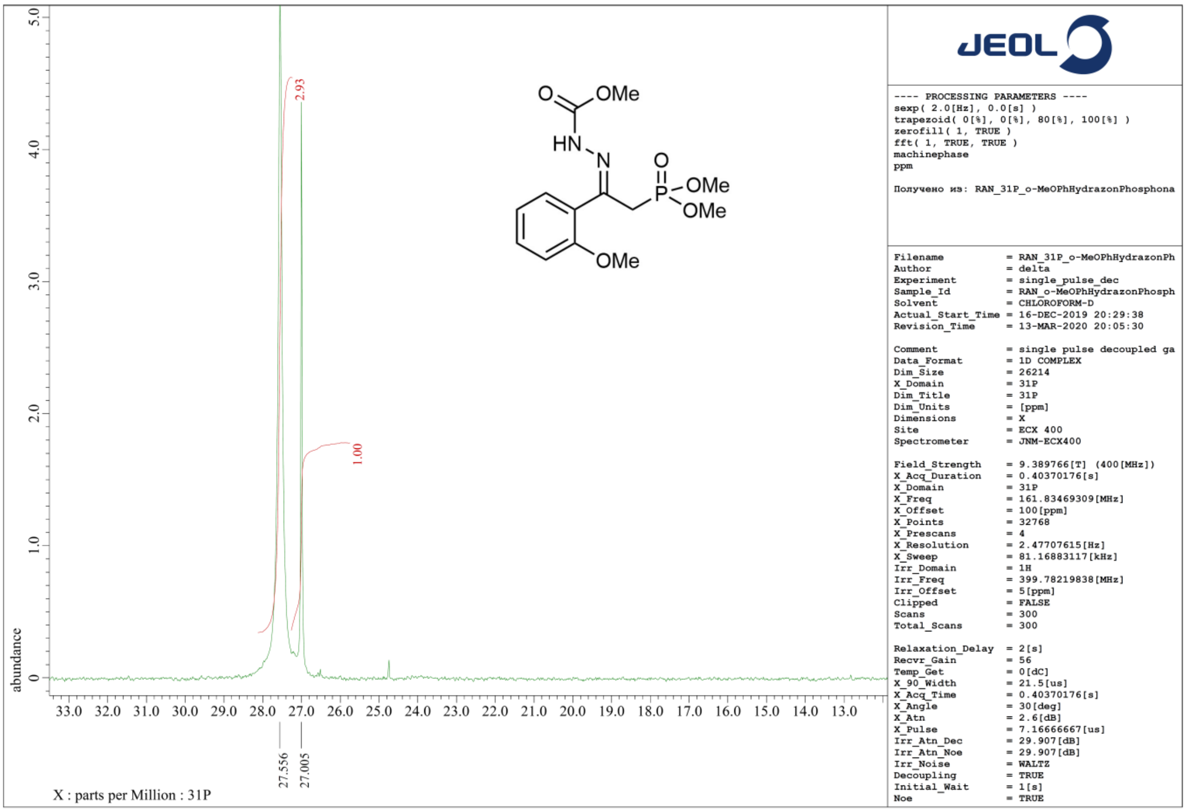

FTIR spectra of methyl 2-(1-(adamantan-1-yl)-2-(dimethoxyphosphoryl)ethylidene)hydrazine-1-carboxylate

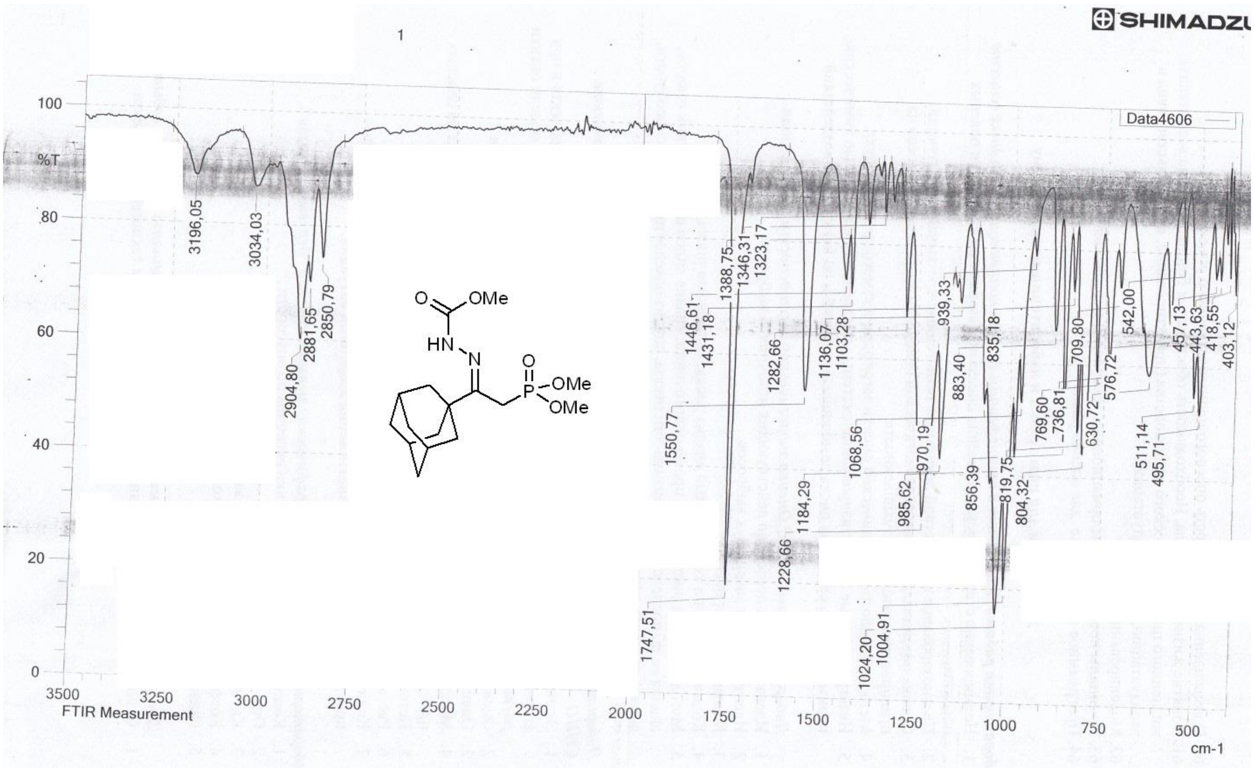

^{1}H NMR spectra of methyl 2-(1-(adamantan-1-yl)-2-(dimethoxyphosphoryl)ethylidene)hydrazine-1-carboxylate in $CDCl_3$

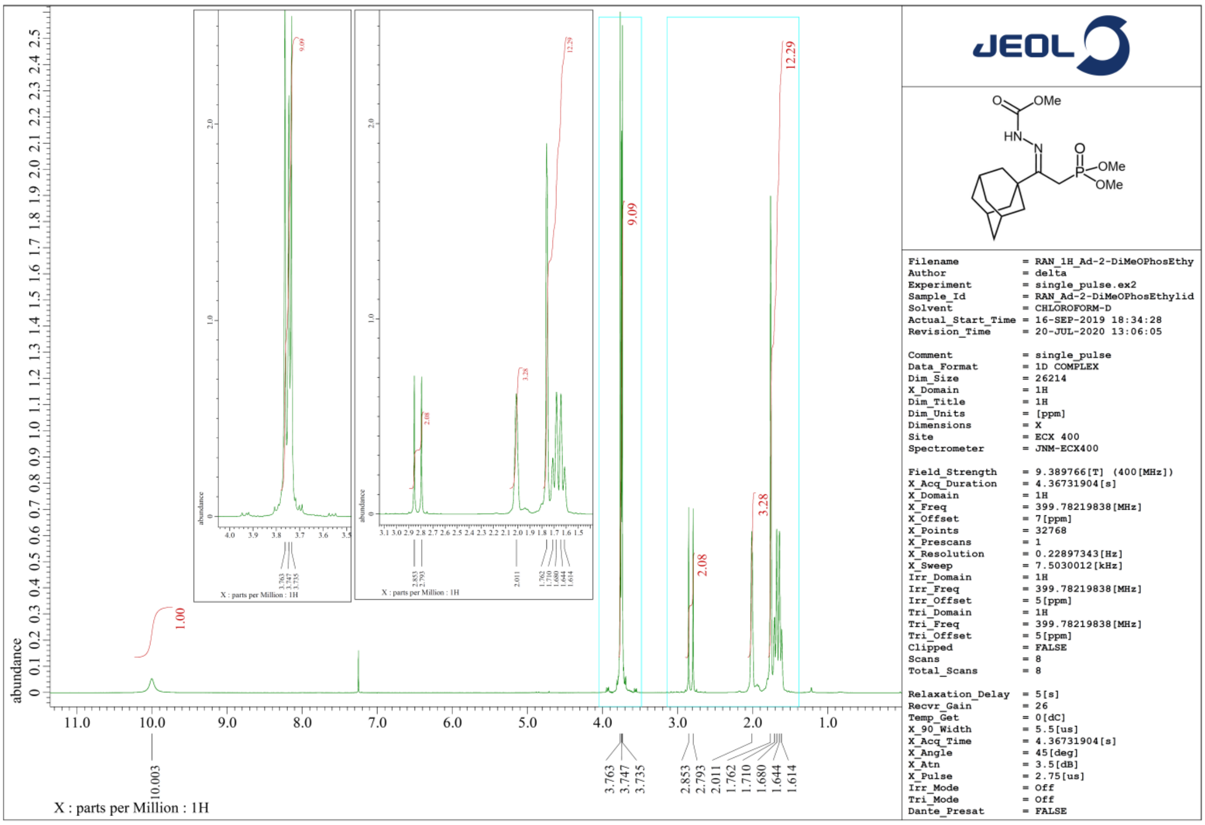

^{13}C NMR spectra of methyl 2-(1-(adamantan-1-yl)-2-(dimethoxyphosphoryl)ethylidene)hydrazine-1-carboxylate in $CDCl_3$

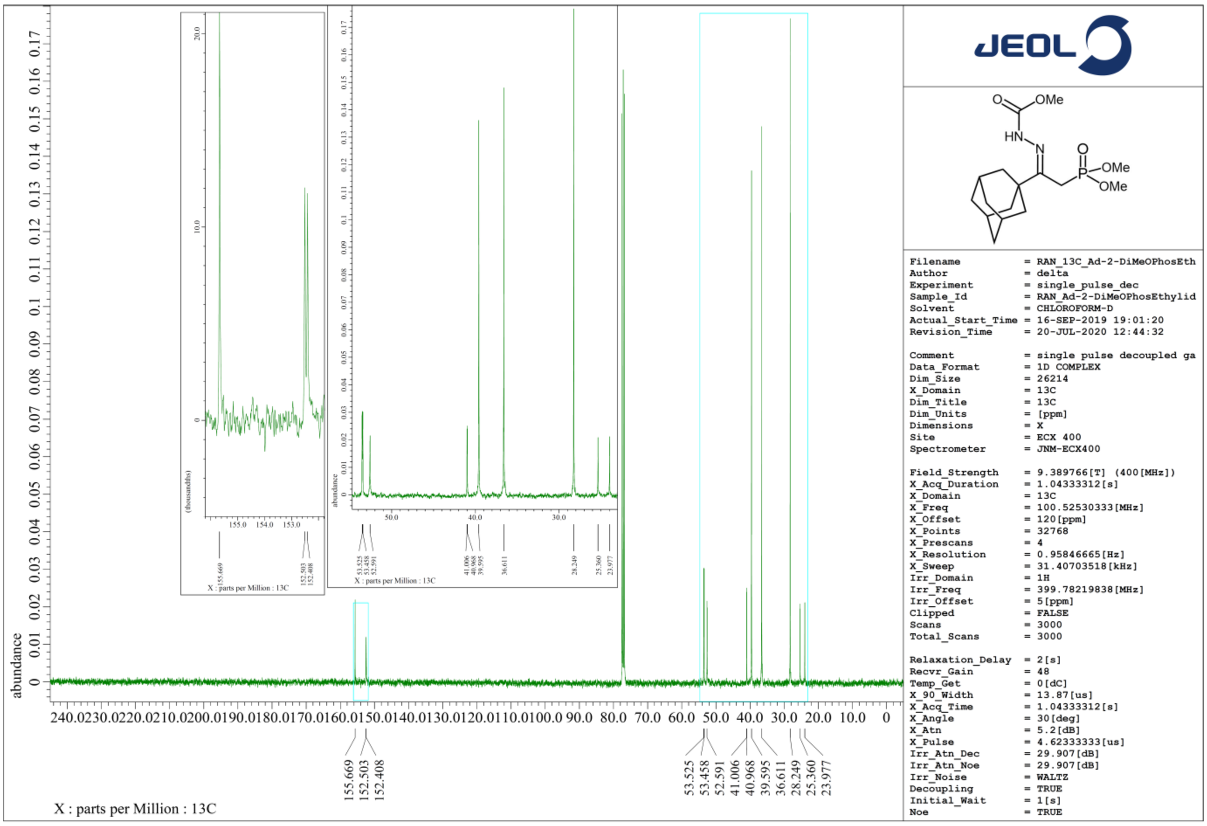

DEPT NMR spectra of methyl 2-(1-(adamantan-1-yl)-2-(dimethoxyphosphoryl)ethylidene)hydrazine-1-carboxylate in $CDCl_3$

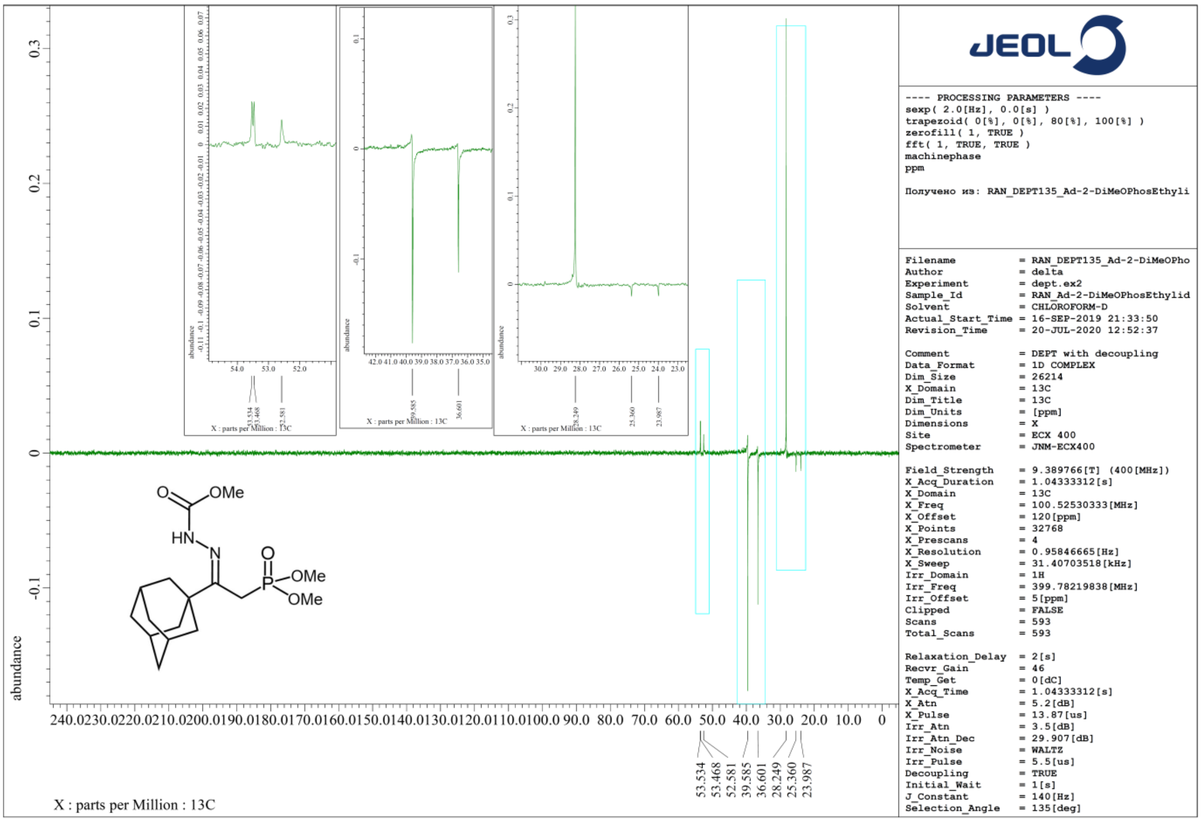

^{31}P NMR spectra of methyl 2-(1-(adamantan-1-yl)-2-(dimethoxyphosphoryl)ethylidene)hydrazine-1-carboxylate in $CDCl_3$

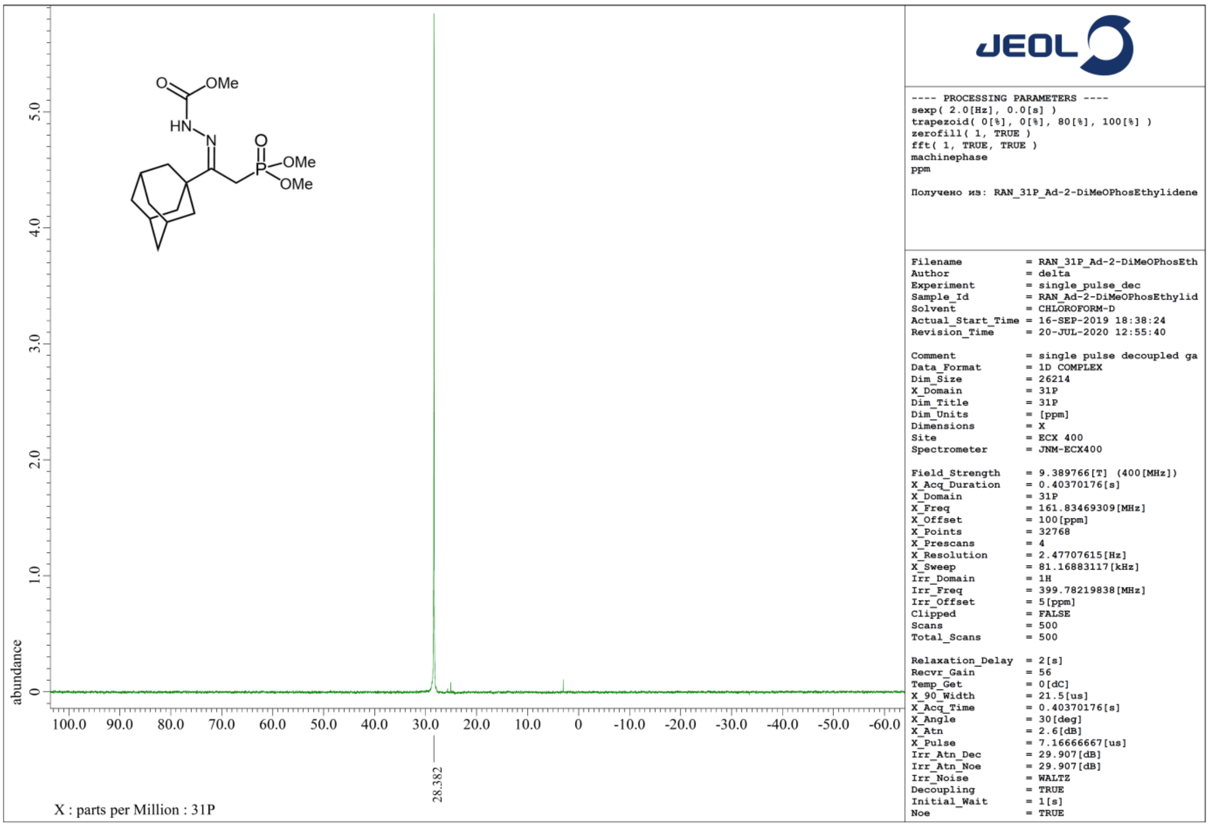

^{1}H NMR spectra of dimethyl (2-oxo-2-phenylethyl)phosphonate **VIa** in $CDCl_3$

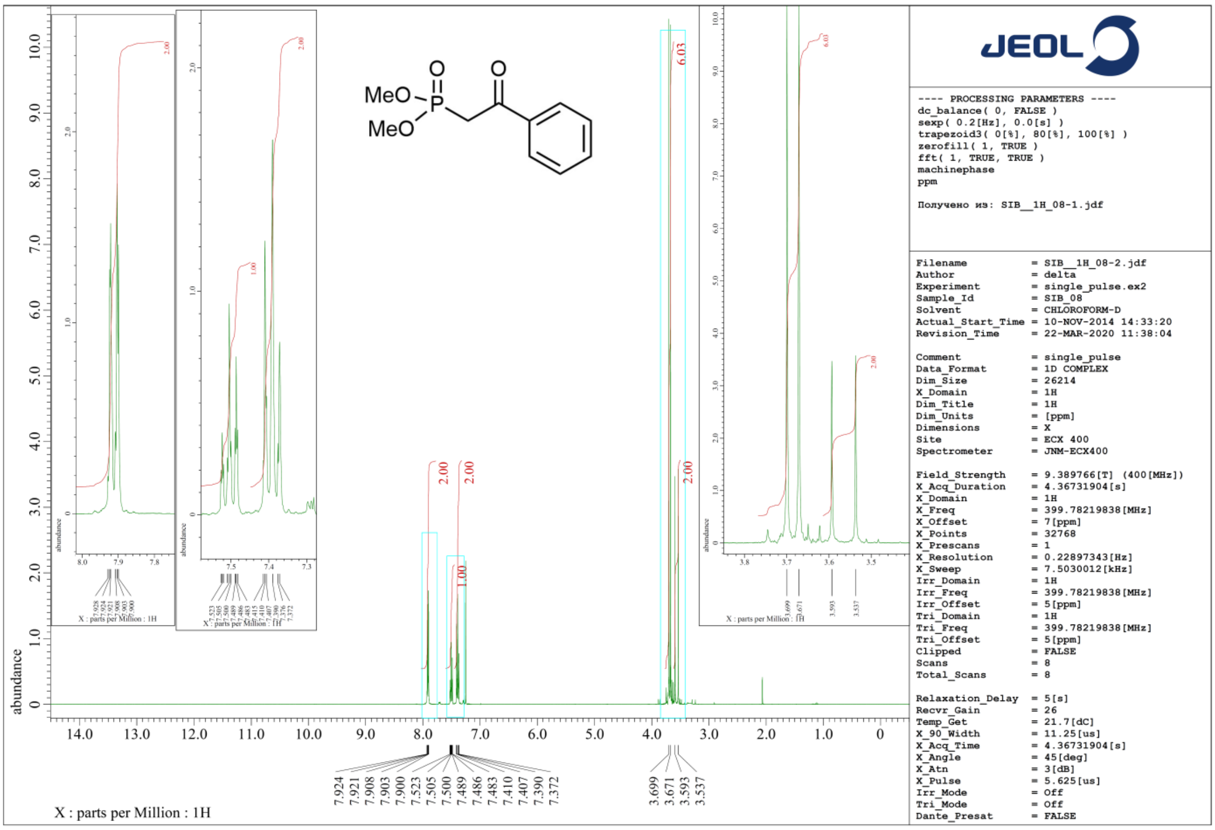

^{13}C NMR spectra of dimethyl (2-oxo-2-phenylethyl)phosphonate **VIa** in $CDCl_3$

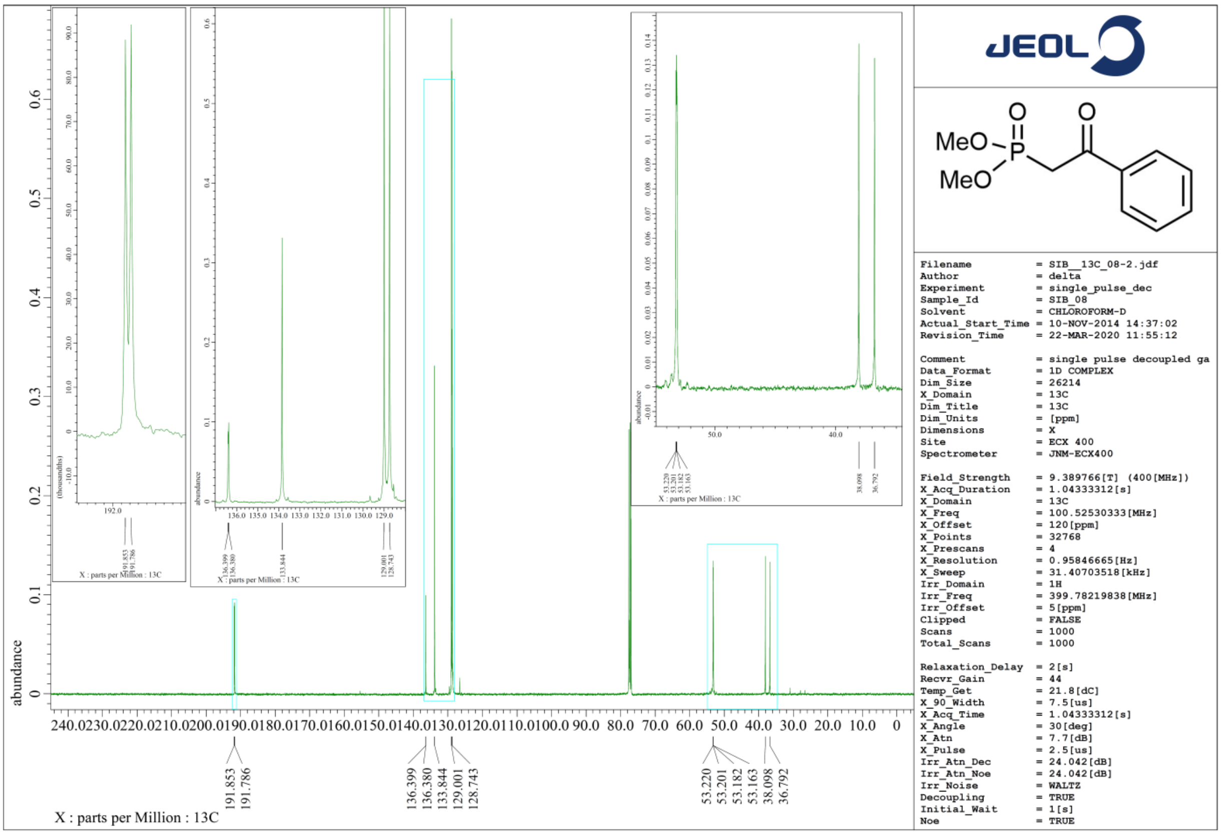

^{31}P NMR spectra of dimethyl (2-oxo-2-phenylethyl)phosphonate **VIa** in $CDCl_3$

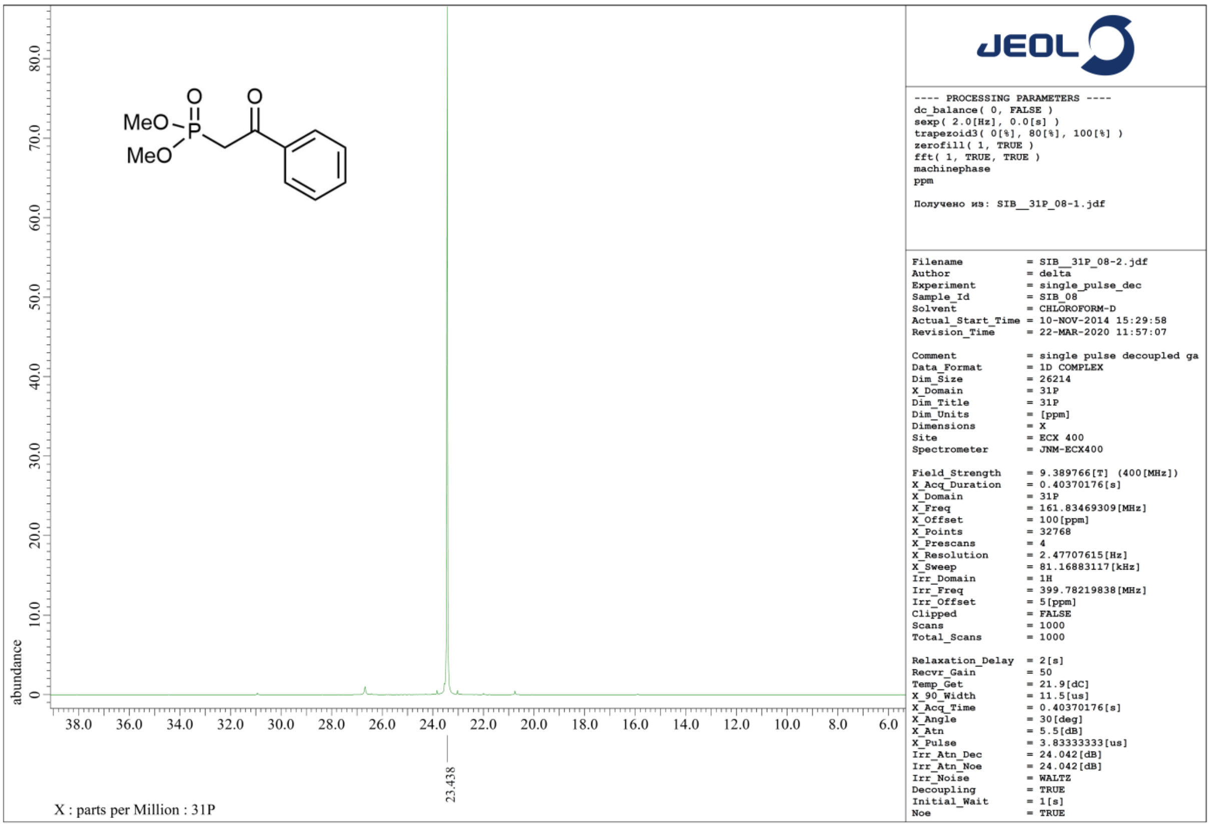

FTIR spectra of dimethyl [2-(4-methoxyphenyl)-2-oxoethyl]phosphonate **VIb**

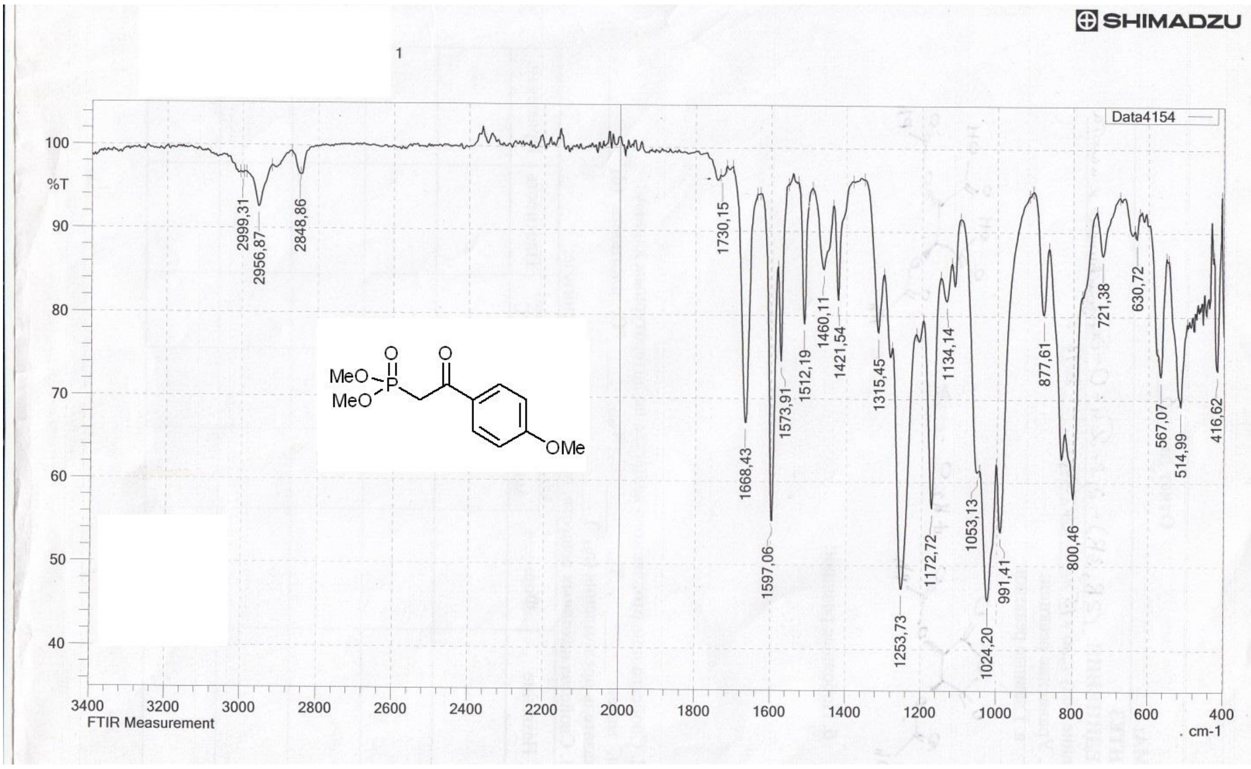

^{1}H NMR spectra of dimethyl [2-(4-methoxyphenyl)-2-oxoethyl]phosphonate **VIb** in $CDCl_3$

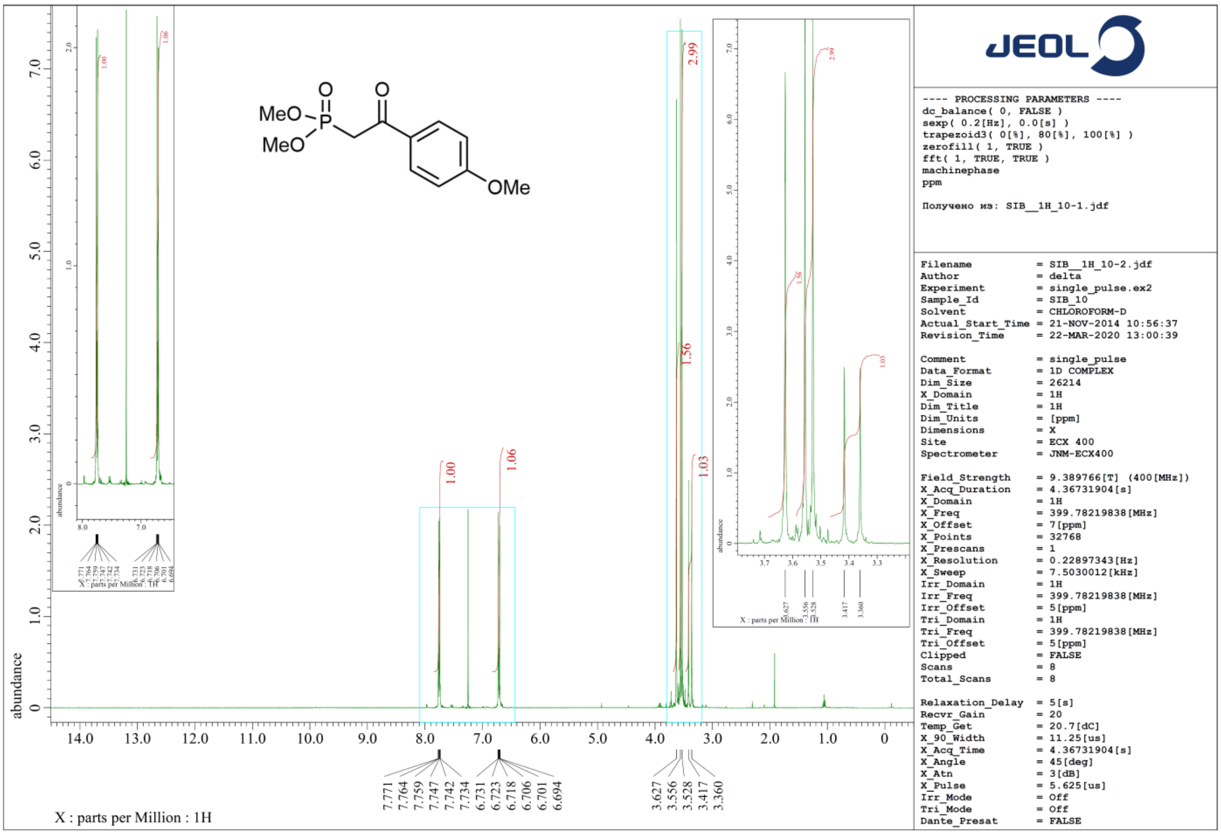

^{13}C NMR spectra of dimethyl [2-(4-methoxyphenyl)-2-oxoethyl]phosphonate **VIb** in $CDCl_3$

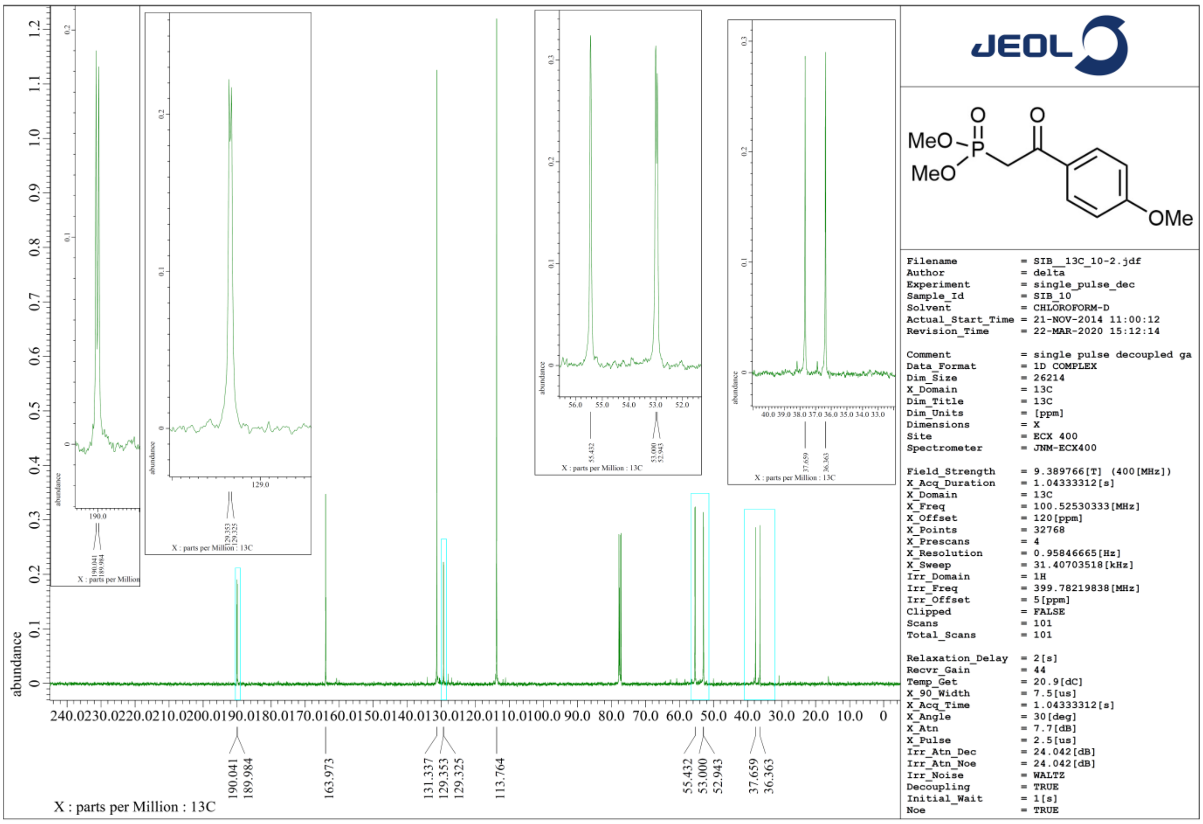

^{31}P NMR spectra of dimethyl [2-(4-methoxyphenyl)-2-oxoethyl]phosphonate **VIb** in $CDCl_3$

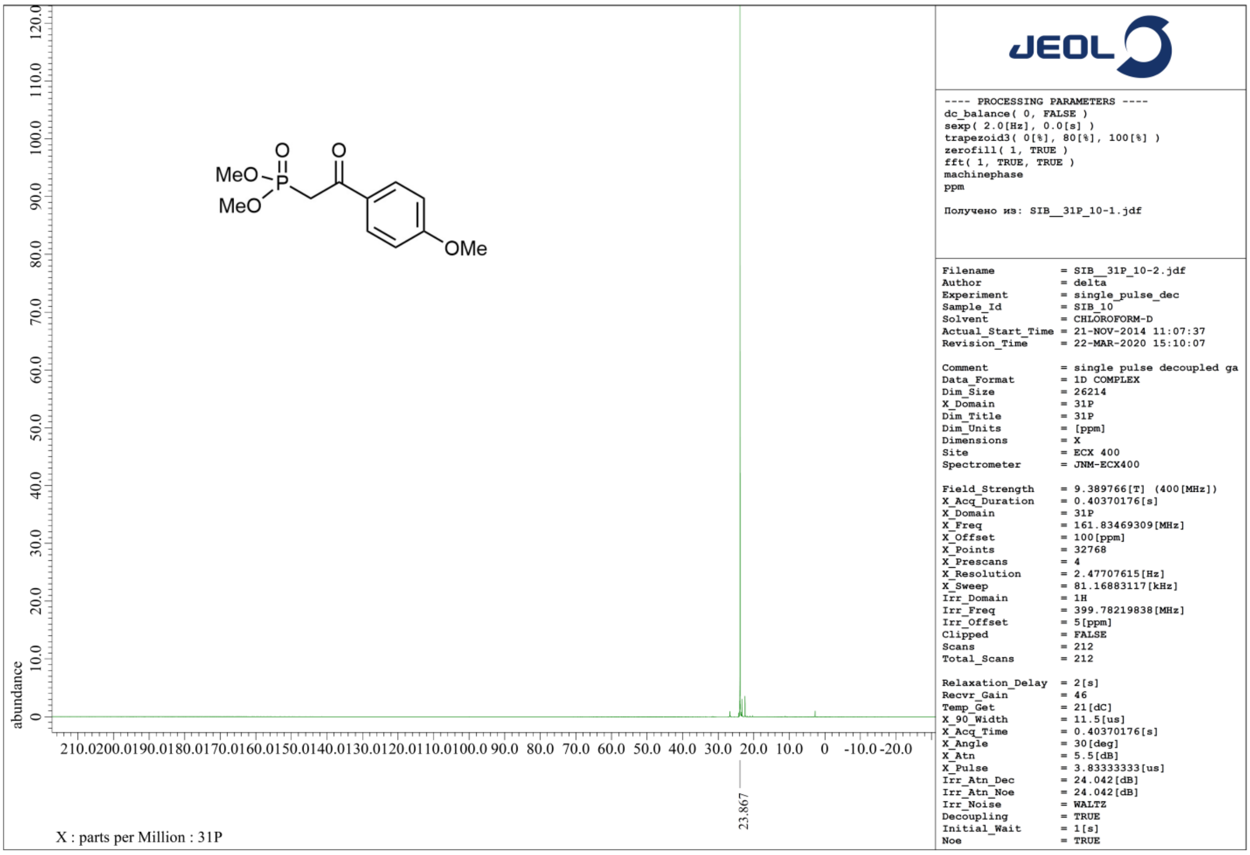

FTIR spectra of dimethyl [2-(3-methoxyphenyl)-2-oxoethyl]phosphonate **VIc**

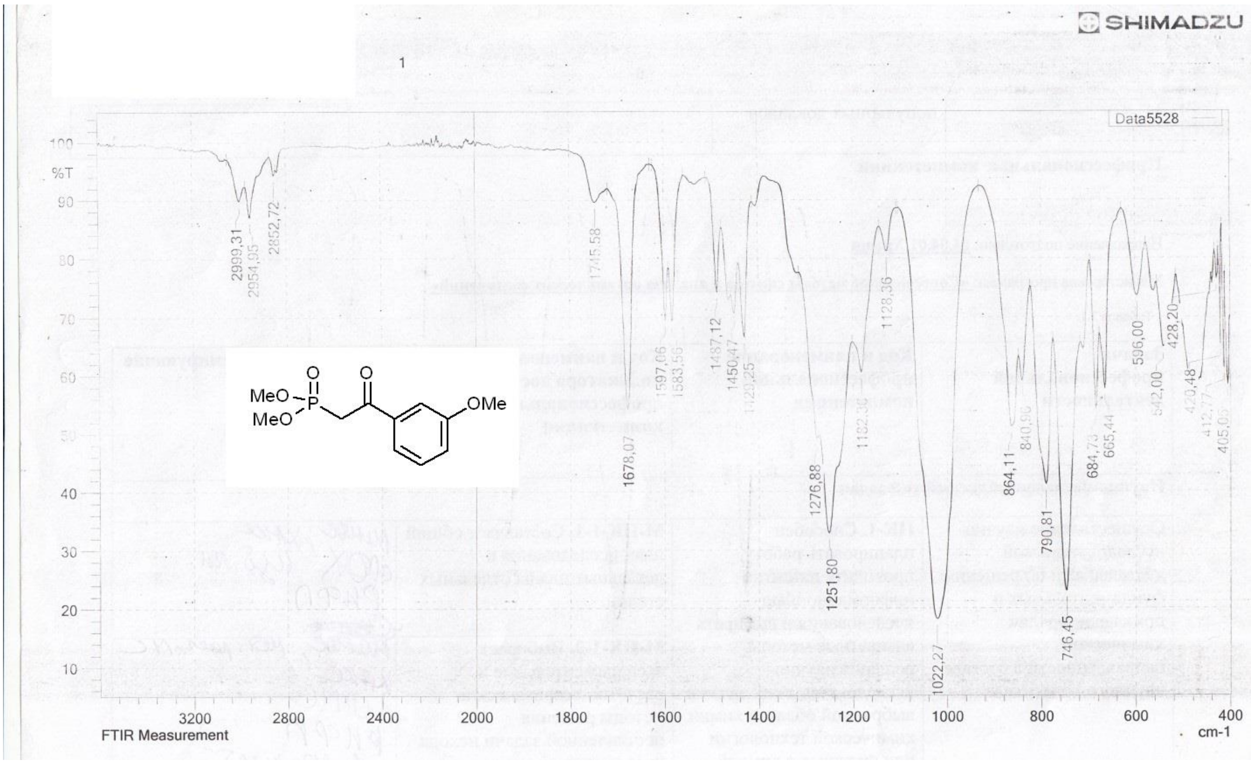

^{1}H NMR spectra of dimethyl [2-(3-methoxyphenyl)-2-oxoethyl]phosphonate **VIc** in $CDCl_3$

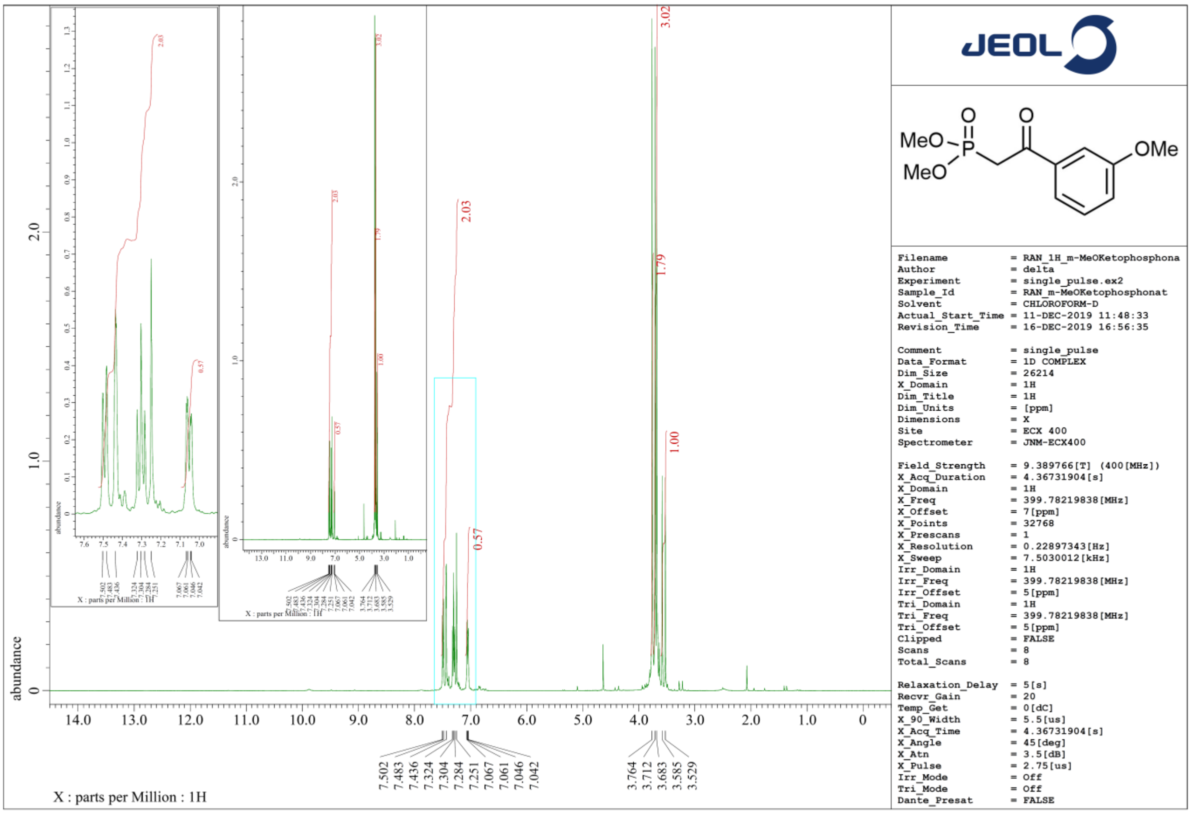

^{13}C NMR spectra of dimethyl [2-(3-methoxyphenyl)-2-oxoethyl]phosphonate **VIc** in $CDCl_3$

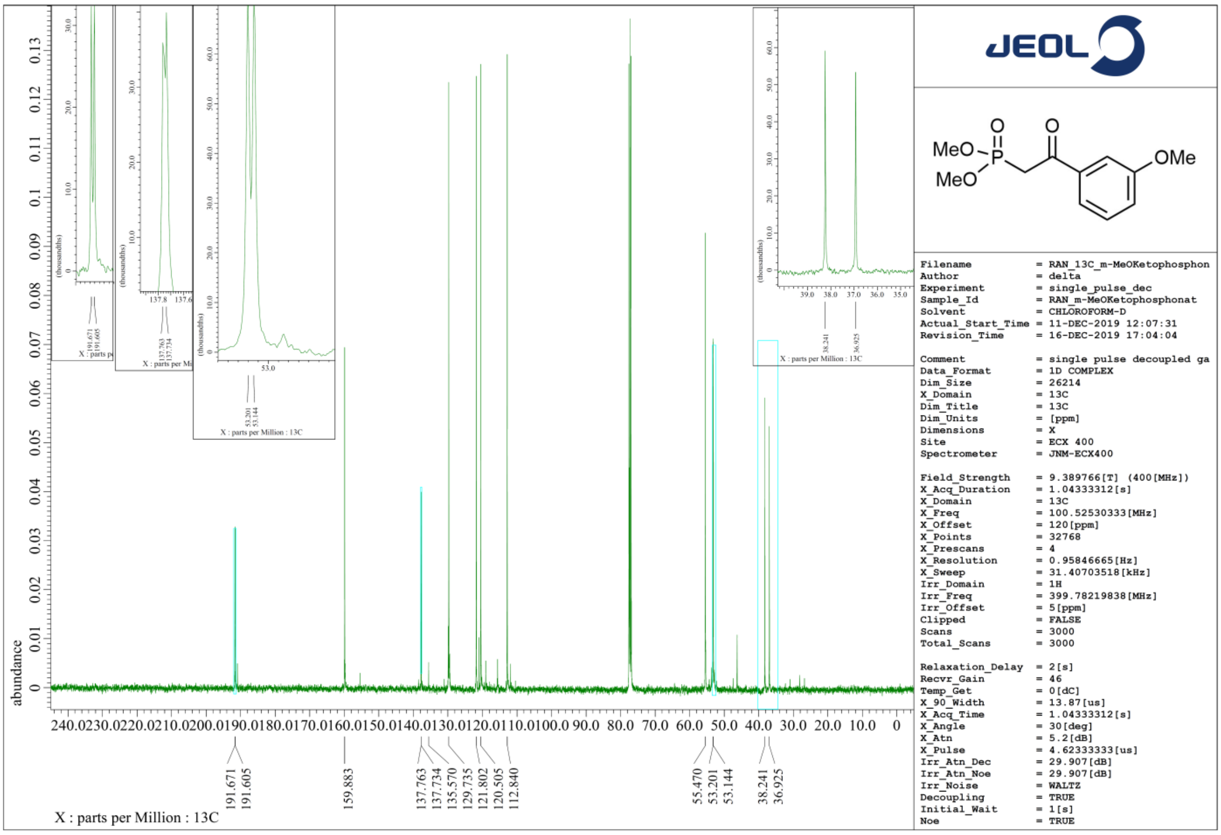

DEPT NMR spectra of dimethyl [2-(3-methoxyphenyl)-2-oxoethyl]phosphonate **VIc** in $CDCl_3$

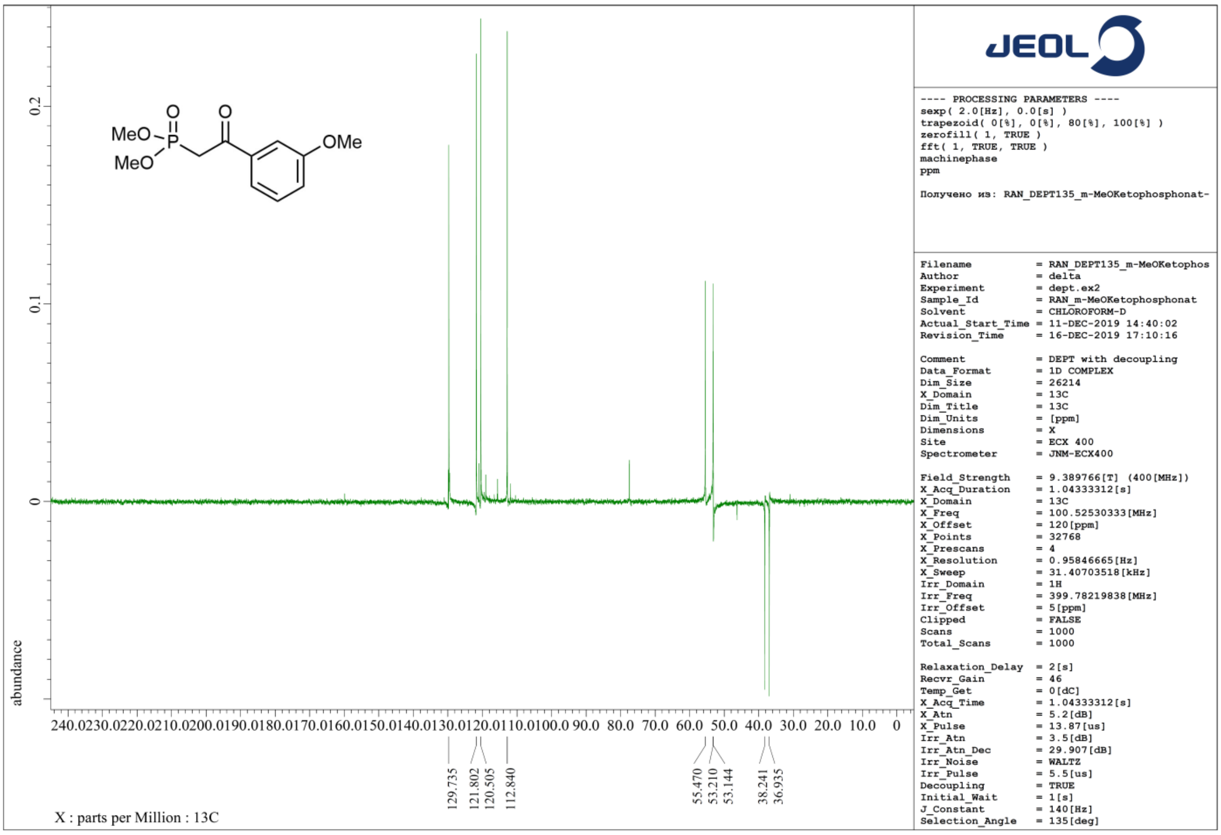

^{31}P NMR spectra of dimethyl [2-(3-methoxyphenyl)-2-oxoethyl]phosphonate **VIc** in $CDCl_3$

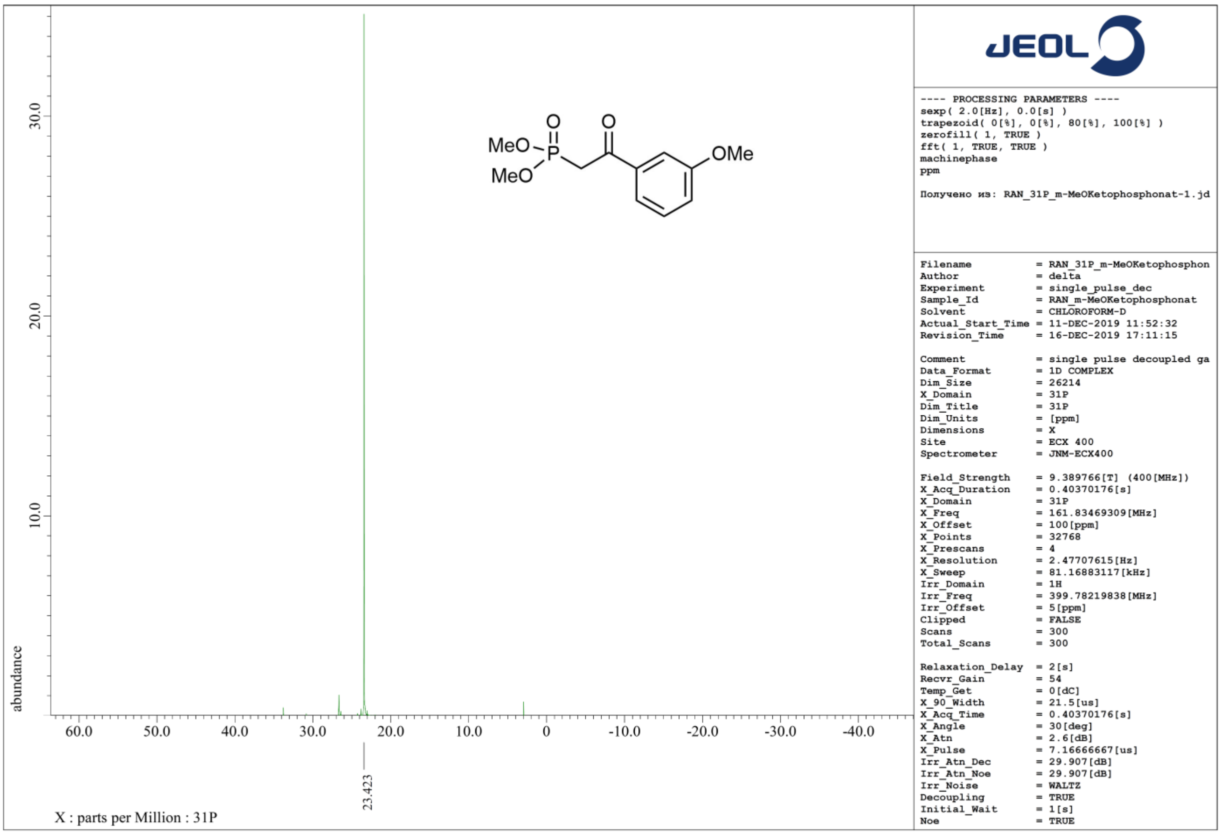

FTIR spectra of dimethyl [2-(2-methoxyphenyl)-2-oxoethyl]phosphonate **VId**

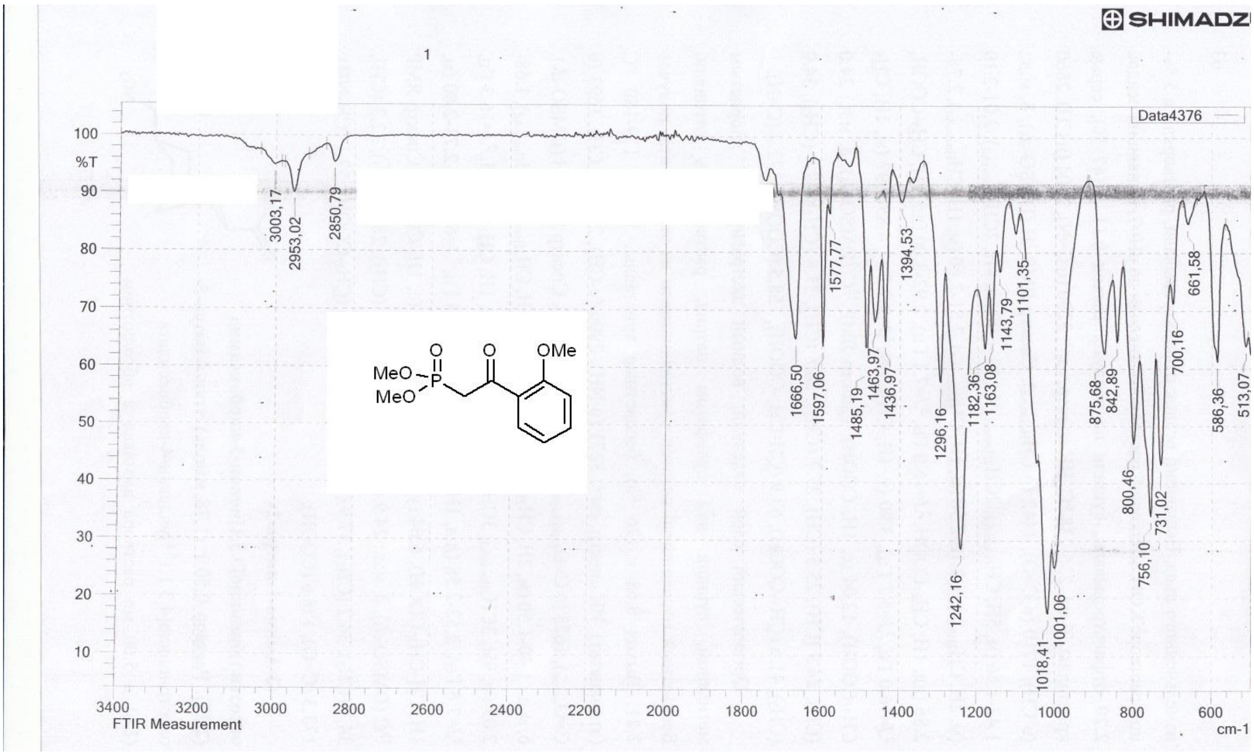

^{1}H NMR spectra of dimethyl [2-(2-methoxyphenyl)-2-oxoethyl]phosphonate **VId** in $CDCl_3$

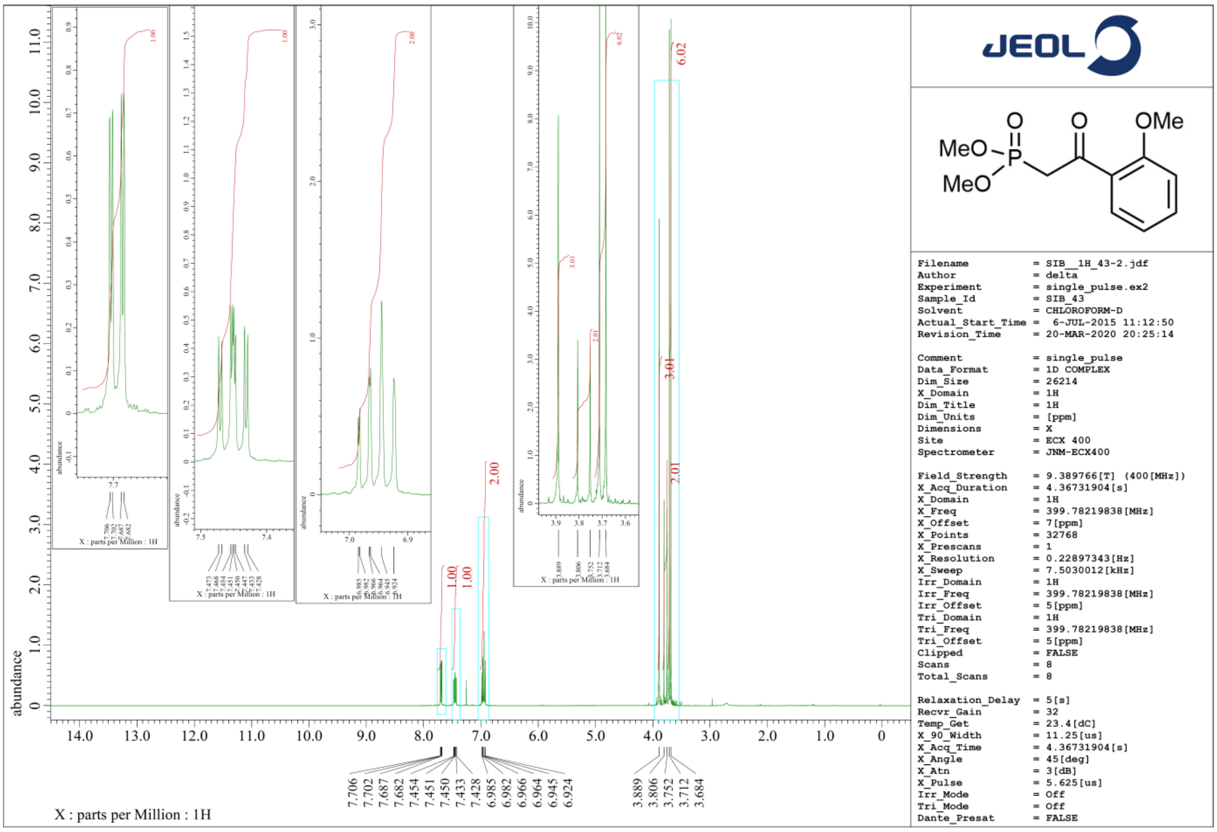

^{13}C NMR spectra of dimethyl [2-(2-methoxyphenyl)-2-oxoethyl]phosphonate **VId** in $CDCl_3$

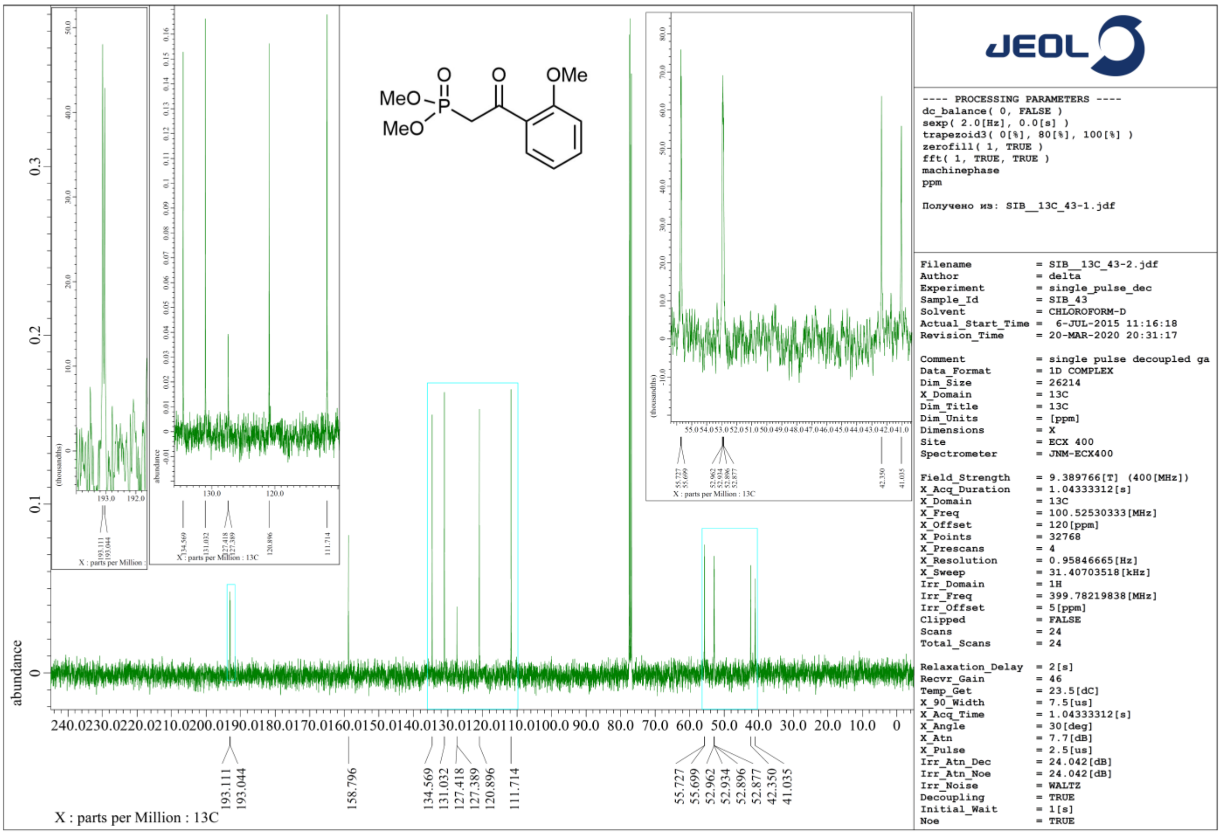

^{31}P NMR spectra of dimethyl [2-(2-methoxyphenyl)-2-oxoethyl]phosphonate **VId** in $CDCl_3$

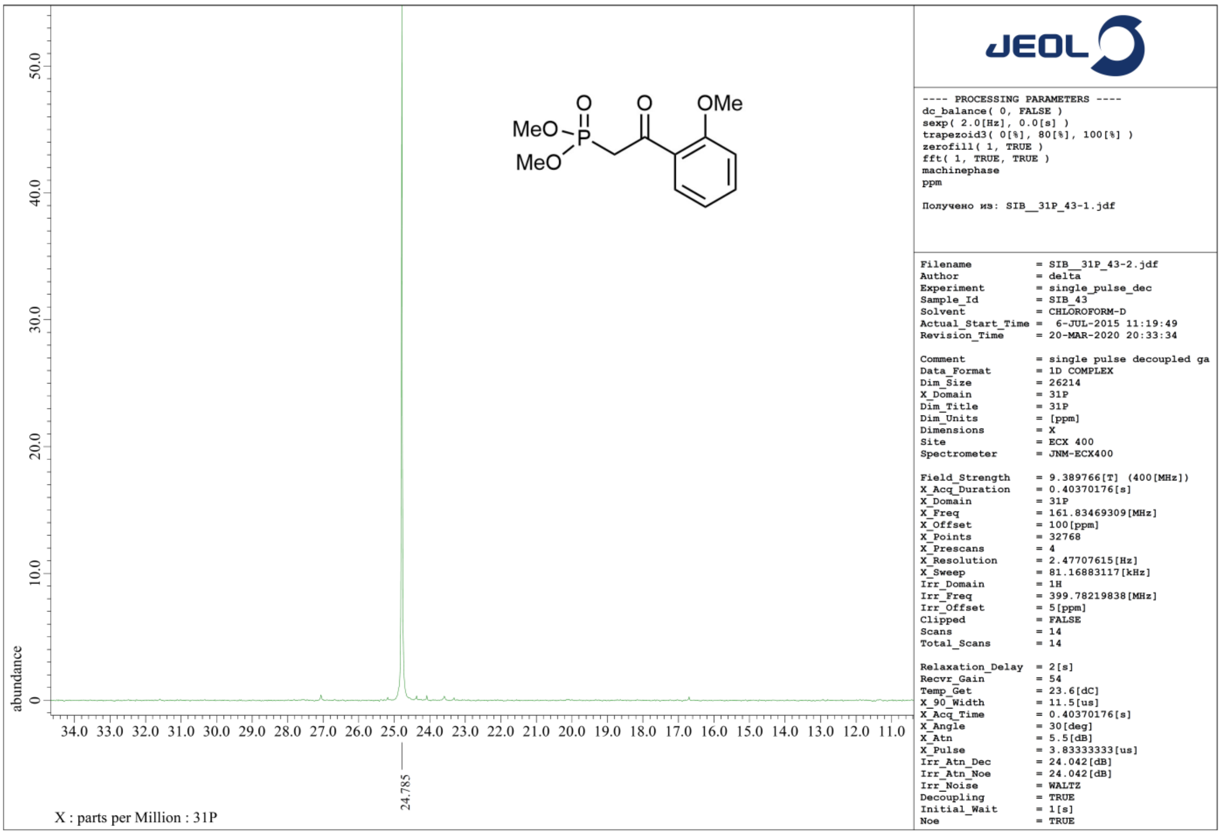

FTIR spectra of dimethyl (2-(adamantan-1-yl)-2-oxoethyl)phosphonate **VIe**

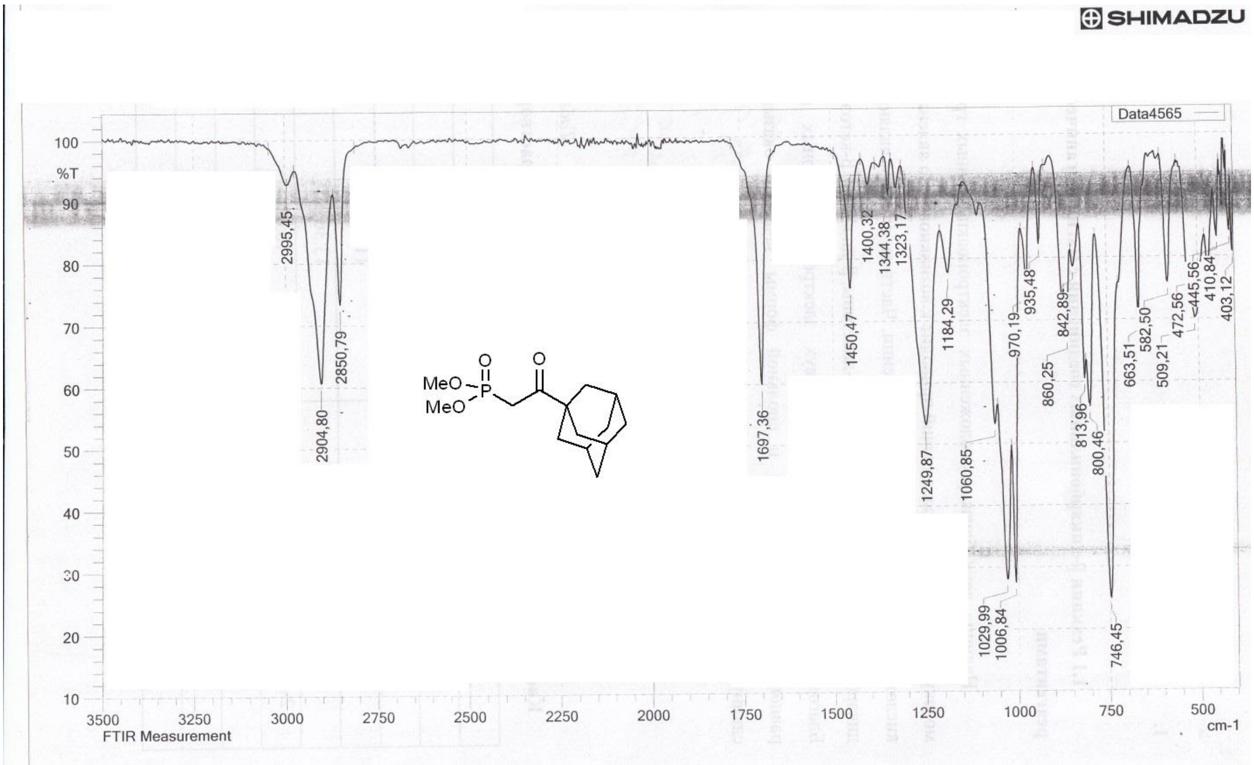

^{1}H NMR spectra of dimethyl (2-(adamantan-1-yl)-2-oxoethyl)phosphonate **VIe** in $CDCl_3$

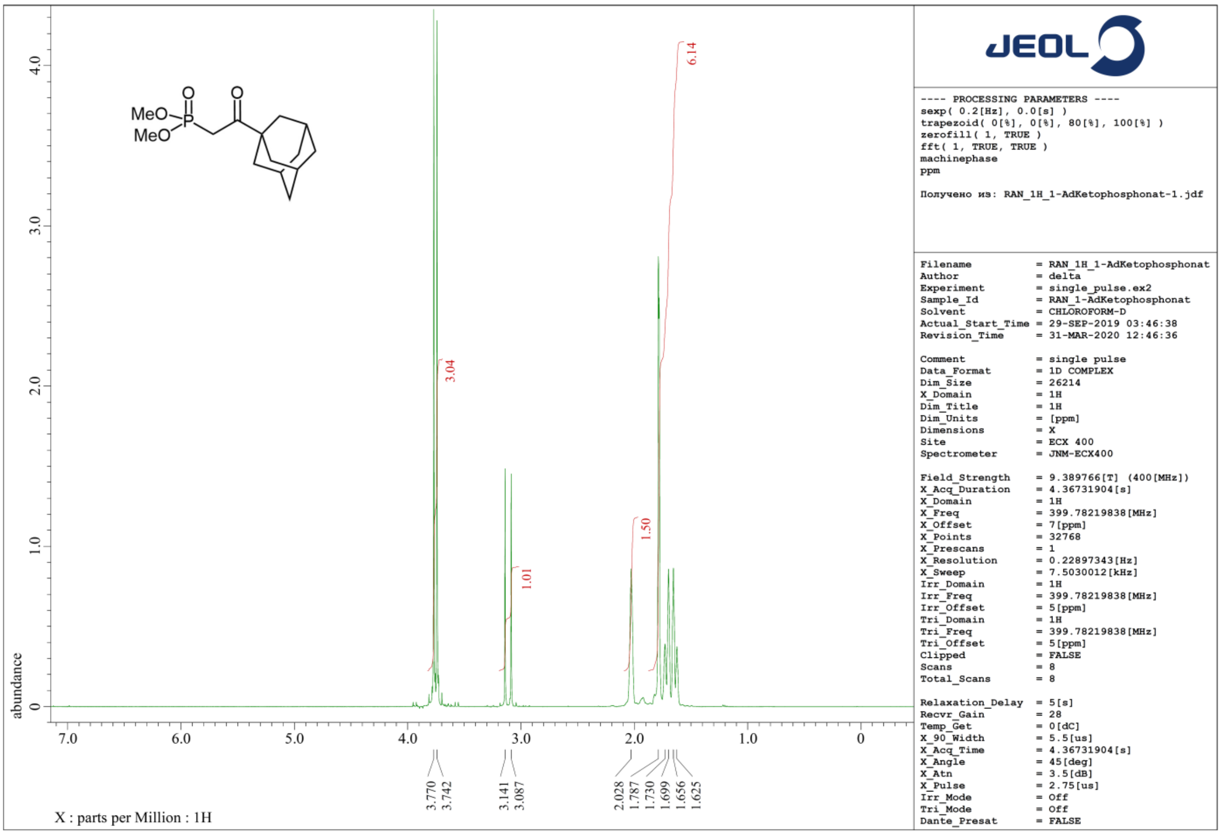

^{13}C NMR spectra of dimethyl (2-(adamantan-1-yl)-2-oxoethyl)phosphonate **VIe** in $CDCl_3$

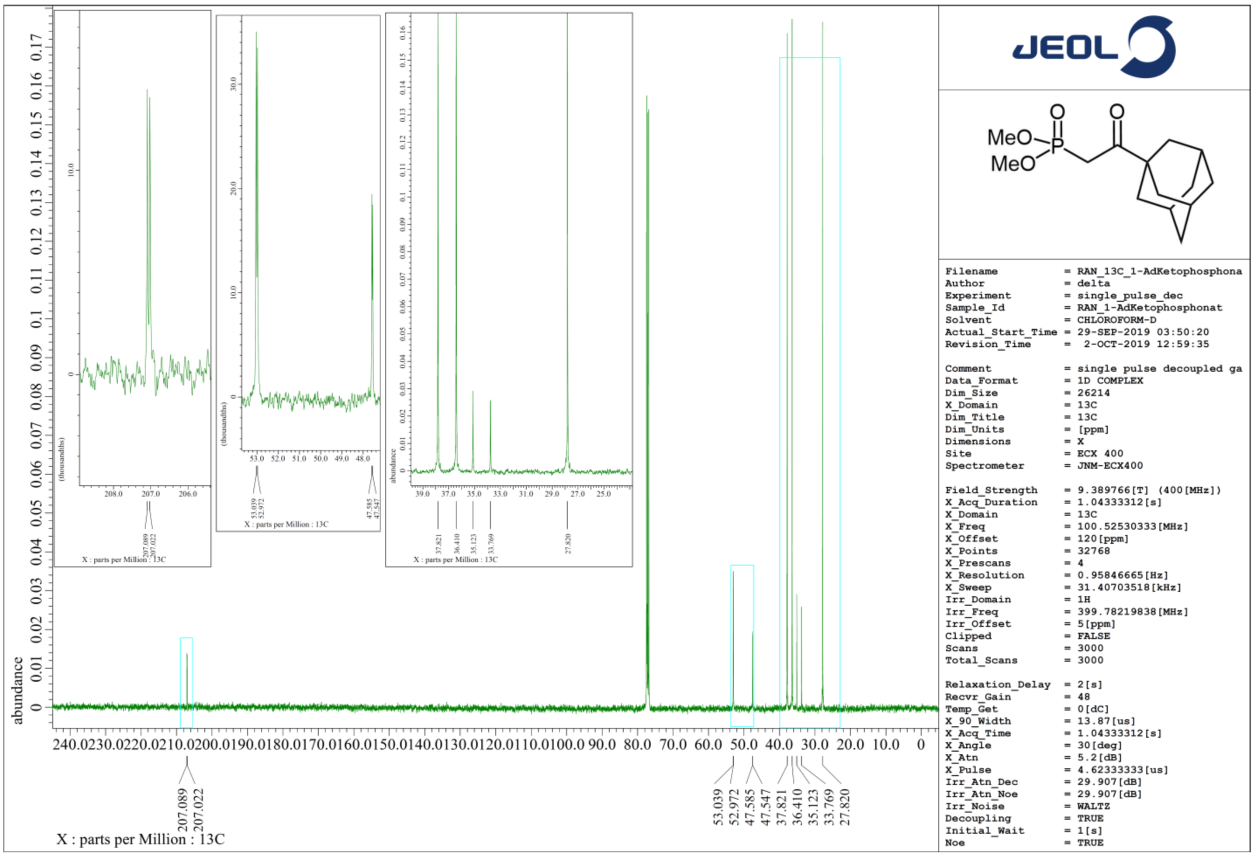

DEPT NMR spectra of dimethyl (2-(adamantan-1-yl)-2-oxoethyl)phosphonate **VIe** in $CDCl_3$

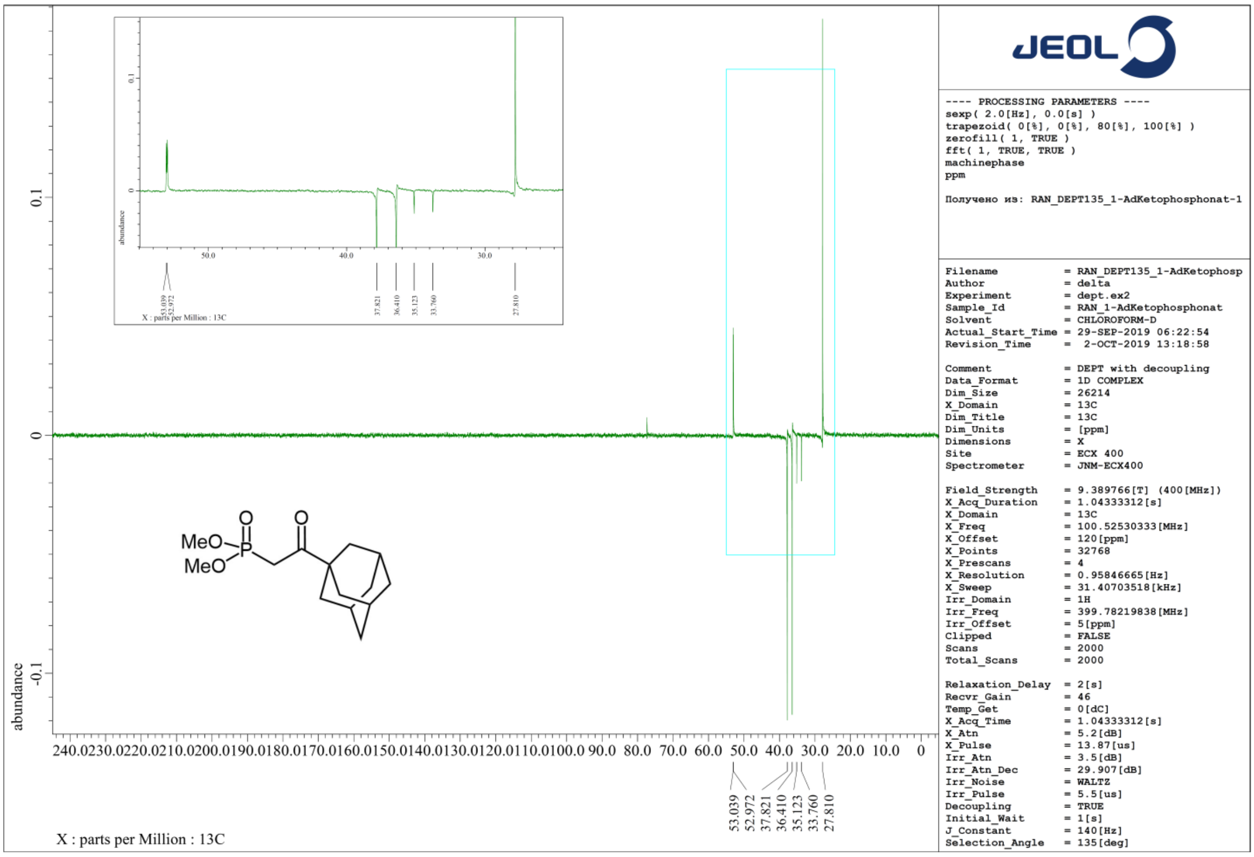

^{31}P NMR spectra of dimethyl (2-(adamantan-1-yl)-2-oxoethyl)phosphonate **VIe** in $CDCl_3$

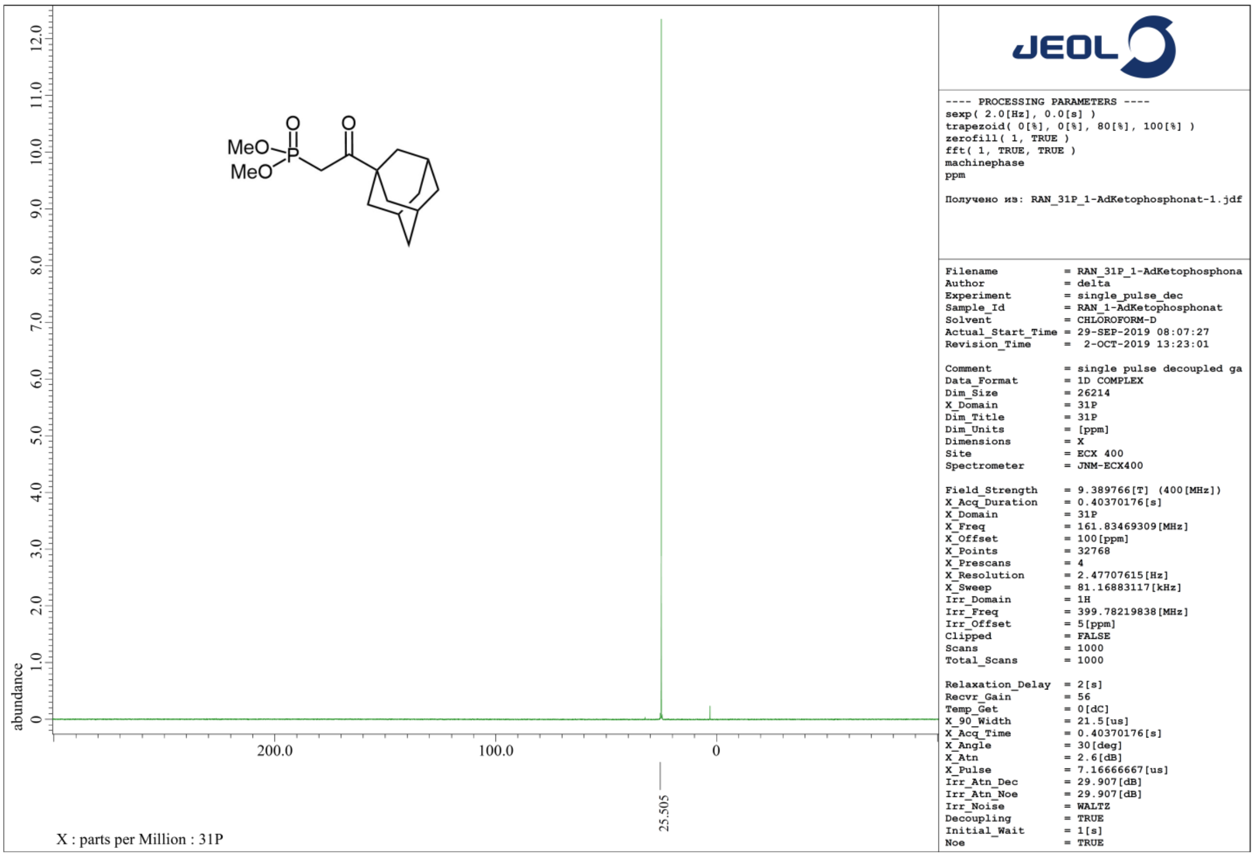

7. Copies of NMR, FTIR and mass spectra for phosphonates 6a-f

FTIR spectra of dimethyl [(2*R*,3*S*)-4-nitro-1-oxo-1,3-diphenylbutan-2-yl]phosphonate (**6a**)

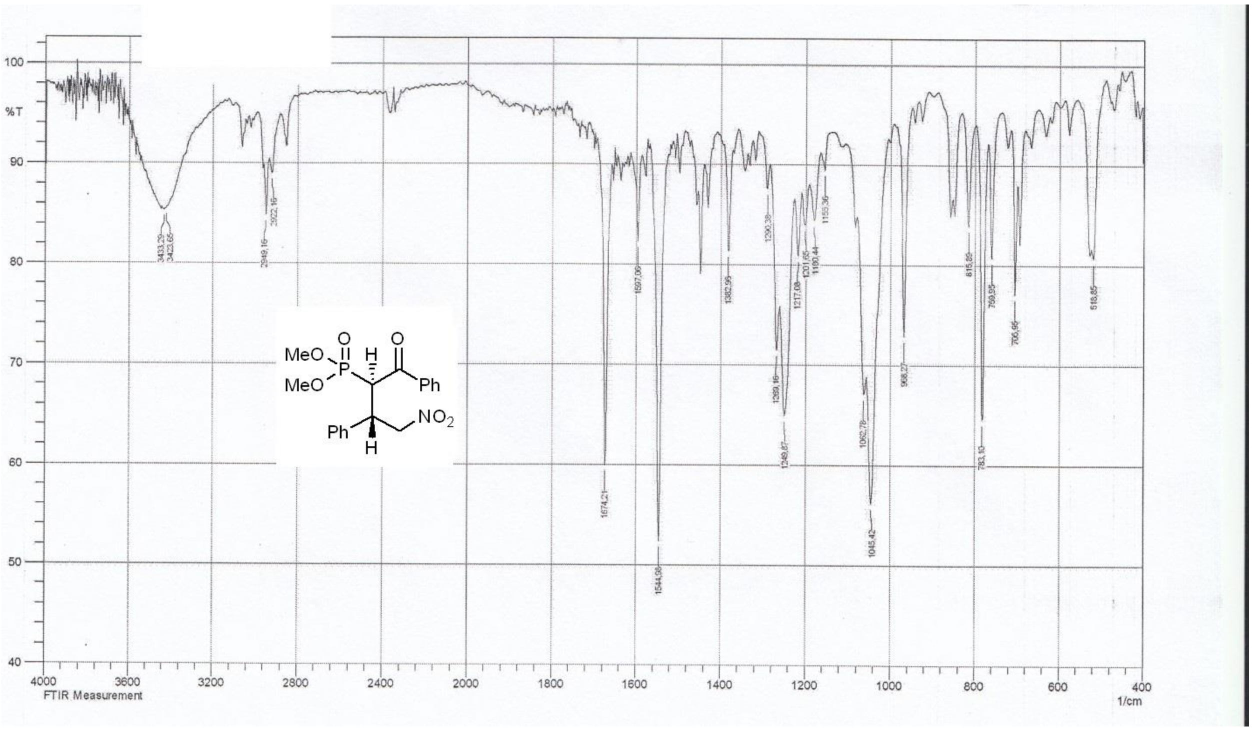

^{1}H NMR spectra of dimethyl [(2*R*,3*S*)-4-nitro-1-oxo-1,3-diphenylbutan-2-yl]phosphonate (**6a**) in $CDCl_3$

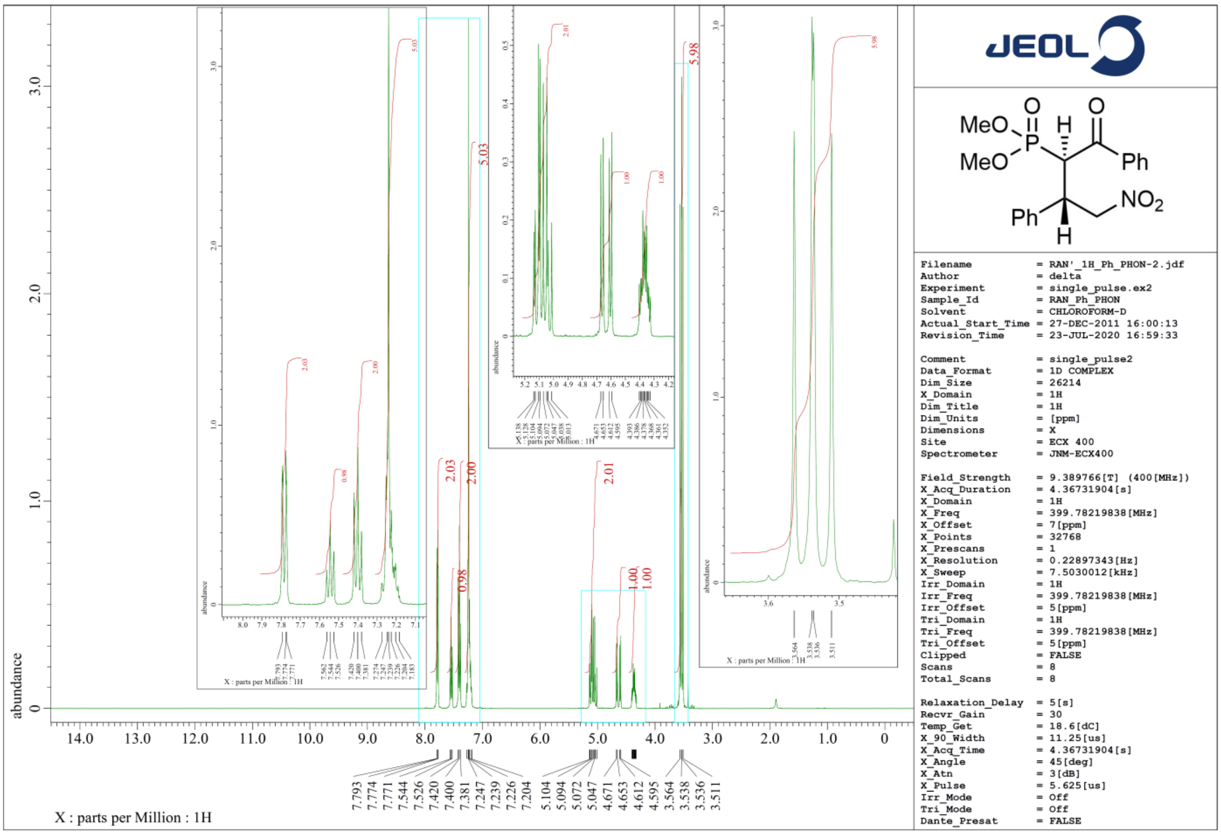

^{13}C NMR spectra of dimethyl [(2*R*,3*S*)-4-nitro-1-oxo-1,3-diphenylbutan-2-yl]phosphonate (**6a**) in $CDCl_3$

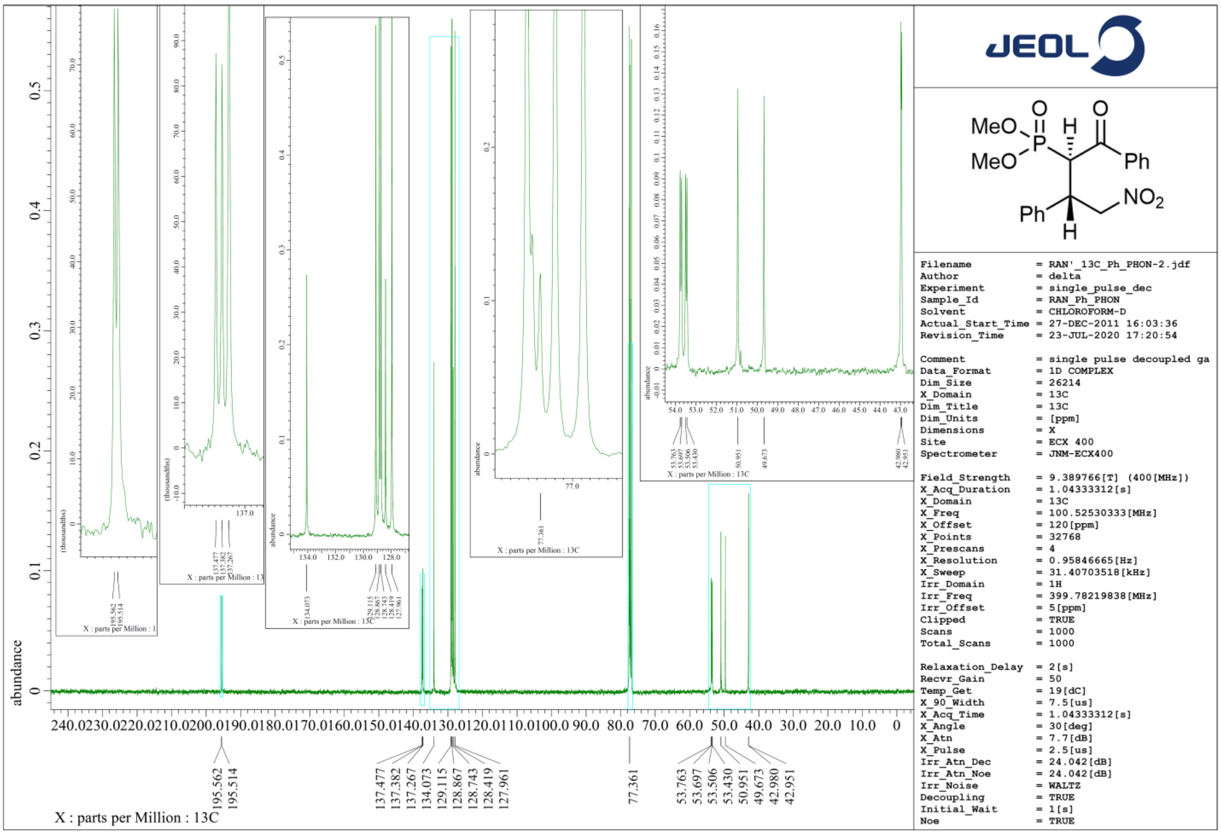

^{31}P NMR spectra of dimethyl [(2*R*,3*S*)-4-nitro-1-oxo-1,3-diphenylbutan-2-yl]phosphonate (**6a**) in $CDCl_3$

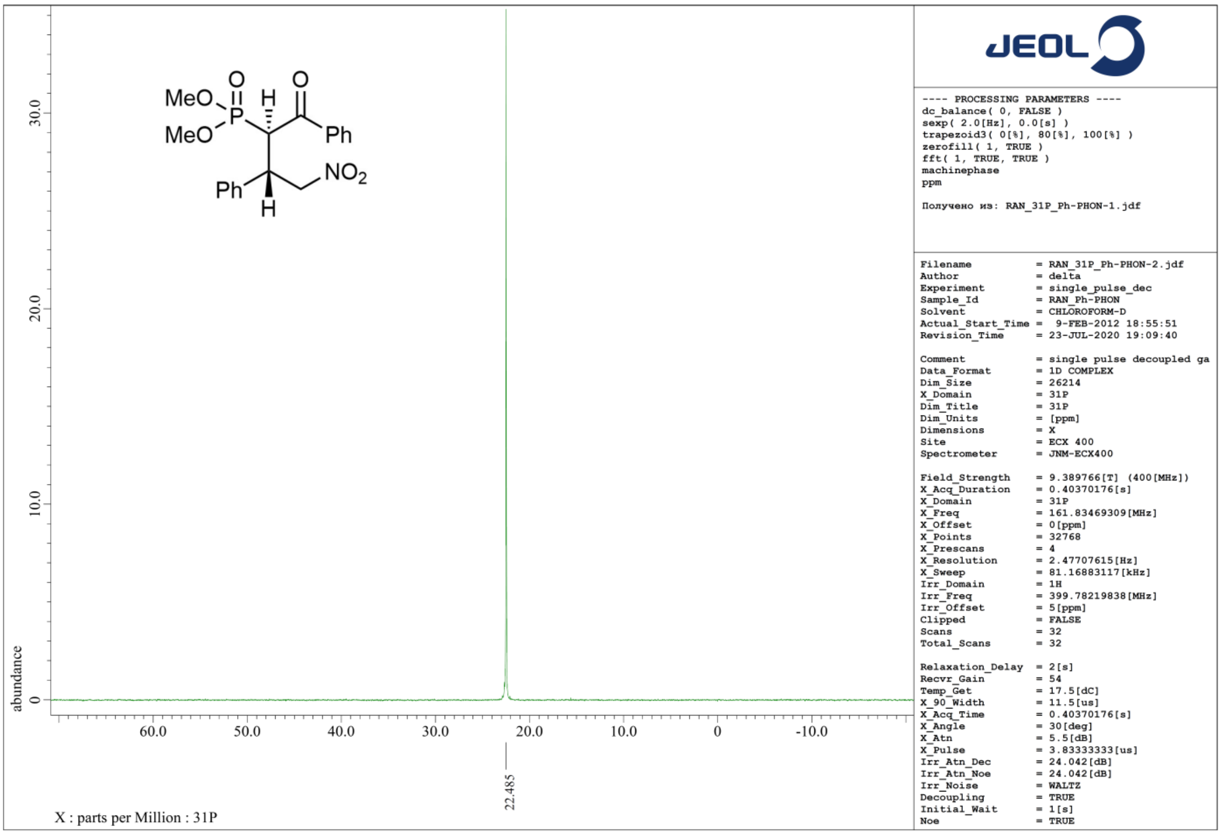

HRMS of dimethyl [(2*R*,3*S*)-4-nitro-1-oxo-1,3-diphenylbutan-2-yl]phosphonate (**6a**)

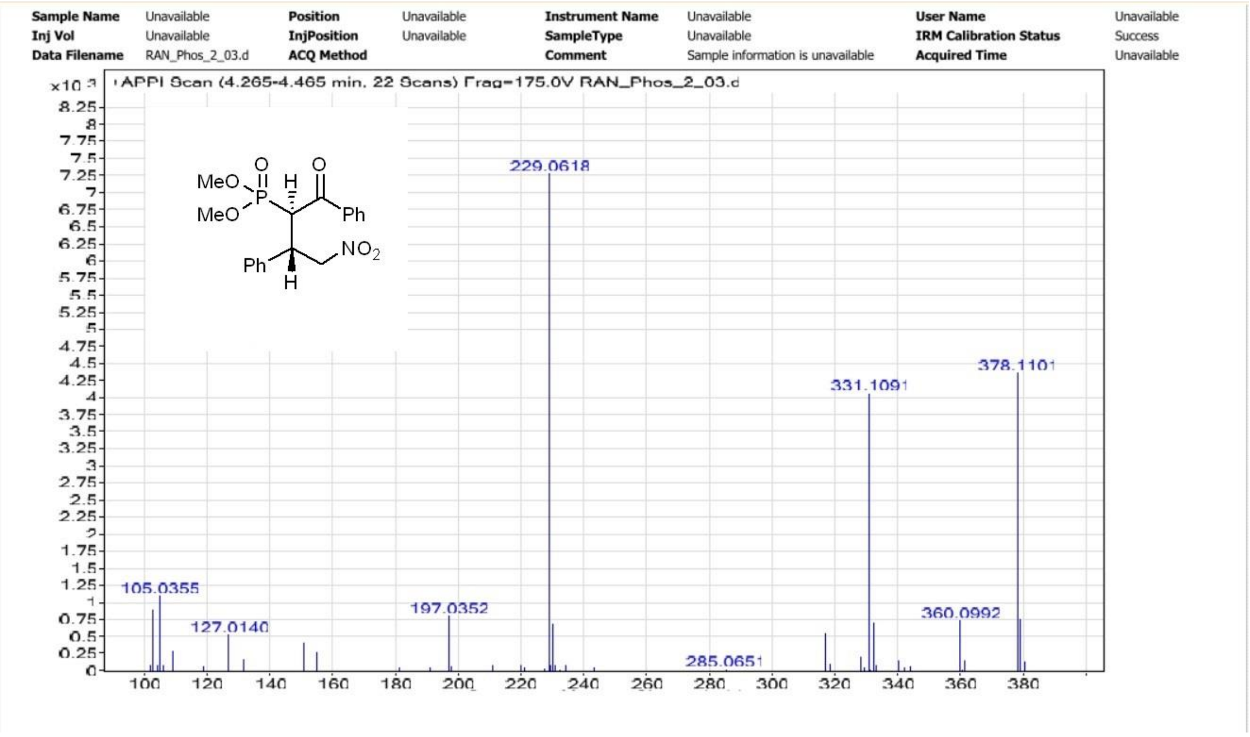

FTIR spectra of dimethyl [(2*R*,3*S*)-1-(4-methoxyphenyl)-4-nitro-1-oxo-3-phenylbutan-2-yl]-phosphonate

(**6b**)

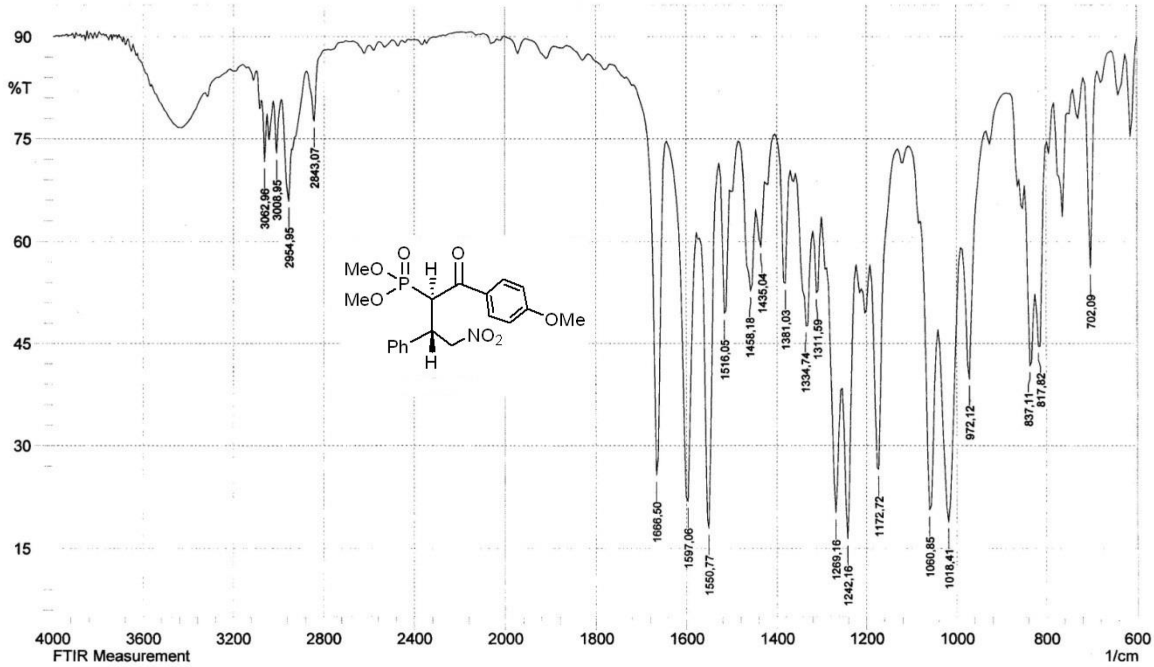

^{1}H NMR spectra of dimethyl [(2*R*,3*S*)-1-(4-methoxyphenyl)-4-nitro-1-oxo-3-phenylbutan-2-yl]-phosphonate (**6b**) in $CDCl_3$

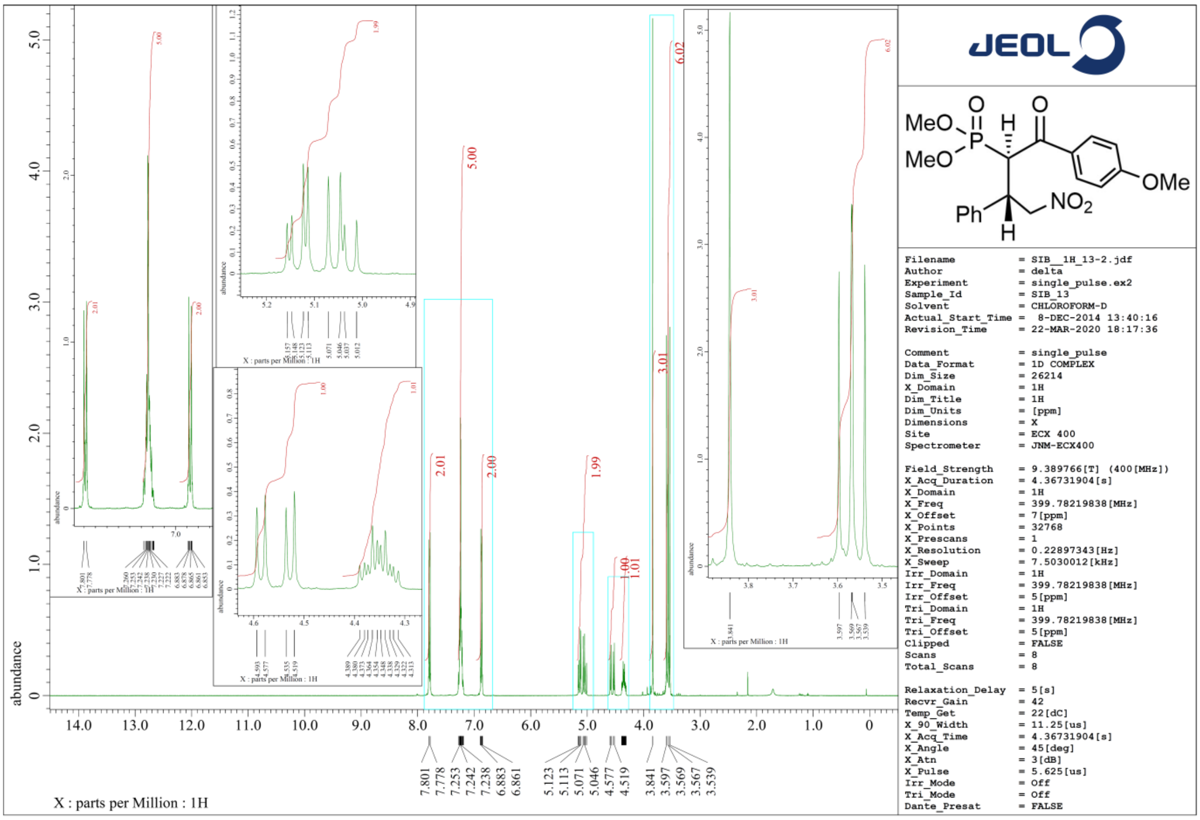

^{13}C NMR spectra of dimethyl [(2*R*,3*S*)-1-(4-methoxyphenyl)-4-nitro-1-oxo-3-phenylbutan-2-yl]-phosphonate (**6b**) in $CDCl_3$

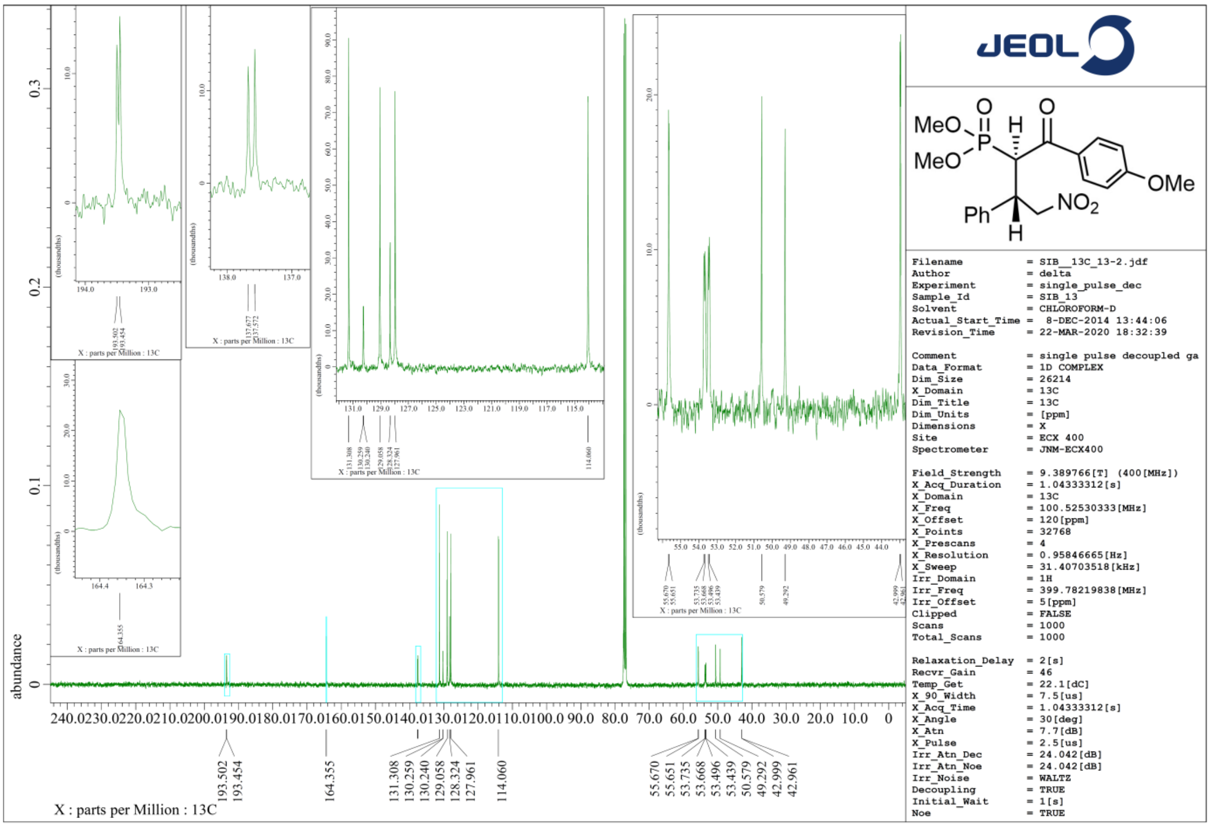

^{31}P NMR spectra of dimethyl [(2*R*,3*S*)-1-(4-methoxyphenyl)-4-nitro-1-oxo-3-phenylbutan-2-yl]-phosphonate (**6b**) in $CDCl_3$

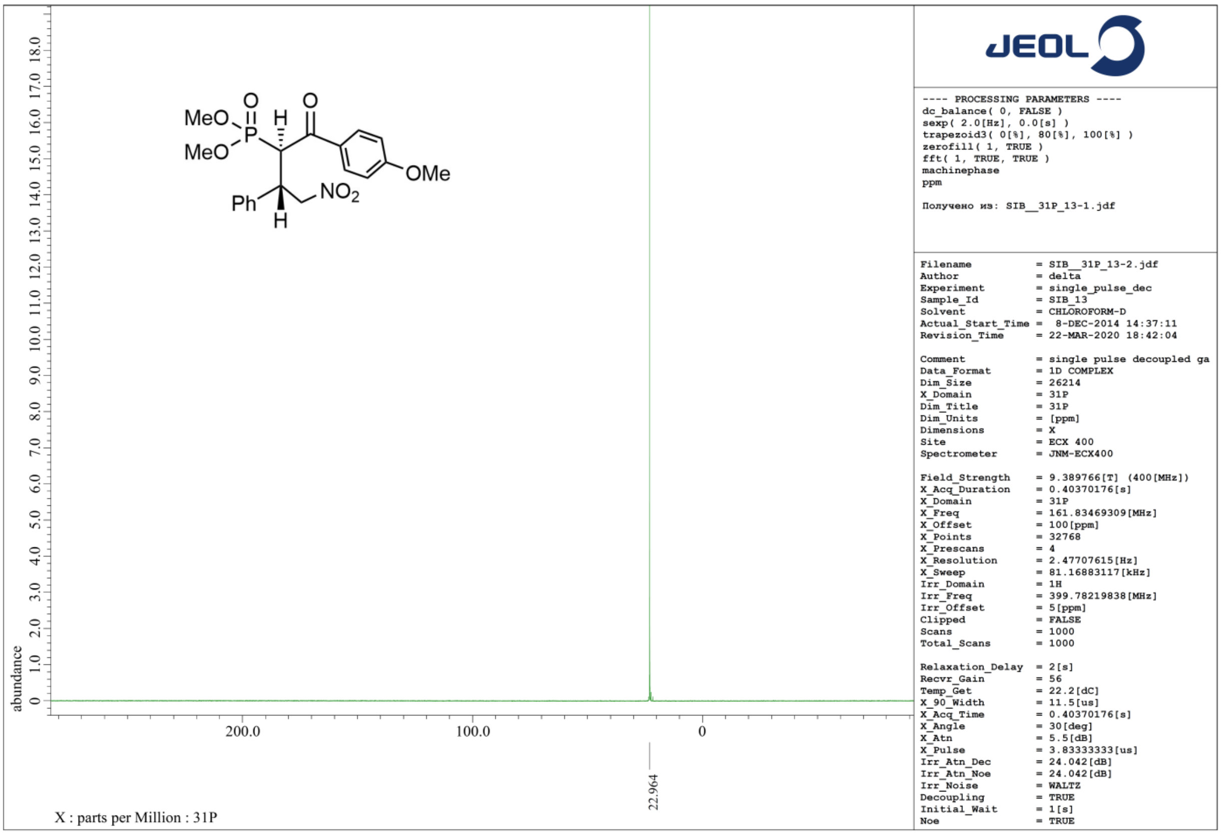

HRMS of dimethyl [(2*R*,3*S*)-1-(4-methoxyphenyl)-4-nitro-1-oxo-3-phenylbutan-2-yl]-phosphonate (**6b**)

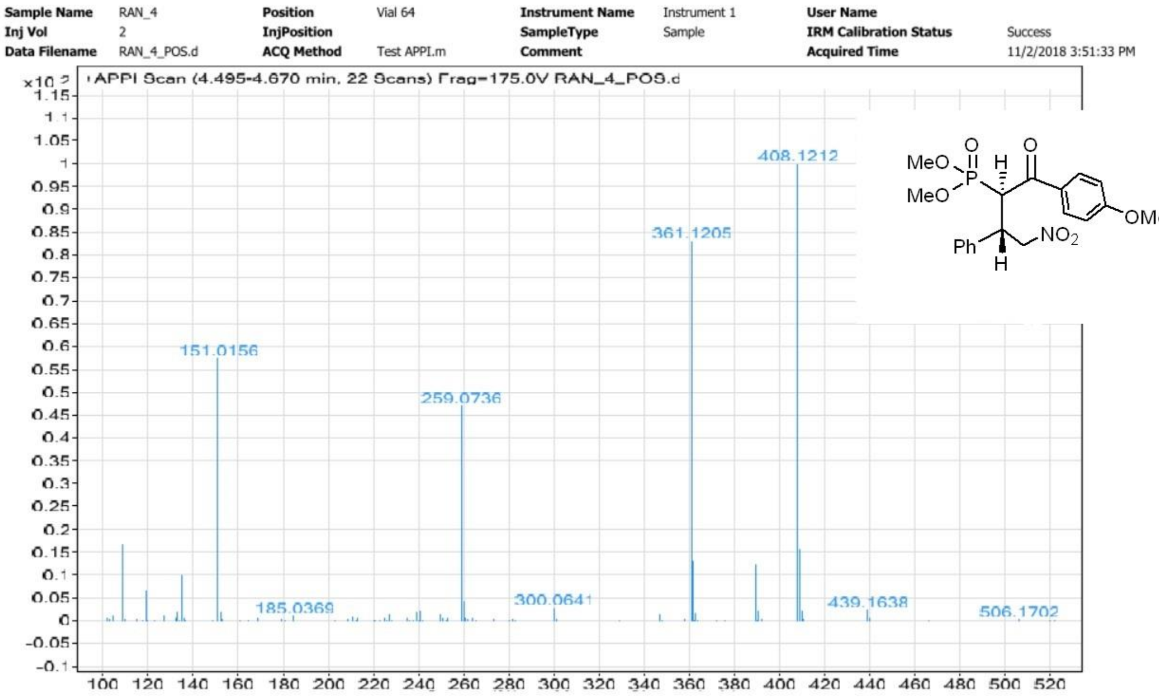

FTIR spectra of dimethyl [(2*R*,3*S*)-1-(3-methoxyphenyl)-4-nitro-1-oxo-3-phenylbutan-2-yl]phosphonate (**6c**)

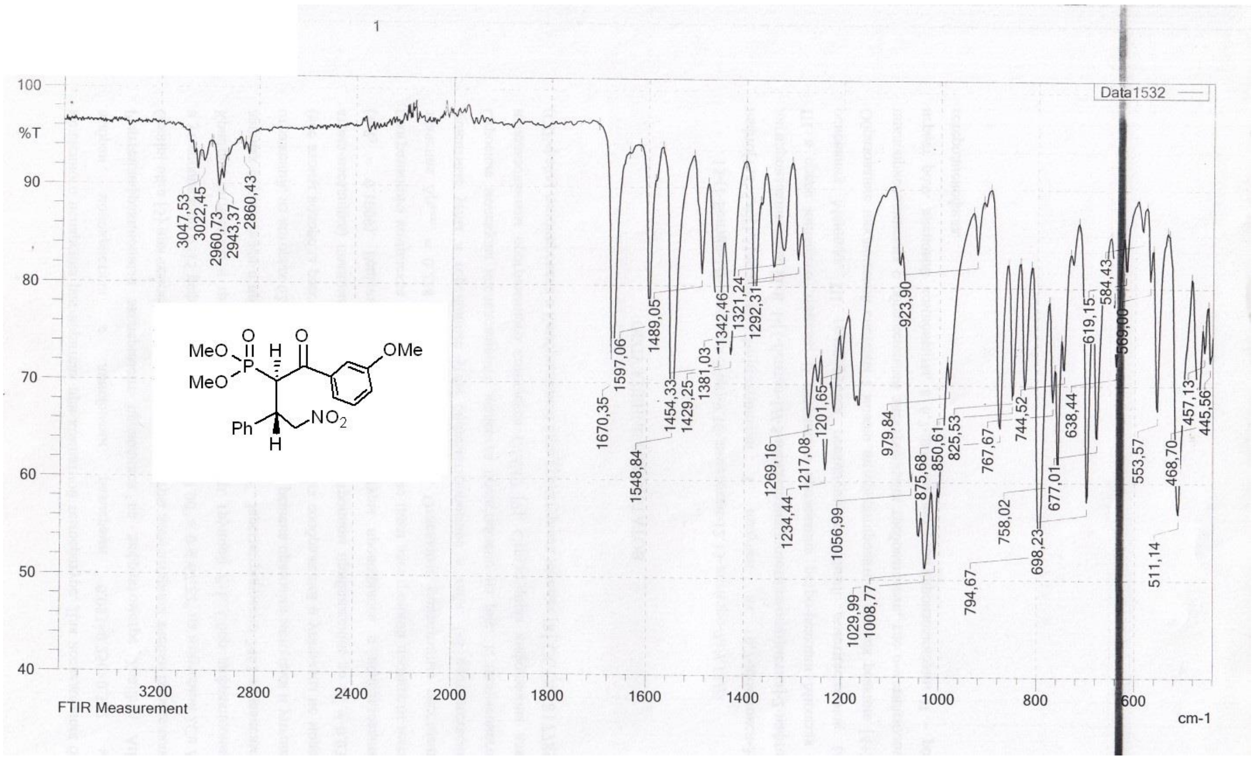

¹H NMR spectra of dimethyl [(2*R*,3*S*)-1-(3-methoxyphenyl)-4-nitro-1-oxo-3-phenylbutan-2-yl]phosphonate (**6c**) in $CDCl_3$

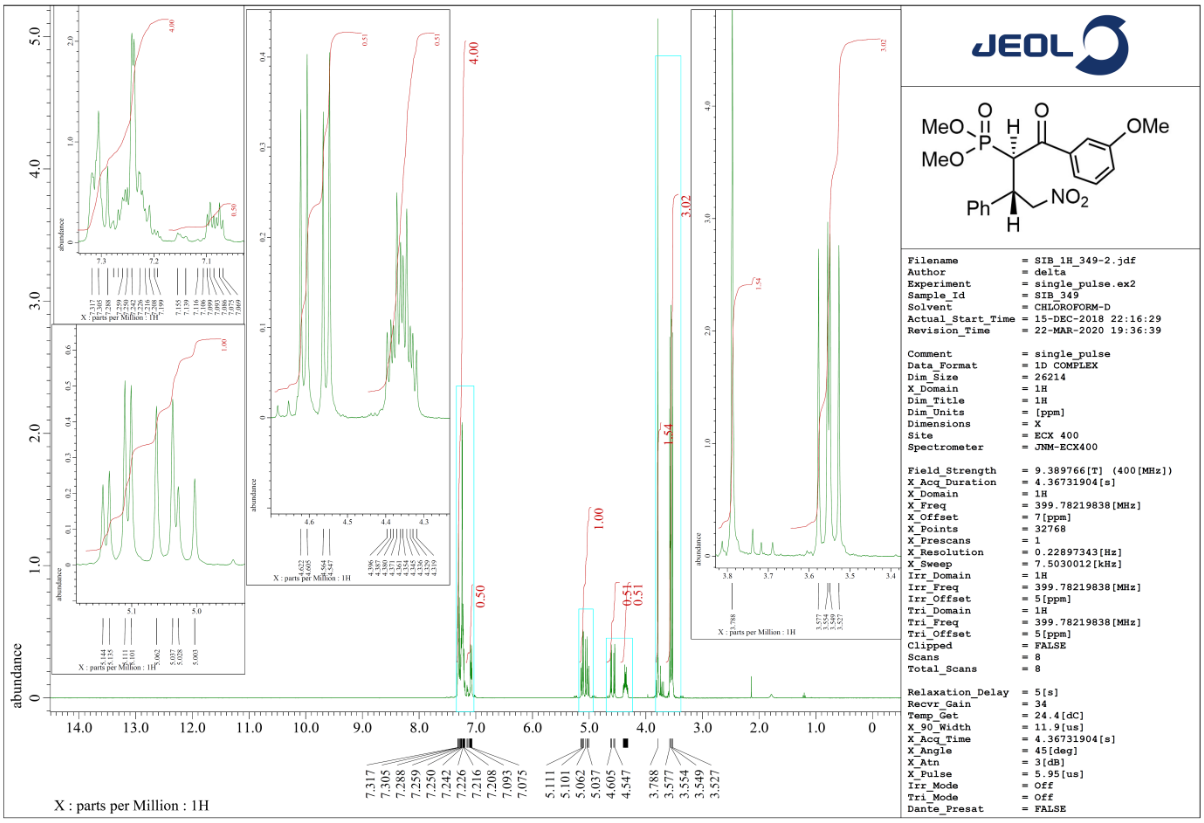

^{13}C NMR spectra of dimethyl [(2*R*,3*S*)-1-(3-methoxyphenyl)-4-nitro-1-oxo-3-phenylbutan-2-yl]phosphonate (**6c**) in $CDCl_3$

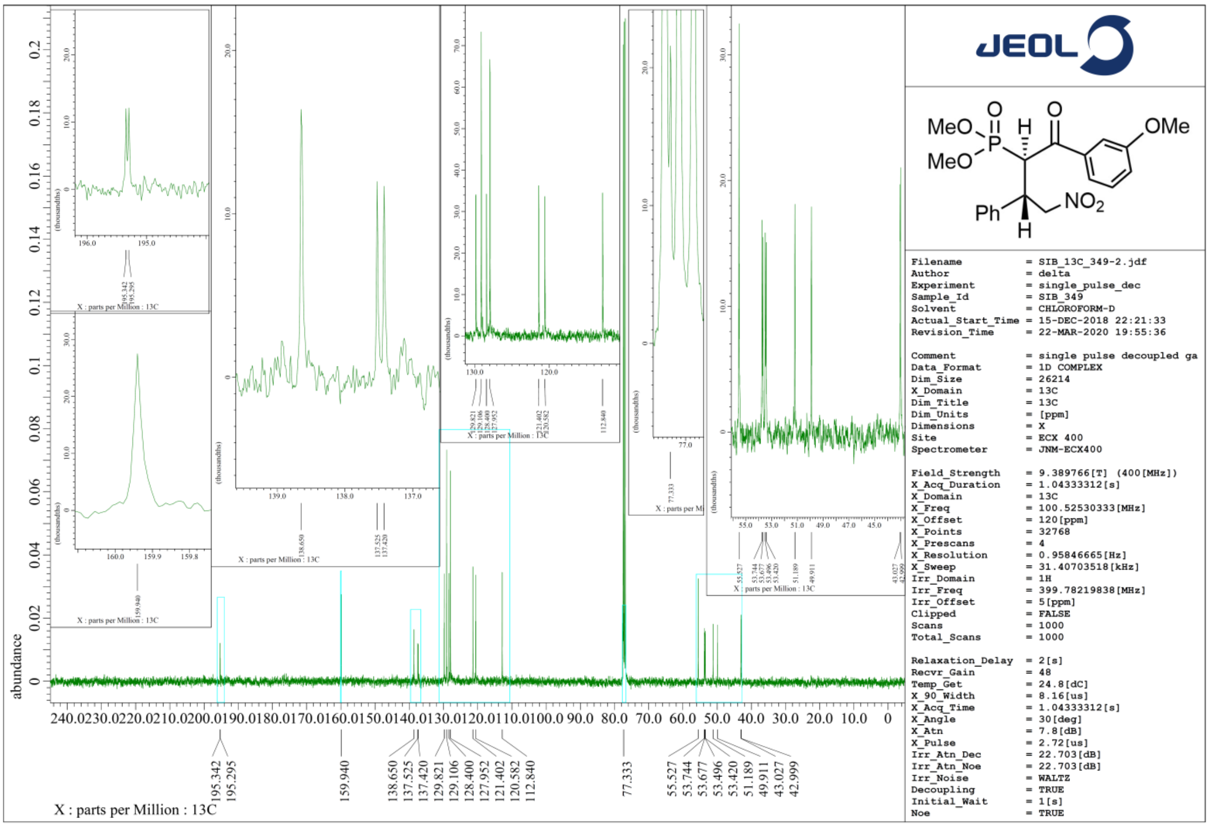

^{31}P NMR spectra of dimethyl [(2*R*,3*S*)-1-(3-methoxyphenyl)-4-nitro-1-oxo-3-phenylbutan-2-yl]phosphonate (**6c**) in $CDCl_3$

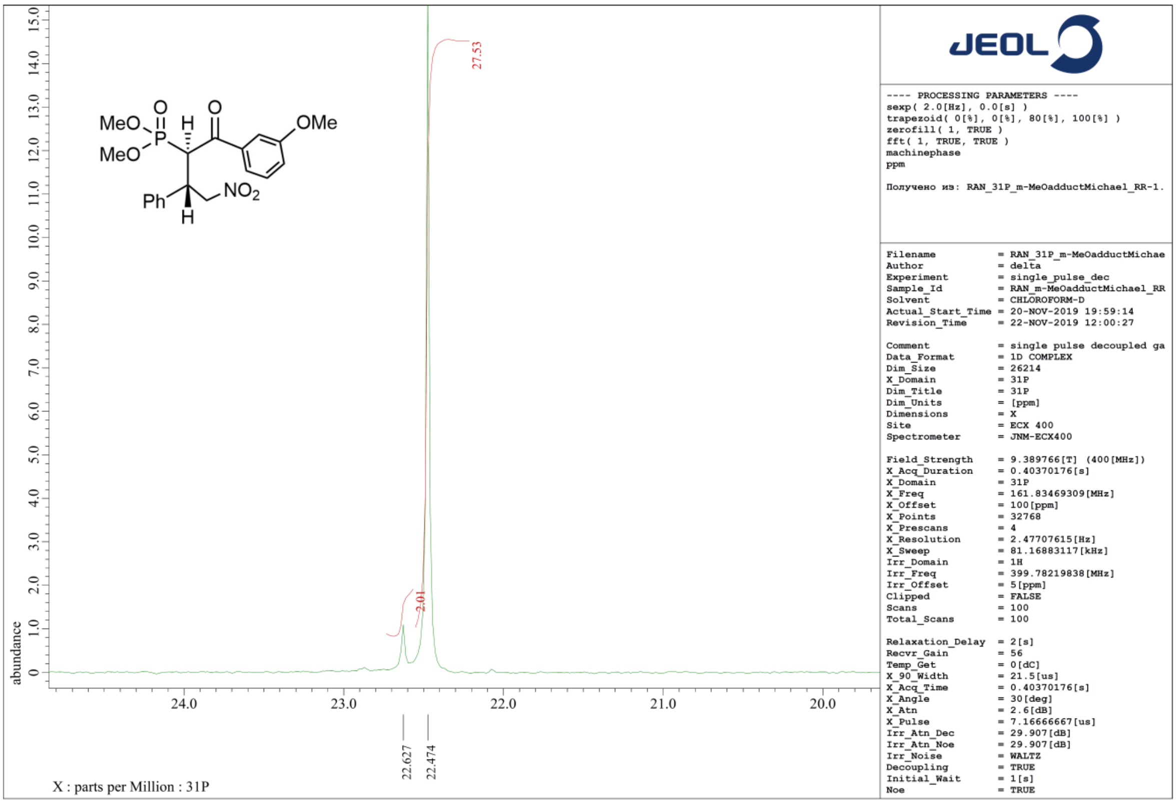

HRMS of dimethyl [(2*R*,3*S*)-1-(3-methoxyphenyl)-4-nitro-1-oxo-3-phenylbutan-2-yl]phosphonate (**6c**)

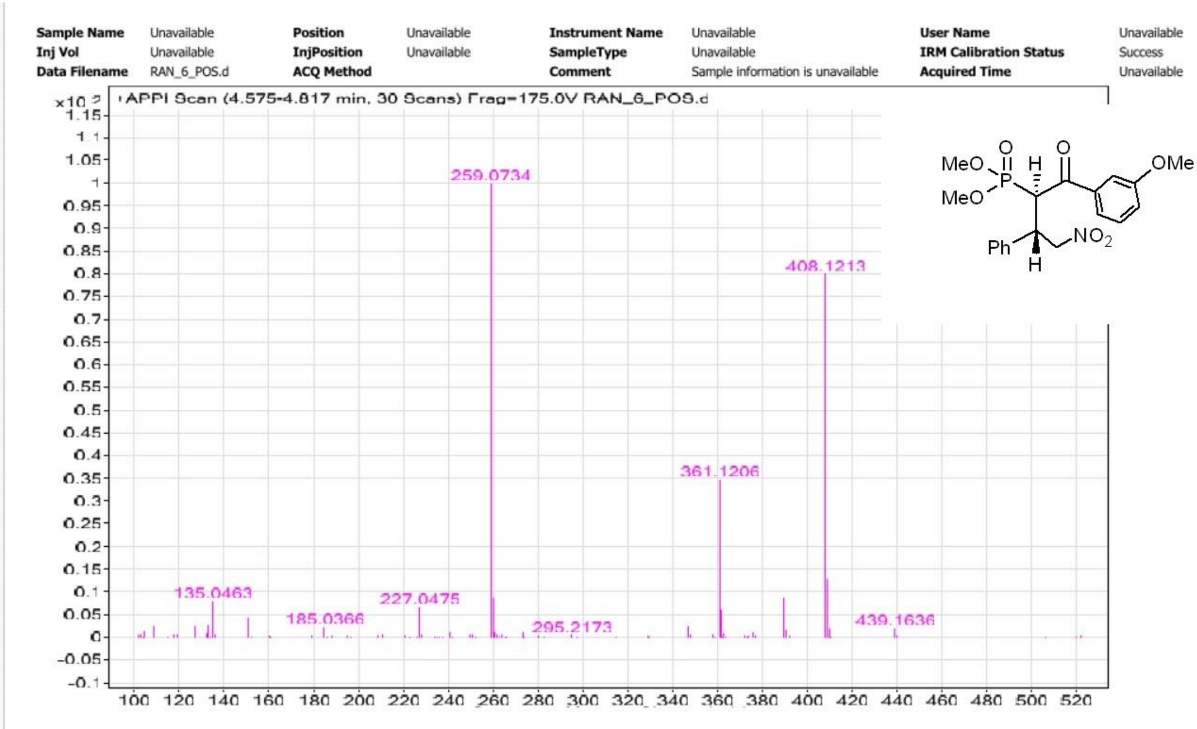

FTIR spectra of dimethyl [(2*R*,3*R*)-1-(3-methoxyphenyl)-4-nitro-1-oxo-3-phenylbutan-2-yl]phosphonate

(**6c**)

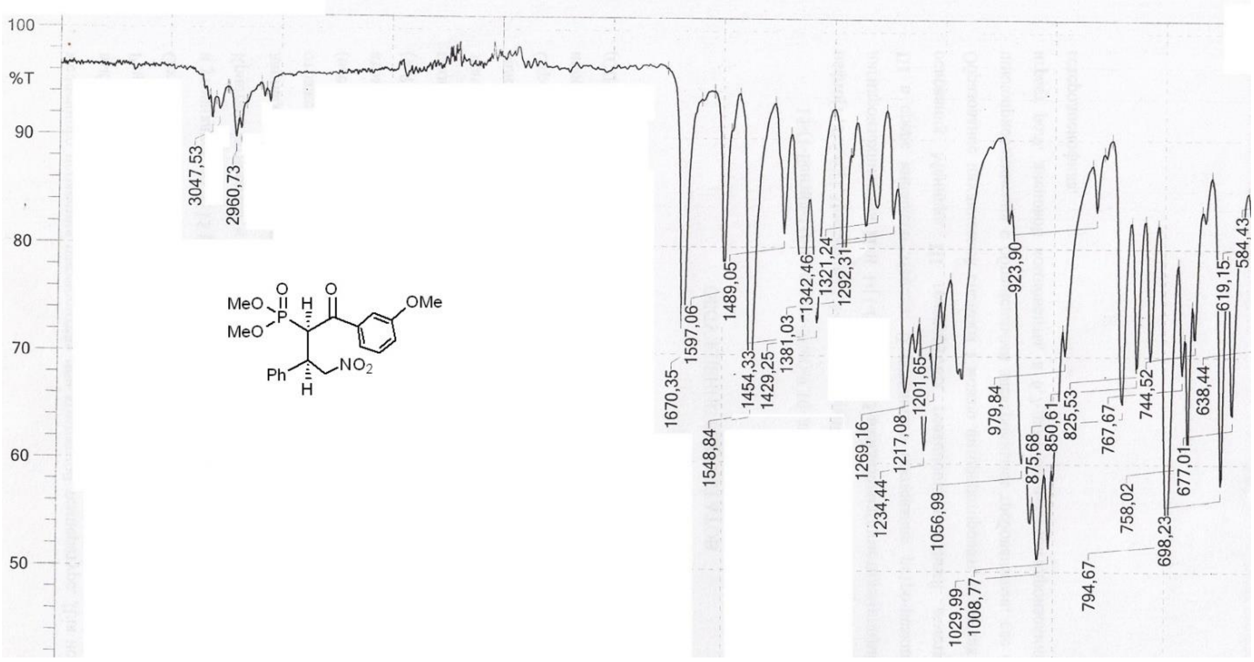

^{1}H NMR spectra of dimethyl [(2*R*,3*R*)-1-(3-methoxyphenyl)-4-nitro-1-oxo-3-phenylbutan-2-yl]phosphonate (**6c**) in $CDCl_3$

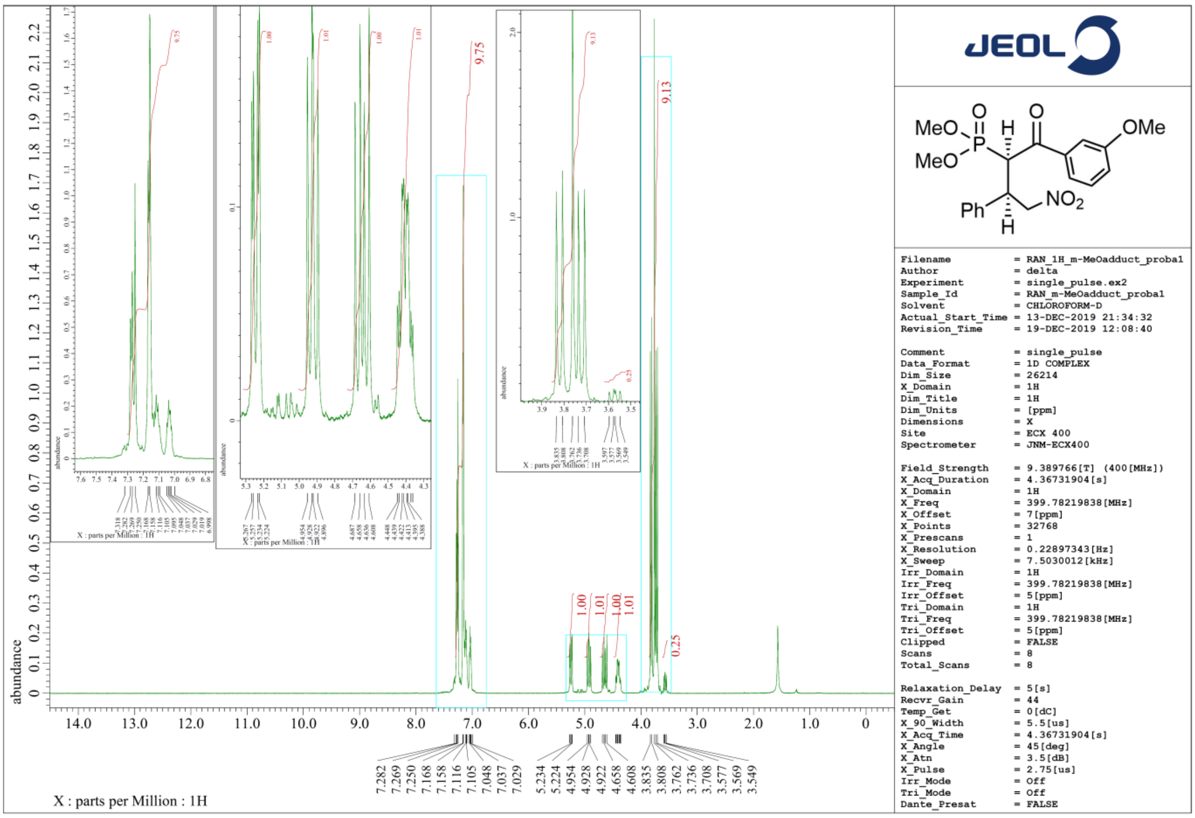

^{13}C NMR spectra of dimethyl [(2*R*,3*R*)-1-(3-methoxyphenyl)-4-nitro-1-oxo-3-phenylbutan-2-yl]phosphonate (**6c**) in $CDCl_3$

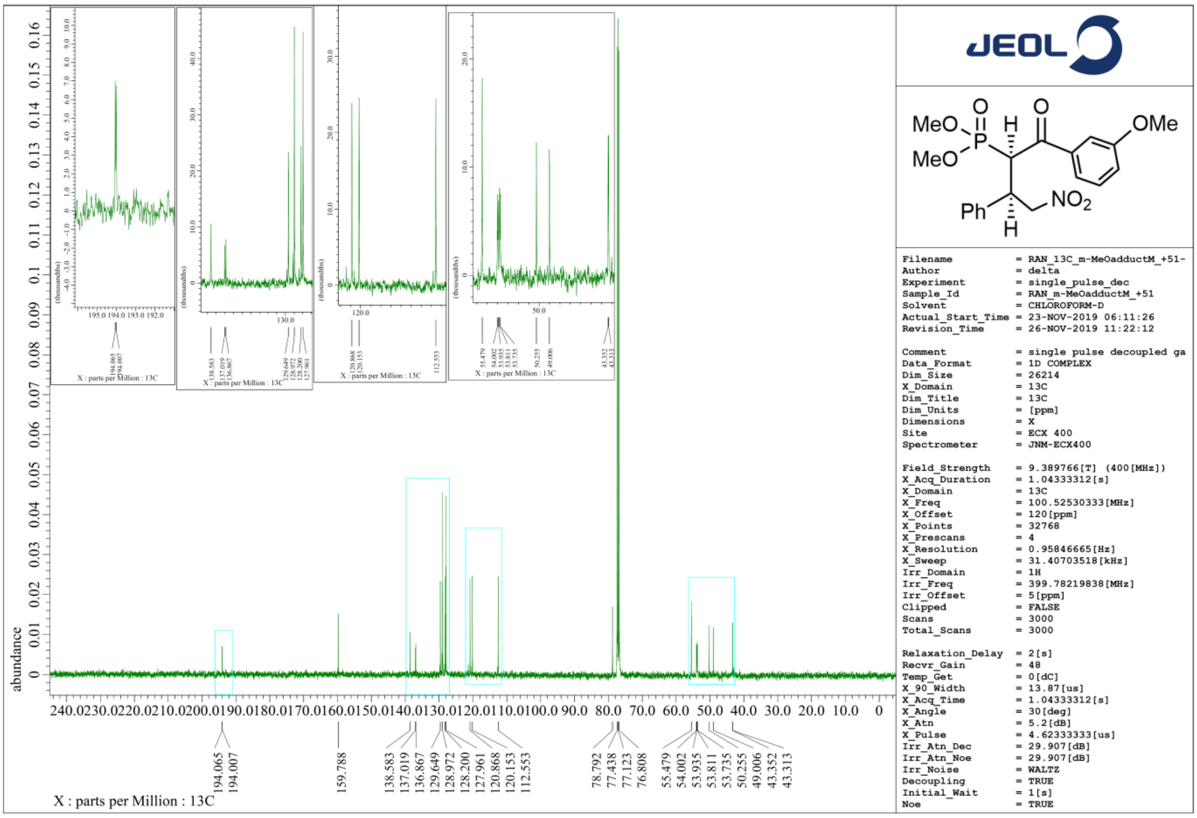

DEPT NMR spectra of dimethyl [(2*R*,3*R*)-1-(3-methoxyphenyl)-4-nitro-1-oxo-3-phenylbutan-2-yl]phosphonate (**6c**)

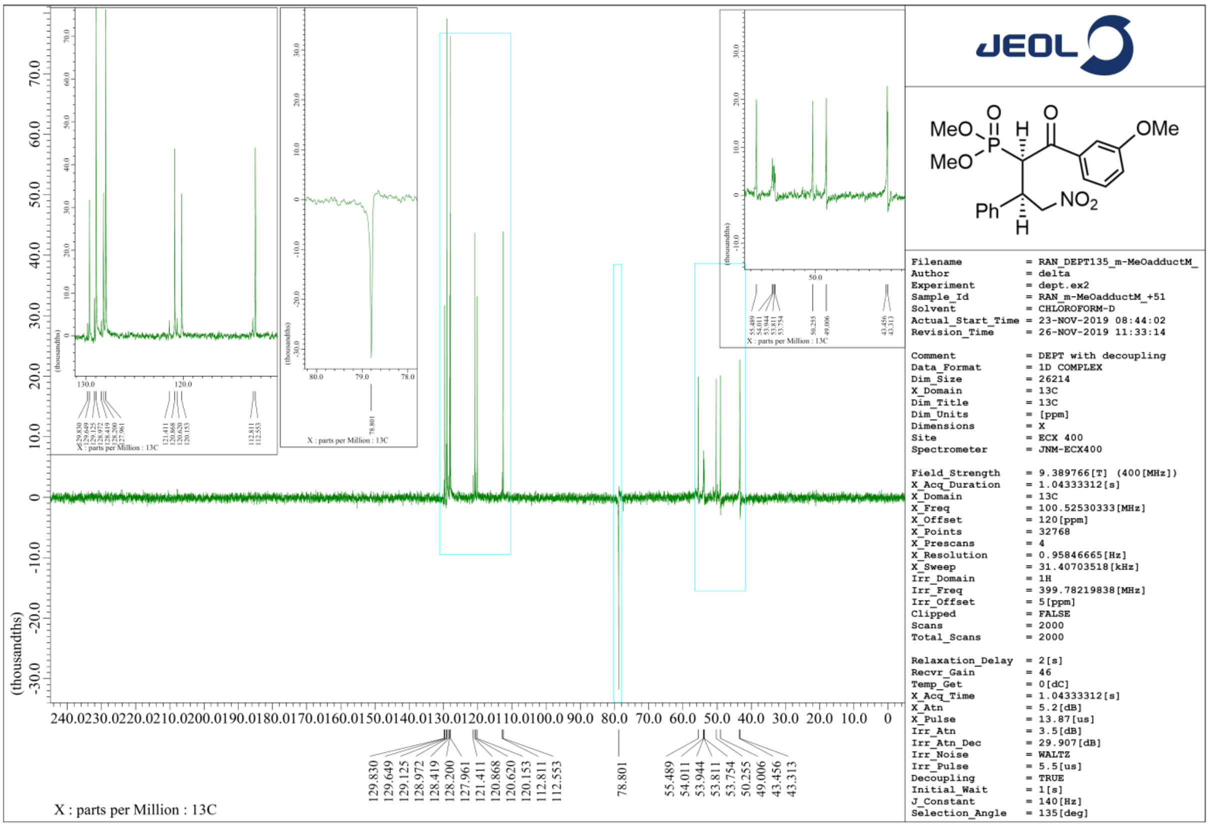

^{31}P NMR spectra of dimethyl [(2*R*,3*R*)-1-(3-methoxyphenyl)-4-nitro-1-oxo-3-phenylbutan-2-yl]phosphonate (**6c**) in $CDCl_3$

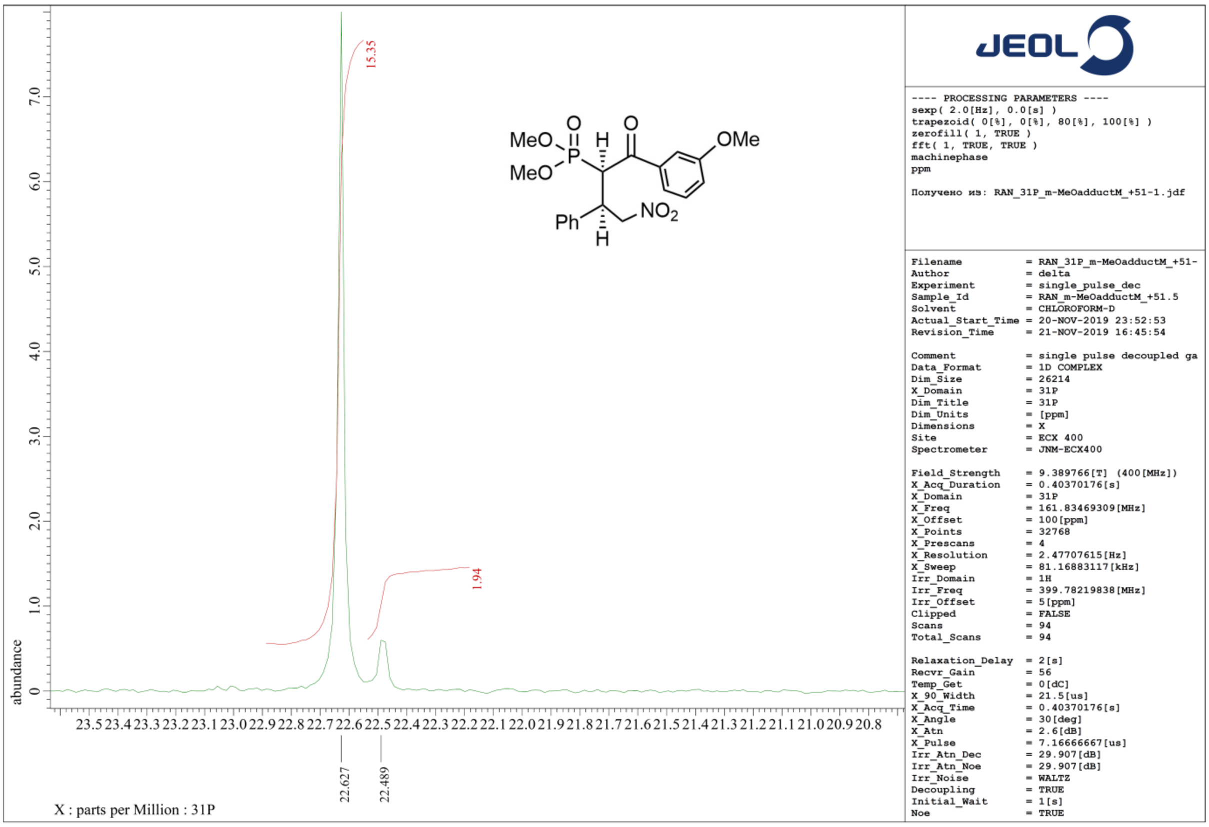

FTIR spectra of dimethyl [(2*S*,3*S*)-1-(2-methoxyphenyl)-4-nitro-1-oxo-3-phenylbutan-2-yl]phosphonate

(**6d**)

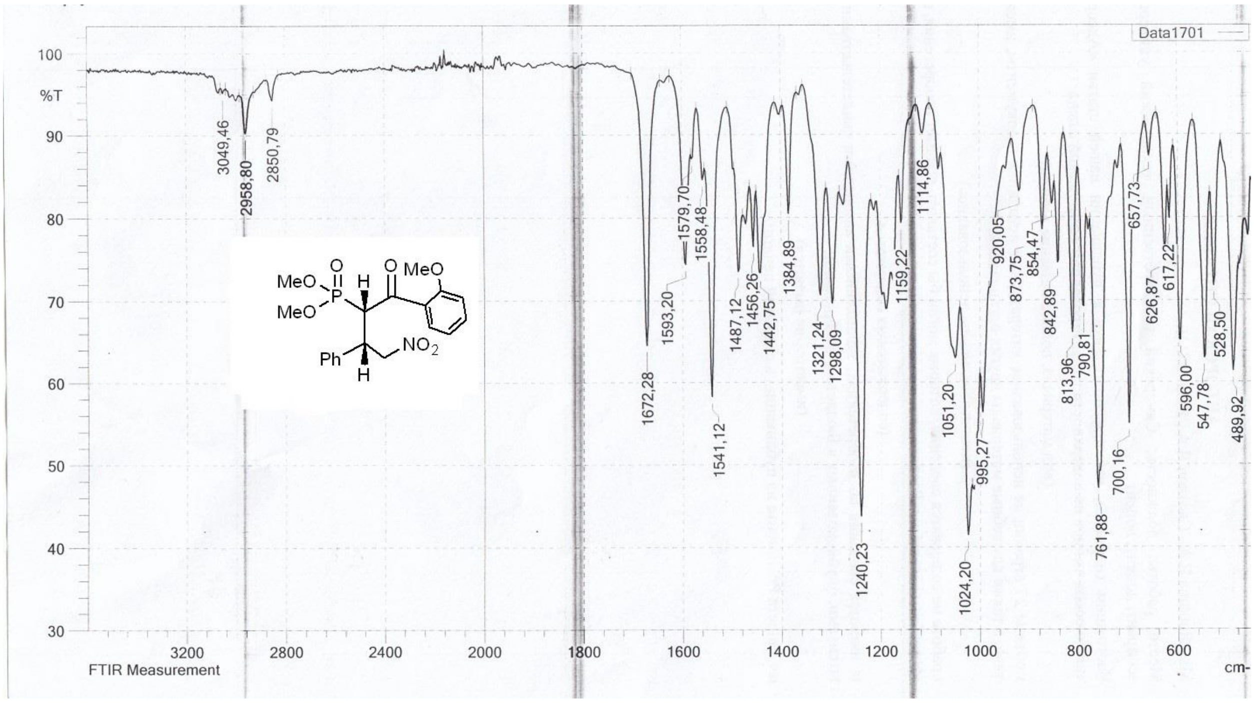

^{1}H NMR spectra of dimethyl [(2*S*,3*S*)-1-(2-methoxyphenyl)-4-nitro-1-oxo-3-phenylbutan-2-yl]phosphonate

(**6d**) in $CDCl_3$

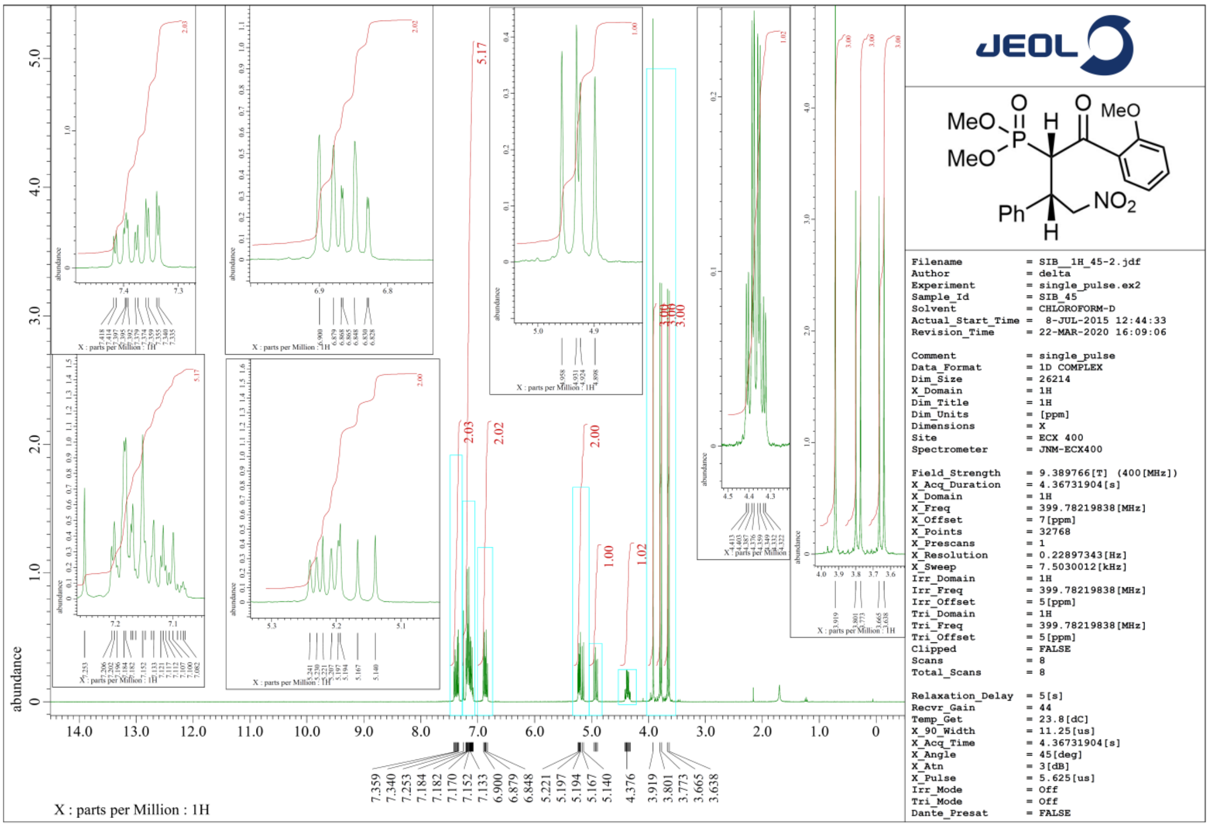

^{13}C NMR spectra of dimethyl [(2*S*,3*S*)-1-(2-methoxyphenyl)-4-nitro-1-oxo-3-phenylbutan-2-yl]phosphonate (**6d**) in $CDCl_3$

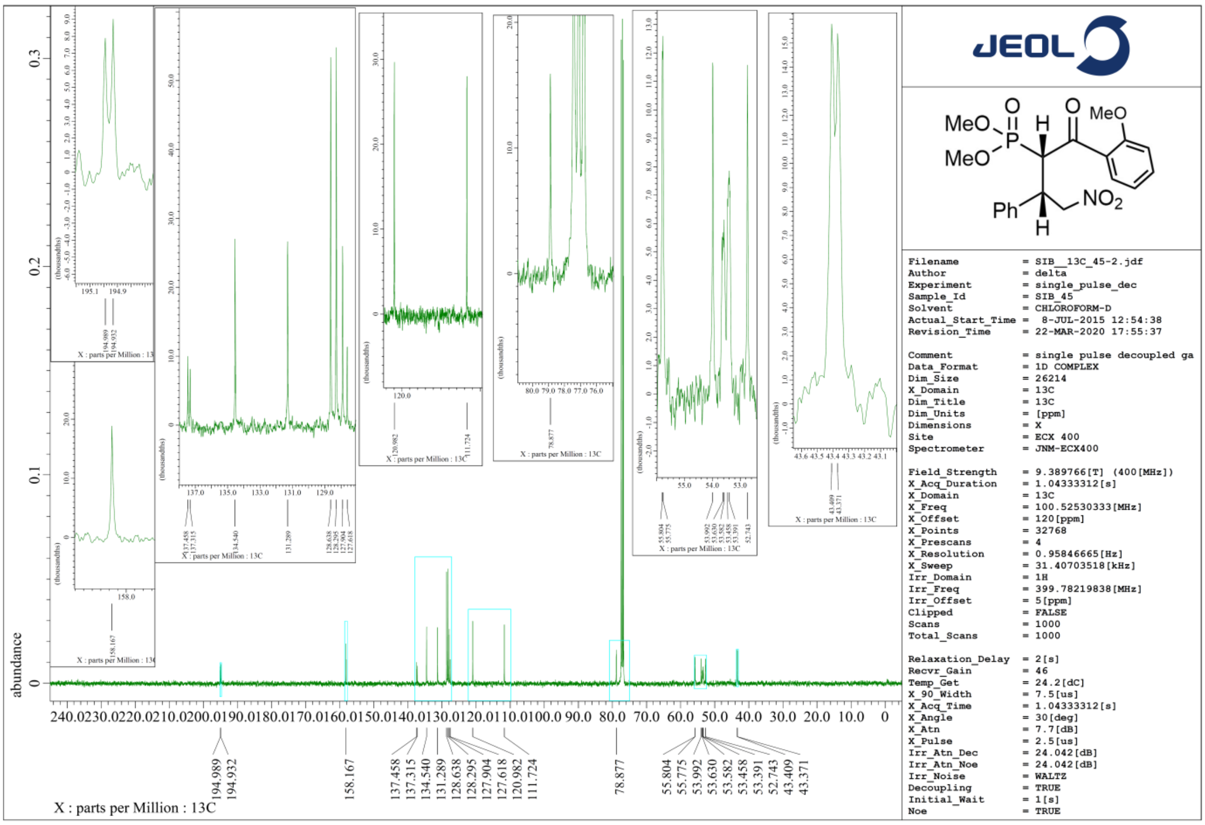

^{31}P NMR spectra of dimethyl [(2*S*,3*S*)-1-(2-methoxyphenyl)-4-nitro-1-oxo-3-phenylbutan-2-yl]phosphonate (**6d**) in $CDCl_3$

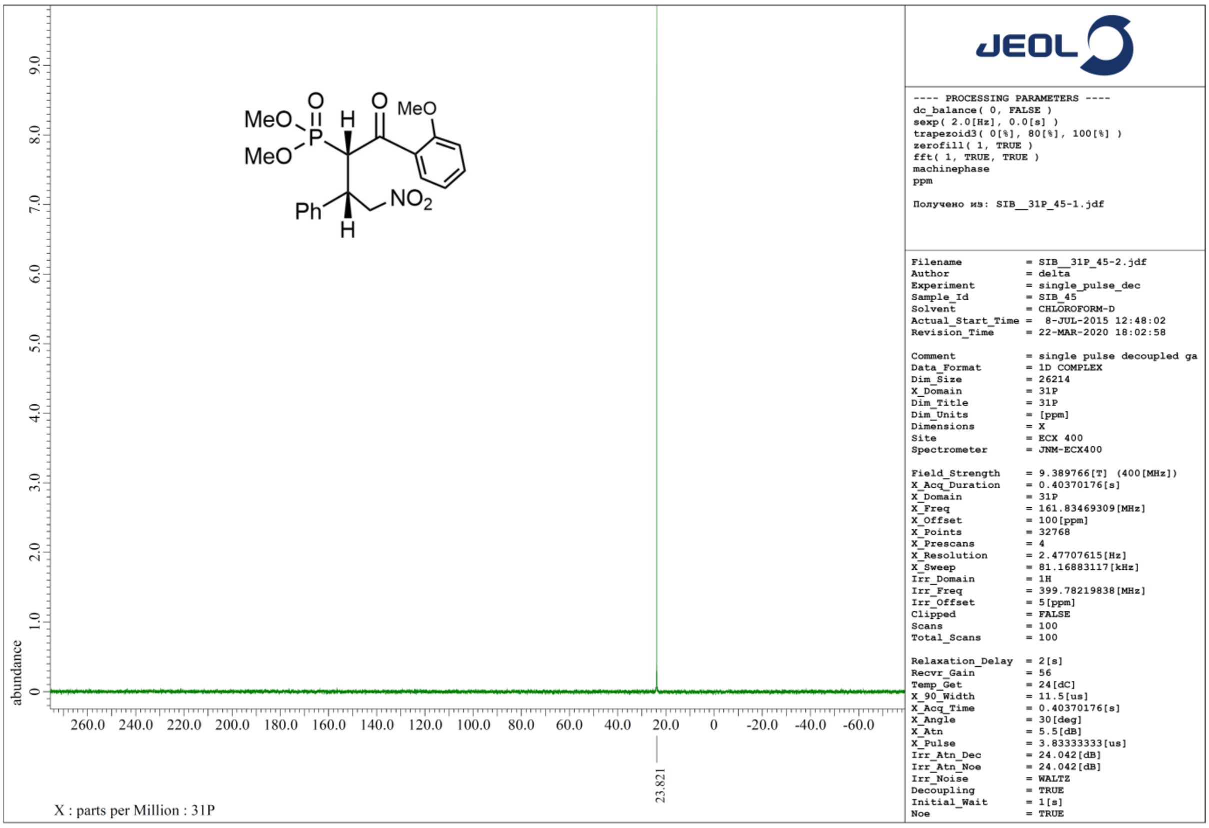

HRMS of dimethyl [(2*S*,3*S*)-1-(2-methoxyphenyl)-4-nitro-1-oxo-3-phenylbutan-2-yl]phosphonate (**6d**)

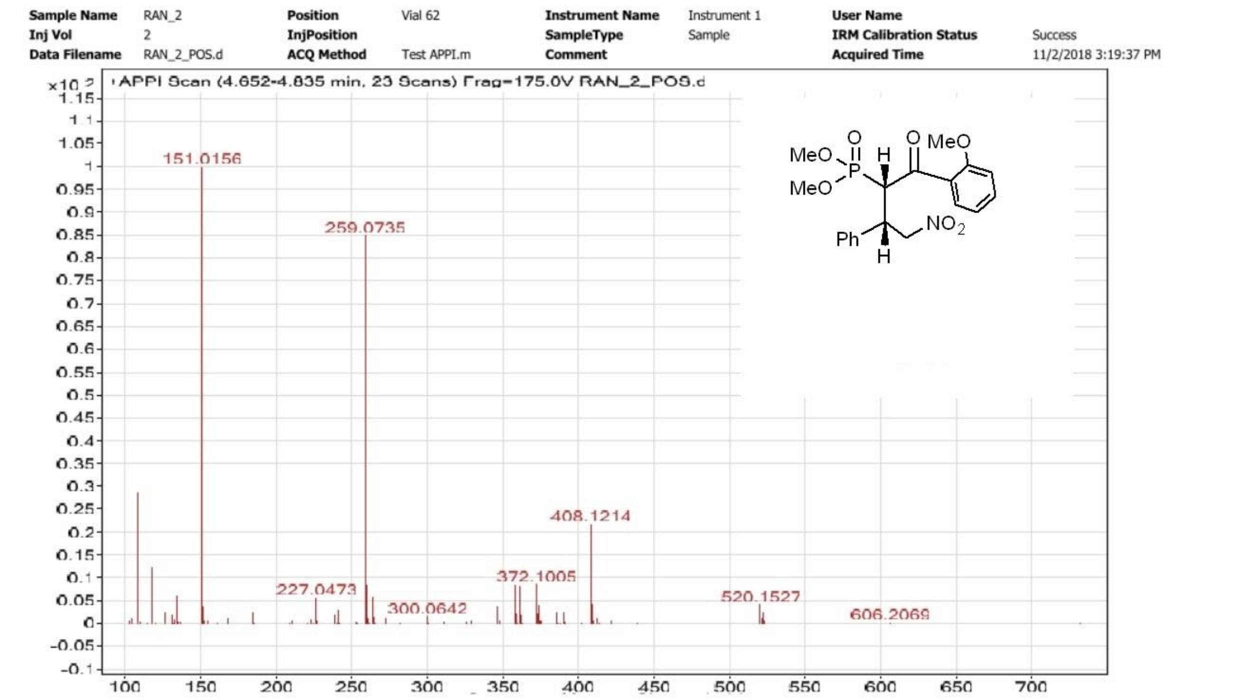

FTIR spectra of dimethyl [(2*S*,3*R*)-1-nitro-4-oxo-2-phenylpentan-3-yl]phosphonate (**6e**)

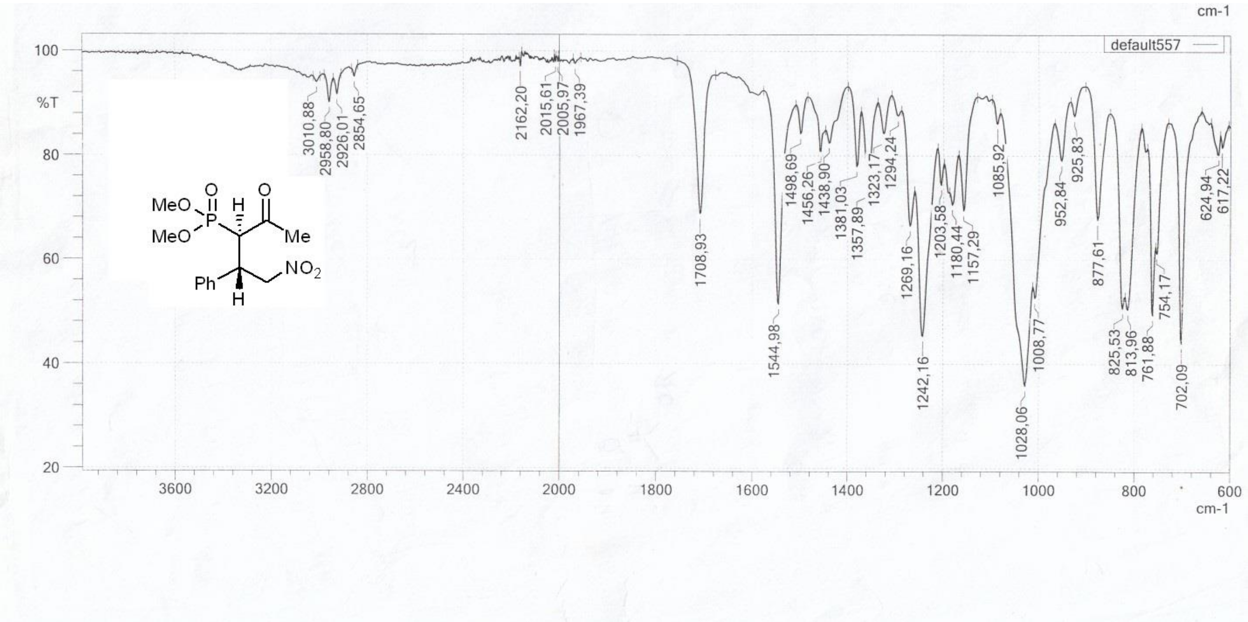

^{1}H NMR spectra of dimethyl [(2*S*,3*R*)-1-nitro-4-oxo-2-phenylpentan-3-yl]phosphonate (**6e**) in $CDCl_3$

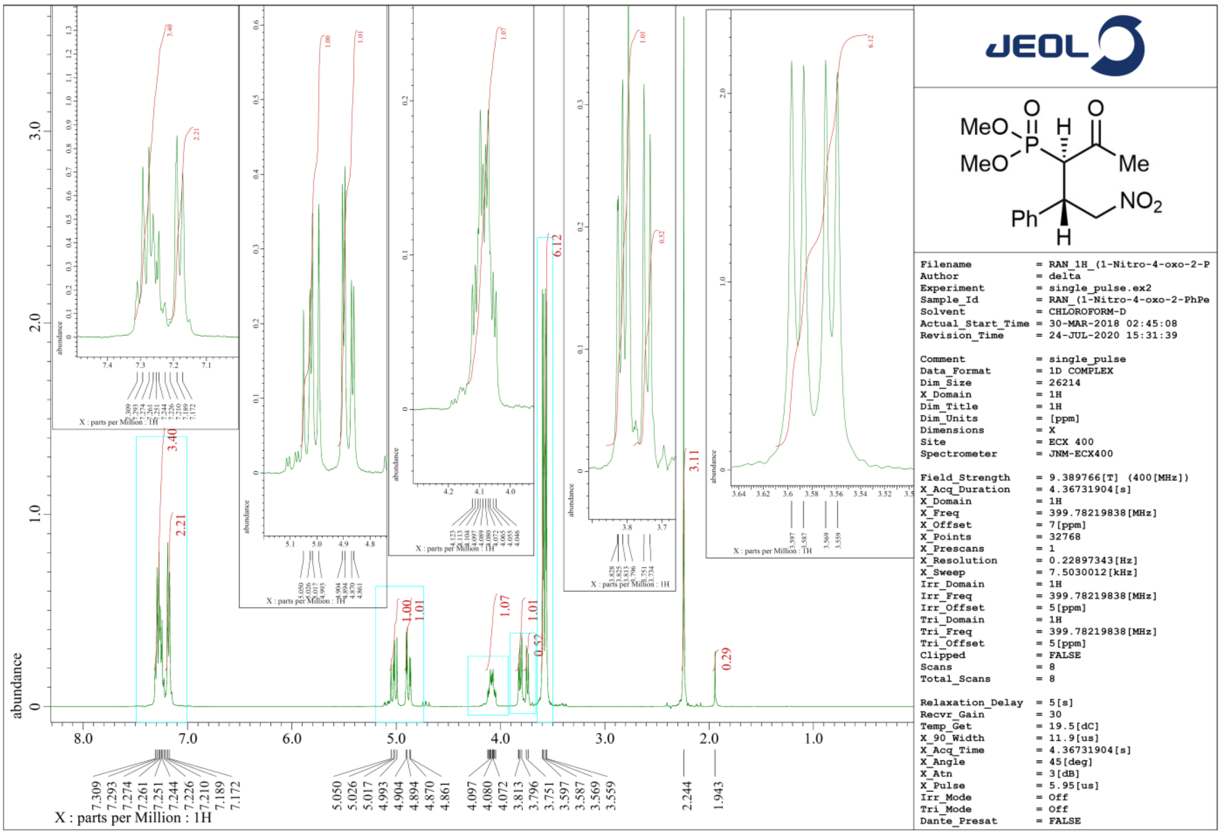

^{13}C NMR spectra of dimethyl [(2*S*,3*R*)-1-nitro-4-oxo-2-phenylpentan-3-yl]phosphonate (**6e**) in $CDCl_3$

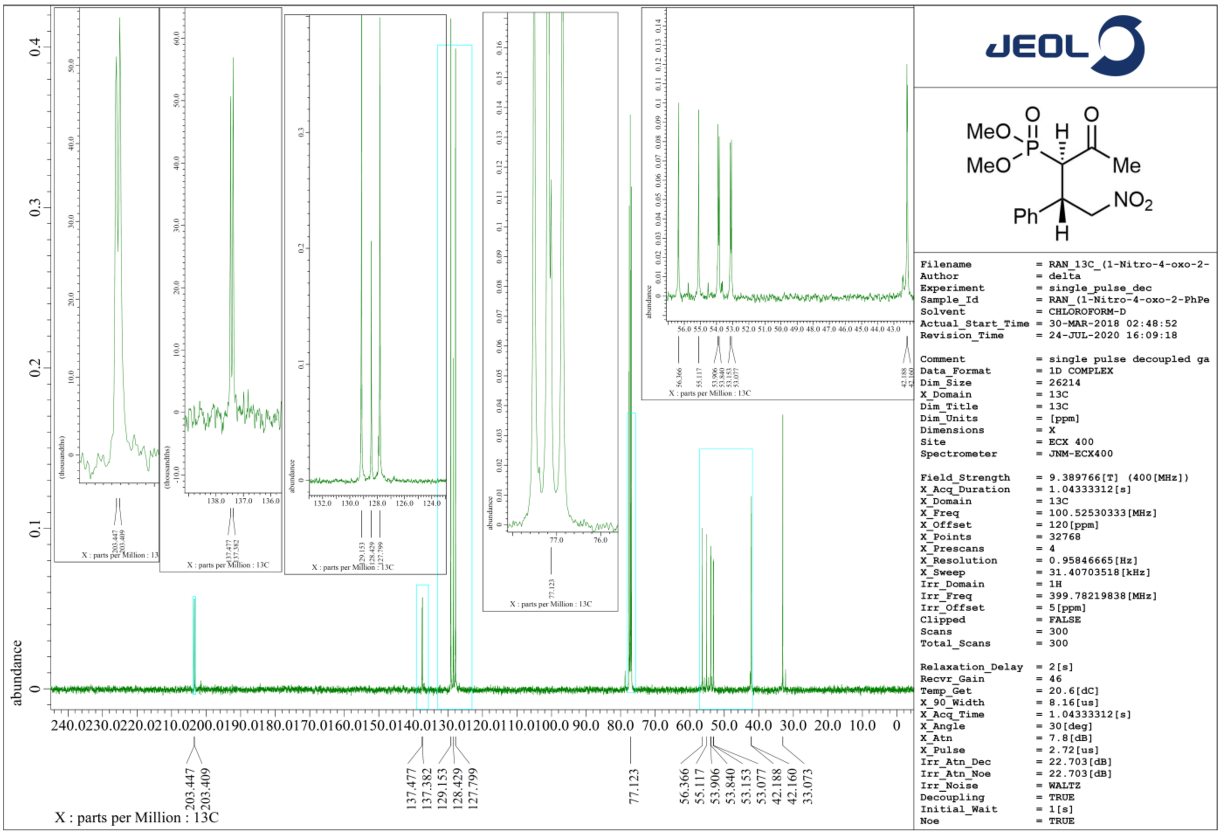

^{31}P NMR spectra of dimethyl [(2*S*,3*R*)-1-nitro-4-oxo-2-phenylpentan-3-yl]phosphonate (**6e**) in $CDCl_3$

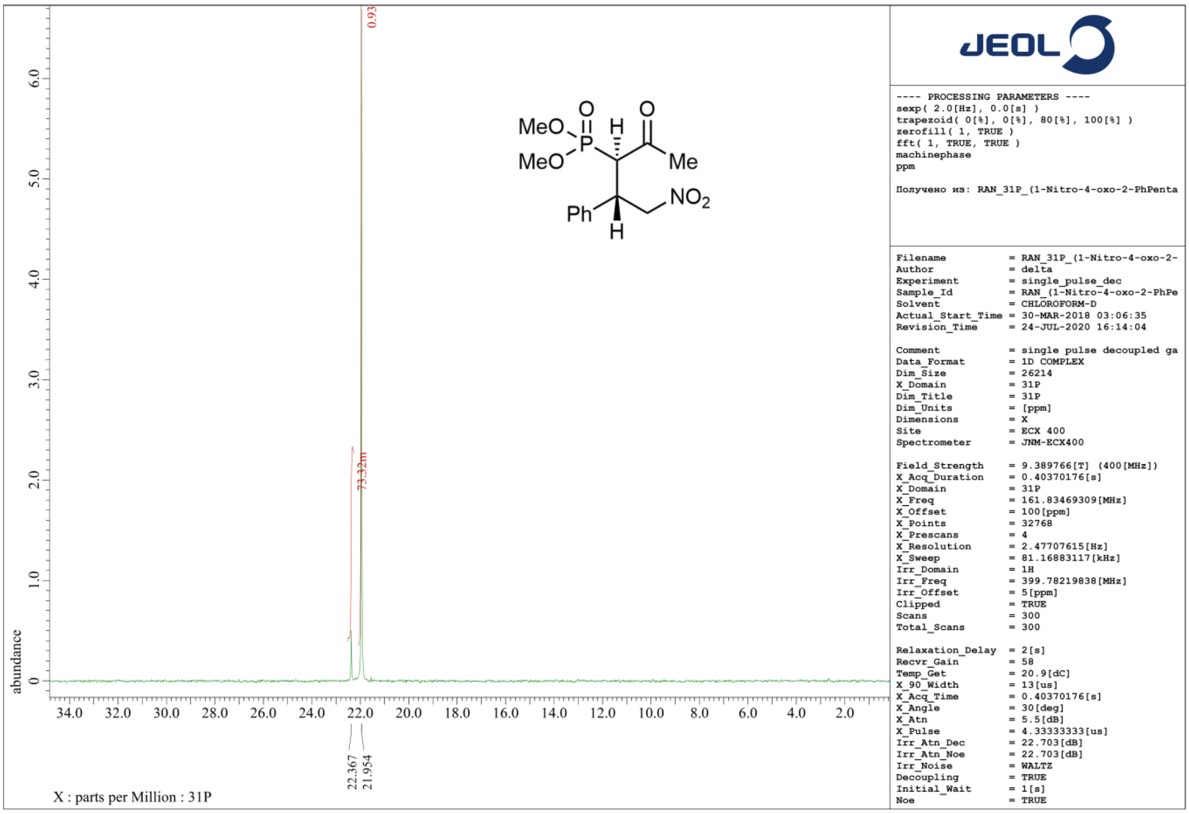

HRMS of dimethyl [(2*S*,3*R*)-1-nitro-4-oxo-2-phenylpentan-3-yl]phosphonate (**6e**)

**Sample Name**	Unavailable	**Position**	Unavailable	**Instrument Name**	Unavailable	**User Name**	Unavailable
**Inj Vol**	Unavailable	**InjPosition**	Unavailable	**SampleType**	Unavailable	**IRM Calibration Status**	Success
**Data Filename**	RAN_1_POS.d	**ACQ Method**		**Comment**	Sample information is unavailable	**Acquired Time**	Unavailable

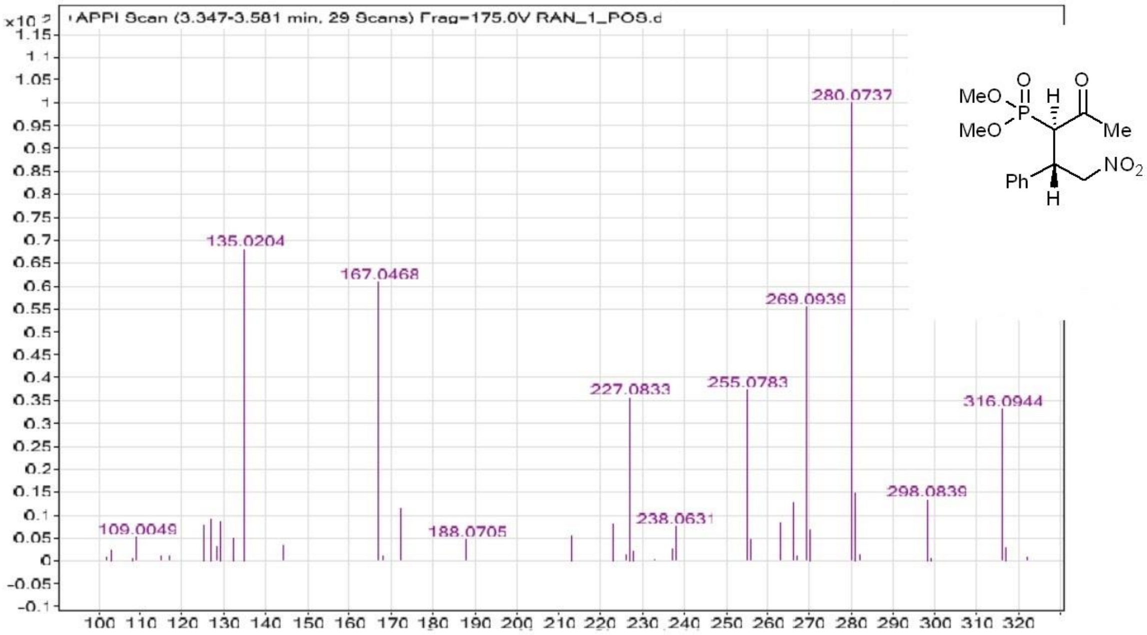

FTIR spectra of dimethyl [(2*S*,3*S*)-1-(adamantan-1-yl)-4-nitro-1-oxo-3-phenylbutan-2-yl]phosphonate (**6f**)

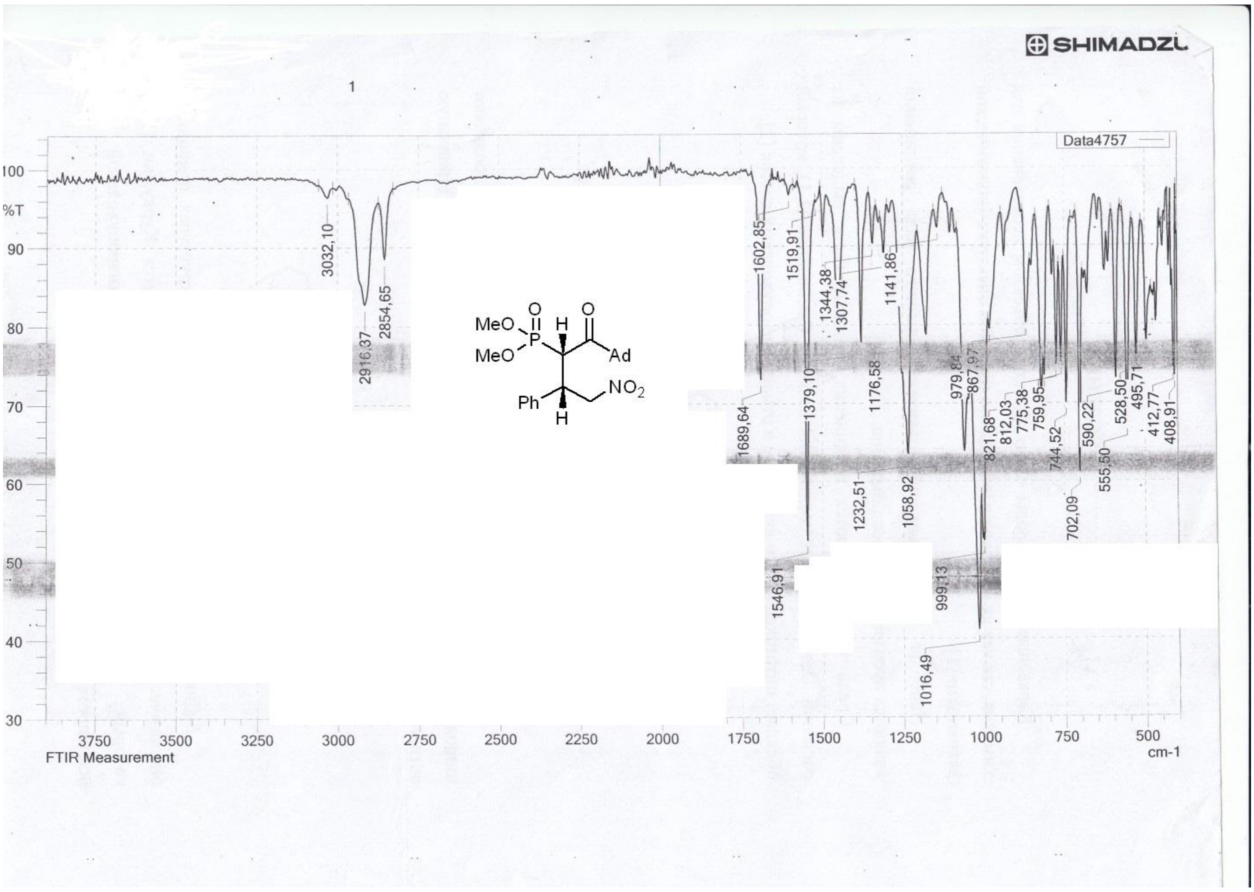

^{1}H NMR spectra of dimethyl [(2*S*,3*S*)-1-(adamantan-1-yl)-4-nitro-1-oxo-3-phenylbutan-2-yl]phosphonate (**6f**) in $CDCl_3$

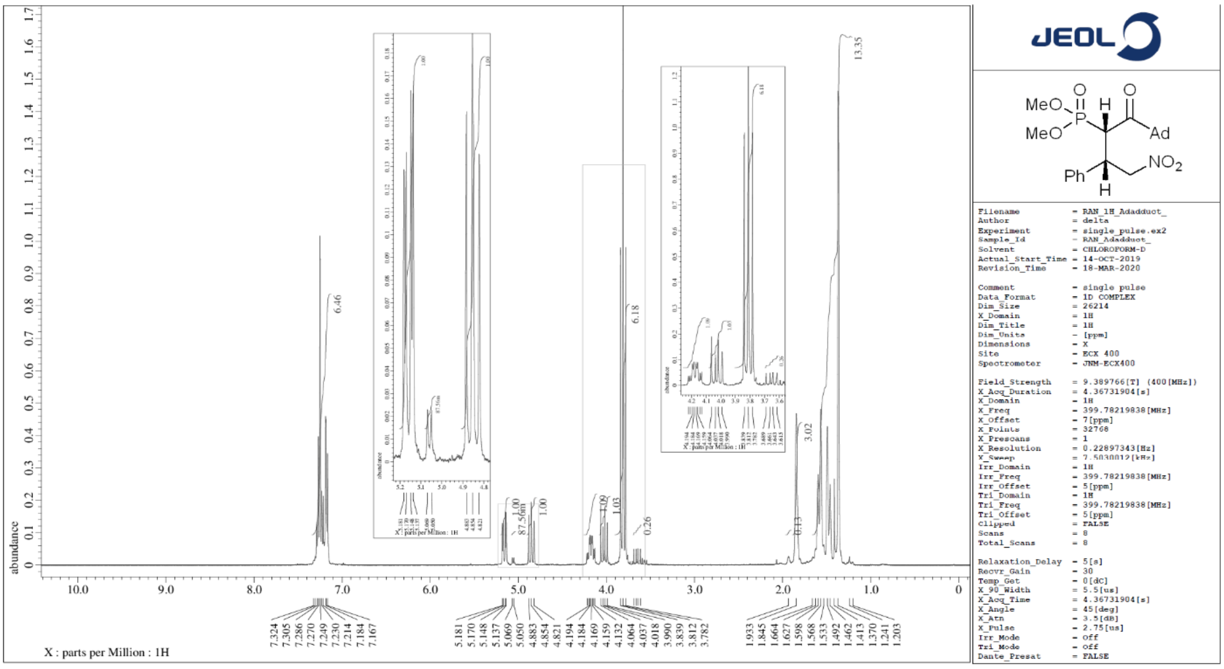

^{13}C NMR spectra of dimethyl [(2*S*,3*S*)-1-(adamantan-1-yl)-4-nitro-1-oxo-3-phenylbutan-2-yl]phosphonate (**6f**) in $CDCl_3$

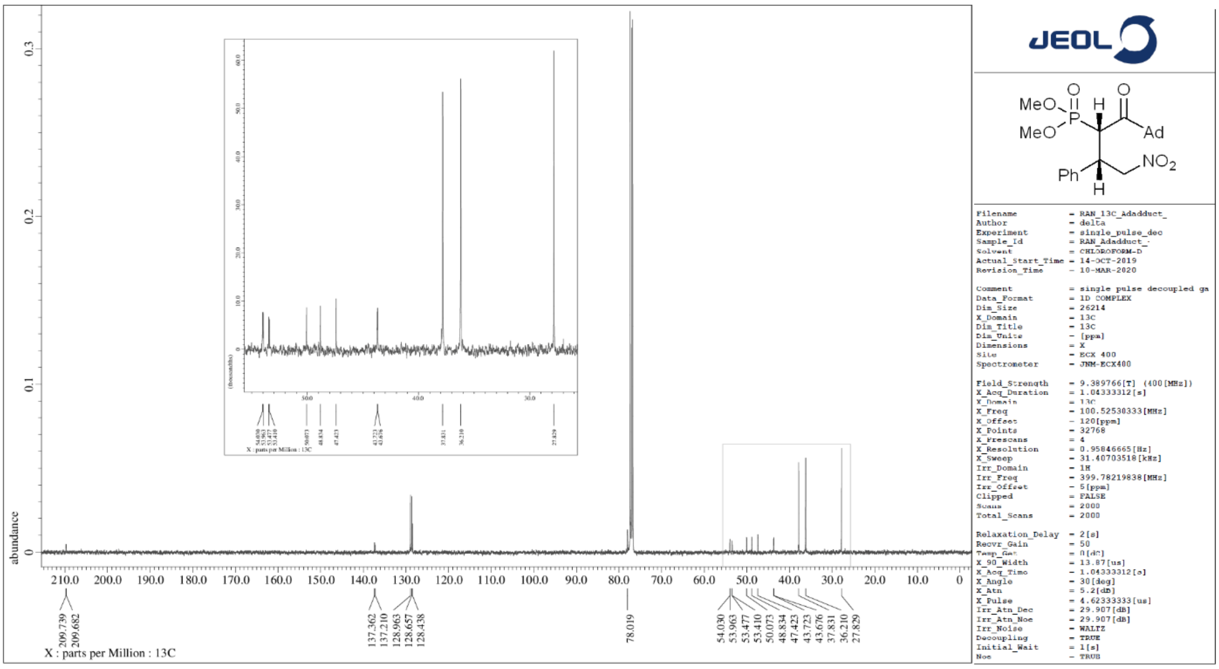

^{31}P NMR spectra of dimethyl [(2*S*,3*S*)-1-(adamantan-1-yl)-4-nitro-1-oxo-3-phenylbutan-2-yl]phosphonate (**6f**) in $CDCl_3$

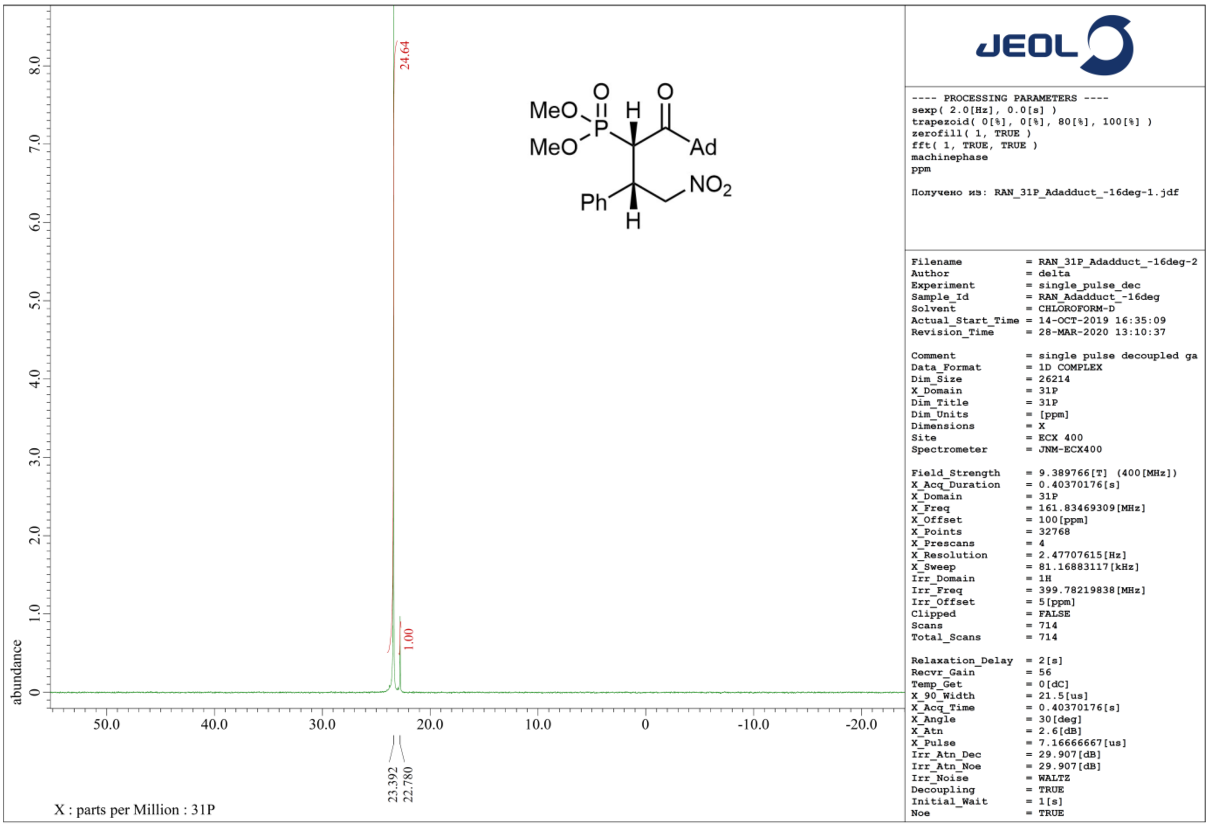

HRMS of dimethyl [(2*S*,3*S*)-1-(adamantan-1-yl)-4-nitro-1-oxo-3-phenylbutan-2-yl]phosphonate (**6f**)

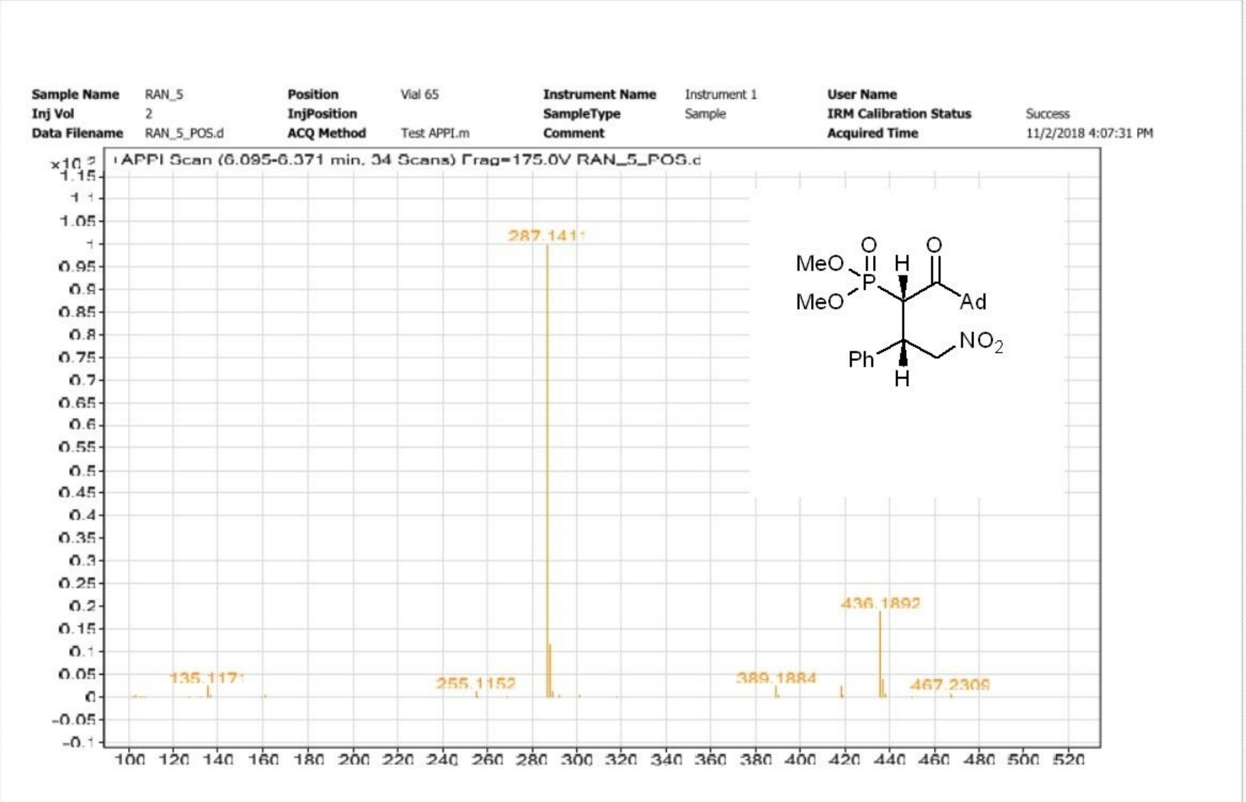

FTIR spectra of dimethyl [(2*R*,3*S*)-1-(adamantan-1-yl)-4-nitro-1-oxo-3-phenylbutan-2-yl]phosphonate (**6f**)

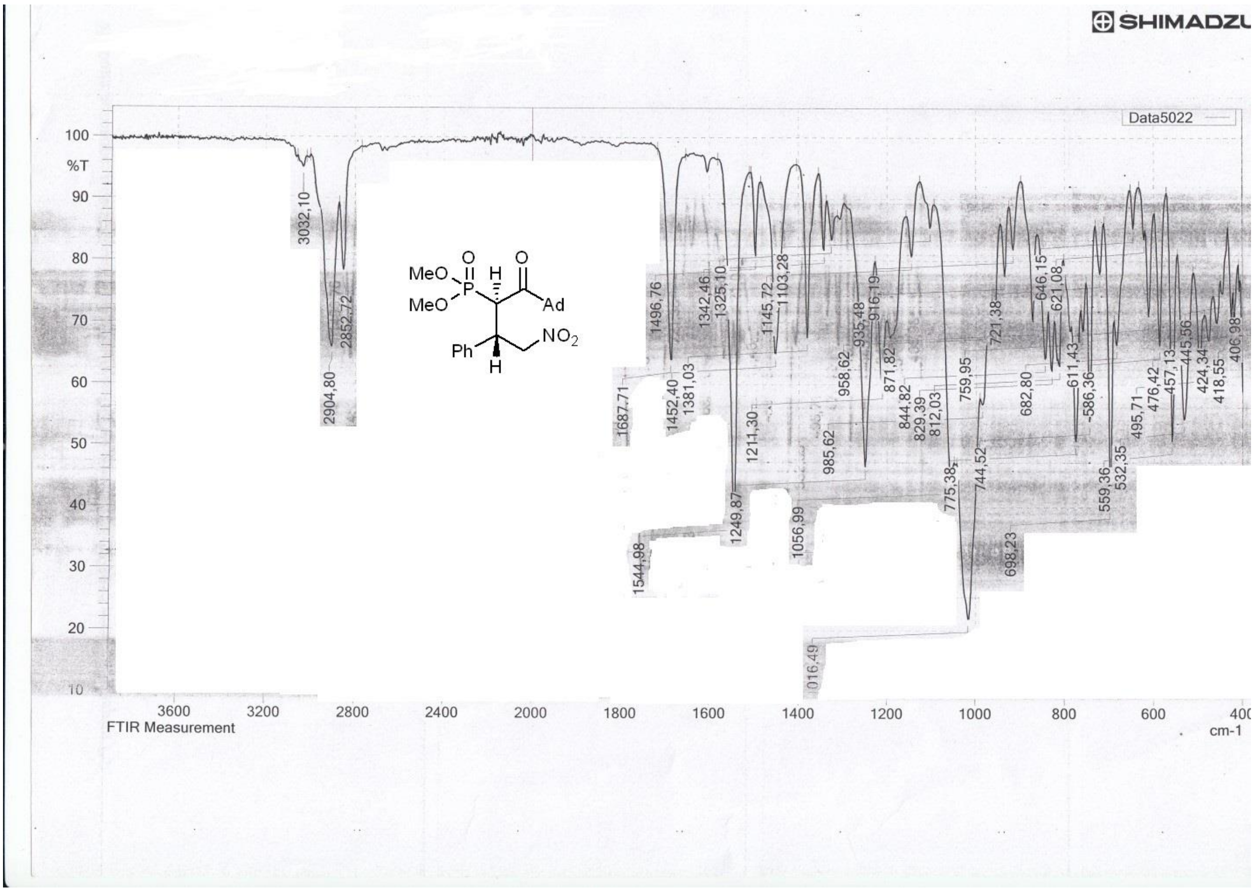

^{1}H NMR spectra of dimethyl [(2*R*,3*S*)-1-(adamantan-1-yl)-4-nitro-1-oxo-3-phenylbutan-2-yl]phosphonate (**6f**) in $CDCl_3$

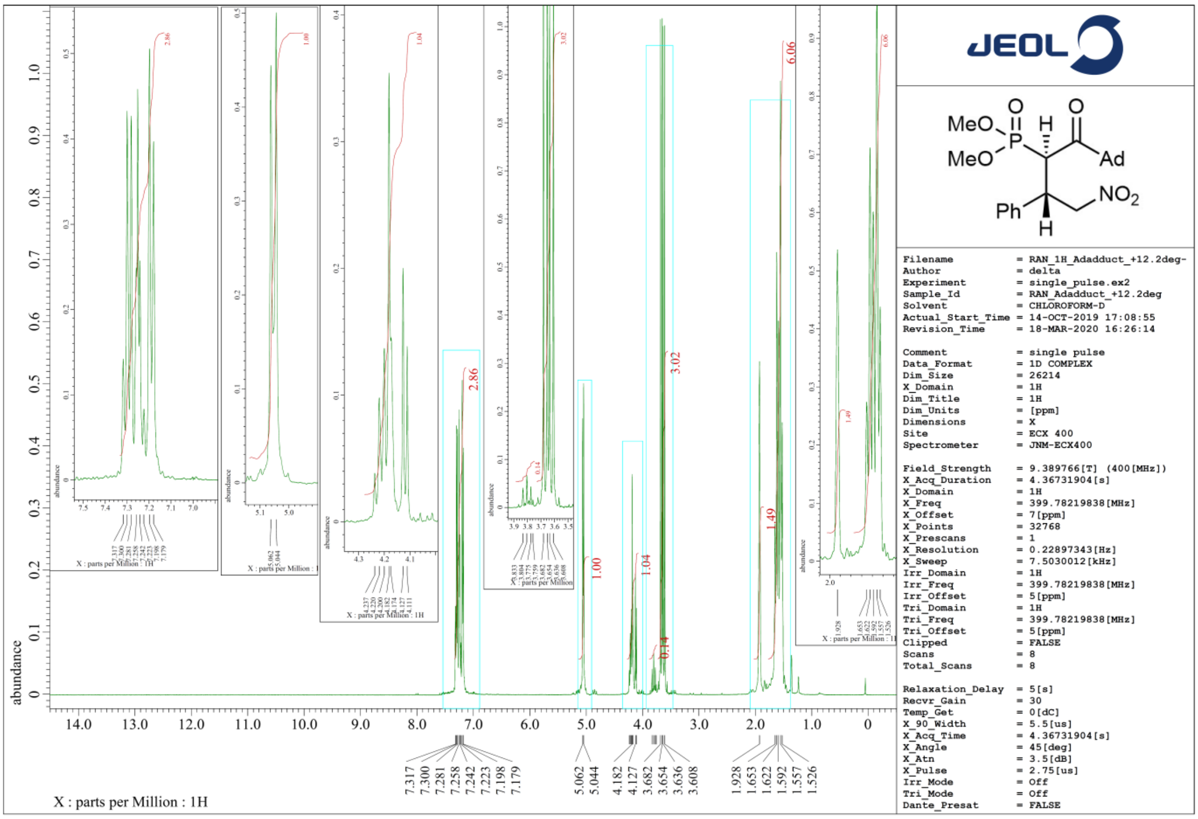

^{13}C NMR spectra of dimethyl [(2*R*,3*S*)-1-(adamantan-1-yl)-4-nitro-1-oxo-3-phenylbutan-2-yl]phosphonate (**6f**) in $CDCl_3$

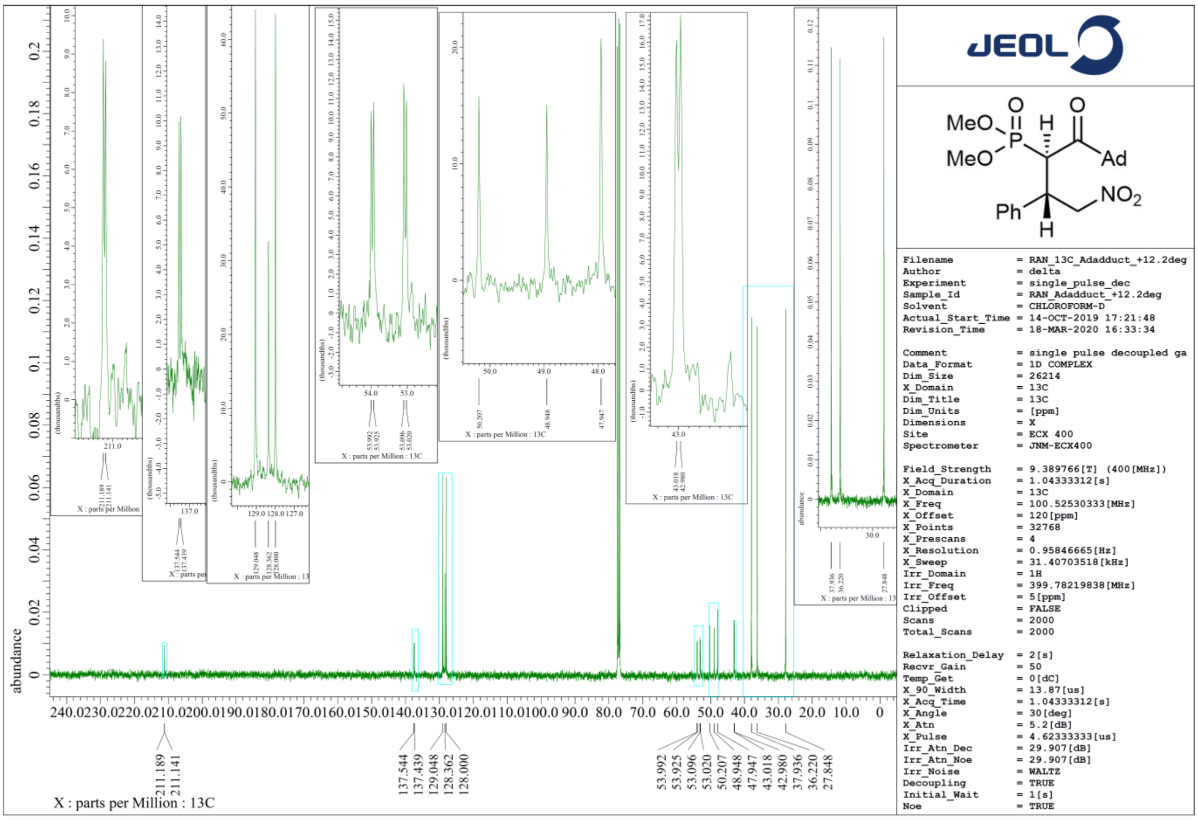

^{31}P NMR spectra of dimethyl [(2*R*,3*S*)-1-(adamantan-1-yl)-4-nitro-1-oxo-3-phenylbutan-2-yl]phosphonate

(**6f**) in $CDCl_3$

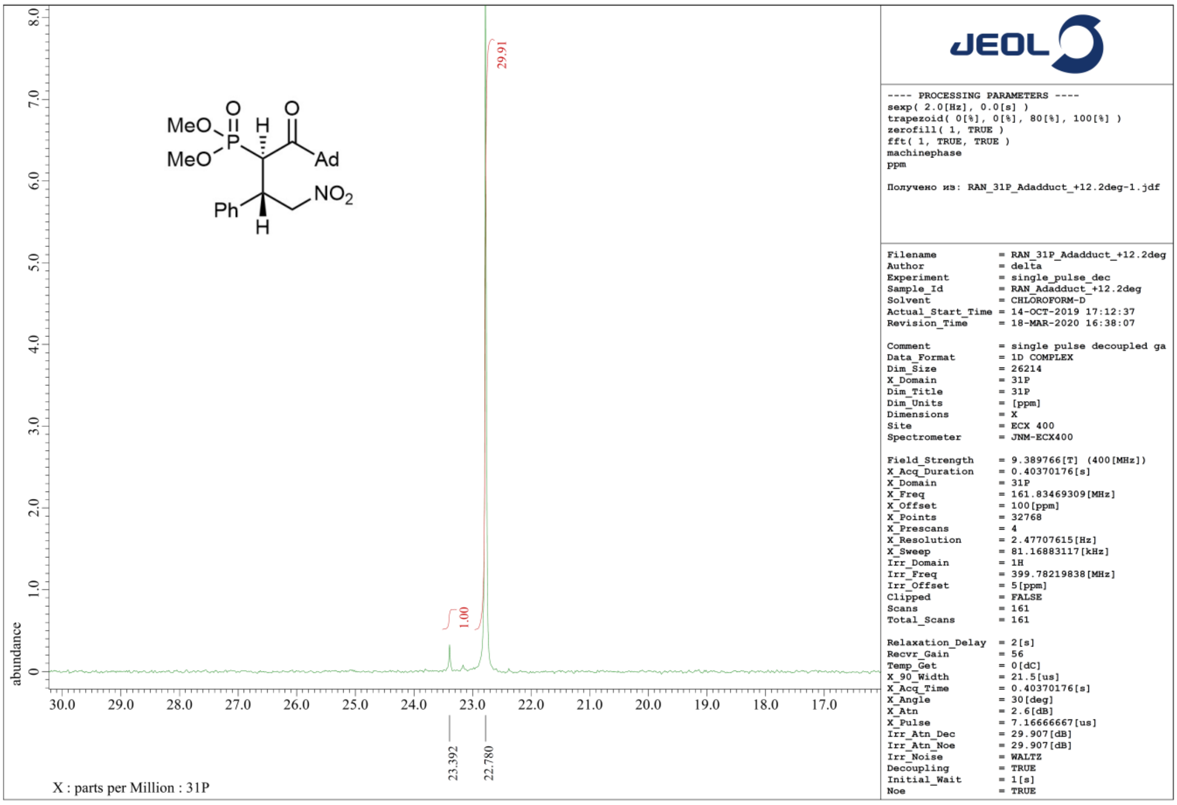

8. Copies of NMR, FTIR and mass spectra for compounds 7-14

^{1}H NMR spectra of dimethyl [(2*R*,3*R*,4*S*)-2,4-diphenylpyrrolidin-3-yl]phosphonate (**7**)

(crude reaction mixture) in $CDCl_3$

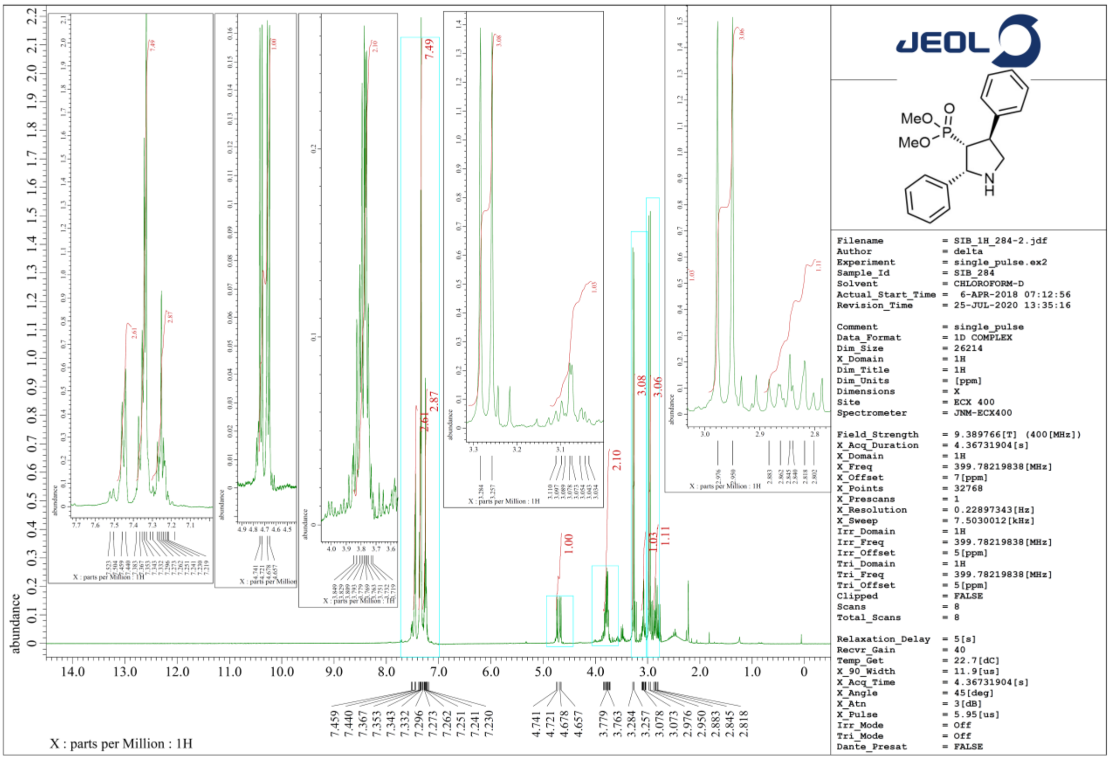

^{13}C NMR spectra of dimethyl [(2*R*,3*R*,4*S*)-2,4-diphenylpyrrolidin-3-yl]phosphonate (**7**)

in $CDCl_3$

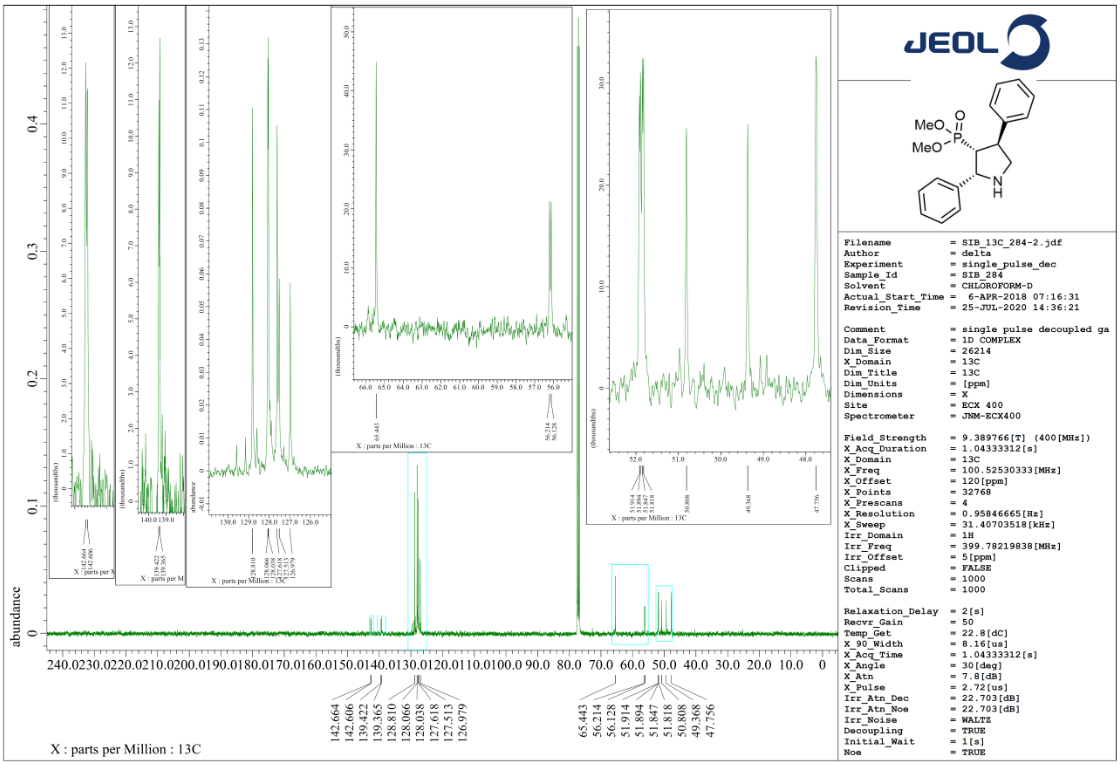

DEPT NMR spectra of dimethyl [(2*R*,3*R*,4*S*)-2,4-diphenylpyrrolidin-3-yl]phosphonate (**7**)

in $CDCl_3$

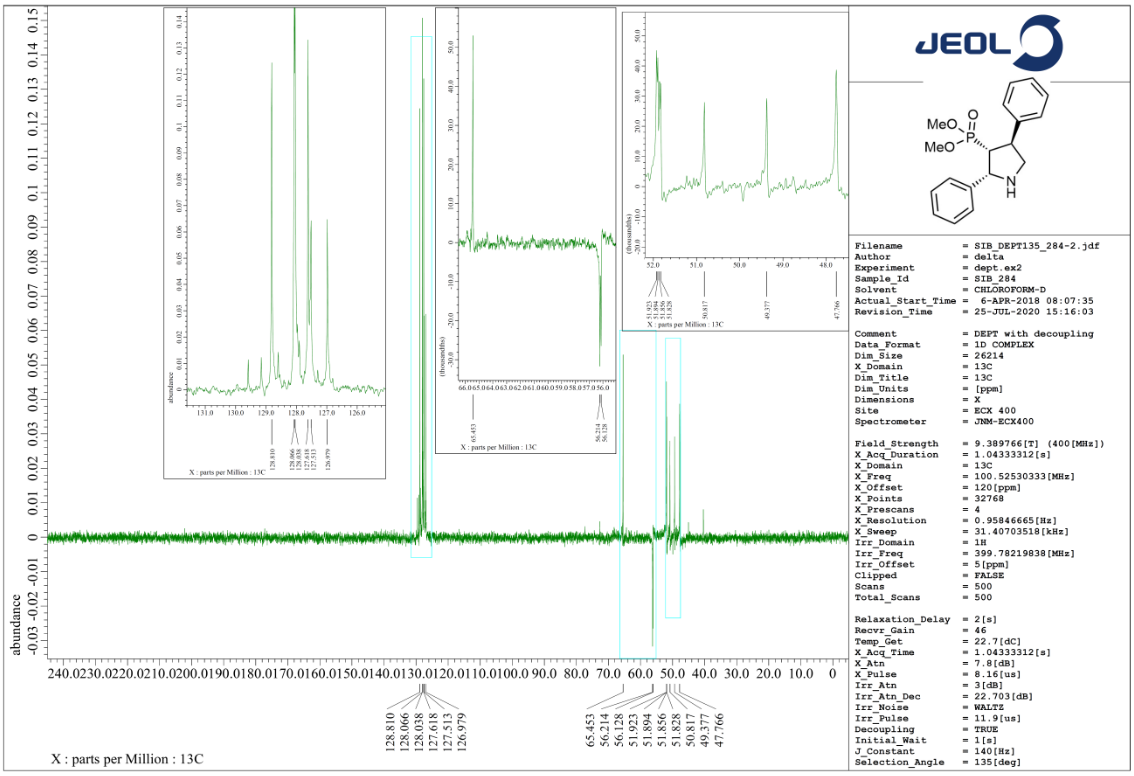

^{31}P NMR spectra of dimethyl [(2*R*,3*R*,4*S*)-2,4-diphenylpyrrolidin-3-yl]phosphonate (**7**)

in $CDCl_3$

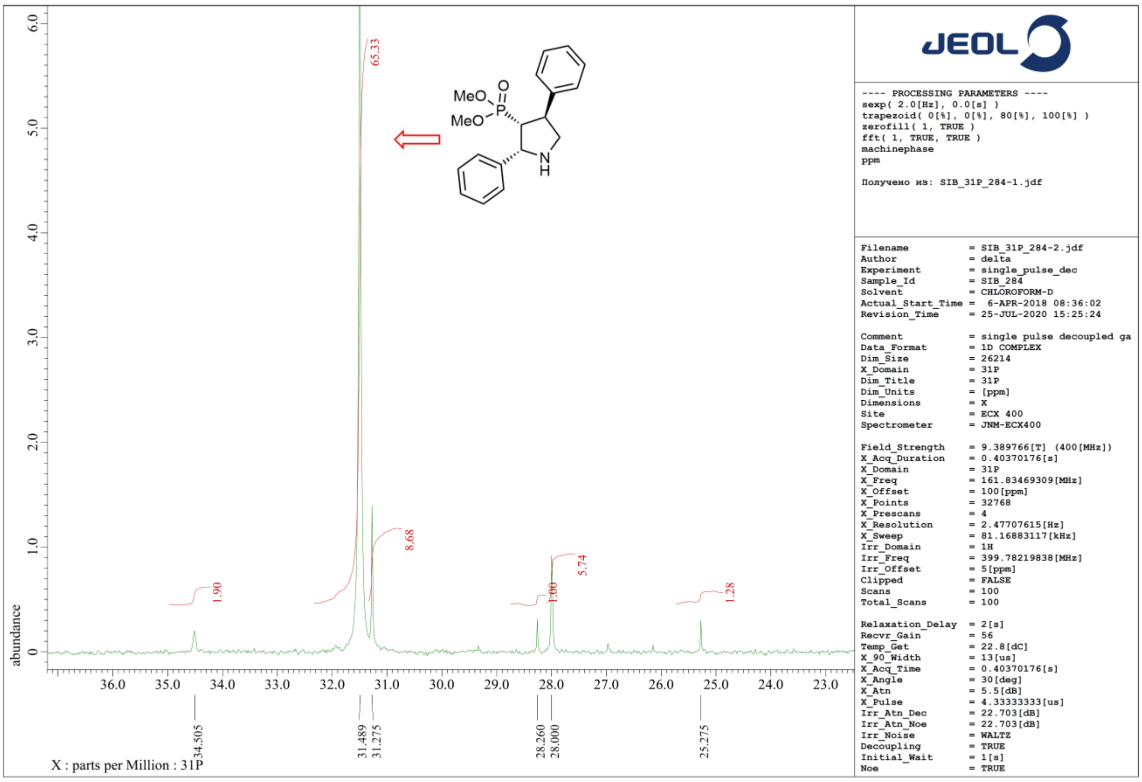

FTIR spectra of dimethyl [(2*R*,3*R*,4*S*)-1-methyl-2,4-diphenylpyrrolidin-3-yl]phosphonate (**8**)

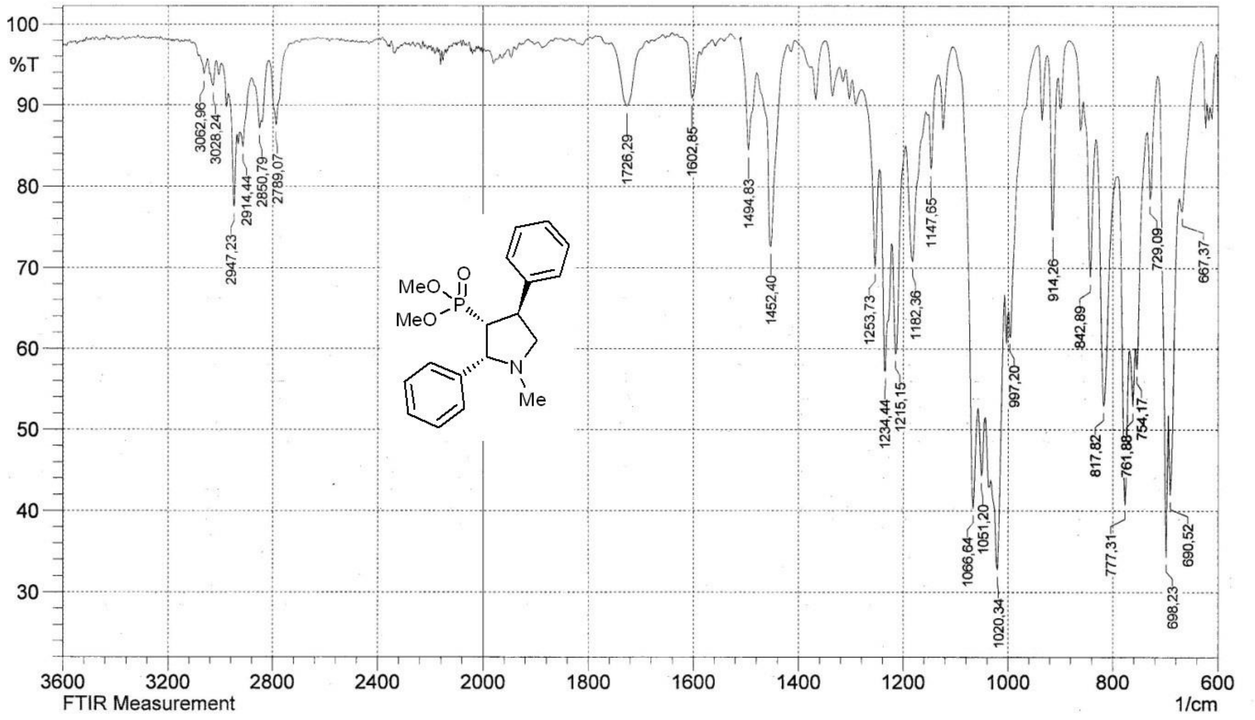

^{1}H NMR spectra of dimethyl [(2*R*,3*R*,4*S*)-1-methyl-2,4-diphenylpyrrolidin-3-yl]phosphonate (**8**)

in DMSO-d6

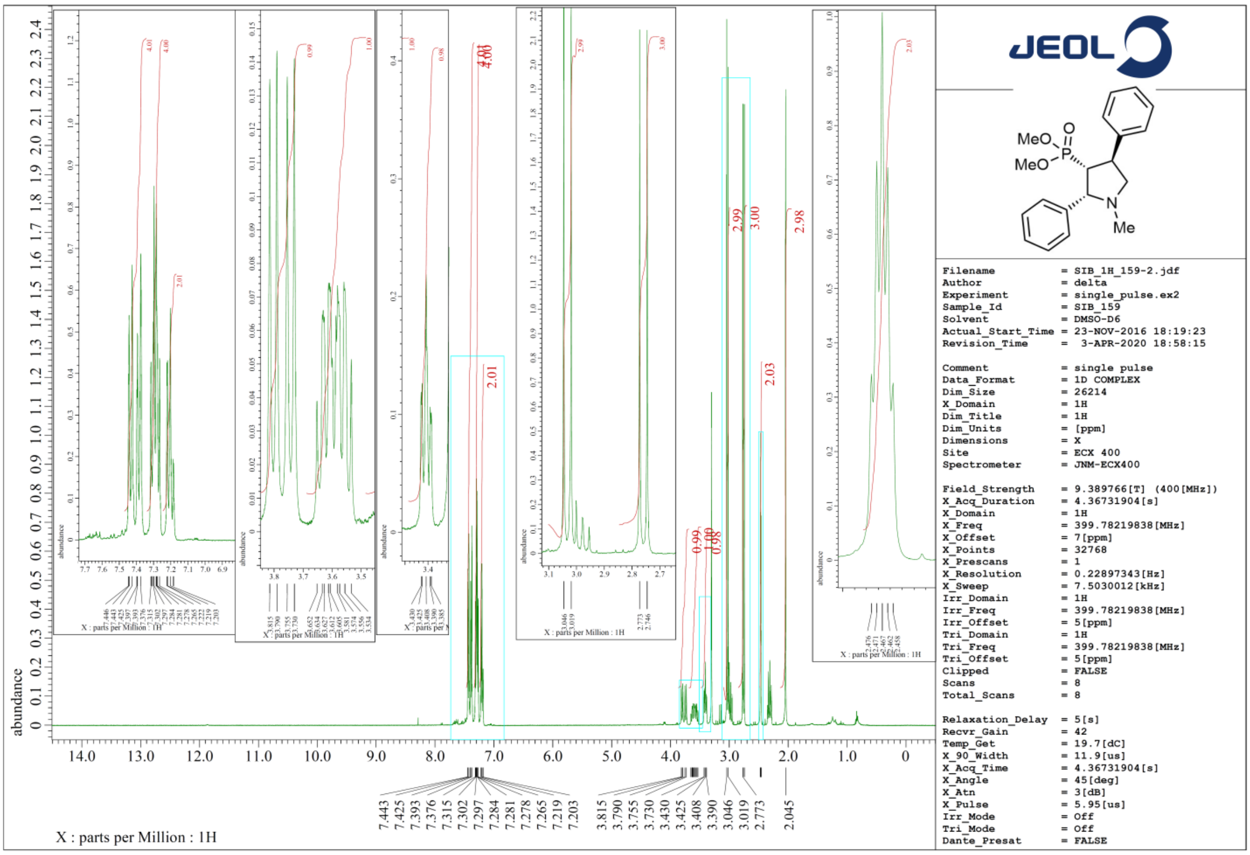

^{13}C NMR spectra of dimethyl [(2*R*,3*R*,4*S*)-1-methyl-2,4-diphenylpyrrolidin-3-yl]phosphonate (**8**)

in DMSO-d6

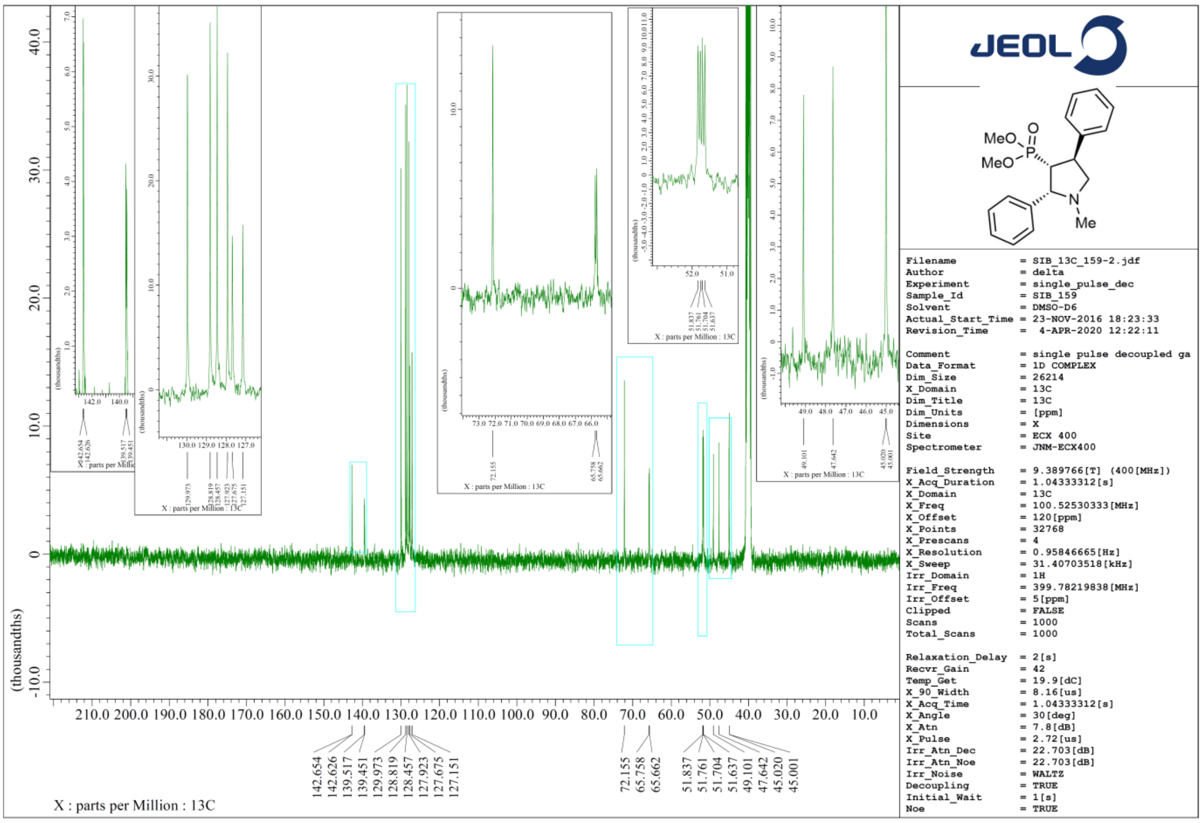

DEPT NMR spectra of dimethyl [(2*R*,3*R*,4*S*)-1-methyl-2,4-diphenylpyrrolidin-3-yl]phosphonate (**8**)

In DMSO-d6

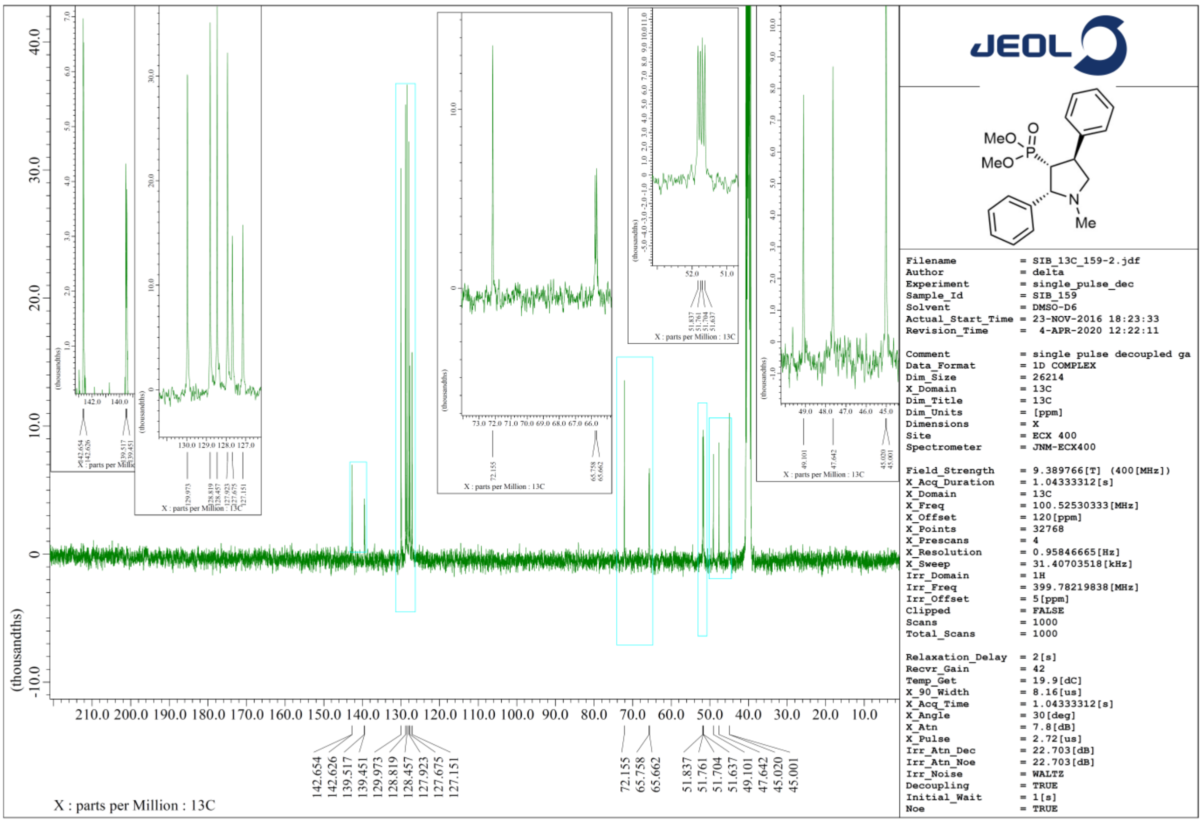

^{31}P NMR spectra of dimethyl [(2*R*,3*R*,4*S*)-1-methyl-2,4-diphenylpyrrolidin-3-yl]phosphonate (**8**) in DMSO-d[6]

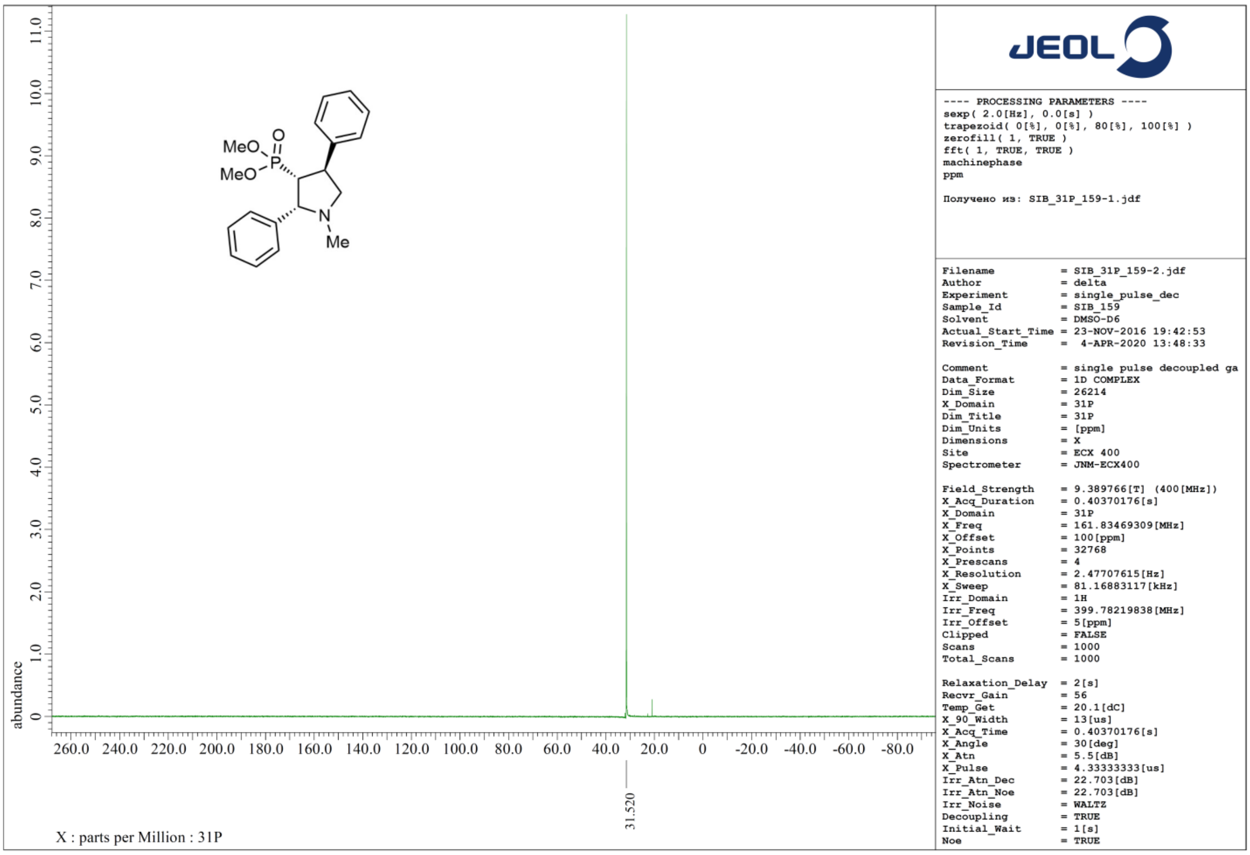

FTIR spectra of methyl hydrogen ((2*R*,3*R*,4*S*)-2,4-diphenylpyrrolidin-3-yl)phosphonate (**9**)

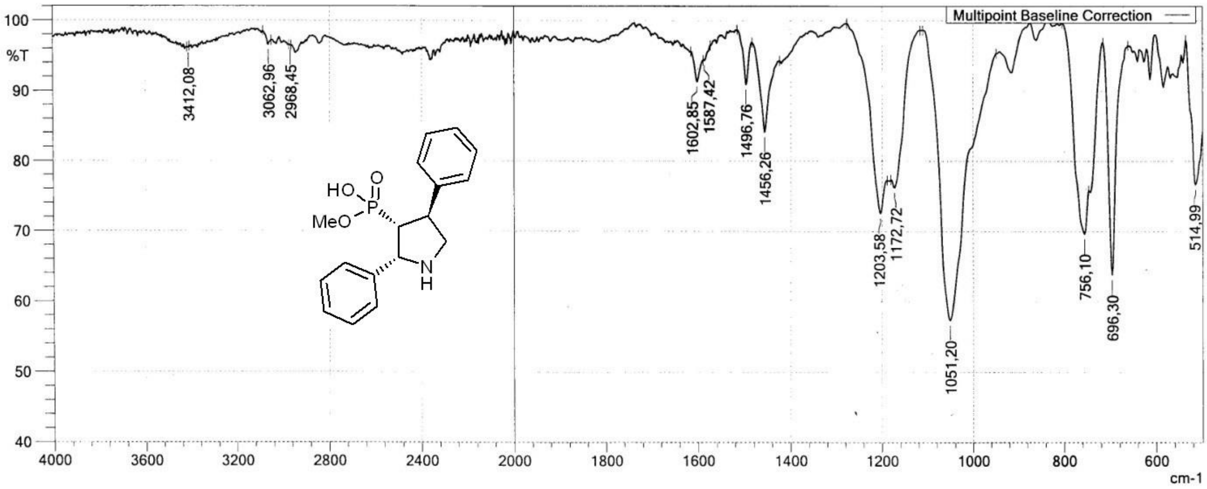

^{1}H NMR spectra of methyl hydrogen ((2*R*,3*R*,4*S*)-2,4-diphenylpyrrolidin-3-yl)phosphonate (**9**) in D_2O

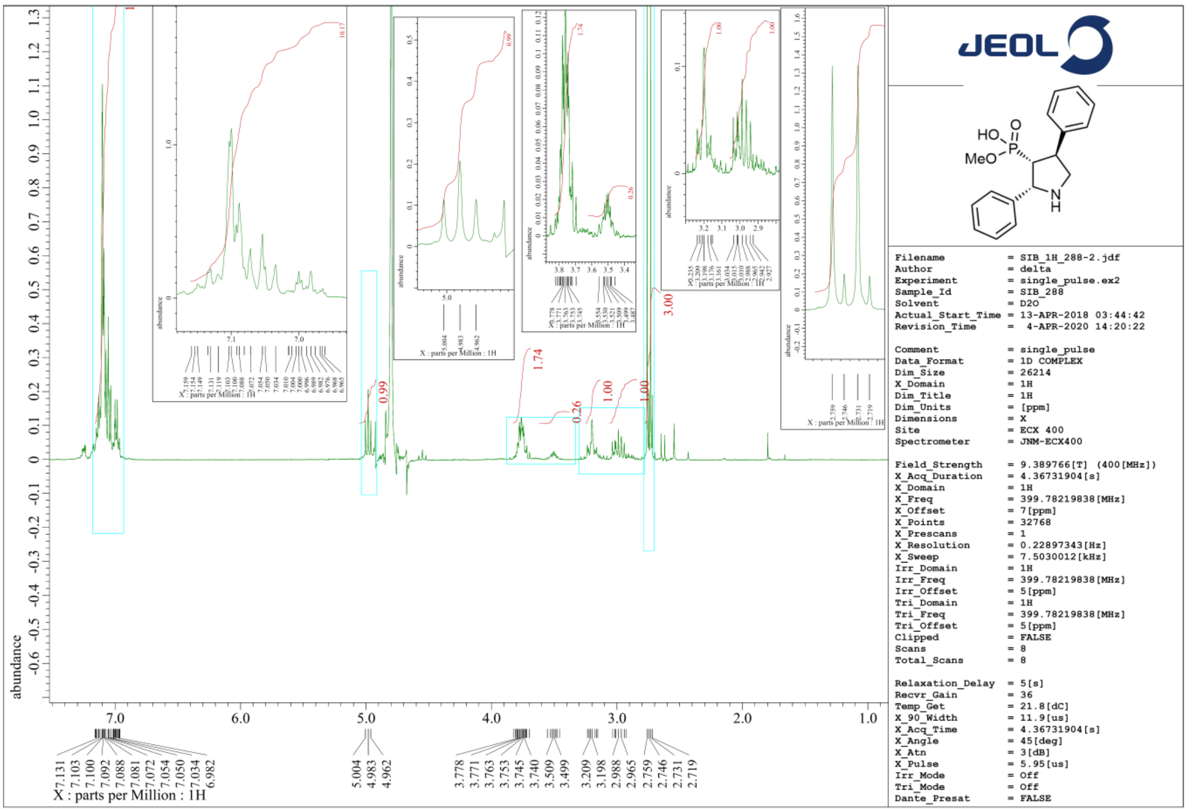

^{13}C NMR spectra of methyl hydrogen ((2*R*,3*R*,4*S*)-2,4-diphenylpyrrolidin-3-yl)phosphonate (**9**) in D_2O

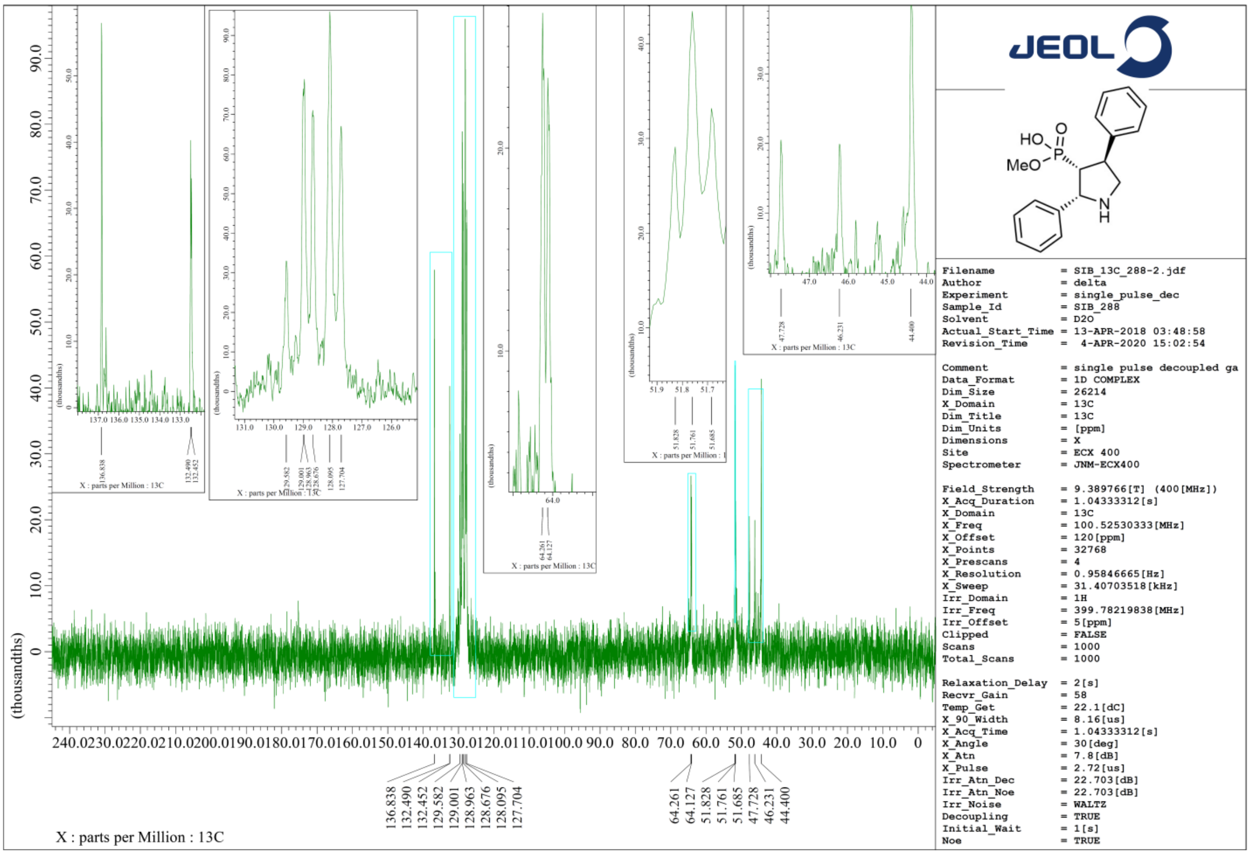

^{31}P NMR spectra of methyl hydrogen ((2*R*,3*R*,4*S*)-2,4-diphenylpyrrolidin-3-yl)phosphonate (**9**) in D_2O

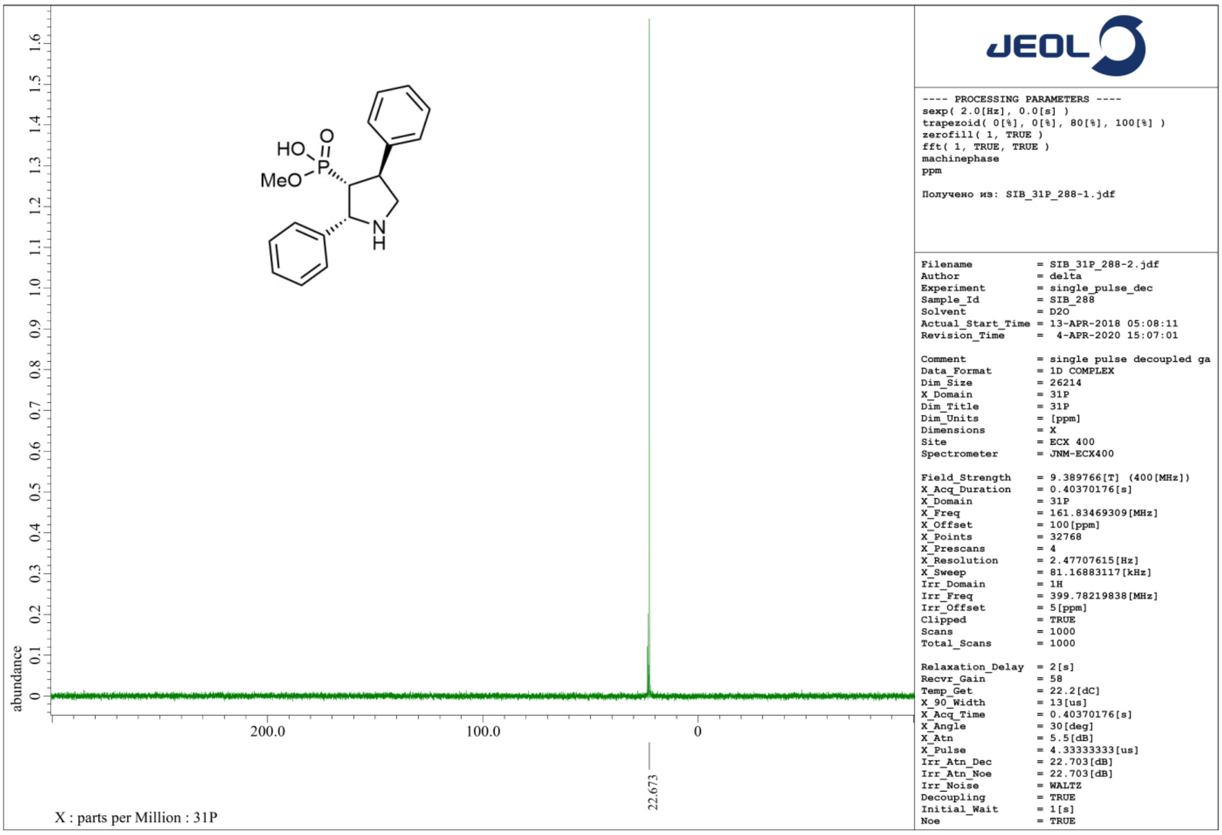

FTIR spectra of dimethyl [(2*R*,3*R*,4*S*)-1-formyl-2,4-diphenylpyrrolidin-3-yl]phosphonate (**10a**)

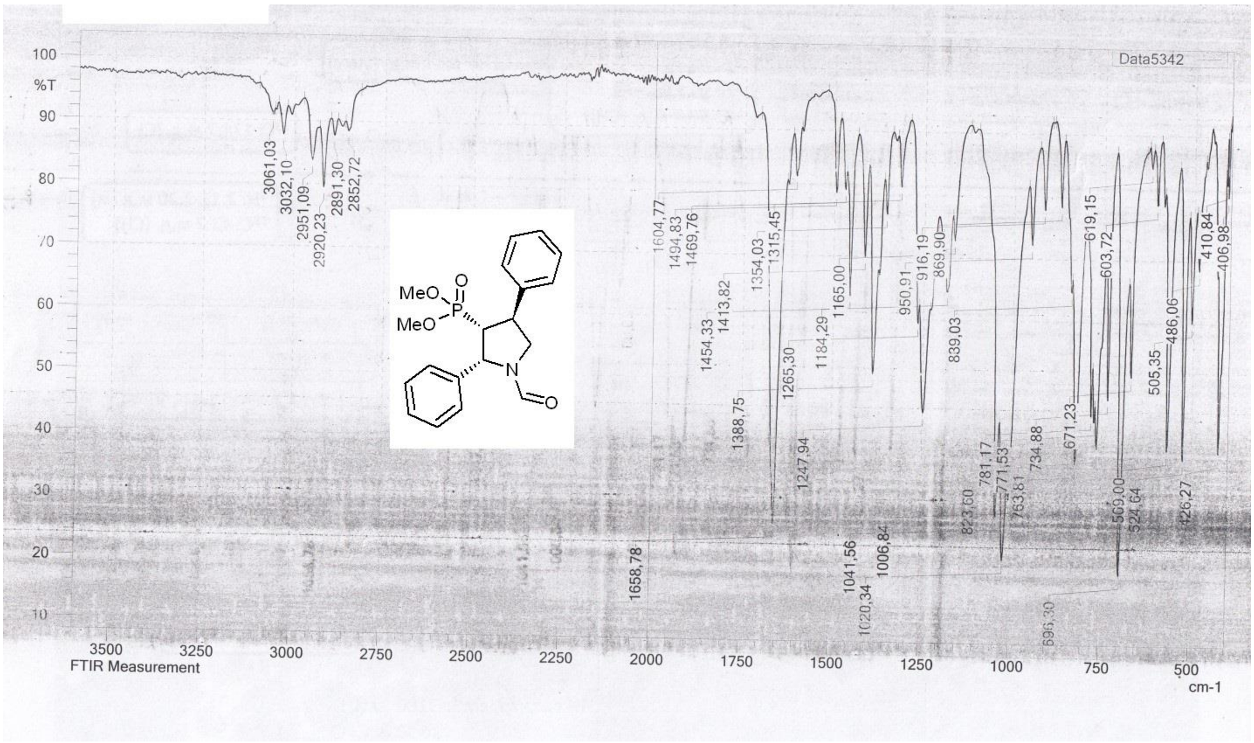

^{1}H NMR spectra of dimethyl [(2*R*,3*R*,4*S*)-1-formyl-2,4-diphenylpyrrolidin-3-yl]phosphonate (**10a**) in $CDCl_3$

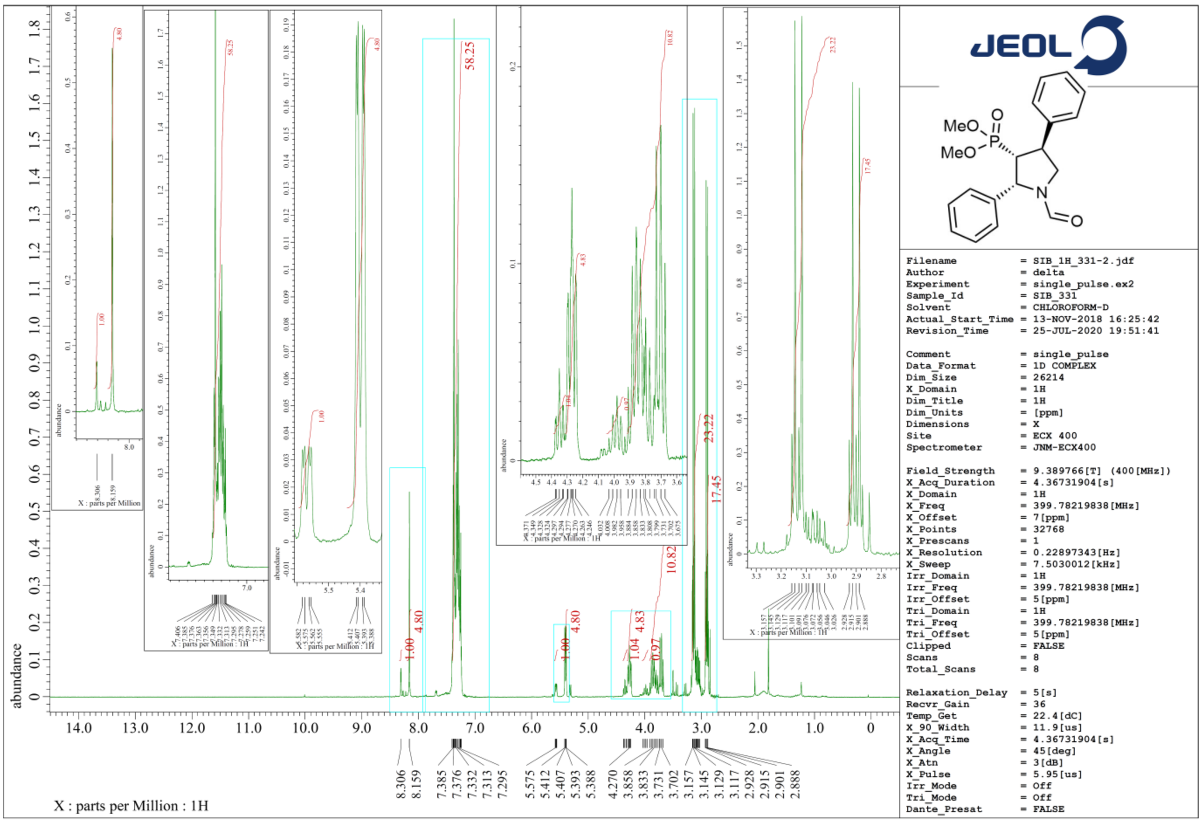

^{13}C NMR spectra of dimethyl [(2*R*,3*R*,4*S*)-1-formyl-2,4-diphenylpyrrolidin-3-yl]phosphonate (**10a**) in $CDCl_3$

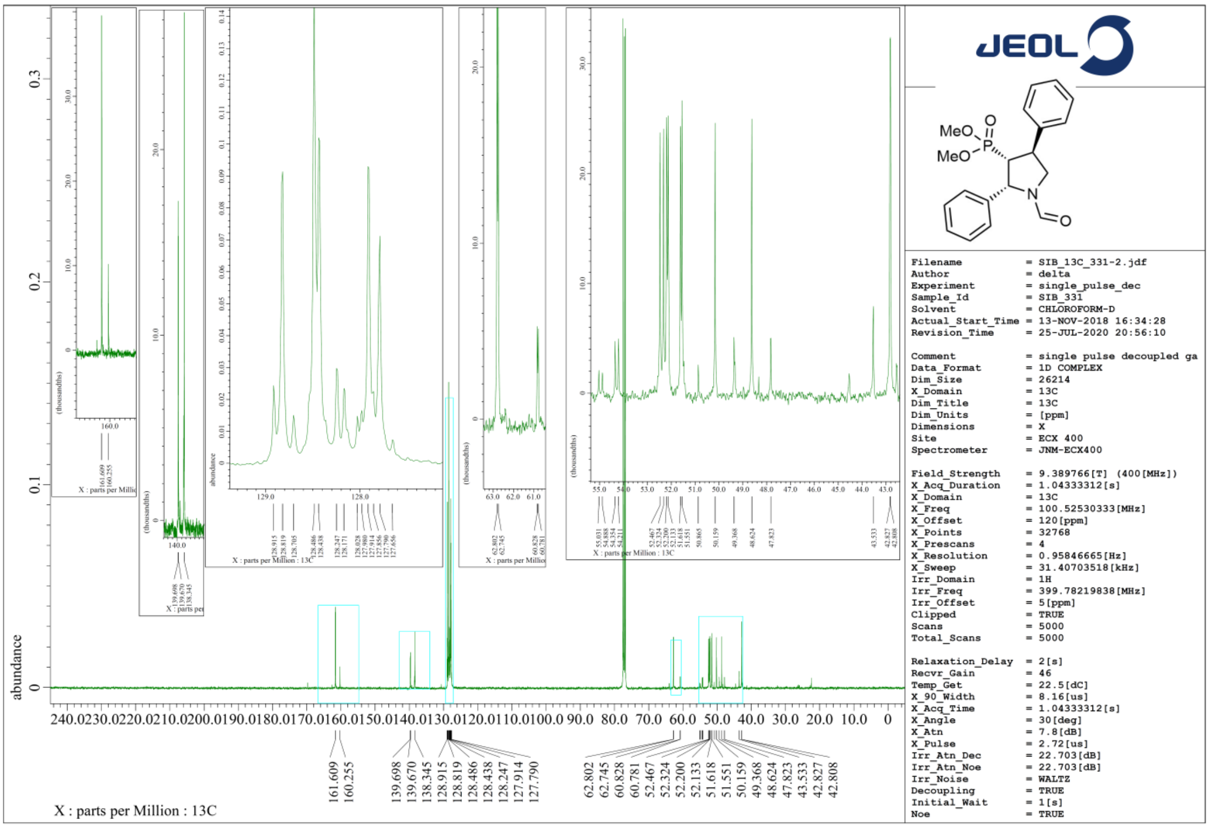

DEPT NMR spectra of dimethyl [(2*R*,3*R*,4*S*)-1-formyl-2,4-diphenylpyrrolidin-3-yl]phosphonate (**10a**) in $CDCl_3$

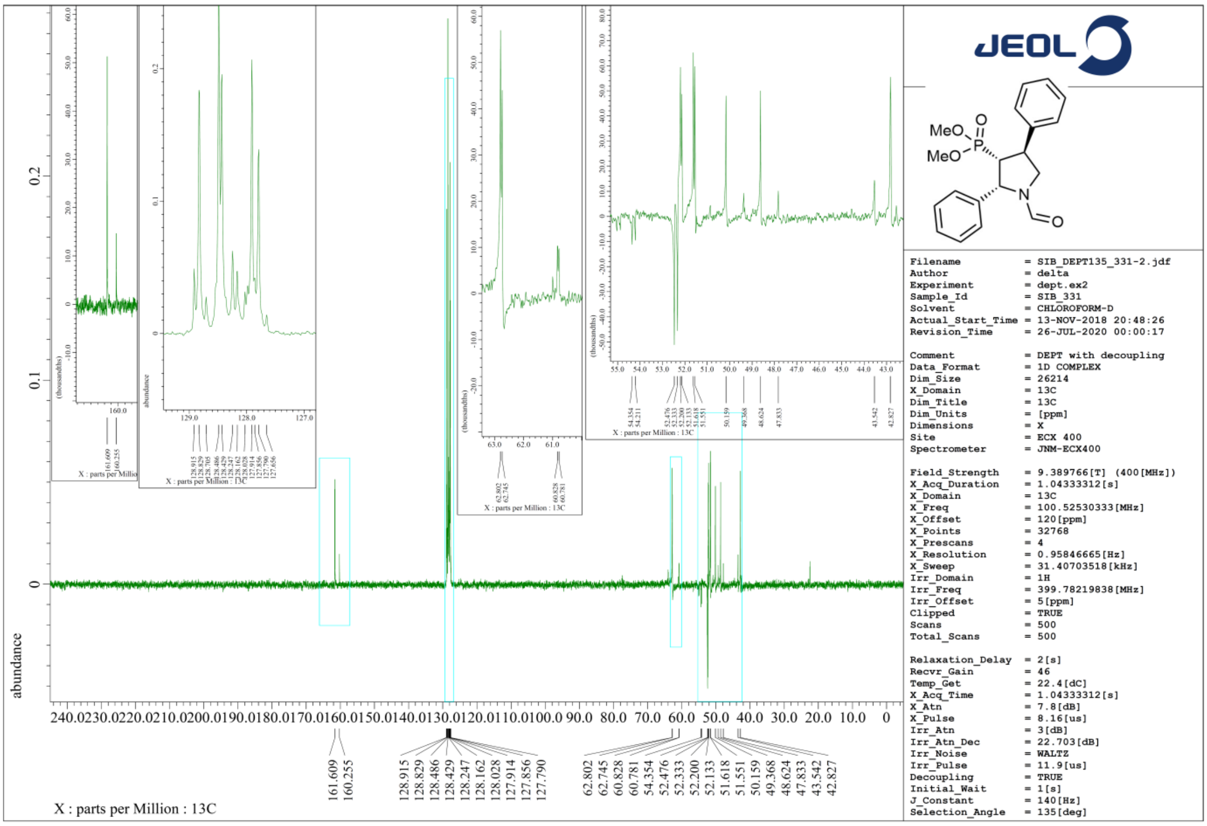

^{31}P NMR spectra of dimethyl [(2*R*,3*R*,4*S*)-1-formyl-2,4-diphenylpyrrolidin-3-yl]phosphonate (**10a**) in $CDCl_3$

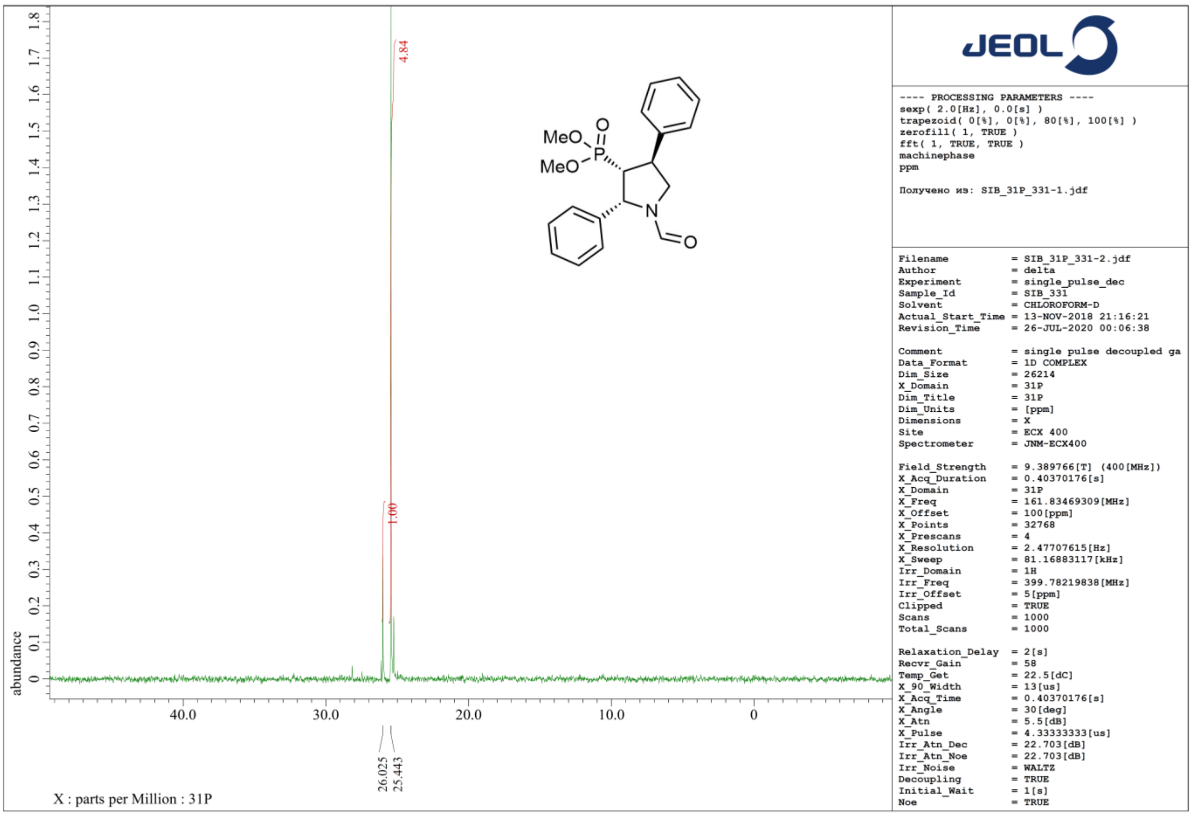

HRMS of dimethyl [(2*R*,3*R*,4*S*)-1-formyl-2,4-diphenylpyrrolidin-3-yl]phosphonate (**10a**)

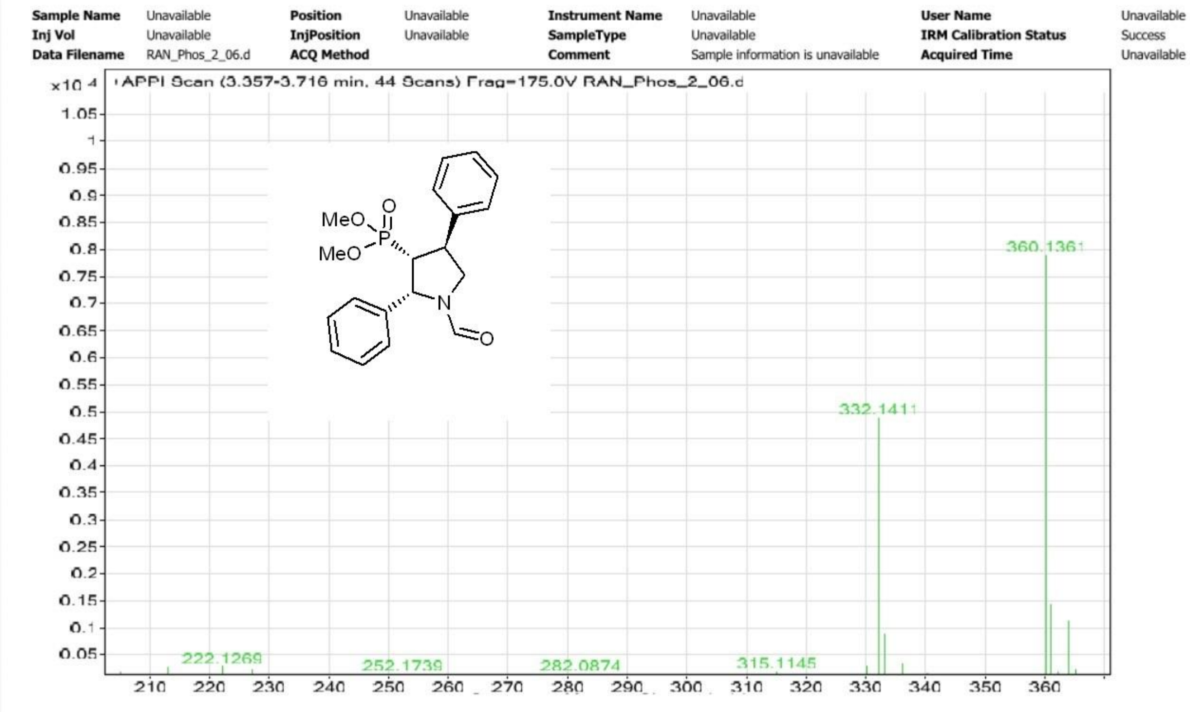

FTIR spectra of dimethyl [(2*R*,3*R*,4*S*)-1-formyl-2-(4-methoxyphenyl)-4-phenylpyrrolidin-3-yl]-phosphonate

(**10b**)

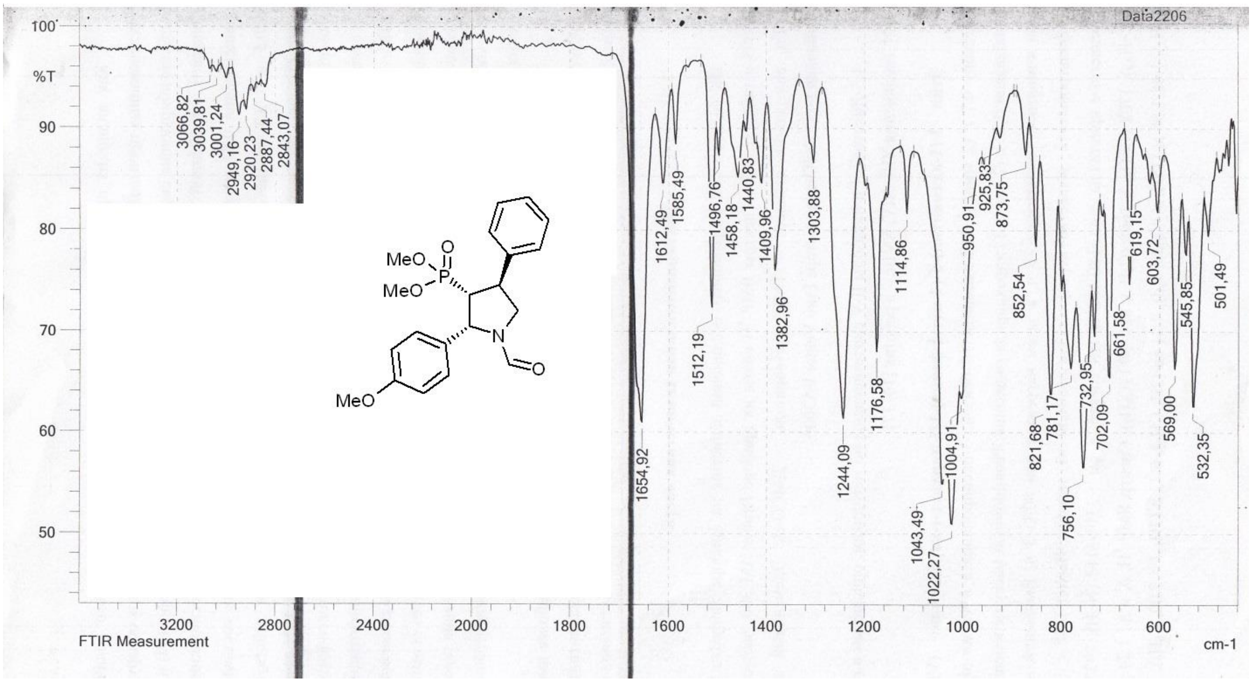

^{1}H NMR spectra of dimethyl [(2*R*,3*R*,4*S*)-1-formyl-2-(4-methoxyphenyl)-4-phenylpyrrolidin-3-yl]-phosphonate (**10b**) in $CDCl_3$

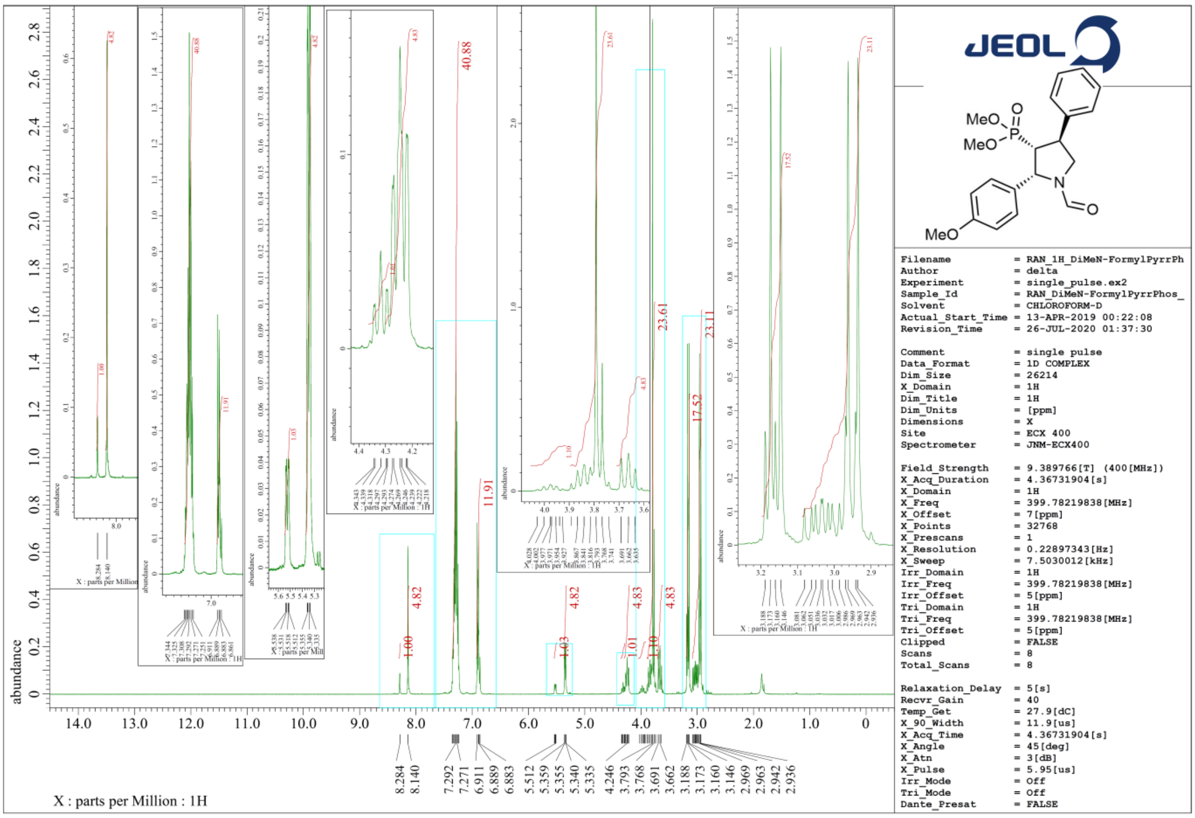

^{13}C NMR spectra of dimethyl [(2*R*,3*R*,4*S*)-1-formyl-2-(4-methoxyphenyl)-4-phenylpyrrolidin-3-yl]-phosphonate (**10b**) in $CDCl_3$

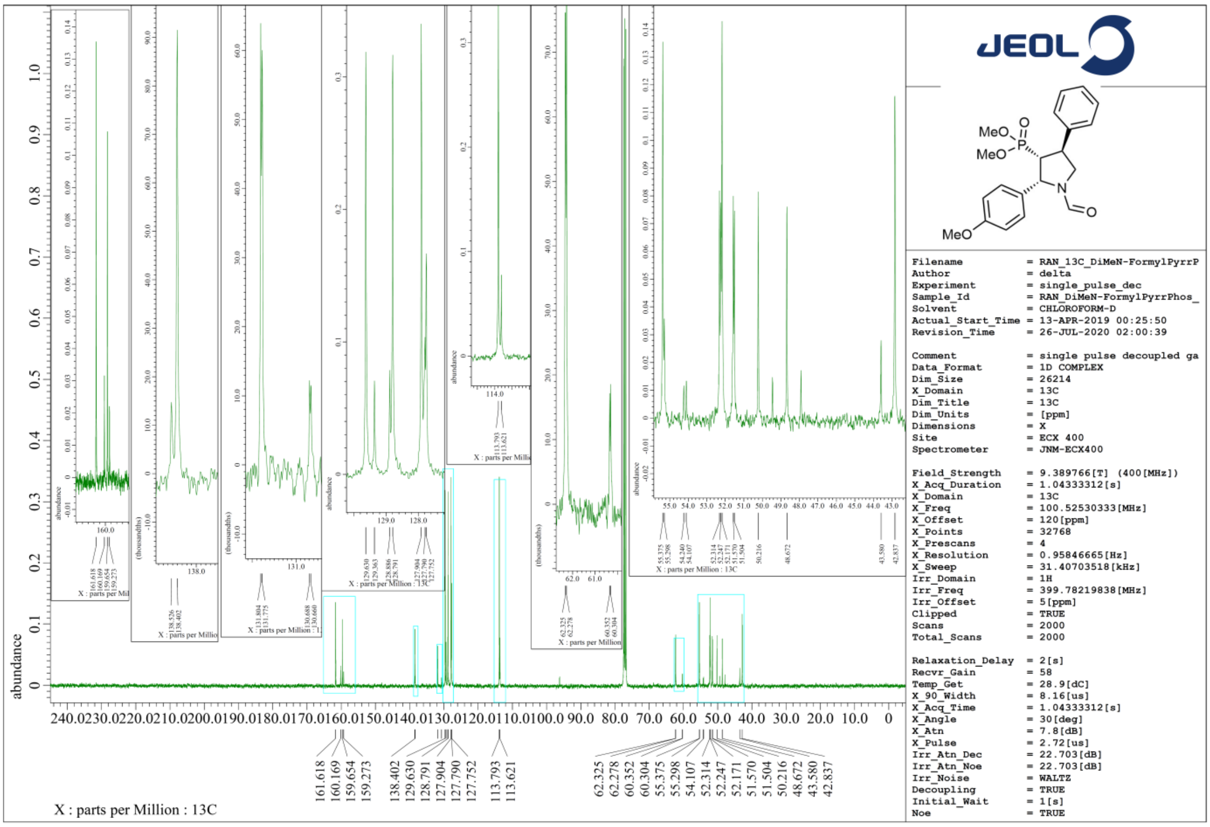

DEPT NMR spectra of dimethyl [(2*R*,3*R*,4*S*)-1-formyl-2-(4-methoxyphenyl)-4-phenylpyrrolidin-3-yl]-phosphonate (**10b**) in $CDCl_3$

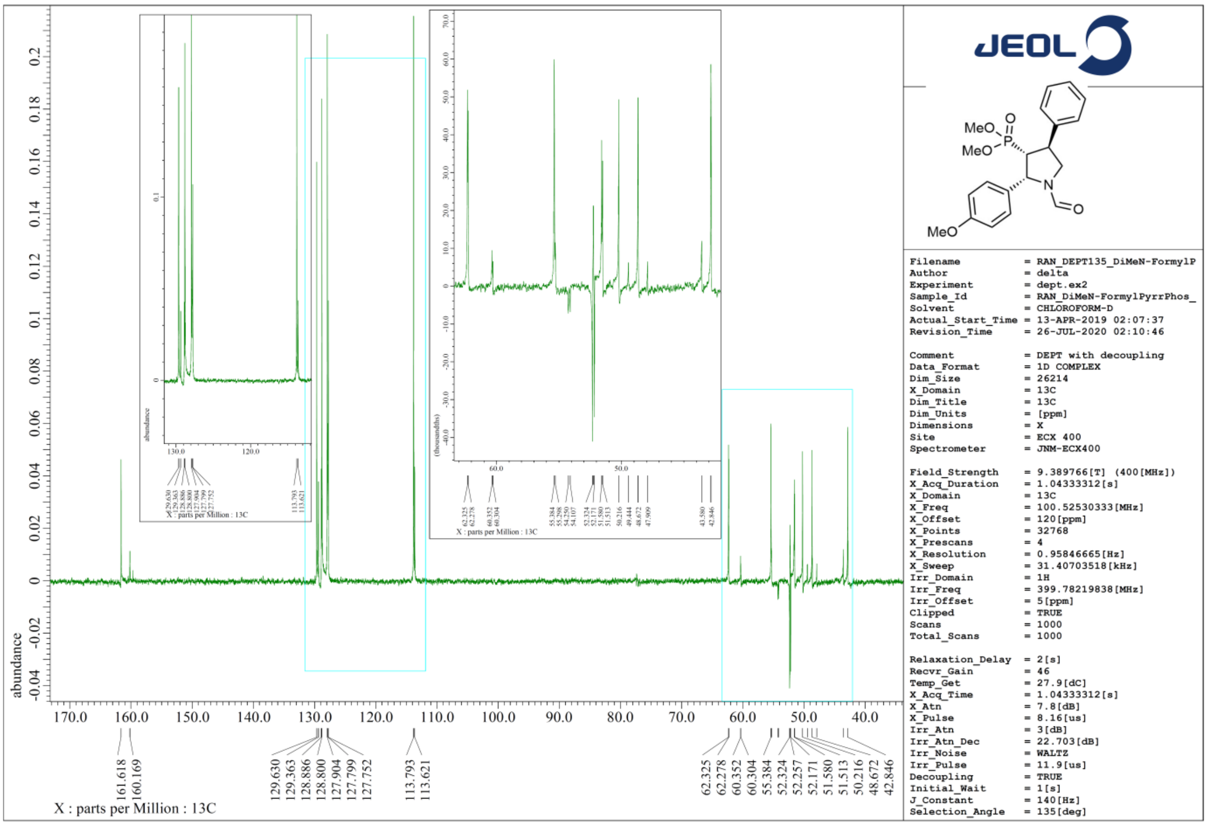

^{31}P NMR spectra of dimethyl [(2*R*,3*R*,4*S*)-1-formyl-2-(4-methoxyphenyl)-4-phenylpyrrolidin-3-yl]-phosphonate (**10b**) in $CDCl_3$

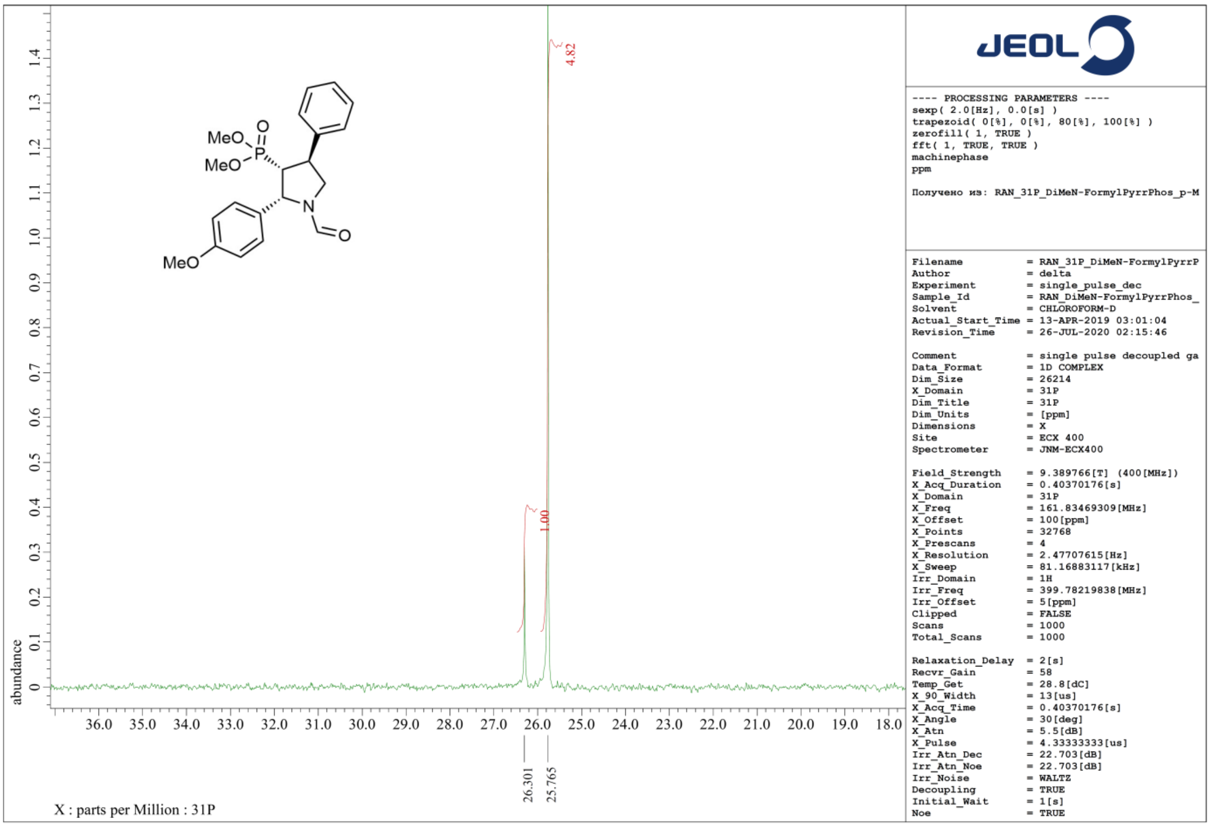

HRMS of dimethyl [(2*R*,3*R*,4*S*)-1-formyl-2-(4-methoxyphenyl)-4-phenylpyrrolidin-3-yl]-phosphonate (**10b**)

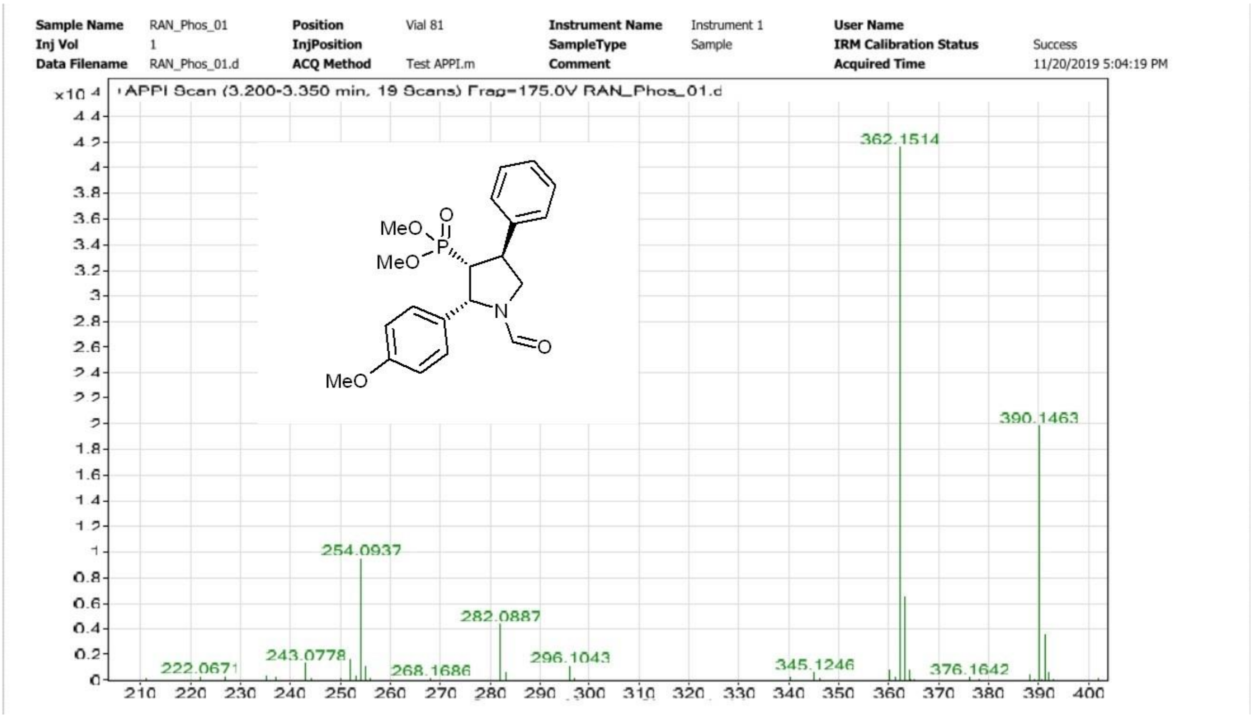

FTIR spectra of dimethyl [(2*R*,3*R*,4*S*)-1-formyl-2-(3-methoxyphenyl)-4-phenylpyrrolidin-3-yl]-phosphonate

(**10c**)

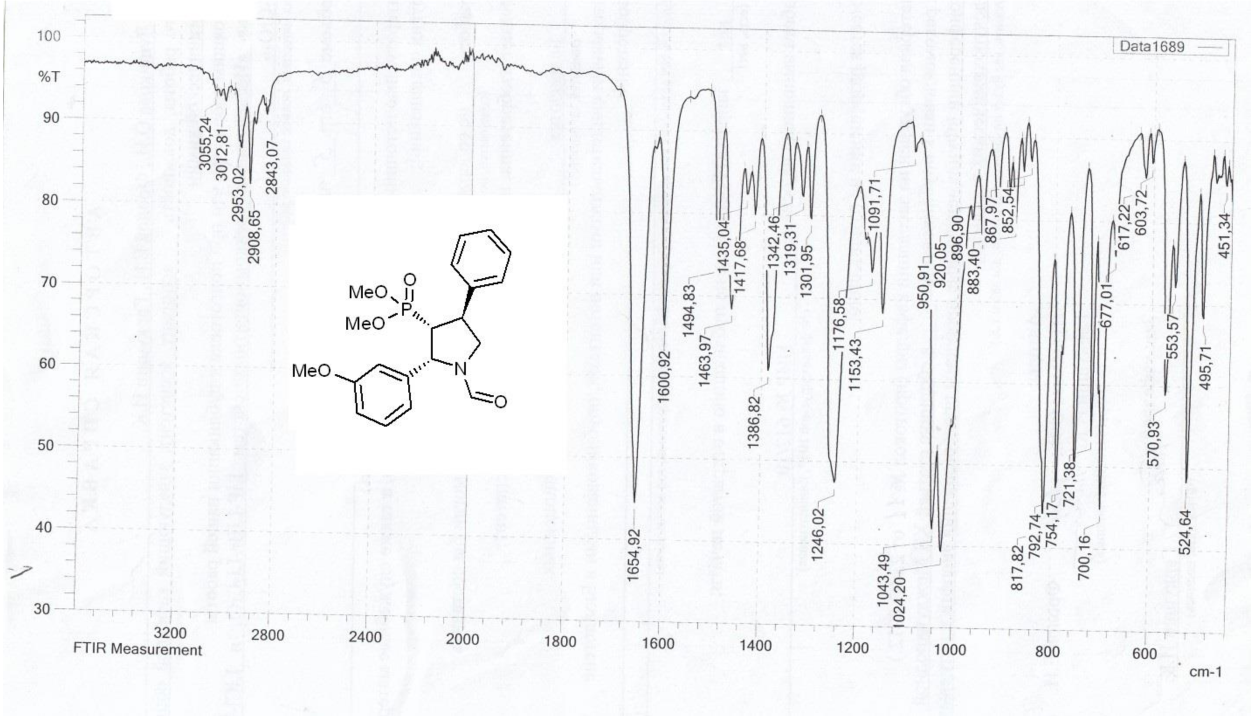

^{1}H NMR spectra of dimethyl [(2*R*,3*R*,4*S*)-1-formyl-2-(3-methoxyphenyl)-4-phenylpyrrolidin-3-yl]-phosphonate (**10c**) in $CDCl_3$

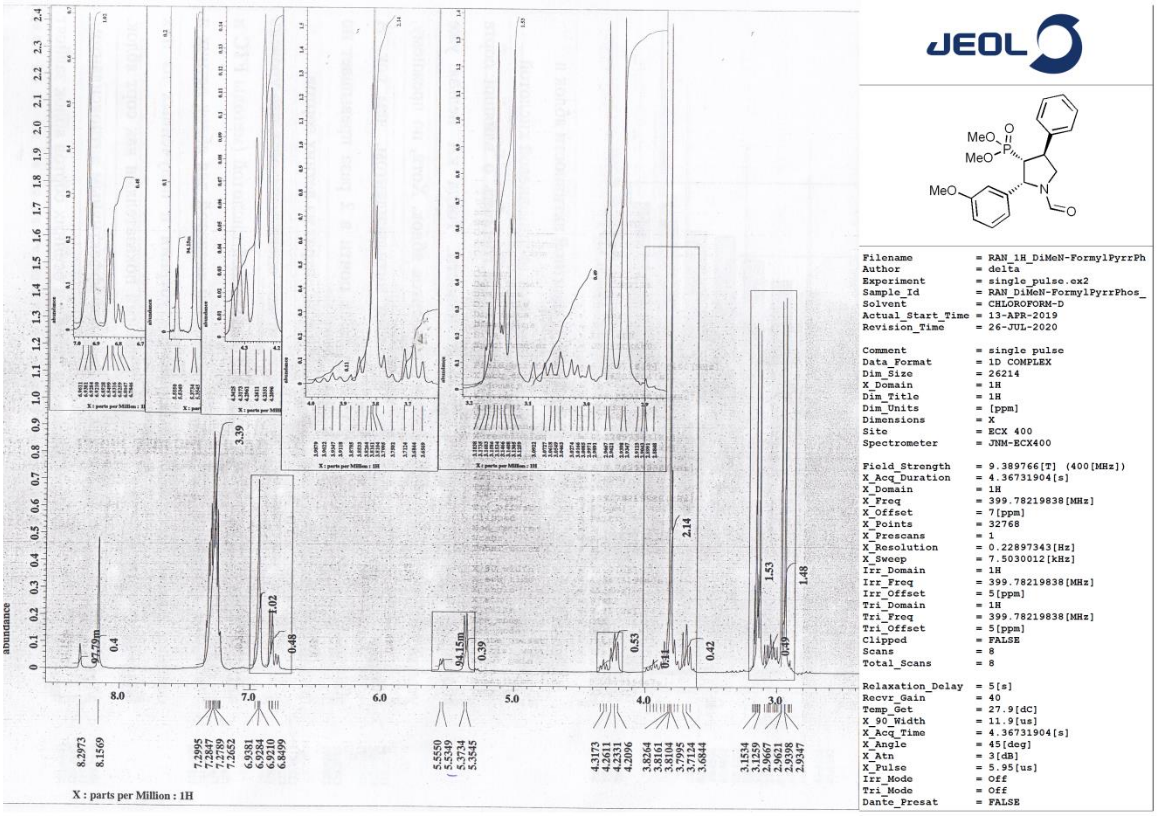

^{13}C NMR spectra of dimethyl [(2*R*,3*R*,4*S*)-1-formyl-2-(3-methoxyphenyl)-4-phenylpyrrolidin-3-yl]-phosphonate (**10c**) in $CDCl_3$

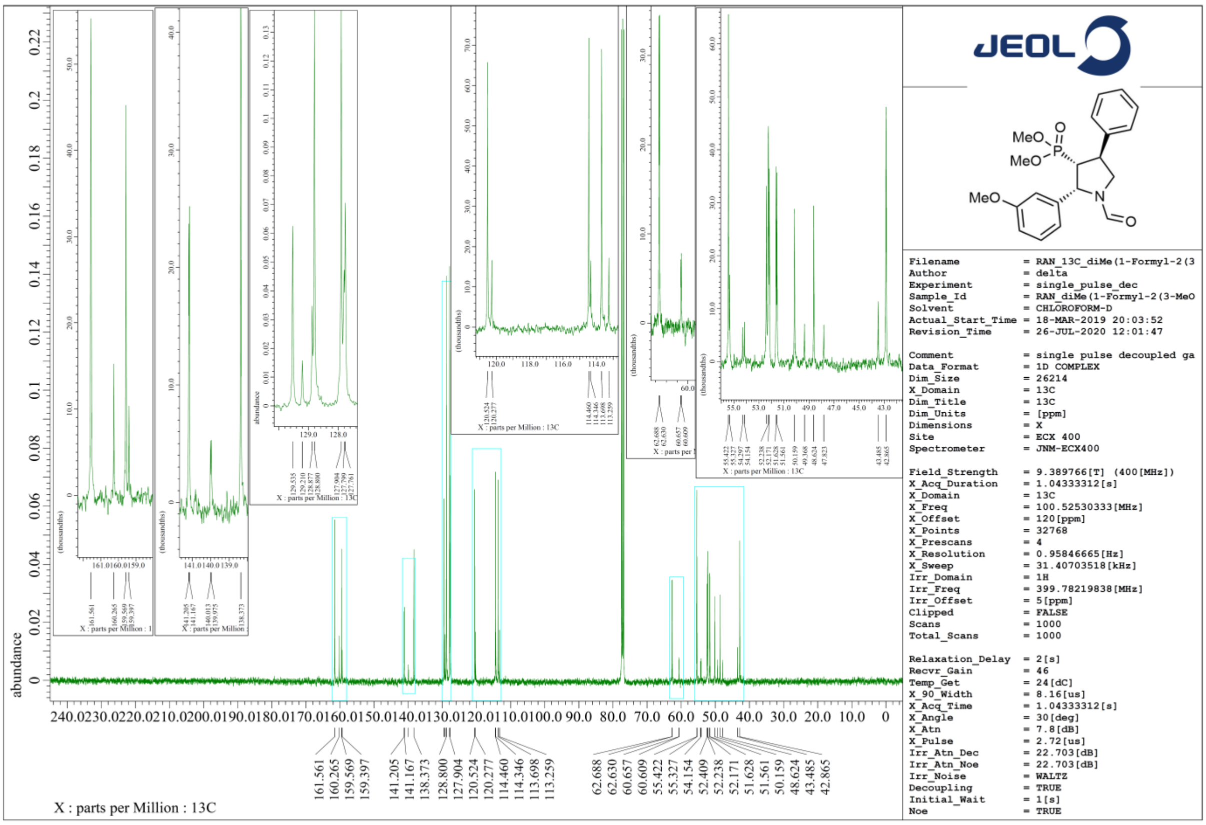

DEPT NMR spectra of dimethyl [(2*R*,3*R*,4*S*)-1-formyl-2-(3-methoxyphenyl)-4-phenylpyrrolidin-3-yl]-phosphonate (**10c**) in $CDCl_3$

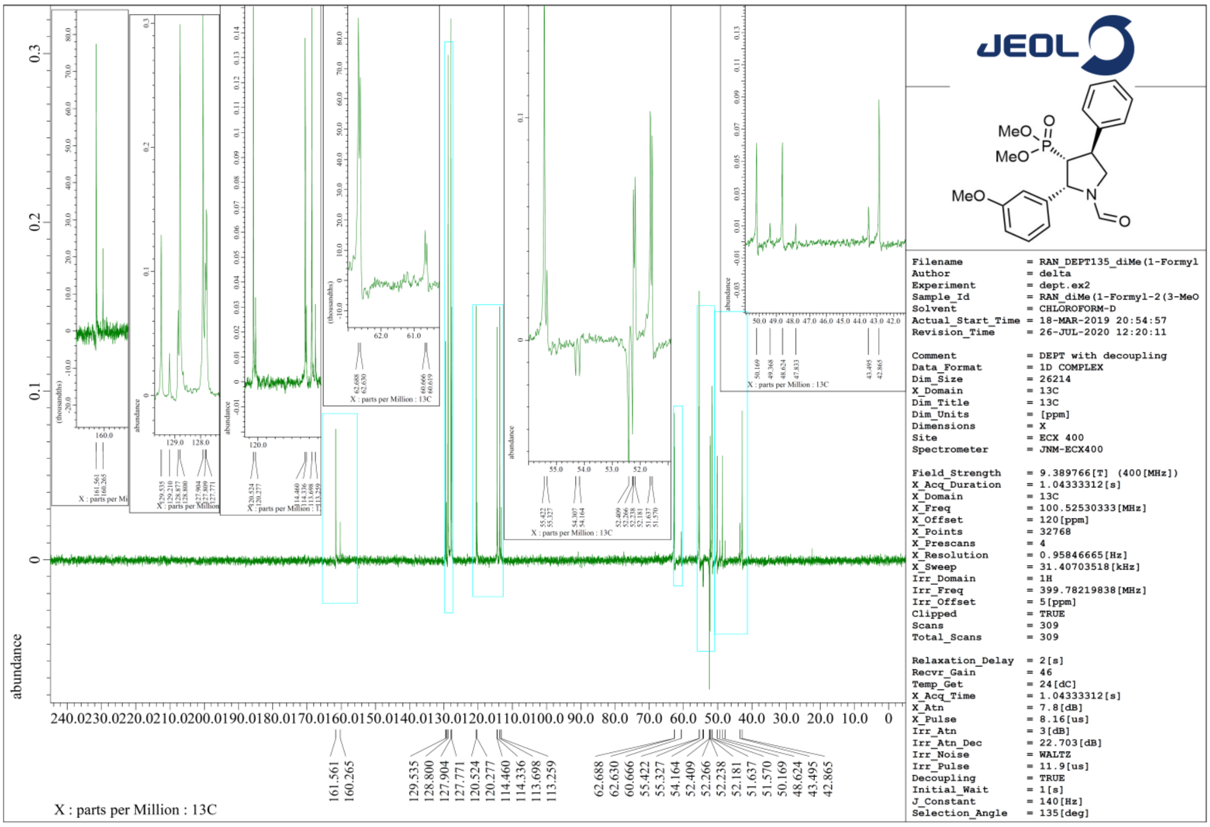

^{31}P NMR spectra of dimethyl [(2*R*,3*R*,4*S*)-1-formyl-2-(3-methoxyphenyl)-4-phenylpyrrolidin-3-yl]-phosphonate (**10c**) in $CDCl_3$

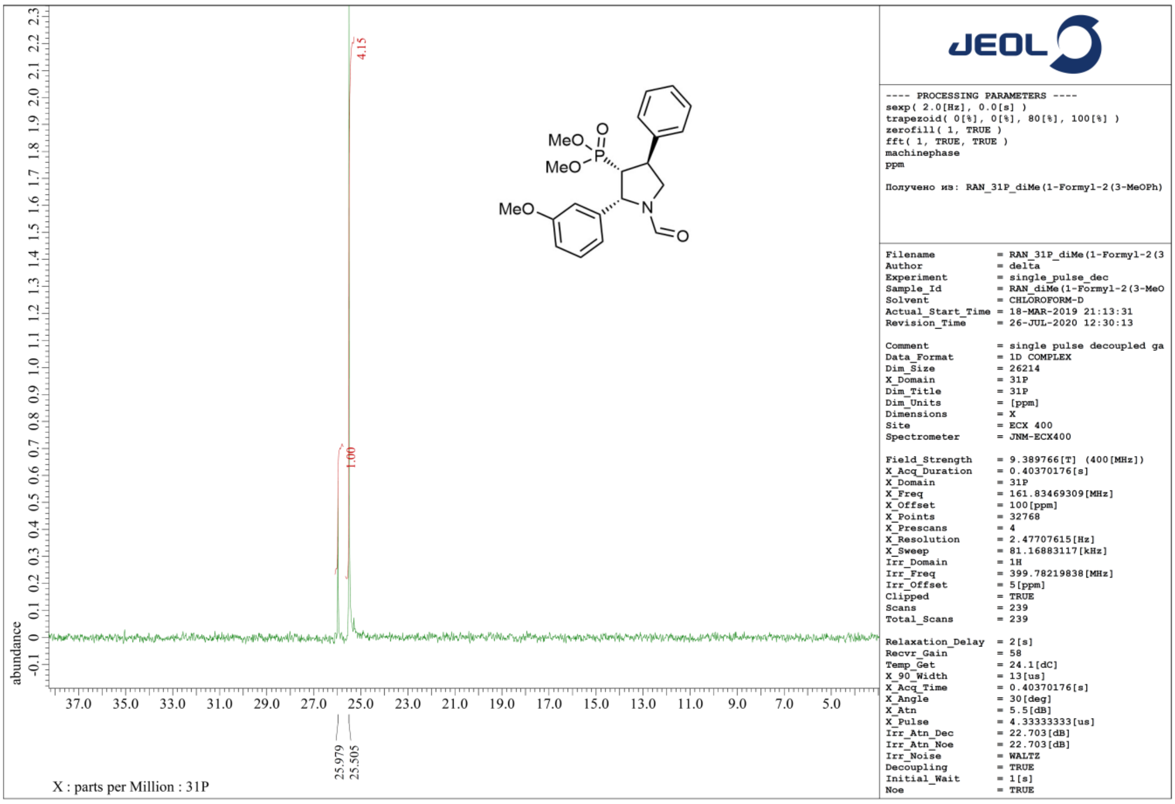

HRMS of dimethyl [(2*R*,3*R*,4*S*)-1-formyl-2-(3-methoxyphenyl)-4-phenylpyrrolidin-3-yl]-phosphonate (**10c**)

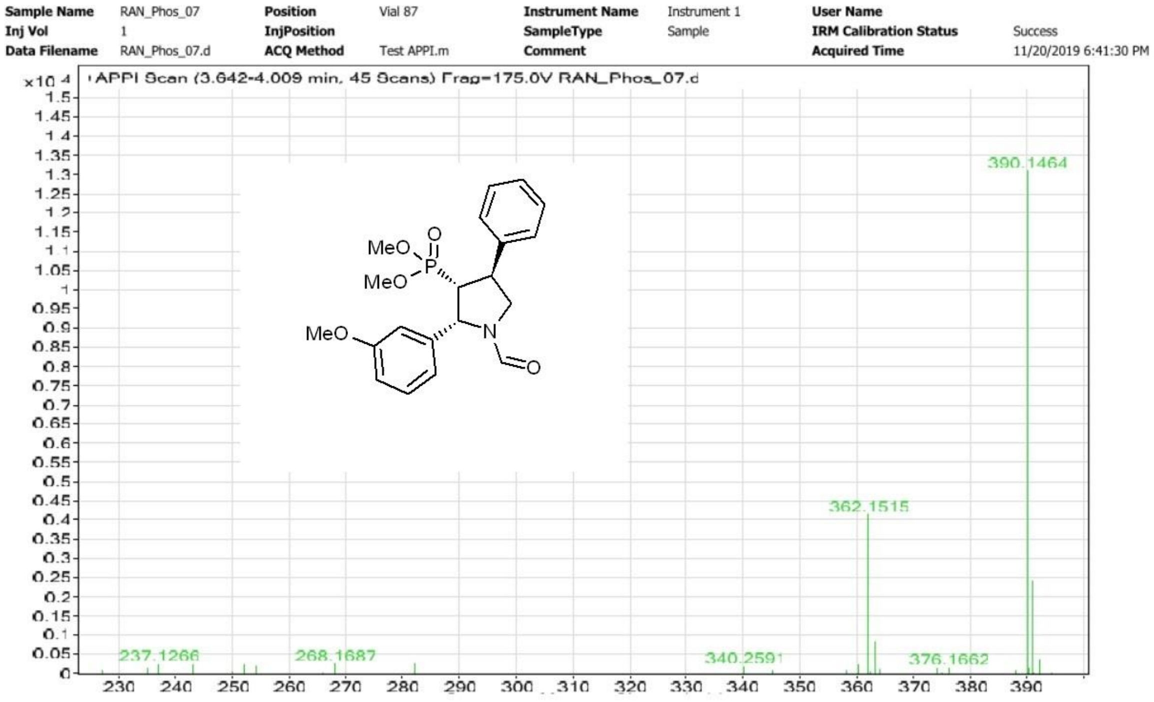

FTIR spectra of dimethyl [(2*R*,3*R*,4*S*)-1-formyl-2-methyl-4-phenylpyrrolidin-3-yl]phosphonate (**10d**)

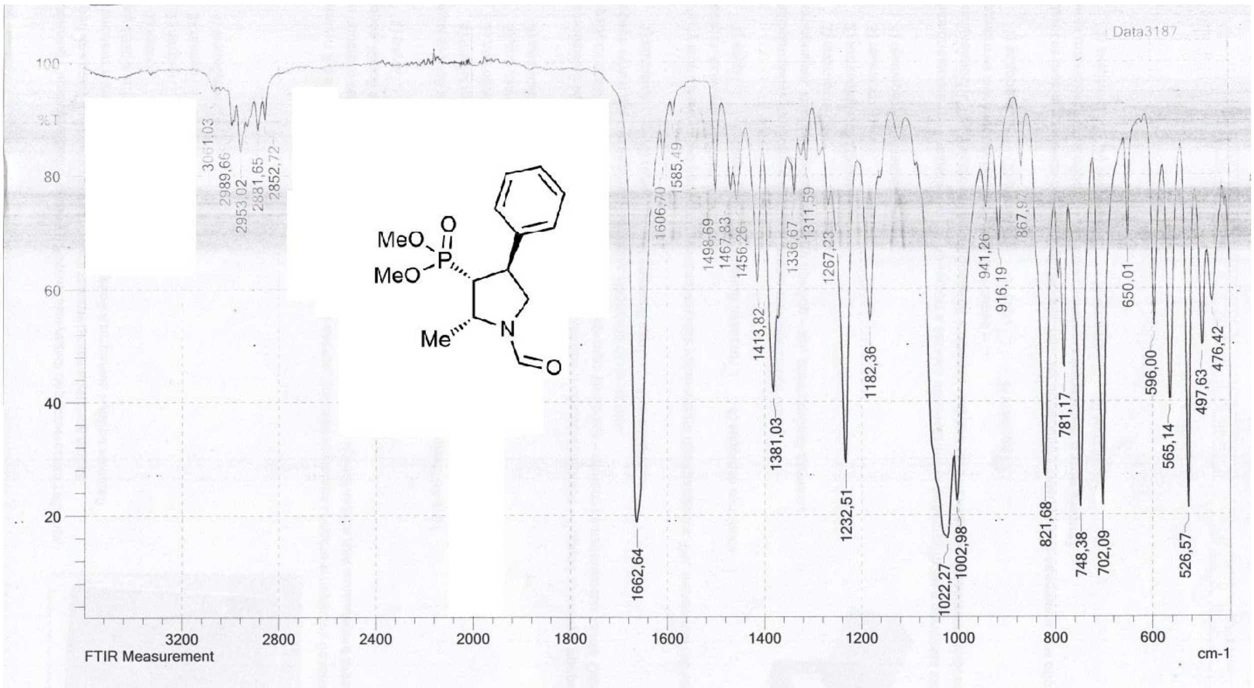

^{1}H NMR spectra of dimethyl [(2*R*,3*R*,4*S*)-1-formyl-2-methyl-4-phenylpyrrolidin-3-yl]phosphonate (**10d**) in

CDCl3

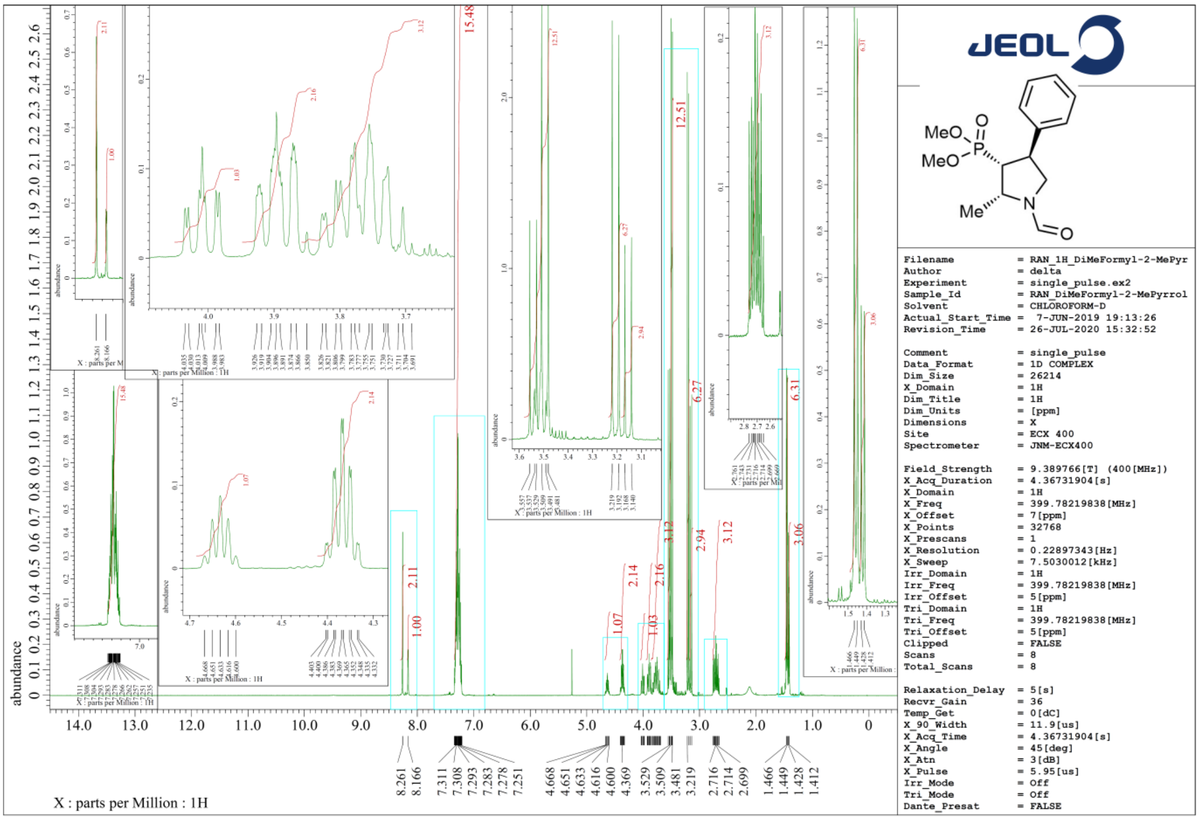

^{13}C NMR spectra of dimethyl [(2*R*,3*R*,4*S*)-1-formyl-2-methyl-4-phenylpyrrolidin-3-yl]phosphonate (**10d**) in

CDCl3

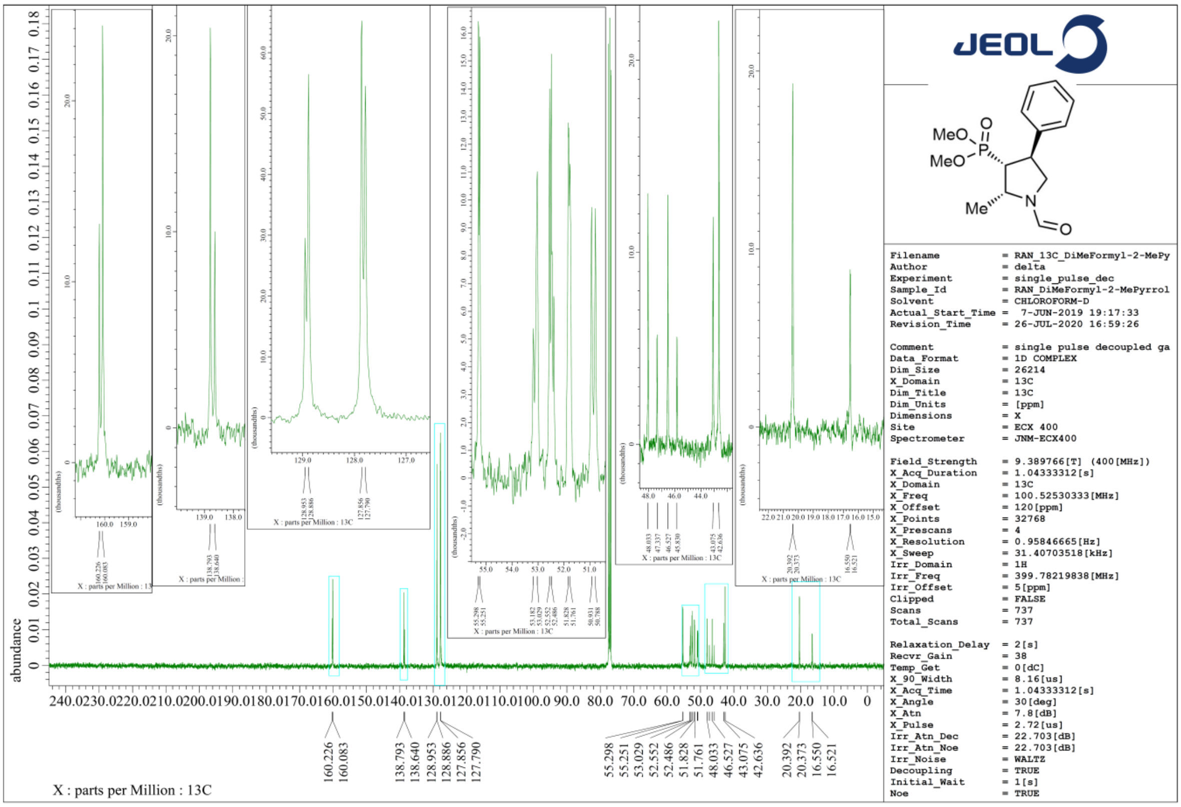

DEPT NMR spectra of dimethyl [(2*R*,3*R*,4*S*)-1-formyl-2-methyl-4-phenylpyrrolidin-3-yl]phosphonate (**10d**) in $CDCl_3$

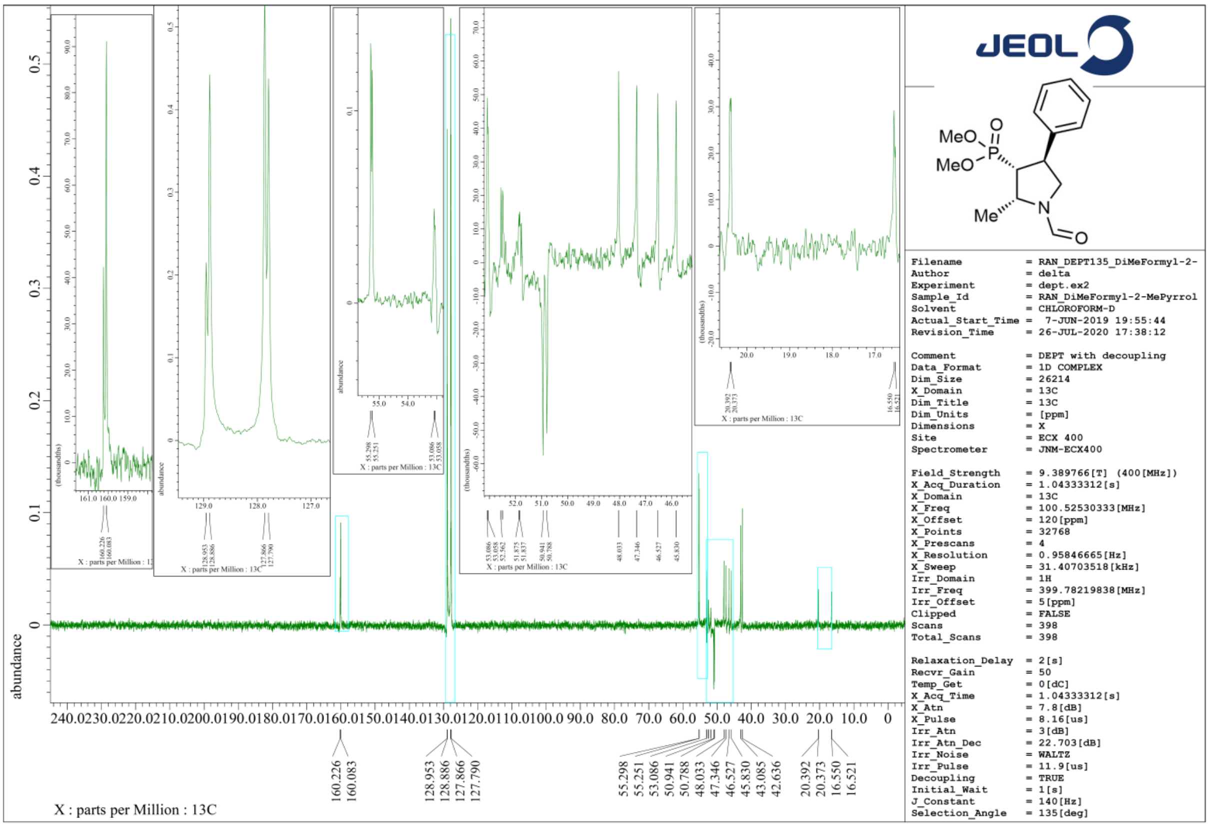

^{31}P NMR spectra of dimethyl [(2*R*,3*R*,4*S*)-1-formyl-2-methyl-4-phenylpyrrolidin-3-yl]phosphonate (**10d**) in $CDCl_3$

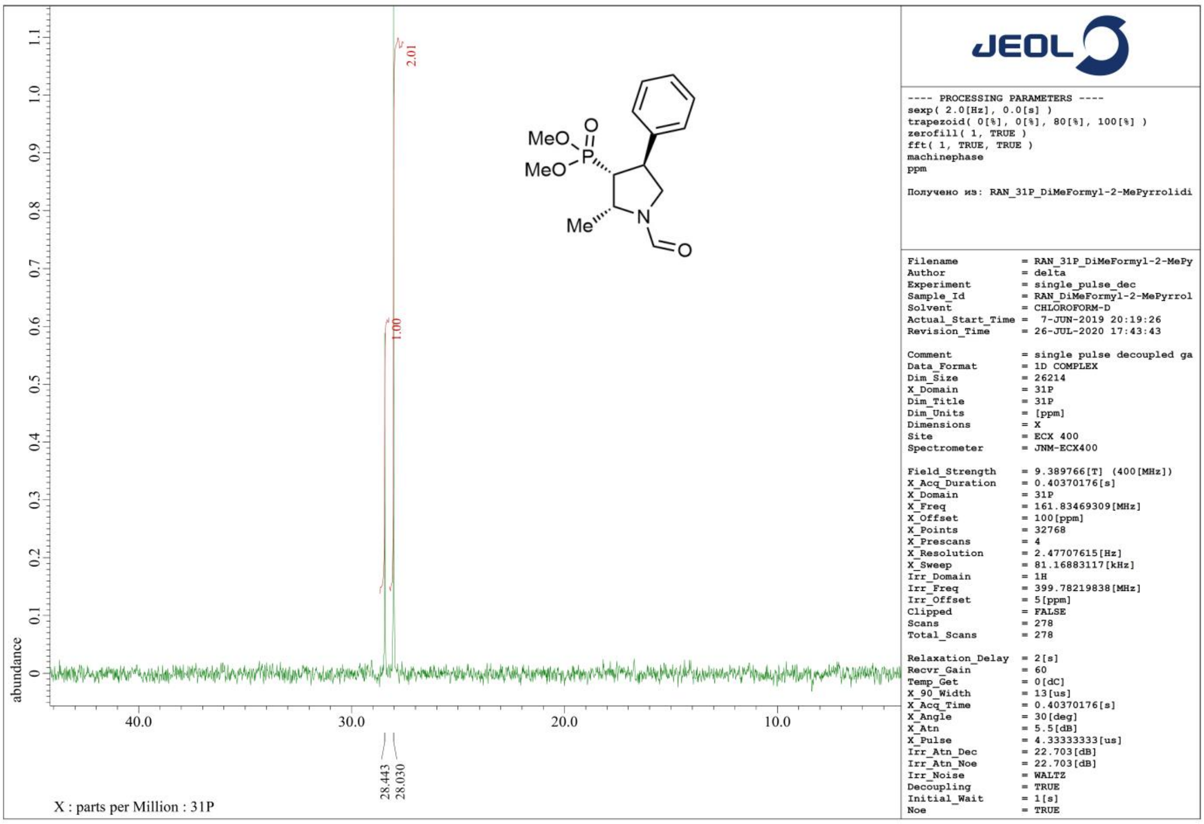

HRMS of dimethyl [(2*R*,3*R*,4*S*)-1-formyl-2-methyl-4-phenylpyrrolidin-3-yl]phosphonate (**10d**)

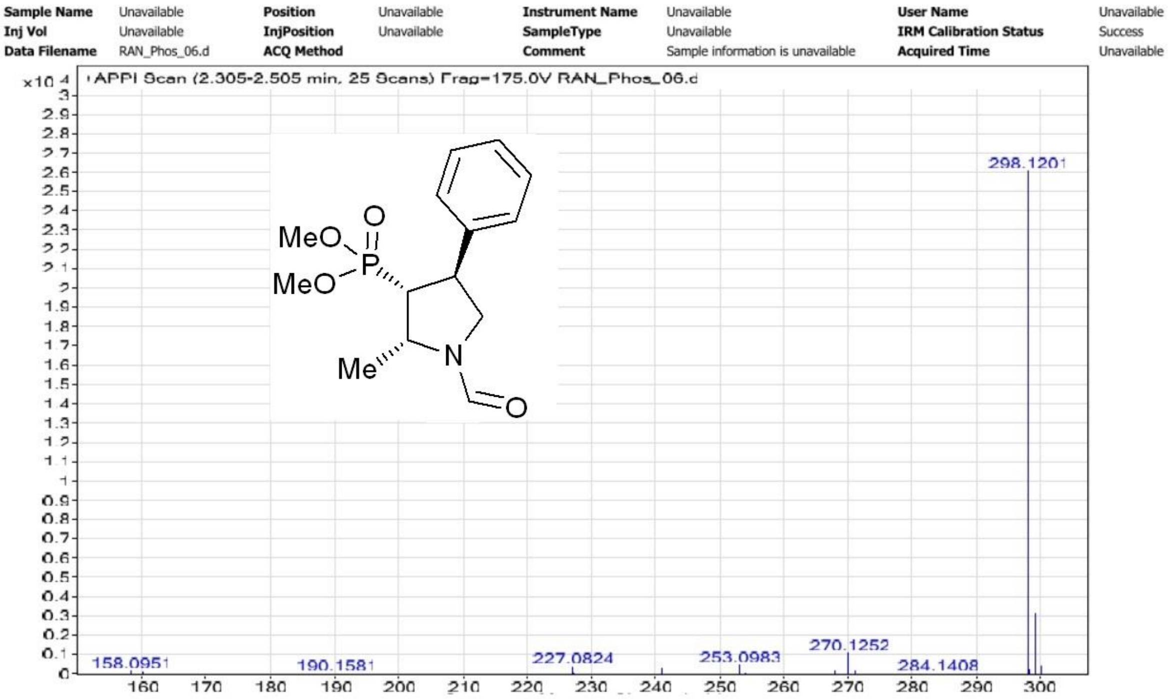

FTIR spectra of (2*R*,3*R*,4*S*)-2,4-Diphenylpyrrolidin-3-ylphosphonic acid hydrochloride (**11a**)

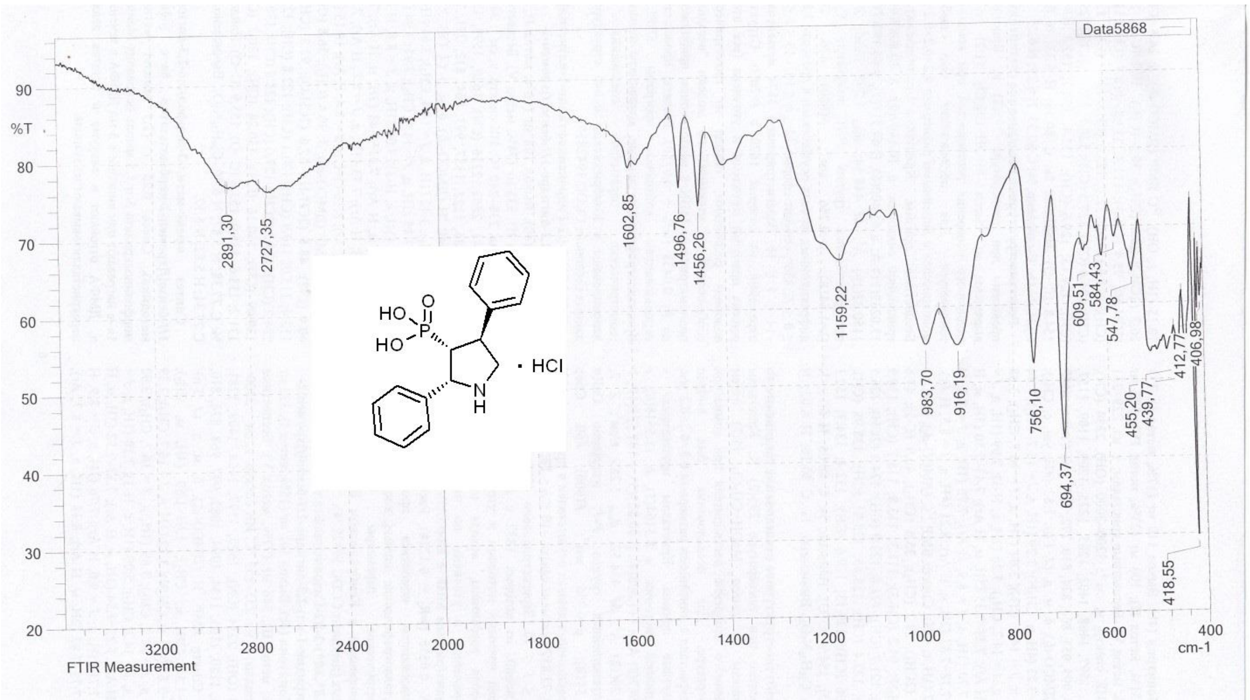

^{1}H NMR spectra of (2*R*,3*R*,4*S*)-2,4-Diphenylpyrrolidin-3-ylphosphonic acid hydrochloride (**11a**) in D_2O

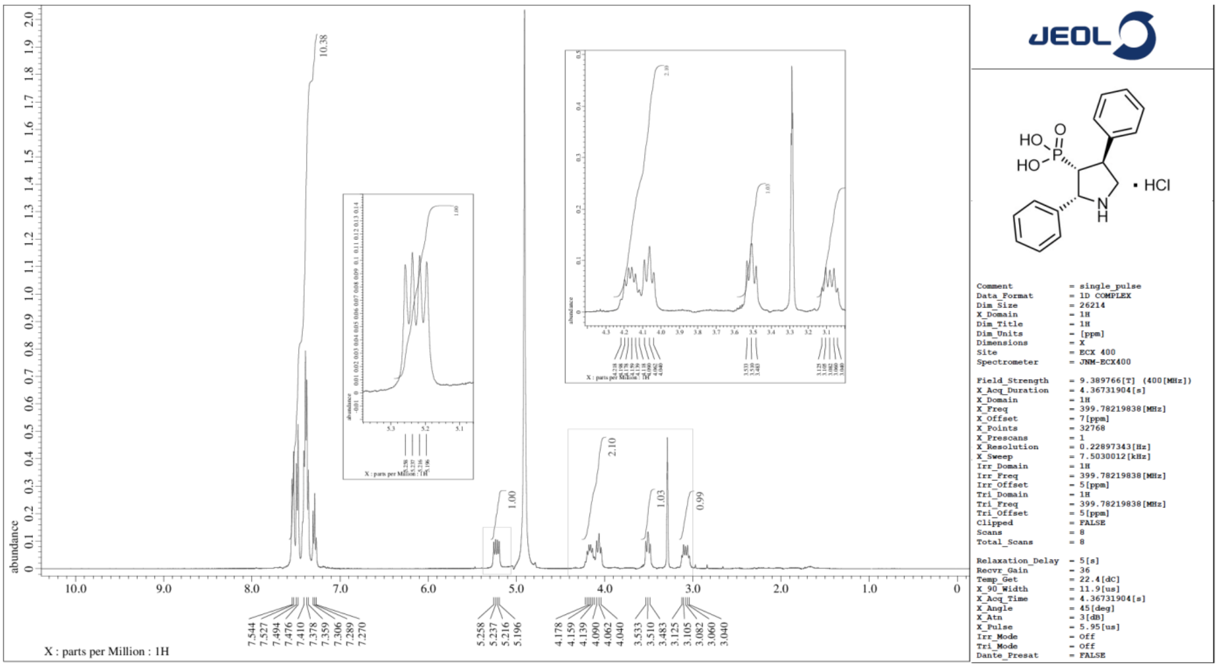

^{13}C NMR spectra of (2*R*,3*R*,4*S*)-2,4-Diphenylpyrrolidin-3-ylphosphonic acid hydrochloride (**11a**) in D_2O

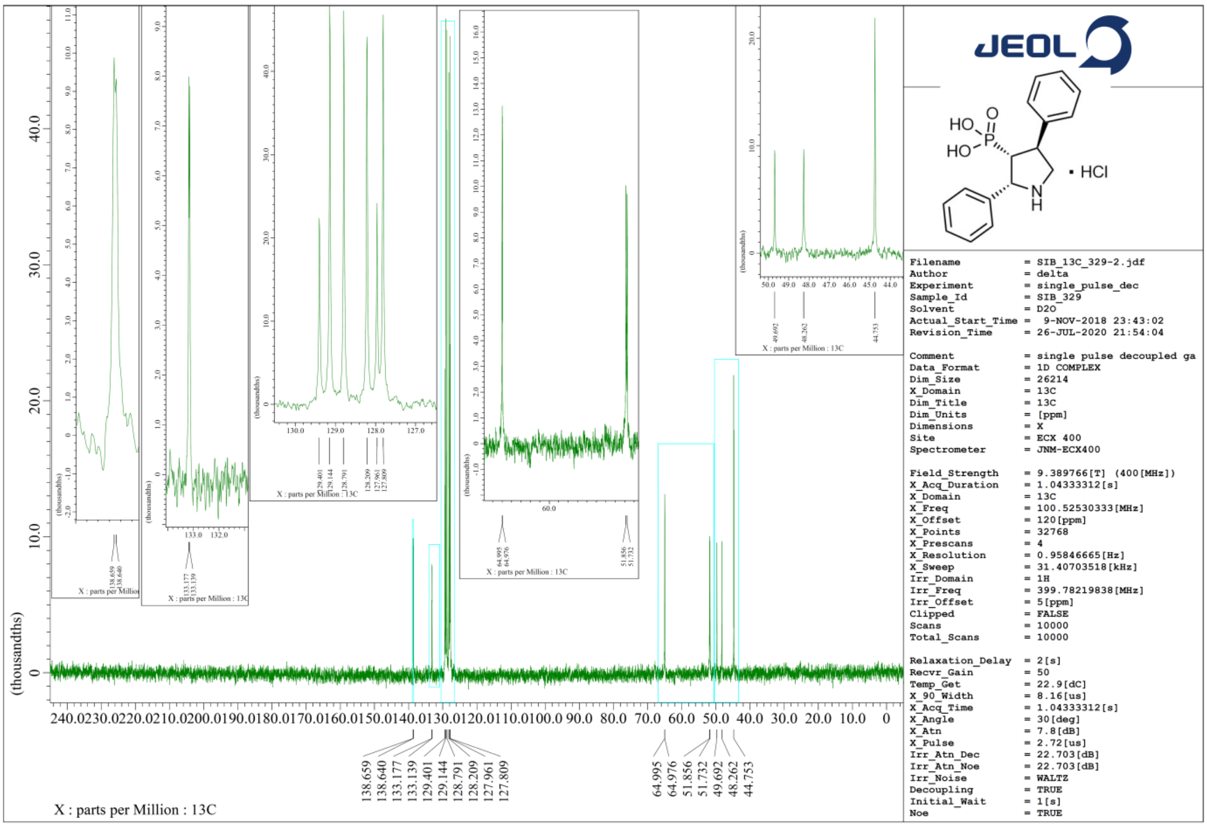

DEPT NMR spectra of (2*R*,3*R*,4*S*)-2,4-Diphenylpyrrolidin-3-ylphosphonic acid hydrochloride (**11a**) in D_2O

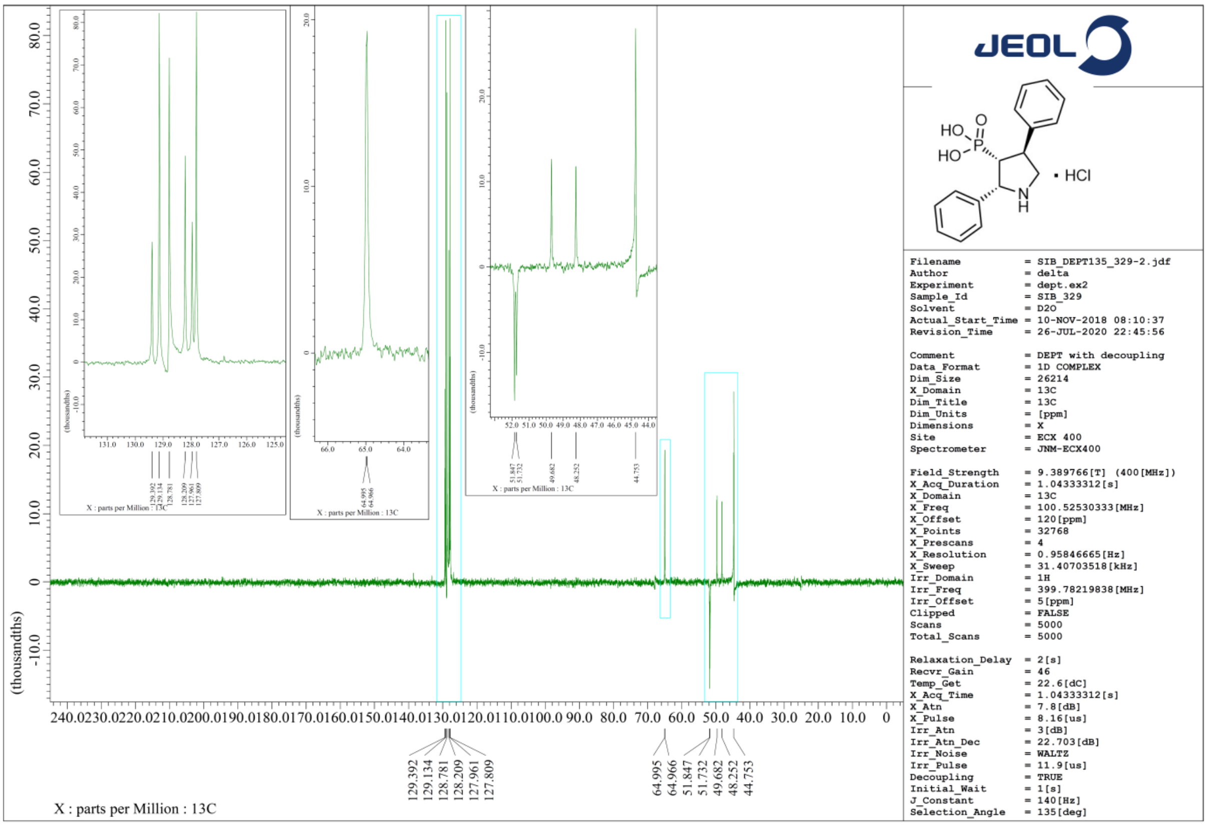

HMQC NMR spectra of (2*R*,3*R*,4*S*)-2,4-Diphenylpyrrolidin-3-ylphosphonic acid hydrochloride (**11a**) in D_2O

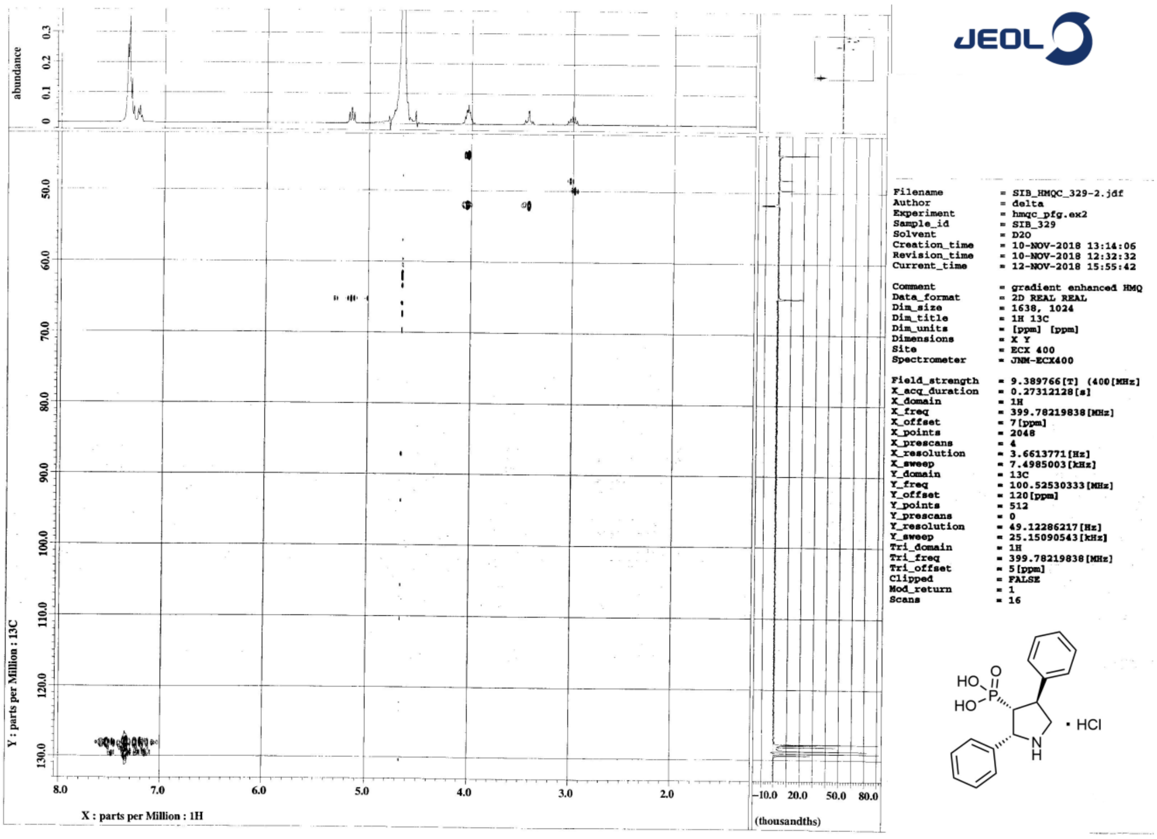

^{31}P NMR spectra of (2*R*,3*R*,4*S*)-2,4-Diphenylpyrrolidin-3-ylphosphonic acid hydrochloride (**11a**) in D_2O

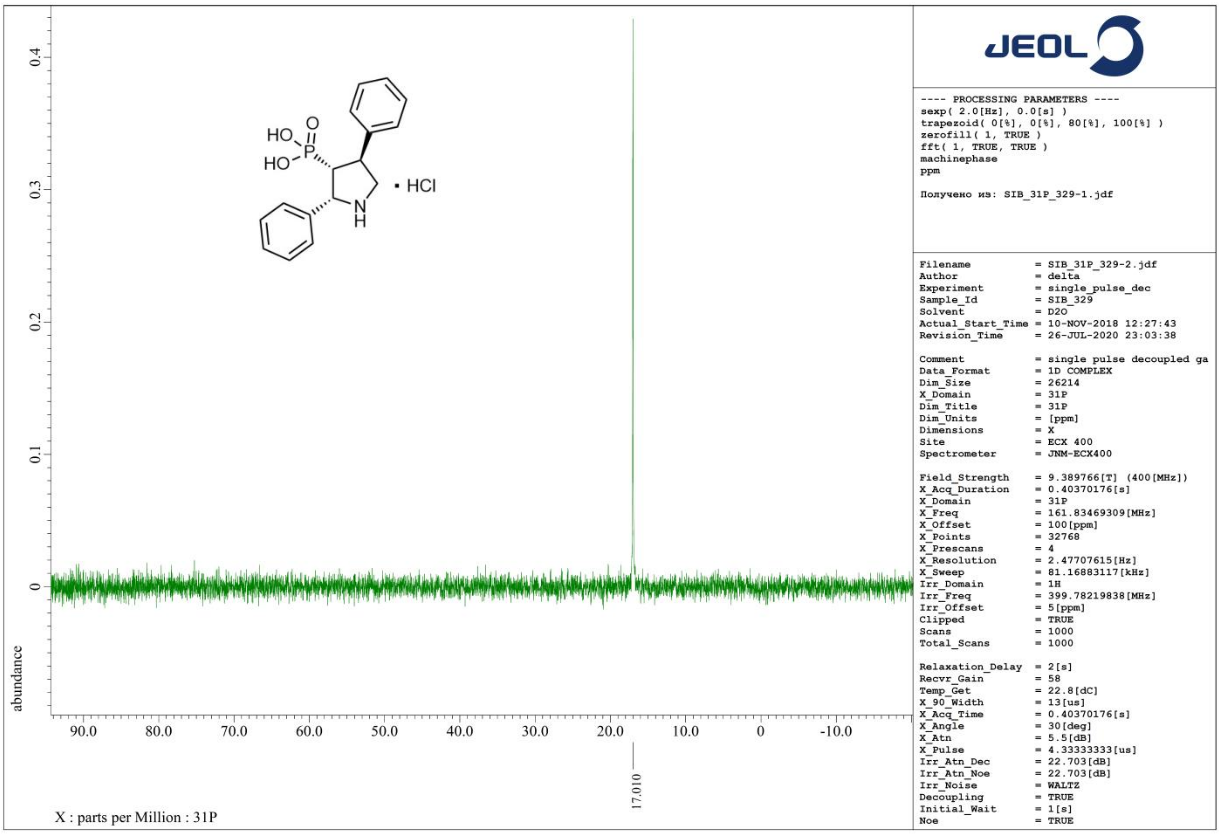

HRMS of (2*R*,3*R*,4*S*)-2,4-Diphenylpyrrolidin-3-ylphosphonic acid hydrochloride (**11a**)

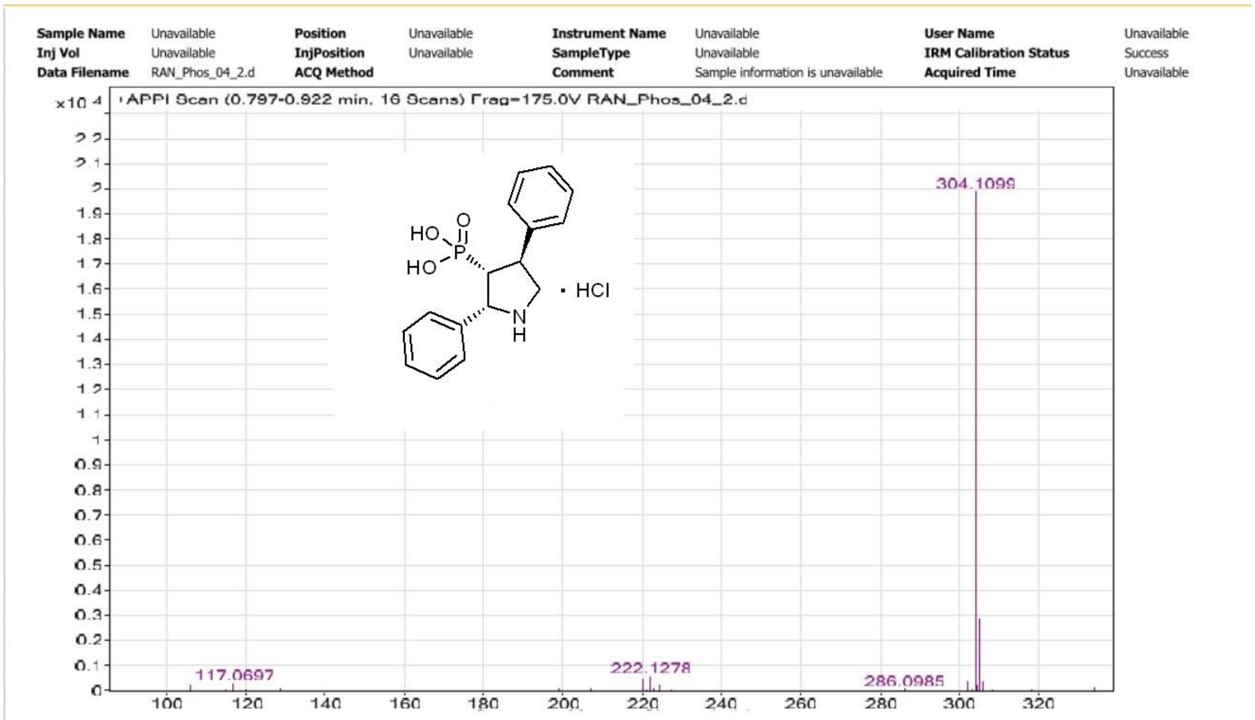

FTIR spectra of (2*R*,3*R*,4*S*)-2-(4-methoxyphenyl)-4-phenylpyrrolidin-3-ylphosphonic acid hydrochloride

(**11b**)

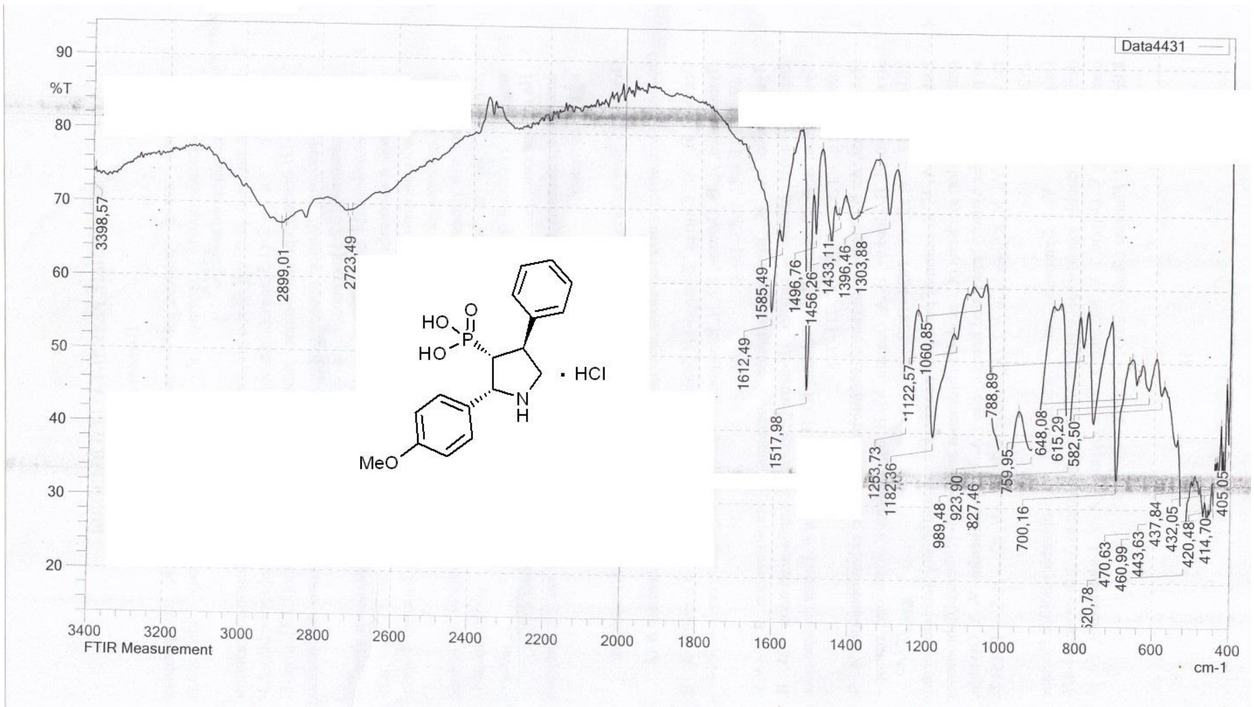

^{1}H NMR spectra of (2*R*,3*R*,4*S*)-2-(4-methoxyphenyl)-4-phenylpyrrolidin-3-ylphosphonic acid hydrochloride

(**11b**) in DMSO-*d*[6]

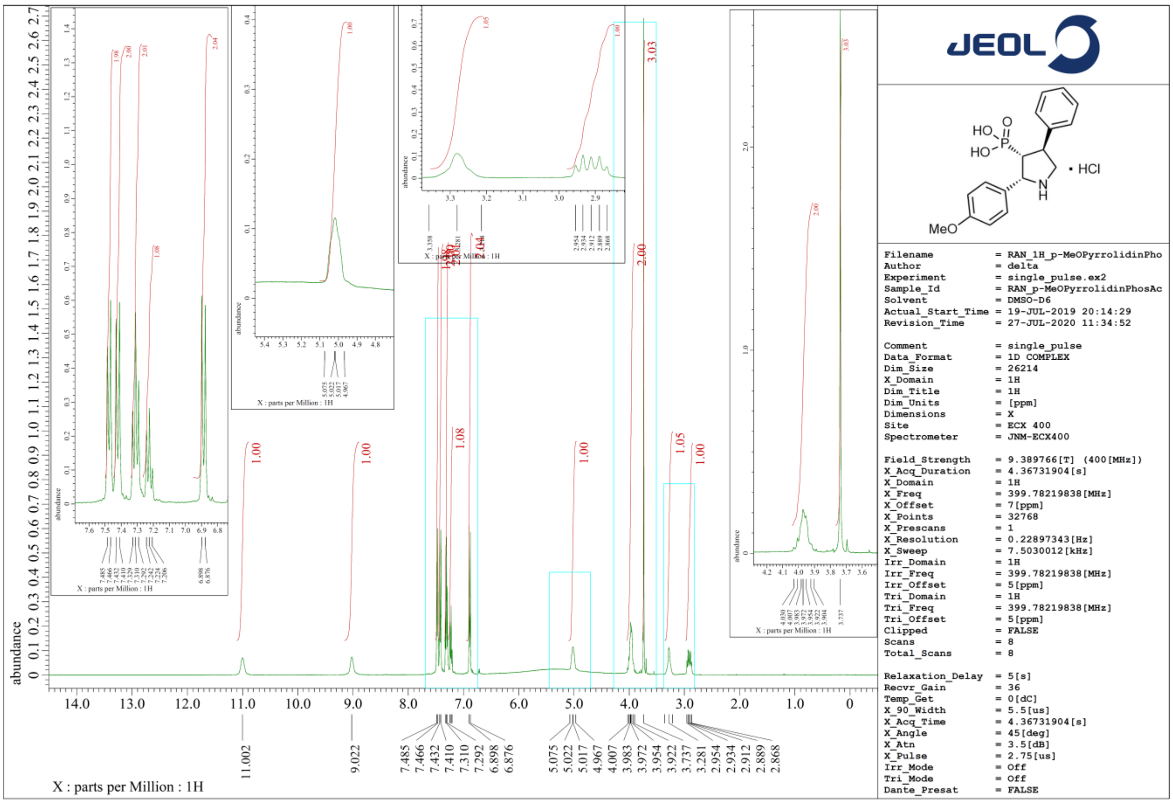

^{13}C NMR spectra of (2*R*,3*R*,4*S*)-2-(4-methoxyphenyl)-4-phenylpyrrolidin-3-ylphosphonic acid hydrochloride (**11b**) in DMSO-*d*6

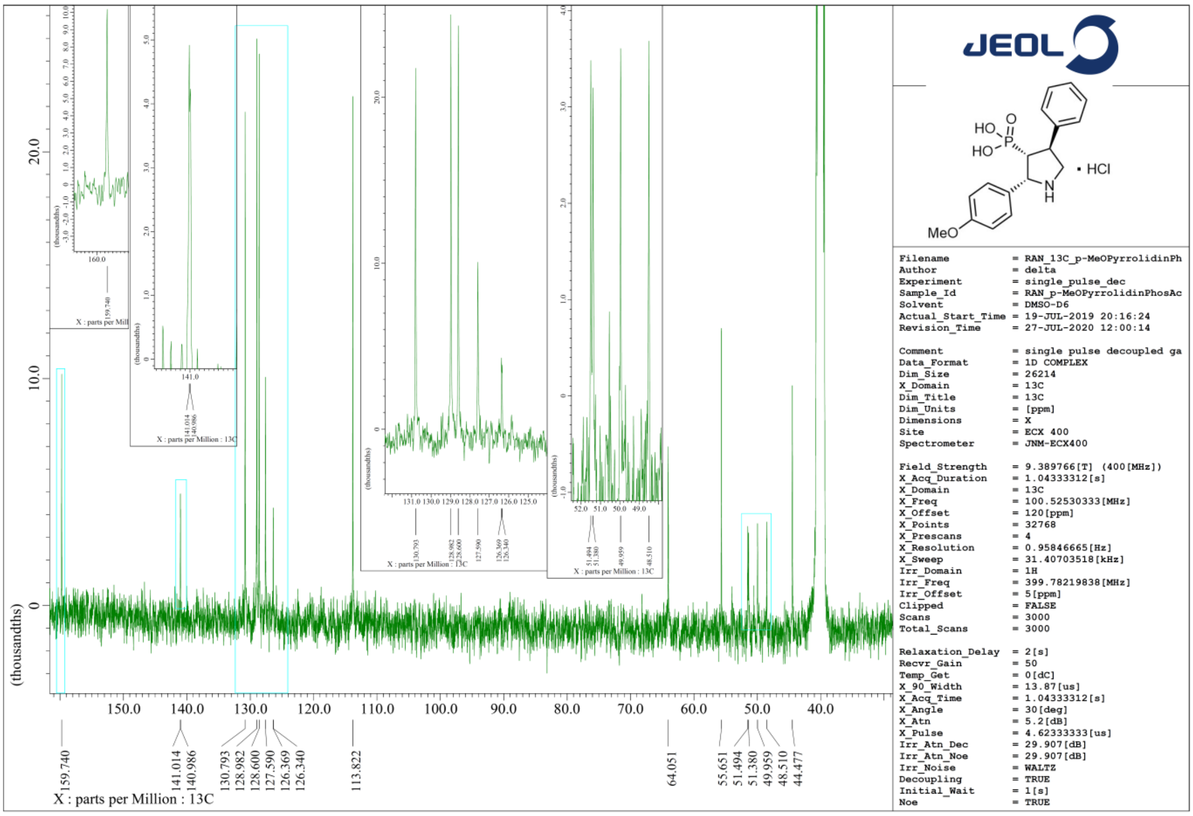

DEPT NMR spectra of (2*R*,3*R*,4*S*)-2-(4-methoxyphenyl)-4-phenylpyrrolidin-3-ylphosphonic acid hydrochloride (**11b**) in DMSO-*d*6

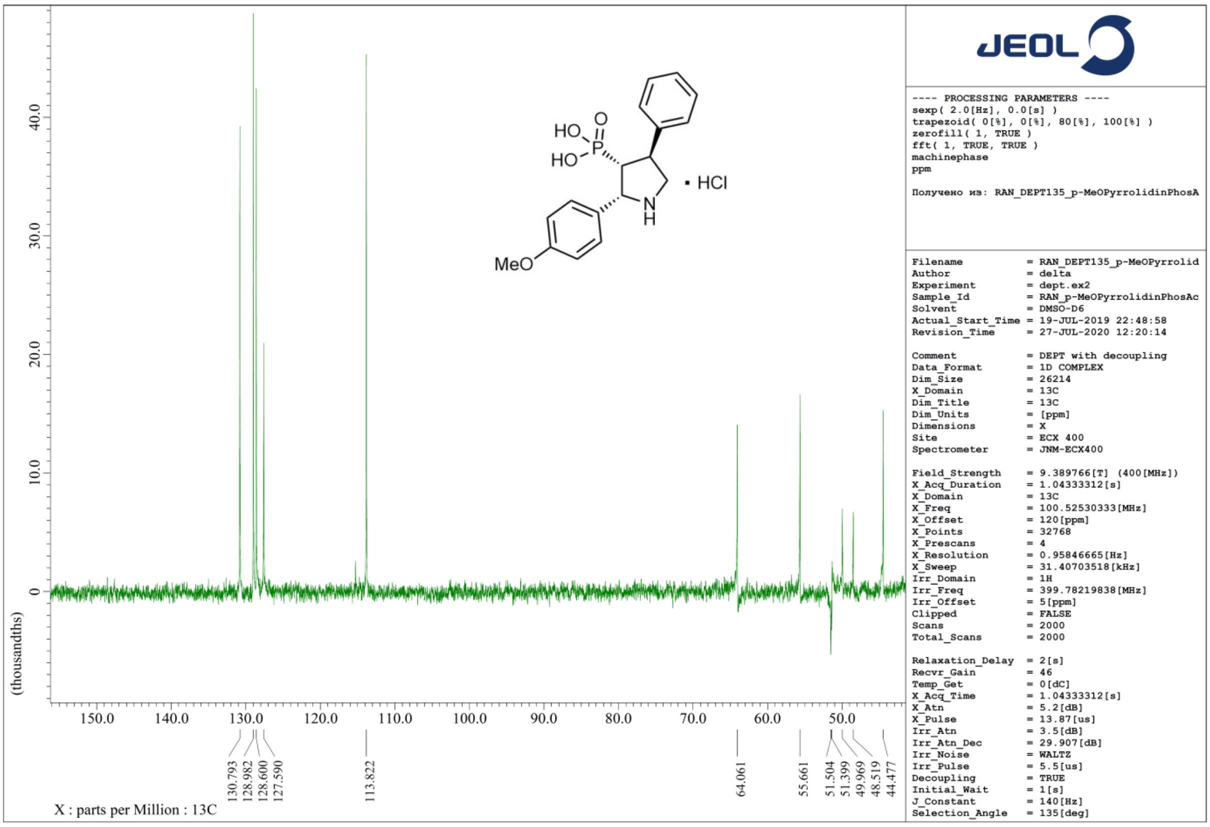

^{31}P NMR spectra of (2*R*,3*R*,4*S*)-2-(4-methoxyphenyl)-4-phenylpyrrolidin-3-ylphosphonic acid hydrochloride (**11b**) in DMSO-d^6

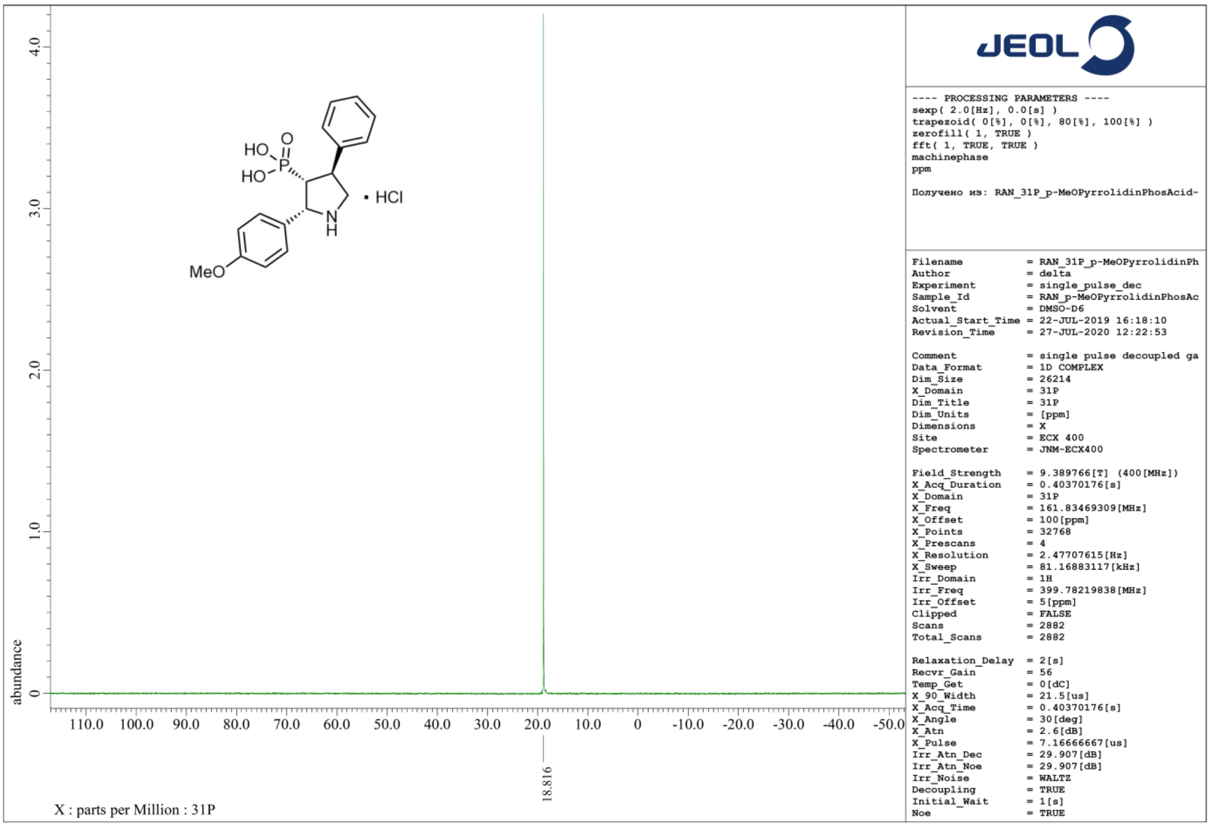

HRMS of (2*R*,3*R*,4*S*)-2-(4-methoxyphenyl)-4-phenylpyrrolidin-3-ylphosphonic acid hydrochloride (**11b**)

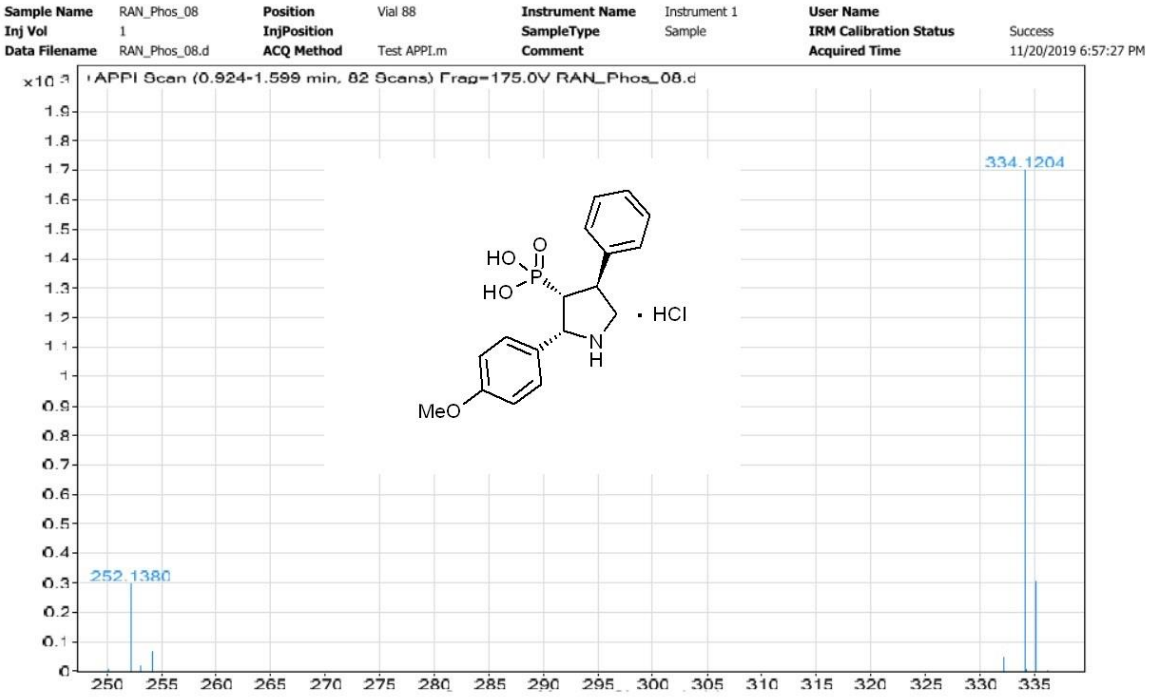

FTIR spectra of (2*R*,3*R*,4*S*)-2-(3-methoxyphenyl)-4-phenylpyrrolidin-3-ylphosphonic acid hydrochloride

(**11c**)

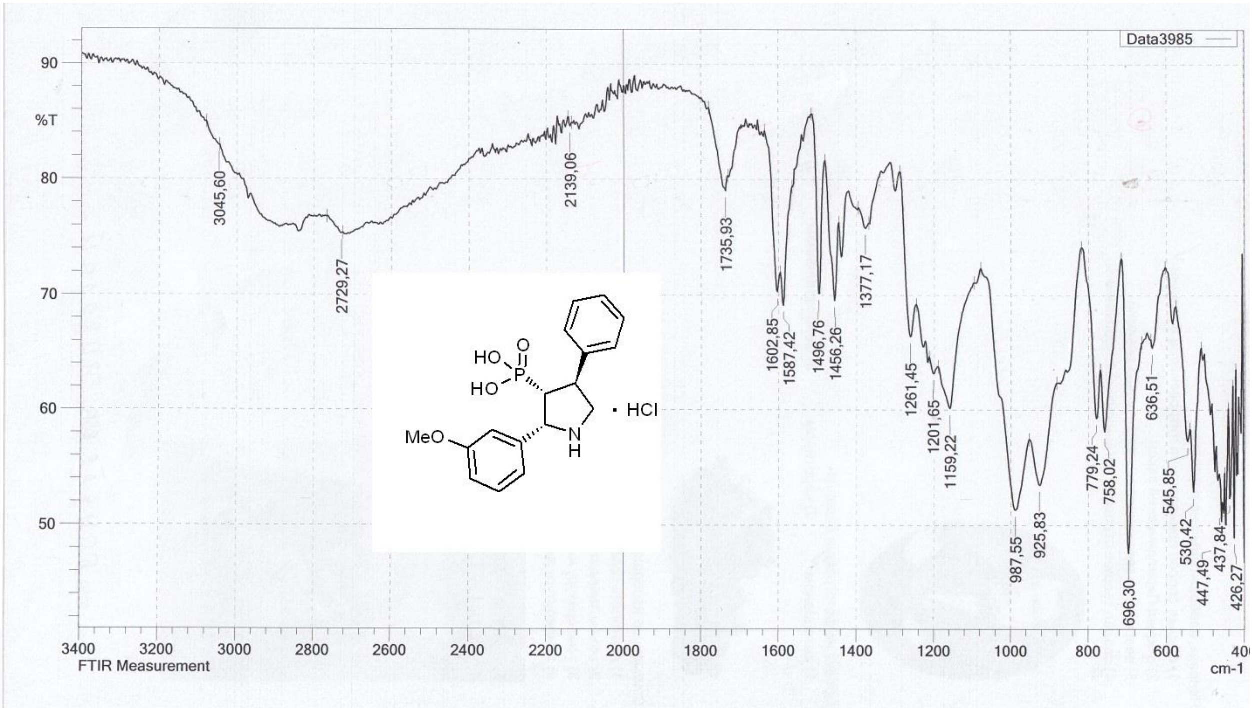

^{1}H NMR spectra of (2*R*,3*R*,4*S*)-2-(3-methoxyphenyl)-4-phenylpyrrolidin-3-ylphosphonic acid hydrochloride

(**11c**) in DMSO-*d*[6]

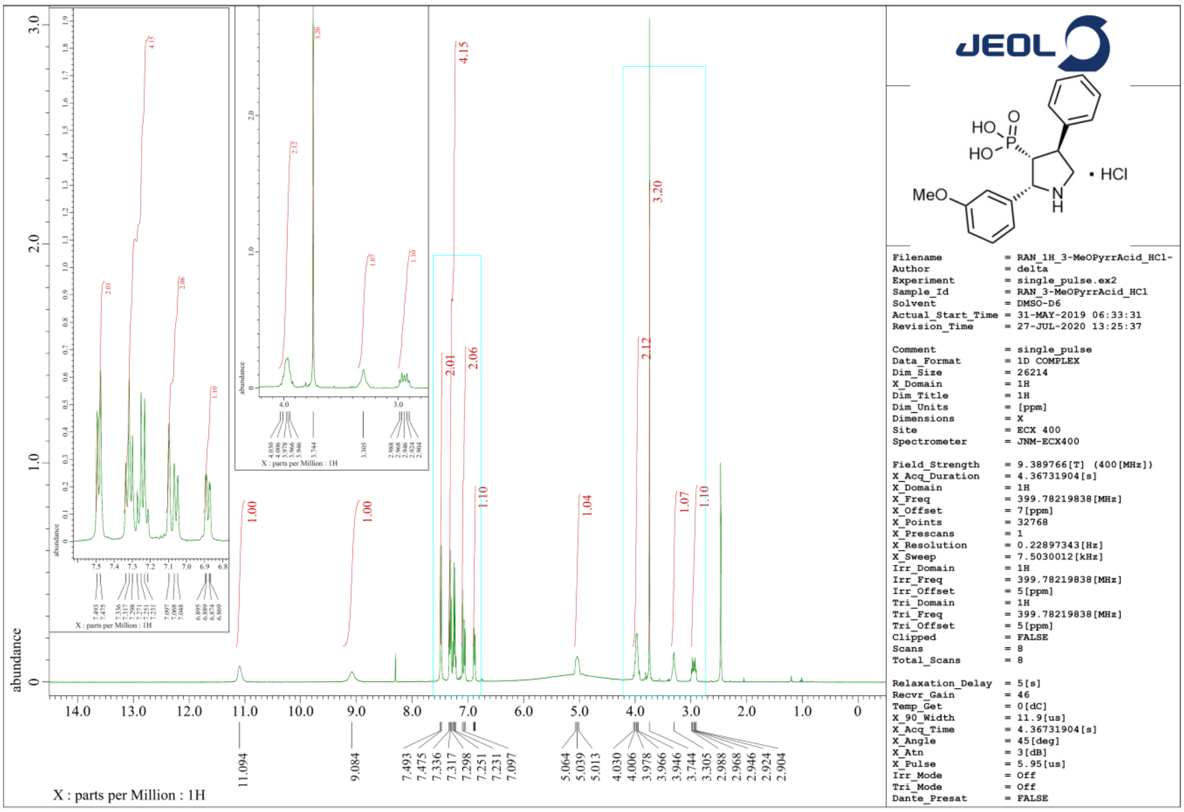

^{13}C NMR spectra of (2*R*,3*R*,4*S*)-2-(3-methoxyphenyl)-4-phenylpyrrolidin-3-ylphosphonic acid hydrochloride (**11c**) in DMSO-d^6

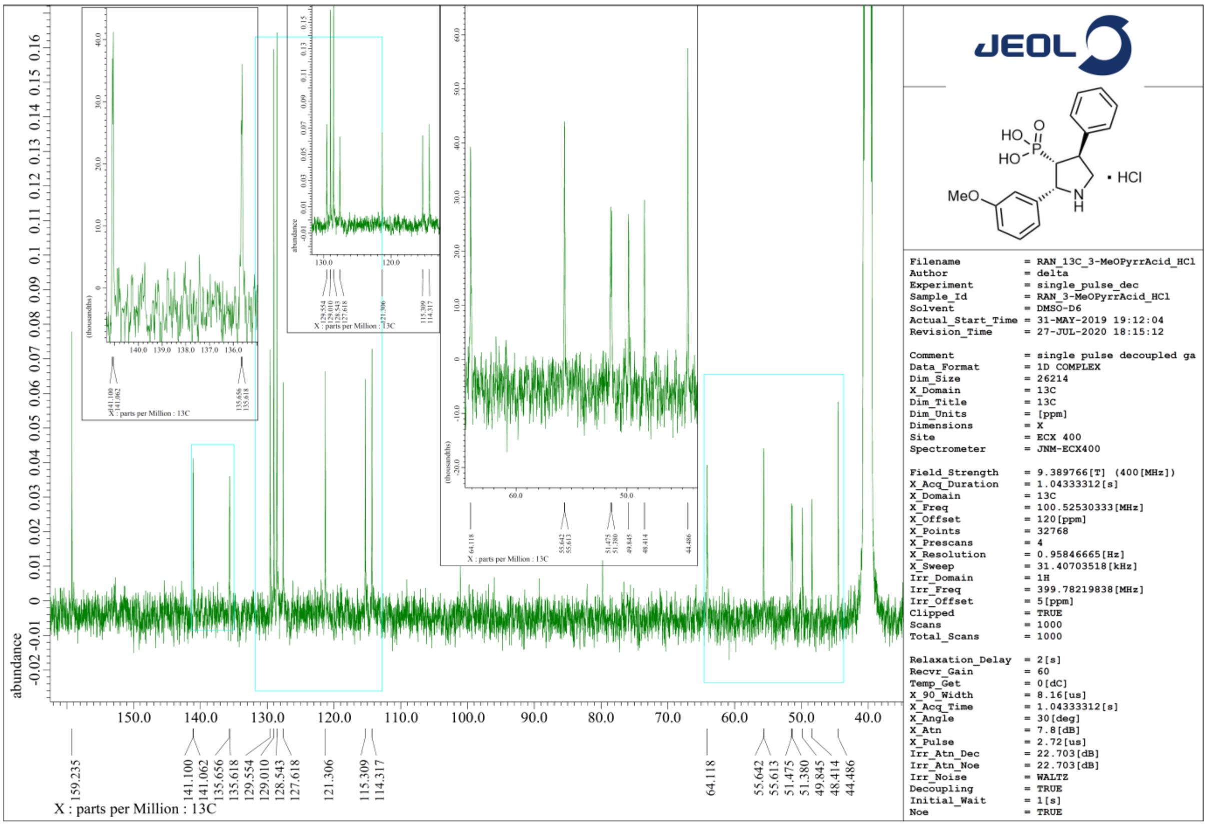

DEPT NMR spectra of (2*R*,3*R*,4*S*)-2-(3-methoxyphenyl)-4-phenylpyrrolidin-3-ylphosphonic acid hydrochloride (**11c**) in DMSO-d^6

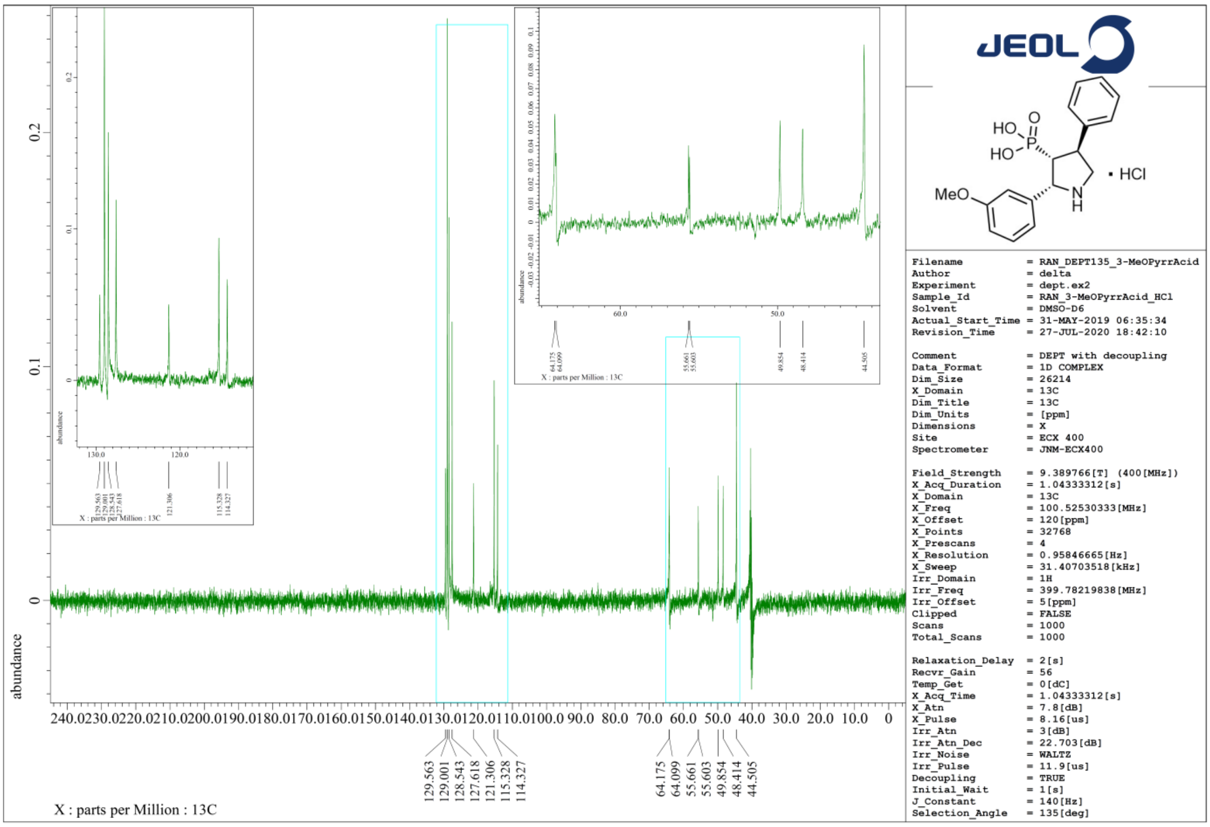

HMQC NMR spectra of (2*R*,3*R*,4*S*)-2-(3-methoxyphenyl)-4-phenylpyrrolidin-3-ylphosphonic acid hydrochloride (**11c**) in DMSO-*d*[6]

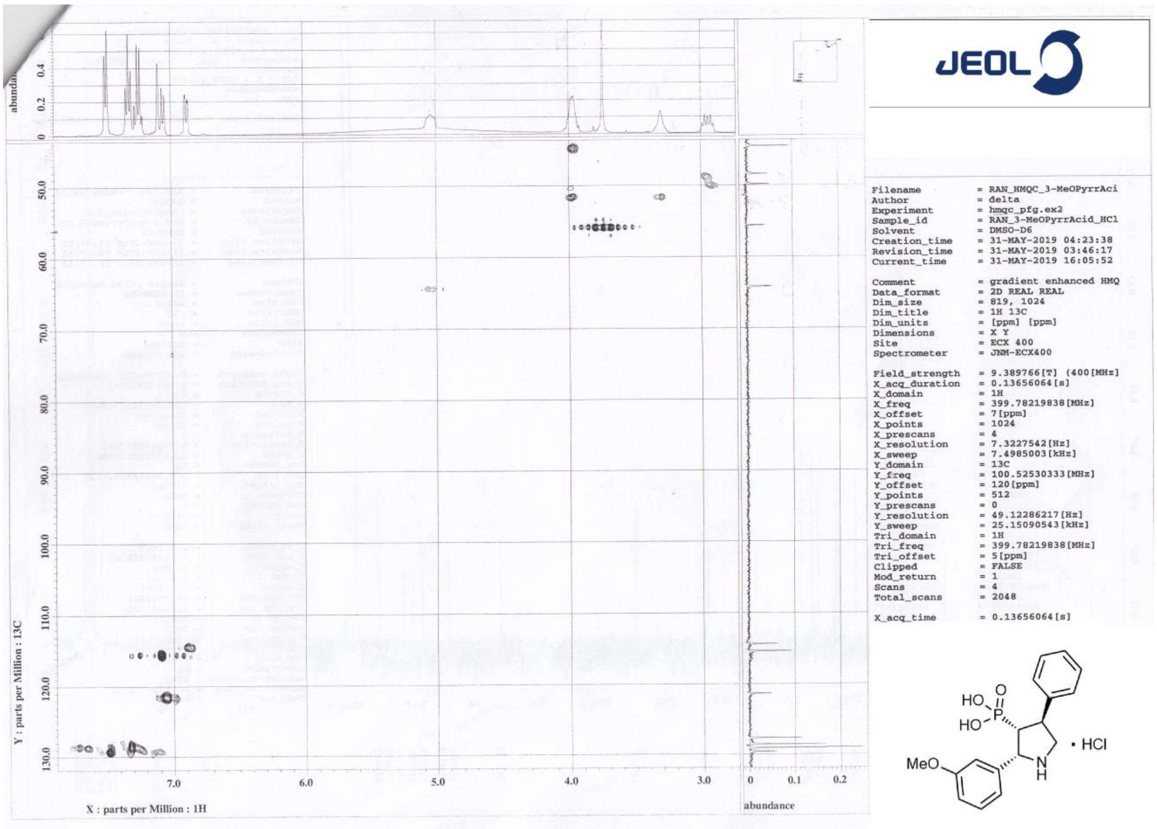

HMQC NMR spectra of (2*R*,3*R*,4*S*)-2-(3-methoxyphenyl)-4-phenylpyrrolidin-3-ylphosphonic acid hydrochloride (**11c**) in DMSO-*d*[6]

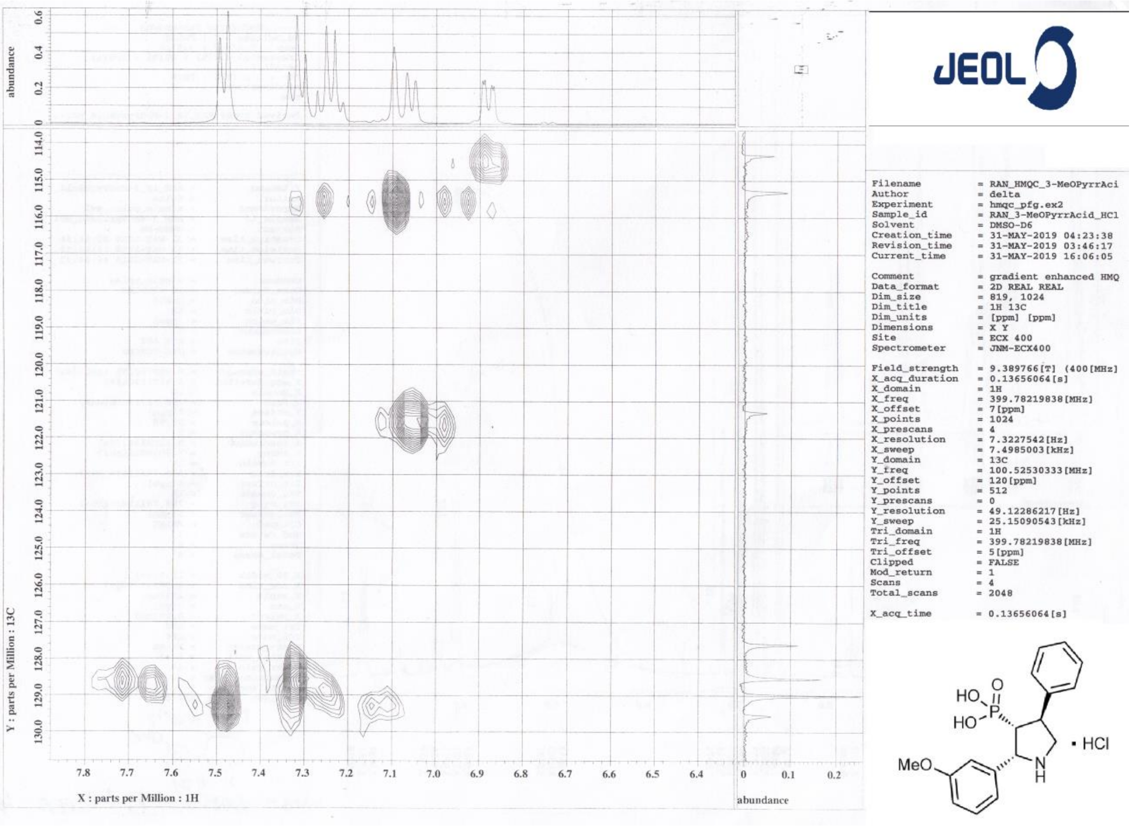

NOESY NMR spectra of (2*R*,3*R*,4*S*)-2-(3-methoxyphenyl)-4-phenylpyrrolidin-3-ylphosphonic acid hydrochloride (**11c**) in DMSO-*d*[6]

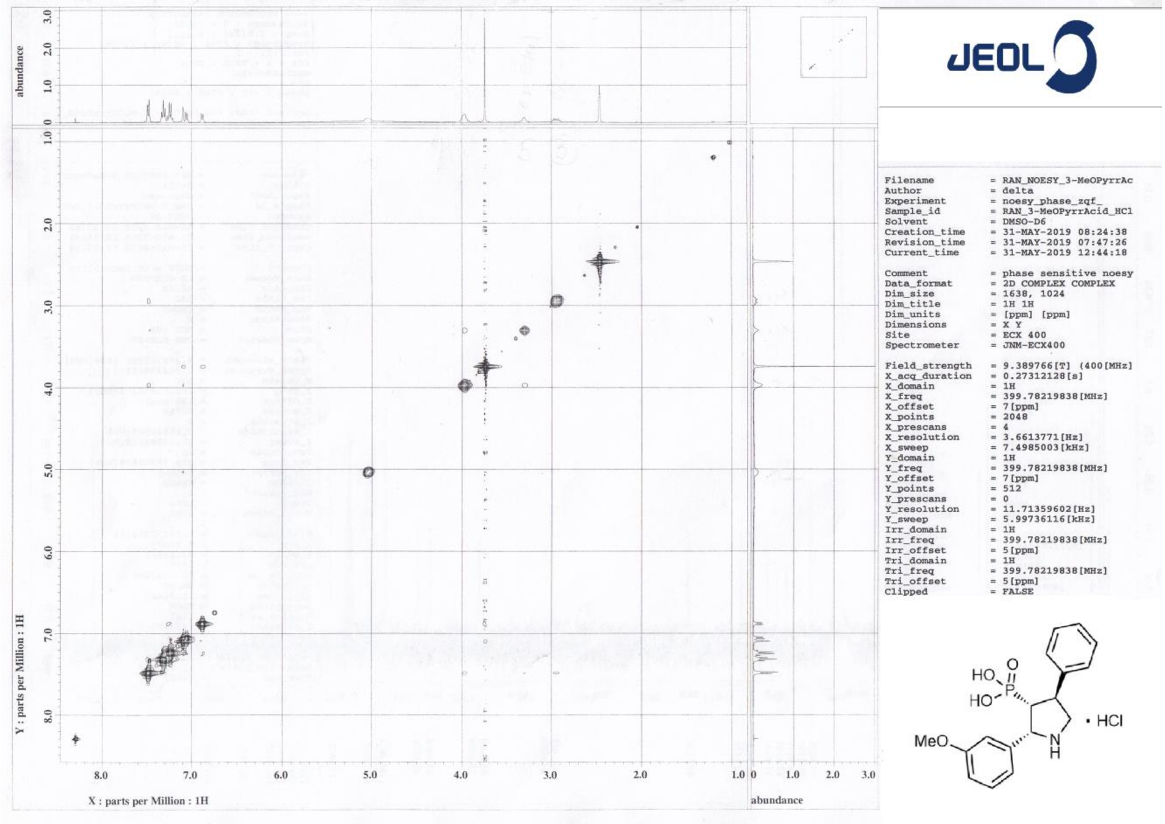

^{31}P NMR spectra of (2*R*,3*R*,4*S*)-2-(3-methoxyphenyl)-4-phenylpyrrolidin-3-ylphosphonic acid hydrochloride (**11c**) in DMSO-*d*[6]

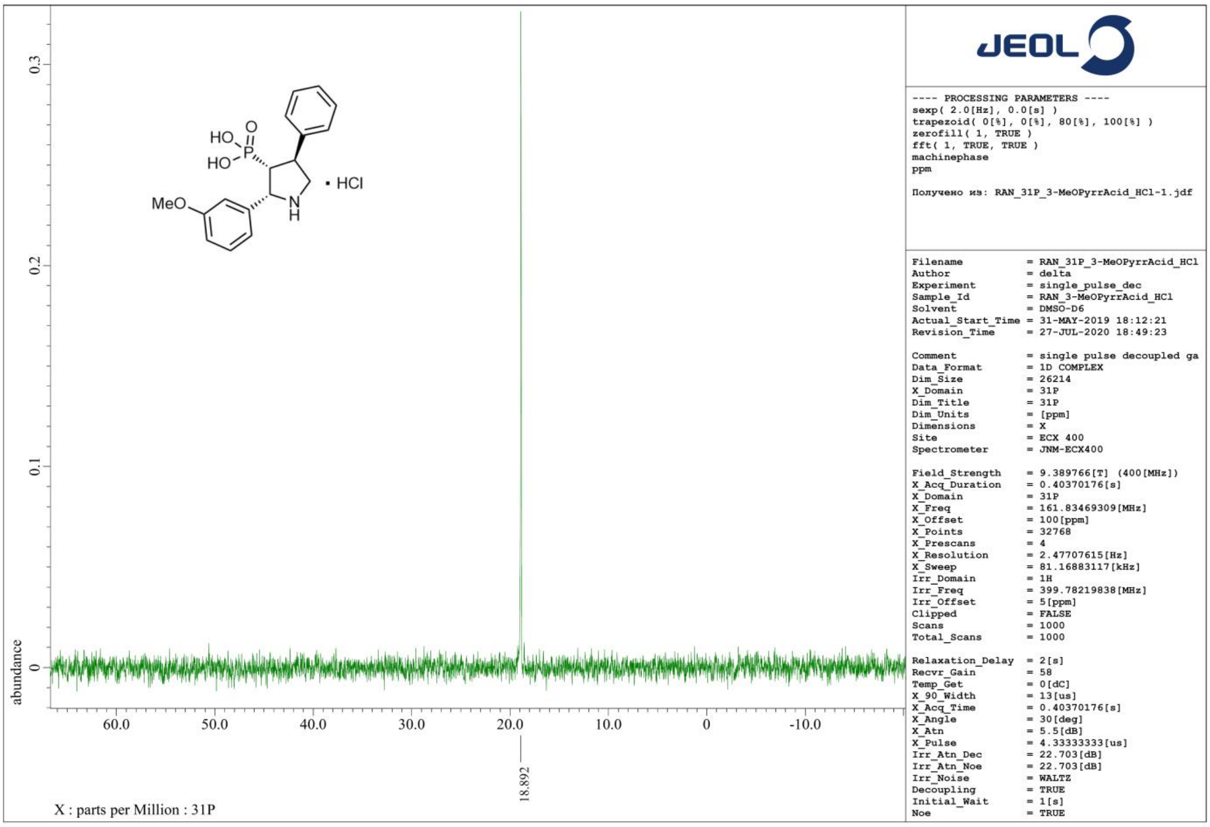

HRMS of (2*R*,3*R*,4*S*)-2-(3-methoxyphenyl)-4-phenylpyrrolidin-3-ylphosphonic acid hydrochloride (**11c**)

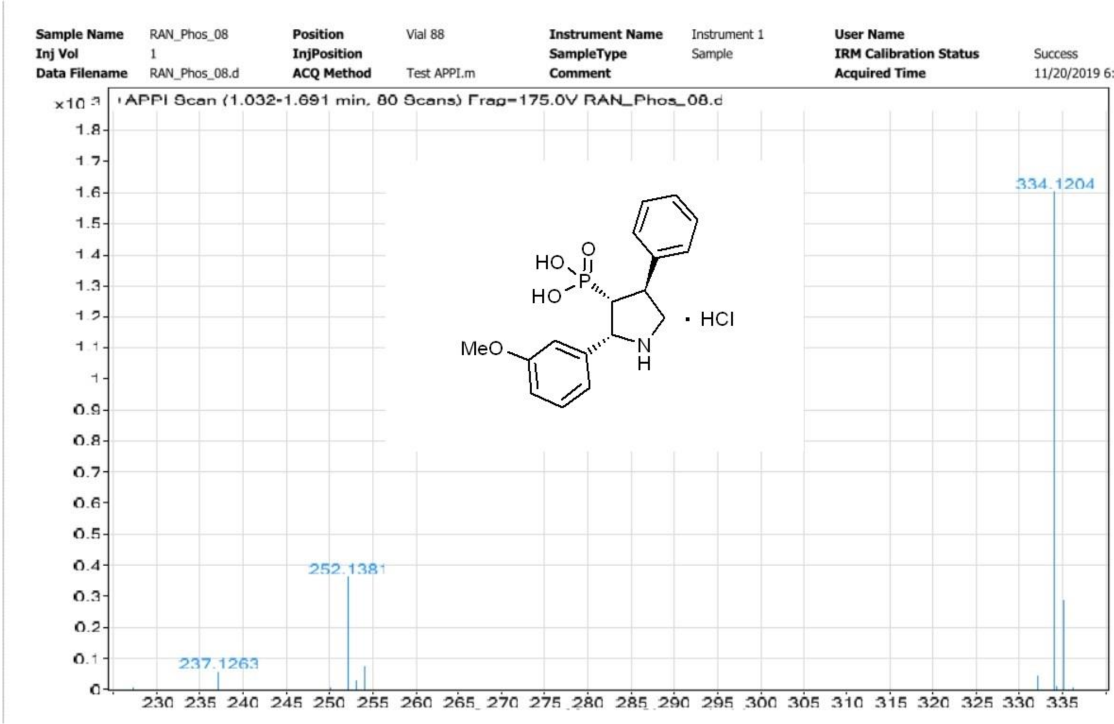

FTIR spectra of (2*R*,3*R*,4*S*)-2-methyl-4-phenylpyrrolidin-3-ylphosphonic acid hydrochloride (**11d**)

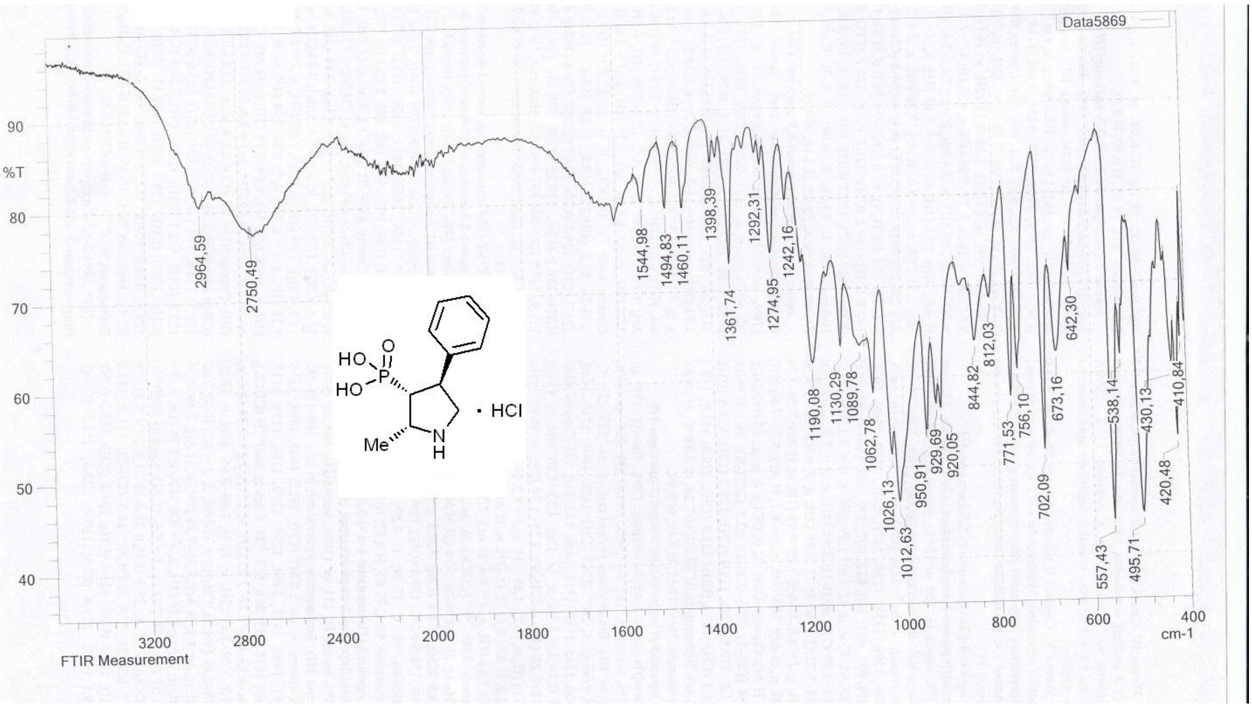

^{1}H NMR spectra of (2*R*,3*R*,4*S*)-2-methyl-4-phenylpyrrolidin-3-ylphosphonic acid hydrochloride (**11d**) in D_2O

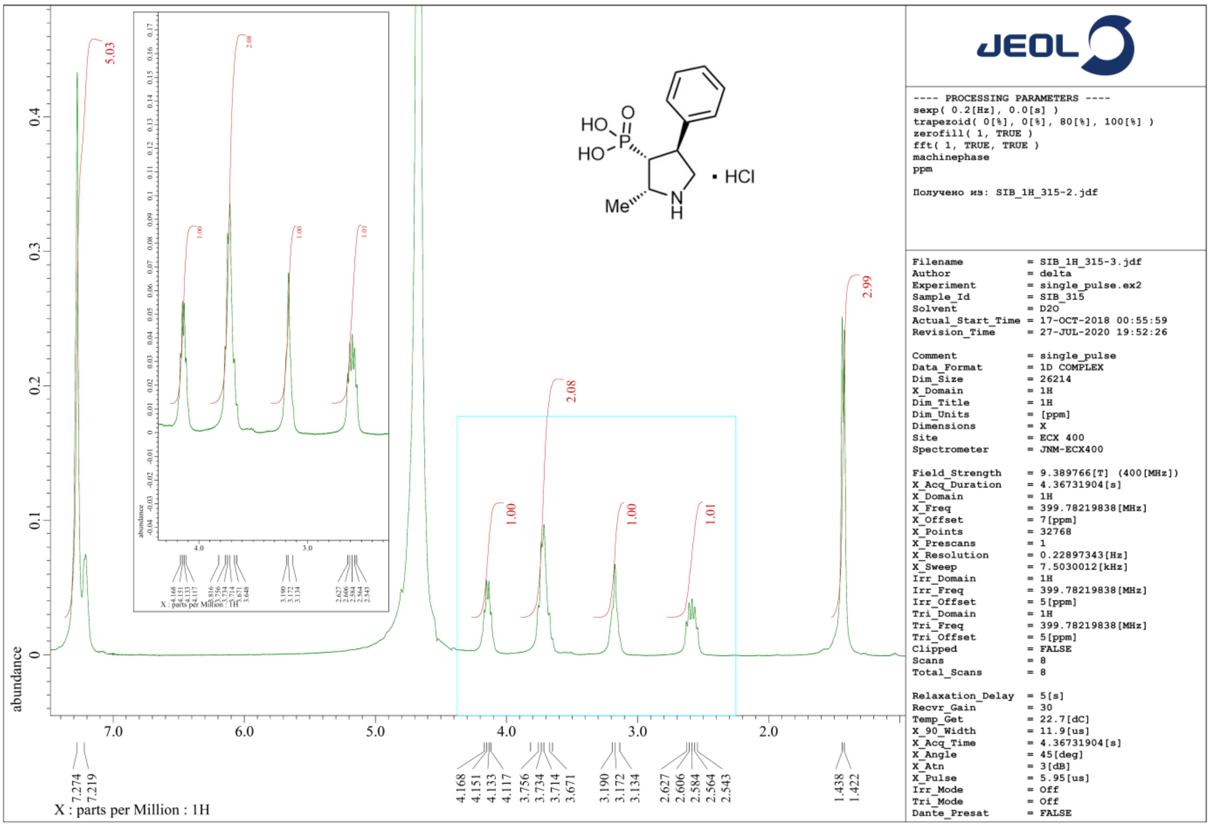

^{13}C NMR spectra of (2*R*,3*R*,4*S*)-2-methyl-4-phenylpyrrolidin-3-ylphosphonic acid hydrochloride (**11d**) in D_2O

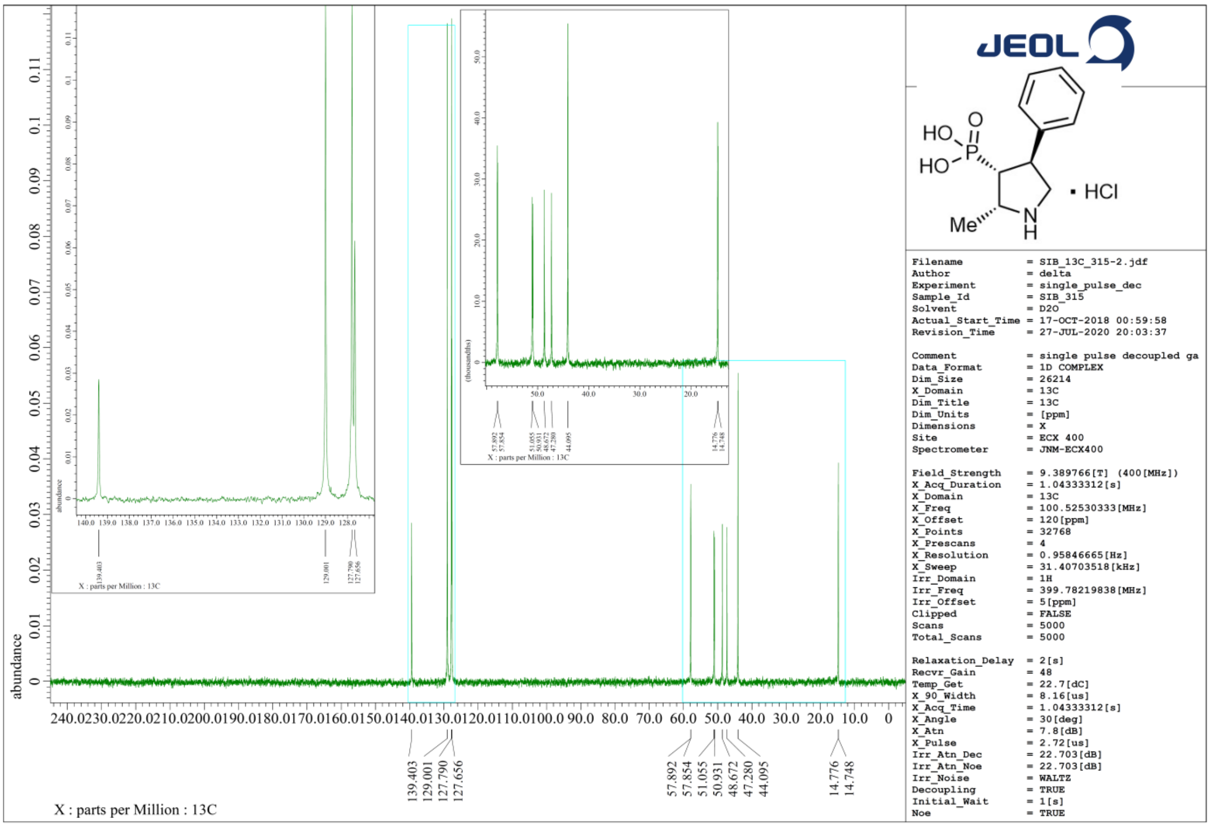

DEPT NMR spectra of (2*R*,3*R*,4*S*)-2-methyl-4-phenylpyrrolidin-3-ylphosphonic acid hydrochloride (**11d**) in D_2O

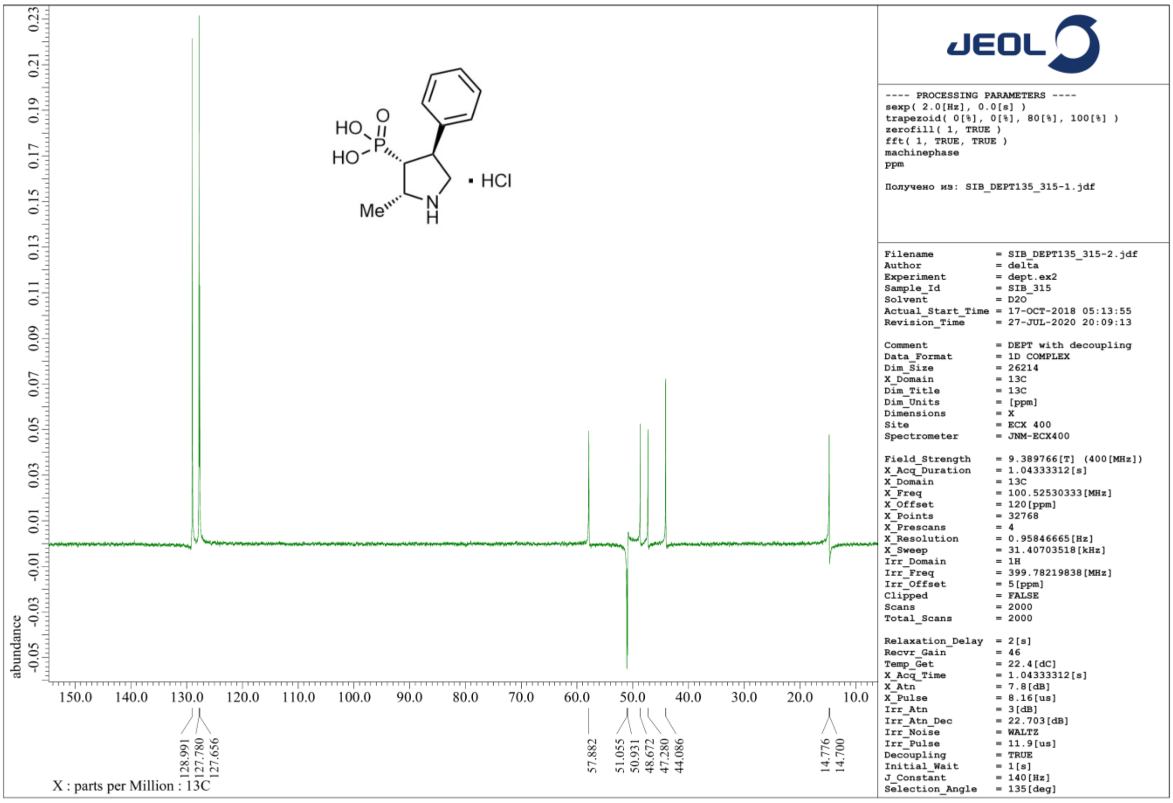

HMQC NMR spectra of (2*R*,3*R*,4*S*)-2-methyl-4-phenylpyrrolidin-3-ylphosphonic acid hydrochloride (**11d**) in D_2O

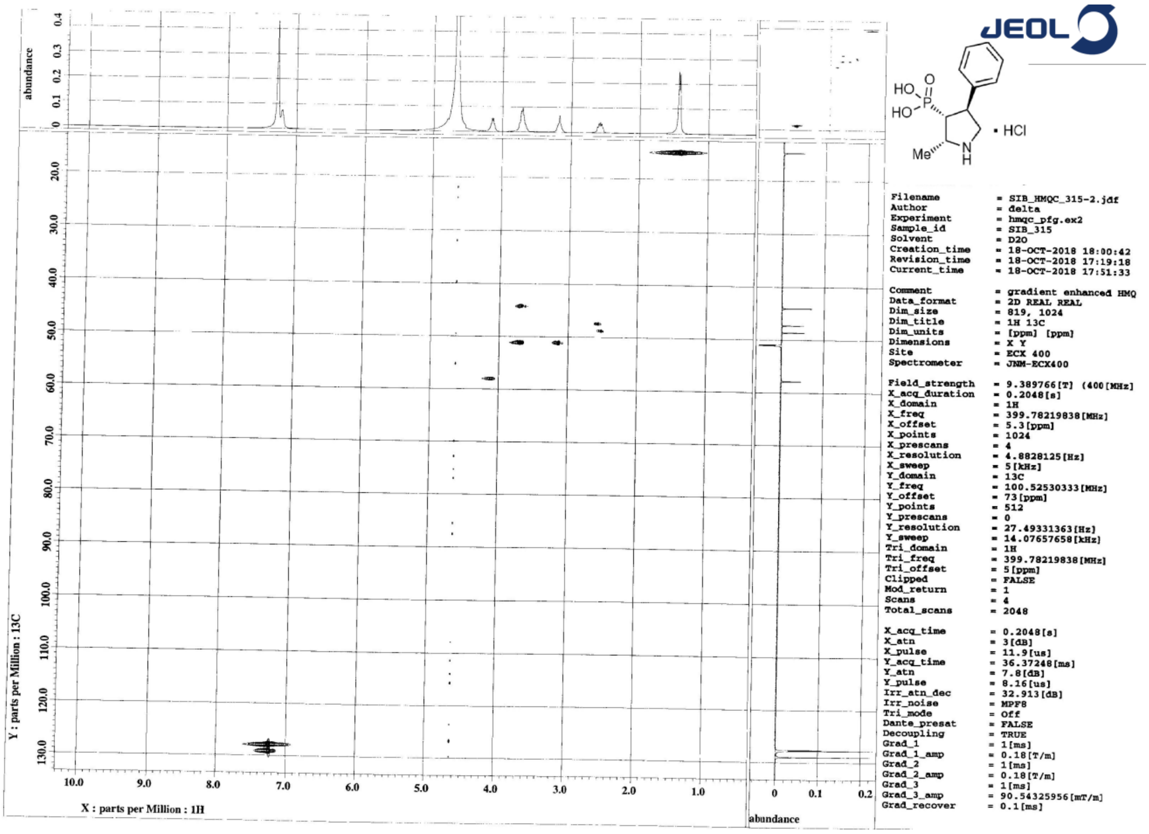

HMQC NMR spectra of (2*R*,3*R*,4*S*)-2-methyl-4-phenylpyrrolidin-3-ylphosphonic acid hydrochloride (**11d**) in D_2O

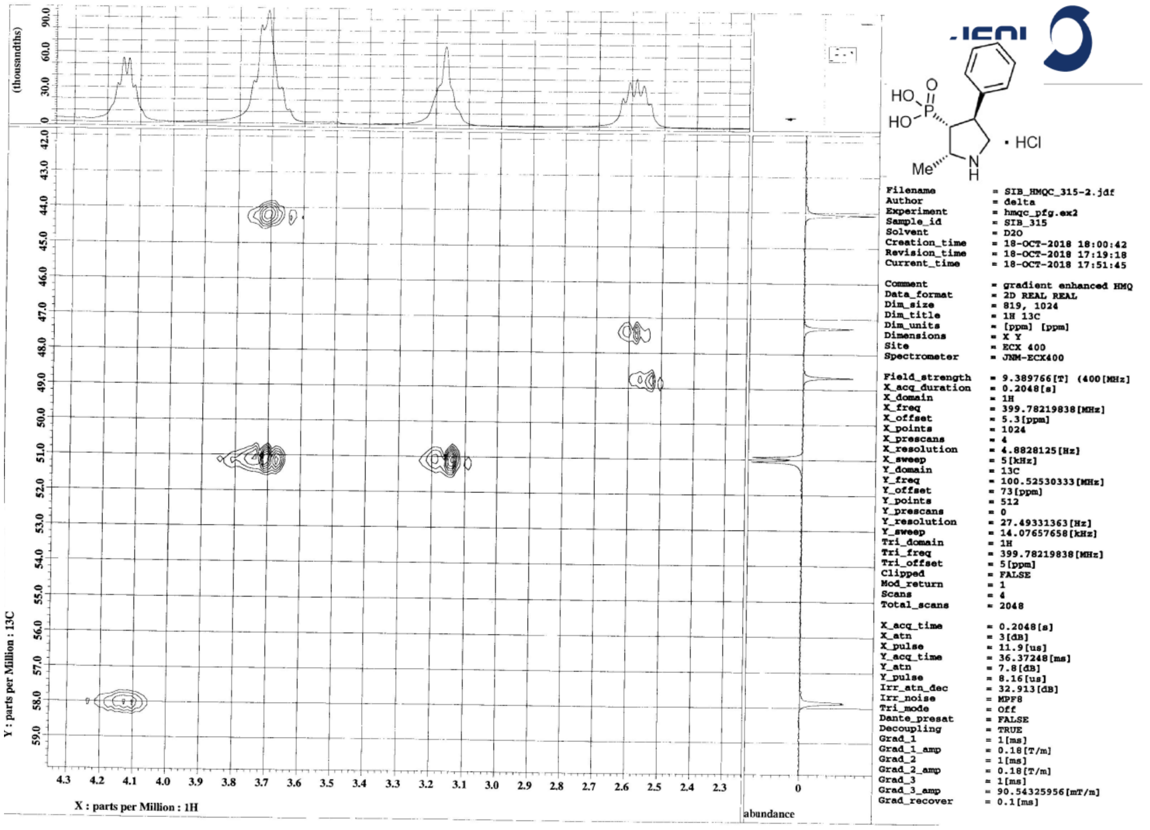

^{31}P NMR spectra of (2*R*,3*R*,4*S*)-2-methyl-4-phenylpyrrolidin-3-ylphosphonic acid hydrochloride (**11d**) in D_2O

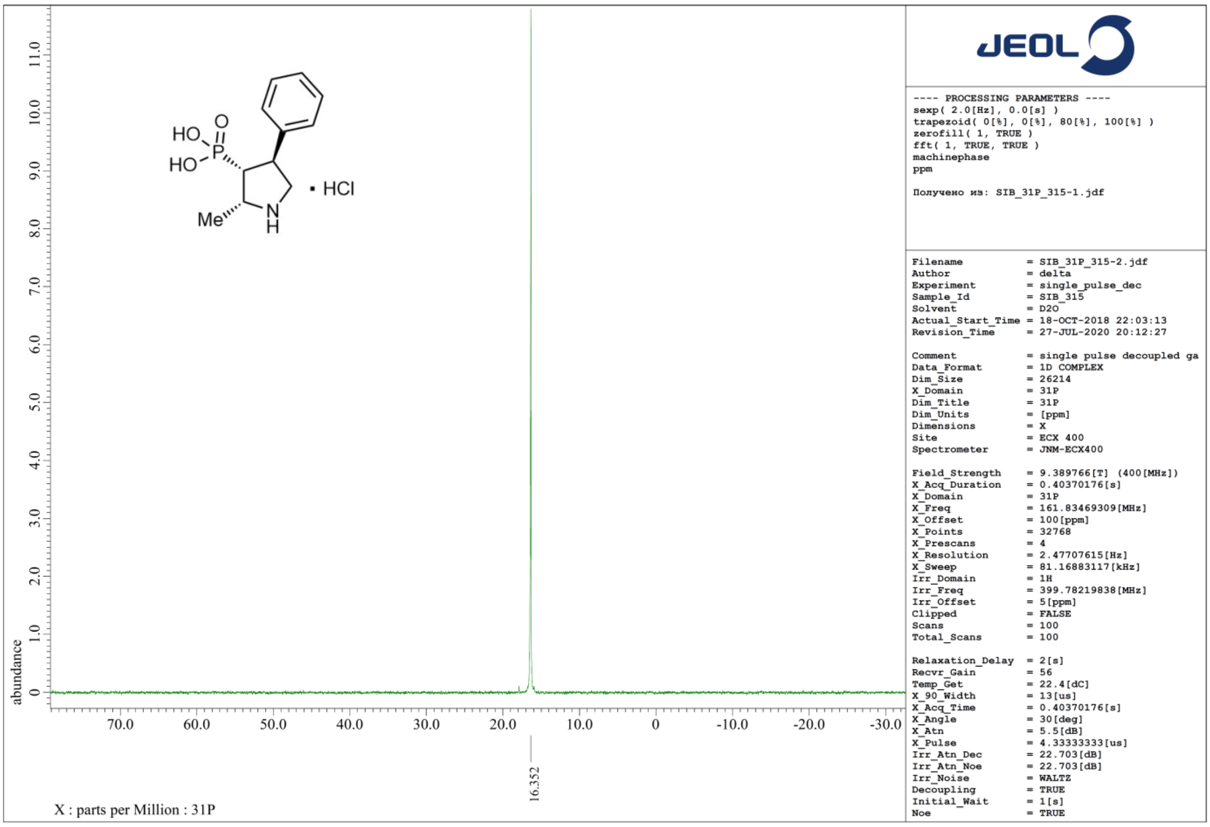

HRMS of (2*R*,3*R*,4*S*)-2-methyl-4-phenylpyrrolidin-3-ylphosphonic acid hydrochloride (**11d**)

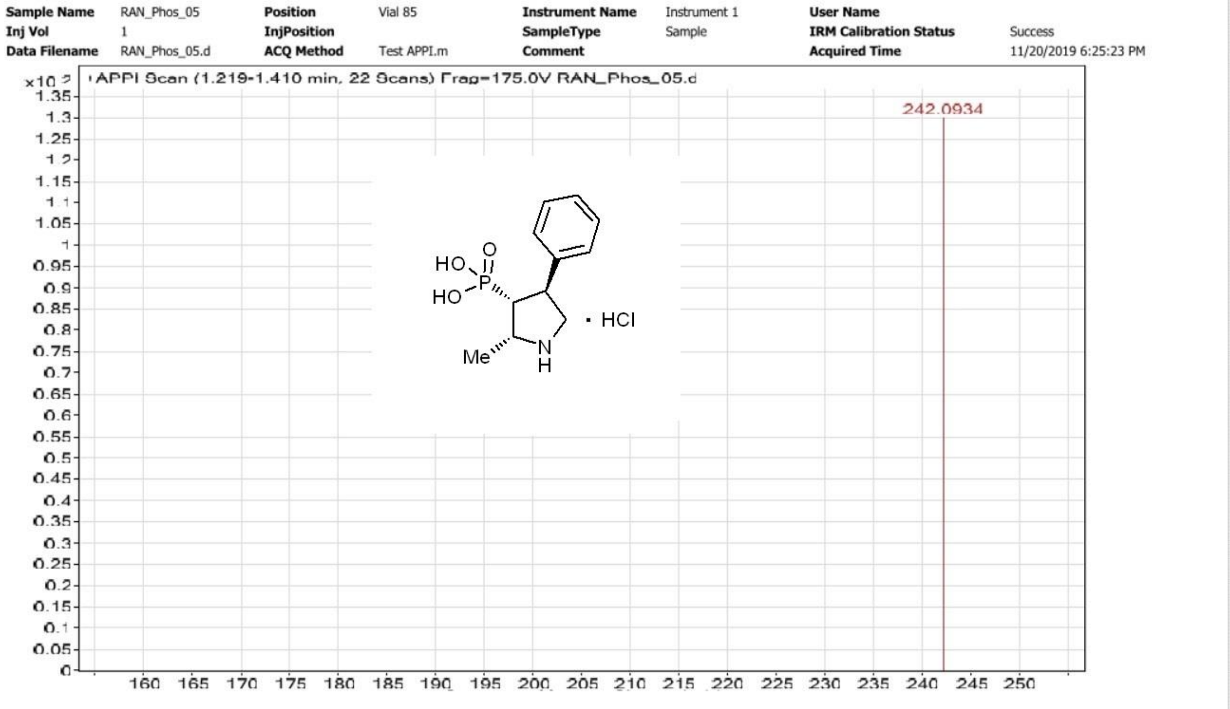

FTIR spectra of dimethyl [(2*S*,3*R*,4*S*,5*S*,6*R*)-6-ethyl-2-hydroxy-2-methyl-5-nitro-4-phenyltetrahydro-2*H*-pyran-3-yl)phosphonate (**13a**)

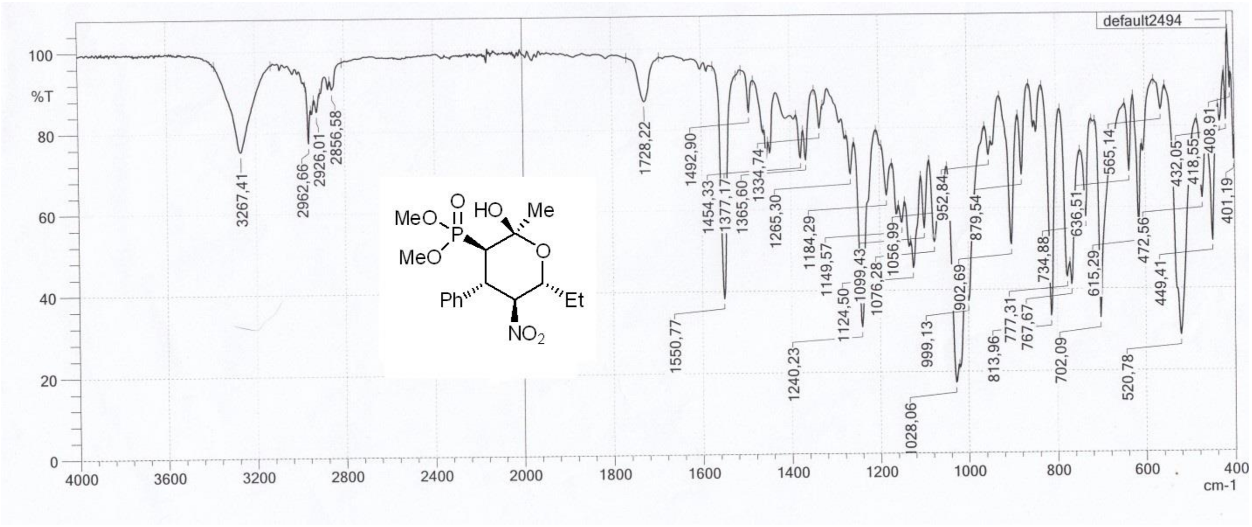

^{1}H NMR spectra of dimethyl [(2*S*,3*R*,4*S*,5*S*,6*R*)-6-ethyl-2-hydroxy-2-methyl-5-nitro-4-phenyltetrahydro-2*H*-pyran-3-yl)phosphonate (**13a**) in $CDCl_3$

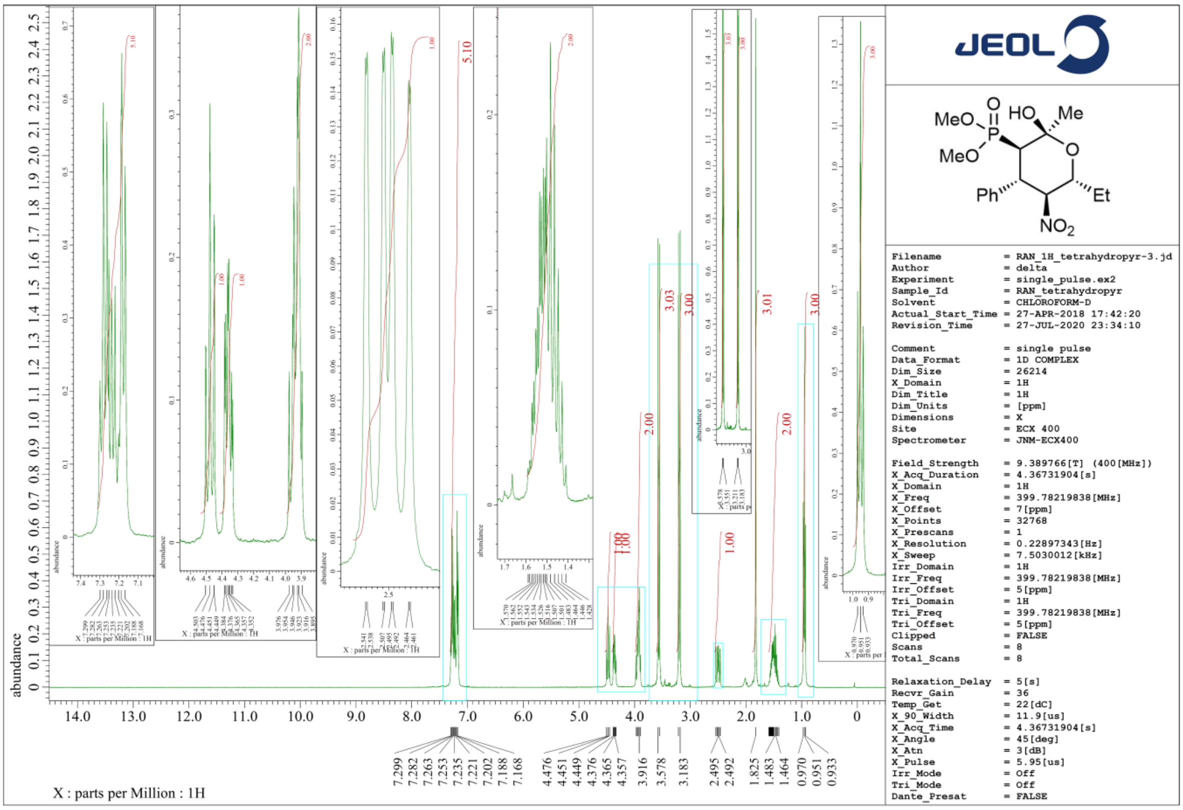

^{13}C NMR spectra of dimethyl [(2*S*,3*R*,4*S*,5*S*,6*R*)-6-ethyl-2-hydroxy-2-methyl-5-nitro-4-phenyltetrahydro-2*H*-pyran-3-yl)phosphonate (**13a**) in $CDCl_3$

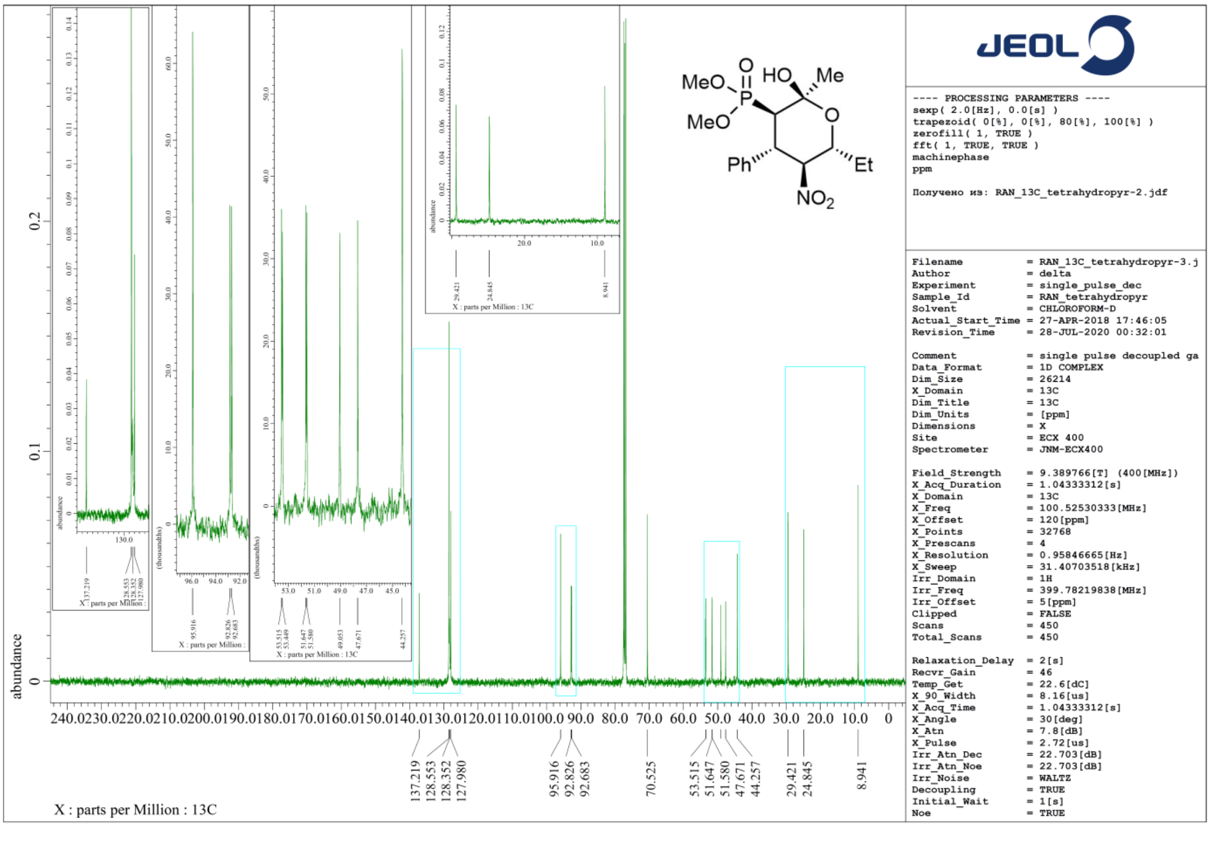

DEPT NMR spectra of dimethyl [(2*S*,3*R*,4*S*,5*S*,6*R*)-6-ethyl-2-hydroxy-2-methyl-5-nitro-4-phenyltetrahydro-2*H*-pyran-3-yl)phosphonate (**13a**) in $CDCl_3$

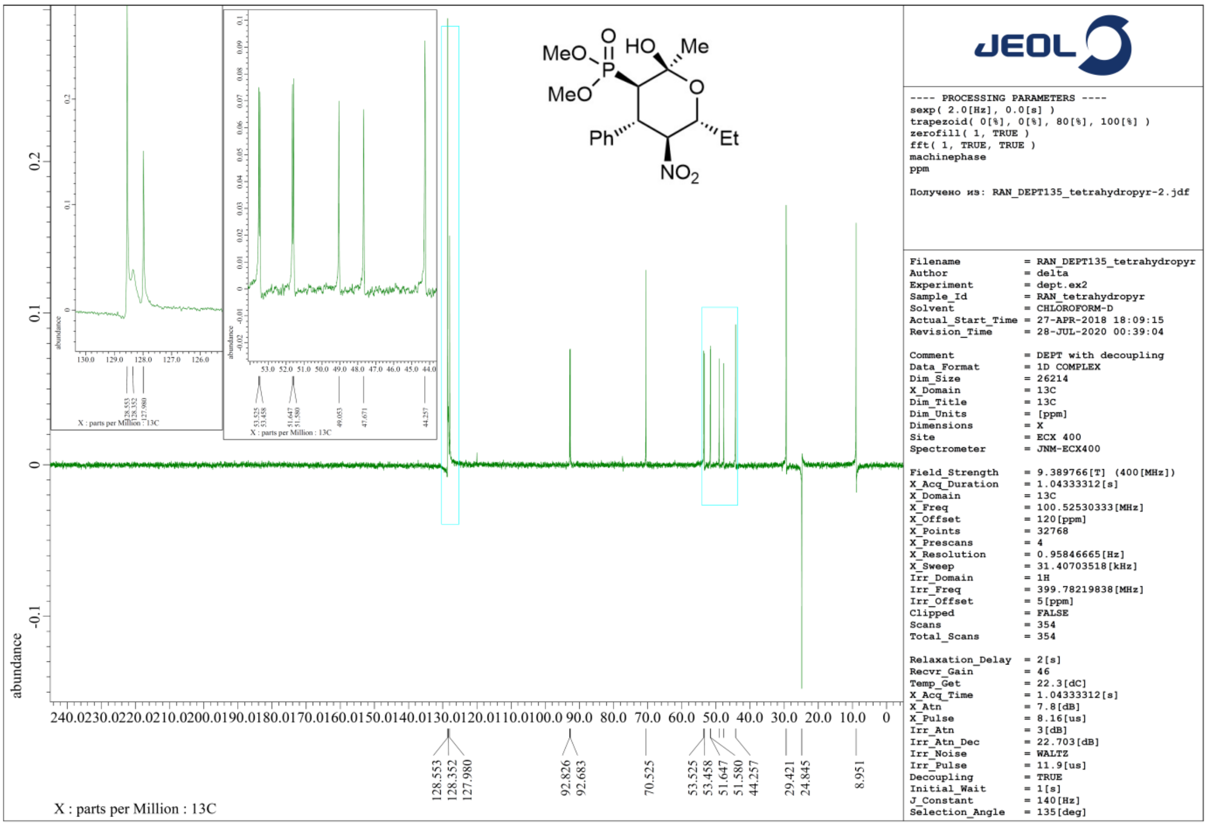

^{31}P NMR spectra of dimethyl [(2*S*,3*R*,4*S*,5*S*,6*R*)-6-ethyl-2-hydroxy-2-methyl-5-nitro-4-phenyltetrahydro-2*H*-pyran-3-yl)phosphonate (**13a**) in $CDCl_3$

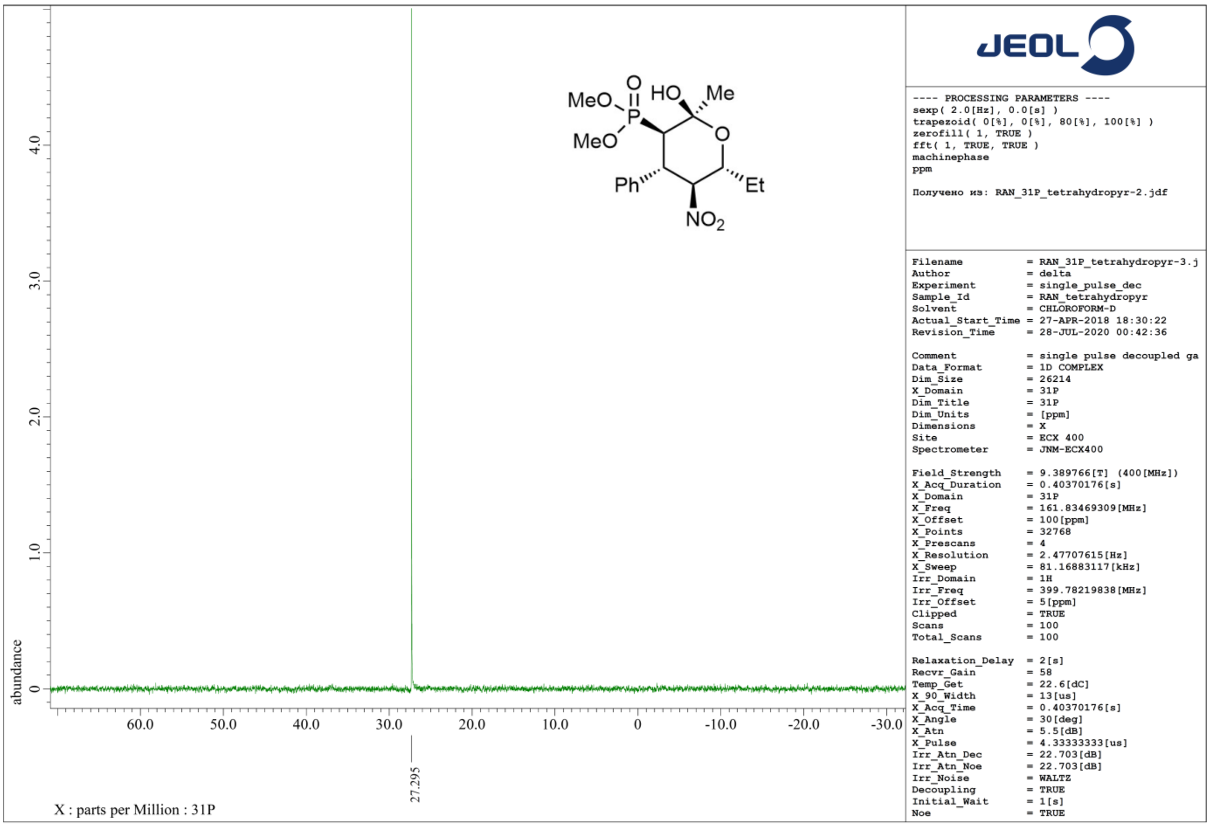

HRMS of dimethyl [(2*S*,3*R*,4*S*,5*S*,6*R*)-6-ethyl-2-hydroxy-2-methyl-5-nitro-4-phenyltetrahydro-2*H*-pyran-3-yl)phosphonate (**13a**)

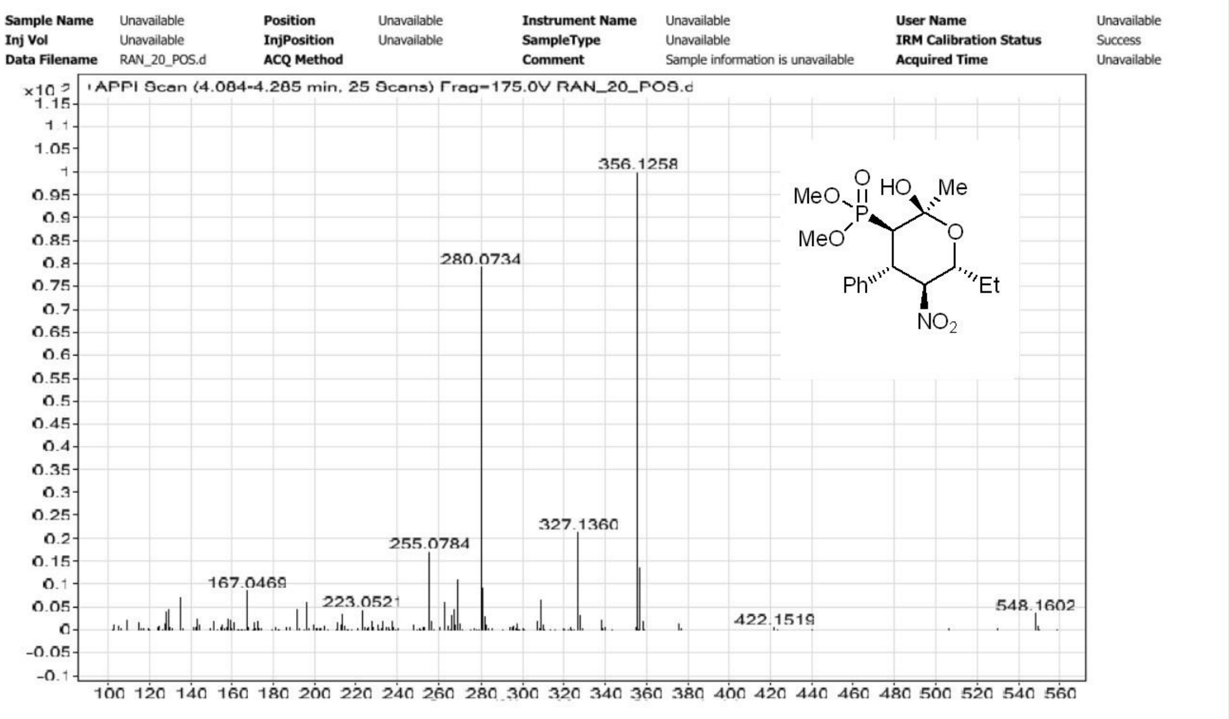

FTIR spectra of dimethyl [(2*S*,3*R*,4*S*,5*S*,6*R*)-2-hydroxy-2,6-dimethyl-5-nitro-4-phenyltetrahydro-2*H*-pyran-3-yl]phosphonate (**13b**)

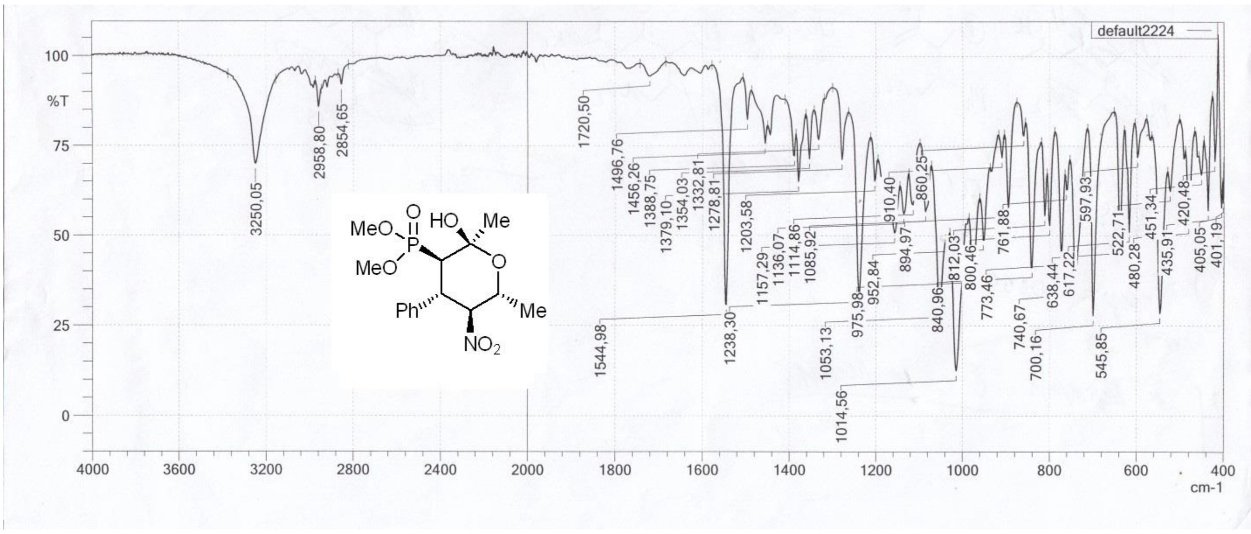

^{1}H NMR spectra of dimethyl [(2*S*,3*R*,4*S*,5*S*,6*R*)-2-hydroxy-2,6-dimethyl-5-nitro-4-phenyltetrahydro-2*H*-pyran-3-yl]phosphonate (**13b**) in $CDCl_3$

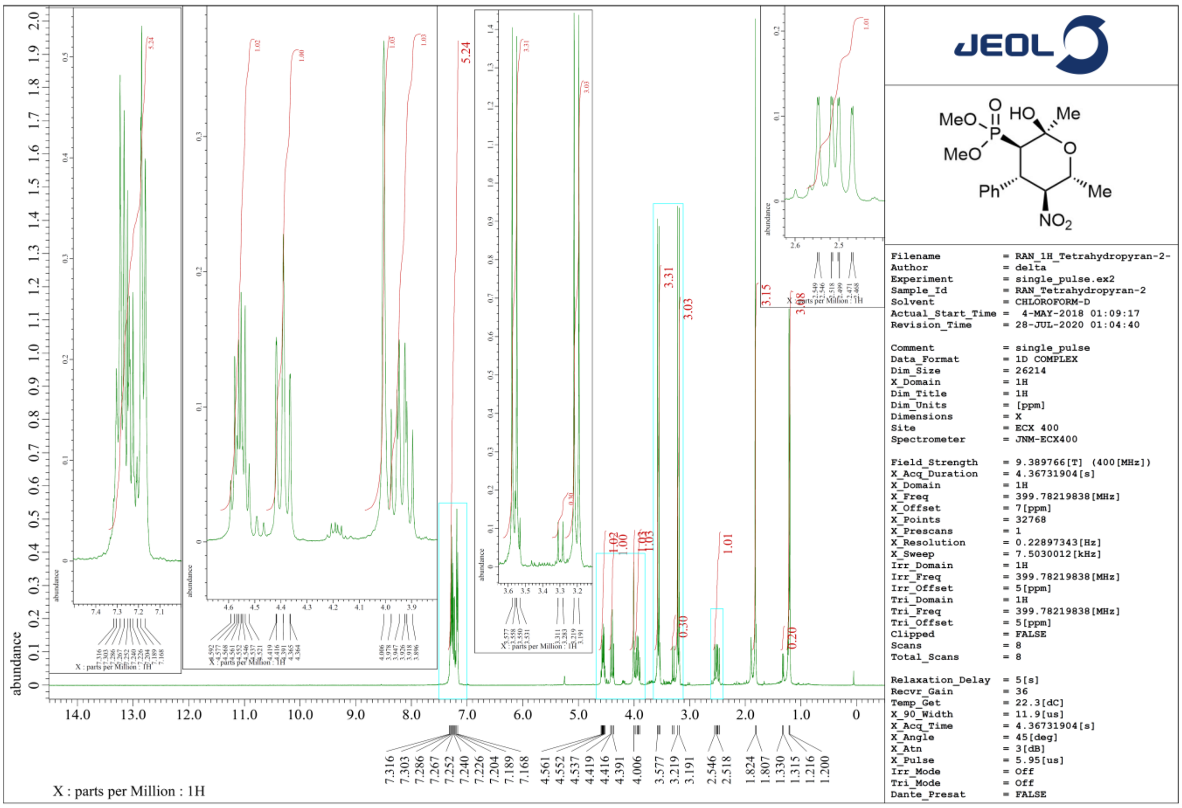

^{13}C NMR spectra of dimethyl [(2*S*,3*R*,4*S*,5*S*,6*R*)-2-hydroxy-2,6-dimethyl-5-nitro-4-phenyltetrahydro-2*H*-pyran-3-yl]phosphonate (**13b**) in $CDCl_3$

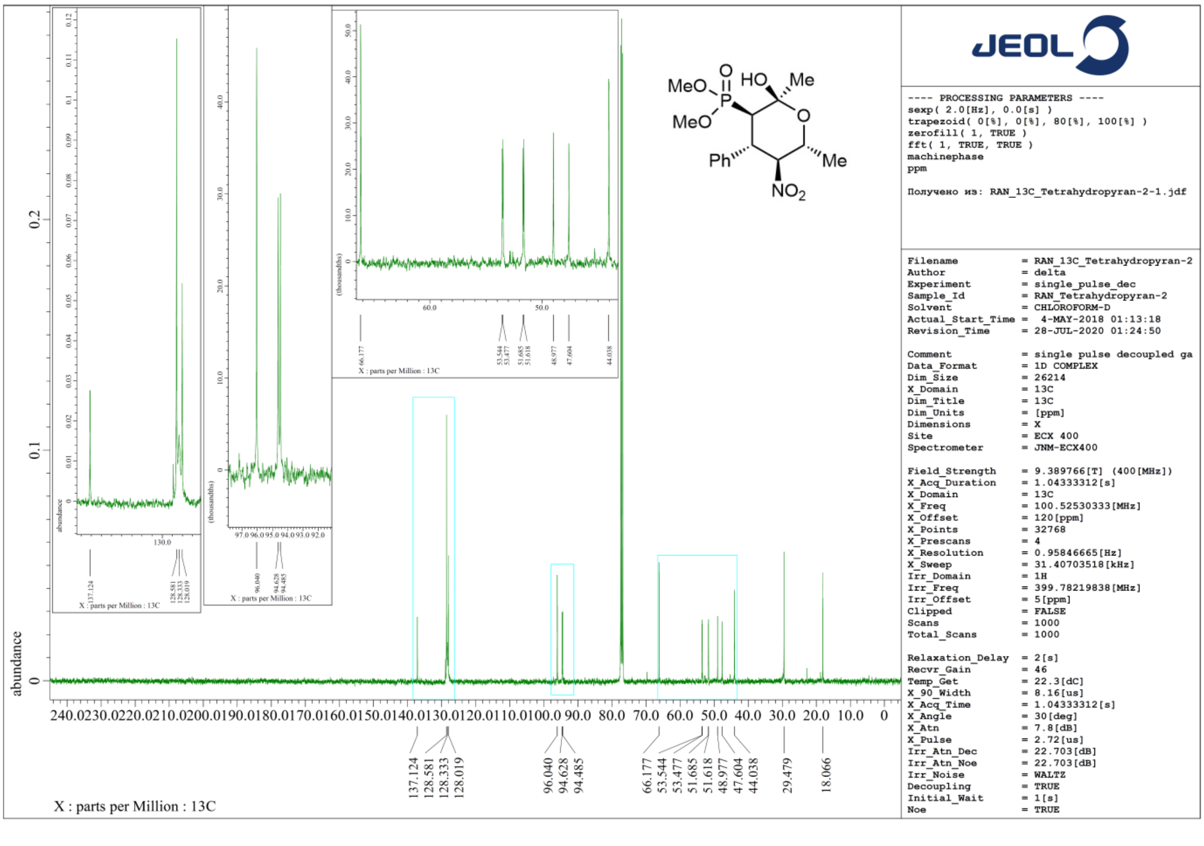

DEPT NMR spectra of dimethyl [(2*S*,3*R*,4*S*,5*S*,6*R*)-2-hydroxy-2,6-dimethyl-5-nitro-4-phenyltetrahydro-2*H*-pyran-3-yl]phosphonate (**13b**) in $CDCl_3$

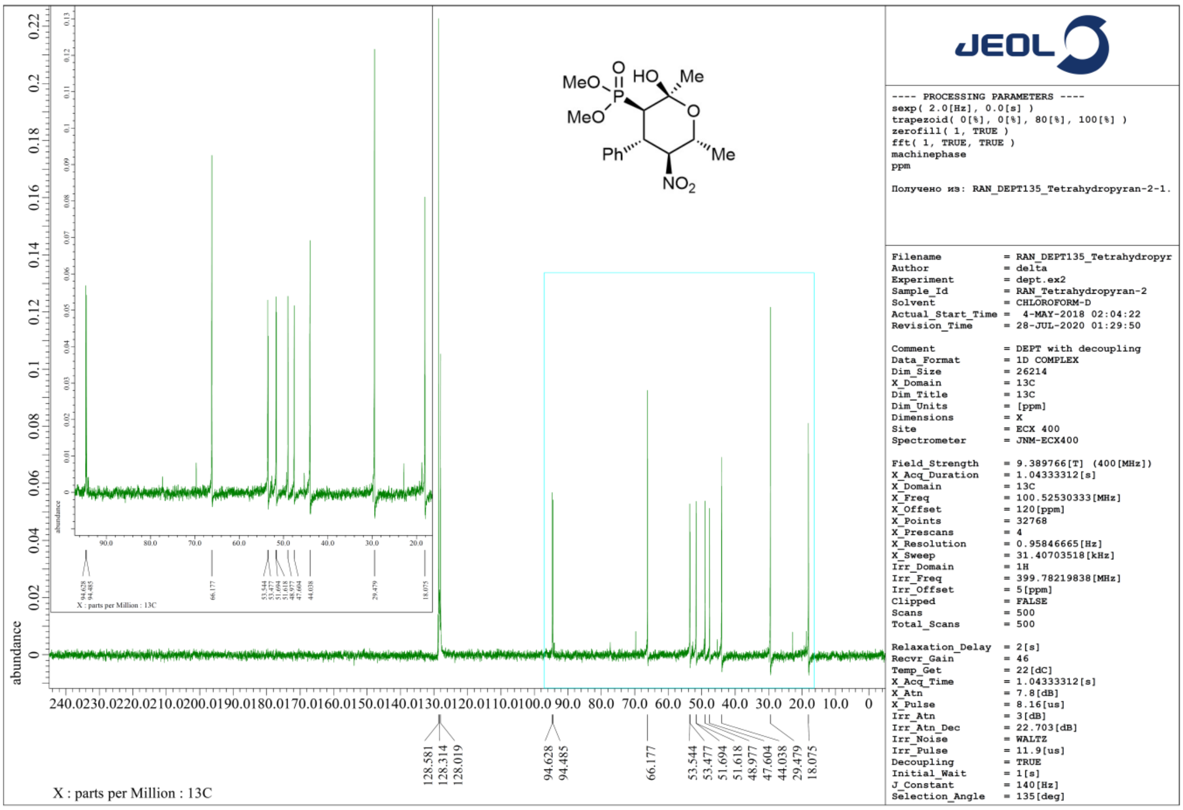

^{31}P NMR spectra of dimethyl [(2*S*,3*R*,4*S*,5*S*,6*R*)-2-hydroxy-2,6-dimethyl-5-nitro-4-phenyltetrahydro-2*H*-pyran-3-yl]phosphonate (**13b**) in $CDCl_3$

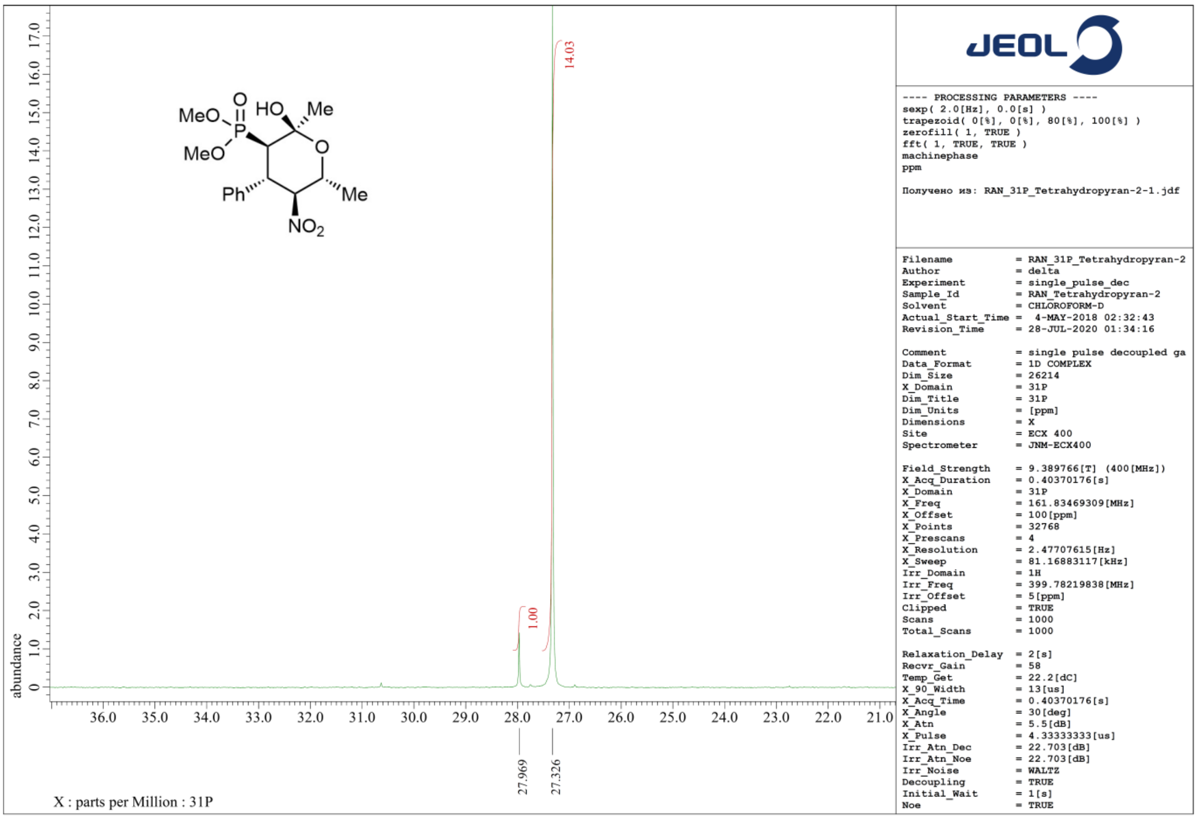

HRMS of dimethyl [(2*S*,3*R*,4*S*,5*S*,6*R*)-2-hydroxy-2,6-dimethyl-5-nitro-4-phenyltetrahydro-2*H*-pyran-3-yl]phosphonate (**13b**)

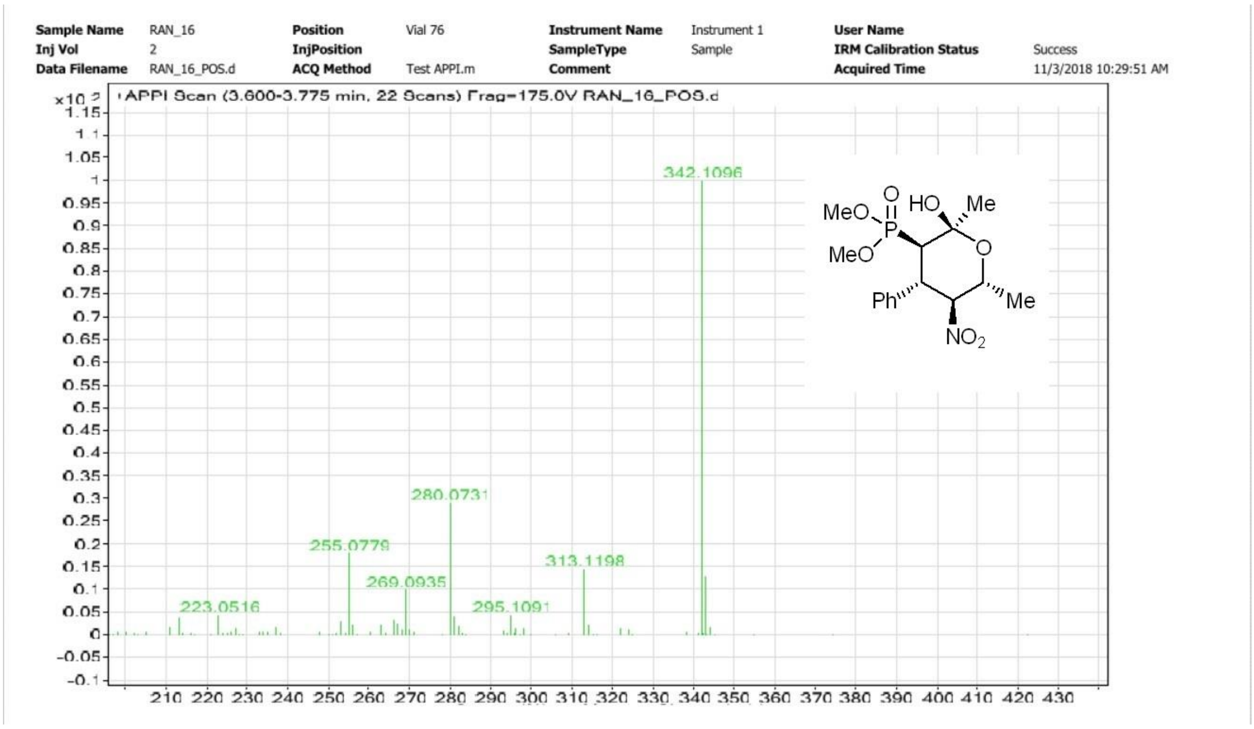

FTIR spectra of dimethyl [(2*S*,3*R*,4*S*,5*S*,6*R*)-2-hydroxy-2-methyl-5-nitro-4-phenyl-6-propyl-tetrahydro-2*H*-pyran-3-yl)phosphonate (**13c**)

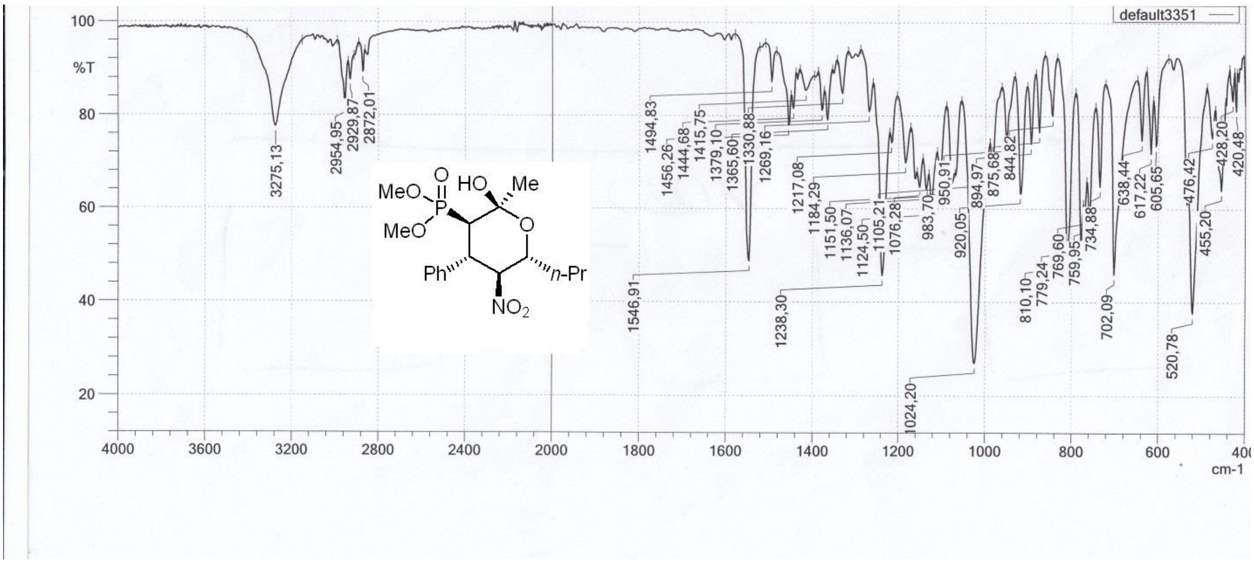

^{1}H NMR spectra of dimethyl [(2*S*,3*R*,4*S*,5*S*,6*R*)-2-hydroxy-2-methyl-5-nitro-4-phenyl-6-propyl-tetrahydro-2*H*-pyran-3-yl)phosphonate (**13c**) in $CDCl_3$

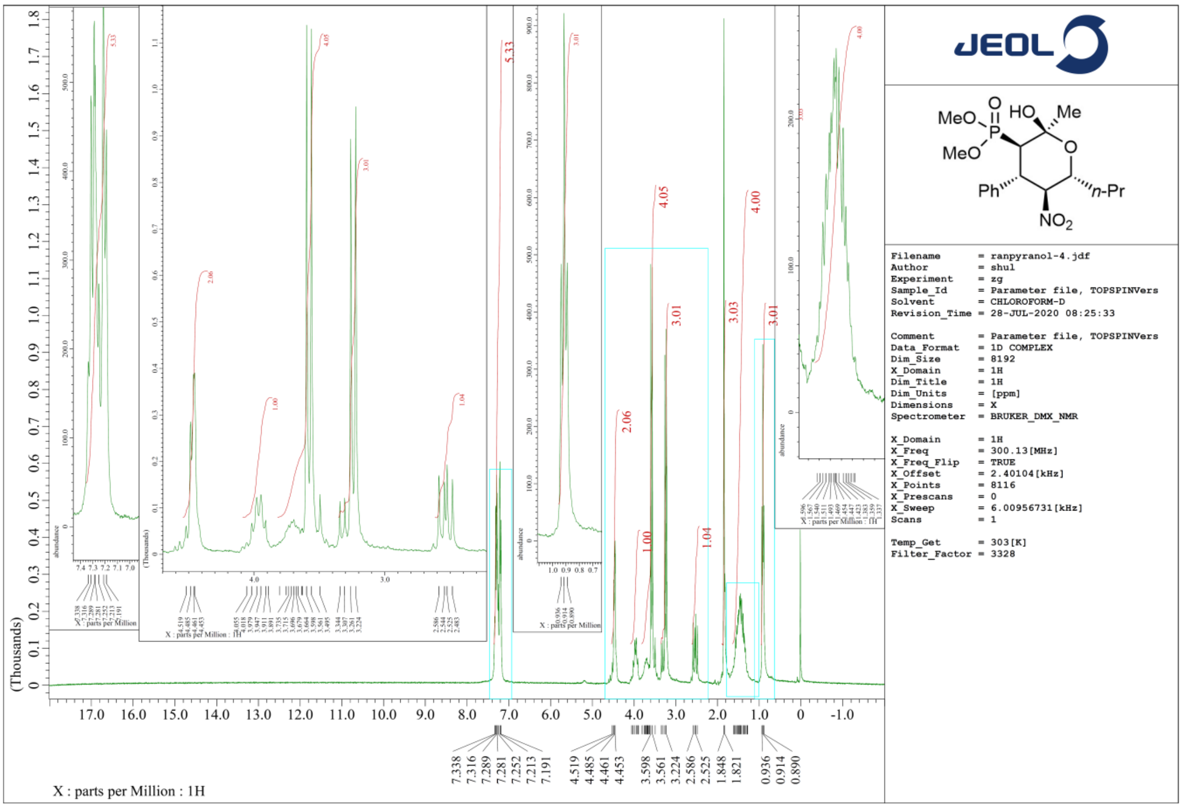

^{13}C NMR spectra of dimethyl [(2*S*,3*R*,4*S*,5*S*,6*R*)-2-hydroxy-2-methyl-5-nitro-4-phenyl-6-propyl-tetrahydro-2*H*-pyran-3-yl)phosphonate (**13c**) in $CDCl_3$

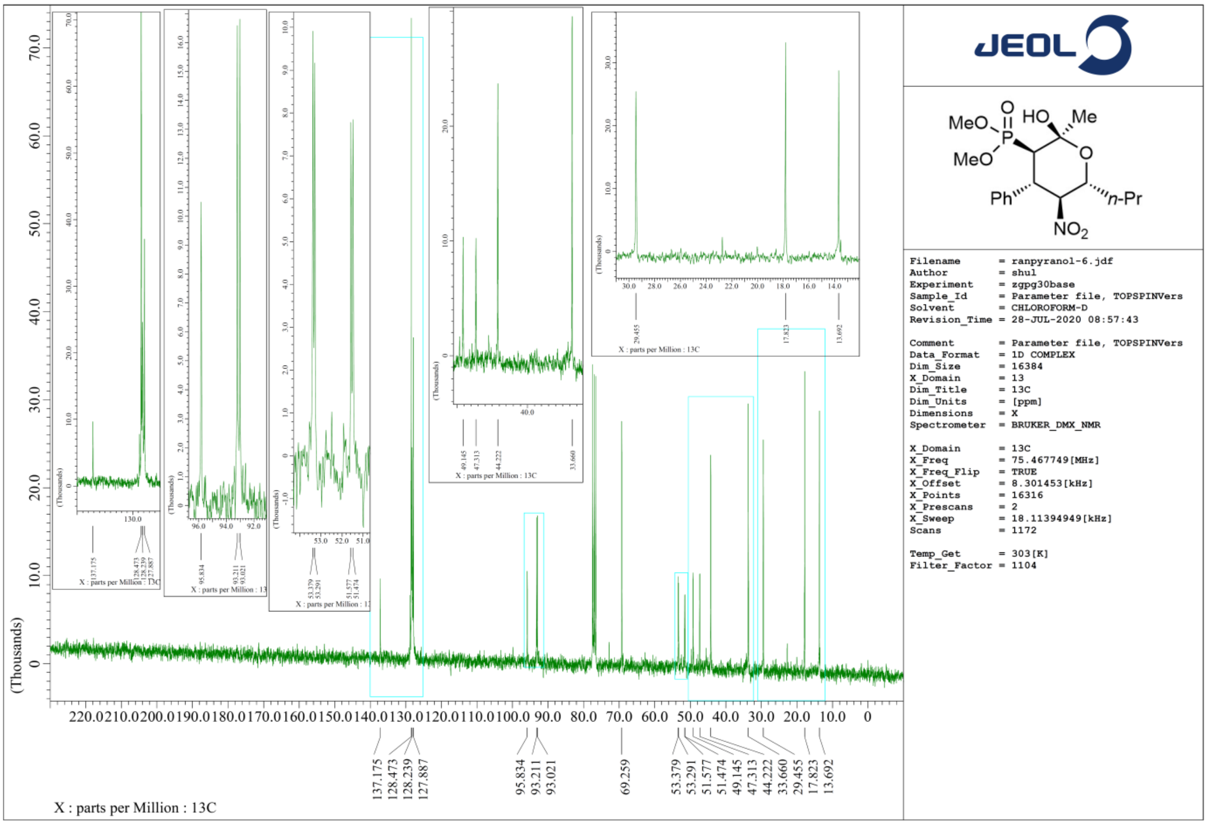

DEPT NMR spectra of dimethyl [(2*S*,3*R*,4*S*,5*S*,6*R*)-2-hydroxy-2-methyl-5-nitro-4-phenyl-6-propyl-tetrahydro-2*H*-pyran-3-yl)phosphonate (**13c**) in $CDCl_3$

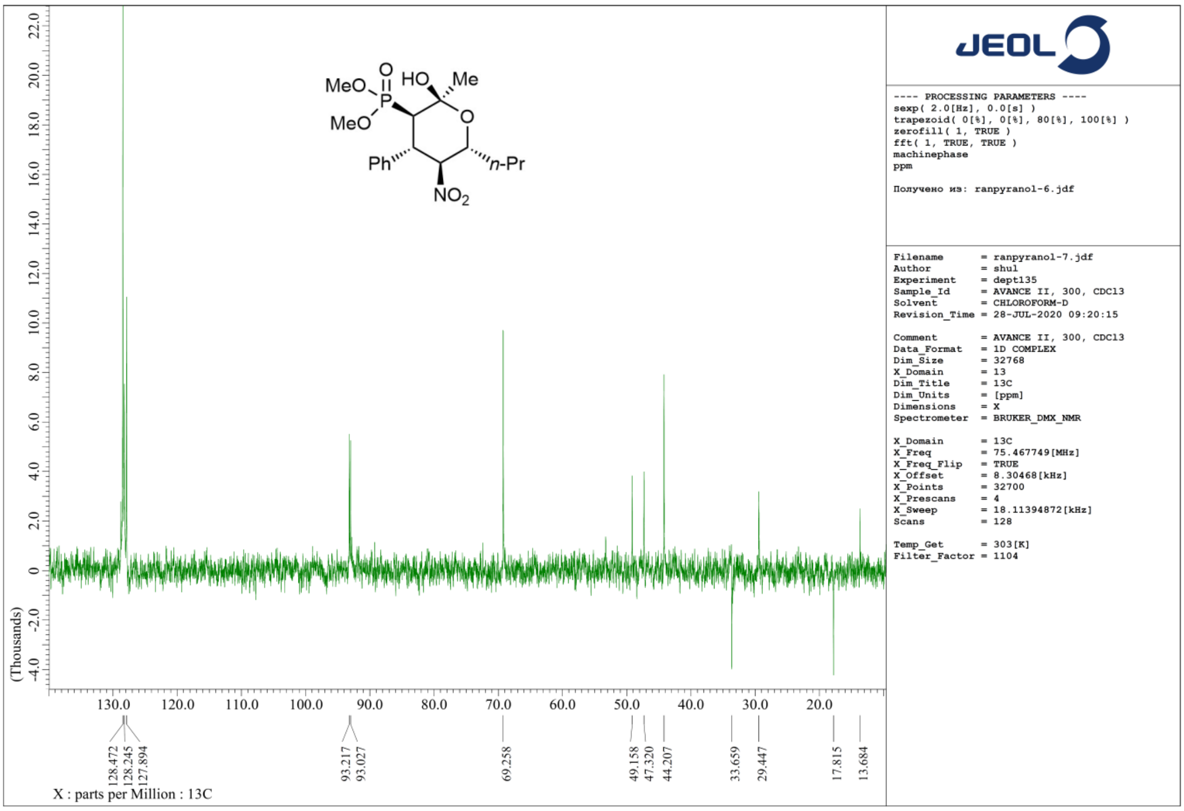

^{31}P NMR spectra of dimethyl [(2*S*,3*R*,4*S*,5*S*,6*R*)-2-hydroxy-2-methyl-5-nitro-4-phenyl-6-propyl-tetrahydro-2*H*-pyran-3-yl)phosphonate (**13c**) in $CDCl_3$

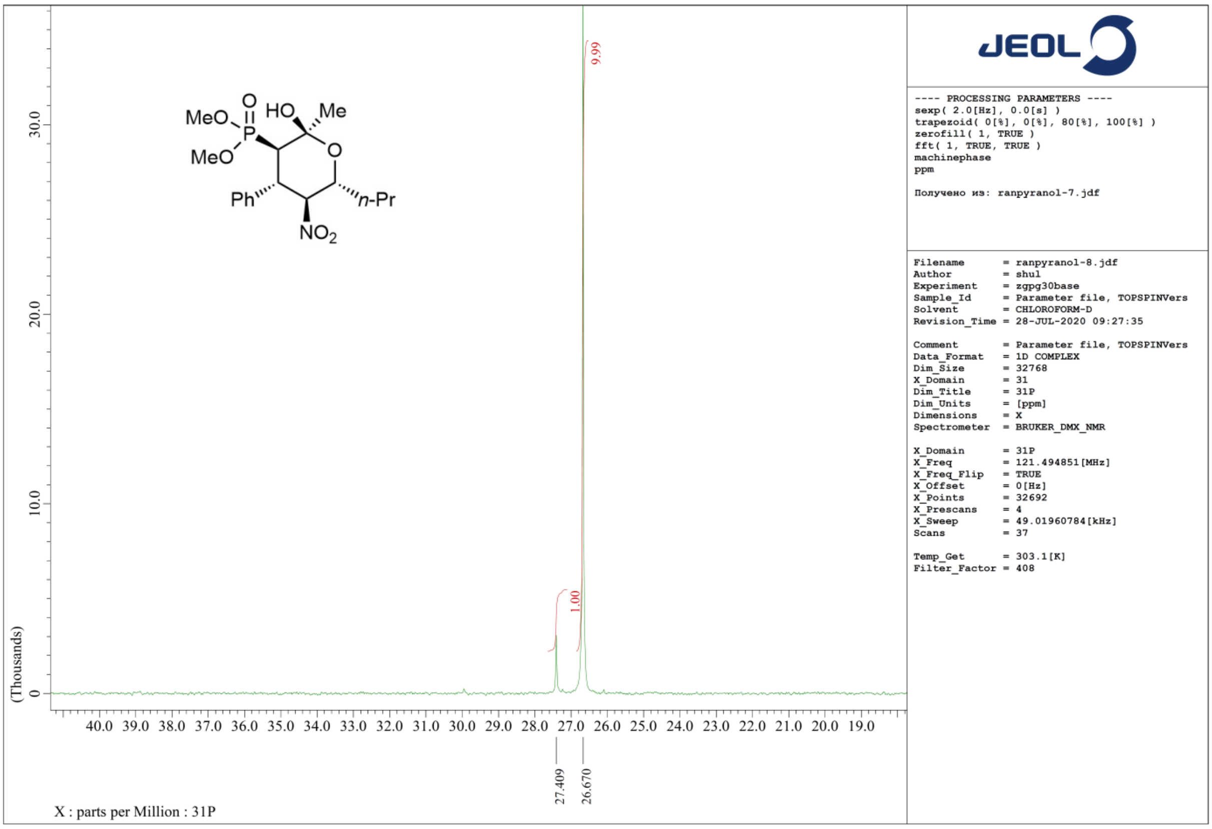

HRMS of dimethyl [(2*S*,3*R*,4*S*,5*S*,6*R*)-2-hydroxy-2-methyl-5-nitro-4-phenyl-6-propyl-tetrahydro-2*H*-pyran-3-yl)phosphonate (**13c**)

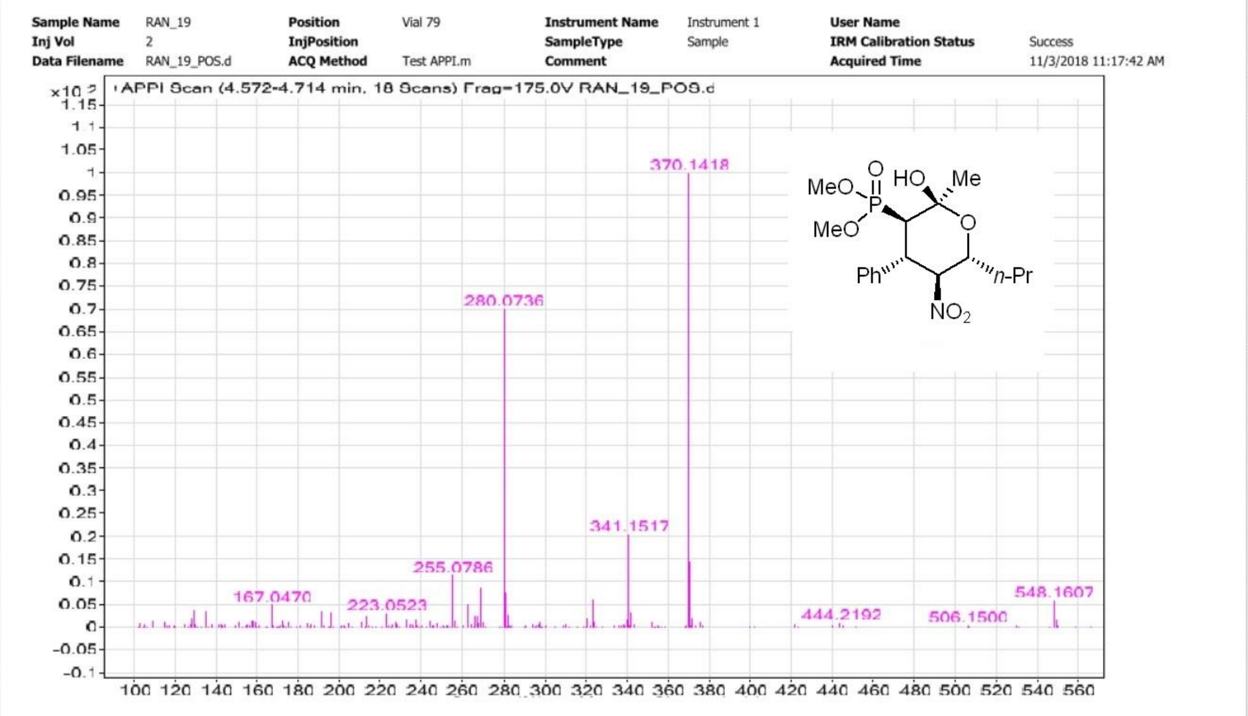

FTIR spectra of dimethyl [(2*S*,3*R*,4*S*,5*S*,6*R*)-2-hydroxy-6-isobutyl-2-methyl-5-nitro-4-phenyl-tetrahydro 2*H*-pyran-3-yl)phosphonate (**13d**)

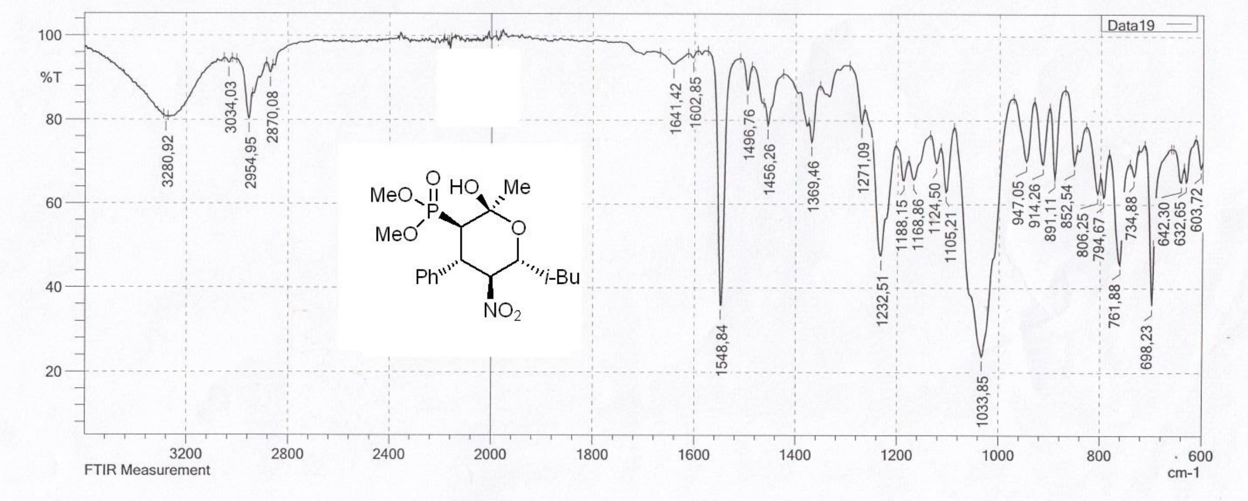

^{1}H NMR spectra of dimethyl [(2*S*,3*R*,4*S*,5*S*,6*R*)-2-hydroxy-6-isobutyl-2-methyl-5-nitro-4-phenyl-tetrahydro 2*H*-pyran-3-yl)phosphonate (**13d**) in $CDCl_3$

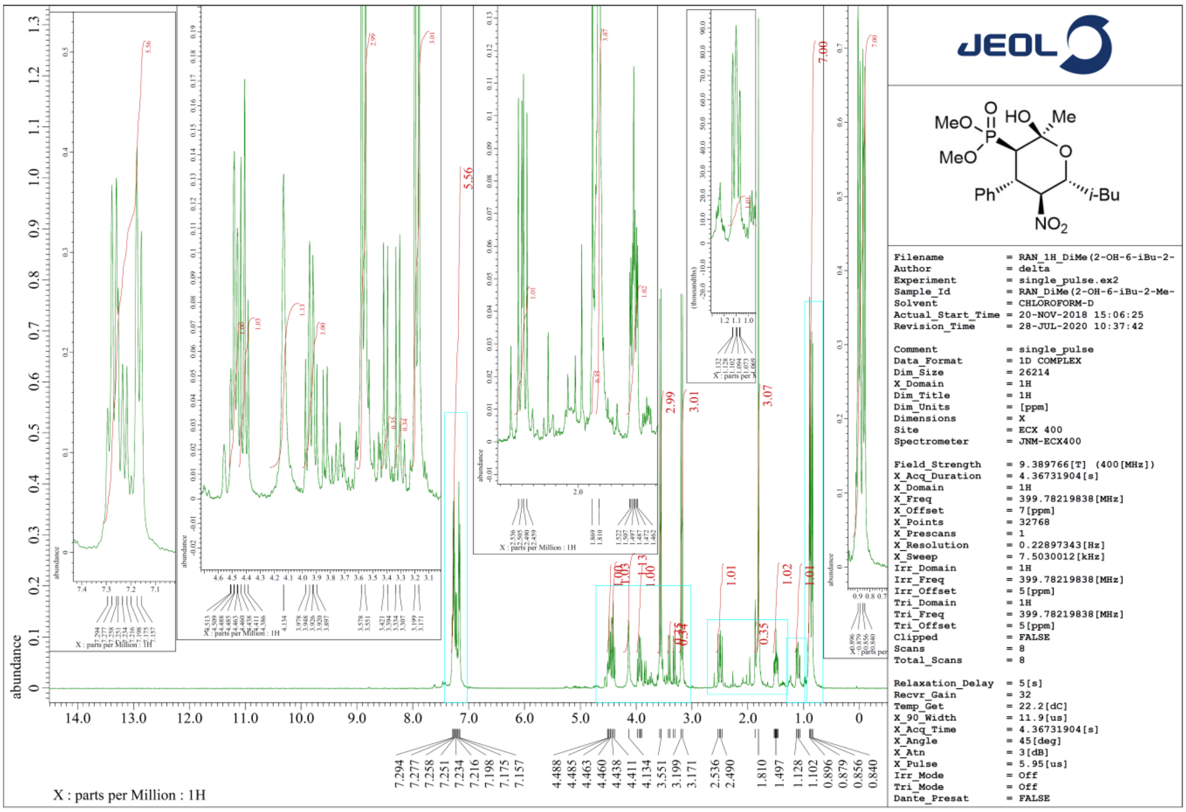

^{13}C NMR spectra of dimethyl [(2*S*,3*R*,4*S*,5*S*,6*R*)-2-hydroxy-6-isobutyl-2-methyl-5-nitro-4-phenyl-tetrahydro 2*H*-pyran-3-yl)phosphonate (**13d**) in $CDCl_3$

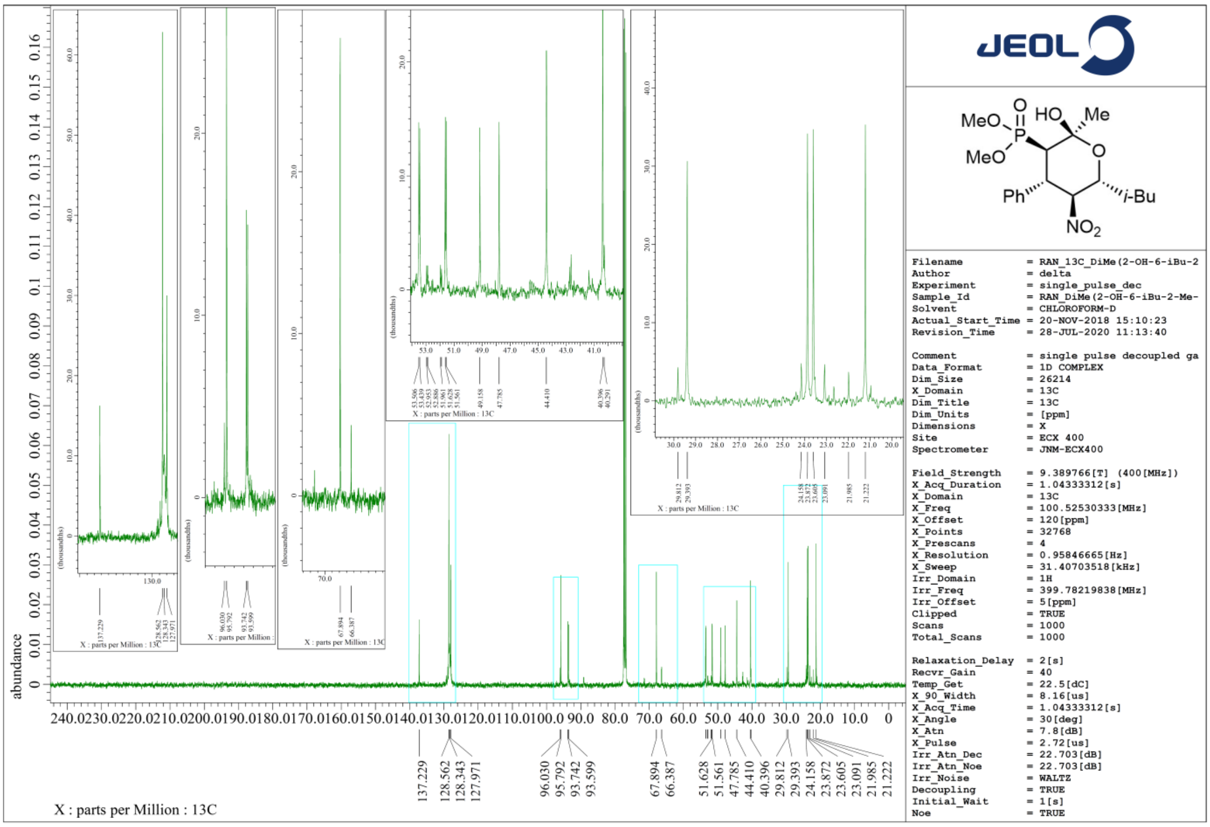

DEPT NMR spectra of dimethyl [(2*S*,3*R*,4*S*,5*S*,6*R*)-2-hydroxy-6-isobutyl-2-methyl-5-nitro-4-phenyl-tetrahydro 2*H*-pyran-3-yl)phosphonate (**13d**) in $CDCl_3$

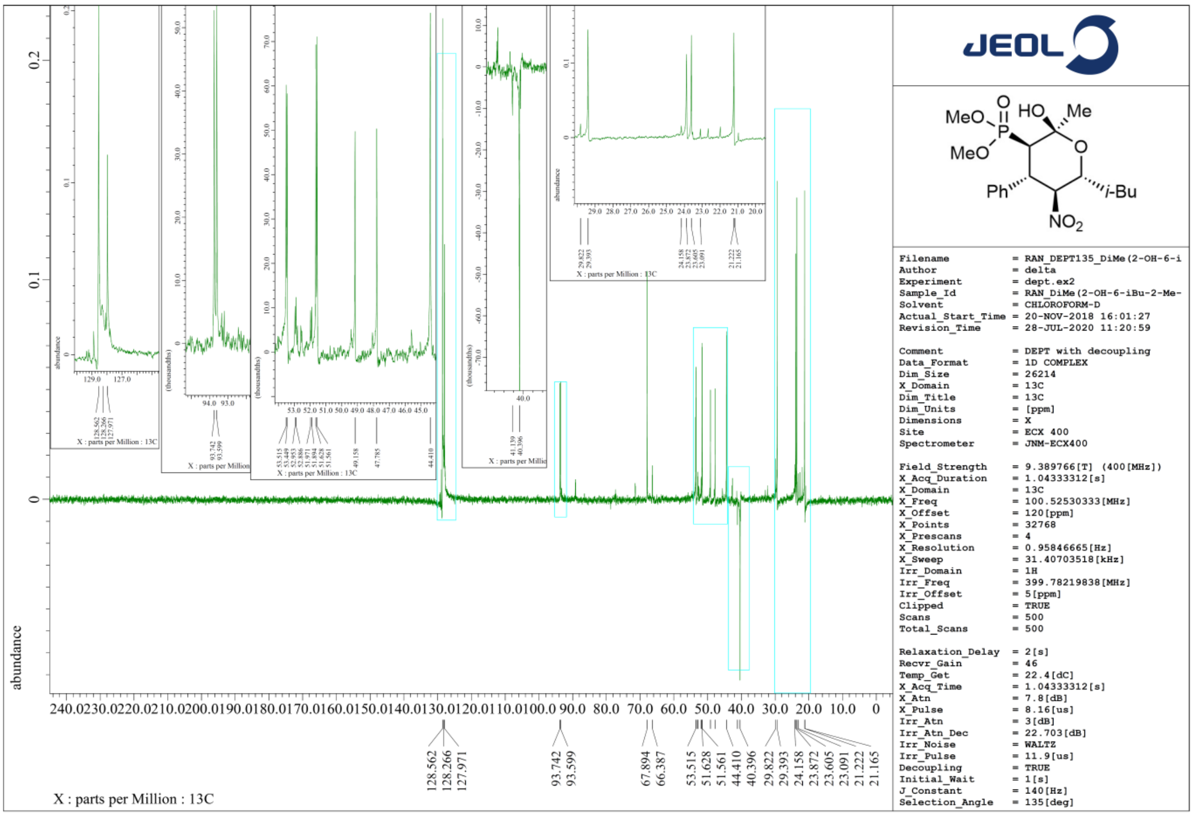

^{31}P NMR spectra of dimethyl [(2*S*,3*R*,4*S*,5*S*,6*R*)-2-hydroxy-6-isobutyl-2-methyl-5-nitro-4-phenyl-tetrahydro 2*H*-pyran-3-yl)phosphonate (**13d**) in $CDCl_3$

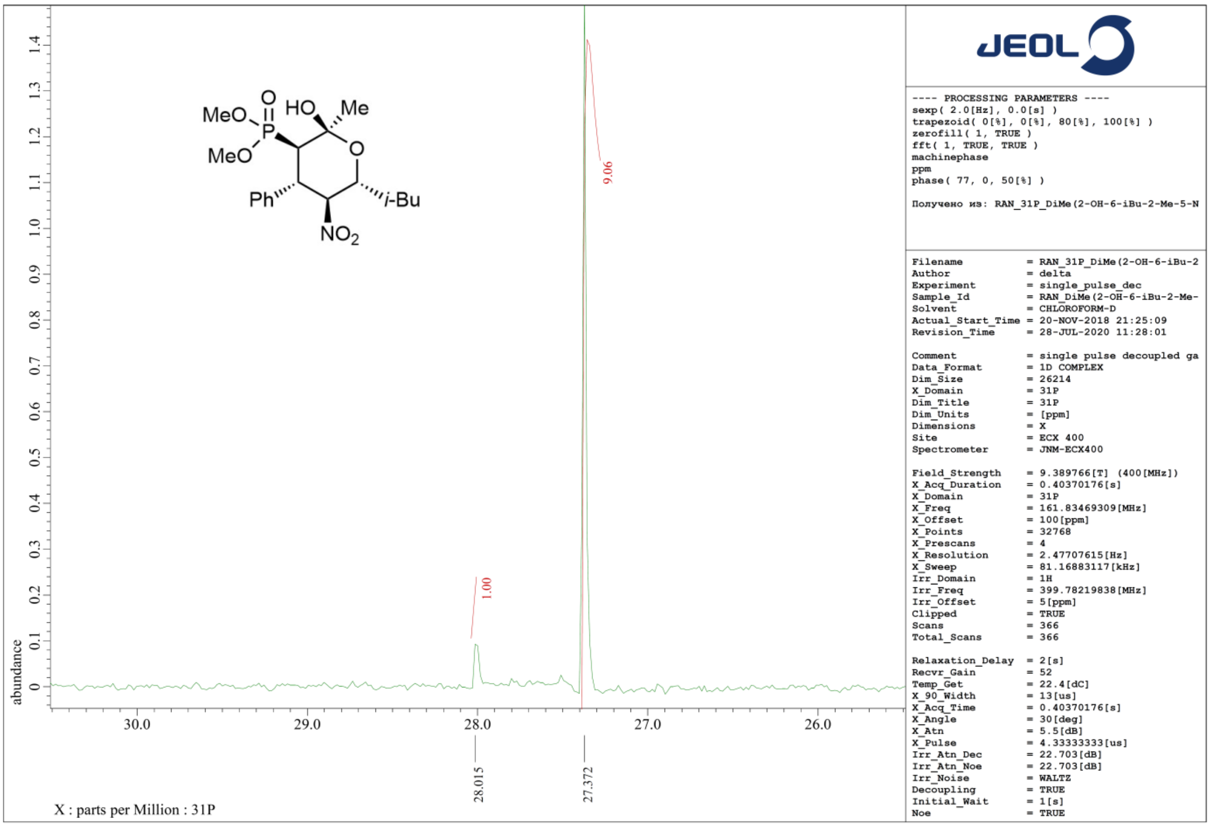

HRMS of dimethyl [(2*S*,3*R*,4*S*,5*S*,6*R*)-2-hydroxy-6-isobutyl-2-methyl-5-nitro-4-phenyl-tetrahydro 2*H*-pyran-3-yl)phosphonate (**13d**)

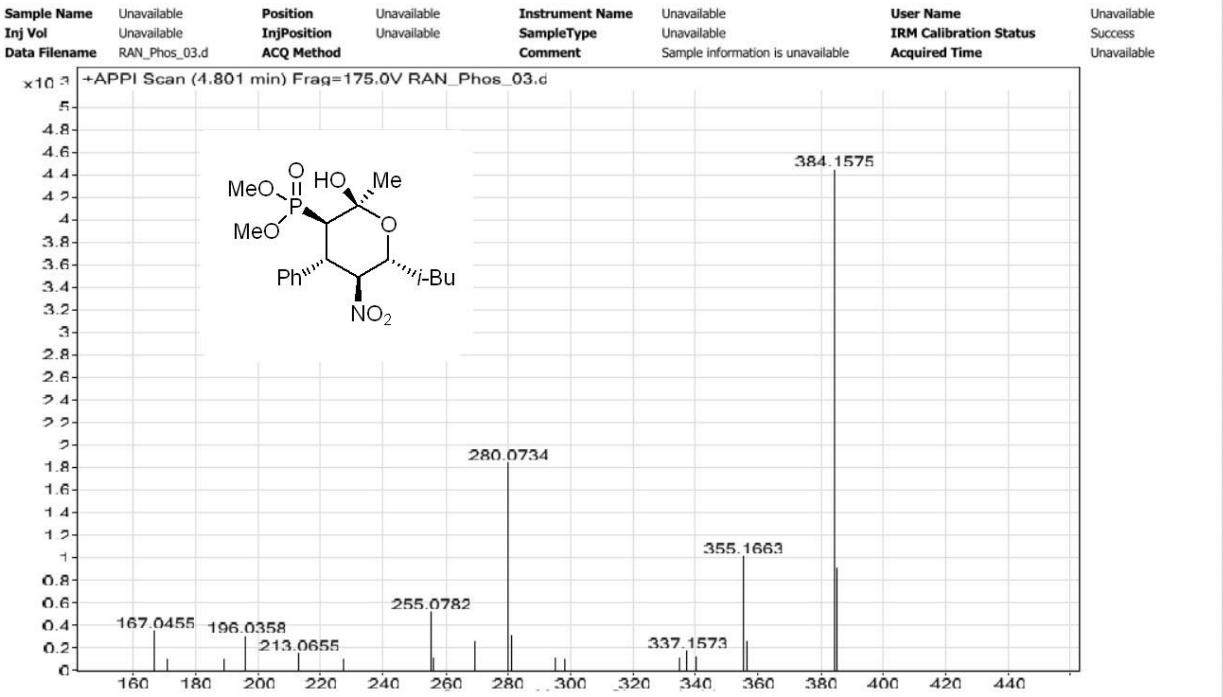

FTIR spectra of dimethyl [(2*S*,3*R*,4*S*,5*S*,6*R*)-2-hydroxy-2-methyl-5-nitro-4,6-diphenyltetrahydro-2*H*-pyran-3-yl]phosphonate (**13e**)

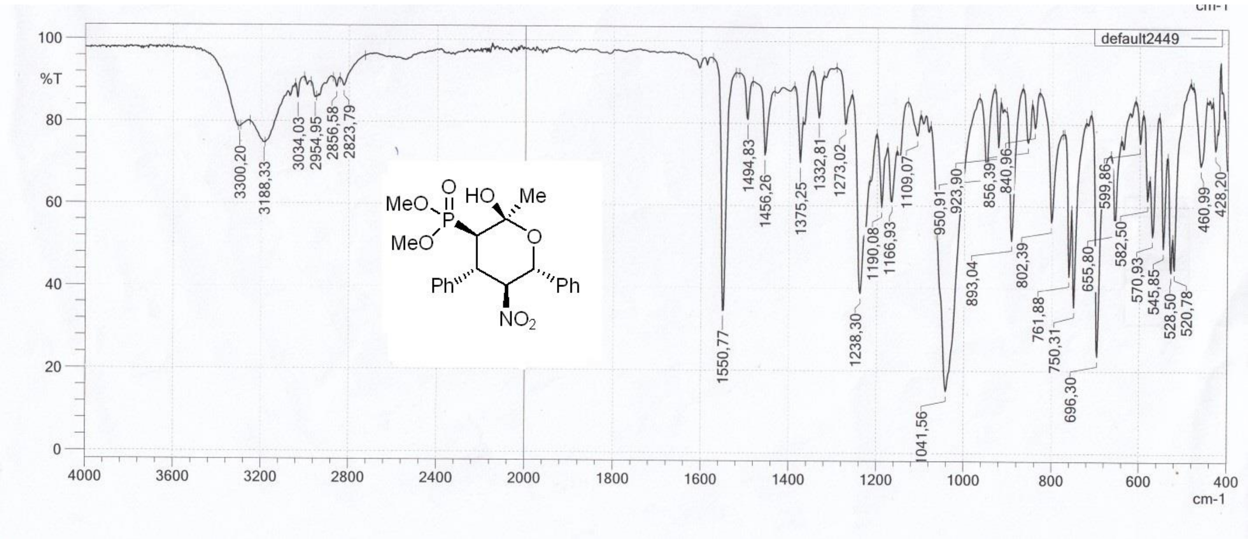

^{1}H NMR spectra of dimethyl [(2*S*,3*R*,4*S*,5*S*,6*R*)-2-hydroxy-2-methyl-5-nitro-4,6-diphenyltetrahydro-2*H*-pyran-3-yl]phosphonate (**13e**) in $CDCl_3$

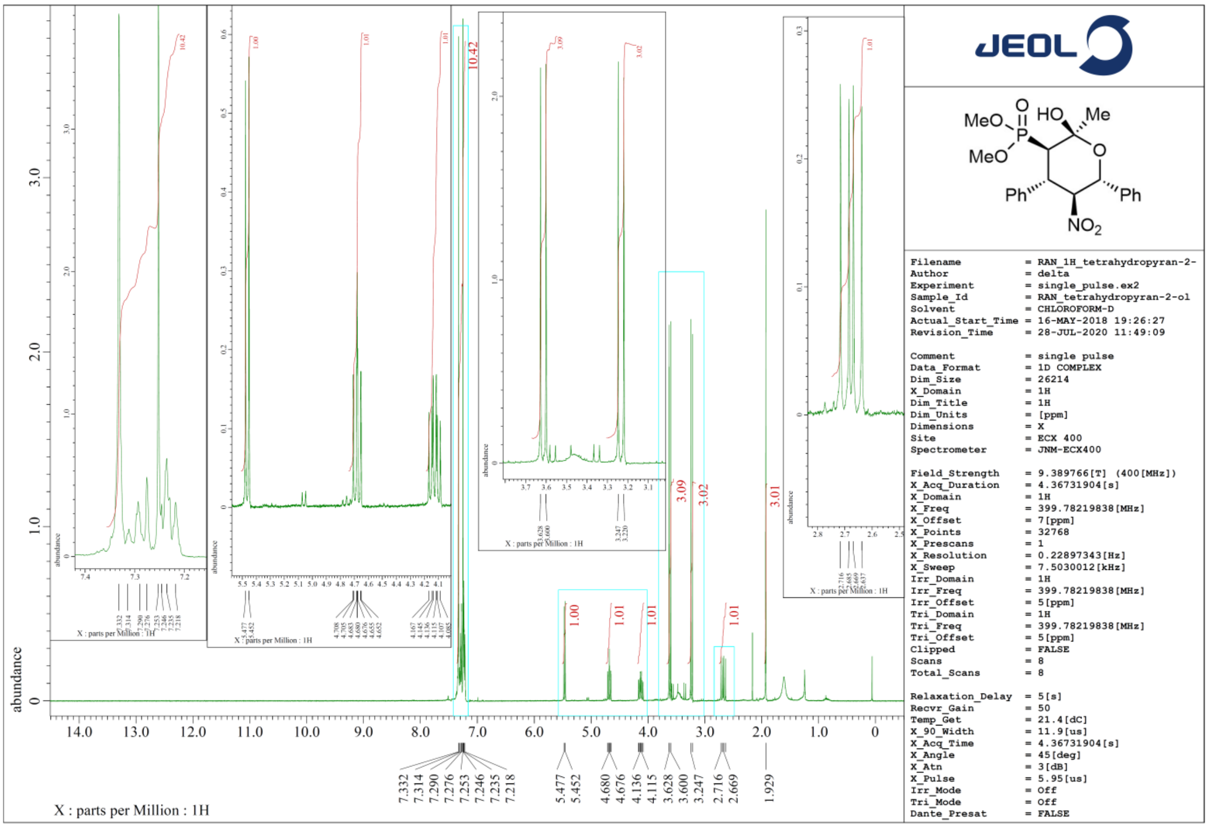

^{13}C NMR spectra of dimethyl [(2*S*,3*R*,4*S*,5*S*,6*R*)-2-hydroxy-2-methyl-5-nitro-4,6-diphenyltetrahydro-2*H*-pyran-3-yl]phosphonate (**13e**) in $CDCl_3$

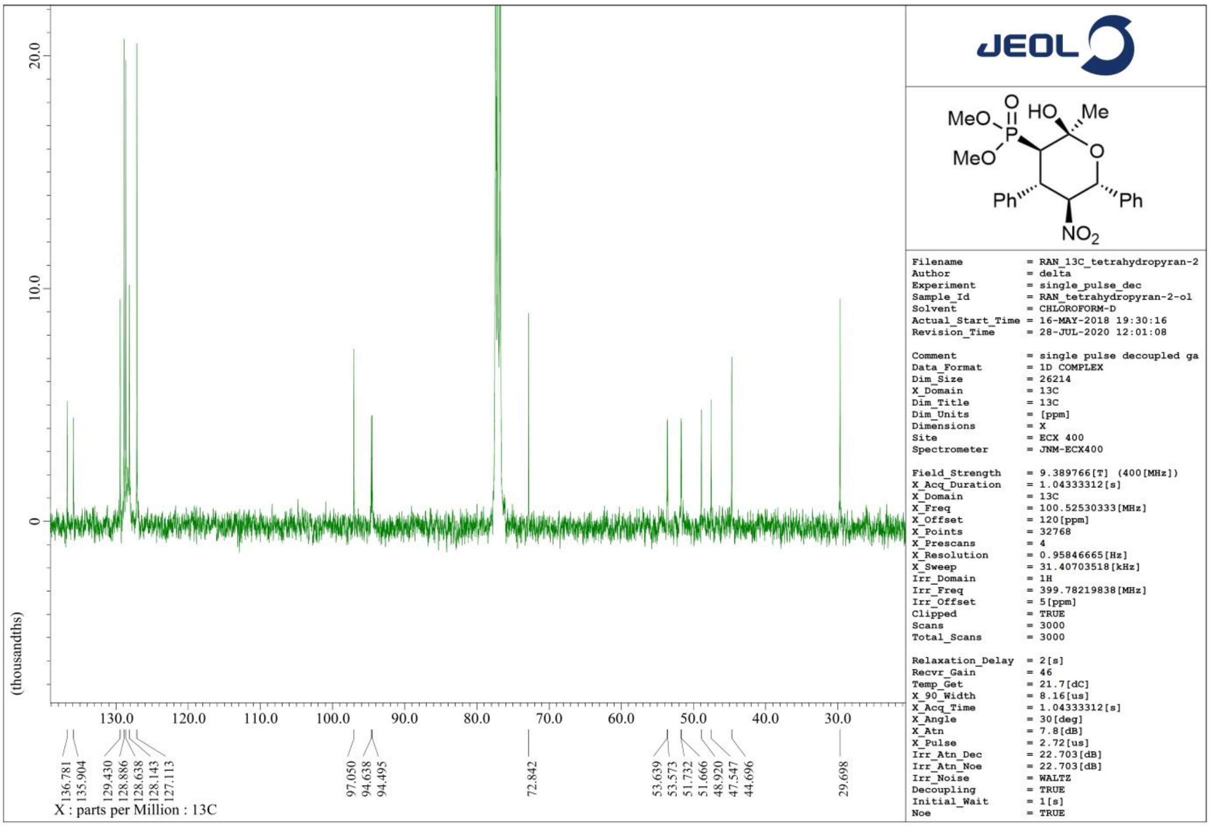

DEPT NMR spectra of dimethyl [(2*S*,3*R*,4*S*,5*S*,6*R*)-2-hydroxy-2-methyl-5-nitro-4,6-diphenyltetrahydro-2*H*-pyran-3-yl]phosphonate (**13e**) in $CDCl_3$

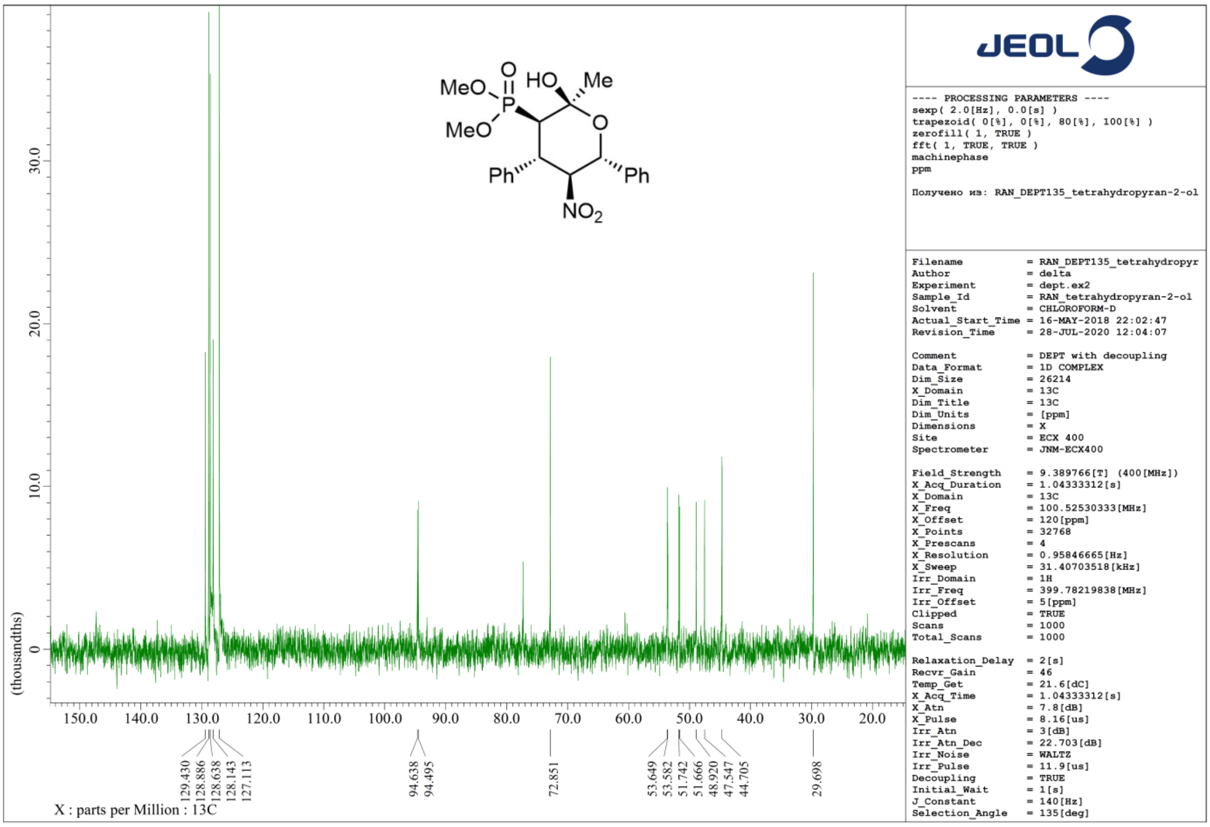

^{31}P NMR spectra of dimethyl [(2*S*,3*R*,4*S*,5*S*,6*R*)-2-hydroxy-2-methyl-5-nitro-4,6-diphenyltetrahydro-2*H*-pyran-3-yl]phosphonate (**13e**) in $CDCl_3$

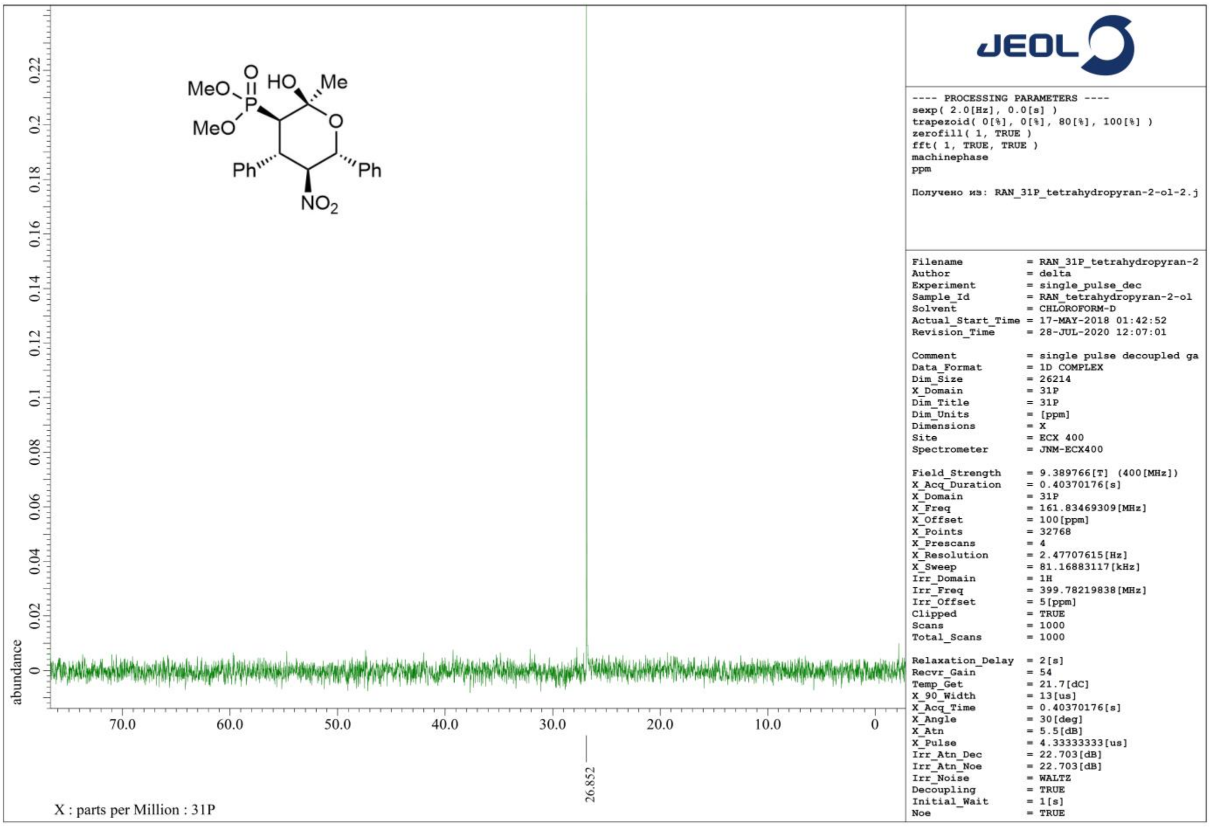

HRMS of dimethyl [(2*S*,3*R*,4*S*,5*S*,6*R*)-2-hydroxy-2-methyl-5-nitro-4,6-diphenyltetrahydro-2*H*-pyran-3-yl]phosphonate (**13e**)

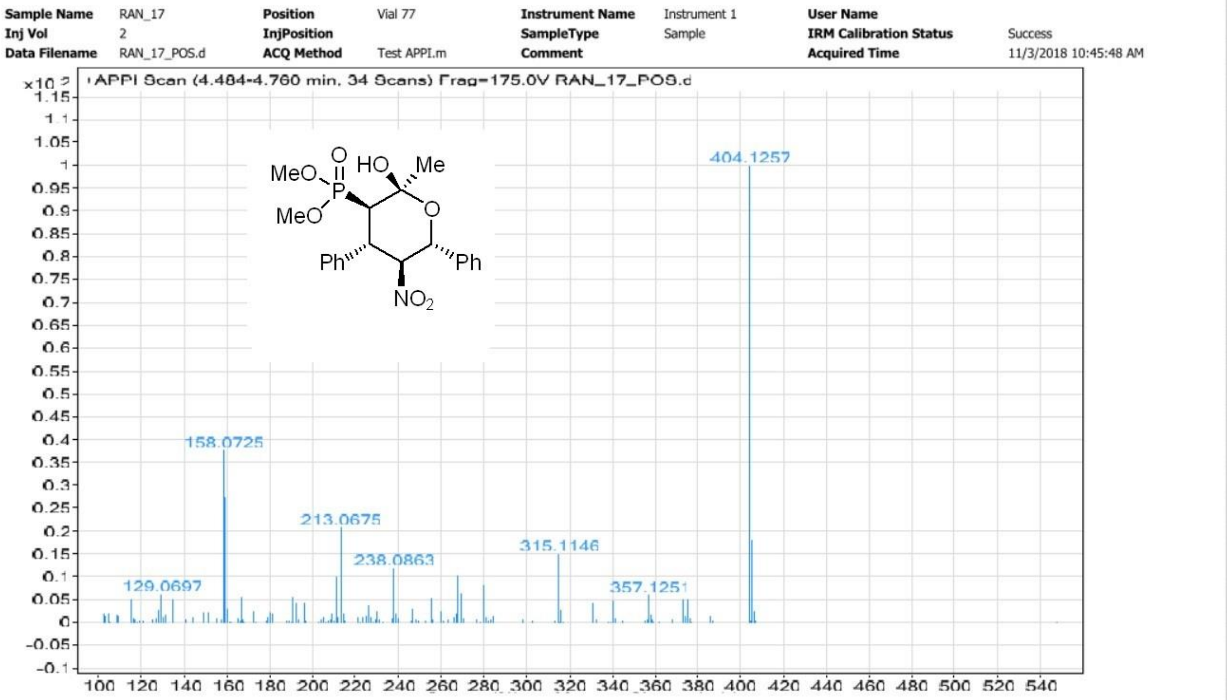

FTIR spectra of dimethyl [(2*S*,3*R*,4*S*,5*S*,6*R*)-6-(4-chlorophenyl)-2-hydroxy-2-methyl-5-nitro-4-phenyltetrahydro-2*H*-pyran-3-yl]phosphonate (**13f**)

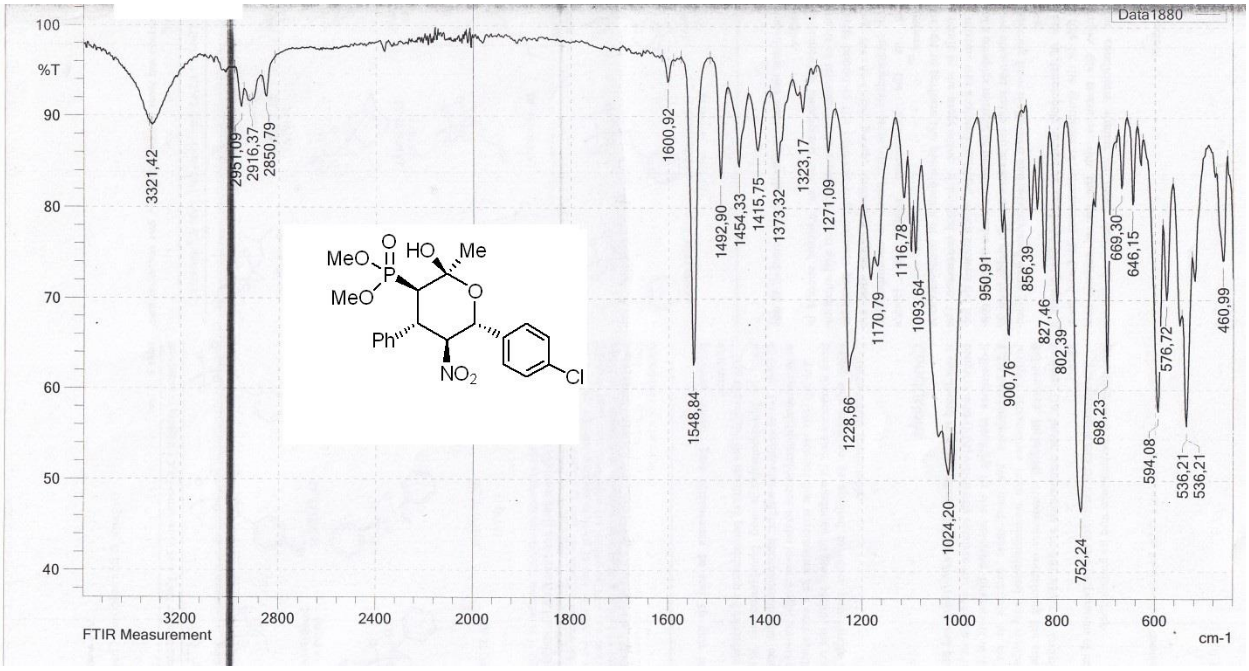

^{1}H NMR spectra of dimethyl [(2*S*,3*R*,4*S*,5*S*,6*R*)-6-(4-chlorophenyl)-2-hydroxy-2-methyl-5-nitro-4-phenyltetrahydro-2*H*-pyran-3-yl]phosphonate (**13f**) in $CDCl_3$

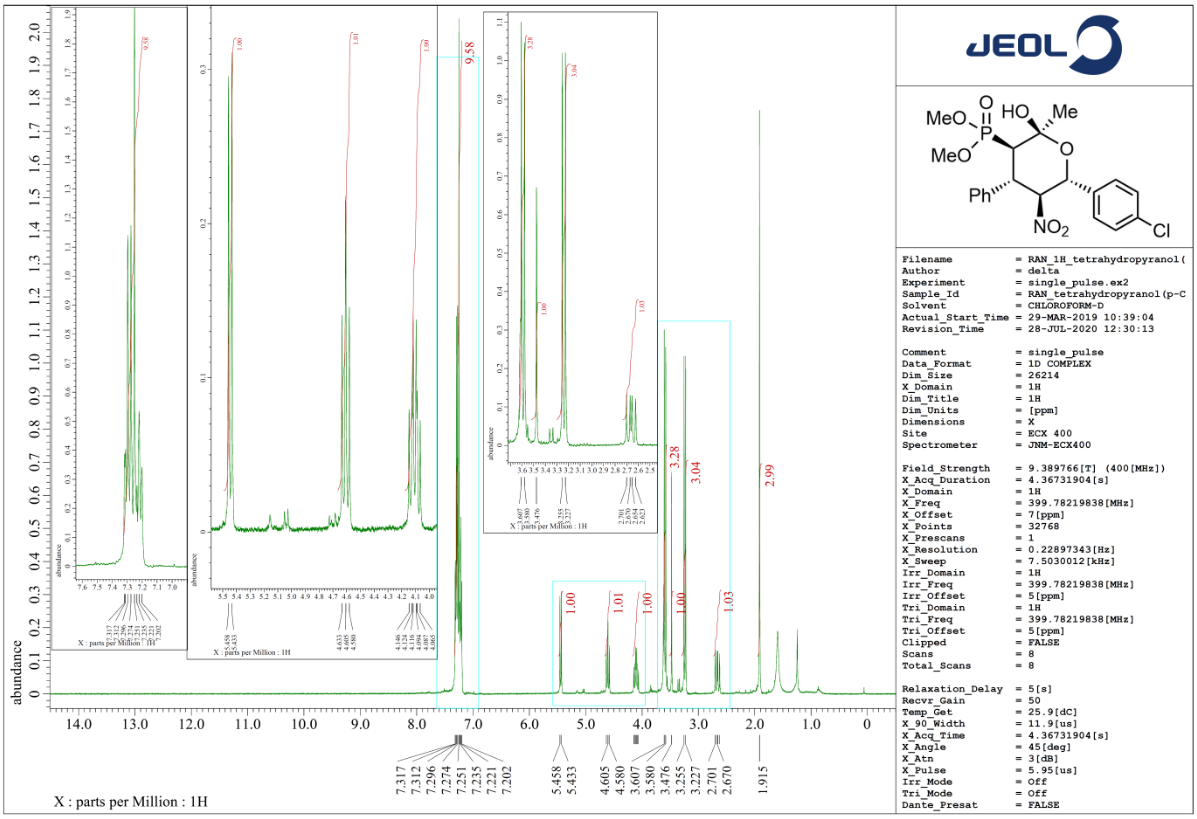

^{13}C NMR spectra of dimethyl [(2*S*,3*R*,4*S*,5*S*,6*R*)-6-(4-chlorophenyl)-2-hydroxy-2-methyl-5-nitro-4-phenyltetrahydro-2*H*-pyran-3-yl]phosphonate (**13f**) in $CDCl_3$

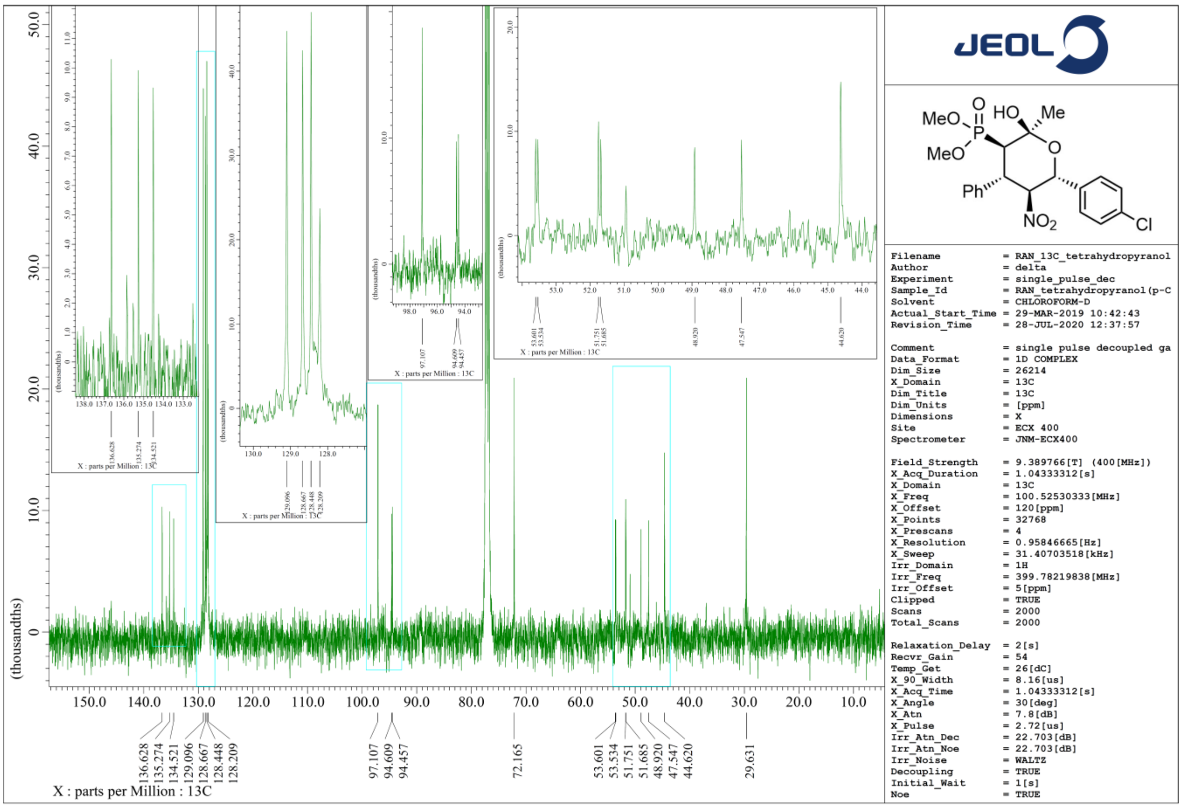

DEPT NMR spectra of dimethyl [(2*S*,3*R*,4*S*,5*S*,6*R*)-6-(4-chlorophenyl)-2-hydroxy-2-methyl-5-nitro-4-phenyltetrahydro-2*H*-pyran-3-yl]phosphonate (**13f**) in $CDCl_3$

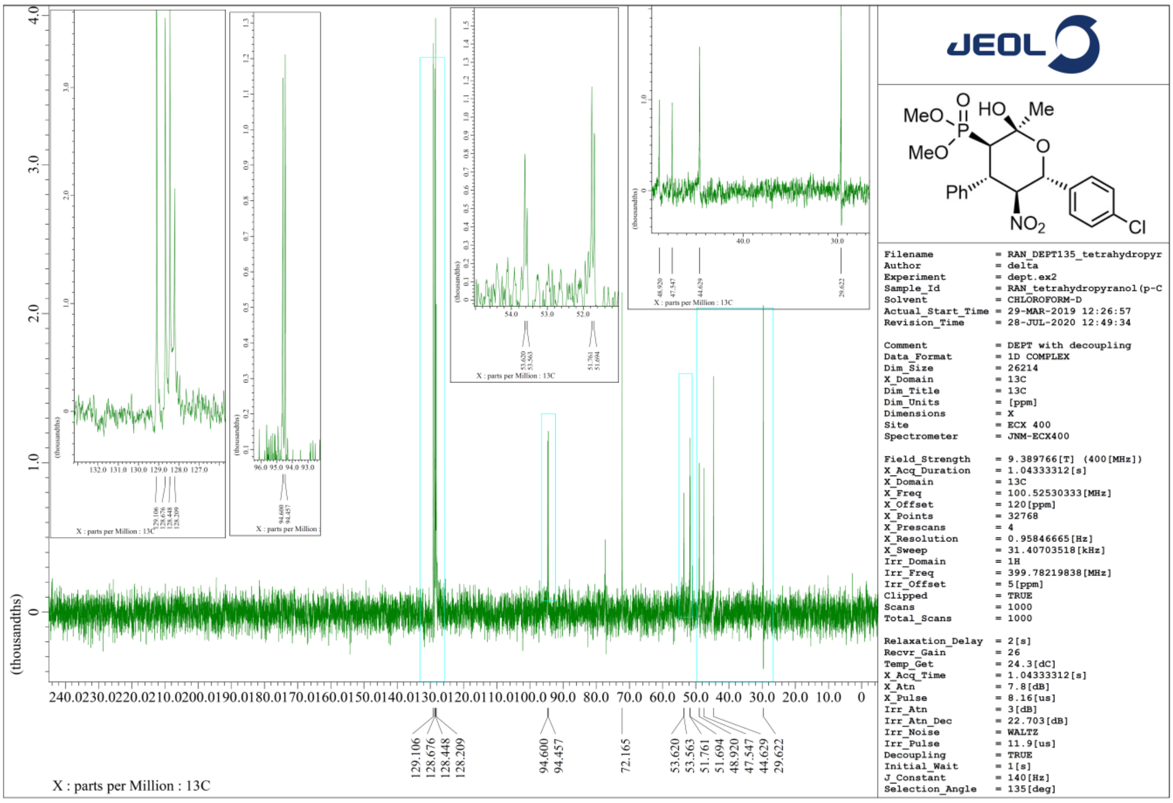

[31] P NMR spectra of dimethyl [(2*S*,3*R*,4*S*,5*S*,6*R*)-6-(4-chlorophenyl)-2-hydroxy-2-methyl-5-nitro-4-phenyltetrahydro-2*H*-pyran-3-yl]phosphonate (**13f**) in $CDCl_3$

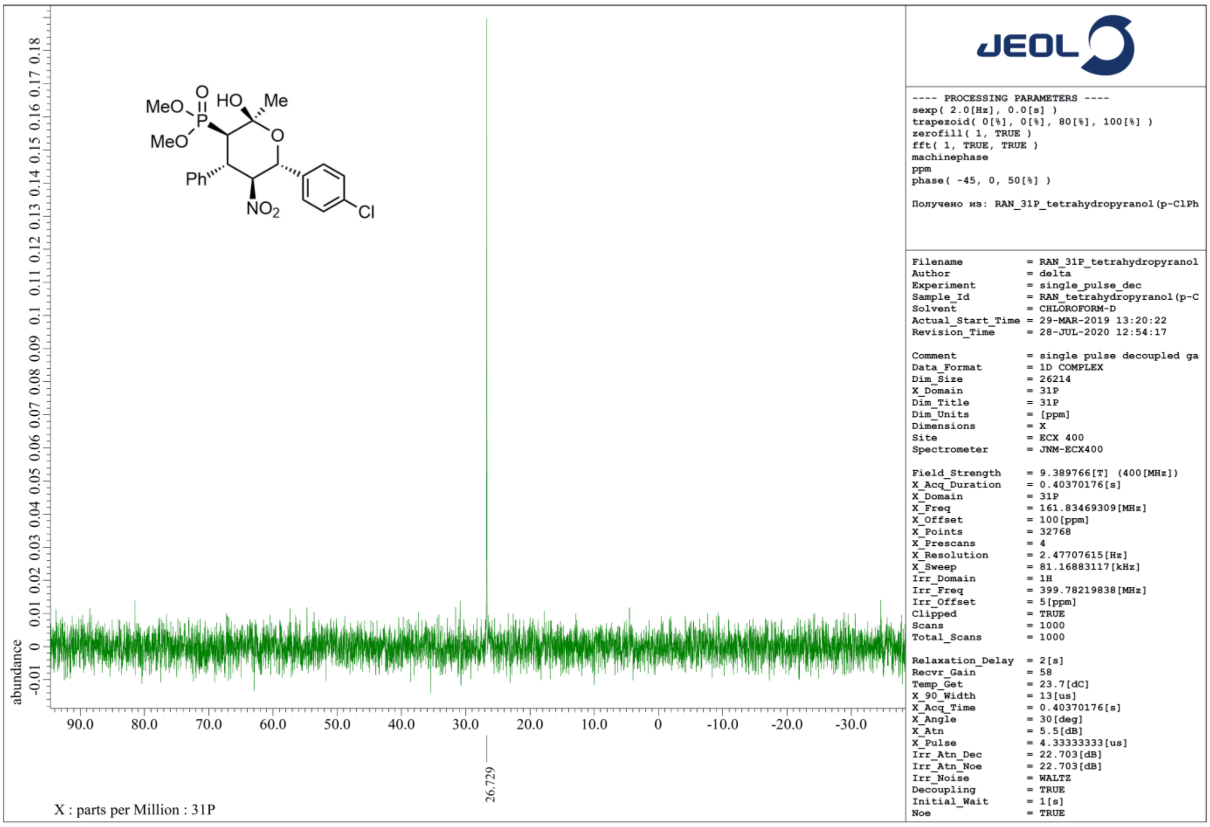

HRMS of dimethyl [(2*S*,3*R*,4*S*,5*S*,6*R*)-6-(4-chlorophenyl)-2-hydroxy-2-methyl-5-nitro-4-phenyltetrahydro-2*H*-pyran-3-yl]phosphonate (**13f**)

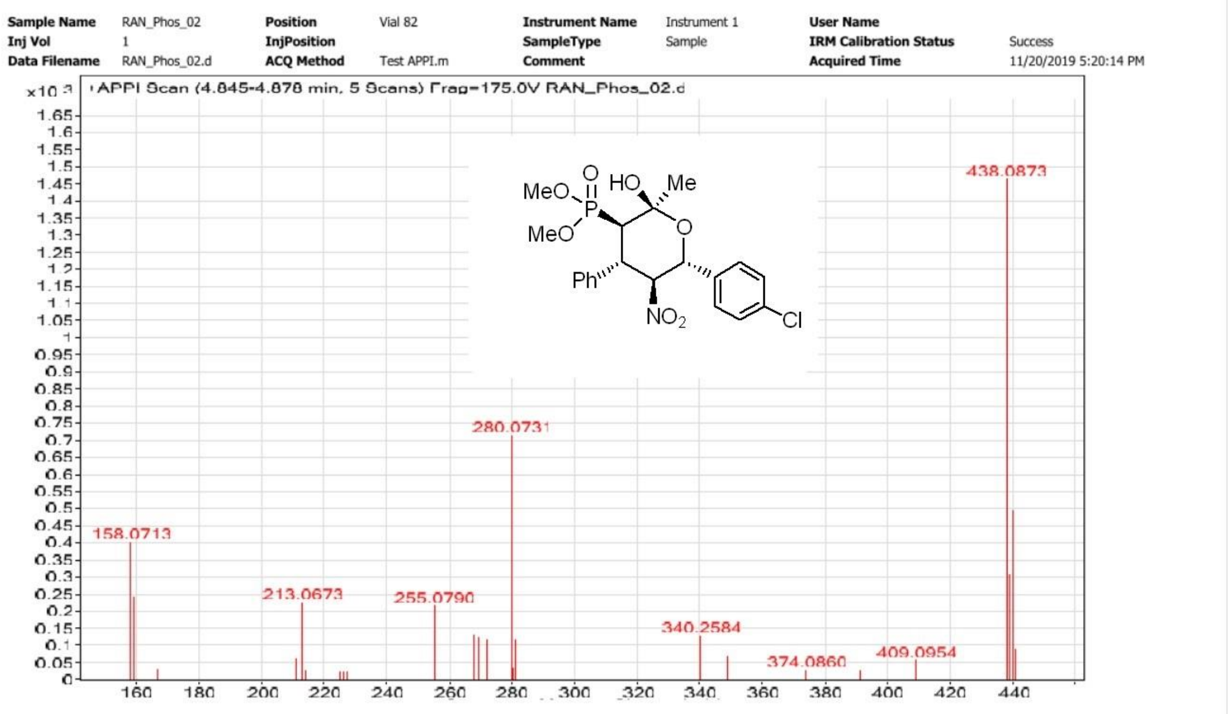

FTIR spectra of dimethyl [(2*R*,3*S*,4*S*)-2,6-dimethyl-3-nitro-4-phenyl-3,4-dihydro-2*H*-pyran-5-yl]phosphonate (**14**)

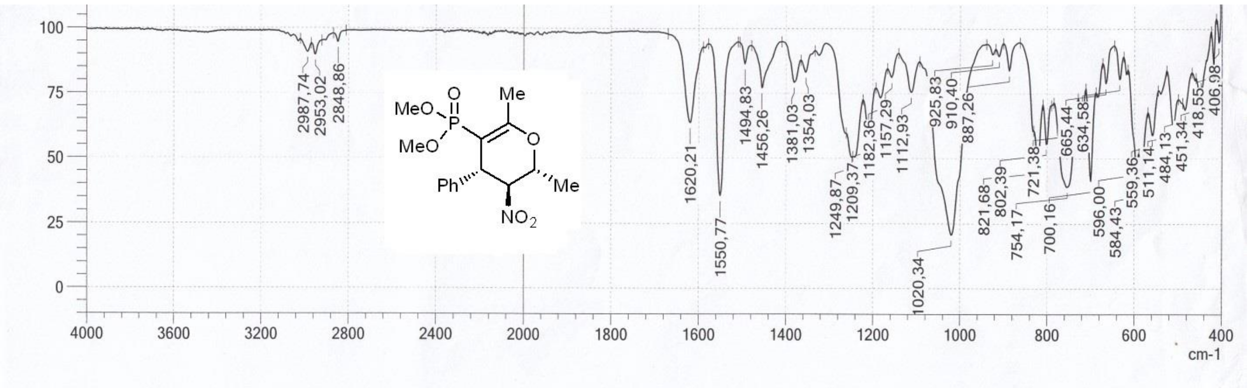

^{1}H NMR spectra of dimethyl [(2*R*,3*S*,4*S*)-2,6-dimethyl-3-nitro-4-phenyl-3,4-dihydro-2*H*-pyran-5-yl]phosphonate (**14**) in $CDCl_3$

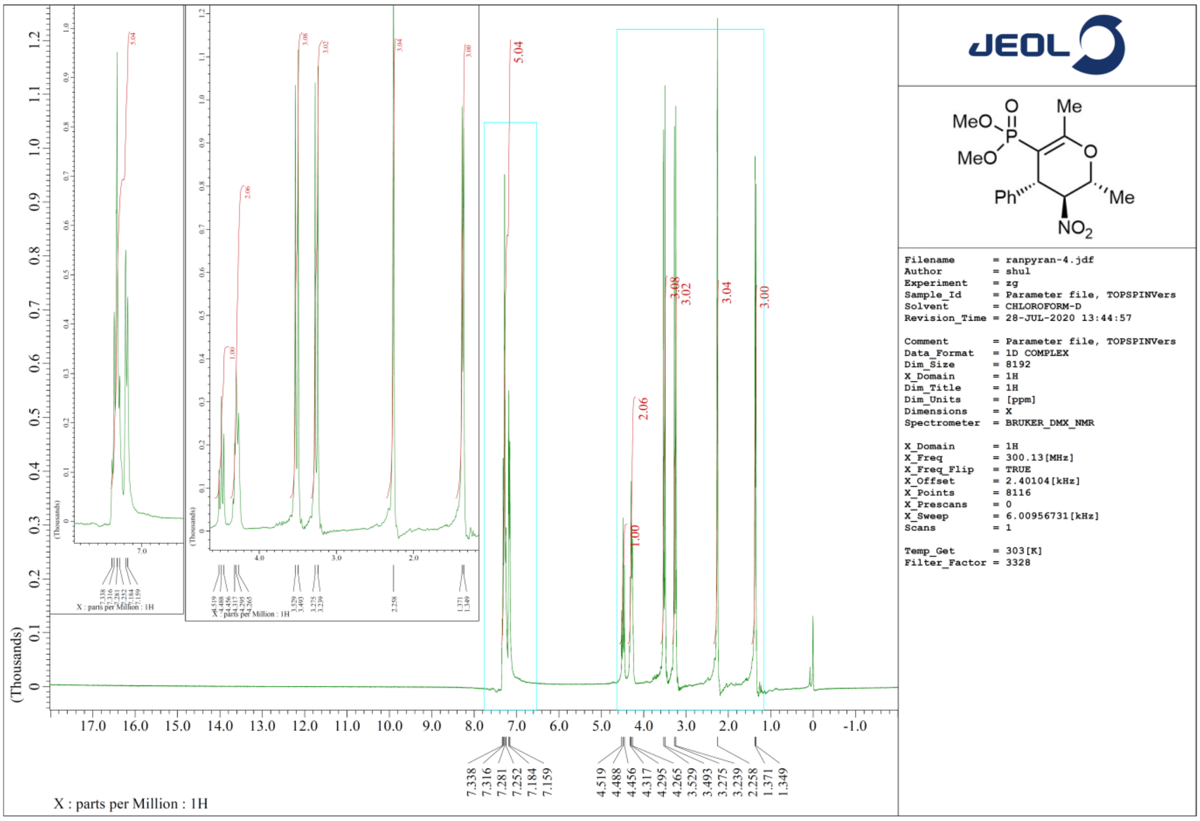

^{13}C NMR spectra of dimethyl [(2*R*,3*S*,4*S*)-2,6-dimethyl-3-nitro-4-phenyl-3,4-dihydro-2*H*-pyran-5-yl]phosphonate (**14**) in $CDCl_3$

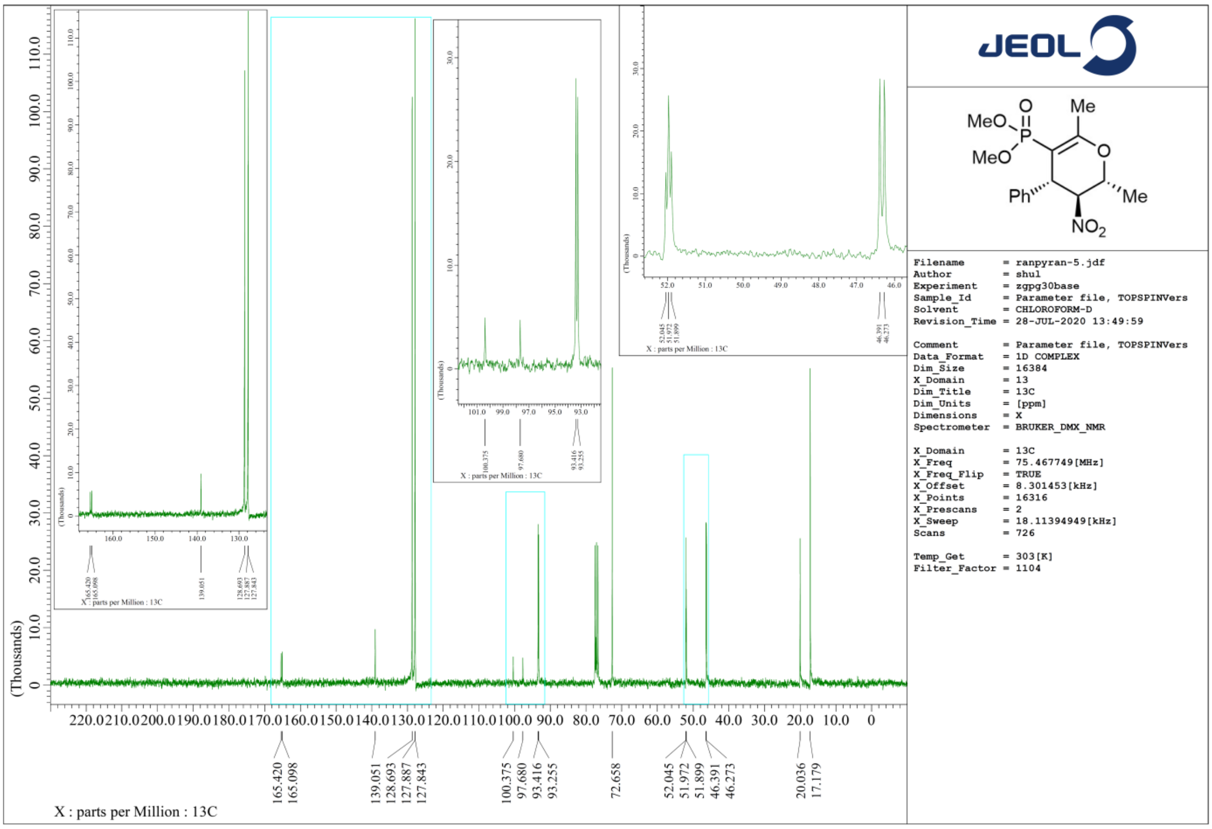

DEPT NMR spectra of dimethyl [(2*R*,3*S*,4*S*)-2,6-dimethyl-3-nitro-4-phenyl-3,4-dihydro-2*H*-pyran-5-yl]phosphonate (**14**) in $CDCl_3$

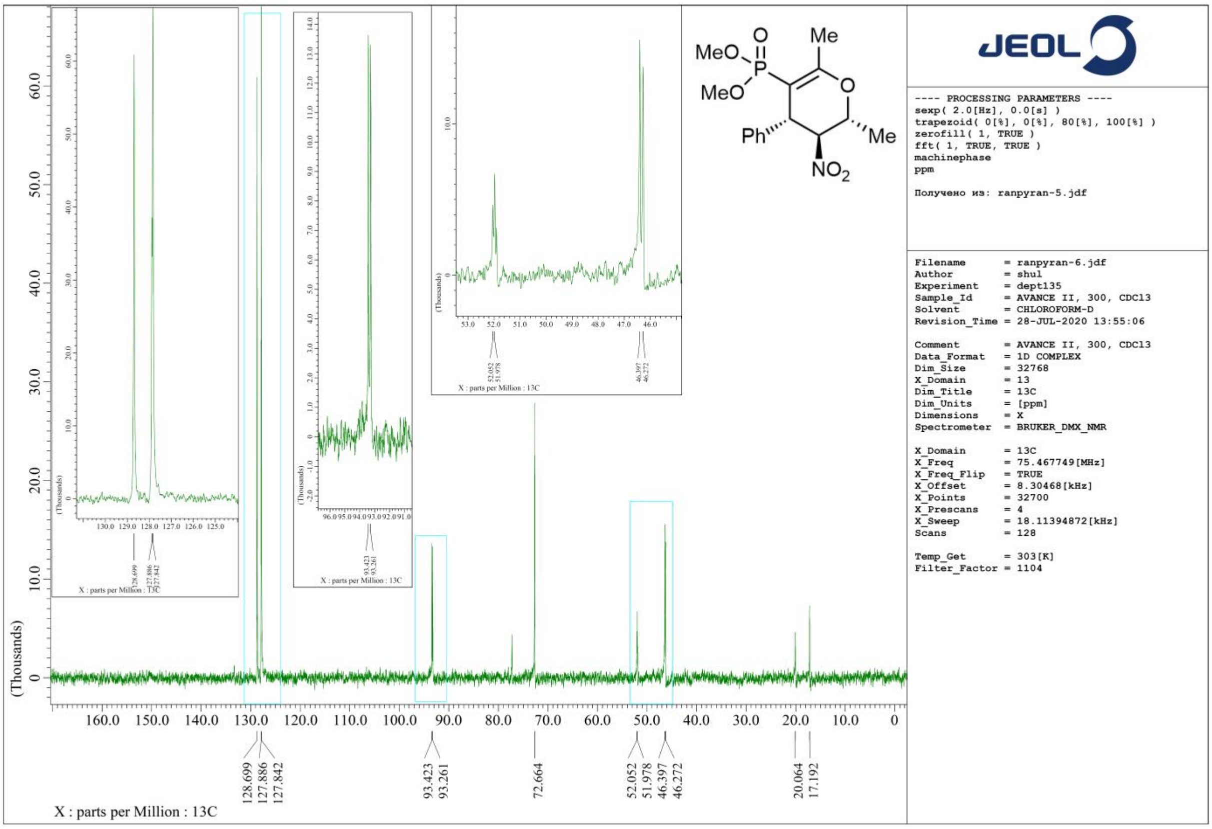

^{31}P NMR spectra of dimethyl [(2*R*,3*S*,4*S*)-2,6-dimethyl-3-nitro-4-phenyl-3,4-dihydro-2*H*-pyran-5-yl]phosphonate (**14**) in $CDCl_3$

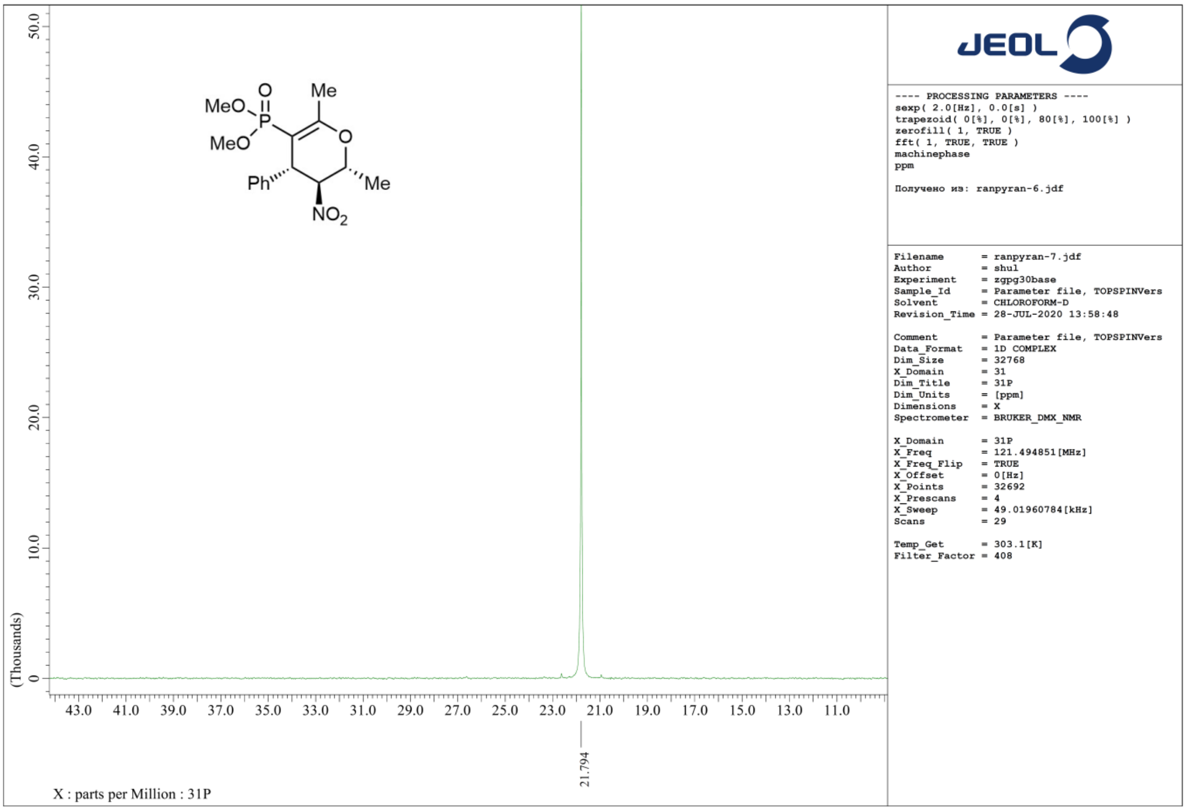

HRMS of dimethyl [(2*R*,3*S*,4*S*)-2,6-dimethyl-3-nitro-4-phenyl-3,4-dihydro-2*H*-pyran-5-yl]phosphonate (**14**)

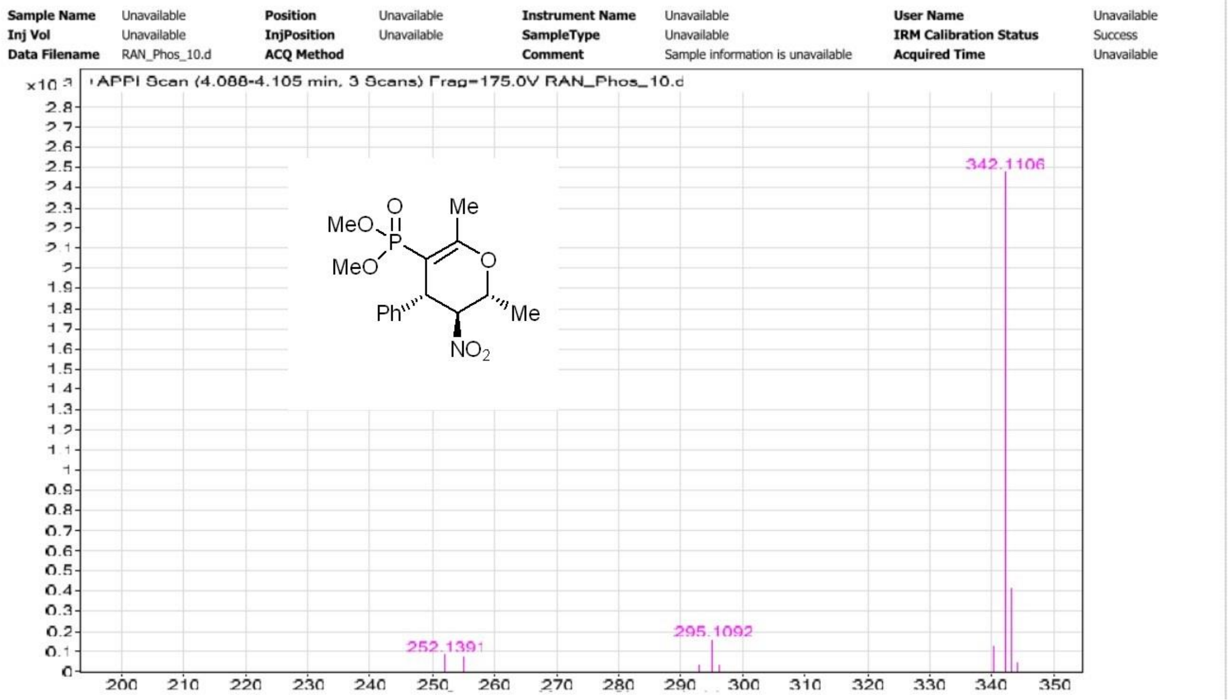

9. Copies of HPLC chromatograms for compounds 6a–d,f and 13b

HPLC for dimethyl [(2*R*,3*S*)-4-nitro-1-oxo-1,3-diphenylbutan-2-yl]phosphonate (**6a**)

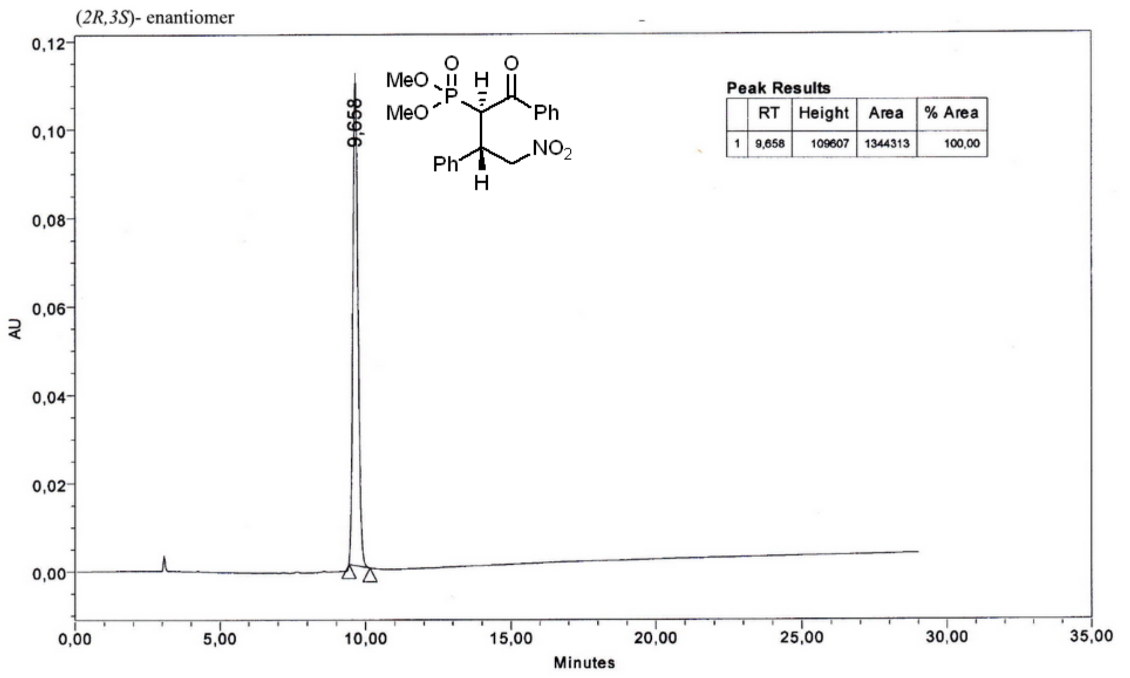

HPLC for dimethyl [(2*S*,3*R*)-4-nitro-1-oxo-1,3-diphenylbutan-2-yl]phosphonate (**6a**)

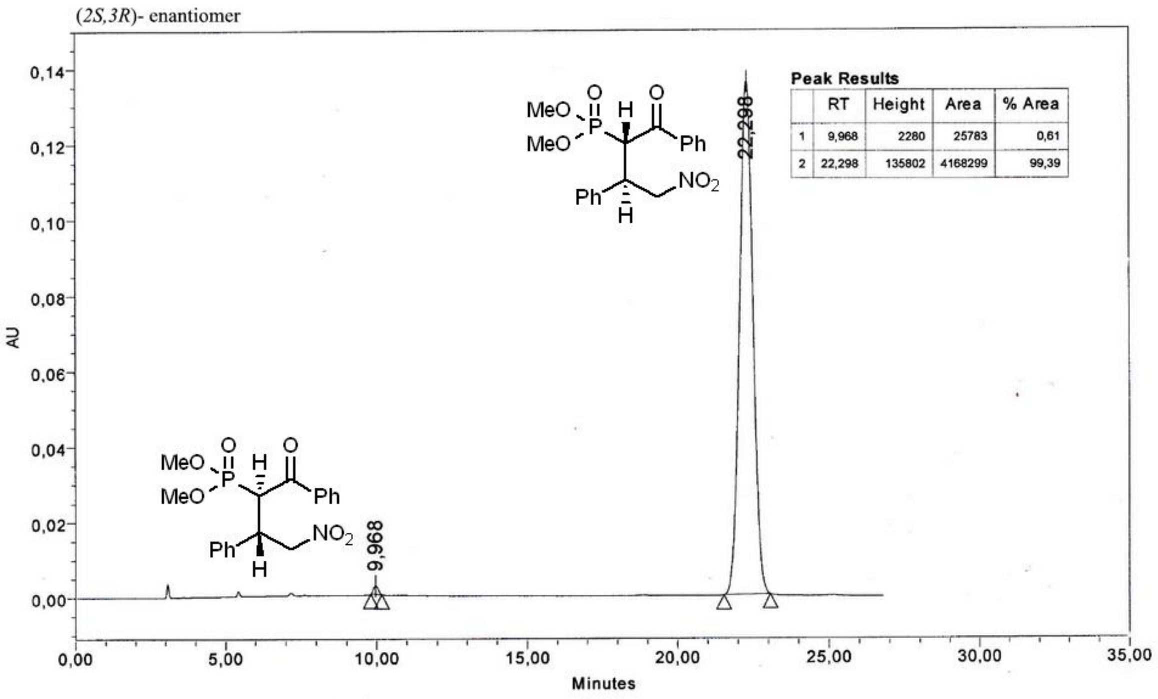

HPLC for mixture of (2*R*,3*S*)-**6a** and (2*S*,3*R*)-**6a** enantiomers

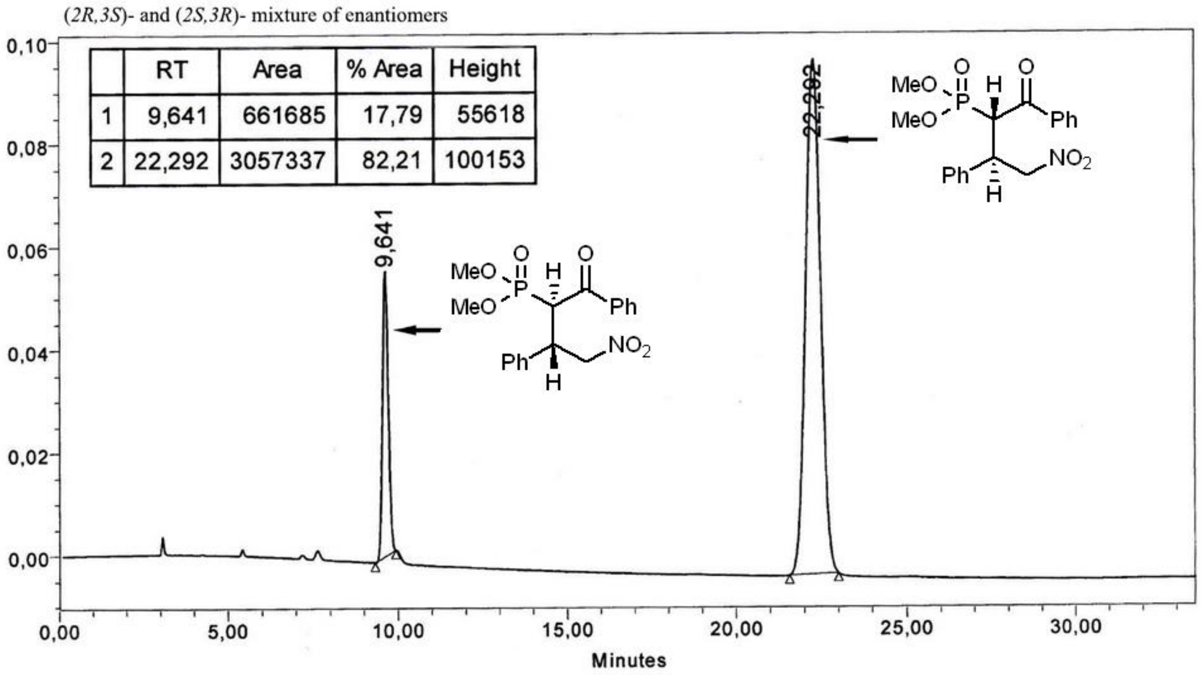

	RT	Area	% Area	Height
1	9,641	661685	17,79	55618
2	22,292	3057337	82,21	100153

HPLC for racemic dimethyl [1-(4-methoxyphenyl)-4-nitro-1-oxo-3-phenylbutan-2-yl]-phosphonate (**6b**)

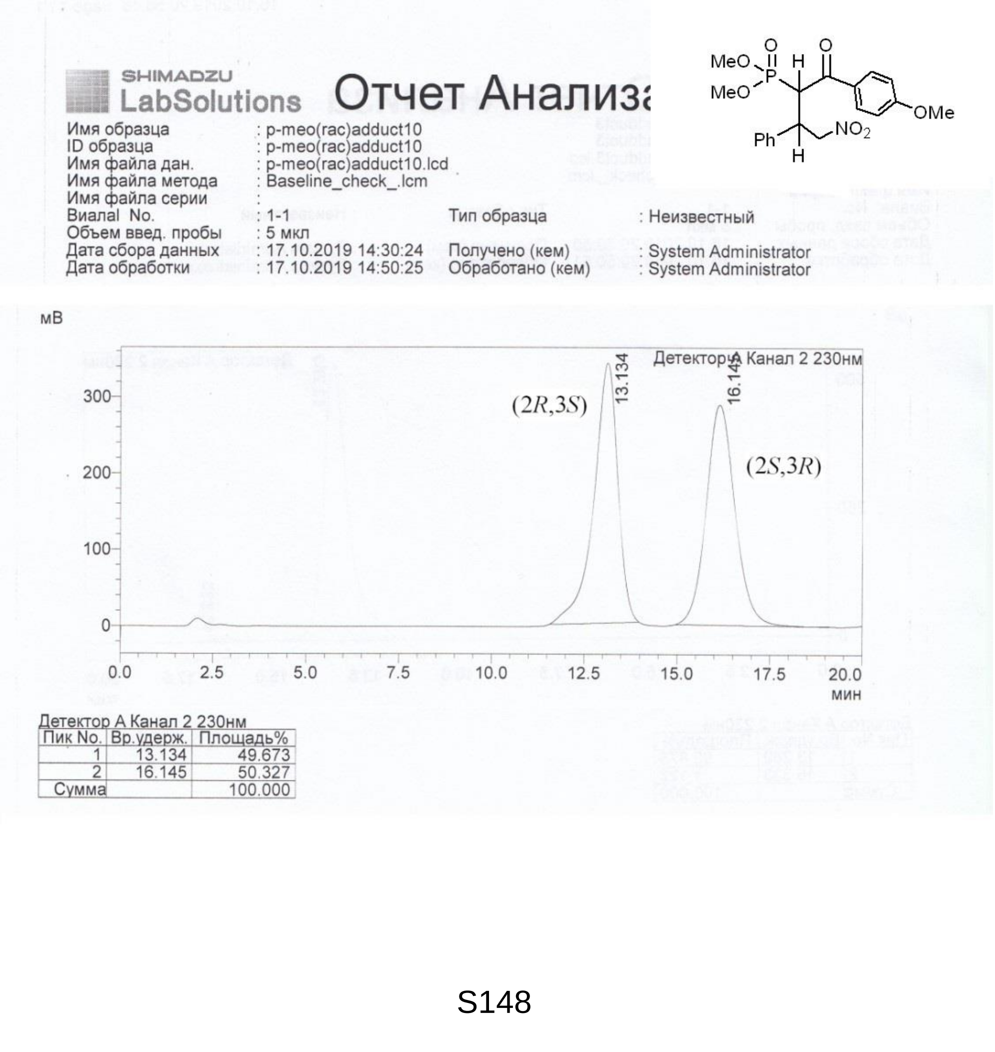

Пик No.	Вр.удерж.	Площадь%
1	13.134	49.673
2	16.145	50.327
Сумма		100.000

HPLC for dimethyl [(2*R*,3*S*)-1-(4-methoxyphenyl)-4-nitro-1-oxo-3-phenylbutan-2-yl]-phosphonate (**6b**)

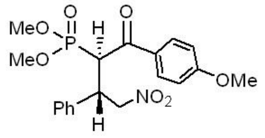

(2*R*,3*S*)

SHIMADZU LabSolutions **Отчет Анализа**

Имя образца	: p-meo adduct3		
ID образца	: p-meo adduct3		
Имя файла дан.	: p-meo adduct3.lcd		
Имя файла метода	: Baseline_check_.lcm		
Имя файла серии	:		
Виалal No.	: 1-1	Тип образца	: Неизвестный
Объем введ. пробы	: 5 мкл		
Дата сбора данных	: 16.10.2019 20:30:50	Получено (кем)	: System Administrator
Дата обработки	: 16.10.2019 20:50:51	Обработано (кем)	: System Administrator

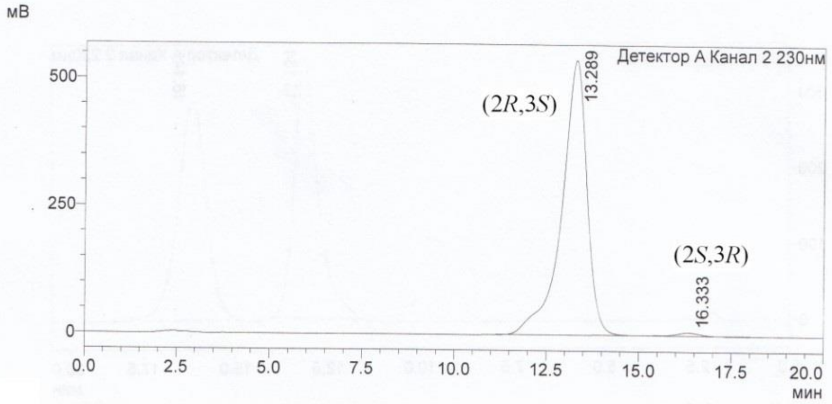

Детектор A Канал 2 230нм

Пик No.	Вр.удерж.	Площадь%
1	13.289	98.878
2	16.333	1.122
Сумма		100.000

HPLC for racemic dimethyl [1-(3-methoxyphenyl)-4-nitro-1-oxo-3-phenylbutan-2-yl]phosphonate (**6c**)

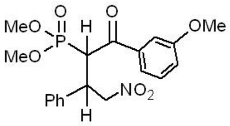

SHIMADZU LabSolutions **Отчет Анализа**

Имя образца	: m-meo(rac)Adduct4_20%130grad_AD120мин		
ID образца	: m-meo(rac)Adduct4_20%AD__120мин		
Имя файла дан.	: m-meo(rac)Adduct4_20%30gradAD_120мин.lcd		
Имя файла метода	: градиент1.lcm		
Имя файла серии	:		
Виалal No.	: 1-1	Тип образца	: Неизвестный
Объем введ. пробы	: 10 мкл		
Дата сбора данных	: 19.12.2019 17:00:43	Получено (кем)	: System Administrator
Дата обработки	: 19.12.2019 17:29:01	Обработано (кем)	: System Administrator

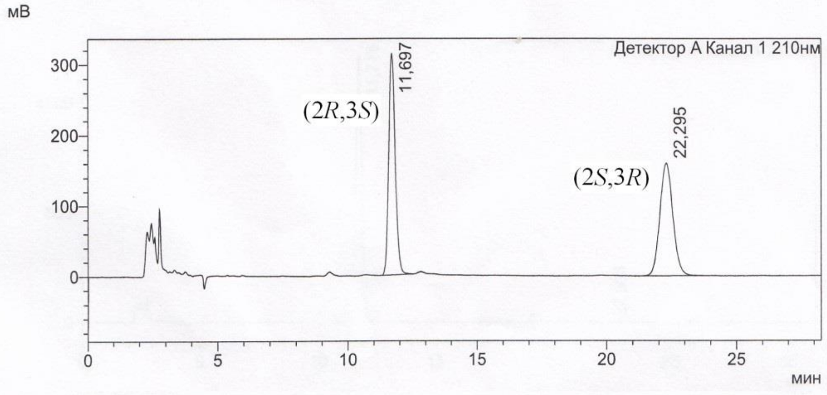

Детектор A Канал 1 210нм

Пик No.	Вр.удерж.	Площадь%
1	11,697	49,814
2	22,295	50,186
Сумма		100,000

HPLC for dimethyl [(2*R*,3*S*)-1-(3-methoxyphenyl)-4-nitro-1-oxo-3-phenylbutan-2-yl]phosphonate (**6c**)

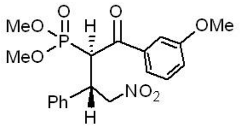

(2*R*,3*S*)

SHIMADZU LabSolutions **Отчет Анализа**

Имя образца : m-meo Adduct_20%130grad_AD120мин
ID образца : m-meo Adduct_20%AD__120мин
Имя файла дан. : m-meo Adduct_20%30gradAD_120мин.lcd
Имя файла метода : градиент1.lcm
Имя файла серии :
Виалал No. : 1-1 Тип образца : Неизвестный
Объем введ. пробы : 10 мкл
Дата сбора данных : 27.11.2019 15:05:59 Получено (кем) : System Administrator
Дата обработки : 27.11.2019 15:42:51 Обработано (кем) : System Administrator

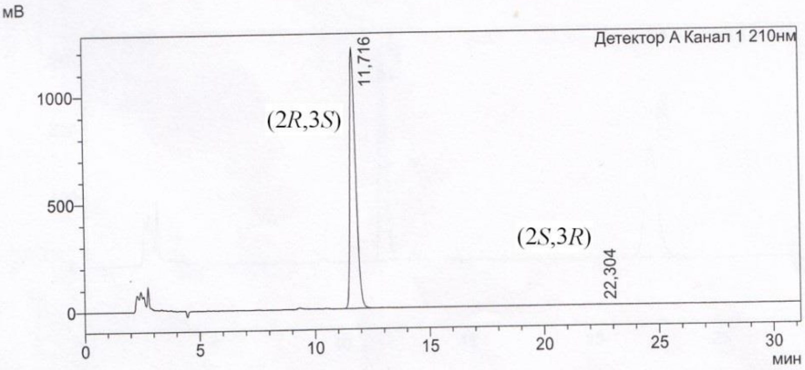

Детектор А Канал 1 210нм

Пик No.	Вр.удерж.	Площадь%
1	11,716	99,809
2	22,304	0,191
Сумма		100,000

HPLC for racemic dimethyl [1-(3-methoxyphenyl)-4-nitro-1-oxo-3-phenylbutan-2-yl]phosphonate

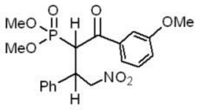

SHIMADZU LabSolutions **Отчет Анализа**

Имя образца : m-meo(rac)Adduct7_20%130grad_AD120мин
ID образца : m-meo(rac)Adduct7_20%AD__120мин
Имя файла дан. : m-meo(rac)Adduct7_20%30gradAD_120мин.lcd
Имя файла метода : градиент1.lcm
Имя файла серии :
Виалал No. : 1-1 Тип образца
Объем введ. пробы : 10 мкл
Дата сбора данных : 19.12.2019 18:55:30 Получено (кем)
Дата обработки : 19.12.2019 19:23:05 Обработано (кем)

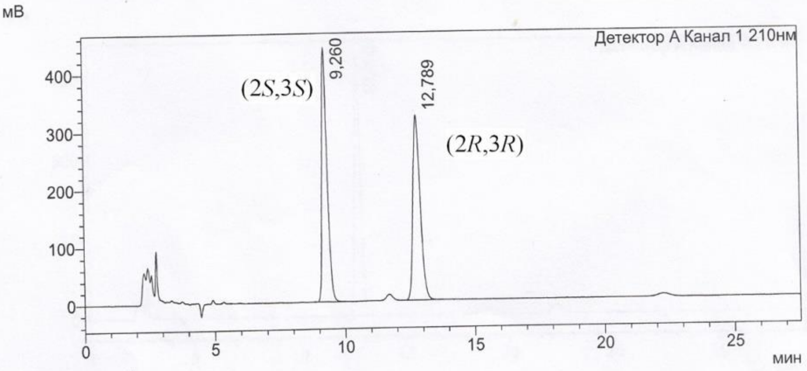

Детектор А Канал 1 210нм

Пик No.	Вр.удерж.	Площадь%
1	9,260	49,781
2	12,789	50,219
Сумма		100,000

HPLC for dimethyl [(2*R*,3*R*)-1-(3-methoxyphenyl)-4-nitro-1-oxo-3-phenylbutan-2-yl]phosphonate

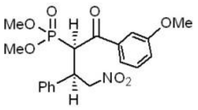

SHIMADZU LabSolutions **Отчет Анализ**

Имя образца	: m-meo Adduct6_20%130grad_AD120мин
ID образца	: m-meo Adduct6_20%AD__120мин
Имя файла дан.	: m-meo Adduct6_20%30gradAD_120мин.lcd
Имя файла метода	: градиент1.lcm
Имя файла серии	:
Виалал No.	: 1-1 Тип образца
Объем введ. пробы	: 10 мкл
Дата сбора данных	: 19.12.2019 18:17:52 Получено (кем)
Дата обработки	: 19.12.2019 19:02:54 Обработано (кем)

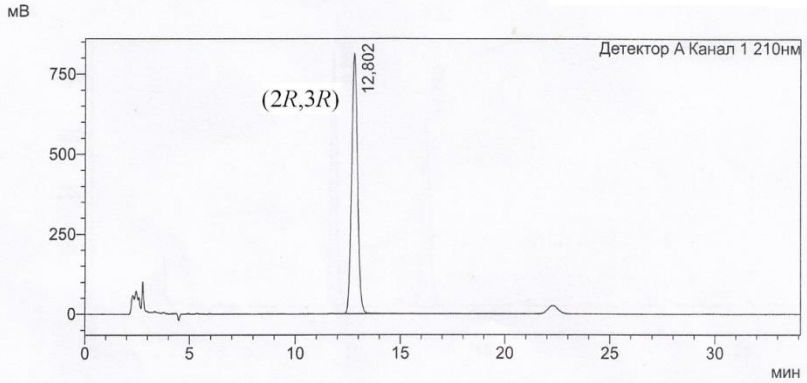

Детектор А Канал 1 210нм

Пик No.	Вр.удерж.	Площадь%
1	12,802	100,000
Сумма		100,000

HPLC for racemic dimethyl [1-(2-methoxyphenyl)-4-nitro-1-oxo-3-phenylbutan-2-yl]phosphonate

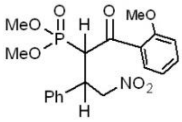

SHIMADZU LabSolutions **Отчет Анализа**

Имя образца	: O-meo(rac)Adduct_15%130grad_AD120мин
ID образца	: O-meo(rac)Adduct_15%AD__120мин
Имя файла дан.	: O-meo(rac)Adduct_15%30gradAD_120мин.lcd
Имя файла метода	: градиент1.lcm
Имя файла серии	:
Виалал No.	: 1-1 Тип образца : Неизвестный
Объем введ. пробы	: 10 мкл
Дата сбора данных	: 27.11.2019 17:50:08 Получено (кем) : System Administrator
Дата обработки	: 27.11.2019 18:32:54 Обработано (кем) : System Administrator

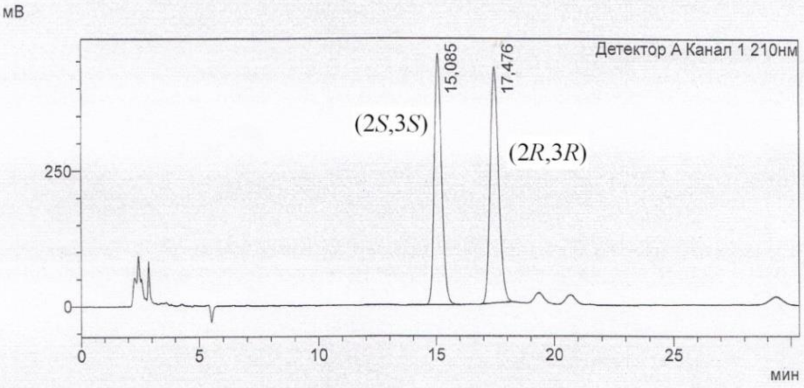

Детектор А Канал 1 210нм

Пик No.	Вр.удерж.	Площадь%
1	15,085	50,111
2	17,476	49,889
Сумма		100,000

HPLC for dimethyl [(2*S*,3*S*)-1-(2-methoxyphenyl)-4-nitro-1-oxo-3-phenylbutan-2-yl]phosphonate (**6d**)

SHIMADZU LabSolutions **Отчет Анализа**

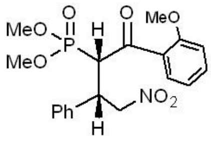

(2*S*,3*S*)

Имя образца	: O-meo)Adduct_15%130grad_AD120мин
ID образца	: O-meo Adduct_15%AD__120мин
Имя файла дан.	: O-meo Adduct_15%30gradAD_120мин.lcd
Имя файла метода	: градиент1.lcm
Имя файла серии	:
Виалал No.	: 1-1
Объем введ. пробы	: 10 мкл
Дата сбора данных	: 27.11.2019 17:19:48
Дата обработки	: 27.11.2019 17:47:39

Тип образца : Неизвестный
Получено (кем) : System Administrator
Обработано (кем) : System Administrator

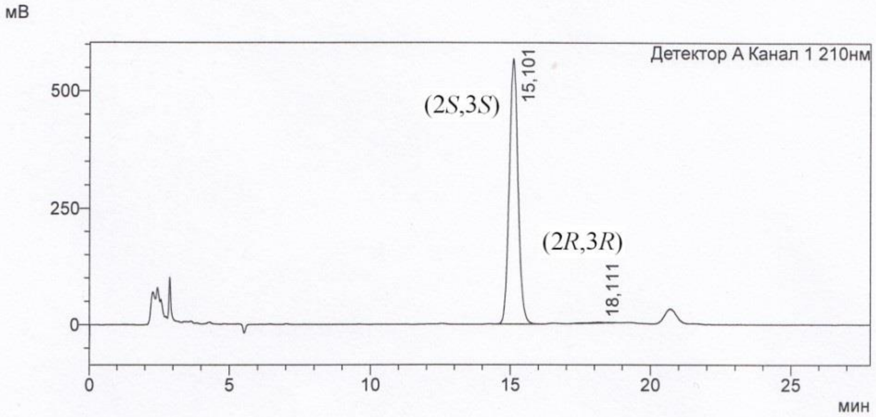

Детектор А Канал 1 210нм

Пик No.	Вр.удерж.	Площадь%
1	15,101	99,243
2	18,111	0,757
Сумма		100,000

HPLC for racemic dimethyl [1-(adamantan-1-yl)-4-nitro-1-oxo-3-phenylbutan-2-yl]phosphonate

SHIMADZU LabSolutions **Отчет Анализа**

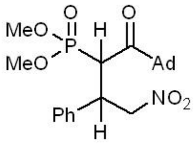

Имя образца	: Ad(rac)Adduct_10%30grad_AD120мин
ID образца	: Ad(rac)Adduct_10%AD__120мин
Имя файла дан.	: Ad(rac)Adduct_10%30gradAD_120мин.lcd
Имя файла метода	: градиент1.lcm
Имя файла серии	:
Виалал No.	: 1-1
Объем введ. пробы	: 10 мкл
Дата сбора данных	: 18.10.2019 14:03:37
Дата обработки	: 18.10.2019 14:56:06

Тип образца : Неизвестный
Получено (кем) : System Administrator
Обработано (кем) : System Administrator

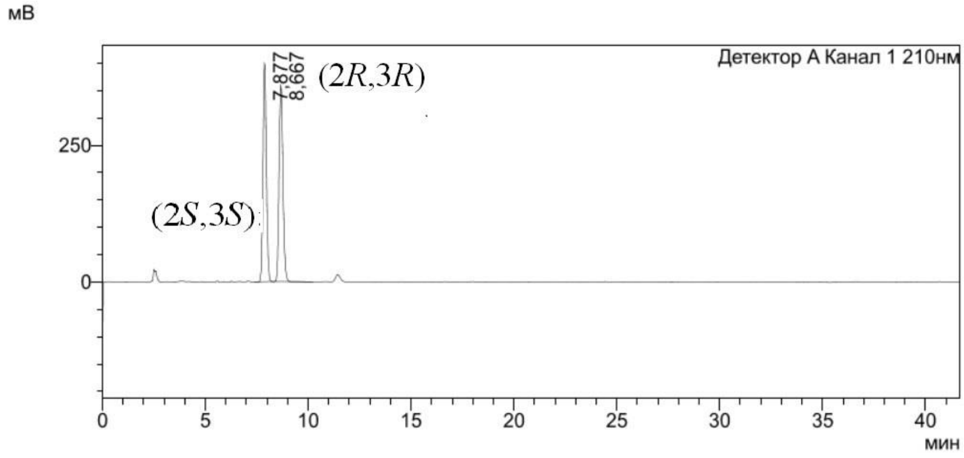

Детектор А Канал 1 210нм

Пик No.	Вр.удерж.	Площадь%
1	7,877	49,391
2	8,667	50,609
Сумма		100,000

HPLC for dimethyl [(2*S*,3*S*)-1-(adamantan-1-yl)-4-nitro-1-oxo-3-phenylbutan-2-yl]phosphonate (**6f**)

SHIMADZU LabSolutions **Отчет Анализа**

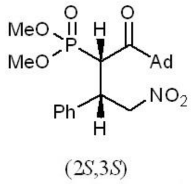

Имя образца	: Ad(rr-)Adduct1_10%30grad_AD120мин		
ID образца	: Ad(rr-)Adduct1_10%AD__120мин		
Имя файла дан.	: Ad(rr-)Adduct1_10%30gradAD_120мин.lcd		
Имя файла метода	: градиент1.lcm		
Имя файла серии	:		
Виалаl No.	: 1-1	Тип образца	: Неизвестный
Объем введ. пробы	: 10 мкл		
Дата сбора данных	: 18.10.2019 15:04:57	Получено (кем)	: System Administra
Дата обработки	: 18.10.2019 15:33:02	Обработано (кем)	: System Administra

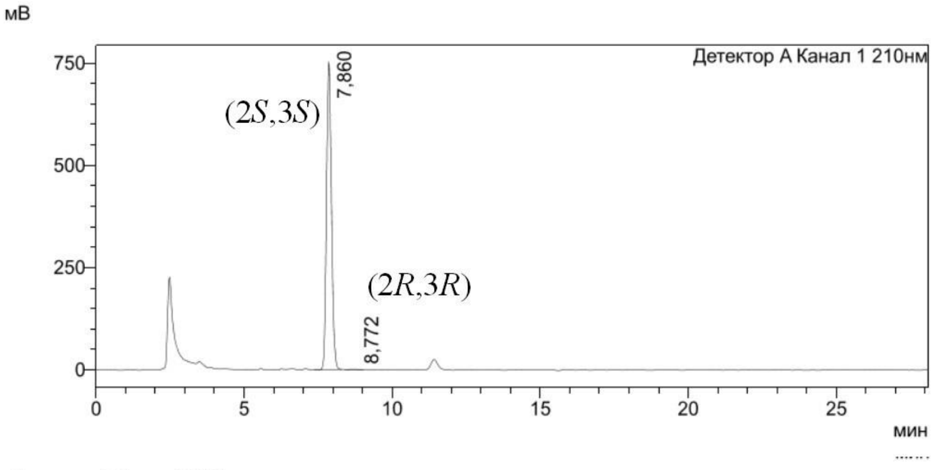

Детектор A Канал 1 210нм

Пик No.	Вр.удерж.	Площадь%
1	7,860	99,787
2	8,772	0,213
Сумма		100,000

HPLC for racemic dimethyl [2-hydroxy-2,6-dimethyl-5-nitro-4-phenyltetrahydro-2*H*-pyran-3-yl]phosphonate

SHIMADZU LabSolutions **Отчет Анализа**

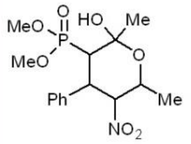

Имя образца	: tetrahydpyr_8%30grad_AD120мин		
ID образца	: tetrahydpyr_8%AD__120мин		
Имя файла дан.	: tetrahydpyr_8%30gradAD_120мин.lcd		
Имя файла метода	: градиент1.lcm		
Имя файла серии	:		
Виалаl No.	: 1-1	Тип образца	: Неизвестный
Объем введ. пробы	: 10 мкл		
Дата сбора данных	: 20.03.2020 19:51:10	Получено (кем)	: System Administrator
Дата обработки	: 20.03.2020 20:50:29	Обработано (кем)	: System Administrator

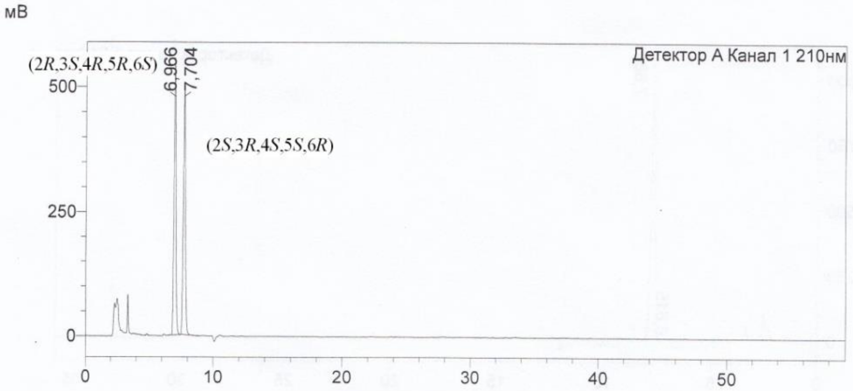

Детектор A Канал 1 210нм

Пик No.	Вр.удерж.	Площадь%
1	6,966	49,894
2	7,704	50,106
Сумма		100,000

HPLC for dimethyl [(2*S*,3*R*,4*S*,5*S*,6*R*)-2-hydroxy-2,6-dimethyl-5-nitro-4-phenyltetrahydro-2*H*-pyran-3-yl]phosphonate (**13b**)

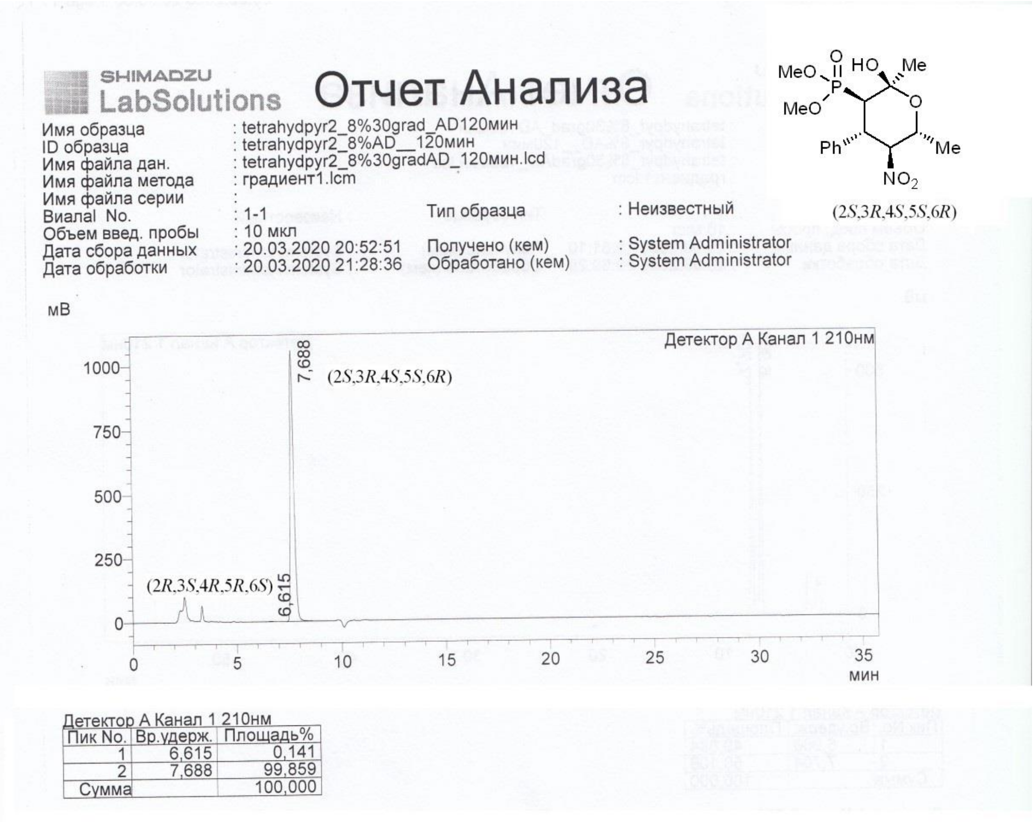
SHIMADZU LabSolutions

Отчет Анализа

Имя образца	: tetrahydpyr2_8%30grad_AD120мин
ID образца	: tetrahydpyr2_8%AD__120мин
Имя файла дан.	: tetrahydpyr2_8%30gradAD_120мин.lcd
Имя файла метода	: градиент1.lcm
Имя файла серии	:
Виалал No.	: 1-1
Объем введ. пробы	: 10 мкл
Дата сбора данных	: 20.03.2020 20:52:51
Дата обработки	: 20.03.2020 21:28:36
Тип образца	: Неизвестный
Получено (кем)	: System Administrator
Обработано (кем)	: System Administrator

Детектор А Канал 1 210нм

Пик No.	Вр.удерж.	Площадь%
1	6,615	0,141
2	7,688	99,859
Сумма		100,000

10. X-Ray diffraction data of compounds 10a and 13b

X-ray data for compounds **10a** and **13b** were obtained with a Enraf-Nonius CAD-4 diffractometer. CCDC 1919294 and 1846644 deposits contain the supplementary crystallographic data for this article [7,8]. These data can be obtained free of charge from The Cambridge Crystallographic Data Centre via http://www.ccdc.cam.ac.uk.

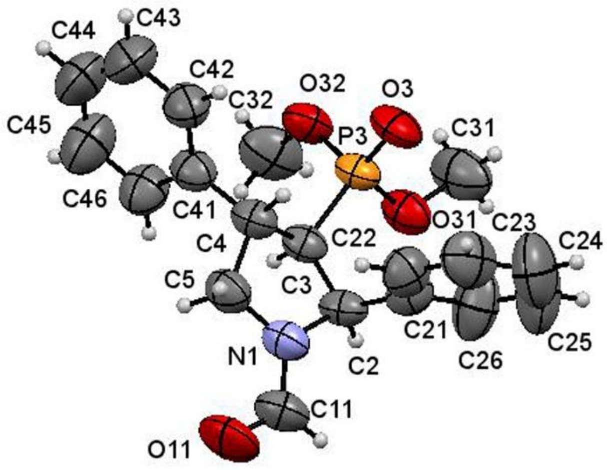

Table S2. X-ray diffraction data for dimethyl [(2*R*,3*R*,4*S*)-1-formyl-2,4-diphenylpyrrolidin-3-yl]phosphonate (10a) [7]

CCDC 1919294	
Empirical formula	$C_{19}H_{22}NO_4P$
Formula weight	359.34
Temperature	295(2) K
Wavelength	1.54186 Å (Cu*K*α)
Crystal system	monoclinic
Space group	$P\ 2_1$
Unit cell dimensions	a = 9.1358(13) Å α = 90°
	b = 10.4978(19) Å β = 96.830(10)°
	c = 13.5078(17) Å γ = 90°
Volume	1286.3(3) $Å^3$
Z	2
Density (calculated)	0.928 g/cm^3
Absorption coefficient	1.087 mm^{-1}
F(000)	380
Crystal size	0.020×0.020×0.020 mm

Theta range for data collection	4.875 to 74.995°
Index ranges	-11 ≤ *h* ≤ 11, -13 ≤ *k* ≤ 13, 0 ≤ *l* ≤ 16
Reflections collected	5235
Independent reflections	3276
Absorption correction	refdelf
Data / restraints / parameters	5235 / 1 / 202
Goodness–of–fit on F^2	1.058
Final R indices [I>2σ(I)]	R1 = 0.1174 wR2 = 0.2912
R indices (all data)	R1 = 0.1461 wR2 = 0.3231
Absolute structure (Flack) parameter	0.10(8)

Table S3. Selected bond lengths for compound 10a

№	Bond	Bondlength (Å)		№	Bond	Bondlength (Å)
**1**	N(1)-C(11)	1.344(13)		**25**	C(25)-C(26)	1.3900
**2**	N(1)-C(5)	1.444(14)		**26**	C(25)-H(25)	0.9300
**3**	N(1)-C(2)	1.464(12)		**27**	C(26)-H(26)	0.9300
**4**	C(2)-C(21)	1.507(11)		**28**	P(3)-O(3)	1.472(7)
**5**	C(2)-C(3)	1.521(13)		**29**	P(3)-O(32)	1.562(7)
**6**	C(2)-H(2)	0.9800		**30**	P(3)-O(31)	1.566(8)
**7**	C(3)-C(4)	1.533(13)		**31**	O(31)-C(31)	1.430(14)
**8**	C(3)-P(3)	1.803(9)		**32**	C(31)-H(31a)	0.9600
**9**	C(3)-H(3)	0.9800		**33**	C(31)-H(31b)	0.9600
**10**	C(4)-C(41)	1.503(12)		**34**	C(31)-H(31c)	0.9600
**11**	C(4)-C(5)	1.537(13)		**35**	O(32)-C(32)	1.447(17)
**12**	C(4)-H(4)	0.9800		**36**	C(32)-H(32a)	0.9600
**13**	C(5)-H(5a)	0.9700		**37**	C(32)-H(32b)	0.9600
**14**	C(5)-H(5b)	0.9700		**38**	C(32)-H(32c)	0.9600
**15**	C(11)-O(11)	1.234(15)		**39**	C(41)-C(46)	1.3900
**16**	C(11)-H(11)	0.9300		**40**	C(41)-C(42)	1.3900
**17**	C(21)-C(22)	1.3900		**41**	C(46)-C(45)	1.3900
**18**	C(21)-C(26)	1.3900		**42**	C(46)-H(46)	0.9300
**19**	C(22)-C(23)	1.3900		**43**	C(45)-C(44)	1.3900
**20**	C(22)-H(22)	0.9300		**44**	C(45)-H(45)	0.9300
**21**	C(23)-C(24)	1.3900		**45**	C(44)-C(43)	1.3900
**22**	C(23)-H(23)	0.9300		**46**	C(44)-H(44)	0.9300
**23**	C(24)-C(25)	1.3900		**47**	C(43)-C(42)	0.9300
**24**	C(24)-H(24)	0.9300		**48**	C(43)-H(43)	0.9300

Table S4. Selected bond angles for compound 10a

№	Angle	(°)		№	Angle	(°)
**1**	C(11)-N(1)-C(5)	123.3(10)		**44**	C(26)-C(25)-H(25)	120.0
**2**	C(11)-N(1)-C(2)	122.7(10)		**45**	C(24)-C(25)-H(25)	120.0
**3**	C(5)-N(1)-C(2)	113.4(8)		**46**	C(25)-C(26)-C(21)	120.0
**4**	N(1)-C(2)-C(21)	112.4(7)		**47**	C(25)-C(26)-H(26)	120.0
**5**	N(1)-C(2)-C(3)	100.2(8)		**48**	C(21)-C(26)-H(26)	120.0
**6**	C(21)-C(2)-C(3)	117.4(7)		**49**	O(3)-P(3)-O(32)	109.1(5)
**7**	N(1)-C(2)-H(2)	108.8		**50**	O(3)-P(3)-O(31)	114.9(5)
**8**	C(21)-C(2)-H(2)	108.8		**51**	O(32)-P(3)-O(31)	106.0(4)
**9**	C(3)-C(2)-H(2)	108.8		**52**	O(3)-P(3)-C(3)	116.6(4)
**10**	C(2)-C(3)-C(4)	104.9(7)		**53**	O(32)-P(3)-C(3)	107.5(4)
**11**	C(2)-C(3)-P(3)	117.8(7)		**54**	O(31)-P(3)-C(3)	101.9(4)
**12**	C(4)-C(3)-P(3)	112.9(6)		**55**	C(31)-O(31)-P(3)	119.7(8)
**13**	C(2)-C(3)-H(3)	106.9		**56**	O(31)-C(31)-H(31a)	109.5
**14**	C(4)-C(3)-H(3)	106.9		**57**	O(31)-C(31)-H(31b)	109.5
**15**	P(3)-C(3)-H(3)	106.9		**58**	H(31a)-C(31)-H(31b)	109.5
**16**	C(41)-C(4)-C(3)	117.2(8)		**59**	O(31)-C(31)-H(31c)	109.5
**17**	C(41)-C(4)-C(5)	112.2(8)		**60**	H(31a)-C(31)-H(31c)	109.5
**18**	C(3)-C(4)-C(5)	100.5(8)		**61**	H(31b)-C(31)-H(31c)	109.5
**19**	C(41)-C(4)-H(4)	108.8		**62**	C(32)-O(32)-P(3)	120.0(8)
**20**	C(3)-C(4)-H(4)	108.8		**63**	O(32)-C(32)-H(32a)	109.5
**21**	C(5)-C(4)-H(4)	108.8		**64**	O(32)-C(32)-H(32b)	109.5
**22**	N(1)-C(5)-C(4)	104.7(8)		**65**	H(32a)-C(32)-H(32b)	109.5
**23**	N(1)-C(5)-H(5a)	110.8		**66**	O(32)-C(32)-H(32c)	109.5
**24**	C(4)-C(5)-H(5a)	110.8		**67**	H(32a)-C(32)-H(32c)	109.5
**25**	N(1)-C(5)-H(5b)	110.8		**68**	H(32b)-C(32)-H(32c)	109.5
**26**	C(4)-C(5)-H(5b)	110.8		**69**	C(46)-C(41)-C(42)	120.0
**27**	H(5A)-C(5)-H(5b)	108.9		**70**	C(46)-C(41)-C(4)	121.5(6)
**28**	O(11)-C(11)-N(1)	121.8(13)		**71**	C(42)-C(41)-C(4)	118.5(6)
**29**	O(11)-C(11)-H(11)	119.1		**72**	C(41)-C(46)-C(45)	120.0
**30**	N(1)-C(11)-H(11)	119.1		**73**	C(41)-C(46)-H(46)	120.0
**31**	C(22)-C(21)-C(26)	120.0		**74**	C(45)-C(46)-H(46)	120.0
**32**	C(22)-C(21)-C(2)	122.0(6)		**75**	C(44)-C(45)-C(46)	120.0
**33**	C(26)-C(21)-C(2)	118.0(6)		**76**	C(44)-C(45)-H(45)	120.0
**34**	C(23)-C(22)-C(21)	120.0		**77**	C(46)-C(45)-H(45)	120.0
**35**	C(23)-C(22)-H(22)	120.0		**78**	C(43)-C(44)-C(45)	120.0
**36**	C(21)-C(22)-H(22)	120.0		**79**	C(43)-C(44)-H(44)	120.0
**37**	C(22)-C(23)-C(24)	120.0		**80**	C(45)-C(44)-H(44)	120.0
**38**	C(22)-C(23)-H(23)	120.0		**81**	C(44)-C(43)-C(42)	120.0
**39**	C(24)-C(23)-H(23)	120.0		**82**	C(44)-C(43)-H(43)	120.0
**40**	C(23)-C(24)-C(25)	120.0		**83**	C(42)-C(43)-H(43)	120.0
**41**	C(23)-C(24)-H(24)	120.0		**84**	C(43)-C(42)-C(41)	120.0

**42**	C(25)-C(24)-H(24)	120.0		**85**	C(43)-C(42)-H(42)	120.0
**43**	C(26)-C(25)-C(24)	120.0		**86**	C(41)-C(42)-H(42)	120.0

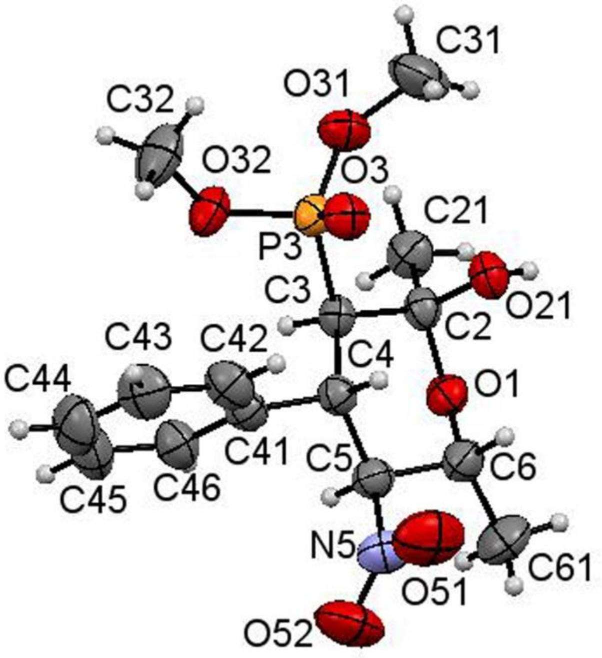

Table S5. X-ray diffraction data for dimethyl [(2*S*,3*R*,4*S*,5*S*,6*R*)-6-ethyl-2-hydroxy-2-methyl-5-nitro-4-phenyltetrahydro-2*H*-pyran-3-yl)phosphonate (13b) [8]

CCDC 1846644	
Empirical formula	$C_{15}H_{22}NO_7P$
Formula weight	359.31
Temperature	295(2) K
Wavelength	1.54184 Å
Crystal system	orthorhombic
Space group	$P\ 2_1\ 2_1\ 2_1$
Unit cell dimensions	a = 9.6607(9) Å α = 90°
	b = 11.0178(10) Å β = 90°
	c = 17.0785(17) Å γ = 90°
Volume	1817.8(3) Å^3
Z	4
Density (calculated)	1.313 g/cm^3
Absorption coefficient	1.660 mm^{-1}
F(000)	760
Crystal size	0.020×0.020×0.020 mm
Theta range for data collection	4.776 to 74.882°
Index ranges	$-11 \le h \le 12$, $-13 \le k \le 13$, $-20 \le l \le 21$
Reflections collected	3613
Independent reflections	3175

Absorption correction	refdelf
Data / restraints / parameters	3613 / 0 / 221
Goodness–of–fit on F^2	1.026
Final R indices [I>2σ(I)]	R1 = 0.0335 wR2 = 0.0800
R indices (all data)	R1 = 0.0402 wR2 = 0.0835
Absolute structure (Flack) parameter	0.001(15)

Table S6. Selected bond lengths for compound 13b

№	Bond	Bondlength (Å)	№	Bond	Bondlength (Å)
**1**	P(3)-O(3)	1.4634(18)	**25**	C(31)-H(31a)	0.9600
**2**	P(3)-O(31)	1.568(2)	**26**	C(31)-H(31b)	0.9600
**3**	P(3)-O(32)	1.5685(19)	**27**	C(31)-H(31c)	0.9600
**4**	P(3)-C(3)	1.825(3)	**28**	O(32)-C(32)	1.452(4)
**5**	O(1)-C(6)	1.423(3)	**29**	C(32)-H(32a)	0.9600
**6**	O(1)-C(2)	1.431(3)	**30**	C(32)-H(32b)	0.9600
**7**	C(2)-O(21)	1.401(3)	**31**	C(32)-H(32c)	0.9600
**8**	C(2)-C(21)	1.508(4)	**32**	C(41)-C(46)	1.386(4)
**9**	C(2)-C(3)	1.547(3)	**33**	C(41)-C(42)	1.388(4)
**10**	C(3)-C(4)	1.557(3)	**34**	C(42)-C(43)	1.388(4)
**11**	C(3)-H(3)	0.9800	**35**	C(42)-H(42)	0.9300
**12**	C(4)-C(41)	1.524(3)	**36**	C(43)-C(44)	1.379(5)
**13**	C(4)-C(5)	1.526(4)	**37**	C(43)-H(43)	0.9300
**14**	C(4)-H(4)	0.9800	**38**	C(44)-C(45)	1.371(5)
**15**	C(5)-N(5)	1.504(4)	**39**	C(44)-H(44)	0.9300
**16**	C(5)-C(6)	1.524(4)	**40**	C(45)-C(46)	1.391(4)
**17**	C(5)-H(5)	0.9800	**41**	C(45)-H(45)	0.9300
**18**	C(6)-C(61)	1.499(4)	**42**	C(46)-H(46)	0.9300
**19**	C(6)-H(6)	0.9800	**43**	N(5)-O(51)	1.214(4)
**20**	C(21)-H(21a)	0.9600	**44**	N(5)-O(52)	1.220(3)
**21**	C(21)-H(21b)	0.9600	**45**	C(61)-H(61a)	0.9600
**22**	C(21)-H(21c)	0.9600	**46**	C(61)-H(61b)	0.9600
**23**	O(21)-H(21)	0.79(3)	**47**	C(61)-H(61c)	0.9600
**24**	O(31)-C(31)	1.443(4)	**48**		

Table S7. Selected bond angles for compound 13b

№	Angle	(°)	№	Angle	(°)
**1**	O(3)-P(3)-O(31)	112.32(12)	**45**	C(31)-O(31)-P(3)	122.7(2)
**2**	O(3)-P(3)-O(32)	115.09(12)	**46**	O(31)-C(31)-H(31a)	109.5
**3**	O(31)-P(3)-O(32)	102.67(12)	**47**	O(31)-C(31)-H(31b)	109.5
**4**	O(3)-P(3)-C(3)	114.77(12)	**48**	H(31a)-C(31)-H(31b)	109.5
**5**	O(31)-P(3)-C(3)	110.55(12)	**49**	O(31)-C(31)-H(31c)	109.5
**6**	O(32)-P(3)-C(3)	100.22(11)	**50**	H(31a)-C(31)-H(31c)	109.5
**7**	C(6)-O(1)-C(2)	115.6(2)	**51**	H(31b)-C(31)-H(31c)	109.5
**8**	O(21)-C(2)-O(1)	110.1(2)	**52**	C(32)-O(32)-P(3)	118.4(2)
**9**	O(21)-C(2)-C(21)	113.6(2)	**53**	O(32)-C(32)-H(32a)	109.5
**10**	O(1)-C(2)-C(21)	103.8(2)	**54**	O(32)-C(32)-H(32b)	109.5
**11**	O(21)-C(2)-C(3)	106.4(2)	**55**	H(32a)-C(32)-H(32b)	109.5
**12**	O(1)-C(2)-C(3)	109.5(2)	**56**	O(32)-C(32)-H(32c)	109.5
**13**	C(21)-C(2)-C(3)	113.5(2)	**57**	H(32a)-C(32)-H(32c)	109.5

**14**	C(2)-C(3)-C(4)	114.1(2)	**58**	H(32b)-C(32)-H(32c)	109.5
**15**	C(2)-C(3)-P(3)	112.96(18)	**59**	C(46)-C(41)-C(42)	119.3(3)
**16**	C(4)-C(3)-P(3)	107.05(17)	**60**	C(46)-C(41)-C(4)	120.6(2)
**17**	C(2)-C(3)-H(3)	107.5	**61**	C(42)-C(41)-C(4)	120.1(3)
**18**	C(4)-C(3)-H(3)	107.5	**62**	C(43)-C(42)-C(41)	120.3(3)
**19**	P(3)-C(3)-H(3)	107.5	**63**	C(43)-C(42)-H(42)	119.9
**20**	C(41)-C(4)-C(5)	112.4(2)	**64**	C(41)-C(42)-H(42)	119.9
**21**	C(41)-C(4)-C(3)	111.5(2)	**65**	C(44)-C(43)-C(42)	120.1(3)
**22**	C(5)-C(4)-C(3)	109.2(2)	**66**	C(44)-C(43)-H(43)	120.0
**23**	C(41)-C(4)-H(4)	107.9	**67**	C(42)-C(43)-H(43)	120.0
**24**	C(5)-C(4)-H(4)	107.9	**68**	C(45)-C(44)-C(43)	119.9(3)
**25**	C(3)-C(4)-H(4)	107.9	**69**	C(45)-C(44)-H(44)	120.1
**26**	N(5)-C(5)-C(6)	108.5(2)	**70**	C(43)-C(44)-H(44)	120.1
**27**	N(5)-C(5)-C(4)	109.7(2)	**71**	C(44)-C(45)-C(46)	120.6(3)
**28**	C(6)-C(5)-C(4)	111.2(2)	**72**	C(44)-C(45)-H(45)	119.7
**29**	N(5)-C(5)-H(5)	109.1	**73**	C(46)-C(45)-H(45)	119.7
**30**	C(6)-C(5)-H(5)	109.1	**74**	C(41)-C(46)-C(45)	119.9(3)
**31**	C(4)-C(5)-H(5)	109.1	**75**	C(41)-C(46)-H(46)	120.1
**32**	O(1)-C(6)-C(61)	107.3(3)	**76**	C(45)-C(46)-H(46)	120.1
**33**	O(1)-C(6)-C(5)	105.6(2)	**77**	O(51)-N(5)-O(52)	124.2(3)
**34**	C(61)-C(6)-C(5)	114.4(3)	**78**	O(51)-N(5)-C(5)	118.3(3)
**35**	O(1)-C(6)-H(6)	109.8	**79**	O(52)-N(5)-C(5)	117.5(3)
**36**	C(61)-C(6)-H(6)	109.8	**80**	C(6)-C(61)-H(61a)	109.5
**37**	C(5)-C(6)-H(6)	109.8	**81**	C(6)-C(61)-H(61b)	109.5
**38**	C(2)-C(21)-H(21a)	109.5	**82**	H(61a)-C(61)-H(61b)	109.5
**39**	C(2)-C(21)-H(21b)	109.5	**83**	C(6)-C(61)-H(61c)	109.5
**40**	C(2)-C(21)-H(21c)	109.5	**84**	H(61a)-C(61)-H(61c)	109.5
**41**	H(21a)-C(21)-H(21b)	109.5	**85**	H(61b)-C(61)-H(61c)	109.5
**42**	H(21a)-C(21)-H(21c)	109.5			
**43**	H(21b)-C(21)-H(21c)	109.5			
**44**	C(2)-O(21)-H(21)	100(2)			

References

1. Du, T.; Du, F.; Ning, Y.; Peng Y. *Org*. *Lett*. **2015**, *17*, 1308.

2. Reznikov, A. N.; Sibiryakova, A. E.; Klimochkin, Yu. N. *Russ*. *J*. *Gen*. *Chem*. **2014**, *84*, 2280.

3. Corbel, B.; Medinger, L.; Haelters, J. P.; Sturtz, G. *Synthesis* **1985**, 1048.

4. Evans, D. A.; Mito, S.; Seidel, D. *J*. *Am*. *Chem*. *Soc*. **2007**, *129*, 11583.

5. Reznikov, A. N.; Sibiryakova, A. E.; Rybakov, V. B.; Klimochkin, Yu. N. *Tetrahedron*: *Asymmetry* **2015**, *26*, 1050.

6. Reznikov, A. N.; Sibiryakova, A. E.; Baimuratov, M. R.; Golovin, E. V.; Rybakov, V. B.; Klimochkin, Yu. N. *Beilstein J*. *Org*. *Chem*. **2019**, *15*, 1289.

7. Reznikov, A. N.; Nikerov, D. S.; Sibiryakova, A. E.; Rybakov, V. B.; Golovin, E. V.; Klimochkin, Yu. N. CCDC 1919294: Experimental Crystal Structure Determination. *CSD Commun.*, 2020. doi: 10.5517/ccdc.csd.cc22f5qf

8. Reznikov, A. N.; Nikerov, D. S.; Sibiryakova, A. E.; Rybakov, V. B.; Golovin, E. V.; Klimochkin, Yu. N. CCDC 1846644: Experimental Crystal Structure Determination. *CSD Commun.*, 2018. doi: 10.5517/ccdc.csd.cc1zzl5q
